# Global Analysis of Genes Essential for Francisella tularensis Schu S4 Growth *In Vitro* and for Fitness during Competitive Infection of Fischer 344 Rats

**DOI:** 10.1128/JB.00630-18

**Published:** 2019-03-13

**Authors:** Philip M. Ireland, Helen L. Bullifent, Nicola J. Senior, Stephanie J. Southern, Zheng Rong Yang, Rachel E. Ireland, Michelle Nelson, Helen S. Atkins, Richard W. Titball, Andrew E. Scott

**Affiliations:** a Defence Science and Technology Laboratory, Chemical, Biological and Radiological Division, Salisbury, United Kingdom; b College of Life and Environmental Sciences, University of Exeter, Exeter, United Kingdom; c Department of Pathogen Molecular Biology, London School of Hygiene and Tropical Medicine, London, United Kingdom; Michigan State University

**Keywords:** essential genes, *Francisella*, gene essentiality, TraDIS, transposon insertion sequencing, genomics, virulence

## Abstract

The highly virulent intracellular pathogen Francisella tularensis is a Gram-negative bacterium that has a wide host range, including humans, and is the causative agent of tularemia. To identify new therapeutic drug targets and vaccine candidates and investigate the genetic basis of *Francisella* virulence in the Fischer 344 rat, we have constructed an F. tularensis Schu S4 transposon library. This library consists of more than 300,000 unique transposon mutants and represents a transposon insertion for every 6 bp of the genome. A transposon-directed insertion site sequencing (TraDIS) approach was used to identify 453 genes essential for growth *in vitro*. Many of these essential genes were mapped to key metabolic pathways, including glycolysis/gluconeogenesis, peptidoglycan synthesis, fatty acid biosynthesis, and the tricarboxylic acid (TCA) cycle. Additionally, 163 genes were identified as required for fitness during colonization of the Fischer 344 rat spleen. This *in vivo* selection screen was validated through the generation of marked deletion mutants that were individually assessed within a competitive index study against the wild-type F. tularensis Schu S4 strain.

**IMPORTANCE** The intracellular bacterial pathogen Francisella tularensis causes a disease in humans characterized by the rapid onset of nonspecific symptoms such as swollen lymph glands, fever, and headaches. F. tularensis is one of the most infectious bacteria known and following pulmonary exposure can have a mortality rate exceeding 50% if left untreated. The low infectious dose of this organism and concerns surrounding its potential as a biological weapon have heightened the need for effective and safe therapies. To expand the repertoire of targets for therapeutic development, we initiated a genome-wide analysis. This study has identified genes that are important for F. tularensis under *in vitro* and *in vivo* conditions, providing candidates that can be evaluated for vaccine or antibacterial development.

## Abstract (precis)

The intracellular bacterial pathogen Francisella tularensis causes a disease in humans characterized by the rapid onset of nonspecific symptoms such as swollen lymph glands, fever, and headaches. F. tularensis is one of the most infectious bacteria known and following pulmonary exposure can have a mortality rate exceeding 50% if left untreated. The low infectious dose of this organism and concerns surrounding its potential as a biological weapon have heightened the need for effective and safe therapies. To expand the repertoire of targets for therapeutic development, we initiated a genome-wide analysis. This study has identified genes that are important for F. tularensis under *in vitro* and *in vivo* conditions, providing candidates that can be evaluated for vaccine or antibacterial development.

## INTRODUCTION

Tularemia is a disease caused by Francisella tularensis, a highly infectious Gram-negative bacterium and member of the gamma subgroup of proteobacteria. F. tularensis can infect a wide range of invertebrates, birds, and mammals, including humans (1). Three subspecies have been identified: Francisella tularensis subsp. *tularensis* occurs in North America, the less virulent Francisella tularensis subsp. *holarctica* is found in North America and Europe, and Francisella tularensis subsp. *mediasiatica* is found in central Asia. The route of infection influences the clinical presentation of the disease, which can be classified as ulceroglandular, glandular, pneumonic, oculoglandular, oropharyngeal, or typhoidal (2). F. tularensis spreads systemically from the regional lymph to organs, including the liver, lungs, and spleen. Symptoms of infection can be nonspecific and include fever, malaise, chills, and headaches (3). Pneumonic infection often produces the most severe disease presentation, and a fatality rate that can exceed 50% without treatment (3), leading to concerns surrounding its potential for biological warfare (4).

The infectious dose of F. tularensis is as low as 25 organisms (5). Therefore, it is important that treatment regimens completely clear the bacterial infection upon completion of the therapy. A number of antibiotics are effective for treatment of tularemia, including streptomycin, gentamicin, tetracycline, chloramphenicol, doxycycline, rifampin, and ciprofloxacin (6–8). The potential for relapse following treatment, which can range from 6% to 21% (8–10), and the high toxicity of some antibiotics (11, 12) provide a need for improved therapies.

There is no vaccine licensed for F. tularensis infection. The live vaccine strain (LVS), an attenuated F. tularensis subsp. *holarctica* isolate, has previously shown to partially protect humans against aerosol challenge with the highly virulent F. tularensis subsp. *tularensis* Schu S4 (13). The genomic changes that contribute to the attenuation of the LVS are not fully understood and have in part prevented licensure. Additionally, numerous live vaccine strain candidates have been constructed through deletion of genes required for metabolism or virulence (14). However, potential reversion of single deletion mutant strains back to wild-type virulence, although of low probability, has led to the view that multiple genetic deletions should be considered (14). Consequently, as the number of genetic changes increases, so does the potential to overattenuate the candidate strain with potential loss of immunogenicity (15). Generating live vaccine strains with multiple lesions therefore continues to pose significant challenge.

A range of virulence factors utilized by F. tularensis to facilitate entry and survival in a host are known. These virulence genes include, for example, the intracellular growth locus (16), *mglA* and *mglB* (17), type IV pilus genes (18), lipopolysaccharide (LPS) genes (19), and capsule genes (20). F. tularensis is able to evade the immune response and, through inhibition of endosome-lysosome fusion, escape and replicate in the macrophage cystol. However, current understanding of F. tularensis virulence stems largely from studies using murine models of infection and *in vitro* cell lines. The use of surrogate strains that can be handled safely at lower levels of laboratory containment, such as the LVS or Francisella novicida, has vastly accelerated developments within the field. Nevertheless, despite causing lethality in murine models, these strains are not virulent in humans (F. novicida infection has been reported with near-drowning incidents and immunocompromised individuals [21, 22]). Thus, studies with F. tularensis Schu S4 and similar lethal strains are crucial to complement work with surrogate strains and increase our understanding of the virulence of this pathogen in humans.

Transposon-based mutagenesis is an effective and increasingly utilized approach to evaluate the impact of a gene on the viability or fitness of bacteria through insertional inactivation. Recent advances in high-throughput sequencing have facilitated techniques such as insertion sequencing (INseq) (23), transposon-directed insertion site sequencing (TraDIS) (24), transposon sequencing (Tn-seq) (25), high-throughput insertion tracking by deep sequencing (HITS) (26), and barcode analysis by sequencing (Bar-seq) (27), that enable the selective enrichment of transposon-genome junctions from saturating transposon libraries. Analysis of transposon insertion frequency across a genome can provide an assessment of all genes under specific conditions at high resolution. The minimal set of genes required under nutritious conditions provides important information regarding key biochemical processes within the organism. However, the essentiality or fitness contribution of a gene largely depends on the context of the environment inhabited by the organism. Therefore, for the purpose of therapeutic development, the identification of genes that are required for fitness of a pathogen in a host can offer an alternative strategy for antibacterial or vaccine development.

Here we describe the generation and analysis of an F. tularensis Schu S4 transposon library. We have used this library to carry out a global analysis of essential genes and genes which contribute to spleen colonization within Fischer 344 rats. The data generated provide a resource that can allow further investigations into the mechanisms of *Francisella* virulence. Additionally, these data can facilitate the rational design of live attenuated vaccine strains and novel antimicrobial targets for the prevention and treatment of tularemia.

## RESULTS

### Construction and sequencing of a saturated F. tularensis Schu S4 transposon library.

An F. tularensis Schu S4 library was constructed using the pHimarH3 transposon (28). Sequencing of transposon-genome junctions that had been selectively enriched by PCR generated approximately 15 million reads for each replicate, of which 92% to 96% mapped to the F. tularensis Schu S4 genome (Table 1). The EMBOSS fuzznuc application (29) was used to identify the existence of 414,318 potential Himar1 insertion site recognition sequences (TA) on the forward and complement strands of the F. tularensis Schu S4 genome. Therefore, a mean mutant library size of 323,702 would indicate that 78% of Himar1 recognition sites in the F. tularensis library genome contained transposon insertions.

**TABLE 1:** Summary of PCR-amplified transposon-genome junction sequencing reads mapped to the F. tularensis Schu S4 genome following outgrowth of the library in MMH broth

Replicate	No. of total reads	No. of reads mapped	% mapped^*a*^	No. of UISs^*b*^	Genome length/UIS
FT1	15,312,503	14,527,401	96.41	316,141	5.99
FT2	15,952,998	14,459,470	92.18	330,262	5.73
FT3	14,392,221	13,420,403	94.63	324,704	5.83

a Determined from only the reads containing the transposon tag.

b UIS, unique insertion sites.

### Prediction of genes essential to F. tularensis Schu S4.

**FIG 1:**
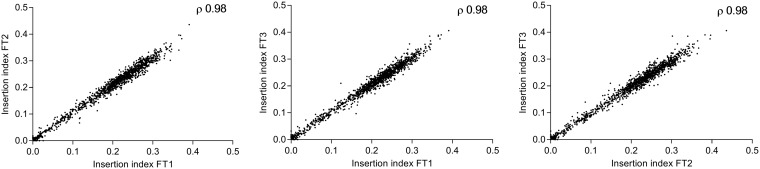
Pairwise comparison of gene insertion index values. Reproducibility of sequencing data was demonstrated by calculating the Spearman rank correlation coefficient between biological replicates.

The use of transposons to identify genes that are required for growth under the test condition can enable identification of those genes that are unable to tolerate transposon insertions and are likely to be essential for bacterial growth or survival. The Bio::Tradis pipeline (30, 31) was used to analyze mapped reads and predict genes essential for F. tularensis Schu S4. Following mapping of PCR-enriched transposon-genome junctions, the insertion count per gene for each replicate was tallied and normalized for gene length (insertion count/gene length) to calculate the insertion index. The insertion index for each was compared across three biological replicates as a scatter plot (Fig. 1) to give a high correlation coefficient, 0.98. We determined that the TA count per gene was highly correlated to gene length, with a Spearman *r* value of 0.97 and a 95% confidence interval of 0.96 to 0.97, thereby validating gene length a suitable normalizing factor for insertion count in the F. tularensis genome.

**FIG 2:**
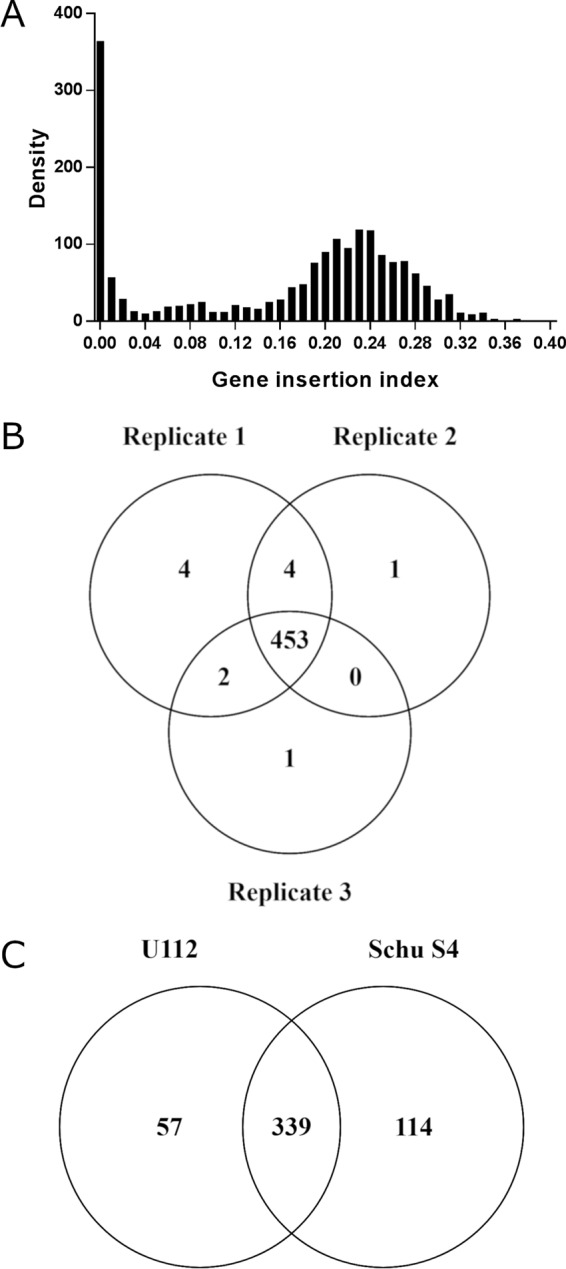
Prediction of gene essentiality from transposon libraries in F. tularensis Schu S4. (A) Insertion index plotted against density demonstrates a bimodal distribution (representative, FT1 replicate). (B) Essentiality analysis was performed on each replicate, identifying 453 genes that were present in all three. (C) Comparison of essential genes from both F. novicida U112 (32) and F. tularensis Schu S4 (this study) identified 339 predicted essential genes that are common to both species.

To differentiate the essential from nonessential genes, the insertion index for each gene was plotted by frequency (Fig. 2A). This revealed a bimodal distribution, and the minimal value between the two modes was calculated. Analysis of F. tularensis Schu S4 transposon libraries in this study using the Bio::Tradis tools predicted genes with an insertion index of <0.06 as essential. Gene essentiality was predicted for each of the replicate libraries, with 453 genes present in all three (Fig. 2B). The raw data for each replicate are available in Data Set S1 in the supplemental material and include the essential gene sequences in fastA format. Genes identified as essential mapped to a number of biochemical pathways using the Kyoto Encyclopedia of Genes and Genomes (KEGG) resource (http://www.genome.jp/kegg/), including glycolysis/gluconeogenesis, the tricarboxylic acid (TCA) cycle, fatty acid biosynthesis, and peptidoglycan synthesis (Fig. S1).

Previously 396 candidate essential genes were identified in the related nonvirulent species F. novicida U112 (32). F. tularensis Schu S4 genes predicted to be essential were used in a BLASTx search (expected cutoff, 1E−10) against the F. novicida U112 data set (32) utilizing the Database for Essential Genes (DEG) (http://tubic.tju.edu.cn/deg/) (33). This identified 339 genes from F. tularensis Schu S4 that were also found to be essential in F. novicida U112 (Fig. 2C). Additionally, a total of 57 genes were identified as essential in F. novicida U112 but not essential in F. tularensis Schu S4.

The F. tularensis Schu S4 genes for which no homologue was predicted as essential in F. novicida U112 (114 genes [Fig. 2C]) were used in a BLASTx analysis against the remaining 45 bacterial essential gene data sets present in the DEG. This analysis identified 25 putative F. tularensis Schu S4 essential genes that had no homologue. To limit the possibility of falsely identifying novel essential genes, as a consequence of either a small gene returning an expected value higher than the cutoff or genes with areas of low complexity, the DEG BLASTx analysis was repeated on these 25 genes, this time increasing the expected value cutoff to 1E−05 and without using the low-complexity filter. Nine genes returned hits, including FTT_0346 (*rpmJ*), FTT_0801c, FTT_0839 (*tolA*), FTT_1030, FTT_1275 (*mglA*), FTT_1453c (*wzx*), FTT_1457c (*wbtG*), FTT_1607 (*minE*), and FTT_1730c, and were therefore excluded from the novel essential gene list.

### BLASTx analysis of F. tularensis Schu S4 non-DEG homologues.

In order to give a suggestion of function for the 16 F. tularensis Schu S4 genes for which no DEG homologues had been identified (Table 2), a similarity search was performed against the NCBI nonredundant protein database, but excluding sequences from the *Francisella* genus using BLASTx with an expected value threshold of 1E−05. Many of the F. tularensis essential genes consistently revealed homology to hypothetical genes found in a wide range of environmental organisms often isolated from marine environments. Three hypothetical genes, FTT_0913, FTT_1051c, and FTT_1113c, showed no significant homology to any sequences in the nonredundant database. However, it should be noted that analysis of the TA site distribution across all genes within the F. tularensis genome identified a single gene outlier (FTT_1051c) with a disproportionally lower number of TA sites (10 sites) relative to its length. BLASTx and domain analyses and structural analysis using the I-Tasser protein structure and function prediction tool (34) were performed on the 16 genes of interest (Data Set S2). A number of conserved domains were identified, including a redoxin domain identified in FTT_0557 (domain E value, 1.20E−22) that also showed structural homology (TM scores, 0.97 to 0.855) to peroxiredoxin. A homologue for FTT_0696 was not identified in the DEG despite the presence of an FtsL pfam (35) conserved domain (domain E value, 2.09E−09). Although 14 studies in the DEG identified FtsL as essential, it has been noted that homologues of FtsL often share domain structures that are similar but are not well conserved at the amino acid level (36).

**TABLE 2:** Putative essential F. tularensis Schu S4 genes with no DEG homologues

Locus tag	Gene name	Start	End	Gene length	Function
FTT_0057	FTT_0057	59627	60058	432	Hypothetical membrane protein
FTT_0070c	*ampG*	70944	72209	1,266	Major facilitator superfamily transport protein
FTT_0181c	FTT_0181c	197194	197730	537	Conserved membrane protein
FTT_0265	FTT_0265	278751	280550	1,800	ABC transporter, membrane protein
FTT_0557	FTT_0557	575197	575721	525	AhpC/TSA family protein
FTT_0696	FTT_0696	715529	715879	351	Hypothetical protein
FTT_0743	FTT_0743	766572	767387	816	Conserved hypothetical protein
FTT_0859c	FTT_0859c	870754	871077	324	Hypothetical protein
FTT_0900	FTT_0900	909502	909873	372	Conserved hypothetical membrane protein
FTT_0913	FTT_0913	920784	921251	468	Hypothetical protein
FTT_0924	FTT_0924	935772	936170	399	Hypothetical membrane protein
FTT_1051c	FTT_1051c	1061830	1061994	165	Hypothetical protein
FTT_1113c	FTT_1113c	1123209	1123739	531	Hypothetical protein
FTT_1152	FTT_1152	1166334	1166717	384	Hypothetical protein
FTT_1489	FTT_1489	1542386	1543111	726	Hypothetical protein
FTT_1637c	FTT_1637c	1702959	1703162	204	Hypothetical protein

### Identification of genes required for optimal fitness during infection.

**FIG 3:**
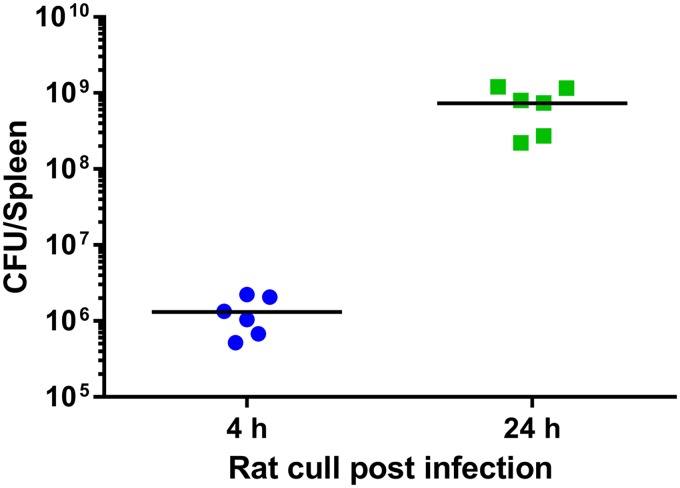
Colonization of Fischer 344 rat spleens with F. tularensis Schu S4. Two groups of rats were challenged by the i.v. route with an F. tularensis Schu S4 Himar1 transposon library. At 4 h or 24 h postchallenge, rats were humanely culled and spleen bacterial burden was determined.

Extending the essential gene analysis, we set out to determine the genes that are important within a host environment. To identify genes important for fitness of F. tularensis in the Fischer 344 rat, a TraDIS approach was again implemented. To avoid potential stochastic loss of mutants from the output pool, the intravenous (i.v.) route was selected for challenge. Fischer 344 rats were challenged i.v. at a dose of 7.8 × 10^6^ CFU of the F. tularensis Schu S4 himar1 transposon library. At this dose, by 4 h postchallenge the bacterial burden in the spleen had reached a mean of 1.31 × 10^6^ CFU/spleen (Fig. 3); this time point was used as an indication of the number of bacteria present in the spleen prior to replication. At 24 h postchallenge there was a mean of 7.32 × 10^8^ CFU/spleen (Fig. 3), representing a 559-fold increase in bacterial numbers.

Genomic DNA was isolated from the input pool and from bacteria isolated from rat spleens at 24 h postchallenge. Transposon-genome junctions were selectively amplified by PCR and sequenced, and fitness analysis was performed using DESeq. To ensure accuracy and reduce the number of genes falsely identified, only genes with an arbitrary minimum value of 200 reads in the input were included in the analysis. A 2-fold reduction cutoff in read count between input and output was applied to generate a list of 163 genes required for fitness within the spleen. Those mutants not present in the output were arbitrarily set a fitness score of −15 (Table S4). Fifty percent of genes conferring reduced fitness in the rat spleen had previously been identified using murine models of *Francisella* infection (Table S4). This includes genes identified using F. tularensis subsp. *holarctica* and F. novicida strains. Of the 163 genes required for fitness in the rat spleen, 101 genes had not previously been identified as important for virulence of F. tularensis subsp. *tularensis* and a further 19 genes had been identified only through changes in gene expression. Additionally, 34 genes had not previously been associated with virulence in any *Francisella* species or subspecies.

### Validation of genes required for fitness in Fischer 344 rats.

**FIG 4:**
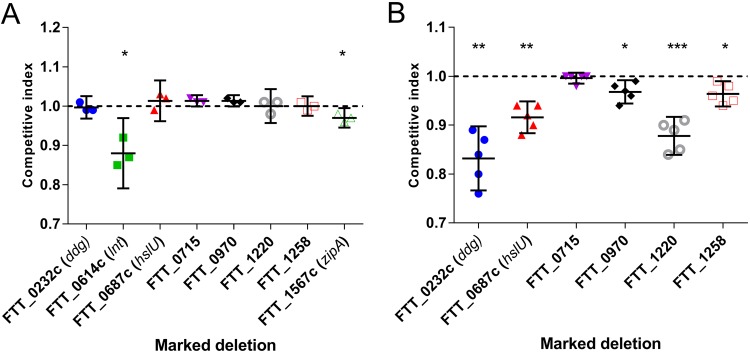
Competitive fitness of F. tularensis Schu S4 mutant strains. The relative fitness of marked mutants was evaluated in competition with WT F. tularensis Schu S4. Values are presented as means ± 95% confidence intervals. Competition under two growth conditions was tested; panel A shows the fitness of individual mutants *in vitro* in MMH broth (*n* = 3), and panel B shows the fitness of mutants in Fischer 344 rats (*n* = 5) that were challenged with the WT and mutant at a 1:1 ratio by the i.v. route. Each datum was analyzed with a one-sample *t* test comparing the competitive index to a value of 1.0. A D’Agostino and Pearson omnibus normality test confirmed Gaussian distribution. *, *P* ≤ 0.05; **, *P ≤ *0.01; ***, *P ≤ *0.001.

To validate data from this TraDIS study, genes were selected for deletion mutagenesis with an emphasis on those not previously reported as involved in virulence (FTT_0232c *ddg* [acyltransferase], FTT_0614c *lnt* [apolipoprotein *N*-acyltransferase], FTT_0970 [uncharacterized protein], FTT_1220 [5-formyltetrahydrofolate cyclo-ligase], FTT_1567c *zipA* [cell division protein], FTT_0687c *hslU* [ATP-dependent protease ATPase subunit], and FTT_1258 [outer membrane efflux protein]) (Table S4). Additionally, a mutant that did not exhibit a fitness change (FTT_0715; *P* = 0.934) was selected as a control. Initially, MMH broth was inoculated with a 1:1 ratio of wild-type (WT) F. tularensis Schu S4 and each mutant. Schu S4 Δ*lnt*::Kan^r^ and ΔFTT_1567::Kan^r^ strains had significantly reduced fitness relative to that of the WT (*P* = 0.020 and *P* = 0.035, respectively) (Fig. 4A).

The targeted marked deletion strains of F. tularensis Schu S4 were tested to assess validity of the Fisher 344 rat fitness data. Mutant strains were individually mixed in a 1:1 ratio with the WT and used to challenge Fischer 344 rats by the i.v. route. Strains exhibiting *in vitro* growth defects were not studied further. Animals were humanely culled at the humane endpoint, spleens were removed, and the WT and mutant bacteria were enumerated by culture (Fig. 4B). All deletion strains tested were significantly less fit than the WT with the exception of the ΔFTT_0715::Kan^r^ strain, for which no change in fitness was observed. The reduced fitness of selected mutants, in competition with the WT, successfully validated the Fischer 344 rat transposon negative selection screen.

## DISCUSSION

The murine model of tularemia has provided significant contributions to our understanding of the molecular pathogenesis of F. tularensis. However, the model is not without limitation. Mutations have attenuated F. tularensis sufficiently for the LVS to be used for human vaccination (37) but are not sufficient to attenuate the organism in mice and prevent lethality, indicating that different mechanisms of virulence maybe be involved for different hosts. The Fischer 344 rat has been used as a model for tularemia vaccine candidate evaluation due to its reduced sensitivity to pulmonary challenge with F. tularensis compared to murine models (38, 39). The lower susceptibility of Fischer 344 rats is described as being closer to that of humans, although similar susceptibilities of mice and Fischer 344 rats to F. tularensis via the intraperitoneal (i.p.) route has been noted (38). Fischer 344 rats, like humans, are highly resistant to the LVS (40), which also provides protection against subsequent challenge with F. tularensis Schu S4. Given that F. tularensis has a broad host range, to fully understand the genetic basis for virulence of this pathogen, it may be necessary to study alternative animal models of infection.

The high insertion frequency of the transposon mutant library described (>300,000 unique mutants) provided the opportunity, prior to study in Fischer 344 rats, to identify genes that are essential *in vitro*. The analysis identified 453 genes as essential under the condition tested. Comparison to data from an F. novicida library of 8,928 mutants (32) revealed that 339 genes were essential in both species. Although differences in essentiality prediction between the species potentially reflect different metabolisms, they may also be a consequence of different experimental conditions utilized in the two studies. To explore genes not previously identified as essential in *Francisella*, we performed BLASTx analysis using the 114 essential genes predicted in F. tularensis Schu S4 but not in F. novicida. Many of these non-DEG essential genes showed homology to genes from bacteria occupying a marine or environmental niche. *Francisella* species have often been associated with aquatic environments (41–43), and the absence of these novel essential genes identified in DEG is therefore likely due to the paucity of essentiality data for organisms occupying this niche.

Our data have identified a number of essential genes for which no homologue was identified in the DEG, of which two conferred a level of functional homology. FTT_0070c encodes AmpG, a putative muropeptide transporter involved in recycling peptidoglycan, and shares 38% identity with Escherichia coli AmpG (95% coverage; E value, 1E−93); disruption of *ampG* in E. coli caused the bacterium to lose up to 40% of its peptidoglycan per generation (36). Peptidoglycan recycling was also identified as an important process *in vivo*, since *ampD* (FTT_0162), which encodes a cytoplasmic amidase (44), was required for fitness within the Fischer 344 rat spleen. A link with virulence has previously been reported for F. tularensis subsp. *holarctica*, for which Brunton et al. (45) observed a reduction in intracellular colonization of *ampD* mutants during negative selection in J774 macrophage-like and TC-1 epithelial cells. Given that the F. tularensis genome has been described as in an advanced state of decay (46), it is possible that a reduced metabolic repertoire could consequently impose a greater requirement for peptidoglycan recycling under a number of different growth conditions. Additionally, FTT_0557 shows homology to peroxiredoxins, a thiol-specific group of antioxidants. Given the requirement of F. tularensis for cysteine (47, 48), a potential role for peroxiredoxin-mediated protection from oxidative stress during aerobic growth is of interest.

In some cases, isogenic mutants have been created in *Francisella* for a number of genes we have identified as essential. Our data predict *mglA* (macrophage growth locus A) and *ripA* (required for intracellular proliferation factor A) to be essential genes for Schu S4. Yet targeted *mglA* and *ripA* deletion strains have been reported for F. tularensis subsp. *holarctica*, F. novicida, and F. tularensis subsp. *tularensis* (with the exception of *mglA* in the last) (17, 49–52), suggesting that this gene is dispensable for *in vitro* growth. However, growth defects have been reported under certain conditions for both of these deletion strains (51–53). To our knowledge, *mglA* deletion has not been reported for F. tularensis subsp. *tularensis*. It is therefore plausible that either an absolute requirement for the *mglA* exists for F. tularensis subsp. *tularensis* or a less fit mutant (for both *mglA* or *ripA*) could be lost through competition during outgrowth of our transposon library. This highlights the need for caution when interpreting competition-based transposon studies, as the potential exists for selection against mutants with a growth disadvantage that are not absolutely required for growth under the conditions of the study.

This Fischer 344 rat screen identified many genes previously associated with virulence of *Francisella* in cell lines, animals, and insect models (fully detailed in Table S4) and contributes to the validation of the sequencing approach used to generate the data. It was not possible to assess the contribution of the *Francisella* pathogenicity island due to sequencing reads mapping to two genomic locations (duplicated 34-kb region).

To further validate the data generated from the Fischer 344 rat *in vivo* screen, 8 targeted deletion mutants were produced in F. tularensis Schu S4. Two mutants, F. tularensis Schu S4 Δ*lnt*::Kan^r^ and ΔFTT_1567c::Kan^r^ mutants, had a reduced competitive index (CI) *in vitro*, suggesting a host-independent growth defect when in competition with WT Schu S4. FTT_1567c contains a ZipA C-terminal domain, as predicted by Interpro annotation (54, 55). ZipA interacts with FtsZ during cell division, promoting septum formation (56), and is known to be essential across a number of bacterial genera (24, 57–62), but due to the narrow conservation of ZipA between bacterial species, further interrogation is necessary to determine the function of FTT_1567c. The second mutant exhibiting a defect in growth is the Δ*lnt*::Kan^r^ mutant, in which *lnt* is predicted to encode an apolipoprotein *N*-acyltransferase, an enzyme involved in lipoprotein maturation (63). Lipoproteins play a diverse role in bacterial pathogens, ranging from antibiotic resistance to sporulation and many aspects of bacterial virulence (64). LoVullo et al. determined that *lnt*, a gene that is often found to be essential in Gram-negative bacteria, is not essential for replication of F. tularensis LVS and detected a minor defect in bacterial physiology (63). Our studies identified more significant changes to bacterial physiology following deletion of *lnt* in F. tularensis Schu S4, primarily a change in cell size during passage *in vitro* (data not shown) with striking similarity to the changes observed previously through deletion of a predicted lipoprotein, FTT_0831c (65).

This study identified 34 genes that had not previously been associated with virulence in *Francisella*. One example is *rng*, which encodes RNase G. RNases are involved in RNA processing and decay (66) and have been shown to influence virulence gene expression (67, 68). RNase C involvement in F. tularensis Schu S4 virulence was previously shown in a negative selection screen using human-derived macrophages (69), and this finding was consistent with our *in vivo* Fischer 344 rat data. Inactivation of *rng* has been linked to upregulation of the heat shock response genes (70), but its role in *Francisella* virulence is unclear. Another potential novel virulence gene identified is the FTT_0750 gene, with homology to the elongation factor P–(*R*)-beta-lysine ligase (EpmB) gene in Salmonella enterica (98% coverage; E value, 1E−101; identity, 45%), which has been shown to modulate virulence and drug resistance (71). As with many of the genes identified in these studies, limited research has been undertaken. This investigation therefore represents a starting point to stimulate investigations that expand our knowledge on the pathogenesis of F. tularensis.

Currently, there is no licensed vaccine that can protect against F. tularensis. The LVS is able to provide protection in humans against aerosol exposure to virulent F. tularensis (5, 72), but the basis for protection remains unknown. Data generated from this Fischer 344 *in vivo* negative screen could provide the basis for the rational design of live vaccine strain candidates. The challenge is to identify candidate mutant strains capable of sufficient host colonization to induce a robust adaptive immune response but then unable to disseminate and cause disease. Strains with a greater potential to overattenuate could be identified by those mutants that have been lost entirely from the library or present at low levels in the output which may not be sufficiently exposed to the immune system to provide lasting immunity. A number of live attenuated vaccine candidates have been proposed, such as strains with the *clpB* (73) and FTT_1103 (74) genes. These strains have been shown to induce protective immunity and in our study presented a moderate log_2_ fold change (−1.83 and −1.88, respectively) in the output (see Table S4 in the supplemental material). Therefore, the potential exists to utilize transposon-based fitness data to increase the probability of identifying mutant strains (live vaccine candidates) that are capable of inducing an immune response but are unable to cause disease.

In conclusion, we have generated a saturated transposon library containing >300,000 unique mutants. In combination with high-throughput next-generation sequencing, we have determined gene essentiality of F. tularensis Schu S4 and identified genes that are required for fitness within the Fischer 344 rat spleen. We were able to confirm previously identified virulence-related genes and identify new ones. Despite having a diverse host range, *Francisella* is rarely studied outside the mouse model. Our studies of gene essentiality and *in vivo* fitness provide the foundation to further understand the nutritional requirements and pathogenicity of this highly virulent organism.

## MATERIALS AND METHODS

### Bacterial strains, plasmids, and growth conditions.

F. tularensis subsp. *tularensis* Schu S4 (75) was used to construct a saturated transposon library. Strains of F. tularensis were grown routinely on modified Mueller-Hinton (MMH) agar or broth (76) or Thayer-Martin agar (77). Where required, medium was supplemented with kanamycin (10 μg/ml), chloramphenicol (5 μg/ml), or polymyxin B (100 μg/ml). All work with F. tularensis was undertaken with the appropriate laboratory containment conditions. Use of chloramphenicol and kanamycin resistance cassettes was approved locally by a genetic manipulation safety committee and approved nationally by the Health and Safety Executive (UK Health and Safety Authority). Lists of strains and plasmids (Table S1) and primers (Tables S2 and S3) can be found in the supplemental material.

### Generation of mutant libraries.

Mutagenesis of the Francisella tularensis Schu S4 genome was achieved utilizing a modified Himar1 transposon (HimarFT) contained within a minimal suicide vehicle (pHimarH3), for which random, single, and stable insertions within F. tularensis have previously been demonstrated (28, 78). F. tularensis, grown at 37°C in MMH broth, was rendered electrocompetent by sequential washing and concentration in 0.5 M sucrose prior to electroporation with 1 μg of pHimarH3 at a field strength of 15 kV/cm in 0.2-cm-gap cuvettes (79). After recovery in MMH broth for 3 h, transformants were plated onto MMH agar supplemented with kanamycin. Cell numbers were determined by serial dilution prior to the collection of transformants directly from the plates. Transformants were pooled into batches of 40,000 before being combined to create the final transposon library of 520,000 mutants.

### *In vitro* essential analysis.

A frozen vial of library stock was diluted into 50 ml of MMH broth to an optical density at 590 nm (OD_590_) of 0.5 and recovered by overnight incubation at 37°C and 200 rpm, achieving a final OD_590_ of 3.40. Genomic DNA was isolated with the Puregene yeast/bacterium DNA purification kit (Gentra Systems) according to the manufacturer’s instructions.

### *In vivo* fitness studies in Fischer 344 rats.

Female Fischer 344 rats 6 to 7 weeks old (∼100 g) were purchased from (Envigo, Indianapolis, IN). The rats had access to food and water with 12-h light/dark cycles and were acclimatized for 10 days prior to the procedure. Animals were handled under biosafety level 3 containment conditions within a half-suit isolator, compliant with British standard BS5726. All procedures within this study were carried out according to the requirements of the United Kingdom Animal (Scientific Procedures) Act 1986. A frozen vial of library stock was diluted into 50 ml of MMH broth to an OD_590_ of 0.5 and was recovered by overnight incubation at 37°C and 200 rpm, achieving a final OD_590_ of 3.40. F. tularensis Schu S4 was diluted in phosphate-buffered saline (PBS) to 1 × 10^8^ CFU per ml, and 100 μl was used to challenge each of six rats via the i.v. route. Humane endpoints were strictly observed, and animals assessed as incapable of survival were humanely killed by the administration of 1 ml of sodium pentobarbital by the i.p. route. Spleens were removed and homogenized with 2 ml of PBS using 40 μM cell strainers, and for each rat, 100 μl of spleen homogenate was spread onto each of eight MMH agar plates. Bacteria were removed from the agar plates following incubation at 37°C for 48 h and suspended into 10 ml of PBS. Genomic DNA was recovered using the Puregene Gentra kit according to the manufacturer’s instructions, with the addition of a proteinase K (100 μg/ml) digestion step for 1.5 h at 55°C following RNase A treatment.

### Illumina sequencing.

The DNA was separated into approximately 500-bp fragments using a Covaris machine with settings as follows: time, 110 s; peak incident power (PIP), 105 W; 200 cycles/burst; and 5% duty factor. The fragments were purified using a Qiagen PCR purification kit according to the manufacturer’s instructions. Libraries were prepared using a NEBNext DNA library preparation kit for Illumina, according to the manufacturer’s instructions: ends were repaired and A-tailed, and then annealed adapters Ind_Ad-T and Ind_Ad-B were ligated to the free ends. The fragments were then PCR amplified in 10 parallel reactions using primers PE_PCR_V3.3 and Ft_1 FC (Table S1B); the reaction mixtures were made up of 10 μl of JumpStart 10× buffer, 6 μl of MgCl_2_, 2 μl of 10 mM deoxynucleoside triphosphates (dNTPs), 0.6 μl of 100 μM PE_PCR_V3.3 primer, 0.6 μl of Ft_1 FC primer, 1 μl of JumpStart Taq DNA polymerase, and 28.8 μl of nuclease-free water. The reactions were amplified at 94°C for 10 min (94°C for 30 s, 65°C for 20 s, and 72°C for 30 s) for 22 cycles and 72°C for 10 min and then held at 12°C. The PCR products were pooled and quantified using quantitative PCR (qPCR). They were then size selected on a 2% agarose–1× Tris-borate-EDTA (TBE) gel; agarose blocks were excised corresponding to 350 to 500 bp. These were treated with a Qiagen MinElute gel extraction kit, and then the DNA fragments were requantified both by qPCR and on an Agilent bioanalyzer and sequenced as 100-bp single end reads on an Illumina Hi-Seq 2500 standard model.

### Bioinformatic analysis for essential genes.

Adapter sequences and low-quality ends were removed from the raw sequencing reads using Trimmomatic (80), and reads that had been reduced to below 24 bp in length were dropped. Processed reads were mapped, insertion sites/read counts were tabulated, and a prediction of essentiality was determined using the Bio::Tradis pipeline as described previously (30, 31) (https://github.com/sanger-pathogens/Bio-Tradis). Briefly, reads containing the pHimarH3 transposon tag were filtered and mapped to the F. tularensis Schu S4 reference genome (EMBL accession number AJ749949) using SMALT-0.7.2, allowing 0 mismatches. Postprocessing of the mapped reads involved determination of the gene insertion index (insertion count/gene length) and excluded the first 5% and last 15% of each gene to avoid insertions that may not affect gene function. Gene essentiality was predicted through fitting gamma distributions to the essential and nonessential peaks of the observed bimodal distributions. Log_2_ likelihood ratios were calculated between the two modes providing the insertion-index threshold and the prediction of gene essentiality. Genes with log_2_ likelihood ratios of less than −2 were described as essential, and those with log_2_ likelihood ratios of greater than 2 were considered nonessential genes (31).

### *In vivo* fitness analysis by DESeq.

Sequence reads were aligned to the reference genome using the sequence alignment tool BWA (81). The mismatch error limit was set 2. All other parameters used default values of the tool. After alignment, an in-house script was used to calculate the frequency data for each gene in each replicate as previously described (82). The “executable C script for annotation” (accessed at http://ecsb.ex.ac.uk/DEM/index.html) established the frequency of transposon hits across the genome. The count data were then converted to reads per kilobase of transcript per million mapped reads (RPKM). The fitness package DESeq (83) analyzed the matrix to predict the differential fitness of individual mutants. DESeq is a tool for analyzing high-throughput sequence reads data for detecting differentially expressed genes with an assumption that the count data follow a binomial distribution. Default values were used within the DESeq package, and a list detailing the relative abundance of the individual mutants was generated. The tool generates a list of differentially expressed genes based on the *P* values (84).

### Generation of marked and unmarked mutants.

Marked mutants were generated using a modified version of the SacB/oriT suicide plasmid pEDL50 (85) to facilitate the use of the kanamycin gene and its GroES promoter derived from pHimarH3 as the marker for mutagenesis. The kanamycin resistance gene on the pEDL50 backbone was replaced with a chloramphenicol resistance gene. Initially, the multiple-cloning site was removed by restriction digestion with AleI and EcorV and recircularized by ligation. The resulting plasmid was named pMIRE14 and was generated to remove a second PstI site. The chloramphenicol gene from pKK201 (86) was cloned, including a 107-bp upstream region, and was flanked by 5′ SpeI and 3′ PstI sites. Restriction digestion of the subcloned chloramphenicol gene (Zero Blunt TOPO; Thermo Fisher) and pMIRE14 with SpeI and PstI followed by ligation allowed the direct replacement of the kanamycin resistance gene with a chloramphenicol resistance cassette, generating pMIRE15.

To generate marked deletion constructs, flanking regions (∼750 bp) of the target gene were amplified by PCR and ligated into pMIRE15 on either side of a kanamycin resistance gene that had been amplified from pHimarH3 (78). The deletion constructs were electroporated into E. coli S17 λ pir and mobilized into WT F. tularensis Schu S4 by conjugation as described previously (87). Merodiploids generated through chromosomal integration of the suicide plasmid were selected on Thayer-Martin agar containing either chloramphenicol or kanamycin and polymyxin B and incubated at 37°C. Following PCR verification, isolated colonies were spread by diminishing streak onto Thayer-Martin agar supplemented with 5% (wt/vol) sucrose and 10 μg/ml of kanamycin and incubated at 37°C until colonies were evident. The marked deletion was confirmed by PCR of all mutants and six by whole-genome sequencing.

### *In vitro* competitive index.

F. tularensis Schu S4 strains were prepared from sucrose stocks onto MMH agar and incubated for 24 h at 37°C. Cells were adjusted by OD_590_ in MMH broth to 5 × 10^8^ CFU/ml and incubated for 24 h at 37°C and 220 rpm. Each strain was adjusted by OD_590_ to 5 × 10^8^ CFU/ml in MMH broth and used to prepare a 1:1 ratio of each mutant with WT F. tularensis Schu S4. Growth in competition was allowed to proceed for 24 h at 37°C. CFU were determined by serial dilution onto both MMH agar alone and MMH agar with 10 μg/ml of kanamycin. CFU values were log_10_ transformed and used to calculate the competitive index of WT to marked deletion using the following formula: (mutant output count/WT output count)/(mutant input count/WT input count).

## Supplementary Material

Supplemental file 1

Supplemental file 2

Supplemental file 3
