# Supplementary material for: Global Analysis of Genes Essential for Francisella tularensis Schu S4 Growth In Vitro and for Fitness during Competitive Infection of Fischer 344 Rats
Source: J Bacteriol. 2019 Mar 13;201(7):e00630-18. doi: 10.1128/JB.00630-18 (PMC6416918; doi:10.1128/JB.00630-18)
Supplement: Supplemental file 1 [file JB.00630-18-s0001.pdf]

Supplemental information for

Global analysis of genes essential for *Francisella tularensis* Schu S4 growth *in vitro* and for fitness during competitive infection of Fischer 344 rats.

Authors & Affiliations

Philip M. Ireland[a]#, Helen L. Bullifent[a], Nicola J. Senior[b], Stephanie J. Southern[a], Zheng Rong Yang[b], Rachel E. Ireland[a], Michelle Nelson[a], Helen S. Atkins[a,b,c,] Richard W. Titball[b], Andrew E. Scott[a].

[a]Defence Science and Technology Laboratory, Chemical, Biological and Radiological Division, Porton Down, Salisbury, SP4 0JQ, UK

[b]College of Life and Environmental Sciences, University of Exeter, Exeter , UK;

[c]Department of Pathogen Molecular Biology, London School of Hygiene and Tropical Medicine. Keppel Street, WC1E 7HT, London, UK

# Address correspondence to Philip. M. Ireland (pmireland@.dstl.gov.uk)

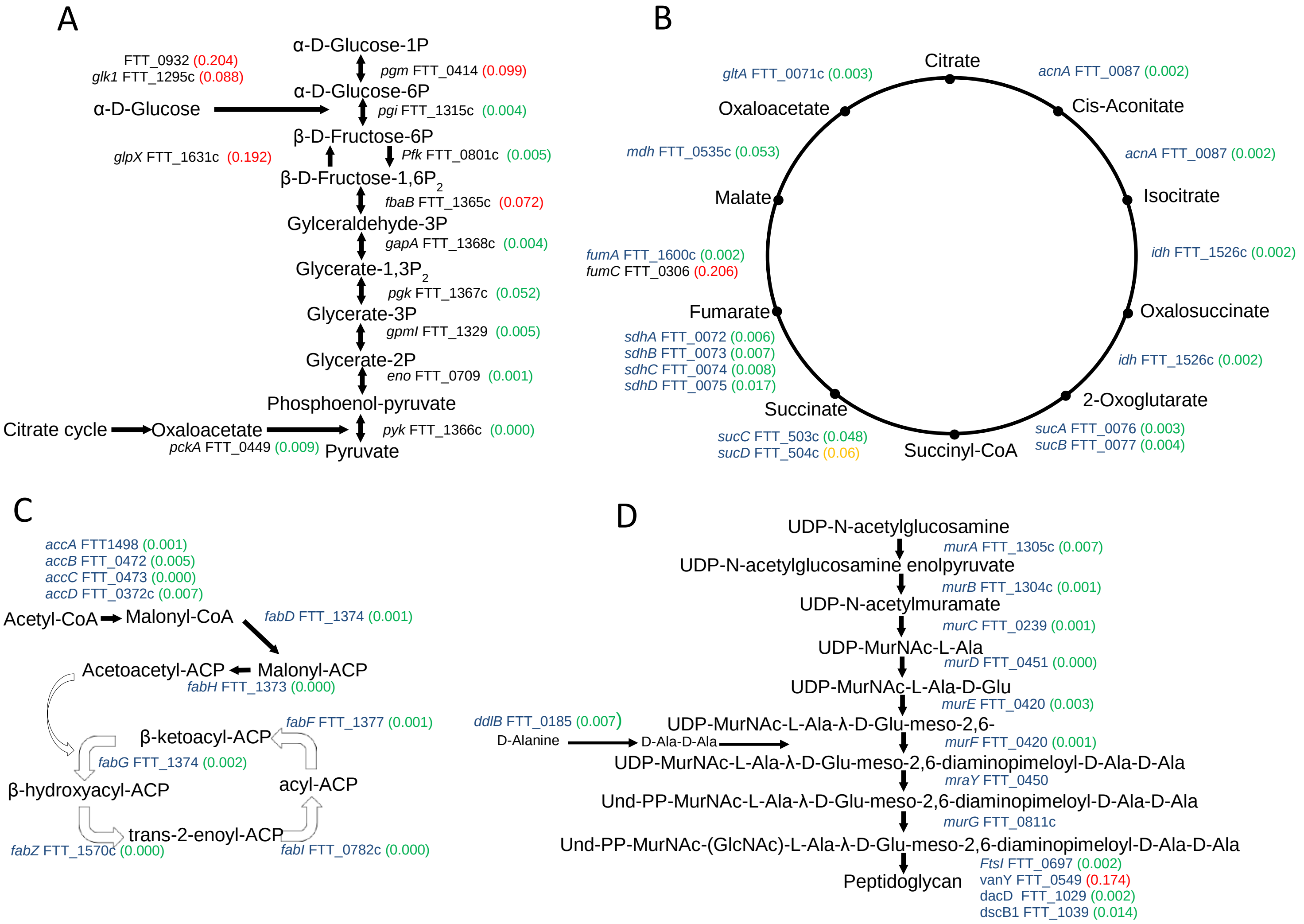

Figure S1. Predicted essential genes in *F. tularensis* Schu S4 map to key metabolic pathways. Predicted genes align to KEGG biochemical pathways (A) glycolysis/gluconeogenesis, (B) TCA cycle, (C) fatty acid biosynthesis and (D) peptidoglycan synthesis. An insertion index below the essentials threshold is indicated in green. The insertion index of those predicted as non-essential are red and ambiguous values are coloured orange.

Table S1: Bacterial strains and plasmids used in this study

	Description	Reference
**Strain**		
F. tularensis Schu S4	Human ulcer, 1941, OH, USA	(1)
F. tularensis Schu S4 ΔFTT1258::Kan^R	ΔFTT1258 gene replacement	This study
F. tularensis Schu S4 Δ*hlsU*::Kan^R	Δ*hlsU* gene replacement	This study
F. tularensis Schu S4 Δ*ddg*::Kan^R	Δ*ddg* gene replacement	This study
F. tularensis Schu S4 Δ*lnt*::Kan^R	Δ*lnt* gene replacement	This study
F. tularensis Schu S4 ΔFTT_0715::Kan^R	ΔFTT_0715 gene replacement	This study
F. tularensis Schu S4 ΔFTT_0715	ΔFTT_0715 gene deletion	This study
F. tularensis Schu S4 ΔFTT_0970::Kan^R	ΔFTT_0970 gene replacement	This study
F. tularensis Schu S4 ΔFTT_1567c::Kan^R	ΔFTT_1567c gene replacement	This study
F. tularensis Schu S4 ΔFTT_1220::Kan^R	ΔFTT_1220 gene replacement	This study
E. coli S17-1	Plasmid mobilization strain	(2)
**Plasmids**		
pEDL50	*F. tularensis* suicide vector for allelic exchange, *sacB*, Kan^R.	(3)
pMIRE14	pEDL50 without MCS	This study
pMIRE15	pEDL14 with Kan^R replaced with Cam^R	This study
pMIRE22	pMIRE15, FTT_1258 replacement construct	This study
pMIRE23	pMIRE15, *hlsU* replacement construct	This study
pMIRE24	pMIRE15, *ddg* replacement construct	This study
pMIRE25	pMIRE15, lnt replacement construct	This study
pMIRE26	pMIRE15, FTT_0715 replacement construct	This study
pMIRE27	pMIRE15, FTT_0715 deletion construct	This study
pMIRE28	pMIRE15, FTT_0970 replacement construct	This study
pMIRE29	pMIRE15, FTT_1567c replacement construct	This study
pMIRE30	pMIRE15, FTT_1220 replacement construct	This study

Table S2: Primers for mutant construction and validation

Primers	Sequence (5'-3')	Template
**Choloramphenicol resistance**		
CAM_F	ACTAGTATCGGCACGTAAGAGGTTCC	pkk202 (4)
CAM_R	CTGCAGTAGCACCAGGCGTTTAAGGG	
**Kanamycin resistance**		
Kan_F	GGATCCTGTATGGATTAGTCGAGCTA	pHimar1H3 (5)
Kan_R	GGATCCTCAGAAGAACTCGTCAAGAA	
Kan_FTT1258F*	GTAGATACATTATAGGTCACAACAGGATCCTGTATGGATTAGTCGAGC	
Kan_FTT1258R*	GCTTGGTTAACTTGCATTTGTTGATAGGATCCTCAGAAGAACTCGTCAAG	
Kan_hslUF*	GTTATACTATGACACAAATAATGGGATCCTGTATGGATTAGTCGAGC	
Kan_hslUR*	GCTTGGTTAACTTGCATTTGTTGATAGGATCCTCAGAAGAACTCGTCAAG	
Kan_ddgF	CATAAAAAATAAATATACTTGTATGGATTAGTCGAGC	
Kan_ddgR	TAACAATCATAAATATCAGAAGAACTCGTCAAGAA	
Kan_614F	CGAAAAATTTAAGAAATATTGTATGGATTAGTCGAGC	
Kan_614R	GAAAATTATTATAAATCAGAAGAACTCGTCAAGAAGG	
**Allelic exchange**		
FTT1258_LFF*	AACGCGCCACCGATCAAGCTTATCGATACCGTCGACATCCACAACAACCAATTTGTTCAC	Schu S4 genomic DNA
FTT1258_LFR*	TTAGCTCGACTAATCCATACAGGATCCTGTTGTGACCTATAATGTATCTAC	
FTT1258_RFF*	TTCTTGACGAGTTCTTCTGAGGATCCTATCAACAAATGCAAGTTAACCAAGC	
FTT1258_RFR*	GCGTAACAGATGAGGGCAAGCGGGCCCCCCCTCGAGAGTTTGTCCTTGCCATAGTCC	
hslU_LFF*	GCCACCGATCAAGCTTATCGATACCGTCGACGCTGCTAGCGAAATAGTTC	
hlsU_LFR*	ATGAGCTTTTTAGCTCGACTAATCCATACAGGATCCCATTATTTGTGTCATAGTATAAC	
hlsU_RFF*	TTCTTGACGAGTTCTTCTGAGGATCCTTCGATGCTCCAGAACTAGTAG	
hlsU_RFR*	GCGTAACAGATGAGGGCAAGCGGGCCCCCCCTCGAGTTCTATCCGCGACTGTCTTC	
ddg_LFF	CATATGGGTGATGTATTCGAAGCTGGAG	
ddg_LFR	GTATATTTATTTTTTATGAGTGGCTTG	
ddg_RFF	TATTTATGATTGTTAAAGTATATG	
ddg_RFR	CATATGAGGCGTCACCTGTAAACTCG	
lnt_LFF	CATATGTATAAAAAGACTTACCTCAAGC	
lnt_LFR	TATTTCTTAAATTTTTCGACTAAAATT	
lnt_RFF	TTTATAATAATTTTCTGCATAATTAATCA	
lnt_RFR	CATATGTATGATTGTCAATGCTATTCG	
FTT0715_LFF	CTCGAGTTCTTCAATATGCTCTTTAG	
FTT0715_LFR	GGATCCCATAAGTATTTCTCCTTATT	
FTT0715_RFF	GGATCCAAACAATAATAAAGTCTTAT	
FTT0715_RFR	CTCGAGGGAATTATATATTTTTTTGTC	
FTT0970_LFF	CTCGAGGAGAGTATCTGTGATGAATATGC	
FTT0970_LFR	GGATCCAATTACCTTTAATCTATACTAATTTAGTATC	
FTT0970_RFF	GGATCCGTATGGTAAGTGGTTATTGG	
FTT0970_RFR	CTCGAGTCTAGTGCCACTAACTCAAC	
FTT1567_LFF	GACTGTCGACTGGGTTTGGCGTGAATAC	
FTT1567_LFR	GACTGGATCCTGTCATTTATCTAATACCTATAC	
FTT1567_RFF	GACTGGATCCCCTAGCAGCTATTTTATAAC	
FTT1567_RFR	GACTGTCGACTGCTTGACCGAAGAAACC	
FTT1220_LFF	GACTGTCGACTCGCTAATTGCTTCCTTG	
FTT1220_LFR	GACTGGATCCAAGCTCTCTACGTATTTG	
FTT1220_RFF	GACTGGATCCCGAGGATATTATAACTATGG	
FTT1220_RFR	GACTGTCGACCAGCAACAACTACTATAGC	

* Primers were used generate deletion constructs by ligationless cloning (NEBuilder HiFi DNA kit, NEB) all other constructs generated by restriction digest and T4 mediated ligation.

Table S3: Primers and adapter sequences for Ilumina sequencing

Primer function	Primer name	Primer sequence
Library preparation	FT_1FC	**AATGATACGGCGACCACCGAGATCTACAC**TAATATGCATTTAATACTAGCGACGC[a]
	PE_PCR_V3.3	**CAAGCAGAAGACGGCATACGA**GATCGGTACACTCTTTCCCTACACGACGCTCTTCCGATCT[a]
Adapters	Ind_Ad-T	ACACTCTTTCCCTACACGACGCTCTTCCGATC*T[b]
	Ind_Ad-B	pGATCGGAAGAGCGGTTCAGCAGGAATGCCGAGACCGATCTC[c]
Sequncing primer	Ft_Himar1_seq	CTAGATGTGTCAGACCGGGGACTTATCAGC

[a]HPLC purified from MWG Eurofins. Region of primer binding to HiSeq 2500 flow cell highlighted in bold
[b]HPLC purified from MWG Eurofins. * phosphorothioate
[c]HPLC purified from MWG Eurofins. Phosphorylated.

Table S4 Genes that through insertional inactivation impose significant reductions in bacterial fitness of *Francisella tualrensis* subsp. *tularensis* Schu S4 in the Fischer 344 rat spleen.

Locus tag	Gene name	Input count	Output count	log2Fold Change	padj	Strain[a]	Host[b]	Reference[c]
FTT_1462c	*wbtC*	1207	0	-12.41	1.34E-33	L, S	Mø, Ep, Mou	(5-8)
FTT_0056c		2526	2	-10.36	2.82E-99	S, N	Mø, Mou, Hep	(9-12)
FTT_1631c	*glpX*	1429	3	-8.97	8.69E-148	L,N	Mø, Ep, Mou	(5, 7, 9, 13-15)
FTT_0936c	*bioF*	862	2	-8.85	5.49E-28	L,N	Mø, Ep, Mou	(7, 9, 13, 16)
FTT_0963c	*aroG*	735	2	-8.45	3.29E-96	L	Mou	(13)
FTT_0941c		466	1	-8.43	8.61E-72	L, N	Mø, Mou	(9, 13, 17, 18)
FTT_0944		2943	11	-8.10	4.60E-108	N	Mou	(9)
FTT_1721c	*purF*	261	1	-8.01	1.62E-41	S, N	Mou, Hep	(9, 10, 19, 20)
FTT_1450c	*wbtM*	713	3	-7.92	3.49E-89	L	Mø	(5)
FTT_1720c	*purL*	641	3	-7.87	4.23E-83	L, S, N	Mø, Ep, Mou, Hep	(7, 9, 10, 18, 19)
FTT_1149c		265	1	-7.72	4.06E-42	S	Mou	(19)
FTT_0934c	*bioD*	332	2	-7.63	3.87E-20	L, N	Mø, Mou	(13, 18)
FTT_1689c		831	5	-7.49	1.59E-46	L, N	Mø, Ep, Mou, Dros	(7, 9, 18, 21, 22)
FTT_0953c		2508	17	-7.19	2.51E-122	L, S(T)	Mø, Ep	(7, 23)
FTT_0479c	*perM*	345	2	-7.17	4.93E-49	L, S	Mø, Ep	(6, 7)
FTT_1762c		435	4	-6.95	6.62E-34	S, N	Hep, Dros	(10, 12)
FTT_1181c	*ggt*	4279	37	-6.86	7.31E-41	L, S	Mø, Hep, Mou	(5, 10, 19, 24, 25)
FTT_0894	*purCD*	481	5	-6.62	8.03E-35	L, N	Mø, Ep, DrosC	(7, 16, 26)
FTT_0857c		210	2	-6.58	6.89E-15	S	Mø	(6)
FTT_0094c	*qseC*	482	6	-6.29	1.09E-54	N	Mou, Dros	(9, 12)
FTT_1647c	*pyrD*	1134	19	-5.92	3.23E-48	N	Mø, Mou, Dros	(9, 12, 18, 21)
FTT_1447c	*manB*	202	3	-5.91	1.65E-26	L, S, N	Mø, Ep, Mou, Dros	(7, 9, 12, 21, 27, 28)
FTT_0204	*purA*	384	7	-5.83	5.48E-43	L, S, N	Mø, Ep, Hep	(7, 10, 18)
FTT_1318c	*pepA*	1591	28	-5.82	1.25E-98	L, N	Mø, Ep, Dros	(7, 12)
FTT_1394c	*recB*	1120	20	-5.77	1.52E-82	L, N	Mø, Ep, Mou, Dros	(7, 9, 12, 21)
FTT_0107c	*dsbB*	951	19	-5.64	2.50E-28	L, S, N	Mø, Ep, Mou, Hep, Dros	(5, 7, 9, 10, 18, 21, 29)
FTT_1238c		253	6	-5.39	4.97E-20	L, N	Mø, Ep, Mou	(5, 7, 9, 16)
FTT_1256	*emrB*	3367	87	-5.27	4.20E-22	S(T), N	Mø, DrosC	(16, 23, 26)
FTT_1502		3154	91	-5.11	6.10E-24	L	Mø, Ep, Mou	(7, 22)
FTT_1258		2320	81	-4.84	2.99E-24	N	Mou	(9)
FTT_1257	*emrA1*	816	37	-4.47	5.02E-51	L, S, N	Mø, Mou, Dros,	(6, 9, 12, 30)
FTT_1640c		599	32	-4.21	5.39E-12	S, N	Mou	(9, 19)
FTT_1471c	*deaD*	281	16	-4.17	4.07E-23	L, S, N	Mø, Mou	(6, 13, 14, 23)
FTT_0807		745	47	-3.98	1.78E-39	L, S, N	Mø, Mou	(5, 6, 13, 14)
FTT_0614c		650	41	-3.98	1.74E-37			
FTT_0805	*capB*	576	41	-3.81	1.87E-32	L, S, N	Mø, Mou	(5, 9, 13, 23, 31, 32)
FTT_0945		2110	166	-3.67	1.04E-14	L, S(T), N	Mø, Mou	(5, 9, 16, 23)
FTT_0946	*trpG1*	772	61	-3.66	2.41E-28	N	Mou	(9)
FTT_0573	*alr*	679	54	-3.66	2.82E-26	N	Mø	(16)
FTT_1397c	*recC*	1169	93	-3.66	4.82E-43	L, N	Mø, Ep, Dros	(7, 12)
FTT_1563	*pcs*	464	37	-3.65	1.07E-05	N	Mou	(9, 14)
FTT_0232c	*ddg*	892	71	-3.65	7.20E-37	S(T)	Mø	(23, 33)
FTT_0476c	*poxA*	430	38	-3.50	0.001188	L, N	Mø, Ep, DrosC	(7, 16, 26)
FTT_0288c	*pdxY*	715	64	-3.48	3.68E-12	N	Mou	(9)
FTT_0238	*aroE1*	269	25	-3.43	0.000231	N	Dros	(12)
FTT_0687c	*hslU*	851	79	-3.43	1.16E-12	S	Mø, Mou	(19, 23)
FTT_0759		957	95	-3.33	1.52E-31	L	Mou	(13)
FTT_0895	*purN*	854	85	-3.32	2.35E-32	L,N	Mø, Ep, Mou	(7, 9)
FTT_0806	*capC*	323	35	-3.19	2.39E-17	L, S(T), N	Mø, Mou	(5, 9, 13, 14, 23)
FTT_1555c	*rnc*	535	59	-3.17	1.01E-23	S	Mø	(6)
FTT_1611		285	32	-3.17	1.62E-13	L, S, N	Mø, Mou	(6, 13, 16)
FTT_1804c	*rng*	353	40	-3.15	3.26E-16			
FTT_1724c	*tolC*	1206	140	-3.11	8.36E-32	L, S	Mou	(19, 34)
FTT_0583	*fopA1*	291	36	-3.02	2.77E-14	L, S	Mø, Mou	(6, 13)
FTT_0626	*lon*	367	45	-3.02	2.86E-17	L, S, N	Mø, Mou	(6, 13, 14, 16, 19, 23)
FTT_1268c	*dnaJ1*	266	33	-3.00	4.20E-14	S(T)	Mø	(23)
FTT_1688		238	30	-3.00	2.26E-11	L, S, N	Mø, Mou, DrosC	(5, 6, 9, 16, 26)
FTT_1567c		536	72	-2.90	2.77E-19			

FTT_0562	*potG*	287	39	-2.89	2.11E-10	N	Mø	(16)
FTT_0453c		243	34	-2.86	3.93E-11	N	Mø, Mou	(9, 18, 35)
FTT_0165c		745	106	-2.82	5.26E-09	L	Mø, Ep	(7, 9)
FTT_0750		2256	329	-2.78	9.48E-14			
FTT_0257		280	42	-2.74	4.23E-13	N	Mø, Mou	(14, 16)
FTT_0414	*pgm*	636	98	-2.70	6.46E-17	S (T), N	Mou, Dros	(9, 12, 21, 23)
FTT_0268		753	118	-2.68	1.79E-17	N	Mou	(14)
FTT_0509c		338	54	-2.66	1.29E-11	N	Mø, Mou, Dros, DrosC	(9, 12, 16, 18, 26)
FTT_1220		505	81	-2.64	5.36E-16	L	Mø, Mou	(36)
FTT_1747		612	99	-2.63	8.13E-18	L	Mou	(13)
FTT_1589c		658	106	-2.63	1.50E-19			
FTT_1255c		304	51	-2.59	8.80E-11			
FTT_1237		404	68	-2.57	3.83E-15	L, S, N	Mø, Ep, Mou	(6, 7, 9)
FTT_1564		952	162	-2.55	9.77E-21	L, N	Mø, Mou	(37, 38)
FTT_0132	*glpA*	552	96	-2.52	3.96E-16	L, S	Mø, Ep, Mou	(6, 7, 19)
FTT_1648c	*pyrF*	650	116	-2.49	5.14E-05	L, S, N	Mø, Mou, Dros	(9, 12, 18, 21, 39)
FTT_0970		423	76	-2.48	2.78E-07			
FTT_1087c	*rep*	1482	266	-2.48	3.42E-23	S, N	Mou	(14, 19)
FTT_1046c		477	88	-2.44	0.001075	S(T)	Mø	(23)
FTT_0564	*potI*	323	60	-2.43	5.09E-10	N	Mou	(14)
FTT_0053		3244	615	-2.40	6.04E-22	L, N	Mø, Ep, Mou, Dros	(5, 7, 9, 12)
FTT_0054		1615	307	-2.39	3.05E-21	N	Mø, Mou	(9, 16, 40)
FTT_1302		404	79	-2.36	9.17E-06			
FTT_0134		598	120	-2.31	1.02E-10	N	Mou, Dros	(9, 12, 40)
FTT_0396	*parC*	765	163	-2.23	4.74E-14	N	Mø	(16)
FTT_0028c		1390	298	-2.22	1.70E-20	S(T), N	Mø, Mou, Dros	(9, 12, 16, 18, 23)
FTT_1095c		1063	228	-2.22	9.23E-15			
FTT_0030c	*fur*	256	56	-2.19	3.14E-07	S(T), N	Mø, DrosC	(16, 23, 26)
FTT_0834	*aroQ*	203	47	-2.11	1.90E-06	L, S	Mø, Ep	(6, 7)
FTT_0618c	*yleA*	318	77	-2.05	1.98E-08	L, N	Mø, Ep, Mou	(5, 7, 14)
FTT_0683c	*pilD*	2192	529	-2.05	4.01E-11	L	Mø, Ep	(7)
FTT_0541c	*yqaB*	1011	256	-1.98	1.70E-08			
FTT_0289c		565	147	-1.95	3.37E-05			
FTT_1254	*fadD1*	361	97	-1.90	1.64E-07	S, N	Mø, Mou	(6, 9, 16)
FTT_1027c	*yrbI*	257	69	-1.89	6.44E-06			
FTT_1103		1046	284	-1.88	2.67E-07	L, S	Mø, Hep, Mou	(5-7, 10, 13, 41, 42)
FTT_0827c		509	141	-1.86	4.04E-09			
FTT_0163c	*parE*	1070	298	-1.84	1.35E-12			
FTT_1769c	*clpB*	4133	1160	-1.83	1.97E-14	L, S, N	Mø, Ep, Mou, Dros	(5, 7, 9, 13, 18, 21, 23, 43-46)
FTT_0162	*ampD*	219	62	-1.83	1.67E-05	L, N	Mø, Ep, Mou	(7, 9)
FTT_0166c		663	189	-1.81	3.19E-10	FSC200	Mø, Mou	(47)
FTT_0511		1001	290	-1.79	0.000844			
FTT_0215	*priA*	324	94	-1.79	1.86E-06	S	Mø	(6)
FTT_0881c		326	96	-1.76	1.66E-07	S	Mø	(6, 13)
FTT_1668	*sdaC2*	1813	535	-1.76	1.47E-11	N	Mø, DrosC	(16, 26)
FTT_1435c	*yagD*	972	288	-1.76	8.53E-05			
FTT_0803	*ans*	1340	406	-1.72	7.25E-13			
FTT_0365	*poxF*	423	129	-1.72	0.01149	S(T)	Mø	(23)
FTT_1303c		430	131	-1.71	0.00246			(48)
FTT_1469c	*nadA*	414	127	-1.71	6.00E-07	S, N	Mø, Mou, DrosC	(6, 16, 26)
FTT_0483c		228	70	-1.70	2.22E-05			
FTT_0240		885	275	-1.69	5.49E-09	N	DrosC	(26)
FTT_1601c	*recG*	2050	639	-1.68	8.95E-08			
FTT_0991		1308	416	-1.65	0.000169			
FTT_0804		2123	676	-1.65	4.07E-11	S(T)	Mø	(23)
FTT_1468c	*nadC*	871	281	-1.63	5.88E-08			
FTT_0106c		1354	441	-1.62	1.23E-07			(29)
FTT_0831c		1454	479	-1.60	5.04E-07	N,	Mø, Dros	(12, 49, 50)
FTT_1154c	*aroB*	1335	441	-1.60	0.028163	N	Mø, DrosC	(16, 26)
FTT_1040		360	120	-1.58	4.78E-06	S, N	Mø, Mou, DrosC	(6, 14, 16, 26)
FTT_0213	*rbn*	1508	512	-1.56	3.72E-10	S	Mø	(6)
FTT_1250		689	235	-1.55	0.003023			
FTT_1608		557	190	-1.55	1.24E-07			

FTT_0809c	*recR*	214	74	-1.54	0.001188	S(T), N	Mø, DrosC	(16, 23, 26)
FTT_0904	*lpnB*	1062	371	-1.52	2.17E-08	N	Mø, Mou	(9, 14, 18)
FTT_1609		457	161	-1.50	0.000118			
FTT_1276	*mglB*	562	199	-1.50	0.000205	N	Mø, Dros	(12, 51)
FTT_1525c		3142	1123	-1.49	0.048928	L, N	Mou	(9, 13)
FTT_1127		420	150	-1.48	2.06E-05	S(T)	Mø	(23, 52)
FTT_1678c	*lepA*	430	157	-1.45	1.59E-05	N	Mø, DrosC	(16, 26)
FTT_1259	*nadE*	1015	374	-1.44	3.91E-05	N	Dros, DrosC	(12, 26)
FTT_0284	*cyoD*	1676	622	-1.43	0.003118	N	Mou	(9)
FTT_0481	*potF*	334	125	-1.42	0.000867			
FTT_1750	*recA*	1110	418	-1.41	7.54E-05	L, S, N	Mø, Mou	(13, 16, 19)
FTT_0863c		779	297	-1.39	0.001822	S(T)	Mø	(23)
FTT_0903		885	345	-1.36	2.22E-05	N	Mou, Dros	(9, 12)
FTT_0161c		2256	891	-1.34	1.30E-07	N	Mø, DrosC	(16, 26)
FTT_0195c		1216	485	-1.32	2.42E-07			
FTT_0456c		895	367	-1.29	2.30E-05	S(T)	Mø	(23)
FTT_1467c	*nadB*	1377	570	-1.27	1.89E-07	N	Mø	(16)
FTT_0356	*htpG*	4911	2065	-1.25	0.000526	S(T), N	Mø, Mou, DrosC	(9, 16, 23, 26, 48)
FTT_1687c	*gidB*	855	360	-1.25	4.56E-05	N	Mou	(9)
FTT_0689		923	389	-1.25	2.76E-05			(38)
FTT_0469	*ksgA*	411	174	-1.24	9.24E-05	N	Mø	(9, 16)
FTT_0907	*parA*	773	328	-1.24	0.016508			
FTT_1224c	*recO*	236	100	-1.24	0.001006	N	Dros, Mou	(12, 14)
FTT_0105c		4535	1933	-1.23	0.000109	L	Mou	(13)
FTT_0290	*moxR*	4895	2136	-1.20	0.001822	L, S	Mø, Mou	(19, 23, 53)
FTT_1401		421	185	-1.19	0.027103	S(T)	Mø	(23, 54)
FTT_1097		803	355	-1.18	0.019484	S(T)	Mø	(23)
FTT_1554c	*truB*	2960	1311	-1.18	2.35E-07	S	Mø	(6)
FTT_1632c		957	424	-1.17	9.98E-05	N	Mø, Mou	(9, 14, 16)
FTT_1434c	*yadH*	617	274	-1.17	0.01359	N	Mø	(16)
FTT_0890c		290	129	-1.17	0.011064	S, N	Mø, Mou, Dros	(6, 12, 55)
FTT_0655		399	182	-1.14	0.004275			
FTT_1534c		2999	1381	-1.12	0.009692			
FTT_0790		1240	577	-1.10	9.94E-05	N	Mou, Dros	(9, 12)
FTT_0634	*hflC*	217	101	-1.10	0.017902			
FTT_0990	*leuS*	346	165	-1.07	0.020816			
FTT_0249	*feoB*	3053	1459	-1.07	6.86E-06	L, N	Mou, Dros	(13, 21)
FTT_1247		980	470	-1.06	0.003318			
FTT_0609		3192	1539	-1.05	1.01E-05	S	Mø, Mou	(19, 23)
FTT_0055	*rluC*	4467	2185	-1.03	0.008941	N	Mø, Mou	(9, 16)
FTT_1334c		1589	781	-1.03	0.036711	S, N	Mø, Mou	(6, 9)
FTT_1370		457	225	-1.02	0.004275	N	Mø	(16)

a Indicates the *Francisella* strain in which this gene has been associated with virulence previously: L = *F. tularensis* subsp. *holarctica* LVS, S = *F. tularensis* subsp. *tularensis* Schu S4, N = *Francisella novicida* U112,. *b* Shows the host or cell line utilised to link the particular gene with virulence: Mø = macrophage, Ep = Epithelial cell line , Mou = Mouse, Hep=hepatic cell line, Dros = *Drosophila melanogaster* DrosC = *Drosophila melanogaster* cell line, S(T)= *F. tularensis* Schu4 through transcriptomics. *c* primary research linking the gene to virulence.

1. Hesselbrock W, Foshay L. 1945. The morphology of *Bacterium tularense*. J Bacteriol 49:209-231.
2. Simon RP, U; Pohler, A. 1983. A broad host range mobilization system for *in vivo* genetic engineering: transposon mutagenesis in Gram-negative bacteria. Nat Biotechnol:784-791.
3. LoVullo ED, Miller CN, Pavelka MS, Kawula TH. 2012. TetR-based gene regulation systems for *Francisella tularensis*. Appl Environ Microbiol 78:6883-6889.
4. Norqvist A, Kuoppa K, Sandstrom G. 1996. Construction of a shuttle vector for use in *Francisella tularensis*. FEMS Immunol Med Microbiol 13:257-260.
5. Maier TA, Casey MS, Becker RH, Dorsey CW, Glass EA, Maltsev N, Zahrt TC, Frank DW. 2007. Identification of *Francisella tularensis* Himar1-based transposon mutants defective for replication in macrophages. Infect Immun 75:5376-5389.

6. Lindemann SR, Peng KT, Long ME, Hunt JR, Apicella MA, Monack DM, Allen LAH, Jones BD. 2011. *Francisella tularensis* Schu S4 O-Antigen and capsule biosynthesis gene mutants induce early cell death in human macrophages. Infect Immun 79:581-594.
7. Brunton J, Steele S, Miller C, Lovullo E, Taft-Benz S, Kawula T. 2015. Identifying *Francisella tularensis* genes required for growth in host cells. Infect Immun 83:3015-3025.
8. Twine SM, Vinogradov E, Lindgren H, Sjostedt A, Conlan JW. 2012. Roles for *wbtC*, *wbtI*, and *kdtA* genes in lipopolysaccharide biosynthesis, protein glycosylation, virulence, and immunogenicity in *Francisella tularensis* Strain SCHU S4. Pathogens 1:12-29.
9. Weiss DS, Brotcke A, Henry T, Margolis JJ, Chan K, Monack DM. 2007. *In vivo* negative selection screen identifies genes required for *Francisella* virulence. Proc Natl Acad Sci U S A 104:6037-6042.
10. Qin AP, Mann BJ. 2006. Identification of transposon insertion mutants of *Francisella tularensis tularensis* strain Schu S4 deficient in intracellular replication in the hepatic cell line HepG2. BMC Microbiol 6.
11. Gesbert G, Ramond E, Tros F, Dairou J, Frapy E, Barel M, Charbit A. 2015. Importance of branched-chain amino acid utilization in *Francisell*a intracellular adaptation. Infect Immun 83:173-83.
12. Moule MG, Monack DM, Schneider DS. 2010. Reciprocal analysis of *Francisella novicida* infections of a *Drosophila melanogaster* model reveal host-pathogen conflicts mediated by reactive oxygen and imd-regulated innate immune response. PLoS Pathog 6.
13. Su JL, Yang J, Zhao DM, Kawula TH, Banas JA, Zhang JR. 2007. Genome-wide identification of *Francisella tularensis* virulence determinants. Infect Immun 75:3089-3101.
14. Kraemer PS, Mitchell A, Pelletier MR, Gallagher LA, Wasnick M, Rohmer L, Brittnacher MJ, Manoil C, Skerett SJ, Salama NR. 2009. Genome-wide screen in *Francisella novicida* for genes required for pulmonary and systemic infection in mice. Infect Immun 77:232-244.
15. Brissac T, Ziveri J, Ramond E, Tros F, Kock S, Dupuis M, Brillet M, Barel M, Peyriga L, Cahoreau E, Charbit A. 2015. Gluconeogenesis, an essential metabolic pathway for pathogenic *Francisella*. Mol Microbiol 98:518-34.
16. Asare R, Abu Kwaik Y. 2010. Molecular complexity orchestrates modulation of phagosome biogenesis and escape to the cytosol of macrophages by *Francisella tularensis*. Environ Microbiol 12:2559-2586.
17. Reynoso CM, Miller MA, Bina JE, Gallivan JP, Weiss DS. 2012. Riboswitches for intracellular study of genes involved in *Francisella* pathogenesis. MBio 3.
18. Llewellyn AC, Jones CL, Napier BA, Bina JE, Weiss DS. 2011. Macrophage replication screen identifies a novel *Francisella* hydroperoxide resistance protein involved in virulence. PLoS One 6.
19. Kadzhaev K, Zingmark C, Golovliov I, Bolanowski M, Shen H, Conlan W, Sjostedt A. 2009. Identification of genes contributing to the virulence of *Francisella tularensis* SCHU S4 in a mouse intradermal infection model. PLoS One 4:e5463.
20. Quarry JE, Isherwood KE, Michell SL, Diaper H, Titball RW, Oyston PC. 2007. A *Francisella tularensis* subspecies *novicida purF* mutant, but not a *purA* mutant, induces protective immunity to tularemia in mice. Vaccine 25:2011-8.
21. Ahlund MK, Ryden P, Sjostedt A, Stoven S. 2010. Directed screen of *Francisella novicida* virulence determinants using *Drosophila melanogaster*. Infect Immun 78:3118-3128.
22. Mahawar M, Rabadi SM, Banik S, Catlett SV, Metzger DW, Malik M, Bakshi CS. 2013. Identification of a live attenuated vaccine candidate for tularemia prophylaxis. PLoS One 8:e61539.
23. Wehrly TD, Chong A, Virtaneva K, Sturdevant DE, Child R, Edwards JA, Brouwer D, Nair V, Fischer ER, Wicke L, Curda AJ, Kupko JJ, 3rd, Martens C, Crane DD, Bosio CM, Porcella SF, Celli J. 2009. Intracellular biology and virulence determinants of *Francisella tularensis* revealed by transcriptional profiling inside macrophages. Cell Microbiol 11:1128-50.
24. Alkhuder K, Meibom KL, Dubail I, Dupuis M, Charbit A. 2009. Glutathione provides a source of cysteine essential for intracellular multiplication of *Francisella tularensis*. PLoS Pathog 5:e1000284.
25. Ireland PM, LeButt H, Thomas RM, Oyston PC. 2011. A *Francisella tularensis* SCHU S4 mutant deficient in gamma-glutamyltransferase activity induces protective immunity: characterization of an attenuated vaccine candidate. Microbiology 157:3172-9.
26. Asare R, Akimana C, Jones S, Abu Kwaik Y. 2010. Molecular bases of proliferation of *Francisella tularensis* in arthropod vectors. Environ Microbiol 12:2587-2612.
27. Rasmussen JA, Fletcher JR, Long ME, Allen L-AH, Jones BD. 2015. Characterization of *Francisella tularensis* Schu S4 mutants identified from a transposon library screened for O-antigen and capsule deficiencies. Front Microbiol 6.
28. Lai XH, Shirley RL, Crosa L, Kanistanon D, Tempel R, Ernst RK, Gallagher LA, Manoil C, Heffron F. 2010. Mutations of *Francisella novicida* that alter the mechanism of its phagocytosis by murine macrophages. PLoS One 5:e11857.
29. Qin A, Scott DW, Mann BJ. 2008. *Francisella tularensis* subsp. *tularensis* Schu S4 disulfide bond formation protein B, but not an RND-type efflux pump, is required for virulence. Infect Immun 76:3086-92.

30. Ma Z, Banik S, Rane H, Mora VT, Rabadi SM, Doyle CR, Thanassi DG, Bakshi CS, Malik M. 2014. EmrA1 membrane fusion protein of *Francisella tularensis* LVS is required for resistance to oxidative stress, intramacrophage survival and virulence in mice. Mol Microbiol 91:976-95.
31. Jia Q, Lee BY, Bowen R, Dillon BJ, Som SM, Horwitz MA. 2010. A *Francisella tularensis* live vaccine strain (LVS) mutant with a deletion in *capB*, encoding a putative capsular biosynthesis protein, is significantly more attenuated than LVS yet induces potent protective immunity in mice against *F. tularensis* challenge. Infect Immun 78:4341-55.
32. Michell SL, Dean RE, Eyles JE, Hartley MG, Waters E, Prior JL, Titball RW, Oyston PC. 2010. Deletion of the *Bacillus anthracis capB* homologue in *Francisella tularensis* subspecies *tularensis* generates an attenuated strain that protects mice against virulent tularaemia. J Med Microbiol 59:1275-84.
33. McLendon MK, Schilling B, Hunt JR, Apicella MA, Gibson BW. 2007. Identification of LpxL, a late acyltransferase of *Francisella tularensis*. Infect Immun 75:5518-31.
34. Gil H, Platz GJ, Forestal CA, Monfett M, Bakshi CS, Sellati TJ, Furie MB, Benach JL, Thanassi DG. 2006. Deletion of TolC orthologs in *Francisella tularensis* identifies roles in multidrug resistance and virulence. Proc Natl Acad Sci U S A 103:12897-902.
35. Llewellyn AC, Zhao J, Song F, Parvathareddy J, Xu Q, Napier BA, Laroui H, Merlin D, Bina JE, Cotter PA, Miller MA, Raetz CR, Weiss DS. 2012. NaxD is a deacetylase required for lipid A modification and *Francisella* pathogenesis. Mol Microbiol 86:611-27.
36. Ulland TK, Janowski AM, Buchan BW, Faron M, Cassel SL, Jones BD, Sutterwala FS. 2013. *Francisella tularensis* live vaccine strain folate metabolism and pseudouridine synthase gene mutants modulate macrophage caspase-1 activation. Infect Immun 81:201-8.
37. Maier TM, Pechous R, Casey M, Zahrt TC, Frank DW. 2006. *In vivo* Himar1-based transposon mutagenesis of *Francisella tularensis*. Appl Environ Microbiol 72:1878-1885.
38. Richards MI, Michell SL, Oyston PC. 2008. An intracellularly inducible gene involved in virulence and polyphosphate production in *Francisella*. J Med Microbiol 57:1183-92.
39. Horzempa J, O'Dee DM, Shanks RM, Nau GJ. 2010. *Francisella tularensis* DeltapyrF mutants show that replication in nonmacrophages is sufficient for pathogenesis *in vivo*. Infect Immun 78:2607-19.
40. Enstrom M, Held K, Ramage B, Brittnacher M, Gallagher L, Manoil C. 2012. Genotype-phenotype associations in a nonmodel prokaryote. MBio 3.
41. Straskova A, Pavkova I, Link M, Forslund AL, Kuoppa K, Noppa L, Kroca M, Fucikova A, Klimentova J, Krocova Z, Forsberg A, Stulik J. 2009. Proteome analysis of an attenuated *Francisella tularensis dsbA* mutant: identification of potential DsbA substrate proteins. J Proteome Res 8:5336-46.
42. Qin A, Scott DW, Rabideau MM, Moore EA, Mann BJ. 2011. Requirement of the CXXC motif of novel *Francisella* infectivity potentiator protein B FipB, and FipA in virulence of *F. tularensis* subsp. *tularensis*. PLoS One 6:e24611.
43. Barrigan LM, Tuladhar S, Brunton JC, Woolard MD, Chen CJ, Saini D, Frothingham R, Sempowski GD, Kawula TH, Frelinger JA. 2013. Infection with *Francisella tularensis* LVS *clpB* leads to an altered yet protective immune response. Infect Immun 81:2028-42.
44. Meibom KL, Dubail I, Dupuis M, Barel M, Lenco J, Stulik J, Golovliov I, Sjostedt A, Charbit A. 2008. The heat-shock protein ClpB of *Francisella tularensis* is involved in stress tolerance and is required for multiplication in target organs of infected mice. Mol Microbiol 67:1384-401.
45. Conlan JW, Shen H, Golovliov I, Zingmark C, Oyston PC, Chen W, House RV, Sjostedt A. 2010. Differential ability of novel attenuated targeted deletion mutants of *Francisella tularensis* subspecies *tularensis* strain SCHU S4 to protect mice against aerosol challenge with virulent bacteria: effects of host background and route of immunization. Vaccine 28:1824-31.
46. Twine S, Shen H, Harris G, Chen W, Sjostedt A, Ryden P, Conlan W. 2012. BALB/c mice, but not C57BL/6 mice immunized with a Δ*clpB* mutant of *Francisella tularensis* subspecies *tularensis* are protected against respiratory challenge with wild-type bacteria: association of protection with post-vaccination and post-challenge immune responses. Vaccine 30:3634-45.
47. Dankova V, Balonova L, Straskova A, Spidlova P, Putzova D, Kijek T, Bozue J, Cote C, Mou S, Worsham P, Szotakova B, Cerveny L, Stulik J. 2014. Characterization of tetratricopeptide repeat-like proteins in *Francisella tularensis* and identification of a novel locus required for virulence. Infect Immun 82:5035-48.
48. Twine SM, Mykytczuk NC, Petit MD, Shen H, Sjostedt A, Wayne Conlan J, Kelly JF. 2006. *In vivo* proteomic analysis of the intracellular bacterial pathogen, *Francisella tularensis*, isolated from mouse spleen. Biochem Biophys Res Commun 345:1621-33.
49. Robertson GT, Case EDR, Dobbs N, Ingle C, Balaban M, Celli J, Norgard MV. 2014. FTT0831c/FTL_0325 contributes to *Francisella tularensis* cell division, maintenance of cell shape, and structural integrity. Infect Immun 82:2935-2948.

50. Mahawar M, Atianand MK, Dotson RJ, Mora V, Rabadi SM, Metzger DW, Huntley JF, Harton JA, Malik M, Bakshi CS. 2012. Identification of a novel *Francisella tularensis* factor required for intramacrophage survival and subversion of innate immune response. J Biol Chem 287:25216-29.
51. Baron GS, Nano FE. 1998. MglA and MglB are required for the intramacrophage growth of *Francisella novicida*. Mol Microbiol 29:247-259.
52. Brotcke A, Weiss DS, Kim CC, Chain P, Malfatti S, Garcia E, Monack DM. 2006. Identification of MglA-regulated genes reveals novel virulence factors in *Francisella tularensis*. Infect Immun 74:6642-6655.
53. Dieppedale J, Sobral D, Dupuis M, Dubail I, Klimentova J, Stulik J, Postic G, Frapy E, Meibom KL, Barel M, Charbit A. 2011. Identification of a putative chaperone involved in stress resistance and virulence in *Francisella tularensis*. Infect Immun 79:1428-39.
54. Lindgren H, Lindgren L, Golovliov I, Sjostedt A. 2015. Mechanisms of heme utilization by *Francisella tularensis*. PLoS One 10:e0119143.
55. Zogaj X, Chakraborty S, Liu JR, Thanassi DG, Klose KE. 2008. Characterization of the *Francisella tularensis* subsp *novicida* type IV pilus. Microbiology 154:2139-2150.

Figure S2 *F.tularensis* Schu S4 predicted essential gene DNA sequences in FastA format

```
>lcl|NC_006570.2_cds_YP_169084.1_1 [gene=dnaA] [protein=chromosome replication
initiator DnaA] [protein_id=YP_169084.1] [location=1..1521]
TTGGCTATCCTTTATATTTTAAGGGTTATTAGGATATTTTTTATTATGACTACATGGGATAAATGTTTAA
AAAAAATAAAAAAAAACCTTTCTACGTTTGAGTATAAGACGTGGATAAAGCCTATCCATGTGGAGCAAAA
TAGTAACTTATTCACAGTTTACTGTAACAATGAATATTTCAAAAAACATATAAAATCTAAGTATGGAAAT
CTTATTTTATCAACAATCCAAGAGTGTCATGGTAATGATTTAATTATTGAATATTCTAATAAAAAATTCT
CTGGCGAAAAAATTACTGAGGTTATCACAGCTGGACCACAAGCTAATTTTTTTAGCACAACAAGTGTTGA
GATAAAAGATGAATCAGAAGATACAAAAGTAGTACAAGAACCTAAAATATCAAAGAAGTCTAATAGTAAA
GACTTTTCTTCATCACAAGAGTTATTCGGTTTTGACGAAGCTATGCTAATTACAGCAAAAGAAGATGAGG
AATACTCTTTTGGTTTACCGTTAAAAGAAAAATATGTTTTTGATAGTTTTGTTGTTGGAGATGCTAACAA
AATTGCTAGAGCAGCAGCTATGCAGGTATCGATAAATCCAGGTAAATTACATAACCCTTTATTCATTTAT
GGTGGTAGTGGTTTAGGTAAAACTCACTTAATGCAAGCAATAGGTAATCATGCAAGAGAAGTTAATCCTA
ATGCCAAAATTATTTATACAAATTCAGAACAATTTATTAAAGATTATGTAAATTCTATTCGTTTACAAGA
TCAAGATGAGTTTCAAAGAGTTTATAGATCTGCGGATATACTTTTGATTGATGATATTCAATTTATCGCT
GGTAAAGAGGGTACTGCTCAGGAGTTTTTCCATACTTTTAATGCATTGTATGAAAATGGTAAACAGATAA
TTCTAACTAGTGATAAGTATCCAAATGAAATAGAAGGGCTTGAAGAAAGACTAGTTTCGCGTTTTGGTTA
TGGTTTAACAGTTTCTGTTGATATGCCAGATTTAGAAACCAGAATTGCTATCTTGCTCAAAAAAGCTCAT
GATTTAGGTCAGAAATTACCTAACGAAACAGCAGCTTTTATTGCTGAGAATGTACGTACTAATGTCAGAG
AACTAGAAGGTGCTCTAAATAGGGTTCTTACTACCTCTAAATTTAATCATAAAGATCCTACTATCGAAGT
AGCACAAGCTTGCTTAAGAGATGTTATAAAAATACAAGAAAAGAAAGTAAAAATAGATAATATCCAAAAG
GTTGTTGCTGATTTTTATAGAATCAGGGTAAAAGATTTAACTTCTAATCAAAGAAGTAGAAATATAGCTA
GACCAAGACAGATAGCAATGAGTTTAGCACGTGAACTAACATCACATAGTTTGCCAGAAATAGGCAATGC
TTTTGGTGGTAGAGACCATACGACAGTTATGCATGCTGTCAAAGCTATAACTAAATTAAGACAAAGCAAT
ACTTCAATATCGGATGATTATGAGTTGCTTTTAGATAAAATTTCTCGTTAA

>lcl|NC_006570.2_cds_YP_169085.1_2 [gene=dnaN] [protein=DNA polymerase III subunit
beta] [protein_id=YP_169085.1] [location=1558..2661]
ATGAATTTTGTACTAAATAGAGATGACTTACTAAAGCCTTTGCAATCTATGCTCTCAGTTGCAAATAGTA
AGAGTACAATGCCTTTATTATCATGTATCTTATTTGATATTGATAATAATAATCTCAAAATTACGGCTTC
GGATCTTGATACAGAGATATCATGCAATATAGCAGTTAGTTGTAACACAACTATTAAGTTAGCATTAAAT
GCTGACAAAATTTATAACATTGTCAGAAGCTTAAATGAAAATTCAATGATTGATTTTAGAATTAATGAAA
ATAAGGTAACTATTGTTTCTAATAATAGTACTTTTAACCTTATATCACTAAATGCTGACAACTATCCTCT
TATTGATAGTAATATCAATGAGCAAGCAAGTTTTGATCTTTCTCAACAAGATTTTCATCATATTATTTCA
AAAGTAGATTTCTCAATGGCTAATGATGATACTCGATATTTCTTAAATGGGATGTTTTGGGAAATCAACG
CAAATCTACTAAGAGCAGTATCTACAGATGGTCATAGAATGTCTATCACAGAGGCTATAATTGATAGTAA
AGTGTTAGATAGTGCTTCTCAGTCGATAATTCCAAAAAAAGCGATTTTAGAGCTTAAAAAGATAGTTGGC
AAAACAGAAGAAAATATCAAAATTTGTCTTGGCAAAAATTATCTAAAAGCGATTTTTGGTAATTATGCTT
TTATATCAAAGCTTATAGATGGTCGCTATCCTGATTACCAAAAAGTAATCCCTAAAAATAATACAAAACT
ATTAGCAGTTGATAAGCAGTTTTTCAAAAATTCATTATTAAGAACATCAATACTTGCTAATGATAAATAT
AAAGGTGTTCGTCTTAACATATCTCAAAATCAATTACTTCTATCAGCTAATAACCCTGATAATGAAAAGG
CTGAAGATAAAATCGAAGTTCAATATAATGATCAACCAATGGAAATTTGTTTTAATTACAAATATCTTTT
GGATATTATAAATGTACTTAGTGAAGAAACTATGTCTATCTACCTTGATAATCCAAATATGAGTGCTTTA
GTCAAAGATGAGAAAGATAATAGTTTGTTTATTATTATGCCAATGAAAATTTAA
```

>lcl|NC_006570.2_cds_YP_169088.1_5 [gene=aspS] [protein=aspartyl-tRNA synthetase] [protein_id=YP_169088.1] [location=6044..7822]
TTGGATATGAGAACACATTATAGTTCAGATATTAATGAAAAATTACAAGGACAAAAAGTTACAGTATGCG
GTTGGGTTCATCGTCGTAGAGATCACGGTGGTGTTATTTTCTTAGATATTAGAGATAGAACTGGTTTGGT
ACAATTAGTTTTTAACCCTGATAATGACAATTTCAAAGTTGCTGATAGTTTAAGATCTGAATTTGTGATT
AAAGCTGAAGGTGTTGTTAACTTAAGACCAGAAGGTCAAGAAAACAAAAATATTTCTAGTGGTAAAGTTG
AGATAATTGGTGATAGTATAGAGGTTATTAATAAATCTAAAACTATTCCATTTCAGTTAGATGATTTCCA
ATCAACAGGCGAAGATGTCAAACTTAAGTATCGTTATATTGATCTGCGTCGTCCAGAAATGCAACATAAA
TTAATTACGCGTTCAAAGGCGATTAGATATGTACGTAACTTTCTAGATAATAATGGTTTTCTTGATATCG
AAACGCCATTTTTAACAAAAGCAACACCAGAGGGTGCTAGAGATTATCTTGTGCCAAGTCGTAATTTTAA
TGGTAAGTTTTATGCATTGCCGCAATCGCCACAACTTTTCAAACAGCTATTGATGGTTTCAGGTTTTGAT
AGATATTATCAAATCGTTAAATGTTTCCGTGATGAAGATTTAAGAGCTGATAGACAGCCAGAATTTACCC
AGATAGATATTGAAGCTTCATTTATTGATGAGGCTTTTATCATGTCGACTATGGAGAGAATGATAGCGGG
ACTTTTCAAAGAAACTATCGGTGTTGAGTTTGCAACTCCTTTCCAAGTAATGACTTTTGCTGATGCTATT
GATAAGTATGGTTCAGATAAACCTGATCTAAGAATTCCACTTGAATTTGTAAATATCAAAGAAGATATGC
AAAACGAAGAGTTTAAGGTATTTTCAGGTCCAGCAAATGATCCACAATCGCGAGTTATTGCGCTGAGAAT
TCCTGGCGGTAATGATAAGCTTACGCGTAAGATGATCGATGAGTATACCAAGTTTGTTGGTATCTACGGT
GCTAAAGGTCTTGCATATATCAAGATTAATTCATTATCACAAGGCAAAGAGGGCTTACAATCACCAATTG
TAAAAAATATCTCCGAAGAAACCTTGTTCAAAGTGATTGATAAAACTAGCGCTAAAGAGGGTGATTTATT
ATTCTTTGGTGCTGGTAAGGCTAAGATTGTAAATGATTCAATGGGTGCATTAAGAGCTAAGATTGGTGAA
GATCTTGATCTATTTAATAAAGATTGGGCACCATTATGGGTTGTTGATTTCCCAATGTTTGAGAAAGATG
ATAATCGTTTATATGCAATGCATCATCCATTTACAGCTCCTAAAGTTAGTTCAGTTGAAGACTTAGTTAA
TACTAATCCAGAAGAGCTAAGCTCAAGAGCATACGATATGGTTATTAATGGTTATGAAGTAGGTGGTGGA
TCTATCCGTATTCATAAACAAGATATCCAAGCAAAAGTATTTAACTTATTGGGCATTTCTGATGATGAAG
CTCGTGAGAAGTTTGGCTTTATGCTTGACGCTTTATCATATGGTACACCTATTCATGGTGGTATAGCTTT
TGGTATTGATAGACTTATTATGCTGCTTACAGGAACCACAAATATCCGTGATGTAATAGCATTCCCTAAA
ACTCAAACGGCGAGCTGTTTAATGACAGAAGCACCATCAACAGTTTCTTTAGAGCAGCTTAATGAGCTTG
GTATAGCTGTTAAGAAAGAAGAAAGGTAA

>lcl|NC_006570.2_cds_YP_169091.1_8 [gene=purB] [protein=adenylosuccinate lyase] [protein_id=YP_169091.1] [location=13182..14480]
ATGATAAAAAGATATGACGTAGCAGAAATCTCAAAAATTTGGGCAGATGAGAATAAATATGCAAAAATGT
TAGAGGTTGAGCTTGCGATTTTAGAGGCACTTGAAGATAGAATGGTACCTAAAGGTACAGCTGCGGAAAT
TCGTGCTAGAGCACAAATTAGACCTGAGAGAGTTGATGAGATTGAGAAAGTTACAAAGCATGACATCATC
GCATTTTGTACCTCTATTGCGGAGCAATTTACAGCTGAAACTGGCAAGTTTTTTCATTTTGGTGTTACAT
CTTCAGATATTATTGACTCTGCTCTTAGCCTACAAATTCGTGATTCTATGAGCTATGTTATCAAAGATTT
AGAGGCGCTTTGTGACTCGCTACTTACTAAGGCAGAAGAAACAAAAGAAATCATTACGATGGGTAGAAGT
CATGGTATGTTTGCCGAACCGATGAGCTTTGGTCAGAAGTTTCTTGGTGCTTATGTTGAGTTTAAGCGTA
GGCTAAAAGATCTCAAAGATTTTCAAAAAGATGGTTTGACAGTACAGTTCTCAGGAGCTGTAGGTAACTA
TTGTATTTTAACTACAGAAGATGAGAAAAAAGCAGCTGATATTTTAGGTTTACCTGTTGAAGAAGTTTCT
ACTCAAGTTATCCCAAGAGATAGAATTGCTAAGTTGATATCTATCCACGGACTTATTGCTTCTGCTATAG
AGAGATTAGCAGTTGAGATTAGACATTTACATCGTAGTGATGTTTTTGAGGTATATGAAGGCTTCTCTAA
AGGGCAAAAAGGCTCATCAACTATGCCGCATAAGAAAAACCCAATTTCTACTGAAAACTTAACAGGTATG
GCAAGAATGCTAAGATCTCATGTATCTATAGCATTAGAGAATTGTGTGCTATGGCATGAGCGAGATATTT
CTCACTCTTCAGCAGAGCGTTTTTATCTGCCAGATAACTTTGGTATTATGGTTTATGCTTTACGTAGAAT
GAAAAATACTATCGATAATCTCGTCGTGCAAAGAGATATTATCGAAGATAGGGTTAGAAGTACTAGTGCT
TATTTATCAAGTTTTTACTTACATTTCTTGGTAGCAAATACGCCATTTATGCGTGAAGATTGCTACAAGA
TTGTTCAGCAAGTTGCTTTTGATCTTAAACAAGGAGAATCTTTCTCGAAGAAATTACAAAAAGTTATGCA
CGATGAGCATAATATTATTTTAGATATTCCAGAGATGGATTTTGAGGGTATCAAGAAAACTTACCTAAAA
GAAATTGATCATGTTTTTGATAGATCTGTTAAGGCGTAA

>lcl|NC_006570.2_cds_YP_169095.1_12 [gene=gatC] [protein=Glu-tRNAGln amidotransferase C subunit] [protein_id=YP_169095.1] [location=17884..18165]
ATGGATAAAAAATTAGTAAAACATATTGCTAAACTATCTTGTTTTGATTTGACTGAAGAGCAGTTGGAGC
AGTATACCAAAGATCTTATCAATATCTGTAAGATTCTTGATACAGTCAAAAATTTCGATGCTCAAGGAGT
CAAGCCGATGATATCACCAATTAGTGTTGATTTTAAATTTCGTGAAGATATCCCTCAAGATCAAGATAAT
CGTACAAGTTTTGATAAATTCGCTTGTGAAGTTGTTGATGATTACTTTATGGTGCCACAAGTTGTTAAAT
AG

>lcl|NC_006570.2_cds_YP_169096.1_13 [gene=gatA] [protein=aspartyl/glutamyl-tRNA amidotransferase subunit A] [protein_id=YP_169096.1] [location=18174..19619]
ATGTCATATATTAAGAAATTAAGAGCTAGATTAGATAGTGGTGAAATAAGCGCTGTTGAACTAACTAAAG
AGTATCTAGCAAAAATTAAAGAGCAAGATAAGCGCATAAACTCTGTAATCACTCTATGTGAAGCAGAAGC

ACTAAAAGAAGCAGAAGATGCTGATGCTATTATTTCTGCAGGTAAGCAAGGATTATTAACAGGAATTCCT
ATTCTACATAAGGATCTTTTTTGCACTAAAGGTATAAGAACTACGGCTGCCTCTAAGATGTTGGATAATT
TCGTAGCACCATATGACTCAACAGTGACAAAAAACTGTAAGGATCAAGGTATGGTCACTCTAGGCAAACT
AAATATGGATGAGTTTGCTATGGGTTCAACTAACGAGTATAGCTATTATGGAGCAGTTAGTAATCCTTGG
GATTTAGAAAGAGTTCCGGGAGGTTCATCAGGTGGCTCAGCTGCAGCAGTTGCTGCAGGTTTTGCGCCTA
TAAGTACAGGGTCAGATACTGGTGGATCGGTGAGACAGCCGGCTAGTTTTTGTGGTCTTACAGCAATGAA
GCCTAGTTATGGAAGTACTTCAAGGTTTGGCATGGTAGCGTTTGCATCATCTTTTGATCAAGCTGGTGTT
TTAGGACACTATGCTGAAGATGTTGCGATCATGTTAGATGCAATTGCTGGCGAGTGTGAGTTTGATTCTA
CTTGTGTTGGTGTTAAACAAAATCATTTCACCCAAGATCTAGAAAAAGATATCTCTGGTAAAGTAATTGG
CGTTGATGAGAGTCTAATCAAAGACCTACCAGCACAAATACAAGAAGCAGTATCAAAAACTCTTGATAAT
TTCAAAAAGCTAGGTGCTGAAATAAAGTCGGTCAAGGTCCCAGATTTAAAAGAAGCTCTTTCAACTTATT
ATATTATAACTCCAGCAGAAGCAGCAGCTAACTTAGCTAGATATGATGGTATTAGATATGGTTACCGTAA
TCCAGAAGCTAGAGATTTAGATGAATTATATAGAAAATCGCGAACAGATGGTTTTGGTGCAGAAGTTAAG
CGTAGAATTATGATTGGTAACTATGTCCTAGCATCGAGTCAGTATGATTCTTACTACAATAAGGCTCAGC
AGCTACGTAAGGTGATGACTGATCAAATTAATCAAATATTTACCCAAGTTGATGCTATCTTTATGCCAGC
ATCTCCAAGTGAGGCATTTAAAAAAGGAGATAAGCTAGATCCTGTTTCGGCATATTTATCAGATATTTAC
ACTATTCCTGCTAACATATCAGGCCTACCAGCTATTGCTTTCCCAATTGGTTTTGCTAATAATTTACCAG
TTGGTGGACAACTTATGGCTAAAGCATTTAATGATAATATCTTGACGCAGATGGTAGTGCAGTATCAAAA
GCATTATGGTATTGAAGAATTTATTTTACAACAAGCGAGGATTTAA

>lcl|NC_006570.2_cds_YP_169097.1_14 [gene=gatB] [protein=aspartyl/glutamyl-tRNA amidotransferase subunit B] [protein_id=YP_169097.1] [location=19622..21043]
GTGAATTGGGAAATGGTGATAGGGCTAGAAGTCCATATTCAATTAAGTACTAAATCTAAGCTATTTTCGA
CTTCTGCAACAAAGTATGGTCAACATCAAAATACTCAAGCAGCATTTTTAGACTTAGGTTTGCCTGGAAC
GTTGCCTGTTGTAAATAAAGAAGCTATTCGTAAAGCAGTGATATTTGGCTTAGCTGTAGATGCTAAGATA
TCAAAGGATAGTTTCTTTGCGCGAAAAAATTATTTCTATCCAGATTTATCAAAAGGTTATCAGATTAGTC
AATCAACTAATCCAATAGTCCAAGAAGGTAGATTAGAGATTGAAACTTCAAAAGGACTAAAAACTATCAG
AATTGAGCGTGCACATTTAGAAGAAGATGCCGGTAAGTCTGTGCATGGTTATATTGCTGGTGAGACAGGA
TTAGATTACAATCGTGCTGGTACACCACTTTTAGAGATTGTGACATATCCTGATTTTAGATCGGCTGAAG
AAGTGGTTGCATATCTTAAAAAACTTCATCAGCTAGTTAAGCATTTAGGTATTTGCGATGGTAACATGCA
AGAAGGTTCGTTTAGATGTGATGTTAACTTGTCAATTAGACCACAAGGACAAGCTAAGTTTGGTACACGT
GCAGAGCTTAAAAATATAAACTCATTTAGGTTTATTGATAAAGCTATCGAGTATGAGTATGCTCGCCAAG
TTAGTGTGTTGGAGTCAGGGGGCGAAGTTGTCCAGGAAACGCGTCTTTATGATGCAGATGCTAATGAGAC
GCGCTCAATGCGTGCAAAAGAAGATGCTTTTGATTATCGTTATTTCCCAGATCCAGATTTATTGCCATTG
GTGATAACCGATGAGTATATCGAGAGTATCAAAAAACAAATGCCACTGAAATCGGAAGAGCGTGAAGCGG
TATATCGTGAGCATCTAGCTGAGCAGGAAGTTGAGTTTTTATTATCAAATCTTGAGATAGCTGATTACTA
TGACAAAGTAGCAGTTGTGATAGGATACAAGCCAGCTTATAATTGGATAACAGTTGATCTAATATCAACT
CTTAATAGGGCTGAAAAGGAGTTTTCTAGTGATGTTGTTCCTGCTGAGATTCTGCTAGAAATTATTGCTA
ATGTCCAAAAAGATATAATATCTCAAGCTAATGCTAAGAAAGTGATAGCAGAATATATTGATGCTCCTAG
TGCAATTGAAGCTATTATTGAAAAGCTCGGTCTAAAACAAGTATCTGATGAGGGTATGATTCGCGAGTTA
GTTCAAGGTATAATTGCTGCTAACCCTCAACAAGCAGCTGACTTTAAAGCTGGTAAAACAAAGCTTATGA
GCTTTTTTGTTGGGCAAGCTATGAAAGCAAGTAAAGGTAAAGCAAATCCTAAGCAGGTTAATCAAATAGT
TCAAGAAGAGCTAAATAAATAA

>lcl|NC_006570.2_cds_YP_169107.1_22 [gene=nuoA] [protein=NADH dehydrogenase I subunit A] [protein_id=YP_169107.1] [location=31160..31558]
ATGAGTACAAGTGTTTATGAGCAATTTGCCCCTATACTGATATTCTTGATTATAGCTTTCGGTTTAGGTG
CTGCTTTTGCAATTATTGGCAAAGTTTTATCAGTGATTGTTGGCGCAAATAATCCAAATAAAACCAAAGG
AGAAACATTTGAATGTGGTTTTCCGACTTTTGGTGATGCAAGAGAGAAACTTGATGTGCGTTTTTATCTA
ATTGCAGTATTGTTTTTGGTTTTTGACTTGGAGTTGGCATTTATTATCCCATGGGGAATAAATCTACGAG
CTAGTGCAGGAATGCCAGCTATTTCAGATCATGCATTTTTTGCGATGATTATATTTTTAGTGGTGTTATT
CTTGGGCTTAATATACGCCTGGAAGAAAGGAGCTTTAGAGTGGGAATAG

>lcl|NC_006570.2_cds_YP_169108.1_23 [gene=nuoB] [protein=NADH dehydrogenase subunit B] [protein_id=YP_169108.1] [location=31549..32025]
GTGGGAATAGGTAACGAAAACAAAGGTTTTATAACTGCAAGTGCGGATGCACTTATAAACTGGGTGCGTA
CAGGATCTTTATGGCCGGTAACAACTGGTTTGGCTTGTTGTGCTGTAGAAATGATGCACGCAGGTGCGGC
TAGGTATGATCTGGATAGGTTTGGTATAGTCTTTAGGCCTTCTCCAAGGCAGTCTGATGTGCTTATTGTT
GCTGGGACTCTTTGCAATAAAATGGCTCCGGCGCTACGCCAAGTGTATGATCAAATGCCTGACCCTAAGT
GGGTAATTTCTATGGGATCTTGTGCAAATGGTGGTGGTTATTATCATTACTCGTACTCGGTGGTTAGGGG
TTGTGATAGGATTGTGCCTGTTGATATATATGTGCCAGGTTGTCCTCCTACTGCTGAGGCTTTAGTTTAC
GGCATTATACAACTGCAGAATAAAATTATTAGAAAAGATACTATAGCGAGGAAGTGA

>lcl|NC_006570.2_cds_YP_169109.1_24 [gene=nuoC] [protein=NADH dehydrogenase I] [protein_id=YP_169109.1] [location=32022..32672]
GTGATCGTGAGTACTAAATTACAAGATCATTTTGATAAAATAACAAAGATTTTGAGTGGCTTTGGCGTTG
AGGGCTGTATATCTTATGGCGAGATAACTTTTTCTATCAGGGATCAGCGTGATATTCATTTAATTTTAAA
AAAACTTAAAAAAGAGTATCTTTTTGAGCAGCTTACAGACGTAACAGCTGTTGATTATTTGACCTATGGT
CAGTCAGACTGGCAGGTTGGTAAGGTTGTTTCACAAACAGGTTTTTCTAGAGGGCGTCAGCAAGATTTCA
AAACCGCTGCTGTGGATAATAGATTTGAAATTATTTATCAGCTACTAAGTATGGCAAATAATGTCAGAAT
TAGAGTTAAGTGTAAACTAAAAGATGCGCAGATCATTTTGGTTGATTCTGTCAGTGATTTATGGCCATCA
GCTAACTGGGCTGAGAGAGAAGTCTACGATATGTTTGGAATTTACTTTAATAATCATCCTGATTTAAGAA
GAGTGCTTACTGATTATGGTTTCGTTGGACACCCTTTGAGAAAAGATTTCCCGCAAACAGGCTATGTTGA
GATGCGTTATGATGAAAACCTTGGTCGAGTTGTGTATGAGCCGGTAGAGATTGATGATAGGGTTAATACA
CCAAGAGTGATTCGTAACTAA

>lcl|NC_006570.2_cds_YP_169110.1_25 [gene=nuoD] [protein=NADH dehydrogenase subunit D] [protein_id=YP_169110.1] [location=32694..33947]
ATGGCAGAGTATAAAAACTATACACTAAATTTTGGACCAGTTCATCCTGCAGCGCATGGTGTTCTTAGGC
TTATTTTAGAACTTGATGGCGAAAATGTTGTGCGAGCTGATCCACACGTTGGATTGTTGCATAGAGGTAC
AGAAAAACTTGCTGAATTTAAGCCATATAACCAAAGTATTGGTTATATGGACAGGCTTGATTATGTCTCG
ATGATGTGTAATGAGCATGCTTATGTGATGGCGATTGAAAAGCTTTTACAGTTAGAAGTTCCCGAGAGGG
CTAAATATATTCGCGTAATGTTTGCTGAGATGACTAGAATTTTAAATCATCTATTATGGGTTGCAGCATG
TGGTATCGACCTTGGTGCAATGACTGTGTTTTTGTATGCTTTTAGGGTCAGAGAAGATTTGTTTGATTGC
TATGAGGCTGTATCTGGTGCACGTATGCATGCGGCATATTTTAGACCTGGTGGTGTTGCTAGAGATTTGC
CAACGCAGATGCCTCAATATCAAAAGACTAGATTTACTAGTAAGAGAAAAGCAAAGAAATTAAATGAGCC
AAGGCAAGGAAGTATGCTTGATTTCTTGGATCATTTTGTTGTTGATTTTGAGAAATCTCTAGATGAGATT
GATACTCTTTTAACTGATAATAGATTGTGGAAGCAGCGGACGGTTGATATTGGTACTGTAACTGCTGAGA
GGGCTAAAGAGCTTGGTTTTACAGGCCCAATGTTAAGAGGAAGTGGAGTTGCGTGGGATCTTAGAAAAAC
TCAGCCTTATGAGGTGTATCATAAGCTGGAGTTTGATATTCCAATTGGTGCAAATGGTGACTGTTATGAT
AGGTATCTTGTCAGAATGGCTGAGATGCGTGAGTCAAATAAGCTTATCAAACAATGTGTTGATTGGCTTA
GGGCTAATCCAGGACCTGTGTTGTCAGATAATAATAAGGTTGCGCCTCCTAAGAGAAATGCGATGAAAAA
TAATATGGAAGAGCTTATACATCACTTTAAACTTTTCTCAGAAGGCTATTGTACTACTGAGGGTGAGGTT
TATGTTGGTACTGAACATCCAAAAGGTGAGTTTGGTGTGTATATCAAATCAGATGGTGCAAATAAGCCGT
ATAGATTAAAAATGAGAGCGCCAGGATTTGCACATATTAGTGCAATGGATGAATTATTATCTGGGCATAT
GTTAGCAGATACTCCAGCGATTATCTCAACAATAGATGTCGTATTTGGTGATGTAGATAGATAG

>lcl|NC_006570.2_cds_YP_169111.1_26 [gene=nuoE] [protein=NADH dehydrogenase I subunit E] [protein_id=YP_169111.1] [location=33957..34445]
ATGTCTTTAGTAGATTTAATATCGCCACAAGCGAGAGAAGATATTGACAGGGTTTTAAGTAAGTTTCCAG
CTGATCAACGAAGATCGGCGATATTGGAGGGCTTGCATATTTTGCAAGATCAAAATGGTGGCTACTTAAC
CGATGATTTGCAAACAGCTTTAGCAGAGTACTTACAGGTTAGTAAAGTTGATGTTTATGAGGTTGCTACA
TTTTATTGTATGTATAACCTAAAACCGGTTGGAAGACATAAATTGAATGTATGTACAAATGTTTCATGCA
TGTTAAATGGTGCTTATGAAATTTTAGCGCATATAGAGAAAAAACTTGCTATTAAGCCTGGCGAGACCAC
TAAAGATGGTCGTATAACTCTAAAAGAAGTTGAGTGCCAAGGTGCGTGCTGTGGTTCGCCTATGCTTGAG
GTCGATAAGGTTTTTTATGAAAATCTAACTATAGAAAAAGTGAATCAAATAATTGATTCTTTGGAGTAG

>lcl|NC_006570.2_cds_YP_169112.1_27 [gene=nuoF] [protein=NADH dehydrogenase I subunit F] [protein_id=YP_169112.1] [location=34452..35726]
ATGGCAAATGAAGTTTGTTTTCGTACTCTACATTTAGATAAGCCATACAGTTTGGAGTCTTATTTATCTG
TGGGAGGGTATTCCTACTGGAATAAAATATTGACAGAAAAAATTCCACCTGAGCAAATTATTGAAGAGCT
CAAAATATCTGGGCTTAGGGGTCGTGGTGGTGCTGGTTTCCCTACGGGATTGAAATGGAGTTTTATGCCA
CGTAATGCTCCGGGGCAGAAATACGTTGTATGCAACTCTGATGAGGGTGAGCCTGGTACATGTAAGGATA
GAGAAATTCTTAGGAATAATCCTCATCAATTGATTGAAGGTATAGCTATTGCTGGATATGTTGTTGGGGC
TACTGCAGGCTATAACTATACTAGAGGCGAGTTCTATGAGCCAATAGCTAGGTTTGAAGACGCGCTTATT
GAGGCATATCAAGCAGGTTTATTGGGTACAAATATCAAATCATCAGGAATCTCTTTTGATTTATACTCAG
CTATTGGTGCTGGTGCTTATATCTGTGGTGAAGAAACAGCATTGCTTAACTCACTTGAGGGTAAAAAAGG
TCGCCCAAGATTTAAGCCACCATTTCCTGCATTTAAAGGTCTTTATGACAAGCCAACAAATATAAATAAT
ACAGAAACATATGCTTCAGTGCCGGCAATATTACAATATGGCGGTGAGTGGTTTAAGAAATTGGGTACTG
AGAAAAGTGGCGGTACTAAGTTATTCTGTGTCTCTGGACATGTACAGCAGCAAAGAGTTGTTGAAATAGG
TTTAGGTATGCCGTTTAAAGAGCTTTTAGACATGTGCGGAGGCGTTCGCAATGGTAATAAGCTTAAGGCA
GTAATCCCTGGTGGCAGCTCATCTAAGATCTTGACAGCTGATGAGATGATGGCTGTTACTATGGATTATG
AGTCAATAGCAGCGGCAGGTTCAATGCTTGGTTCAGGAGCAGTTGTAGTGTTGGATGAGACTACATGTAT
TGTTAGAACTCTAGCAAGGCTTGCTGATTTCTATTATGAAGAGTCTTGTGGTCAATGTACGCCTTGTAGA
GAGGGTACTGGGTGGTTAGCACGAACTTTACATAGAATTGTTGCTGGTGAAGGAAGACCGGAGGATATTG
ATACTTTAGTTAGAGTAGCAACAAATATAGAGGGTAATACTATTTGTGGTTTGGGTGATGCAGCTGCATG
GCCAGTCCAGAGTTATATAGATAAATTTAGGCATGAGTTTGTTTATATGATCGAGAATAATGGTAGAAGT

ATAGTGGATCAATAG

>lcl|NC_006570.2_cds_YP_169113.1_28 [gene=nuoG] [protein=NADH dehydrogenase subunit G] [protein_id=YP_169113.1] [location=35744..38110]
GTGTCAGACAATAAAGAATCCAAAAAGATAAGTATAGAGATTGATGGTAAGGATTATCAAGCCCTACCAA
ACCAATCGATAATTGAAGTGGCAGATGCAAACGGTATATATATTCCGAGATTTTGCTACCATAAAAAATT
ATCTGTAGCGGCAAACTGTAGAATGTGTTTGGTAGATGTTGAGGGTGCAAGACGCGCTTCACCAGCATGT
GCAACACCAGTTATGGATGGAATGAAAGTTAAAACTCGTTCAGAAGAAGCATTGCAAATGCAAAAAGATG
TTATGGAGTTTTTGCTTATAAATCACCCATTAGATTGTCCTATCTGTGACCAAGGTGGCGAGTGTGAGCT
ACAAGATATTGCGATGGGTTATGGTAATACAACGTCAGAGTATCTTGAGTCTAAGAGAACGGTAGAAGAT
CCTGAGTTGGGGCCTTTAGTTGCTACTGATATGACAAGATGTATTTTGTGTACACGATGTGTACGCTTTG
GTGAAGAGATCGCTGGTGTCAAAGAGCTAGGGGTTATGGGTAGAGGCGATCACTCTGCTATTAGTACTTA
CATTTCCGGCGAAATGGTTGATTCTGAAATCTCTGCTAATATTATAGATCTTTGTCCTGTTGGCGCTTTG
ACATCAAAACCATTTAGATTTAAAGCTAGATCATGGGAATTAAAACAGTTTCCAACGCTTTCAGCTGGCG
ATGCTTTAGCAACTGAGATCAATGCACATATTTATCAAAATAAACTCGTCAGAGTAGTGCCAAGAGAAAA
TGATATTACAGGTACTTGGATAGCTGATAGAGATAGGTTTGAATATACTGGTTTGTATAGCCAAGATAGA
ATTCAACAACCAATGATTAAAAAAACTGGAGACTGGGTTGATTTATCTTGGGAAGAGGCATTAGACTTTG
TTAAAGTAGCTATACAAAAAACTATAGAAAAAGATGGTGCTGATGCAATCTCTGCTATTGTGTCTGAGAC
TGCAACTTCAGAAGAAATGTACTTAACTAAGAAGCTCTTAGCAGCTGTGGGTTCAGTTAACATTGATGCT
CGCGTTAGACAATATGCTGATATTCCGGGCATTAGTGCAGGCAAGGGCTTAAGTTGTTCATTAGAAGATA
TTCGTGAAAGTGACTTTATCCTTGTTTTTGGTTCTAATATTAGAAAAGAATATCCACTAGTAAATATTGC
GATCAAAGACGCTGTTGAAAAGAATGCTGCAAAGGCTGTAGCTTGGAACGTTTGTGATTATAACTTCAAT
TATGATATCAAACAGGTAAGATTAGCTGCTGATAACATTCAATATATTGCACTGTCTTTACTTAAGGCTA
TTTTTGTTAGAGCAAATTTACCTTATGGCGATCTTGATGAAATCCTAAGGAAAGTTGATCCAGCTGCAGA
GGTTAGAGATGTTGCTGATAGAATAGTCGCAGCAAAAGCTCCAAAGATAATTATTGGTCAAGATATTGTT
AATACAAGTGGTTTTGAAACAGTTTTTAGTATTTTGGATGTATTAGAAAAAGTTACTAGTGTAAGAGGTG
GTGTCTTAGCAACTAATGTTAACTCTGTTGCTGCTGATAGAATATTTAGTTCTTCTAAATCTAAGTTTAG
CACGTATAAGTGCTTAAATGGTCGAACAAATACTAAGCTACTTTTGACAGTACATACTGAGTTGGCTAAA
GATAGCTTGTACGGTGAGCAAAAGCTTAGAAATGCTTTAGAAAATATTGATATTGTAGTCAGTTTTACAG
CATTTGCTGATAAGTTTACCAAAGATACTGCAGATATTATTCTACCAATAGCAACTCACTATGAAACTAG
TGGAAGCTTCGTTGATTTATTTGGCAATAGAAAAGAGTTTAAGCAAGTAGTCAAACCATATGCTGGTAAC
AAGGAGTTATGGAGAGTCCTAAGAGTACTAGGTAATCTGTTGGAGCTAGAAGGCTTTGATTATAACTCTA
TAGCAGAGGTTACTAATGATGCTTATGCTACACGAGCTAGAGTAGGTGTTAATCATGTTACGCAGATTCT
CAATGCTAATCTTGATTATCAAAAAGAAGTAGCATTTGTGGCTTCAAACTCAATGTATAGTACAACTAGC
TTGCTTAGAAGAGCTGAACCTTTGCAGAAAACTACTGATGCAAAACGTTTTGCTGGTGTGAGAATATCGC
AAGAACTAGCAGATGAGATAGGTTTAAAAGGTCAGTCAGGAGTTATCAAAATTTATAATATCGACAATGA
AATAAATCCTGAAGTAGTTGTTGATCCAAGCTTACAGGCTAAAAATATTATGCTACCAAGAGCACTATTT
AAGGACTTTTTGAGTAGTGATAACATTAGTATTAAGCTGGTAGAGGAGGAGAGATAA

>lcl|NC_006570.2_cds_YP_169114.1_29 [gene=nuoH] [protein=NADH dehydrogenase I subunit H] [protein_id=YP_169114.1] [location=38112..39122]
ATGCTAGGGTATATTTTATGGACATCACTTTATGTATTGTTGATAGTCATTCCTTTGATACTAGTTGTTG
CATACTATACATATGCAGAGCGTAAAGTTATCGGTTATATGCAAGATAGAATCGGTCCAAATAGGGTAGG
ATCTTTTGGTTTATTGCAACCTATATTTGATGCGCTTAAGCTTTTTCTCAAAGAGATTATAGTGCCAACA
AATTCAAATAGATATTTATTCTTTATTGCACCGATTTTAGCTTTTGCTCCGGCATATGCTGCTTGGGCGG
TGATTCCTTTCTCTAAAGGTGTTGTTTTATCTGATATGAATCTCGGCTTACTATATATATTGGCAATGAC
TTCATTTTCTATATATGGGATAGTAATTGCTGGTTGGGCATCAAATAGTAAATACTCATTATTTGGCGCA
TTAAGAGCTGGAGCTCAGGTTATTTCCTATGAGTTGGCGATGGGTTTTGCTATCGTTGGAGTTGTCATAG
CAGCAGGCTCTATGGGTATAACTGGAATTATTGAAGCTCAAAGCGGTGGTATTTGGCACTGGTATTTTAT
TCCGCTATTTCCATTATTTATTGTGTATTTTATTGCTGGTATAGCAGAGACAAATAGAGCTCCTTTTGAT
GTTGTTGAGGGTGAGTCTGAGATAGTTGCTGGACATCATATAGAATATACAGGGTCTAGGTTTGCTTTGT
TTTTCTTGGCTGAATATGCAAATATGATTTTAATTAGTATCCTAACATCAATAATGTTTTTAGGCGGTTG
GAATTCGCCATTCCAAGCTACGGCATTAGAGTCTATATTTGGTTTTGTACCAGGTGTGGTATGGTTATTT
GCTAAGACTGGTATATTCATGTTTATGTTCTTATGGGTCAGAGCTACATACCCTCGTTATAGATATGACC
AAATTATGCGTTTAGGTTGGAAGATATTTATACCACTAACGTTTGTATGGGTTGTTATTGTTGCTTGCAT
GGTTAGACTTGGTGTAGGACCTTGGTGGTAA

>lcl|NC_006570.2_cds_YP_169115.1_30 [gene=nuoI] [protein=NADH dehydrogenase subunit I] [protein_id=YP_169115.1] [location=39136..39624]
ATGAGAAATATAACAAATTTTTTAAAGACTTTTCTACTTTGGGAGCTTCTAAAAGGGTTAAAAGTAACCG
GTAAGCATTTTTTTACTCGTAAGGTTACAGTACAGTATCCAGATGAAAAAACTCCTATTTCTAATAGATT
TAGAGGTCTACATGCCTTAAGACGCTATGAGAATGGCGAAGAGAGATGTATTGCATGTAAATTATGTGAG
GTTGTTTGTCCAGCATTGGCAATTACAATCAACTCCACAGAAAGAGAAGATGGTACCAGAAGAACTTCAA
GCTATGAGATGGATTTATTTAAGTGTATCTTTTGTGGCTATTGTGAAGAGTCGTGTCCAGTTGATTCTAT

TGTTGAGACAAATATCTTAGAATATCACTTCGAAGAGCGTGGTGAGAATATTATGACAAAAGCTAAACTT
CTTGCTATAGGTGATAAGTATGAAGCACAAATAGCTGCTGATAGATTGCAAGATAAGGACTTTAGGTAG

>lcl|NC_006570.2_cds_YP_169116.1_31 [gene=nuoJ] [protein=NADH dehydrogenase I subunit J] [protein_id=YP_169116.1] [location=39629..40234]
ATGGTTGTAACAGATATTTTATTTTATACATTTGCGTCACTTGCTATCATTTTTGCTTTAGTACTGGTTT
TAGCAAATAATCCAGTAAATTCTGTAATAGCAATGATTTTTACATTCATATTTACAGCTGCAGTATGGAT
AATCTTACAACAAGTATATTTAGCATTATTACTTATAGTGGTTTATGTTGGTGCTGTGTTAGTGATGTTC
TTATTTGTAGTCTTTATGCTAGATTTGCATGTTGAGGAACAGGGTAGAGTCGGTAGATTTTTCTATGCTC
TAGCTGCTGTGGTTGTCTGTGCTATATTTGCTACAGTTATAAGTTACGCTGCAACAAATGTTTTTGCCGG
AGCTATGATGCAAGGCGGAGTCGGTGGTCTTAAGATCATCGGTCTAACTATGTTTAGTAATGCTAATTTG
TATGTATTTGAGCTTGTTGATTTTATTTTATTAGCAGCTATGACTGCAGCGATAACATTAACATTAAGAG
CTAAGCGAAAAGGAAACAAAACTGTTGATCCAGCTCAGCAAGTTAAAGTCAGAGCAAAAGATCGTCTAAC
CATGGTGAAAATGCCAAGTAATAATGAGGGCGCTAAAGATGAATAG

>lcl|NC_006570.2_cds_YP_169117.1_32 [gene=nuoK] [protein=NADH dehydrogenase I subunit K] [protein_id=YP_169117.1] [location=40212..40544]
ATGAGGGCGCTAAAGATGAATAGTATTTCAGTCTCAGTCACACACGGGCTGATTTTTAGTACACTTCTGT
TTGTGATAAGTGTTGCTGGTATAATTATAAATAGAAGAAATATTCTTATATTATTGATGTCGATAGAGTT
AATGCTTCTAGCAGTCAATACTAATTTTTTGATATTTGCTAATATGCATCAGCAGGCAATGGGTGGAGTT
TTTGTATTCTTTATAATGGCAGTAGCTGCTGCTGAGACAGCAATTGGTTTAGCAATTGTTGTGGCAATAT
TTAGAAAACGCAAAACTATTGATTTAAGTAAACTTAATACACTAAGAGGTTAA

>lcl|NC_006570.2_cds_YP_169118.1_33 [gene=nuoL] [protein=NADH dehydrogenase I subunit L] [protein_id=YP_169118.1] [location=40554..42563]
ATGATAATAAACACTCAAGTAGCAGCTGTATTAATCGCAGTTATAGTTCTAGCACCTCTATTAGGTGCAT
TGATAGCTGGGTTTGGTGGTAAATCGGTAAAAAATGCAGGTGTTAACTTTTTCACAATTTCTCTATGTGG
TCTGTCTTTTGTCCTTAGTGTGATTTTAGCTTATGGTGTTTTTAGTGGCGCTGAAGTCTATTCTGTTAGC
TTTTATCAGTGGGCTCCTATTTCAAATATGTTTGCCTTTGATGTTGGCTTTACTGTAAATAAGATAACTG
TGTATATGATGTTGATAGTAACATTTGTATCAACACTAGTTCATATTTACTCGATTGGGTATATGAAAGG
CGAAGAAGGATATGCAAGGTTTTTTGCATATATTTCAGGTTTTACATTTGCAATGCTTTGTTTGGTAATG
GGAAATAATTTCTTACTATTATTCTTTGGTTGGGAAGGCGTAGGCTTATTTTCATACTTACTAATTGGTT
TTTATTTCAATAGAGATAAGGCAAATGTTGCAAGTTTAAGAGCTTTTATTGTAAATAGAATTGGTGACTT
GGGCTTCTTGTTGGGAATTGGAGCAGTTATTTTATATACAAAATCGGTTGACTATACGACAGTTTTTGCA
GCTTTACCCGATATTGATAATACTCAAACTATACACTTTCTAGGTATTAGTTTTAGTCCAGTTACGCTAA
TGTGTTCATTATTGTTTATAGGAGCAATGGGTAAGTCAGCTCAGTTTCCTTTACATTCATGGTTAGAAGG
ATCAATGGAGGGTCCGACACCGATTTCAGCACTAATCCATGCAGCAACAATGGTTACAGCTGGGGTATTT
ATGGTTGCGAGACTTTCTCCAATGTTTGTATTGTCACCAGCGGCCCTAAGCTTTGTATTAATTATTGGCG
CTATAACATGCTTGTTTATGGGGTTGATTGCAATCGTTCAAACAGATATTAAAAGAGTCATAGCATACTG
TACGCTATCGCAATTAGGCTATATGATGGTAGCTCAAGGCGCTGGTGCGTTTTCAATAGGTATGTTCCAT
TTGATGACACACGCAATGTTTAAGGCATTATTATTCTTGGCAGCTGGATCTGTTATTGTTGCGATGCATC
ATGAGCAAGATATTCGTAGAATGGGCGGTTTAAGGAAATATATGCCGGTTACATATTTGTGTATGTTAAT
AGGTGCTTGGGCGCTTGCGGCTTTACCACCTTTCTCAGGATTTTTCTCAAAAGACTTGATTATTGAAGCA
GCTCAAGCTACTACAGTATATGGACATGAGTTTGCTTACTATATGGTATTAGCGTGTGCGTTTGTTACAT
CGTTTTATATCTTTAGAATGTTTTTCTTGGTATTCCATGGTAAAGAAAGAATGTCAGATGAAGAAAGATC
ACATCTTAAAGAATCTCCATTTAGTATATTAATACCTTTAATATTATTGGCTATACCTTCTGTATTTGTT
GGGGAGTATTTCTTTAGTAGTATTTTGTCACAGGAGCATGGTTTATTCGGTAATACTATAACCCCATTTA
TACAAGCTGGCCTTGGTTGGGAGTCGGTAACAGCACACTTAGCTAATGAGCCAGCTATAGTAAATTCAAT
GGCATTCATTAAACATTCTGTAACAACGCTACCATTCTGGTTAGCTTTTAGTGCTTTAGTATTGGCATAT
GTTTTATATGTGTGGCTACCAGCAATTCCAAGACTATTTGCAAATGCAAAATCAGGATTTGGAATAATCT
ATCATATTTTAGTTAAGAAGTATTTTATTGATGCTTTGTATGATGTGATATTTGTTAATATCTTCTTGGC
AATTAGTAATTTCTTGTGGAAAGTTGTTGATATCTTCATTATCGATAAAACAGTTGTAAATGGTACATCT
AATCTTATATATCATACTGGCGATAGTTTTAGAAAGATCCAAAGAGGATATTTGTTTGACTATGCATTTG
TGATGATGGTTGGTGTATTATTGTTTATGATTTTGCTAATTTCTGTTTAG

>lcl|NC_006570.2_cds_YP_169119.1_34 [gene=nuoM] [protein=NADH dehydrogenase I subunit M] [protein_id=YP_169119.1] [location=42589..44178]
ATGAATTTAGGTAATTACTTATTAAGTTTAATAATCTGGCTCCCAATAGTTGGTGGTTTTGTTGTTTTGG
CGACAAGAACCAAAGAGTTGCATGGCGATGCAGCACGTTGGGTAGCATTAGTCTTTAGTTGTTTAACTCT
AGCTTTATGTGTTCCGTTAGTTACATCTTTTGATTATAGTAGTTCAGCAATGCAGTTCCAAGAATCAGTA
AAATGGTTTAAGGTTTTTGGCATGCATGATATTTACTATAGTTTGGGAGTAGATGGATTTTCTGTATTAT
TTATAGTTTTGACATCTTTTGCTACATTAGTGATAGTTTTAGCTGCTTGGACATCTATCAAAACTAAAGT
TAGACAATACATGGCGATATTTTTGATTACATGTGGTTTGACTAATGGCGTTTTCTGTGCGACTGATTCG
ATACTTTATTATGTTTTCTGGGAGGCGCTATTAATTCCAACTTGCCTTGGTATTGGTATTTGGGGTGGTA

AGCACAAAGCGTATGCTGCAGTTAAATACTTTATGTATACATTCTTTGGTTCGGTATTTTTACTAGCAGC
TATTTTGTATATTCAAACACGAGTTGCTGCTTCACCGACACACTTTTTGGTAAATCAAGATACTTATTCT
ATTCAGAATTTTATCGCTTGGGCAACTCAGTCACAAGGCTTTATAGATTCTGTTAAATTTACTCTAACAG
CACAATGGTTAGTTTTTGGGGCATTTTTCTTAGCATTTGCTGTTAAGATTCCGATGTGGCCATTTCACTC
ATGGTTACCAGATGCTCACTCAGAGGCGCCTGCTGGTGGTTCTGTCATCCTTGCAGCCCTTATGCTTAAG
CTAGGTGCTTATGGTTTCTTAAGATTTGCTATTCCAATGCTGCCAGAGGTAACAGCATCTCTCGAGTATG
TTTTGATTATAATGTCTTTGATAGCTATAGTTTATGTTGGGGTTGTTGCTGTTGCACAAACTGATGTTAA
AAGACTAATTGCATATTCATCAATTTCTCATATGGGTCTAGTTACTCTTGGTTTATTCTCAATATTTATA
TTGAAAAATGCTGATCCAGTATTAGGGACAACTCATGCGCAATTAGCTTTACAAGGCGCAGTGTTTCAGA
TGATTGCTCATGCTTTTTCATCTGGTGGTATGTTTATTGGTATTGGTTATTTATATTTGAGAATGCATAC
TAGAGAAATATCAGATTTCTCTGGTGTAGCTAAAACTATGCCAATATTTGCAACATTCTTTTTGCTTTTC
TGTATGGCGAATGTTGGTTTACCTGGCACAAGTGGATTTGTTGGTGAGTTTATGATCTTACTGGCAGTAT
TTCAATACTCTCCATTAATAGCATTAATTGCAGGATTAACTCTTGTGATCGCTCCAATTTATACTCTTTG
GATGTATAAGCGAGTTTTCTTCGGTGAGGTTGTATCTACACAGGTAGCAAGCTTGACTGATTTAAATAGG
ATGGAGCTATTTGTATTTATATTATTAGCGGTACCGACTTTATTGTTTGGTTTCTATCCAGAGCCAATTT
TGCAGCTATCAGCAGCAGCATCGGCACATATTGTTGGTCTATCTCTATAA

>lcl|NC_006570.2_cds_YP_169120.1_35 [gene=nuoN] [protein=NADH dehydrogenase I subunit N] [protein_id=YP_169120.1] [location=44188..45645]
TTGATTATGAGTTTATCAATCCTTTATATTTTGCCAGAAATACTACTAGCACTAGGTGTCATAGTTGTAA
TGTTTTCTGGACTTTTTCTACATGGTAAGATTAGAAACATTAACTATATTTTTTTCCAAGTATTTACACT
ACTTGCTTTGATAGCAACTTTTGCTAAAGAGTATCTGATACAGACTACTGGCTCAGCGTTTGAGGGTCAG
GTTGTATTTAGTGGCTTTGCATATACATTACAACTAGTAATACTTGTCTTAGCAGTCTTTGTTGCATTAT
ATTCAAGAGATTATGTCAAAGATAGAAAAATATCAGATGGTGATTTCTATACATTGTTAATGCTATGCGT
ACTTGGTGCAATGGTTTTAACAGCGGCACATAGTTTAGTTACAATATATGTTGGTCTAGAGTTATTATCA
TTACCAATGTATGCATTGATCGCAATCTATAGAGATTCAGGCAAAGGTCTTGAGGCCGCGATCAAATACT
TTGTTTTAGGGGCTATTGCATCGGCTCTACTATTATTTGGGATGTCATTTGTCTATGGAATGACAGGCAA
GCTTGATATTACAGAAATAGCCAATGTATTAGCACATGGTAACTTCGCAGGCTTACAGCAGCAGTTTTTG
CTTGTATGCTTGGTAATGATGATCGCTACATTTTTATTCAAACTTGGTGCTTTCCCTTTTCATATGTGGT
TACCAGATGTTTATCAAGGTGCTCCAAACGCAGTAGCAAATATCGTTGCAACTATTCCAAAAGTTGCTGC
CTTTGCGATGCTTGTGAATATCTTATTTGTTGGCTTCCCATCACTAAAAGATTCATGGATATATCTGTTT
AGGATTATAGGTATATTGTCAATATTTTTTGGTAGCTTGGTGGCACTCTCACAGACTAATGTTAAACGAC
TTTTGGGTTACTCAACAGTTTCACAGATTGGTTTTGTGCTATTGGCAACAACATTAAATCCTCAAGGATA
TGCCTTAACTGCGGCAAGCTTCTATGTCATAGTGTATGTATTTACAACTTTAGCTGTATTTGGCGTACTT
ACAACTATTTCTGTCGGGGGATATGAAGTTCAGGATCTTAATGACTTAAAAGGTTTCAACACAAAAGATT
CATGGTTAGCATTTATCTTGTTGATTGTGCTATTTTCAATGGCGGGTATCCCTCCATTTGGTGGCTTTAT
TGCTAAACTATTTGTAGTTATGGGATTAATTAATGATGGTAATTACTTCTTGGCGTGTTTTGTACTATTT
ATGGCAGTAATAGCATCATTCTACTATGTGCGTGTCATCAAAACCATGTACTTTGATGATCCTGATAATG
ATGAAACTGTTAAGCCACCGTTGACATCTCTAATAGCATTAAGTATTAACGGTTTGGTACTATTATTCTT
AGGGATTATGCCAATGCTCCTTTTGGGAGTTCTTACCCAAGTTACTAATGTTATATAA

>lcl|NC_006570.2_cds_YP_169121.1_36 [gene=FTT_0045] [protein=hypothetical protein] [protein_id=YP_169121.1] [location=45721..45915]
ATGAAGGAAATTCTAGGACCGCTTAATGATGTAATCAAGAATTCAAAAGAAAATATTGTTAATAAGAGTT
TGAAAAAGCTTGATGTTGTAAGTAGAGAAGAGTTCGAAGTACAGAAGAAAATTCTATTAAAAACTCGTCA
AAAGCTTGAGCAGGTAGAAGCTAAGCTTGATAAGCTTTTAGCTGAGAAAAAATAA

>lcl|NC_006570.2_cds_YP_169122.1_37 [gene=hemE] [protein=uroporphyrinogen decarboxylase] [protein_id=YP_169122.1] [location=47462..48496]
ATGAGAAAATTATTTTTGGATGCATTTGGCGAAAAGAAATTAGATAAGCCCCCAGTTTGGATAATGCGTC
AAGCTGGTAGATATCTACCAGAGTATCGTGCAGTTAGGGCAAAGTTTGATAATTTTATGGATATGTGCCG
CAATGCTGATGCTTGTTGTGAAGTTGCGCTTCATCCACTTCAGAGGTATGATCTTGACGCAGCAATTGTT
TTTTCAGATATTCTGACAATTCCAGAAGCTATGGGGATGGACCTAAAATTTATAAAAGGTACGGGGCCAG
TCTTTTCTGAGCCGATACAATCGCAAAAAGATTTAGATAAGCTAAAATCTATTGAAGATAGTATTGGTTC
GCTAGATTATGTTTATAATGCTGTCAAGACTACTAGTTCAGCAATAAATGTACCTTTAATAGGCTTTACT
GGAAGTCCTTGGACTTTAGCGGCATATATGATTGAAGGTTCAGGATCTAAGCAGTTCAATAAATTGCGTA
AGATGATGTATGCAAATCCTCAGTTGATGCATAGCTTATTACAACGCCTTGCAGATATAACAATTATCTA
TTTGCTTGAGCAGGTTAAGGCTGGCGCGAGTTCTGTGATGATATTTGATACTTGGGGAGGTATACTACCT
TTAGAACATTATAAAAATTTCTCTCTTAAATATATGGAGTATATTGCTAAGAATGTAAAGCAAAAAATTA
ATATACCTATAGTTTTCTTTACTAAGGGTGGATCAAACTTTTTTGAAGAGATAAAAGATAAATCCTGTGA
TGGAGTAGGAGTTGATTGGAGTGTTACATTAAAACAGGCACGACACAGAATAGGGGTTGGGAAAGTTCTG
CAAGGGAACTTTGATCCCGCATTTTTATATGGGTCTAAGCAAAGTATTAGGGAGACTGTTAGAGCCAATA
TTGAATTTATCCAGTCAGATAAGCTAAATAATTATATTGTCAACTTAGGTCATGGAATTTATCCTGATAT
AGATCCTGATAGTGTTAGAGTTATGATAGATGCGATTAGAGAATTTAGTGCTTAA

>lcl|NC_006570.2_cds_YP_169123.1_38 [gene=FTT_0048] [protein=hypothetical protein] [protein_id=YP_169123.1] [location=48716..49174]
GTGAAAATGTTGCTAGACGATTTATATGAGATAGTAGAACCCATCACAGCTGATCTAGGCTATATTTTAT
GGGGAATTGAAGTTGTAGGAAGTGGTAAACTTACCATACGTATTTTTATTGATCATGAGAATGGTGTTTC
AGTTGATGATTGCCAAATAGTTAGTAAAGAAATCAGTGCAGTTTTTGATGTTGAGGATCCAGTTTCAGGT
AAATATATTCTTGAAGTTTCTTCACCTGGGATGAATCGCCAAATTTTTAATATAATTCAGGCGCAAGCTC
TGGTAGGATTTAATGTAAAGGCGGTTACTTTGGCTCCAGTTGGATCACAAACTAAGTTTAAGGGCGTGCT
AGAAAGAGTAGAAGGAAACAATGTCATTCTAAACTTGGAAGATGGTAAAGAAATAAGTTTTGATTTTGAC
GAGCTTAAAAAACTAAGGGTATCACCTGATTTTAGTTAG

>lcl|NC_006570.2_cds_YP_169124.1_39 [gene=nusA] [protein=transcription elongation factor NusA] [protein_id=YP_169124.1] [location=49192..50661]
ATGAGCAAAGAATTATTGTTAGTATTAGAAACTGTAGCAAATGAAAAAGATATCTCAAAAGATCTTTTAT
TTGAAGCTATGGAAGAGGCTTTGGCTATTATAACTAAGAAAGAACTTGATGAGCATATGAATATCGAGGT
TAAAATCGATAGAGTTACGGGTGATTTCAAGGCTGATAGAGTTTGGCATATTGTTTCTGAAGATGAAGAT
TTAATTGATTATTCAAAAGAGCTTTATGAAGATGTCGTTCAAGAAAAAGGCTATAATGTAAAAGCTGGCG
ATGTTATGCGTGAACCTGTTGAGGTCAAAGAATATGGGCGTATAGCTGCGACAATGGCTAAGCAAATTCT
AATGAAGAAAATCAAAAATTTTGAAAGAGAAAAGTCAGCTAGATTTTATCAAAATAAAATTGGCGATATT
GTTTACGGCGAGATAAAGCGCGCTACATATGAAATCTTAGTTGTTGATTTGGGCAATAATGCTGAGGGGA
TTTTACCTAAAAAAGATCTAATAGCTAGAGAAAGATATCGAGTTGGTGACAAAATAAGAGCATGTGTTGA
AGCTGTTGAGAGTGATGAGTCTGGCAAGCCAAATACGATTATGTTAAGTCGTTCAAGCAATACTATGCTT
AAAGCTTTATTTAAGCTTGAGGTTCCGGAGGTTGAAGAAGAGCTTATTAATATAGTGAATGTAGTTCGTG
AACCTGGTTTTAGGTCTAAAGTAACTGTTAAAAGTAATGATCAAAGAATAGATCCTTGTGGTGCCTGTGT
TGGTGTGAGAGGTTCTAGAATACATTCTATAATGAGTGAGCTAAATGGTGAGAAGGTTGATGTTATACTT
TGGAATGAAGATATGGTTCAGTATGCAATTAACTCATTATCACCAGTTGATGCAGCAGACATCTTAGAAG
TGAATGTTGATGAAGAAACTAATTCGATAGATATTGTTGTTAAGCAAGAAAGTTTATCTAAAGCAATTGG
TAAAAATGGTGTTAATGTAAGATTAGCAAGTTCGTTAATAGGTTGGAAAATTAATGTTCTATCAGATGCT
GAGCAGGAAGAAAAACAAATGTCTATAGTAGAGAAATTTGTTGAAGCCTTAGATATAGATCACGATTTTG
CATTAGTATTGATTGAAGAGGGTATAGAGACTCTTGAAGATTTAGCATATTTAGATAGAGCTGAGCTTTT
AGAAATTGAAGGGTTTGATGAAGAGATCGTTGATGAGCTTCAAGAGAGAGCAAAAGCAGTTATTTTATCT
CAAGCTTTAGGAGGTAAAAAACCAGCTCAAGATTTGTTAGATATGCAAGGTATGAGTCTGGATTTGGCTG
AGCAGCTAGCGCAAAATAATATAGTTACTATGGAAGATCTAGCAGAGCTATCTGTAGATGAGTTGCTTGA
TATTGTGGATATAGATGAAGAGCAAGCAACTAATTTAATTATGCAAGCAAGAGCTCCTTGGTTTGAATAA

>lcl|NC_006570.2_cds_YP_169125.1_40 [gene=infB] [protein=translation initiation factor IF-2] [protein_id=YP_169125.1] [location=50700..53240]
ATGGCAGAGATTACAGTTGGGCAGTTAGCACAGCAAACAAATAAAGAAGTAGATGCACTACTAAAACAGC
TTAAATCTTTTGGTATTGAAAAATCTAGTGAGAAAGATACATTAACTCCTACGGAGATGAAAACTTTGCT
AGAGAAGATTAATAGTGCGAAAAATACCGCAACAAGGAAGAAAGTAACTTCAGTGAAACTTGACGGCAAA
CATAAAATTAATGTTTCTGTCAAAAGAAAAAGGCGAGTTGCTAAGAAAGTGGAACAGCAAGAGTCTACTA
CGTTAGAACAGCCACAAGAGCTTGAAACTATGGTTCAAGAAGTCTCTCAGCAAGTAGATATCGTAAAAGA
ACAGGATAATATCGAGCAAATTGTTGAGAATAAAGAGGCTGTGAAGGTTCAAGAACAGCGACAAGCTGAA
ATAGCAAAACCAGTTATAAAAGATAGTGGTTTTAAGATAACGGCAATGCCAGAGATTAAGATTGAGGAAA
TCGTTGCTGAAGATGATGAAGGGTTGGCTGCTAGTGATAAGCAAGCTAAGAAAAAAGCTGCTAAGAAAGT
ATTCTCTGAAGCTGTGAATACTAATACAAAGTATAAGCGTGAGGAAGAAGAGAAAAAAATCTAAAGCAAAG
AAAGCTGGAGGTAAAGGTTTTAAAAAAGCTAACCCAAGGCAGCTTTCACAACTTGCTGGTGATCTGGAGT
CATTTGATGAGTTTGGAGCTAAAAAAGGTAAACTAAAAGCGCCAAAAGTTAAAAAGCAGGAATTTACAAA
ACCTGTTGAAAATACAGTTAGAACTGTTGAAATTCACGAAGGAATTACTGTAAGTGAACTAGCTCAAAAA
ATGGCTGTCAAAGGTGCTGAAATAGTTAAAGTACTATTTAATATGGGTGTGATGGCGACAATTAACCAAT
CGTTAGATCAAGATACAGCAATCTTAATCGTTGAAGAGATGGGTCATAAATATACACTACATAACGAGAA
TGCTCTTGAAGAGGCGGTAACAATAGTTGATAGAAGCTCATACAAGAAGATTTCTCGTGCTCCAGTTGTA
ACTATAATGGGACACGTTGATCATGGTAAAACATCATTGCTAGACTATATTCGTCAAACACGAGTGGTTG
CTGGTGAAGCTGGTGGTATAACACAGCATATTGGCGCGTATTCTGTTAAGACTGATAAAGGTTCAATCAC
ATTCTTAGATACTCCAGGGCATGAGGCTTTTACTTCAATGCGAGCACGTGGTGCTAAGAGTACAGATATT
GTAATTTTAGTTGTTGCTGCTGATGATGGTGTTATGCCGCAAACTGAAGAGGCAATTCAGCACGCTAAAG
CAGCAAGGGTACCAATTGTTGTTGCAGTAAATAAAATTGATAAGCCAGAGGCTGATCCTGATAAAGTGAT
TAGTGAACTTGCACAAAGAAACGTAATCCCAGAATCATGGGGTGGTGATGTAATGTTTGTAAATGTTTCT
GCTAAAACTGGTGAAGGTGTTGCTGATCTATTAGAAGCAGTACTTTTACAATCAGAGGTTTTAGAATTAG
AGGCATTTGCTGAGGGCTTGGCTGAGGGTGTCGTAATTGAATCACGTCTAGAAAAGGGTCGTGGTCCAGT
GGCAACTGTACTTGTACAAAATGGTAACCTTAAGCAGGGTGATAATATCTTGTGTGGTACTGAGTACGGT
AGGGTAAGAGCTATGCATAATGATCTTGGGAAAAAGATAAAAGCAGCTGGTCCAGCTACACCTGTTGAAA
TACTTGGTTTATCGGGTATGCCAGCAGCTGGTGATGAGATGGTTGTGATTGAAAATGAGAAGAAAGCAAA
AGAGCTTGCAGCACAGCGATCTCAGAAACAAAAAGAAGCTAAAATTGCCCAAGAACAATCACTTAAGCTA

TCAAATATGTTCAATAACATGGGTAAAGAAGGTGAACAGCAAGTACTTAAAATTATCCTTAAGGGCGATG
TACAAGGTTCTGTTGAGGCAATTAGAGAGTCACTACTTAAGCTTTCAACAGATGAAGTTAAGGTTGATAT
AATTGCAAGTGGCATAGGAGCGATTACATCTTCAGATGTGACTTTAGCTGTAGCATCAACAGCGGTTGTT
ATAGGTTTTAACGTCCGTGCTGATAGTGCTGCTAAAAAGCTTGCAGAAACTGATGGTGTAGAGTTTCGTT
ATTACAATATTATTTATGATTTAATAGATGATGTTAAAAAAGCGATGTCTGGTTTACTTTCTCCTGAAAT
GAAAGAGCAGATTATTGGTATTGCTGAGGTTAGAGAAGTTTATCGTTCATCTAAATTCGGTTCAATTGCT
GGATGTATGGTTATTGAGGGAGTAGTTAAGCGTACTAATCCTATCCGTGTCTTACGAAATAATGTTGTTA
TTTACGAAGGTACACTAGAGTCACTTAAGAGATTCAAAGATGATGCTAGTGAAGTTAAAAAAGGCTTAGA
GTGTGGTATTGGTGTCAAAAACTATAATGATGTCCGTGAGGGTGACCAAATAGAGGTATTTGAGGTTATT
GAGGTTGCTAAGGAGTTGTAA

>lcl|NC_006570.2_cds_YP_169126.1_41 [gene=rbfA] [protein=ribosome-binding factor A] [protein_id=YP_169126.1] [location=53243..53674]
ATGGCTGCAGAGGGTAGAGTACAAAGAGTAGCGAGTGAGTTTCAAAAAGTTATATCTTTATTACTGCGTA
CTAGAATAAAAGATGCTAAGCTAGCTAGTGCAACTATTACAGAAGTGGATCTTTCTAAGGATCTATCTTA
TGCAAAAATATATTATACTTGCTTAGCTATTGAGGATGCTGAATATATTGACAAAGCATTTGAAAAATCA
AAAGGTTTCTTTAGATCATCTATAGCTAAGTCATTAAGTCTACGAATTGTTCCAAATCTTAAGTTTATAT
ATGACACTTCGCTTGATTATGGTATGCAGATGGAAGAAAAAATCCAGCAGGCACTTGAAGCTGACTCAAA
AATTATCAAGCAAGATGATAAATCTTTACAAGAAAATTATAAGCAAAACGATAAAGAAACTAAAGCTGAG
AAGTTAAGGTAG

>lcl|NC_006570.2_cds_YP_169127.1_42 [gene=hisS] [protein=histidyl-tRNA synthetase] [protein_id=YP_169127.1] [location=53682..54947]
ATGAGTAAGCTTACTATTGTTCGTGGATTTAATGATGTATTGCCACTAGACAGTTATAAATGGCAACTCT
TGGAATCAAAAGTAAAACTCATACTTGATAGATATAACTACAGTGAAACAAGATTGCCTATTGTTGAGAG
GAGCGAATTGTTTCATAGAAGTGTAGGTGAAAGTTCTGATATAGTTTCAAAAGAGACTTATGACTTTCAA
GATCGTAATGGTGATAGCCTGACATTGCGTCCAGAAGGAACAGCTGGCTGTGTTAGGATGGTTATTGAAA
ATAATTTGGCTACTCGAGGCCAAACACAGAAACTATGGTATTGTGGACCTATGTTTCGCTATGAGCGCCC
ACAAAAAGGCCGCTATAGACAATTTTATCAGCTTGGGGTTGAGGCTTATGGCTTTGATGGTATAGCTATT
GATTTAGAGGTTATTGCTATAGCTTGGAGTCTTTTCAAAGAGCTTGGGATTTATGAGTATGTAACTTTAG
AACTAAATAGCCTTGGTTCTAGTCTTAACAGACAAGAGTATACTCAAGCGCTATTACAATACCTAAAACC
ATATCATGCTGAGCTTGATGAAGATTCTATCAAGAGGTTGGATAAAAATCCTCTAAGAATATTAGATTCT
AAGATAGAAAAAACACAAAAAATCTTAGCAAACGCACCTAAACTTATTGATTTTATAGATCATGATTTGC
GTTTGAGATTTAAACAAACTTGTCAATATTTAGATGCCTTGGGTGTCAGATATAAATTAAATGAAAATCT
TGTTAGAGGTTTGGATTATTATACTGGTTTAGTTTTTGAATGGACTACTGATAAACTTGGTTCACAGAGT
GCAATCTGTGCAGGTGGTCGTTATGATGGTCTTGTAGAGAATCTTGGTGGTCAGAAAACTGCTGCGATAG
GATTTGCTATAGGTATGGAGCGTTTGTTACTCTTATTAGAAGATTTAGGTAAATTACCTAATCAAGATAA
TGCTTGTGATGTGTTTTTCATATTAGATAGTGCCCAGCTGCATCAATCTCTTGCTATAGTAGAGAATATT
CGCCAAGAGCTTCCCCAACTTAAAATAGATATGGATCTAAAGTTTGGTAGTTTTAAATCACAATTTAAGA
AAGCGGATAAATCTGGAGCTAAAGTTGCTATAATAATCGGCCAAGATGAGTTAGATAATGGCTTTGCTGG
TATAAAATTTCTTCAACAAAATGAAGAACAACAACAAGTTGCTTTTAATGAGTTAATAAATTTTTTAGAA
AGATAG

>lcl|NC_006570.2_cds_YP_169132.1_47 [gene=FTT_0057] [protein=hypothetical protein] [protein_id=YP_169132.1] [location=59627..60058]
TTGCTAATATGGGCTTTTTTGGTATTATGTGAGAGTGTTTTTATGAATGAGATTCATGTAATGTTAACAA
ATATTAAAATTGATACCAAAAAATTTATCTTACTGCAATTGATCTTAGTATTAGTTGGTTACTTTGTTAT
TTTAGTATTTTATAATTATGATTATGCTAATTCATTTTTAATAGGTTCATTAACAATGTTTTTAGCTAAT
TTTGTGTTTTTTTTCAGATTGTTTATAAATAAGCAGTTCAGTCCAGGGATTGAAATAGCAATATTTTATC
TCAGCGAGCTTTTGAAACTAAGCATTGTAGCACTGCTGACAATATTGTTAGCAATTTATGTAAAACCGAA
ATTATTTTCTTACATTTTTGGTTTAGTTTTGTTACAATTAGCAGTGTGTTTTGTGCCTATCTTGTTCAAA
AGGGTCAGATAG

>lcl|NC_006570.2_cds_YP_169133.1_48 [gene=atpB] [protein=ATP synthase F0F1 subunit A] [protein_id=YP_169133.1] [location=60111..60902]
ATGGCAAATACAGAAGCAGGCTCTCAAGTAGCTACTGAATATGTGCAGCACCACTTGCATCACTGGCAAG
TAAGTTTAGGTCAAGGTTCTTTTTGGCAGCTTAATGTTGATTCATTATTGGTGAGTGGCATATTAGGGGT
TGTATTTATATTGGCTATGTTTTTGGCAGCGAGAAAAGCTAACTCAGGAGTTCCAGGCAAGTTTCAGAAT
ATGATTGAGGCTGTTTGGGAATGGATGGATGGCATGGTTGCTAATAATTACCATCATAAAAGAGATTTTG
TTACACCACTAGCACTTACTATATTTGTTTGGGTAGTGTTGATGAATTTTATGGATTTGCTCCCAGTTGA
TCTTTTTCGGTTGGATAATTAGCTTCTTTACTGCTAGTCATGAAGCTTATTTTAGAGTTGTTCCTACTGCT
GATCCTAATGTTACTTTTGCTATGTCAATAGCAGTTTTCTTCCTTGTGATTTTTTATAATCTCAAGGCAA
AAGGCTTTGGGTTGATCAAAGAAGTGCTAAGCTCACCTTTTGGTATTTGGTTATTTCCTTTAAATATCTT
CTTTAGACTGGTTGATGAAATAGTTAAGCCAGTTTCACTGTCGCTGCGTTTGTTTGGTAATATTTTTGCG

GGAGAACTTATATTTATCCTTATAGCTTTATTGCCGTGGTGGTTTCAGTGGACTCTTGGTGGGATATGGG
CAATATTCCATATCCTAATTGTTTTGATACAGGCTTTTGTATTTATGATGCTAACTGTAGTTTATTTAAA
TATGGCGCAGGAAGCTCACTAA

>lcl|NC_006570.2_cds_YP_169134.1_49 [gene=atpE] [protein=ATP synthase F0F1 subunit C] [protein_id=YP_169134.1] [location=60953..61258]
ATGGATATGTCTTTACAAGTTTTAGGGAACTTAAATGGTTTGACAGCAGTTGCAGTAGCTTTGCTTATAT
CTTTACCAGCTTTAGGAACAGCAATAGGTTTTGGTGTTTTAGGTGGTAAATACCTTGAAGGCGTTGCGCG
TCAGCCTGAGTTAGGTGGAATGTTACTTGGTCGTATGTTTATCGTTGCGGCTTTCGTTGATGCTTTCGCT
GCTATCTCAATAGCAATTGGTTTCTTAGTTCTTTATGCAAACCCTTTAGCAATTCCAGGCTTAGCTGAAG
CAGCTCAGAAAGTAATCGGCGCATAA

>lcl|NC_006570.2_cds_YP_169135.1_50 [gene=atpF] [protein=ATP synthase F0F1 subunit B] [protein_id=YP_169135.1] [location=61304..61774]
ATGGATATTAATATAACTCTAATAGGGCAAATGATAACGTTTGCAATCTTTGTTGGATTTACAATGAAGT
TTGTATGGCCTCCGCTACGTAAAGCTTTAGAAGAGCGTAGAGAAAAGATTGCTGAAGGTCTAGCATCTGC
TGATAGAGCATCTAGAGAACTAGAGGTTGCTAAGAGACAATCTGCAGAGATTTTGCGTGAGGCTAAGGCT
AAGGCGACCGAAATAGTTGAAAATGCTTATGTAAGAGCTCATAAGGTTGATGAGCAAGCAAAAGAGGAAG
CTATTGCTGCTGCTGATAAGATTAAAAGCATGGCCATTGCTGAAATAGAGCAAGAAAAGGTTAAAGCGAA
AGAGCAACTTAAGCAAGAGCTTGTTAATTTAGCTATGGCGGCAGCTAGTAAAATCATAGCAGCAAGTGTT
GATGAAAAAGCTAGTAAAAAAGTTTTAGAAGACTTTGTAGAAAAAGTATAA

>lcl|NC_006570.2_cds_YP_169136.1_51 [gene=atpH] [protein=ATP synthase F0F1 subunit delta] [protein_id=YP_169136.1] [location=61794..62318]
ATGACTAATATAAGTGTGATTGCGAAGCCGTATGCTAAAGCAGCATTTGAGTTTGCAAATGAACATAACC
TACTACAACAATGGTCTAAATTGCTTCAAACATTTTCTGAGTTGATCAAAGATAAATCGGTAGCTGCAAT
TGTATCTAGTCCTACTATATCTCAAATTGAGGTTGTGGATGCTCTAAAAAAACAGTTAGACGAAAACTTT
TTTAATTTTCTTGCTTTGATAGCTGAAAATAAAAAAATGTTGATAATGCCAGAAATAGCAGATCAGTTCG
AGTCTATAAAAAATATCCATAACAACGTTAGAGTTGCTGATGTTACTCTAGCTTACGCTACAGATAAAAA
TATCTTAGATAGTCTGAAAACTAGTTTGGAGAAAAAGTTTGGCTGTACAATTGATATGCATATAAATATA
GATCCGGCGATAATTGGTGGAGCAGTAGTCAAGGTTGGGGATACAGTTATTGATAGTTCAGTATCTGGCC
ATTTAGAGAAATTAAAAAGTATTTTGTTATCATAA

>lcl|NC_006570.2_cds_YP_169137.1_52 [gene=atpA] [protein=ATP synthase F0F1 subunit alpha] [protein_id=YP_169137.1] [location=62337..63878]
ATGCAATTAAGTCCATCAGAAATAAGTGGTTTAATAAAACAAAGAATCGAGAAGTTTGACAATTCAGTTG
AACTAAAATCAGAAGGTACAATCGTCAGTGTTGCTGATGGTATCGTTACTATTTATGGATTAAATGATGT
TGCTGCTGGTGAAATGATTAAATTACCAGGCGATGTTTATGGTCTAGCTCTGAACTTAAATACAGATTCG
GTTGGTGCTGTAGTTTTAGGGGATTATGAGCATATTAAAGAAGGCGATAAAGCATACTGTACTGGTAGAA
TACTAGAAGTCCCAGTTGGAGAGGCTCTCTTAGGCAGAGTTGTTGATGCTTTAGGTAATCCTATCGATGG
TAAGGGTGAAGTGGCAACTGATTTAACTTCACCTATCGAAAAAATTGCTCCAGGTGTTATTTGGAGAAAA
TCAGTAGATCAAGCGCTACAAACAGGTATTAAGTCAATTGACTCAATGGTACCAATTGGTAGAGGTCAAA
GAGAGCTTATCATTGGAGATAGACAGATAGGTAAAACCGCAATTGCTGTTGATACGATTATTAACCAAAA
GGGTACAGGTGTTAAGTGTATCTATGTTGCTATTGGTCAGAAGGCATCTACAATTGCAAACATTGTAAGA
CAGCTTGAAGAGCATGGCGCAATGGAACATGCAATTATTGTTGCAGCTACAGCATCTGATTCTGCAGCGC
TGCAATATATCGCGCCTTATGCGGGATGTTCTATGGGTGAATACTTTAGGGATCGCGGTCAAGATGCACT
TATTGTTTATGATGATTTGACTAAACAAGCATGGGCTTATAGACAGATTTCACTTTTATTAAGAAGACCG
CCTGGACGTGAAGCTTATCCTGGTGATGTATTCTATCTTCACTCTAGACTTCTTGAAAGAGCTGCAAGAG
TCAACGAAGAATATGTCGAGAAATTTACAAATGGTGAAGTGAAAGGTAAGACAGGTTCTTTGACTGCACT
ACCTATCATTGAAACACAAGCTGGTGATATCTCTGCATTCGTTCCAACGAACGTTATATCAATCACTGAC
GGGCAGATATTCTTAGAGACAGATTTATTTAACTCTGGCTTAAGACCTGCGATAAACCCTGGTAACTCTG
TTTCACGTGTCGGTGGTGCAGCACAAACTAAGATTATCAAGAAACTTGGTGGTGGTATCCGTTTGGCACT
TGCGCAATATAGGGAGCTAGAGGCGTTCTCACAATTTGCATCAGACTTGGATGAAGCAACTCGTGCACAA
TTAAACAGAGGTCAAAGAGTAACAGAATTGCTAAAACAAAAGCAATTCTCAACTTTATCTGTGGCTTTAA
TGGCATTGTCACTTTATGCTGCTGATAATGGATACTTAGATAACTTAGAGGTTTCAGAGGTTATACCTTT
TGAATCTGCGCTACATGCATTAGCAGAAACAAAGTATAGTGATGTAATTGCAGAAATTAATGAAACTGGT
AAGTATGATGCTGATATTGCTGATAAGTTAAAAATTATTGTTGAAGACTGTAAAGCAAATCAAGCTTGGT
AA

>lcl|NC_006570.2_cds_YP_169138.1_53 [gene=atpG] [protein=ATP synthase F0F1 subunit gamma] [protein_id=YP_169138.1] [location=63893..64789]
ATGTCTAACGCTAGAGAAATACGTTCCAAAGTACAGAGTGTTAAAAACACGCAAAAAATCACAGGTGCAA
TGGAATTAGTTGCTGCTAGTAAAATGAGAGGCGCGATTGTTAAGATGAATAATGTGCGTCCATATGTTGA
AAGCGCAAATACTATTATCAAAAATGTCACAGCTGCAAGTATTGATTACCCTAATCCTTATTTATTCGAC

AGAGATGTAAAAAGAGTTGGCTATATAGTCATATCTACGGATAGAGGCCTTTGTGGTGGTCTTAATATTA
ATCTATTTAAACATGTTCTAAAAGAAATCAAGAACAATATTGAAGATAGAGTTGGGGTTGATGTATGTGT
GATTGGCTCAAAAGCTGAAAATTTCTTTGCAAAACTAAAAGATGTTAATATTGTAGCAACAGCTCATTAT
AATGATAAAGACAAGGAAGGAAGCATTAGAGCTATTGGTGGCGCTGTTAAAGTAATGCTGGATAAGTTCA
CTGCAGGTGAGATTGATAGGTTATATATGAGTAGTAACCAATTTGTTAGTACTATTAAACAAAGACCTAG
GTTGCAAACATTACTACCAATTCAAGATATTTTTTCAGCAGAAGAGATAAAAGCAAATAAAGAAAAAGCA
ACTAAAGGGCATTGGGACTATATCTATGAAAGAGATATAGAGGAAGTTTTAAATGCTCTATTTATCAGAT
ATATCGAGGCTCAAGTCAGAGGCGCTATATTAGAAAATGCAGCATGTGAGCAAGCTGCTCGTATGATGGC
GATGAAAAATGCAACTGATAACGCTAGTGATATAATTGATCAGTTGAAATTAGATTACAACAAAGTAAGA
CAGGCTATGATTACGCAAGAACTTGCAGAAATTTGTTCAGGTGCGGCAGCAGTTTAG

>lcl|NC_006570.2_cds_YP_169139.1_54 [gene=atpD] [protein=ATP synthase F0F1 subunit beta] [protein_id=YP_169139.1] [location=64799..66175]
ATGAGTACAGGTAAAATTATTCAAGTAATTGGTGCTGTTATTGATGTAGAGTTTGCTCGAGATAACACGC
CTAAAGTATATGATGCTTTAAATGTAGTAGAAGCTGGTTTAGTATTAGAAGTTCAGCAACAAATTGGTGA
TGGCGTAGTTCGTACAATTGCTATGGGATCTAGTGATGGTCTTAGACGTGGTATGGAAGTTAAGAACACA
AATGCGCCTATTTCTGTTCCAGTTGGACATGGCACACTTGGACGTATCATGAATGTTTTAGGTGAACCAA
TTGATGAAGCTGGTCCAATTGAATATACTGAGAAAAGATCTATCCATCAAGCTCCTCCTGCATATGATGA
GTTAGCATTAAGTACAGAAATATTAGAAACAGGTATCAAAGTAGTTGACCTTATTTGTCCATTTGCTAAG
GGCGGTAAAGTTGGTTTATTTGGCGGTGCAGGTGTTGGTAAAACTGTAACGATGATGGAACTTATCAACA
ATATTGCAAAAGAACATAGTGGCTACTCTGTATTTGCCGGTGTTGGTGAAAGAACTCGTGAAGGTAATGA
CTTCTACTATGAGATGAAAGATTCTAATGTATTGGATAAAGTATCATTAGTATATGGTCAGATGAATGAG
CCGCCTGGAAACAGATTAAGAGTAGCTCTTAGTGGCTTAACAATAGCAGAAGGATTCCGTGATGAAAAGC
GTGATGTTTTGATGTTTATCGATAACATCTATCGTTATACATTAGCAGGTACAGAGGTATCGGCGCTACT
TGGTCGTATGCCATCTGCTGTGGGTTATCAGCCAACGCTTGCAGCTGAGATGGGTGCTTTACAGGAGCGT
ATTACATCTACTAAGACAGGATCTATTACTTCTGTCCAGGCTGTATATGTACCGGCAGATGACTTAACAG
ATCCTTCACCAGCTACAACTTTCTCACACTTAGATGCAACGATTGTACTATCACGTCAAATTGCTGAGTT
AGGTATTTATCCTGCGGTTGATCCTCTAGATTCAACTTCTAGACAGTTAGATCCTTTAGTTGTAGGTCAG
GACCACTATGAAACAGCTCGTGCAGTGCAGAAAGTACTTCAAAGATACAAAGAGTTAAAAGATATTATCG
CTATTCTTGGTATGGATGAGTTATCTGATGAAGATAAGAAAATTGTAGATAGAGCTCGTAAGATTCAGAG
ATTCTTATCACAGCCATTCCATGTTGCAGAGGTGTTTACTGGTAACCCTGGTAAGTTCGTATCACTTAAG
GATACTGTAGCAAGCTTCAAAGCTATAGTTAACGGTGAATATGATCATTTACCAGAGCAAGCTTTCTATA
TGGTTGGTTCTATACAAGAAGCAATTGAGAAAGCAAAAACTCTATAA

>lcl|NC_006570.2_cds_YP_169140.1_55 [gene=atpC] [protein=ATP synthase F0F1 subunit epsilon] [protein_id=YP_169140.1] [location=66188..66625]
ATGACAAAAAAAATATCTAAAAGTTGATGTCGTTAGTCCTCTAGGTTCAGTTTTCAAGGGTGAAGCTGATA
TGGTGAGTCTGCGCGGCTCTGCTGGTGAGATGGGCATAGCATACGGTCATACTGAGCTATTATCTACTTT
ACCGGCTGGTGTAGTAAATGTCAGAAAAGATCAGCATACTGATGTGCTATATGTTTCAGGCGGTATAGTA
GAGGTTACTCCAACTCGTGTAACTATTATGGTTGATGATATGGAGAGAGCTGAAAACCTTAATCAAGCTG
AAGCAGAGAAAGCAAGAGCAAGAGCAAAAGAAGTTCTAAAAAATCCAGATGCTTCAAAATTAGATATTGA
AGCGGCAAATAAAAGACTAAAAGAAGCTGATGCTCGTCTTAAAGCTCTTAATTCTTCAAATGGGTTATAT
TATTCAAAGGACGATTAA

>lcl|NC_006570.2_cds_YP_169143.1_58 [gene=sodB] [protein=superoxide dismutase] [protein_id=YP_169143.1] [location=70161..70739]
ATGAAATTTGAATTACCAAAACTACCTTACGCTGTTGATGCATTAGAGTCAACAATATCAAAAGAAACAA
TAGAGTATCACTATGGTAAACATCATCAAACATATGTAACTAATCTAAATAATTTAGTTGAGGGTACAGA
GCACGATGGCAGAAACCTAGAAGAAATCGTAAAAACTTCTAATGGCGGAATATTTAATAACGCTGCTCAA
GTTTTTAATCATACTTTTTACTGGAATTGTTTAACTCCAAACAAAACAGAAGCTTCAAGTCAGTTAAAAG
CAGCATTGATCGAGACATTTGGTTCTGTAGAAAATTTTAAAGAACAATTCTCTAAGGCAGCTATTGCAAC
ATTTGGTTCTGGTTGGGCTTGGTTAGTAAAAAATACTGAAGGTAAACTTGAAATAGTAACTACAAGTAAC
GCTGGTTGCCCATTAACAGAGAACAAAAAGCCATTGCTAACTTTTGATGTTTGGGAGCACGCATACTATA
TTGATTATCGTAATGCTAGACCTAAATATGTTGAAGCATTATGGGATATCGTAAACTGGCAATTTGTTTC
TGAGCAATTCGCTGATTAG

>lcl|NC_006570.2_cds_YP_169145.1_60 [gene=ampG] [protein=major facilitator superfamily transporter] [protein_id=YP_169145.1] [location=complement(70944..72209)]
ATGACTAGTTCTCCATCATTTATAGATAAACTCAAAGCCCCTTTCAAAGAAGCTAAAATGTTTACTATGT
TAATATTAGGCTATGCATCAGGATTTCCTCTTATGCTTACAGCCTCATCACTATTCTTATGGTATAAAGA
TAACGGCATAGAAACCAAAGATATTGGTTTTTTGACCCTTATAGCAATCCCATACACTTTTAAGTATTTA
TGGGCTCCTTTCTTAGATAAGATAAAAAATTCCCATATTAGGGCGCAGAAAAGGCTGGATACTAATAACTC
AAGTATCTTTAGTCATTCTAATTGCCATTATGAGTCGATTCTCACCAGCGAACTCACCACTTATAATTGC

TTTTATTGGCTTTCTAATCTGTTTTATATCAGCAACGCAAGATATTGCTATAAATGCTTACCAAACAGAA
GTTCTCCTAGAGCACGAAAGAGCTCTAGGGAATGCTATAGCAGTCATGGGTTATCGTATTGGTATGCTAG
TGACAGGATCATTGGTATTAATTATTGTAGATAAGCTTAACAACAACTGGAATCTTGCCTGGCTTATGAT
TATACTATTTTTTATCATATGCCCACTCTATACTTTGTTAATCAAGGAGAGTCAGTATCAAGATGCTCCT
AAAAGCTTTAAAGATGCTTTTATACTCCCCTTTGTTGAGTTTTTTAAGCGGCAGGGATTATATACGGCAA
TAATTATAATAATAATTCTTATAAGCTATAAACTAGCTGATGCAATAGCATTTTCACTGAATTCAATCTT
TTTTATTGATCTTGGATTTAGTAAAACTACTATTGCGGTTTCATACAAAGCATTTTCACTATTTGCATCG
TTAGCAGGCTTATTTGTTGGAGGTCTAATTGCAAAACAAATTGGAGTTTATAAAAGCTTCTTGTACTTTA
GCATTATTATGGCATGTGCAAATTTAACTTATGTATTATTAGCAATAGTCGGTAAAAACTACTATTTGAT
GCTAGCCTCTGTAAGTGTAGAATATTTTTGCGGCGCAATGGGTACAGCAATGTTAGTAGCAATGATCATG
AGCCTTGTTAATGTAAAATTTTCAGCTACTCAATTCGCAATTCTAAGTTCAATAGACTCTTTAGCAAGAG
TATTTGTAGGACCTTTAGCTGGTTATATTCAAGCACACTATAAATGGGAAGGATTATTTATCTTCAGTTT
TATAGTTGGAATGTTTATATCACTACTAATATTTATTTTTAGAACTAGGATAAAATTAATGGCGAATTTA
CAATAA

>lcl|NC_006570.2_cds_YP_169146.1_61 [gene=gltA] [protein=citrate synthase] [protein_id=YP_169146.1] [location=complement(72298..73572)]
TTGGAGGTCATGTTAATGAGTAAATACGCAACTCTTAAGTATGCAGATAAAAATATTGAAATAGAATTAC
CAGTATATTCTCCTAGCTTGGGTAATGACTGCATAGATGTTTCATCATTAGTAAAACACGGAATTTTTAC
TTATGATCCTGGATTCATGTCTACAGCTGCATGCGAATCTAAAATTACATATATCGATGGTGGTAAAGGT
GTACTCTTACATAGAGGCTACCCAATCGAAGAATGGACTCAAAAATCAAACTACAGAACTCTTTGCTATG
CATTGATTTATGGCGAACTCCCAACAGATGAACAGGTTAAAAGCTTTAGACAAGAAATTATCAATAAAAT
GCCAGTATGCGAGCATGTCAAAGCTGCAATAGCTGCAATGCCTCAACATACACATCCTATGTCAAGCTTA
ATAGCTGGTGTTAATGTCTTAGCTGCTGAACATATCCACAATGGACAAAAAGAATCTCAAGATGAAGTCG
CAAAAAATATCGTAGCAAAAATTGCAACTATAGCAGCCATGGCTTACAGACATAATCACGGCAAGAAATT
CTTAGAACCTAAAATGGAATATGGCTATGCTGAGAATTTCTTGTATATGATGTTTGCTGATGATGAGAGC
TACAAACCAGATGAGCTACATATCAAAGCAATGGATACAATATTTATGCTTCATGCTGATCACGAGCAAA
ATGCTTCAACATCAACTGTAAGGTTATCAGGATCAACTGGTAACTCTCCTTATGCGGCAATTATCGCAGG
TATTACCGCATTATGGGGACCTGCTCATGGAGGCGCTAATGAAGCTGTGCTTAAAATGTTATCTGAAATT
GGTAGCACTGAGAATATCGATAAGTATATTGCTAAAGCGAAAGATAAAGATGATCCATTTAGATTAATGG
GCTTTGGACATAGAGTCTACAAAAATACTGATCCGAGAGCTACTGCAATGAAAAAGAACTGTGAAGAAAT
ACTTGCTAAACTTGGTCATAGTGATAACCCTCTTCTCACAGTAGCTAAAAAACTAGAAGAAATTGCTTTA
CAAGATGAGTTCTTTATCGAGAGAAAGCTTTTCTCTAATGTTGATTTTTACTCAGGTATAATCTTAAAAG
CCATGGGCATTCCAGAAGATATGTTTACAGCCATATTTGCTCTAGCAAGAACATCAGGGTGGATATCTCA
ATGGATAGAGATGGTTAATGATCCTGCTCAAAAGATTGGTCGTCCAAGACAATTATATACTGGTGCAACA
AATAGAAATTTCTAA

>lcl|NC_006570.2_cds_YP_169147.1_62 [gene=sdhC] [protein=succinate dehydrogenase cytochrome b556] [protein_id=YP_169147.1] [location=73815..74288]
TTGAGTAAAAATATATCATTAAGAGTTGTTTGTATAACATTGGTTACATCTGATCTACTTCTCTACAAAA
ATTGCTTCGGAGGAATGTCGAGCATGAAAAAAATTACTAATATTGACTTGATGTCAATAAAATCATATAA
CTTCCCAATCACAGCTATTAGCTCTATTATGCACCGCATATCAGGAGTTGTGTTGATAATTGCGATACCG
CTTTGCGTGGTTGCGATGAACTATGCTCTTGCAGGGCCTGATGGTTACCAGCAAACAGTTACTGTATTAA
CTAAATCTTGGTTTAGTGTATTTTTTTGGCTTTTTTTATCATCGATAACATATCACGTTTACGCTGGTAT
TAGACATATGATTATGGATATGGGCTTTGGTGAGAGTATGAAAGTGGCAAAAATCACTTCACTACTAGTT
ATCGTTTTGGGTGTTTTATCAGCTATTTTATGGGGGTGTTACTTATGGCTGTAA

>lcl|NC_006570.2_cds_YP_169148.1_63 [gene=sdhD] [protein=succinate dehydrogenase hydrophobic membrane anchor protein] [protein_id=YP_169148.1] [location=74264..74632]
ATGGGGGTGTTACTTATGGCTGTAATTTCATTAACTTCTTCTGGAATTAAAGACTTTTTCGTACAAAGAG
TTACGGCTGTAATTATTGCTGTATATTTTGGTTATCTTATCCTAGAAGCTTTGTATTTGTCGCATATAGG
AGCTCTTAACTATGATAGTTGGAGAGGTTTGTTTACCGATGGGATGTTTTTTAGAGTTGCTACGTTAATG
GCGTACTTGGCGATGTTTTTTCATGCATGGGTAGGTATCTGGATTATATGTGGCGATTATATTAAATTTG
CTTGGGCTTCTGCGCTTGTGATGCTGAGCTTTGTTTTGGTGTATATATTCTGCTTTTTTTGGTTATTTGC
AGTTTTATTTTTCTATTAA

>lcl|NC_006570.2_cds_YP_169149.1_64 [gene=sdhA] [protein=succinate dehydrogenase catalytic and NAD/flavoprotein subunit] [protein_id=YP_169149.1] [location=74645..76438]
ATGAGTATAGCTACGCAAGAATTTGATGCTATTGTTATCGGTGCTGGTGGTGCGGGTTTAAGAGCTTCTT
TCCAGCTGTCACAGTCGGGTTTTAAAACTGCTGTTGTTTCTAAAGTCTTTCCAACAAGATCACATACGGT
TGCAGCTCAAGGTGGTATTGCAGCTGCTTTAGGTAATATTGAATTTGATGATGGCTTGCCATCAGATGAT
TGGAAGTGGCATATGTATGATACTGTTAAAGGTTCTGATTATATTGGTGATCAGGATGCTATTGAGTATA

TGTGTGAGCATGCGCCACAATCAATTATTGAGTTGGAGCATATGGGTATGCCTTTCTCAAGACTTAAAAA
TGGCAAGATATATCAACGAGCGTTTGGTGGGATGTCAAGGAACTATGATCCTGCTAATCAAGCAGAAAGA
ACTTGCGCAGCAGCTGATAGGACCGGGCATGCTCTTTTACATACACTGTATCAGGGTAACTTAGCGCACA
AAACTGATTTCTATACGGAGTGGTTTGCTGTTGATTTGGTTAAGGCGGATGATGGTAGTATTGCTGGGGT
TATAGCTCTTTGTATAGAAACTGGTGAAACTGTTTTCTTAAAAGCAAAGATTACTATATTAGCTACTGGT
GGCGCTGGGCGTATATACGAGTCAAGTACTAATGCTTATATCAATACTGGTGATGGTATGGGTCTTGCTT
TAAGAGCTGGATTACCTCTTCAAGATATGGAGTTTTGGCAGTTCCATCCTACAGGCATTGCAGGTGCTGG
AGTTCTTGTTACTGAAGGTTGTCGCGGTGAAGGTGGTGTGCTACGCAATAAAGATGGTGAAAGATTTATG
GAGCGATATGCTCCTAATGCTAAAGATCTTGCTTGTCGTGATGTTGTATCGCGTGCCTCTCAGCAAGAAA
TTATGGAAGGGCGTGGTGATACTTTCTCTGGGACAAGCTGTGTGTGGTTGGATTTGACTCACTTGGGTGA
AGACGTCATTAATGAGAGATTGCCTACTGTTAGAGAATTAGGTAGAACTTTTGCTGGGATTGATCCAGTT
GAAAAACCAATACCGGTTGTGCCTACTTGTCATTATCAAATGGGTGGGATACCAACTAATAAACATGGTC
AAGTAATTACTCAGGTTGATGGTAAAGATAAGGTTATAGGTGGATTGTACGCTGTTGGTGAATGCGCCTC
TGTGTCAGTTCATGGTGCAAACAGGCTTGGAAGTAATTCTTTATTAGATTTAGTTGTTTTTGGTAGAGCT
GCTGGTATGCATGCGGAACAAAGCCTAAAAGAAGGTATGCCTATGAAAGAAGTTTCTCAAGAGAATATTG
AGAAAGCTACTGCTAGAATCACTAAGTGGGATACTTCAGAGCAAAGAGGCTGTAAGGAAAAAATTTCTGA
ATTAAGAAAAGAATTACAGCGCGTAATGCAGCAGTACTTCTCTGTATTTAGGCAAGAAAGCACAATGAAA
GAAGGGCTTGATAAGTTATTTAATATTAGAGAAAGGTTAGATAATGCTGTTCTGGAAGATAACTCTAGAA
TTTTCAATATGATGAGAATAGAAGCGTTAGAGTTAGATAATCTAGTATTAACTGCGATAGCTACTGCAAA
ATTAGCACTTGAAAGAAAGGAATCAAGAGGCGCTCATTCAAGAGTAGACTATCCTGAAAGAGATGATAAG
AATTGGATGAAGCATACGCTATACTTCTTAGAAGGAGATAGAACATCTCTGCGTGATGTAAATATGTCTC
CTACTAAAGTAAAAGCTTTTCAACCAGCAGAACGTAAGTACTAA

>lcl|NC_006570.2_cds_YP_169150.1_65 [gene=sdhB] [protein=succinate dehydrogenase iron-sulfur subunit] [protein_id=YP_169150.1] [location=76456..77157]
ATGGAAGTAAGATTTAAAATTTATAGATATAATCCAGAAGTTGATAAAAAACCTTATTACGATGAGTATA
CGGTTGAAGTAGAAAATGAAGGTGTTAAGGTTCTAACAGCTCTAGAATTAATTAAAGAGCAGGATCCTAC
CTTGGCTTTAAGAAGATCTTGTCGTGAAGGTGTTTGTGGTTCTGATGGTATGAATATCAACGGCAAGAAT
CGTTTGGCGTGTATCACTTCGGTTGGTGAGTTAAAGCAGCCTATTAAAGTTAATCCTTTACCAGGGTTGC
CAGTTATTAGAGATTTAATCGTCGATATGAAACAGTTCTATAAGAATTATGAGAAAGTTAAACCATATCT
TATCAATGATGATGAGCCGCCTGTTAAAGAGAGGTTACAATCACCAGAGGATAGGGCTAAACTTGATGGG
CTGTATGAGTGTATTTTATGTGCTTGTTGTACTACATCGTGCCCTTCGTTTTGGTGGAATCCCGACAAAT
TTATAGGTCCTTCTGGATTGTTACAAGCTTATAGATTTATCGCCGACTCAAGAGATACTGCTACAGAGCA
AAGACTTGAAGATCTAAAAGATCCATTTAGTCTTTTCAGATGTAGGACTATTATGAACTGTGTTTCTGTG
TGTCCAAAAGGTCTTAACCCTACAGAAGCAATAGGTAAGATTAGATCAGCATTATTAAAGAAGAATGTAT
AA

>lcl|NC_006570.2_cds_YP_169151.1_66 [gene=sucA] [protein=2-oxoglutarate dehydrogenase subunit E1] [protein_id=YP_169151.1] [location=77175..80000]
TTGGGTGCCTATATGAAAAAAAAACAACCAGATTTTAGTCAGTGGCTGGAGACTACCCAGTTCTTTGGAG
GTAATCTAGAATACCTTGAGTCAATATATGACGATTATATAAGGGGCAACCATGATGGAATAGATCCTAA
GTGGCTGTCCTTTTTTGATTCTATAGCAAGCTCAACAGATACAGTTCATGGTGAACTGGTTGATGAGTTT
AAATACTTGGCTAAAAACAAAAATAATACAGCTAACGTAAACACAGTTGTAAGTACTGAAGGGGATATTG
GTTTAAAAGCTAAAGCTCTAGTTAAAGCTTACCGTTCTTATGGTTATAAATCAGCCAATATTGATCCACT
TGGACTGACAAGGTTTGAAAGAGATTCAGATTTGGAGTTGGCAGCACATGGATTATCTGAAAAAGATCTA
ACACAGTTGGTAAACCTCGGAGACTTTACTGATAATAAAGCAATTCCTTTGCAGCAGGTAATCAATAAAG
CTAAAGCAATTTATGAGTCTAATATAGGCTATGAGTATAGATATATAGGTAACAAAGAAGAAAAGCTTTG
GCTTCAAGATAGAATAGAAGATACTGCTGTTATTCCTAGTGACAGCAAAAAATGGATTTTACAGCAATTA
GTAGCTGCGGAAGGATTAGAGAAATACCTTGCGCTAAGATATGTGGGTCAAAAAAGATTTGGCTTGGAAG
GTGGTGAGTCATTAATCCCATCTTTACAGCATATAGTCGAGAAAGCTGTTTCTCGACATTCAACTCGCTT
TATTCAATTGGGTATGGCACATAGAGGCCGTCTAAATGTATTAGTTAATGTAATGGGTAAAAACCCTAAA
GATTTATTTGAAGAGTTTGAGGGCAAGCAAAGCGAAAAAAGCTTATCTGGTGATGTGAAGTACCATATGG
GTTACTCTAATTACAGAAGTATTGATGGTAAAGAAGCTAAGATTGCTTTAGCATTTAATCCTTCACATTT
AGAGGCAGTCGATCCAGTTGTTGAGGGTGCCGCTAAAGCAATTCAAGACAAATTAGATGGCGATGTTTAT
AGTAAGGTTTTACCAATATTGATACATGGTGATTCAGCTTTTTGTGGTCAAGGTGTGGTAATGGAGACCT
TTGGTTTCTCGCTTACAGAAGCCTATGGTACAGGTGGGACGATTCATCTTGTTGTGAACAACCAAGTTGG
CTTCACTACAAGTAGTGCTTTTGGCGTAAATAGAAGTAGCAATTATTCTACTGATGTTGCTAAAATGGTT
GACGCACCGATATTTCATGTAAATGGCGATGATCCAGAAGCTGTGCTTAAAGTTACTGATATTGCTTTAG
AATATCGTATGAAATTCAACAAAGACATTGTTATTGACTTAGTTTGTTACCGTAGAAATGGTCATAATGA
AACTGATGAGCCATCAGGAACACAGCCACAGATGTACGAAGTGATTAAGAAACTTCCTTCAACATTAAAG
CTATATAGCGACAAGCTGATAAAAGAAGGTGTGGTTGATGCTGATCACTTTGCACGTATGAACGCTAATT
ATCGTAGCAAACTAGATAATGGTAAGGTCACGATAGATGTTCTTGATAGAAAGATTATCAAAGATAAGTT
AAATGTTTGTGATTGGCTTCCTTATCTAGGTAAGCAAGAATCAGATTATAATTATATGCCTATACCAGAA
AAAACTCTCAAAGAGTTAGCACTGAAGATTAGTGAAGTGCCTGCTGAGGTGGAAATGCAAATGCAGGTCA

AAAAAGCTGTTACTGATAGAATCAAAATGGCTAATGGTGAACTTCCTCTAAACTGGGGATTTGCTGAATC
ACTTGCATATGCGACATTACTTAGTGATGGCTACCCAGTGAGAATTTCTGGAGAAGATAGTGGTCGAGGA
ACTTTCTCACATCGCCATGCGGTTATCAAAAATATGAATACTAAATCGCAGCCAAAAGAGTATGTACCTT
TAAGACATATTAATGAAAAAGTAAGATTTGATGTTATTGACTCTACTCTTTCAGAATATGGTGTATTAGG
TTTTGAGTATGGCTATAGCTGTTACAGTCCTGATGCTCTAGTTGTATGGGAAGCTCAATTTGGCGATTTT
GTTAATACAGCACAAGTTGTGATTGATCAGTTCCTTGTTGCAGCAGAAGAAAAATGGGGTATTTTATCAG
GTTTAACTTTATTTTTACCTCATGGCCAAGAAGGTGCTGGTGCAGAACATTCATCTGCTAGGTTAGAAAG
ATTTTTAAACTCTTGCGCTAATGATAATATGCAGGTATGTACACCTACAACACCAGCACAAATTTATCAT
CTACTAAGACGTCAAGTTATTCGACCGCTTAGGAAGCCTTTGATTGTAATGACACCGAAAAGTTTATTGA
GAAATCCTATGGCGGTATCTTCATTACAAGAGCTTTCTCAGGGTAAATTTGAGGCAATAATTGATGATGT
AAATGCTAAAGCCGCTAAAGTTACAAAGCTTATACTATGTAATGGTAAGGTATATTATGATCTTATGGCT
AAGAAACAAGATAATTATGAGCATATAGCTGTTGTAAGATTAGAAGAGTTGTATCCTTTCCCACAACAGC
AGCTTGCGCAAATATTTACTAAGTACAATAATGTAAATAAAGTGGTATGGTTACAAGAAGAACCTGAAAA
CAAAGGGGCTTGGTATAATATTAGGCATTTCATAGAAAAGTTAGTAGATAAAAAGCAAGAATTGTTATGT
GTGGCAAGAGAAAGATCATCTACACCTGCTGTTGGATATCATGCCTTATATGTAAAACAGCAGGAAGAAA
TTATTAATACAGCTTTAGAAATATAA

>lcl|NC_006570.2_cds_YP_169152.1_67 [gene=sucB] [protein=dihydrolipoamide succinyltransferase component of 2-oxoglutarate dehydrogenase complex] [protein_id=YP_169152.1] [location=80026..81495]
ATGGTTGAATTAAAAGTACCTATGTTCCCAGAGTCTGTAGCAGATGGCACATTAGCTCAATGGAATAAAA
ACGAAGGTGACTTTGTAAATGAGGGCGATATCTTGGCAGAGATTGAGACTGATAAAGTTGTTCTAGAAGT
ACCTGCAACATCTAGTGGTGTTTTAAAAGGGATAAAAAAACATGCTGGTGATACAGTGCTTTCAGAAGAG
TCATTAGCGATCATTGATACTGCTGTTTCTACATCTGAACCTAACCAACAAACTACTAATCAAGGAAATG
CTTCAGAAGCAACTGCTACTGGGCAAGAAATTGATATTAAGGCGCCTGTATTTCCAGAGTCTGTAGCAGA
TGGCACGATCTCAGAGTGGCATAAGAAAGAGGGTGAGGCTGTTTCTGAGGGTGATATCTTAGCAGAGATT
GAGACTGATAAGGTTGTTCTAGAGGTTCCGGCAACATCAAATGGTGTTTTGACAAAAATATTAAAAACAG
CAGGAGAGACTGTACTATCTGCAGAGCTTATCGCTAAGATTACAGCAGGAGGCGCAACTGCTACTACGAA
ATCAGAAGCTTCGGTGGGAGTTTCTCAAGCAAATAATGATCCGCATCTAGTACCTTCAGCACGTAAAGCT
TTTAATGCAAGCGGCTTGGATACTGCTGCTAATATCGAAGGTACAGGTAAAAAAGGGCGTATAACTTCTG
AAGATGTCAAAAAAGCAGTTGCATCAGTAAATAAACCTCAACAACAGACAGTTGTTATAAATCAAGGTGC
TAGATATGAAAAAAGAGTCAAGATGACTCGTCTGCGTCAGACTATAGCAAATAGGTTAGTTGAGGTTCAA
CATACTAATGCAATCTTAACTACTTTCAATGAAGTAGATATGAGTGCAGTTATGGAGCTTAGAAACAAAT
ATAAAGATATGTTTGTCAAAGAACATGATACTAAGCTTGGCTTTATGTCTTTCTTTATCAAAGCAGCAAC
AGAAGCACTTAAGAAATTCCCAGATGTAAATGCCTCTATTGATGGTGATGAGATTGTTTACCATAATTAT
TTTGATATTGGTATTGCTGTAGGTACTGATAGGGGTCTAGTGGTACCTGTACTAAGAGATACAGATACTA
AATCTCTAGCTGAATTAGAAGCCGATGTTTTAGACAAAGCGATTAAAGGTCGTGATGGTAAATTAAGCCT
TGAAGATATGCAAGGTGGTACATTTACGATTACAAATGGCGGAACTTATGGTTCGATGTTATCTACGCCT
ATTATTAATTCACCGCAAAGTGCTATTTTAGGTATGCATAATATTGTTGAGCGTCCTGTAGTTGTTAAGG
GTGAGATTAAGATTCGTCCAATTATGTATTTAGCGTTATCTTACGACCATAGAATCATTGATGGCGGTAC
ATCTGTAAGATTCTTGAAGATGATCAAAGAGCTAATTGAAGATCCAAATAGAATTCTTCTACAAGTATAG

>lcl|NC_006570.2_cds_YP_169154.1_69 [gene=glmM] [protein=phosphoglucosamine mutase] [protein_id=YP_169154.1] [location=82073..83404]
ATGGCAAAGTATTTTGGAACTGATGGTATCCGTGGTGAAGTCGCTAACTCAACAATAACAGTAGAGTTTA
CGCAAAAATTAGGTAATGCTGTTGGTTCGTTAATAAACCAAAAGAATTATCCAAAATTTGTTATTGTTGG
TCAAGATACACGTAGCTCAGGAGGGTTCTTAAAATTTGCTTTAGTTTCTGGCTTAAATGCTGCAGGAATT
GATGTGCTAGATTTAGGAGTAGTTCCAACTCCTGTGGTGGCATTTATGACTGTTAAGCATCGAGCTGCGG
CTGGATTTGTAATTACAGCATCACACAATAAATTTACTGATAATGGTATTAAATTATTTTCCTCTAATGG
TTTTAAGCTAGATGATGCTTTAGAAGAAGAAGTTGAGGATATGATTGATGGTGATTTTATCTATCAGCCA
CAATTTAAGTTTGGCAGCTATAAGATTTTAGCAAATGCTATAGACGAGTATATTGAGAGTATTTATAGTC
GTTTTGCTAAATTTGTTAATTATAAAGGTAAAGTTGTCGTTGATTGTGCTCATGGAGCAGCATCACATAA
TTTTGAGGCTTTACTTGATAAATTTGGAATAAATTATGTTTCTATAGCCTCTAATCCTGATGGTTTAAAT
ATAAATGTTGGGTGTGGTGCTACTTGTGTTTCAAATATTAAAAAAGCTGTTAAAGAGCAGAAAGCTGATT
TAGGTATTTCTCTGGATGGTGATGCAGATAGAATAATTATTGTCGATGAAAATGGTCAAGAAATTGATGG
CGATGGAATATTAAATATTCTTGCTCAGTATAGTGACATTTGTGGTGGTACTAATGGTATTGTTGGAACA
CAAATGACAAATATGAGTTATGAAAATCATTATAGGGCTAACAAAATCCCATTTATCCGTTCAAAAGTTG
GTGATAGATATGTCTTAGAAGATTTAGTCAAGTATGGCTATAAAATTGGTGGTGAATCATCTGGGCATGT
CATTAACCTAAACTTTGGAACTACTGGAGATGGTTTGTTTACAGCAATACAACTCTTAGCTATTTTCTCT
CAAGCGGATAAGCCTGTATCTGAATTTAAACTGCAGGGAGAGTTGATGCAACAAACATTAATCAATGTGC
CATTGACTAAGAAAGTGGCACGTGAAGATTTACAAAAAGTTGCTAGCGATGTTAATGATGTTGAGAAGCG
TTTAGGTAATAGAGGAAGAGTATTATTAAGACCATCGGGAACCGAACCTGTTTTAAGAGTTATGGTTGAA
GCAGATGACAAGAGTCTCGCTACAAACGAGGCTGAGTATTTGGTTGAAAAAGTAAAACAAAAATTGGTGT
AG

>lcl|NC_006570.2_cds_YP_169155.1_70 [gene=tpiA] [protein=triosephosphate isomerase] [protein_id=YP_169155.1] [location=83407..84168]
ATGCAAAAATTAATAATGGGTAACTGGAAAATGAATGGTAACTCTACAAGCATAAAAGAGCTCTGTAGTG
GTATATCACAAGTGCAATATGATACTTCAAGAGTAGCTATTGCTGTTTTTCCATCAAGTGTTTATGTTAA
AGAAGTAATCTCACAGCTGCCAGAGAAAGTAGGTGTTGGTCTACAAAATATTACTTTTTATGATGATGGT
GCTTATACTGGTGAGATATCTGCTAGGATGTTGGAAGATATTGGTTGTGACTACTTACTAATTGGTCATT
CTGAGAGAAGATCTCTATTTGCTGAGTCTGATGAAGATGTTTTTAAAAAGCTTAACAAGATTATAGATAC
TACTATAACGCCAGTAGTGTGTATTGGTGAATCACTAGATGATAGACAAAGTGGTAAGCTCAAACAAGTT
TTAGCAACACAACTAAGCTTAATCTTAGAAAATTTATCTGTTGAGCAGTTAGCAAAAGTCGTAATTGCAT
ATGAACCTGTCTGGGCAATAGGCACAGGAGTTGTGGCTTCACTAGAGCAGATTCAAGAAACACATCAATT
TATTCGTTCATTGTTAGCTAAAGTTGATGAAAGACTTGCTAAAAATATAAAAATAGTGTATGGTGGTAGC
CTAAAAGCTGAAAATGCTAAAGATATATTAAGCTTACCAGATGTCGACGGTGGTTTAATTGGTGGCGCAT
CTTTGAAGGCTGCTGAATTTAACGAAATAATAAATCAAGCAAACAAGATATGTACGGAATAA

>lcl|NC_006570.2_cds_YP_169156.1_71 [gene=secG] [protein=preprotein translocase subunit SecG] [protein_id=YP_169156.1] [location=84156..84509]
ATGTACGGAATAATTTTAACTATTGATATTATCGCAGCTATTGCGATTGTGGTACTAGTGTTGCTGCAGC
AAGGTAAGGGCGCTAATATGGGCGTTTCTTTTGGAGCAGGAGCATCGAGTACTGTTTTTGGGAGTAAAGG
AGCGGCATCATTCTTATTTAAGATGACAGTATTTTTTACCGCGGTATTTTTTGTGTCTTGTCTTACTTTA
GGGTACTTAGGCAAAAGTAGTGCGATAACAGCGAATATAAGTACTGCTAATACTGATAGCTCAATTGCTA
GTCAGTATGACCAGTACCAAAAAGAAGTTTCCCAAGCTGGTACAGCAGATACCTCTAAACAAGCTTCTAA
ATAA

>lcl|NC_006570.2_cds_YP_169161.1_76 [gene=acnA] [protein=aconitate hydratase] [protein_id=YP_169161.1] [location=88770..91583]
ATGTCTGATATTAAAAATATCACTAAGCTACAAATAGAGGATAAAGGAAAGAAATATTCTCTATATAGCT
TAAAGAAACTTTCTCAAGAGCTTGGTAAAGATGTTACGCGTCTTCCTTATTCAATCAGAGTATTACTTGA
AAACCAACTTAGAAATATAGATGGTTATAAAGTAAAAGAAGATGATATGCACAAGGTTTTAGATTGGGAC
GCTAAAGCTAGCTCAAGACCTGAGATCCCACATATGCCTGCTAGGGTTGTGATGCAAGATTTCACAGGTG
TACCAGCAGTTGTGGATTTAGCTGCGATGAGAAAGGCGATCAAAGATGCTGGGGGTGACGCAGACAAAAT
TAACCCTCTTGTAGATACAGCAATGGTAATTGACCACTCAGTACAGGTTGATTTTTATGGAACAAAAACT
GCTTTAGCTCAAAACGTAGCTAAAGAATTTGAAAGAAATGGTGAGAGATACAGCTTGCTTAAGTGGGCAC
AAAAAGCATTTGATGATTTTATTGTTGTACCACCTGGGATGGGTATTATCCACCAAGTTAATTTAGAGTA
CCTTGCAAAAGGCGCTTTAGTTAAGAATATTAATGGTGAAGATGTAATTTATCCAGATACTCTAGTAGGT
ACAGATTCACACACTACTATGATCAATGGTGTTGGTGTAGTTGGTTGGGGTGTCGGTGGTATTGAGGCTG
AAGCTGTAATGCTTGGTCAACCATACTATATGGTTTTACCTGATGTTGTTGGTGTGAAATTTACTGGTAA
GCTAAAAACTGGTGTAACAGCTACTGACCTTGTACTTAAAGTTACAGAAGTGCTTAGAAAGCATGGTGTT
GTTGGCAAGTTTGTTGAGTACTATGGCGAAGGTTTAGAGAGCTTATCTTTACCAGATAGAGCAACTATCG
CAAACATGGCTCCAGAATATGGTGCAACAATAGGTTTCTTCCCAGTTGATGAAGTAACATTAGACTTCTT
CAACAATACAAACCGTAGTGAGTTAGTTGATGCCGCGCGTGAAATGTATAAAGAACAATTGCTATTTAGG
GAGAATCCAGCTGAAGAGCCAGAATATTCTAGTATAGTAGAAATAGATCTATCAGAAGTTGAATCTAACC
TTGCTGGTCCTAAGCGTCCACAAGATAGAGTTGCATTCCATGATATGAAAAAAGCTTTTGCAGAAGCTTT
GGTACATGAGCAAGGATTACATGGTTTTGGCTTAACTGACGAGCAATTACAAAAATTAGCTGAAGTAAAA
GGTCTTAATGAAAGAATTACTCACGGTTCAGTTGCTATTGCTGCAATTACTTCATGTACAAATACTTCTA
ATCCATCACTGCTTTTAGGTGCTGGTTTATTAGCTAAGAAGGCAAATGAGAAAGGCTTAAAAGTTAAGCC
TTTTGTTAAAACATCATTAGCACCAGGATCTCAAGTTGTAACTCAATACCTTGAGAAAGCTAATTTATTA
CCAGAGCTTGAGAACTTAGGCTTCAACCTTGTTGGTTATGGTTGTACAACTTGTATCGGTAACTCTGGTC
CTTTAGATGAGCCTGTAGTAGAGGCTATTAACGAGGCTGATCTTATAGTGGCTTCTGTAAGTTCAGGTAA
CCGTAACTTTGAAGGTCGTATTAATCCTCATGTTAAGGCAAATTACCTTGCATCACCAATTCATGTGGTT
GCTTATGCATTAGCTGGAACTGTTGATTTTGATCCAGTTGAGGATGCTATTGGTAAGGATGCAGAGGGTA
ATGATGTTTACCTTGCTGATATTTGGCCAACTACAGAGGAAATTGCTGCTATTCAATCTCATGTAATTAA
CTCTGATATGTTTAAAAAGGCTTATGCAACAGTTCTTGATGGTACTGAGGACTGGCAAAAACTTAAAGCT
CCTGAAGGTAAGCTGTATGAGTTTGATAGTTCTTCTACTTATATCCAATGTCCTAACTTCTTTGAGAAGT
TTGCAGAAGGTAATGATGATTTAGATATCAAAGGTGCTAGAACTCTTTTAATGTTAGGTGATTCTGTAAC
TACAGACCATATTTCACCTGCTGGAGCTATTCCAGAAGAGTACCCTGCTGGACAGTATCTAAAATCTCAT
GGGGTTGAAAAGAAAGATTTTAACTCTTATGGTTCTCGTCGTGGTAACCATGAAGTTATGATGAGAGGTA
CTTTTGCTAATATCCGTATCCGTAACTTACTTTTAGACAATGTCGAAGGTGGCTTTACTAAGTATCACCT
TGATGGATCTCAACAGTATGTATTTGATGCTGCTATGAAATATAAAGAAAAAGGTATTCCATTAGTTATA
TTAGCAGGTAAAGAGTATGGTACTGGTTCTTCACGTGACTGGGCTGCAAAAGGTACATTCCTATTAGGCG
TGAAAGCAGTTATCGCAGAGAGCTATGAAAGAATTCATAGATCTAACTTAGTTGGTATGGGTGTATTACC
ACTTGAGTATGTAAATGGACAGAATGCTAAGACTTTAGGTTTAGATGGTACCGAAATGTTTAATATTAAG
AACTTAAACAATATCAAACCGCGTCAAATAGTTATTGTAGAGGCAGTACATCCTAAGACTGCACATACTA
CAACATTTGAAGCATTAGCTCGTTTAGATGCTGATGTTGATGTTGATTACTTGAAGAATGGCGGTATCTT

ACAAACTGTTCTTAAAGACATTATGGGCGATAAAAAAGAATCTAAGTCTACGCAATCAACTACTAGTAAA
GGTTGTGGTAGTGCAGATACTTCTTCTGAGACATCTTGTCCATTTGCAAAGATTGCTAATTTCTTCAAGA
AACTATTTAAGTAA

>lcl|NC_006570.2_cds_YP_169179.1_94 [gene=msbA] [protein=lipid A transport protein ABC transporter ATP-binding protein/permease] [protein_id=YP_169179.1] [location=115424..117253]
ATGGCTAATATGATTGATAAAATAGATCTTAAGTCTCAGGGATCTAGTAATCTAAGTGGAGAAATGACAA
ACCATCAGAAAGTAGGCACTCTTTATAAAAGGTTGTTACTCCAAGTTAAGCATTTATGGCATTTTCTGCT
TTTGGCTGCTATCGGAAGTATATTTTTCTCAGCGGCTGATGCCTCAATGATATATCTGATTAATCCGATT
TTGAATTATGGTTTTGGTCCTGGTGGCGGGATTACTAAACAAAGTGCTACTATACTAATGCTTATGGGTG
TTGGCATGGTTGGTTTACTAGCGTTAAGATCAGTAGGTTCATTTGTGTCACAGTACTTTATAGGTTCTTT
AGGTCAAAAAGTTGTTTATAAATTTAGGAAAGATATTTATAAAAGGTTAATGGATCTACCAGCTAGTTTC
TTTGATAAGCACTCAACAGGACAGATTATCTCAAGATTATTATACAATGTTGATCAGGTTATAGAGGCTA
CTTCTACAGCAATTATAACAGTTGTTCAGGATGGAACTTTTGTTATTGGGCTAATTGTTGTAATGTTTGT
CTCAAGCTGGCAATTGTCACTATTTTTAATAGTTGTCGGACCATTTCTGGGATTATTTATATCTATAATT
AATAAAAAGTTTAGGAATTTAAGTAGAAATACTCAGTCATCAATGGGTAATGTTACGCATACTGCTGAAG
AGACGATTAGAAACTATAAAGAAATAAGAATTTTTGGTGCGCAACAAAAACAACAAAATAAATTCTTTAA
AAATCTTGATTATACATACTCACAGCAAATTAGAACGATAGCATTAGATGCTCTGACATCACCAGTAATA
CAAATTATAGCTTCATTAGTTTTGGCTTTTTCATTATTTACGATAGCTATATTTGGTACTAATGAGGGCG
ATGGTTCATCTTGGTTAACCGCGGGTTCTTTTGCTTCTTTCTTTGCAGCTGCAGCAGCTATTCTAAAACC
GATTAAGAATCTTACTAAAGTAAATGTTGTTATCCAAAAGGCGGTAGCAGCAACTGAGGATATATTTTAT
ATCCTTGATTATCCAGCAGAGAAAGAAACTGGTAGTAAGGAATTAGCTAAAGTTGATGGTAATGTAACTA
TCAAAGATCTAAGTTTTGCTTTTGGTGAACATAAAGTACTTAGTGGCGTAAGTGTTGATATCAAAGCAGG
TCAGACTGTAGCATTTGTTGGTAAGTCAGGAAGTGGTAAAACTACTTTGACCAGTATTATATCGAGATTT
TACACTCAGCATGAAGGTGAGATTCTTCTTGATGGAGTTGATACAAGAGAATTAACTTTGGAGAATCTAA
GGTCGCACTTGTCTATAGTTTCACAGAATGTTCATTTATTTGATGATACAGTTTATAATAATATAGCTTT
TGGCCTTTCAAGAGAGGTTTCCGAAGAAGAAGTAATCGATGCGCTAAAAAGAGCTAATGCATATGAGTTT
GTCCAAGAATTATCTGATGGTATTAATACTAATATAGGTAATAATGGTTCAAAGCTATCAGGAGGTCAGC
GTCAAAGAATATCAATAGCAAGAGCTTTGTTAAAAAATGCTCCTGTATTAATATTTGATGAGGCAACTAG
TGCTCTTGATAATGAATCTGAGAGAGTAGTACAGCAAGCTCTTGAGAGTTTGACTAAATCATGTACTACT
ATAGTTATAGCTCATAGACTTAGTACCGTTGAAAATGCTGATAAAATTGTCGTGATGGATGGTGGTAGGG
TTGTTGAAAGTGGTAAGCATCAAGAATTGCTAGAGCAAGGTGGACTTTATACGAGGCTCTATCAATCAGG
ACTTCAATAG

>lcl|NC_006570.2_cds_YP_169180.1_95 [gene=lpxK] [protein=tetraacyldisaccharide 4'-kinase] [protein_id=YP_169180.1] [location=117259..118227]
ATGCTAGATAAGATTTGGTACAGATCAAAACCAAACTTGCTTAGTCGGGTGCTACAACCAATATCTTTGG
TTTTTATAGATATTGCAAATAAACGTAAAATAAAACAGCAACTCAAGCAATATAAATCAAAAATTCCTAT
AATAGTTGTTGGCAATATCTCTGTTGGCGGTACTGGCAAAACTCCAGTTGTTAGAATGTTAGCTCAGCAA
TATTTAGCACAAGATAAAAAACCAGCTATAATTAGTCGTGGATATGGTGCAAAGGCTGATAATTATCCTT
TTGAAGTAACAAGTGGTACTCTAGCAACTCAATGTGGCGATGAGCCTGCGATGTTATTTGATGCTTTGCA
AGCACAGGTTCCTATTGTTATTGCTCCAGAGAGAGTTCAAGCTGTTAAATACATTGAAAAGAATTTTCCT
GATACAGATATAATTATGTCTGATGATGGCTTGCAACATTATAAATTAGCTAGAGATAAGGAAATAGTGG
TCGTAGATGCTATTAGAATGTTTGGCAACAAATTATGTTTGCCTGCTGGTCCATTGAGAGAACCGATTGA
GAGATTAAAAGAAGTAGATCAAATTATAGTTATAGGTAATTGCTCAGATAAAGATAAAGAGTTACTCAAA
AACTATAAAAATGTGACTTATGCAAAAGTCGTAGCTACTGAATTTGTTAATATATTAACAGCTAAAAAAG
TAGCTAAGACTGAATTTAATCATCAAAATGCAATAGCTATAGCCGGGATTGGCAATCCAACAAAATTTTT
TAAGACTTTAGAAGAGAGTGCTATAAACATAACAGCTAAAAAAGTTTTTAAAGATCACCATAAGTTTACT
CAGAGTGATTTTGAGGGTATAGATAGTGACATAACTGTAGTGATGACATATAAAGATGCTATTAAATGCA
AAAATTTTGCTAAAGCTAATTGGTGGTATCTGGATATAGCTTTAGATATCAATGTTTAA

>lcl|NC_006570.2_cds_YP_169187.1_102 [gene=tmk] [protein=thymidylate kinase] [protein_id=YP_169187.1] [location=126208..126837]
ATGCAAAGTAAATTTATTGTAATAGAAGGTCTTGATGGTGCTGGTAAAAGTACAGCTATTAGCTTTGTTA
GGAAATATTTAGAAAAAAATAATCTAGCAGCAATTTATACTCGTGAACCAGGCGGAACTAAAATAGCTGA
AGAATTAAGAAACTTAGTTCTACATAATAAGTATGATGAAGAAATCCATTCAGATAGTGAGCTATTGATG
ATTTATGCTGGAAGAGTACAGCATTATAGAAACCTTATCGCACCAGCTTTAGAAAAGGGTATTAATGTGG
TTTCAGATAGATTTTACTGGTCAAGCATGGCTTATCAAGGTGGTGGTCGTGGGGTTGAGCTAAGCAAAAT
CAGAGCGTTAAATGATAATTTTCTTAATGGTTGTGAGCCAGATTTAGTAATCTATCTTGATATTGATCCA
ATATTGGGGCTACAAAGAGCTCAAAAAGTTGGAAGTCCTGATAGGATTGAAAAAGCTGGGCTAGAATTTT
TTAATAGAACCCGTAAAGTTTTCAAAGATCTTGTCAAAGATTCAGATAATGCTATTGAAATTGATGCTGC
AAAGTCTATTCAAGAAGTTGAAAAGCAAATATATCTAATATTAGATAAACATTTTAATTTTCAAAATTAA

>lcl|NC_006570.2_cds_YP_169189.1_104 [gene=FTT_0119] [protein=hypothetical protein] [protein_id=YP_169189.1] [location=128551..129867]
ATGTTAAAAAGTATCAGTAGGGTTCTTGTAGCTGGATTTGCATTAGCGAAGGTAAGTCCAGTTTTCTCAA
TGGAAATATATACCGTTAAATCAAATGATTATCTATATAAAATAGCAAAAAATCACGCAGTAACAGGTGT
AAGCATATCTGAGTTAACAGATGCTATTAAGGGTATCAATAAATCTGAGATACCTGGAATTATTGATAAT
AGAATAAGAATTGGTGATAAACTTGCTATCCCAACTACTAAGGCTGAGGTTGAGGATGGCTTAACACTGA
TGAGAAATCAGATAATTCAAAGCTCATACCAACAGCCTAGCAGTGATACAACTTCGCAACAACCTAGTAC
ACCTGCTGCTAATCTTGGTGATAATACAGCAGCTGCTAATGATGCTGATGATTCATCAACTCCAAGTGTG
GTAAGCCAAGACAAAATTCCAGTACTAATCCCTACTGATGATAATAGTACTCCAGAGACATATAAAAGTA
ATCTAGATACTCAAATAAACTCTGATAATATTCAAGAAACAGCATCTTATGAGCAACAGCCAACACAATC
TTCAAGTACTTGGGGATCATTATTTAGATTTATTATTTATGTCATAATATTAGCTGTTGTTGTTGTTGTT
GGTAAAAGGTTTTGGGAAACTAGAAACTCAAAAAAAGAGCAAGAACTTGAGCTCATATCAAAAAAGAAGA
GAGATCATTTGATGTCACGAATATCTCCGGTTGTTTCTGATAATGAGTTTTATAGATCTGATAAGGTTAA
TAATAGCCCACAAGAGGAATTTGATTTCTTTGGAGCTGCAAAATCATCTAGATCAGAGGTAACTACTGTT
GATGAAAAAATTCAATCACAGCAAGAATCTGAAGATACTTTTGAGCAGCCTGCTCAAAATGAAGAGGATT
TATTTGCTCAAAGAGATAAAAATATTATTGTCAAAACTGAGAAAGGCGTGGTCTTTGAGACCAATACAGA
TGACACACTTGTCAATTCTACAGCTGATGAAACTAAAGTTGAAGAGATAGACTCACAACAGCAAGCAGAA
CAAGAATTACAATATATAAACGAACTTATTGAACAGTTCTTAGATAGTGAAAAGTATGTTGAGGCTAGTA
TCACGATTCAGGATTCATTAGAAAAAGATCCAAATAATATTGATTTACGTTATAAGCTTCTAGAAGTATA
TGCTCGTGCAGGCGATGAAATAGCTTTTGAAGGAGAGGTACATTTTATTAAGTCTAAAAATATTGTCAGT
ATGTTTGATCCATTACATCAAAAAATTGCTAAACTTAGAGATAAATATTTTGAGTGA

>lcl|NC_006570.2_cds_YP_169190.1_105 [gene=ftsY] [protein=signal recognition particle receptor FtsY] [protein_id=YP_169190.1] [location=129864..130895]
GTGATTATTTACTTAACTAAATTTTTAGAGAGAAAGATGTTTTTCAAGAAAAAAAAGAATACAGAAATTG
AATCACCTATAGAAAGAGTACAAGAGCAGGATAATAAAAAAGGATTATTTTCTCGACTCCAAGCAGGTTT
ATCAAAAACAGCAAATAAGTTCGGTAGTGGTTTAAGTACCATACTTATGGGTCAAAAAGTTGTTGATGAA
GAGCTTTTGGAAGACATTGAGATGCAACTACTAACAGCAGATGTTGGTGTTGAAGCGACAGATGAGATAG
TTACTTATTTGCGTGACAAAGTTGCTAGAAATGAGTTGCAAACAGCTGATAAGCTTAATCAAATAATCCA
ACAAAAACTTACAGAAATAATTTTACCTTGTGAAAAGCCATTAGAAGTAGATACACAAAAGTCGCCTTTT
GTAATATTAGTTGTAGGGGTTAATGGTGTTGGTAAAACGACAACTATAGGTAAACTCACAAAGAAACTAC
AATCACAGGGAAAATCAGTAATTTTAGCTGCTGGTGATACTTTTAGGGCTGCCGCAGTCGAGCAGCTTCG
TGAGTGGGGTGATAGAAATAATACCCACGTTGTCTATCAGCATGAAGGAGCAGATAGTGCTTCGGTTATT
TACGATGCTATAGGCTCAGCTAAATCAAAAGGAATTGATGTTGTGATTGCTGATACAGCAGGTAGACTAC
ATAACAAAGATAATCTTATGCAAGAGCTTAAAAAAGTTGTTAAAGTTATCAAGAAAACTGATGAAACAGC
GCCACATGAGATTATGCTCGTTGTTGATGCAACAACCGGAGGCAATGCTTTAAATCAAGCAGAAGCATTT
AACCAAATCGTTAATTTAACTGGTATAACAATTACTAAGCTTGATGGCACAGCAAAAGGCGGTATTGTAT
TTTCAATTGCTAAAAAACTTGGTTTACCACTTAGATTTATAGGTGTAGGTGAAAAAATTGATGACCTACA
AGTTTTTAATGCCAGAGATTTTACTACTGCATTATTTAATTCTAACGATTAA

>lcl|NC_006570.2_cds_YP_169203.1_118 [gene=tufA] [protein=elongation factor Tu] [protein_id=YP_169203.1] [location=150045..151229]
ATGGCTAAAGAAAAATTTGAGCGTTCGAAGCCGCATGTAAACGTAGGTACAATTGGTCACGTTGACCACG
GTAAAACTACTCTTACAGCAGCTATTACAAAAGTTATGGCTGAGAAGAACGGTGGTATGGCGCGTAAATT
TGATGAGATTGATAGTGCGCCAGAAGAAAAAGCGCGTGGTATTACTATTAATACTTCTCACGTTGAGTAC
GAGTCTCCTAATAGACACTATGCTCACGTAGACTGTCCAGGACACGCTGACTACGTTAAGAATATGATTA
CTGGTGCTGCACAGATGGATGGTGCTATTCTAGTATGTTCTGCTGCGGATGGTCCTATGCCACAAACTCG
TGAGCACATTCTGCTTTCTCGTCAAGTTGGTGTACCAAAAATCGTTGTTTTCTTAAACAAGTGTGACATG
GTTGATGATGAAGAGTTATTAGAGCTAGTTGAGATGGAAGTTCGTGAGCTTTTAGATCAGTATGAGTTCC
CAGGTGATGACACTCCAGTTATTATGGGTTCAGCTCTTAGAGCTATTGAAGGTGACGAAGCTTACGTTGA
GAAAATTGTTGAGCTAGTTCAAGCTATGGATGACTATATTCCTGCTCCTGAGCGTGATACTGAGAAGCCA
TTTATTCTTCCGATCGAAGATGTATTCTCAATTTCAGGTCGTGGTACTGTTGTAACTGGTCGTATTGAGC
GCGGTGTAGTTAACATTGGTGATGAAGTTGAAGTTGTTGGTATTCGTCCAACTCAAAAAACTACAGTAAC
TGGTGTGGAAATGTTCCGTAAGCTTTTAGATAGAGGGGAAGCTGGTGATAACGTTGGTATCCTAGTTCGT
GGACTTAAGAGAGATGATGTTGAGCGTGGACAAGTATTATGTAAGCCAGGTTCAATTAAGCCACATACTA
AGTTTGAAGCTGAGGTTTATGTATTATCTAAAGAAGAGGGTGGTAGACATACTCCATTCTTCAAGGGATA
TAGACCACAATTCTACTTCCGTACTACAGACATTACTGGAGCTGTTGAGCTTCCAGAGGGTGTAGAAATG
GTTATGCCTGGTGATAACGTTAAGATGACTATCACTCTAATTAACCCAATCGCTATGGATGAAGGGTTAC
GTTTTGCAATCCGTGAGGGTGGTAGAACAGTAGGTGCTGGTGTTGTAGCTAAAATTATCGAGTAA

>lcl|NC_006570.2_cds_YP_169204.1_119 [gene=secE] [protein=preprotein translocase subunit SecE] [protein_id=YP_169204.1] [location=151421..151891]
TTGCCTGTGTATAAATTATTTTTTTTGAAGGATTGTTTTGTGAAAAGGAATCAAGGTTTTAATAATAACA
AAGTATGGATAAGTGGTGCAACCAAGACCACTGAAGTTAAAAAAATCTCAAAATCTAACAATTTGATGTT

ATGGTTGGCTGTTGTGGCTATCATTGTTTTGGGTGTTGCTGTTACTATGTATTCAGATATTTTGGGTGAT
AGCTATAGTACTTACAATACGTCTTTGGCAGTAGTTGTTGTGCTACTTGCTGTAGTTGTGGCTAGATTTA
CAAATCAAGGACGTCGTTTTTGGGCATTTTTCCAGGCTTCTAGGCTTGAGTTGGCAAAAGTTGTGTGGCC
TACACGTAAAGAGACAATGACTATTTCTCTGATGGTGATAGTTGTGGTAATAATTTTTGCGCTGATAATA
TCTTTATTTGGTGTTATATTTGAGAATTTTATTCAGTATTTTCTGGGTTAA

>lcl|NC_006570.2_cds_YP_169205.1_120 [gene=nusG] [protein=transcription antitermination protein nusG] [protein_id=YP_169205.1] [location=151907..152440]
ATGCTTTGGTATGTTGTGCAGGTGCACTCAGGTTACGAAAAAAGAGTGAAAGCTCAGCTTGAAGAAAATA
TCGAGATAGCTGGATTGAAAAATAATTTTGGCAGAATTCTTGTTCCTACTGAAAATGTGGTTGAGATGAA
AGGTGGGCAAAAGCGTAAGAGCGAAAGAAAATATTTTCCAGGCTATGTTTTGATTGAGGCTGATTTATCA
ACAGATGCTTGGAATCTTGTTAAATCGGTTCCACGTGTGCTAACTGTTGTTGGTTCTAAGGGTAAGCCGA
TACCTTTGAGCAAAGCGGAAGTAGATAGAATATTAGATTTTGTCGAAGGAAGTAAATCCACTGTAGAGCC
AAGATTGAGAAAATCTTATCACGTTGGTGAAGTTGTTCGAGTCCTAGAAGGACCATTTAATGACTTTACT
GGCGTTATTGAAGAAGTTAACTATGAGAAATCAAGGTTAAGGGTTGCAGTGTCTATCTTTGGTAGATCTA
CGCCTGTTGAGCTTGAGTTTTCTCAAGTTGAAAAAGAATCTTAG

>lcl|NC_006570.2_cds_YP_169206.1_121 [gene=rplK] [protein=50S ribosomal protein L11] [protein_id=YP_169206.1] [location=152509..152943]
ATGGCTAAGAAAAAAATAGAAGCTATTATTAAGTTGCAAGTTGCAGCAGGAAAAGCTAACCCTAGTCCAC
CAATTGGTCCAGCGCTGGGTCAGCATGGTGTTAATATTATGGGATTCTGTAAAGAGTTTAATGCTAAGAC
ACAAGGTATGGAGCCAGGTATGCCAATACCGGTAGAAATATCTGTTTATAGTGATCGTAGTTTTACGTTT
GAAATGAAGACGCCACCTGCTTCTTACTTAATTAAGAAAGCAATTAATGTAAAATCAGGTTCATCAAAGC
CTTCTAAAGAGTTTGTTGGAACTATAACTCGTGCGCAATTAGAAGAGATCGCAAAAGTTAAAGATCCTGA
TCTAACAGCTGCTGATTTAGATGCTGCTGTAAGAATTATTGCTGGTTCTGCTCGTAGTATGGGCGTAAAA
GTAGAGGGGGTTTAA

>lcl|NC_006570.2_cds_YP_169207.1_122 [gene=rplA] [protein=50S ribosomal protein L1] [protein_id=YP_169207.1] [location=152946..153641]
ATGGCTAAAGTTTCAAAAAGAATGAAAGAAATCTCAGCAAAAATTAATGCTGAGAAAAAAATATCCAGTAT
CAGAAGCTTTTGATCTTTTAAGAGAAGTTTCTTCTGTTAAGTTTGTTGAGTCTGTAGATGTATCTGTAGC
TTTAGGTGTTGATCCGCGTAAATCTGACCAAGTTGTAAGAGGAGCTTCAGTTCTGCCTAATGGTACTGGT
AAAACTGTAAGGGTTGCTGTTTTTGCAAAAGGTCCTGCAGCTGATGCGGCTAAAGAGGCTGGTGCTGAAG
TCGTTGGTATGGAAGATTTGGCTGATGAAGTTAAAAAGGGTAATATGGATTTTGATGTTGTCATTGCTTC
TCCAGATTCTATGAGAGTGGTTGGACAACTAGGTCAAATACTAGGTCCTAAAGGTCTTATGCCAAACCCT
AAGGTTGGTACTGTAACTATGGATGTTGCTAAGGCAGTTAGAGATGCTAAAGCAGGTCAAGTTAGATATA
GAGTTGACAAGGCTGGTATAATTCATACTACTATTGGTAAGGTAAACTTTACTAGCGATGCGCTTAAGCA
GAACTTAGAGCAGTTATTGACTGATCTGAAAAAAGCTAAGCCAGCAGTATCTAAGGGTATTTATCTGAAA
AAAGTTTCTGTATCTAGCACAATGGGTCCAGGAATAAATGTTGACTTTTCAGATTTGAACATATAA

>lcl|NC_006570.2_cds_YP_169208.1_123 [gene=rplJ] [protein=50S ribosomal protein L10] [protein_id=YP_169208.1] [location=153811..154329]
ATGGCACTTAGAATAGAGGATAAAAAAGCAATTGTTGCTGAAGTTGCTGAACAAGTGTCCTCAGCATTGT
CTGCAGCAGTAGCAGACTACCGTGGTTTGACTGTTAATGAAATGACTTCATTAAGAAAACAAGCTCGTGA
GTCTGGAGTTTATTTAAGAGTTGTGCGTAACAACTTAGCACGTTTAGCAATTAAAGGAACTGAGTTTGAG
TGTCTTGCTGATGCTCTTAAAGGTCCTCTTGTTCTTGCTCTTTCTAAGGATGAGCCAGGTGCAGCAGCTA
AGCTATTCAAAAACTTTCAAAAAGATCATAATGCTTTTGAAGTTAAGAATTTGGCTATGTCTGGTGAACT
GTTTGGTCCTGAAAAGTTAGATGACTTTGCTAAGCTTCCTACTAGGGAAGAGGCACTTGCTACATTACTT
AATGTTATGCAAGCACCAGTTACTAAGTTTGTTCGTACTCTTAATGAGATTCCGTCTCAGGCGGTACGAG
TATTTGCTGCTGTTGGAGATAGTAAATAA

>lcl|NC_006570.2_cds_YP_169209.1_124 [gene=rplL] [protein=50S ribosomal protein L7/L12] [protein_id=YP_169209.1] [location=154392..154769]
ATGGCTATAACAAAAGAAGATATCTTAAATGCTGTTGCTGAAATGAGCGTTATGGATGTATGTGATTTAG
TTAAAATGATGGAAGATAAGTTTGGTGTTTCTGCTGCTGCAGCTGTTGCTGTTGCTGCTGGTCCAGTTGC
TGGTCCTGCTGAAGCTGCTGAAGAAAAAACTGAGTTTGACGTTGTTTTAGTTGATGCTGGTTCAAATAAA
ATTGCTGCTATTAAAGCAGTAAGAGGCGCAACTGGTTTAGGTCTTAAAGAAGCTAAAGATGCTGTAGAAG
GTACTCCTTTCACAGTTAAAGAAGCTGCTTCTAAAGAAGAAGCAGAAGTTCTTAAAAAGCAACTTGAAGA
AGCTGGCGCTAAAGTTGAGCTTAAATAA

>lcl|NC_006570.2_cds_YP_169210.1_125 [gene=rpoB] [protein=DNA-directed RNA polymerase subunit beta] [protein_id=YP_169210.1] [location=154923..158999]
ATGTCTTACTCATACGCTGAGAAAAAAAGAATTCGTAAAGAGTTTGGGGTTCTTCCTCATATCTTAGATG
TACCATATTTGCTTTCTATTCAAACAGAGTCATACAAAAAATTCTTGACTGCAGATGCAGCAAAAGGAAG

ACTTCACTCTGGACTTGAAATAGTTTTAAAACAGTCTTTTCCTGTTGAGAGTAAAAATGGTCAATATGAA
CTTCACTATGTTGATTATCAAATTGGTGAGCCTACTTTTGATGAAACTGAATGTCAGGTTCGTGGTGCTA
CTTATGATGCACCATTAAATGTGAAGTTAAGATTAGTTGTATATAATAAAGATGCTCTTCCAAATGAGAA
AATTGTCGAGGACATTAGAGAAGAGTATGTGTACATGGGCGATATTCCGCTAATGACAACTAATGGTACT
TTTATCATTAATGGGACAGAAAGAGTAGTAGTCTCTCAGTTGCATAGATCACCAGGGGTGTTCTTTAGTA
AGGATGACTCTGAGGAAGGAGCATTCTCAGCACGTATTATCCCATATAGAGGCTCATGGCTTGATTTTGA
ATTTGACTCAAAAGGTATTATATGGGCTAGGATTGATAGAAAAAGAAAATTCTGTGCTACAGTTATTTTA
AAAGCTCTTGGTTATACTCAAGAGCAAATATTAGAGAATTTTTCTGAGAGTAAGACTATTACTTTTAATA
GTAAAGGTTTTGCTCTTAGACTTGATAACCTTTCAAATATGAAAGGTGAGCTGCTTAAGTTTGACATTGT
AGATGCTCAAGATAATGTTATTGTTAAAAAGAATAAAAAATTAACTTCAAGAGATGTTAAGAAAATTAAA
GATGCAGGCGTTGACTCTGTAGCTATTGATTTTGATTTGGTCAGCACTCTTAGAGTTGCAAAAGATATCG
TTAATGAAGCAACAGGTGAAGTGATAGCTTATGCTAATGACGATGTTACAGAAAGTTTATTAGAGTCATG
CGTAGAAGTTGGCATGCTTGAATTAGAAGTTATTGATTTTATAACTACTGAAAGAGGTAGGTATATATCT
GATACTTTAAAATATGATCTTACAAGAAATACTGATGAGGCACTTGTCGAGATATATAAGGTATTACGAC
CAGGTGATCCTCCTGCAGCAGCTTCAGTTAAAGCATTATTTGAAGGTTTATTCTTTATTGAAAGTAGATA
TAGCCTTTCTGATATTGGTAGAATGAAGCTAAATGCTAGATTAGGTTCGGATAAGGTATCTAAAGATATC
TATACTTTAGAAAATAGTGATATAGTTGGCGTAATTGAAGAGCTTATAAATATCCGTGATGGTAAAGGTA
AAGTTGATGATATTGATCATTTAGGTAACAGACGTGTACGTTCTGTTGGCGAGATGGTTGAGAATCAGTT
TAGAATAGGCCTCTACCGTGTAGAGAAAGGTATTCGTGAAAGTATGTCTTTAGTACATAAAGATAAGCTT
ATGCCAAAAGATATCGTTAACTCGAAGCCAATCACAGCAGCAATTAAAGAATTCTTTACTTCTGGAGCGT
TATCACAATTTATGGATCAAGATAACCCATTGTCAGAAGTTACACATAAGCGTAGAATATCTGCATTAGG
TCCAGGTGGTTTATCACGTGATAGAGCAGGTTTTGAAGTACGTGATGTTCATGCAACACACTATGGTAGA
TTATGTCCAATTGAGACTCCAGAAGGTCCAAATATTGGTCTAATTAACTCATTAGCAAGTTATGCTCGTG
TTAATGATTATGGTTTCTTAGAAGCACCATATCGAAAAGTTGTTGATGGTAAGGTTACAGATGAAATTGA
ATACTTATCTGCTATCGATGAAGATAATTATGTAATTGCTCAAGCATCAACTAAACTTGATGAAAATAAC
CATTTTGTTGAAGATCTTATTCAATGTCGTTCTGGTGGTGAGGCAATATTTACTGAGTCAAGTAGAGTTC
AGTATATGGATGTTTCTGCTAAACAGATGGTTTCAGCAGCTGCGGCACTAATTCCTTTCCTTGAGCATGA
TGATGCTAACAGGGTATTGATGGGTGCAAACATGCAACGTCAAGCGGTACCGACTCTTAAATCTGAGAAG
CCTTTAGTCGGTACAGGTATGGAGAAGATTGTTGCTAGAGATTCTGGTAATTGTATTATCGCAAGAAATG
CTGGTGAAGTGGCTGAAGTTGATTCTAACAGAATCGTTATTAAAGTAGATACTGAAAAATCACAAACTAG
TAATTTGGTTGATATTTATAGTTTAACTAAATTTAAACGCTCAAACAAAAATACTTGTATTAACCAGCGT
CCAATAGTAAATGTGGGTGATAAAGTAGAAGCTGGTGATATTTTAGCGGATGGTTTTGCAACTGATTTTG
GTGAATTGTCACTAGGGCATAACTTGATGGTTGCTTTTATGCCATGGAATGGTTATAACTTTGAGGATTC
GATCTTACTATCTGAAAGAATAGTAAAAGATGATAAGTATACTAGTATCCATATTGAAGAGTTCACATGT
GTGGCTCGTGATACTAAGCTTGGTCCTGAAGAGATAACTGCAGATATTCCAAATGTTAGTGAGTCTAGTT
TGGCAAAACTTGATGAATCAGGTATCGTCCATATTGGCGCTAATGTCGAAGCAGGCGATATTTTGGTAGC
TAAGATTACTCCAAAAGCTGAGCAACAACTAACTCCTGAAGAGAGACTACTTAGAGCAATCTTTAATGAA
AAAGCATCAAATGTTGCTGATAGTTCATTAAGAATGCCAAGTGGTACTTCTGGTACGGTTATTAATGTTC
AAGTATTTGAGAATGACAAAGGTGGTAAAAGTAAGCGAGCTCTTAAGATTGAAAAAGAACTGATCGATAA
AGCTCGTAAGGATTTTGATGAGGAGTTTGCAGTAATTGAATCTGTAGTTAAATCATCAATTGAGCAAGAA
GTTGTTGGTGCAAAAATCCAAAAAGCAAAAGGTCTTAAAAAAGGTGCTATCCTTACAAAAGAATTTTTAG
CAACACTACCTTTCTCTAAATGGCTAGAAATTTCTTTTGAGGATGAGAAACTTGAAGAGAAAGTTCAAAA
CGCTAGGGAGTATTATGAGGAAGCTAAAATTGCTATAGATGCTAAATTTGAAGCTAAGAAAAAAATCTATT
ACTCAGAGTAATGAGCTATCTCCAGGTGTGCTTAAGACTGTTAAAGTTTTTGTTGCGATTAAAAAGCGTA
TCCAGCCAGGTGATAAGATGGCTGGTCGTCATGGTAACAAAGGTGTGGTTTCACGCGTATTACCAGTTGA
AGATATGCCATATATGGAAGATGGTACTCCTGTAGACGTTTGTCTAAATCCTTTAGGTATCCCATCACGT
ATGAACATTGGGCAGATTTTAGAAGCACACTTAGGATTGGCTTCATATGGTTTAGGTAAAAAAATCGAAA
AAACTTTAGAGAAAACTAGAAAAGCAGCTGAACTAAGAAAGACTTTAGAAGAAGTATATAATAGCGTTGG
CGATAAGAAAGTTAATCTTGAGGCTTTAAATGACGAAGAGATACTAACTCTTTGTGACAACCTAAAAGGT
GGAGTACCAATCGCTACACCAGTATTTGATGGTGCTAAAGAAGAAGATATTAAATCACTACTGAAAATAG
GCGGCTTTGCAACTAATGGTCAGATGAAGCTTTTTGATGGTCGTACTGGTAAGCCTTTTGATAGACATGT
AACTGTTGGTTATATGTATATGCTAAAACTAGATCACTTAGTAGATGATAAGATGCATGCAAGATCGACT
GGCTCATATAGTCTAGTTACGCAGCAACCATTAGGTGGTAAAGCACAGTTTGGTGGACAAAGATTCGGTG
AGATGGAAGTTTGGGCATTACAAGCATATGGTGCTGCTTATACATTAAGAGAGATGCTAACAGTTAAGTC
AGATGACATAGCAGGTAGATCTAAAATGTATAAGAATATAGTTGATGGTAAGTTGACTATGAATGTAGAT
GTTCCAGAATCATTCAATGTTTTAAGAAATGAAGTTAGAGCATTGGGTATAGATATGGATTTTGACTATT
CATCTGAGGAAGAATAA

>lcl|NC_006570.2_cds_YP_169211.1_126 [gene=rpoC] [protein=DNA-directed RNA polymerase subunit beta] [protein_id=YP_169211.1] [location=159061..163314]
GTGAATAACGGTATCCTACATCAAAATTACAATAGTAAAAAGTTTGATATTATTAAAATATCTTTAGCAT
CGCCTGAGGTTATTCGTTCGTGGTCTCATGGTGAAGTTAAAAAACCAGAAACTATAAACTATAGAACATT
TAAGCCTGAAAGAGACGGATTATTCTGTGCTAAGATATTTGGTCCAATAAAAGACTATGAATGCTTATGT
GGTAAGTACAAAAGACTTAAGCACCGTGGCGTAGTCTGTGAGCGTTGTGGTGTTGAGGTTGAGCAAGCTA

AAGTAAGAAGAGAAAGAATGGGGCACATTGATTTAGTATGTCCTGTAGTACATATTTGGTATCTAAAGTC
TTTACCTTCTAGAATTGGTTTATTTTTGGATATGCCATTAAAGAATGTTGAGAAGGTTTTATACTTTGAG
TCATACATAGTTACTGACCCAGGTATGACTCCATTAGAGAAAAAACAACTTCTTACAGATGAAGAGTATG
CTGAAGCATTAGAAAATTATGGTTATGAGTTTGAAGCATCTATGGGTGCTGAAGCAATAAGAGATTTATT
GGCAGACACTGATATTGAGTCAGAGATTGAGTTACTTCAAGCTGAATGTGAAGAGAGTAAATCTACAGCT
AAGAAAGAAAAAGCTATTAAGAGATTAAGACTTCTTGAAACTTTCCAAGCTTCTGGTAATAAACCTGAAT
GGATGGTAATGACAGTACTACCTGTATTACCACCGGATTTAAGACCATTGGTACCAATCGAAGGTGGTAG
ATTTGCAACTTCTGATCTTAATGACTTATATCGCCGAGTAATCAACAGAAACAATAGACTTAAGAAGCTA
TTGGACCTAAATGCTCCTGATATTATTGTCAGAAATGAAAAGAGAATGTTACAAGAAGCGGTAGATGCTT
TATTAGATAATGGTAGACGTGGTAGAGCAGTAACGGGCTCTAATAAAAGACCACTTAAATCATTAGCAGA
TATGATTAAGGGTAAACAAGGTCGTTTCCGTCAAAACTTATTAGGTAAGCGTGTTGACTATTCTGGTCGT
TCTGTAATTACAGTTGGTCCATCTTTGAGATTACATGAGTGTGGTTTACCTAAGAAGATGGCACTAGAGT
TATTTAAACCATTTGTATACTCTAAGCTAAGGTTAGGTGGTCATGCAACGACTATCAAACAGGCTAAGAG
AATGGTTGAGCTTGAGGAAGCTGTAGTATGGGATATTCTTGAAACTGTGATTAATGAGCATCCGGTATTA
CTAAACCGTGCTCCTACATTACATAGACTAGGTATTCAAGCGTTTGAGCCTAGACTTATTGAAGGTAAGG
CTATACAGCTACATCCATTAGTTTGTGCTGCGTTTAACGCTGACTTTGATGGTGACCAAATGGCTGTACA
CGTACCATTAACAGTTGAGTCGCAACTAGAAGCTAGAGTATTGATGATGTCAACAAACAACATCTTATCA
CCAGCATCTGGTCAACCAATTATTACTCCTACTCAAGATATTGTATTAGGTCTATACTATATCACTAGAG
AGAAAGAAGGTGCGCGTGGCGAGGGTAAATTATTCTCTAGCTATGAGGATGTTAGTAGAGCTTATAACTC
AGGGACTATAGATATCCATGCTAAGATTAAACTAAGAATAGATAGACAAGTATTTGATACTAAAGGTAAT
ACATACAATGAGAAAGGTGTTGTTAATACTACCGTTGGTAGAGCACTTTTATTGAATATACTACCAGAAG
GTCTATCTTTCTCTCTATTAAATAAAGTACTAGTTAAGAAAGAAATTTCTAAAATTATTAATCAGGCATT
TAGAGTCTTAGGTGGTAAAGCTACGGTAGTTTTAGCAGATAAGTTGATGTATGCAGGATTTAAATATTCA
ACATTGTCAGGGGTATCTGTTGGTGTTGATGATATGACTATTCCTGATAACAAAGAAGCTAAAATTGAAG
AAGCAGAAAAAGAAATTAAGCAAATTACCGAACAGTATCAGTCTTCATTAATTACTGAAAATGAGAGATA
TAACAATATTATCAATATTTGGAGTAAGACTTCAGATGAGGTTGGAGCTTCGATGATGGATGCTATATCT
AAAGATACAGTTAGTATTAACGGTGAGAAAAAAGAAATCGAGTCATTCAACTCTGTATATATGATGGCTA
AATCAGGTGCAAGGGGTTCTTATAATCAGATGAGACAGCTTGCAGGTATGCGTGGTCTGATGGCTAAGCC
AGATGGTACGATGATTGAAACTGCAATTACAGCAAACTTTAGAGAAGGTCTGTCAGTATTGCAGTACTTT
ACATCTACGCACGGTGCACGTAAAGGTCTAGCTGATACGGCTCTTAAGACAGCTAACGCGGGTTATCTGA
CTCGTAGACTAGTTGATGTGGCTCAAGATTTAGTTGTTATTGAAGAAGATTGTGGTACTGATGATGGTTT
GATGTTCTCAGCTATCGTTGAAGATGGTGAAGTCAAAGTTCCTCTAGTAGAGCGTGCTTTAGGAAGAACT
TTAGCAGCAGATGTGGTTACTGAGAAGGGAGTTGTATTGCTTGAAGCAGGTACTTTATTAGATGAGAATC
TTGTTGAGTTACTTGATGATAACGGTATTGATATGATCAAAGTCAGATCACCAATAACTTGTAAAACACG
TAGAGGATTATGTGCTAAGTGTTATGGTCGTGATCTAGCGCGTGAAAGACAAGTTAATGTTGGGGAATCT
GTAGGTGTGATTGCTGCTCAATCAATTGGTGAGCCAGGTACGCAGTTGACAATGAGAACATTCCACACTG
GTGGTGCTGCGTCTCTGGGTATTACAGTTTCTGACATTAAAGTTAAAACTGCTGGTAAGATTAAGTTTAA
GAATATTAGAACTGTAACTAACAAAGAAGGTCAAGAAATTGTTATATCTCGCGCAGGTGAGATTATCGTT
TCTGATACTATGGGTAGAGTGAGAGAACAGCATAAGATCCCTATGGGTGCAGTCGTTCCTTTAGCTAGTG
GTAAAGCAGTAGAAATTGGTGATGTGATTGCTACGTGGGATCCGCATGCTCAGCCATTAATTACAGATGT
TGCTGGTAAAGTTGTTCTTGAAGATGTGATTGATGGTATAACATCTAAGCATACTTATGATGATTTGACT
GGTCAGCAAACTATTGAGATAACTTCAATATCTCAAAGAACAACATCTAAAAACTTAAAACCAGTTGTTA
AGATTGTTGATGAAAAGGGTGCCGAGCTTAAGTCAATACCTCTAGCAGTTGGAGCAGTACTAAACGTTGC
CGATGATTCTATACTAGAAGTTGGTGATATCGTAGCTAAGATTCCTCTAGAAGGTTCTAAGAACAAAGAT
ATTACCGGGGGTCTTCCACGTGTTGCTGAGCTTTTCGAAGCTAGACGTCCTAAAGATGCGGCAATACTTT
CTCCATGTGATGGTATGGTTAGGCTTGGTAACAGAGACACTAAAGAAAAACAGCGTATTGAGATTATAGA
TAAAAATGGTCATATTGTTGAAGAGATATTATTACCTAAGTCTCGTCACCTAGTAGTTTTTGATGGTGAG
CAGGTTTCTAGGGGTGATGTTTTAGCTGATGGACCTACTGATCCACATGATCTTCTTAAGTACAAAGGAC
TTGAGGAATTTGCTGATTATATTCTTATCGAAGCGCAGTCTGTATATCGTATGCAGGGTGTTGTTATTAA
TGATAAGCATATTGAAACAATTGTAAGACAAATGTTAAGAAAAGCTGTAATCCTTGATGAAGGTGATAGT
AAGTTTGTCAAAGATGAAAGTATTGAGTTAGTAAGAATACTTGAAGAAAATGATAAACTACGTAAGCAAG
GTAAAAAAGAAGTAGAGTATGAGCTGGTATTAATGGGTATTACACGCTCTTCTTTATCTACGGAGTCATT
CTTATCGGCTGCTTCTTTCCAAGAAACAACAAGAGTGCTTACTGAGGCTTCGATAAACTCACAAATTGAT
AATTTAAGAGGACTTAAAGAAAATGTTCTTATTGGTAGATTAATACCAACAGGTACTGGTCTAGCAGTAA
GAAAAGAGTCAGCTAAAATTGAAAAAATGCGTGAAGAATTAGGGGTTGAAGATAATATGGTGTTTACTGA
TTTATCGTCTTTTAACCCTGAAGAAATCTCTTTTGATAGTATTCAATCTCAAAAAGAAGATAAAGATATT
AATGAAGACATCGAGGAGTCATTAAGAAACGCTCTTGAGTCACTTGATTTCTAA

>lcl|NC_006570.2_cds_YP_169213.1_128 [gene=gcp] [protein=DNA-binding/iron metalloprotein/AP endonuclease] [protein_id=YP_169213.1] [location=163653..164663]
ATGATAGTACTTGGTATTGAGAGCTCCTGTGATGAAACAGGTTTAGCGATATATGATTATTCAAAAAAGA
AATTAATTGCAGATGAATTATATAGCCAAGTCAAATTGCATAAGAAGTATGGTGGTGTTGTACCGGAACT
TGCCTCGCGTGAGCATATCGCTAAGCTTAATCTTTTAGCTAAGAAAATATTCAAAGAAACTGGTCTTAGC
TTTGAAGATATTGATTGTATAGCATATACCGCGATGCCTGGTTTAGTTGGTGCATTGATGGTTGGAGCTA

CATTTGCCAAAACACTAGGCTTAATCCATAATATTGATACAATTGCAGTTCATCACCTTGAGGGTCATCT
TTTATCACCATTATTGGATCACAATAGTAATATAGAATATCCTTTCGTAGCCTTGCTAGTTTCTGGAGGA
CATACACAACTATTTGAGGTAAAAGAGTTTGGTGAGTATAGTTTACTAGGGGAATCAATTGATGATGCAG
CAGGTGAGGCATTTGATAAGACGACTAAGCTTTTAGGTATGGGTTATCCTGGTGGAGTTGAGGTGGCAAA
TTTAGCTGATCAAGCTACTGATAAGTCTAAGTATATTCTACCAAGGCCGATGAAAAATAAACCTAATTTA
GATTTTAGCTTTAGTGGTTTAAAAACTGCTGTGCTAAATACATGGTATGATGAGCAAGATCAGTCATTAG
AGAATAAGGCAAATCTATGCTACGCATTCCAAGATGCGGCTATAGATGTATTGGTTTCTAAATGTGCTAA
AGCATTACAAAAAACTAAAAATACAAGATTGGTTATTTCAGGCGGAGTCAGTGCAAATAAACTATTGCGT
CATCAGCTAGATTTATTGGCTAAAAATAGAGGATATCAAATATTTTTTCCTCCAATGAAATATTGTACAG
ATAATGGTGCAATGATTGCTCTAGCGGGAGCATATAGGTATGTAAATGGTTTTAAGGACTCTAATTTAGA
GATTAATGTTAAAGCAAGATCACCACTCTAG

>lcl|NC_006570.2_cds_YP_169215.1_130 [gene=metK] [protein=S-adenosylmethionine synthetase] [protein_id=YP_169215.1] [location=complement(166155..167315)]
ATGTCAAAAAATTACCTATTTACTTCTGAATCTGTATCAGAAGGACATCCAGATAAACTAGCTGACCAAA
TATCAGACGCAATCCTAGATGAGATTTTAAAACAAGATAAAAATGCTCGTGTAGCATGTGAAACTTTAGT
AAAAACTGGTATGGCTTTAGTTGCTGGAGAAATTACAACATCTGCTTGGGTAGATATCGAAGAATTAGTA
AGAAATGTTATTACAGAAACAGGCTATGATAATGCTAGTAAAGGTATTGATGGTAGAACTTGTTCTGTAA
TTAATGCTATTGGTAAACAGTCTAGAGATATTACCCAAGGCGTAGATCGTGGCTCCCTAGAAGATCTTGG
TGCTGGTGACCAAGGATTAATGTTTGGATTTGCTACAAATGAGACACCAACACTAATGCCATCAGCCATT
TACTACTCTCACCTATTGATGAGAAAACAAGCAGAACTTAGAAAATCAGGGAAGCTTGCATGGTTAAGAC
CAGATGCAAAAGCTCAAGTTACATTAGCTTACGAAAATGATAAGCCTAAATTCATTGATACTATAGTACT
ATCTACACAACATAATGAATCAATCTCTCAAAAAGAATTGCATGATGCTGTGATTGAGGAAATTGTTAAA
AAGGTTATTCCTAACGAACTAATTACTAAAGATACCAAATATCATATTAACCCAACAGGTGTATTTTTAA
TTGGTGGTCCTCAAGGCGATTGTGGCCTTACAGGAAGAAAAATTATTGTTGATACTTATGGCGGTGCTGC
TCATCATGGTGGTGGTGCTTTCTCTGGCAAAGATCCTTCTAAAGTTGATCGCTCAGGGGCATATATGGGT
AGATATATAGCTAAAAACATTGTTGCTGCTGGTCTAGCTGACAAGTGTGAAGTACAAGTAGCGTATGCAA
TTGGTGTAGCAAAACCAGTTTCCCTTATGGTTAATACTTTTGGTACTGGCAAAATTACAGATAATCAGAT
AGAAAAGCTCGTAGCTGAAGTTTTTGATCTTAGAGTTGGTAAAATTATCGAAAACCTTGACTTATTAAGA
CCAATTTATCGCAAAACTTCTAACTATGGTCATTTTGGTCGCGAGTTACCAGAGTTTACTTGGGAAAAAA
TTGATAAAGCTGATATTCTTAAATCAGCAGCCAGAATATAA

>lcl|NC_006570.2_cds_YP_169216.1_131 [gene=rpsP] [protein=30S ribosomal protein S16] [protein_id=YP_169216.1] [location=167609..167857]
ATGGTAGTAATTCGTATGGCTCGTGGTGGAGCTAAAAAGCGTCCTTTCTATAGAATCGTAGTTGCTGATA
AAAGAAGTCCAAGAGATGGTAGATTTATTGAGAAATTAGGTTTCTTCAATCCTTTAGCTAAAGGTGGTGA
AGAGAGATTAAAGCTTGATGTTGCTAAAGCTGAAGCATGGTTAGCAAAAGGTGCACAACCTTCTGATAGA
GTAGCAAGCTTAATCAAAGAAGCTAAGAAAGCCGCTTAA

>lcl|NC_006570.2_cds_YP_169217.1_132 [gene=rimM] [protein=16S rRNA-processing protein RimM] [protein_id=YP_169217.1] [location=167900..168409]
ATGTCACAGGATTTTGTAGAAATAGCCAAAATTGGCGCTACTTATAAGCTTAATGGTGAGCTTAATTTAT
ATCCTTTGGCAAATTCAATAGAAACACTTCTAAGTTATGGTGATTGGTATATCCAATTACCAGCGACTAA
TGTTTGGCAACAGCTCAAAGGTGAAAGTGTACTAAAGAGGGCTGATAAAGTTTATATTAAACTTGCTAAC
ATTAACAATGCTGATACTGCTAAGAAATATGTAAATGCACTGATTGGTGTGCCAAAGCGAGCATTACCAC
AGCTAGCAGAGGATGAAGTGTATTTTAAAGATTTAATTGGTTGTAGTGTCAAAAATATTAACAATGATTC
ATTTGGTGTTGTTGTAGATATTATTGAAACTGATGCTAATGAAGTTTTGGTATGTAAAGAAGATAATAGT
GAATATTTGATTCCTTATGTTAAGCAATATATTGTTAGTGAAGATCTTAATTCTAAAAAGATAGTTGTTG
ATTGGGAATACGATTATTAA

>lcl|NC_006570.2_cds_YP_169218.1_133 [gene=trmD] [protein=tRNA (guanine-N(1)-)-methyltransferase] [protein_id=YP_169218.1] [location=168806..169570]
TTGAAGATGAAATTTGGGATAATATCAATATTTCCAGAGATGTTTAAAGCAATAAATGATTTTGGAGTTA
CAGCAAGAGCTATAAAGGACTCTAAAGTATCGATAAGTTGCTTTAATCCGCGTGACTACACTACAGATAG
ACATGCTACAGTCGATGATACTAGTTTTGGTGGTGGCGCAGGGATGGTAATGAAGTATCAACCTTTATCA
GCAGCTATTGAAGATGCAAAAAATACTTTAGGTTGTGGTACAAAAGTAGTATACTTGTCACCTCAGGGTA
GTATATTTAATCATAGAAAAGCCCAAGAGCTTTTGCAAAATGATTCATTAATACTGCTCTGTGGTAGATA
CGAAGGGGTTGATGAGCGCCTAATACAAGATTATGTTGACGAAGAAATTTCGGTTGGAGATTTTGTGTTA
AGTGGTGGTGAGCTCCCTGCTATGCTAGTTATGGATAGTCTAATTAGGCTGTTACCGGAAGTGTTGGGGA
ATAAAGAATCTGTCGTAGAAGATTCATTTTATGATGGTCTTTTGGATTATCCACACTATACAAAACCGGC
TGTTTTACCTAATGGTAATGCAGTACCTGACGTTTTGTTATCTGGTAATCATAAAGAAATAGCTAAATGG
AGGCGTAAACAAAAGTTAATAAGAACTTATGAGCGTCGTAAAGATTTAATAGAGTGCCTATGCCTATCTG
CAAAAGATAAGCAAATTTTAGATGATTATAAAATAGATAAGGTAAGCACAAAAGGAGAAGAATAA

>lcl|NC_006570.2_cds_YP_169219.1_134 [gene=rplS] [protein=50S ribosomal protein L19] [protein_id=YP_169219.1] [location=169570..169917]
ATGAAAAATAAATTTGTTGAGCTAGTAGAAAAGTCACAATTAAGAACTGATCTTCCAGAATTTAATCCAG
GTGATTCTATTACTGTTAATTTATGGATTAAAGAAGGCGATAAGCAAAGAATTCAGGCTTTCAAAGGTTT
CGTTCTTAGAAAAAGAAATAGAGGTCTTCACTCTGCTTTCACAGTAAGAAAGATGTCTTCAGGTATGGGT
GTTGAAAGAACTTTCCAAACACACTCACCATTAATAGATAGTATTATTGTAGAGAAGAGAGCAGATGTAC
GTAGAGCTAAGCTTTACTATATGAGAGGTCTTACTGGTAGGGCTGCTAGAATTAAAGAAAAAGTATAA

>lcl|NC_006570.2_cds_YP_169226.1_141 [gene=nudH] [protein=dinucleoside polyphosphate hydrolase] [protein_id=YP_169226.1] [location=175200..175667]
ATGATAGATAAAAGTGGGTATCGAGCAAATGTGGCGATAGTTTTACTTAACAAGCAAAATAGAGTATTTT
GGGGACAGCGAAGAAACCGCACATCTTGGCAGTTTCCACAAGGTGGTGTAGCTACTGGAGAAACACCTTT
GCAGGCAATGTATCGTGAGCTACATGAGGAGATTGGCTTGCGTCCACAAGATGTTGAGGTAATCGCTTCA
ACAAGAGATTGGTATAAATATGATATTCCAGACTCATTAGTTAGAACTAAAGAACCTATATGTATTGGTC
AAAAACAGAAATGGTTTCTATTAAAATTAAAGAGTCCTGAAAGTTATATTGATTTAGACGCTAATGACTC
ACCTGAATTTGATAATTGGCGTTGGGTAAGTTATTGGTATCCAATCAATCATGTAGTGTATTTCAAACAA
GAGGTTTATCGTAAGGCTTTGACTTATTTTAAGGAGTATATAGCTTAA

>lcl|NC_006570.2_cds_YP_169233.1_148 [gene=hemA] [protein=glutamyl-tRNA reductase] [protein_id=YP_169233.1] [location=182323..183573]
TTGAATATGGCGTTAATATCTTTAGCAATTGATTATAAAAAGTCTCCGATAGAAGTTCGTAGTGAGTTTG
CACTCTCTGGTTTAGATGTATCTATGCTATATAGATCAATACTTGCTATAGATAATGTTGTACATGCTGT
AATCTTATCAACTTGTAATCGTACAGAAGTATATCTTGAGATATCTGATCTAAGAGTAGTTGATGATATT
TTAGTATGGTGGCAAGGCTATGTTAGAAACCCTAATTATAAAATAAAAGATTATTTTAAGCTAAGACAAG
GTACAGAAGTAATTATGCACCTTATGAAATTGGCTTGTGGTTTAGAATCGATGGTTTTAGGTGAACCGCA
AATATTAGGACAAGTAAAAGATTCTTATACTCTCAGTAAAAAAAATCATGCTATTGGTAAAGAACTAGAT
AGGGTTTTTCAGAAGGTTTTTGCAACTGCTAAAAGAGTACGTAGTGAAACTAGAATTGGTCATTGCCCAG
TTTCAGTTGCATTCTCAGCAATTACTTTGGCTAAAAGGCAACTAGATAATATTTCTAGTAAGAATGTCCT
TATTATTGGTGCTGGTCAAACAGGAGAGCTTTTGTTTCGTCATGTTACAGCACTTGCTCCTAAGCAGATA
ATGCTAGCAAACCGAACTATCGAGAAAGCACAGAAAATTACTTCTGCTTTTAGAAATGCTAGTGCTCATT
ACTTATCAGAGCTACCGCAATTGATCAAAAAAGCTGATATTATAATTGCTGCAGTCAATGTTTTAGAATA
CATTGTCACATGCAAATATGTTGGCGATAAACCTAGAGTTTTTATTGATATATCAATACCTCAAGCTTTA
GACCCTAAATTAGGCGAGTTAGAGCAAAATGTTTATTATTGCGTTGATGATATTAACGCTGTTATTGAAG
ATAACAAAGATAAAAGAAAGTATGAAAGCTCTAAAGCACAGAAAATTATTGTAAAATCTCTTGAGGAATA
TCTTGAAAAAGAAAAGGCTATTATCTCAAATAGTGCGATTAAAGAGCTTTTCCAAAAAGCTGACGGTTTA
GTTGATTTATCGCTAGAGAAAAGCTTAGCTAAGATAAGAAATGGTAAAGATGCCGAAGAGATAATTAAAA
GATTTGCTTATGAAATTAAAAAGAAAGTTTTACACTATCCTGTTGTAGGAATGAAAGAGGCTTCCAAGCA
GGGTAGAAGTGACTGTCTTGTATGTATGAAACGTATGTTTGGTTTAAATGTGGAAAAATAA

>lcl|NC_006570.2_cds_YP_169234.1_149 [gene=prfA] [protein=peptide chain release factor 1] [protein_id=YP_169234.1] [location=183574..184659]
ATGAAAGATTCTATTAAAGCAAAATTGCAAAGTTTGATAGAAAGGCATGAGGAAGTTAGTGCCTTATTAA
GTGAGGCAGGAATTATCTCTGATCAAAATAAGTTCAGAGATTTATCAAAAGAATACTCTCATCTTGAGCC
GATTGTAAAGGCGTTTAAAAAATATACACAAGCTTTAGAAGACAAACAAGCTGCTTATGAAATGCTTAAT
GAAAAAGATGCTGAGCTTGTAGAGATGGCAAAAGAAGAATTAAAGTTGGCAAATGAAGCTATTGAGAAAC
TGGAGAGTGAATTACAAATATTTCTTTTACCTCGTGATCCTAATGATGATGCTAACGTTTTCTTGGAGAT
TCGTGCAGGAACTGGCGGTGATGAGGCTTCGATATTTTCAGGTGATTTGTTTAAGATGTATTCAAAATAT
GCTGAGCAAAGAGGCTGGAAGATTGAAGTAATATCGGCTAGTGAAGGCGAGCATGGCGGTTATAAGGAAA
TCATCTCAAGAATATATGGCGATGGTGTATATTCACAACTAAAATTTGAATCTGGTGCTCACCGAGTACA
AAGAGTTCCTGCTACAGAGTCGCAGGGTAGAATCCATACTTCAGCGTGTACTGTTGCGGTTATGCCTGAA
GCAGATGAGGTTGAGGGGATTGATATTAATCCTGCAGATATCAAAGTGGATACTTTTAGAGCATCGGGTG
CGGGAGGACAGCACGTCAACAAGACTGATTCAGCGATTAGGATTACCCATATCCCAACAGGTGTTGTTGT
CGAATGCCAAGATCAAAGATCACAGCACAAAAACCGTGCAGCTGCAATGTCGATGCTTAAATCAAAGCTG
TTACAAGCAGAAATTGATAAACAGCAAAAAGAGCAATCTGATACACGTAAGAGCCTGGTCGGTAGCGGTG
ATAGATCAGAGAGAATTAGAACTTATAATTACCCGCAAGGTAGAGTTACCGATCATCGTATAAATTTAAC
TCTTTATAAGCTTGATGAGGTCATGGAAGGTAGTCTAGATAGTATTATCCAACCACTAGTTCTAGAACAT
CAGGCGGATCTTTTAGCAACCATGTCTGATGAGTAA

>lcl|NC_006570.2_cds_YP_169235.1_150 [gene=FTT_0169] [protein=HemK protein] [protein_id=YP_169235.1] [location=184652..185506]
ATGAGTAATATTACAATTTCTCAATTATTAGCTTTAAGCTTAGCTAAATTTAATCAATGCGATATATCAA
CCAAACATGATCTACAAATGATTATTTGTGATGTTTTGGGTGTTGATAAAACTTATCTTTATATAAATTT
GGATAAGCAGTTAGACAAAAATACACTTAAAAAAATAGATGAGAAAATCTTACGTCTTTTAGCTGGTGAA
CCATTGGCTTATATTCTTGGTTATAAGTATTTTTGGAATCAAAAACTTTATGTAACTAAAGATACTCTCA

TACCACGGGCAGATACTGAGACTGTTGTTGCTACAGTGTTAGATGATATACAAAATAAGGACGCTCAATT
AAAAATTTTAGATCTTGGTACAGGTACGGGAGCGATAGCTTTGGCTTTAGCAGCTGAACTAGCAAATAGT
CAAGTTGTTGCGGTTGATTTATATCAGCAGAGTCTTGATGTTGCTAAAAAAAATGCCCAAGCAAACAATA
TCACTAACGTTAAATTTATCCAAAGTAGTTGGTATACAAATCTCGATACTGATAAATTTGATATTATAGT
TTCTAATCCACCATATATAGATTTAGCGGATACAAATATTGACCAAAGCGTCAAAGACTATGAACCCGCT
CGAGCTTTATTTGCTGCAGATAATGGTTTAGCGGATATTAGGATAATTATATCTCAAGCAAAAGATTTTC
TAAATCTAGGGGGCTTCATTTATATAGAGCATGGTTTTACTCAGGCAGATGCTATTACAGCTTTATTTTC
TCAGTGTAATTTTACTGATATTAAAATAGTTAAAGATTTAAATAATAACGATAGATGTACTAAGGCACAA
CTTGGCTATTTATGA

>lcl|NC_006570.2_cds_YP_169242.1_157 [gene=FTT_0181c] [protein=hypothetical protein] [protein_id=YP_169242.1] [location=complement(197194..197730)]
ATGGAAAAGATTAGTCATAAAGATATACAAGATAAATTATGGAGTAATGAAGATGAAAGTAATTTATCAA
ATGAGCAATATAATTCACTACTAATTGAACAGTATAGAATCTATGTTGAATTAACAGACAGAACTAGCTA
TAGAAGGATTGTTATTAACCTATTCTTTTTGGTTTTTAACCTTGTTCTAGTCGGAGTTGTTGCCTTAGCT
ATTAGTAACAACATTAATGTCGAAAATCCTCCATCTAGTATACTTGTGAGTATTCCATACTTTGCTGGGT
TAGTATTTTGTTATGCTTGGTGGAAGATTATTAGATTCTTTAGACACCACATACAAATAAAAAATAGTAT
TGTGCCATCACTTGAAAGACGTCTTCCTTCAAGAGTATGGTTAACAGAAGAGCACATTGCTGAAGAAAAA
GGTTCATTTAAACCTATTAGAATATTAGAAATATATATGCCTTTTATTTTCATGGGTATTTATACAGCAT
TATTTTTATTTGTCGAAATTGCTTGGTTACCTCATACTTTAAACTAA

>lcl|NC_006570.2_cds_YP_169243.1_158 [gene=FTT_0182c] [protein=Sua5/YciO/YrdC family protein] [protein_id=YP_169243.1] [location=complement(197828..198370)]
ATGCTTACAAAAGATTTGAATAAAATCATCGGTGAAATAACAGAAGATAATGTTGTTAGTATTCCTACAG
ATACAGTATATGGGCTAAGTTGCAACATCTCTAAAGCTGCCGTAGCAAAAGTTATAAACTTAAAAAAAAG
GGATTCAAGCAAAGGTTTTATTATAATCTCACATGATCACAAGCATTTACTCAAGTATGCAGATACAACT
AAACTATCTAATGAGCAAATCAATAAAATATCCTCTAAACAAGCTCAACCAACTACTTGGATAGTACCAG
GTAAGAAAGATATTCAATGGTTAACTGGTGATAAAACAACTATTGCCATACGCTTAGTAACAACTGAAAT
AGTAACTTATATTTGTGAAAATATTAATGATGCTATTATTTCAACTAGTGCTAATATATCCGGCAAAGAT
TTCATTAATAATGCTAAATCGATAAGTAAAACCTTTGATAATATTTATGTACTAGAAACAGAAGTTAGGT
CTTCACAACCATCTAGAATTATTGATATAATTAGCGGACAGCAGTATAGATAA

>lcl|NC_006570.2_cds_YP_169244.1_159 [gene=rpsA] [protein=30S ribosomal protein S1] [protein_id=YP_169244.1] [location=complement(198487..200157)]
ATGTCAGAAAATTTCAAAGAACTATTTGAGCAATCTCTTAAACAAACAGAGATGAGAATAGGTAAAATCA
TCGAAGCAACTGTAGTAAGCATAGACAAAGAATTTGCAATGATTGATGCTGGTCTTAAGTCAGAATCATT
CATCCCTGTATCTTCTTTAAAAAATAGCAATGGTGAGCTAGAAGTTGCTGCTGGTGATAGAATCAACGTT
GTTTTAGAAGCTCTAGACAACAGTTGCGGTGAAACTAGATTATCAAGAGATAAAGCTAAGAAAATCGAGC
TTTGGGATAGAATTGAAAAAGCTTTCGAAAATAACGAAACAGTTCTTGGTAAAATCACTAATCACGTTCG
TGGTGGTTACACTATGGATGTTGAAGGTTTAAGAGCATTCTTACCTGGCTCATTAGTTGACACAAGACCT
ATCAAAGATGTAGCTCATTTAGAAGATAAAGATATCGAATTAAAAGTTGTTAAAATCGATACTAAGAGAA
ATAACATCGTTGTTTCTAGAAAAGCAGTTATAGAAGAGAATAACTCTGGTGATAGAGATGCTATGCTAGA
GAAAATCTCTGAAGGTAGTGTTCTTAAAGGTATCGTTAAAAATATCACTGATTTCGGTGCGTTTATTGAT
CTTGGTGGAGTTGATGGTCTACTACACATCACTGATATCTCTTGGAGCAGAATCAGCCACCCTACAGATG
TATTATCTATCGGTCAAGAAATCGATGTTAAAGTAATCAAGTTCGACAAAGAGAAGCAAAGAATTTCTCT
AGGGATCAAACAACTTGGTGAAGATCCATGGTTAAATATCGCGAATGAACTTCCTGTAGGTGCTAAGCTT
ATGGGTACAGTAACTAATATTACTGACTACGGTTGTTTTGTTAAGTTAAAAGAAGGTATCGAAGGTCTTG
TTCATACATCTGAAATGGATTGGACAAACAAAAACGTTAACCCTCATAAAGCTGTATCTATTGGTCAAGA
AATTGAAGTTATCGTACTTGAACTAGATGCTGATAACCACAGAATATCTCTTGGTATCAAGCAATGCAGA
CCTAATCCTTGGAGCGAGTTTGAGAAAAACTACAAACCAGGTGATAAAGTTACTGGTAAGATCAGATCAA
TTACTGAATTTGGTGTGTTTATCGGTCTTGAAGGCGGTATTGATGGTCTTGTACATATTTCAGATGTTGC
ATGGGATAATCCAGCTAAAGCTATCAAAGAGCTTAAGAAAGGTGATGAAGTAGAAGCTGTACTAGTTTCT
GTGAACACTGACCTTGAGAGAATTGCTCTTAGCATGAAGCAACTTTCTGAAGATCCGTTCAAGAACTTCA
TAAATATTCACCCTAAAGGTTCTTTAGTAACAGGTAAAGTAACTAAAGTACAAGATAATGGTGCAGTAGT
TATGCTTGACGAAGATAACAACATCGATGGTTTCATCAGAATTTCTGAAATTTCTGCTGAGCATACAAAA
GATGTTCGTGATGAGTTAAGCGAAGGTCAAGAAGTAGAAGCTAGAATTATTAACATTGACGCTAAGAAGA
GAAGCATTACTCTTTCTATCAAAGCTGTTGATGAAGATAACACTGCTGCAGGCAAGTCTAACTACAAAGT
AGAGCAAATGACTCCTACAACTCTTGGTGACCTAATCAAAGAGCAACTAAATAAGAAGTAA

>lcl|NC_006570.2_cds_YP_169245.1_160 [gene=FTT_0184] [protein=zinc-binding domain-containing protein] [protein_id=YP_169245.1] [location=200305..200766]
ATGTCTAATTATTCAGATCAAGATATTTTTTTATGCAAAAAGCTTATCAGCAGGCATTGTTAGCTTATC
AAGCTGGTGAAGTTCCGATAGGTGCAGTGCTTGTCAGGGATGATCAAATAATTGTACAAAATTTTAACCA
AACTATAGGTTTAAATGATCCAACAGCACATGCAGAAATCTTGGTTTTACGCTCAGCAGCATTAAAGCTA

GGCAACTACAGATTAGTCAATACAAAATTGTATGTTACTTTAGAGCCATGTATAATGTGTTTGGGTGGTT
TGATTCAGGCAAGAGTACCTGAGCTGGTTTATGCTTGTGATGATAGTCGAGTAGGAGCTTTTTCACGTGA
AAAACTTCACCACAATAAGAATATTAACCATAATCTTGGAGTAACAGCTGGAGTAATGGCTGATGAATGT
AGCAAACTGCTAAAGGATTTTTTAAACAAAGAAGAAATTAG

>lcl|NC_006570.2_cds_YP_169246.1_161 [gene=ddlB] [protein=D-alanyl-alanine synthetase A] [protein_id=YP_169246.1] [location=200770..201660]
ATGAAAAACGAAAAAATTGTTGTTTTATATGGTGGCGACTCTCCAGAAAGAGAAGTTTCTTTGAAATCTG
GTAAAGCAGTTTTGGATTCTTTAATAAGCCAAGGTTATGATGCCGTAGGTGTGGATGCTAGTGGTAAAGA
GCTGGTAGCAAAACTTTTAGAATTAAAGCCAGATAAGTGTTTTGTTGCATTACACGGTGAAGATGGTGAG
AACGGTAGGGTGTCAGCATTACTTGAGATGTTAGAAATCAAGCATACAAGCTCATCGATGAAATCATCTG
TAATTACTATGGATAAAATGATCTCTAAAGAAATATTGATGCATCATCGGATGCCTACACCGATGGCAAA
ATTTCTTACTGATAAATTAGTTGCAGAAGATGAGATTAGCTTTCCTGTTGCAGTTAAGCCAAGTAGTGGT
GGTTCAAGTATTGCTACTTTTAAAGTGAAATCTATACAAGAGCTCAAACATGCCTATGAAGAGGCATCGA
AATACGGTGAGGTAATGATTGAACAATGGGTCACAGGTAAAGAAATTACAGTGGCTATTGTCAATGACGA
GGTTTATTCTTCTGTTTGGATTGAGCCACAAAATGAGTTTTATGATTATGAGTCAAAGTATAGTGGTAAA
TCAATCTATCATTCACCGAGTGGTTTATGCGAGCAAAAAGAACTTGAAGTTCGTCAATTAGCTAAAAAAG
CTTACGATCTGCTTGGTTGTAGCGGTCATGCAAGAGTAGACTTTATCTATGATGATAGGGGCAACTTTTA
CATTATGGAGATAAACTCTTCACCAGGAATGACCGATAATAGTCTATCGCCTAAGTCAGCAGCAGCAGAG
GGGGTTGATTTTGATAGTTTTGTGAAGAGAATAATAGAACAGGCGCAATGA

>lcl|NC_006570.2_cds_YP_169247.1_162 [gene=ftsQ] [protein=cell division protein FtsQ] [protein_id=YP_169247.1] [location=201657..202340]
ATGACAAAAATTATCAAGAAGTTTCTAATATTGAGCTTGATACTGTTAGTAATCTTGGGATCGACTATTT
TTGTTGCTGCTAAAACAGATAAGACAGTTTCAAAGATTGATGTTGTATCTAACGATGGGCTTATTTACAT
ATCTAAGCAAGATTTAATCAATAAAATTGCTACGCTTGATAATAAACAATGGTTTGATATTAATATTGCT
AATATTGAGAAATATATTTACTCAATCGATGGTGTTGACTATACATTGGTGAAAAAAGTCTGGCCATCGA
CATTGGTAATATATTTATATGACCATAAGCCTGTAGCTTACTGGAATAATAATCAGATCCTTCTTGATAA
TATGCAAATCATCACTCCAGCAGTTTTTAACTATAATGGTGATTTACCATATATTCAAAGCAAAGATGAT
AGTAGTAAAGACTACATATATCAAACCTATAAAGAGTTAAACAGTATTGCTAAACAAAATCATATGCAAA
TACTCAAAATCTCTTATACTGGTAATCAATTTGGTATTTTACTCTCAGATGATATTGAAGTTATGCTTGG
TTCAGTTAAGCTGAAAAAGCGCTTGGAATTATTTTTTAAATCTTATACAAAAGTTAAAGATTATAAGTCA
GTTAAATATTTCGATATGCGTTATAGTGATGGCTTTGCGGTAAAGTATAAATAG

>lcl|NC_006570.2_cds_YP_169248.1_163 [gene=ftsA] [protein=cell division protein FtsA] [protein_id=YP_169248.1] [location=202442..203704]
ATGGGTTTTGGGAATATTAATTTTTGTGCTGTAGATTTAGGCTCGCACAAAATAACAGTTGCGATTGGTC
AACTTGCTGAGAATAACAGTATAAAGATATTGGGAGTTAGTCAGAAACAGTCAAAAGGGATCAAGCAGGG
TTCTGTAATTAACCTTGAAATCGCTATGGAAACTCTTAATGCTGCTCTTGATGAGGCTAAGAGTATAGCA
GGTGTAGATGTTAAGGAAGTTACTCTAGGTGTTAGTGCACCTAGTATTAGTGGTTTTAACTCATATGGCT
TAGCAGCAGTTGAAAATGGCGAGGTTAGTATTGAAGATTTAGCTATGGCGATTAAAACTGCAAAAGCAGT
TCCAATGTCTGCTGATACAGAGATGTTACATGTTTTACAAAGAGATTATATTGTTGACGGTCAAGCTGGT
GTTACTGAGCCAATAGGGATGTTTGCAGTAAGACTAGAATCTAATGTACATATAATAGTAGCATCATCGC
GCTTATTGCAAAATGTACGTAAATGTGTTTCAAACTGTGGTTACAGTATTAGTAATCTTGTTGTTGAGCA
TTTAGCTGCTAGTAGCGCGACCCTTACTGATAATGAAAAAGAAATGGGTGTTTGCCTTGTCAATATTGGT
GCTGATTCAACAAGCTTTTCAGTATTTGCTGATGGCGGTATTTGTTACACATCAAGTATTAAGACAGGTG
GTGCAAGTATTTCTTCAGATATTTCTAAAGTATTTAGACTTCCTATCGAAGCAGCTGAGAGCTTGAAGTT
ACAATATGGTTATGCTGCAAGTAAATATCTTAAAAACCCTGATGAAAAAATAGATATACCAAACTCATTA
GGCAATGCTAAAAAGAGAATATCATTACAAGACTTATCTTTAGTTATTGAGGCAAGAGTAGAGGAGATAT
TTGAGTCATTATATCGAGAGTTAGATCAACATCGCTTGTTAGAAGTTATATCTTCTGGAATTGTATTCAC
TGGTGGTGGAGCTAAGCTTAAAGGTCTTGCTAGGCTTGCTGAAGATATGTTTAAATTACCAGTGAGAGTC
GGTGGTCCTATTGAGGTCTCTGGAGCTAATGAGGTTGTTCATAATCCTTCGTATGCAACTGTTGTAGGGT
TACTAAAGTATGCAGCTGAGAATAGTGATACAAGCAATCAGCAGAAGATCGAAGAAGATGTGATGGAAAT
AGATGAAAATACAGGTAAATCTAAGAAAAAGATAATCTCATCTGTAAAAGGTTGGTTTTCAAATAATTTT
TAA

>lcl|NC_006570.2_cds_YP_169249.1_164 [gene=ftsZ] [protein=cell division protein FtsZ] [protein_id=YP_169249.1] [location=203748..204893]
ATGTTTGATTTTAACGATTCAATGGTTTCAAATGCCATAATTAAAGTTGTCGGTGTTGGTGGCGGTGGCG
GTAATGCTGTACAACATATGTGTGAAGAAGTTTCTGATGTTGAGTTTTTTGCCCTAAATACAGATGGTCA
GGCATTATCAAAATCAAAAGTTCAAAATATATTACAAATTGGTACAAACCTAACAAAAGGTTTGGGTGCT
GGTGCAAATCCTGAAATTGGTAAGAGAGCTGCAACTGAAGATAGAGCGAAAATCGAGCAACTTTTAGAGG
GTGCTGATATGGTTTTCATCACTGCTGGTATGGGTGGTGGTACAGGTACAGGTGGAGCTCCTGTAGTTGC
AGAAGTTGCAAAAGAGATGGGTATACTTACAGTAGCTGTAGTTACTAAGCCTTTCCCTTTTGAAGGACCA

AGAAGAATGAAAGCAGCAGAGCAAGGTATAGATGAGTTAACAAAGCATGTTGATTCAATAATTACTGTAC
CAAATGAGAAACTTTTAAGTGTACTTGGTAAGGGAGCATCGTTAATAGATGCATTTAATGCGGCAAATGA
TGTTTTGGGTAATGCTGTAAAAGGCGTATCTGAGCTTATCACTAAGCCTGGACTTATTAACGTTGACTTT
GCGGATGTCAGAGCGGTTATGACTAATATGGGTCTAGCAATGATGGGCATGGGTGAAGCTAGTGGCGAAA
ATAGAGCTAGAGAAGCTGCAGAAGCTGCTATCTCAAGCCCACTTTTAGAAGATATTAACCTTGATGGTGC
TAAAGGTGTAATTGTAAATATTACAGCTGGTATGGATATGTCTATCGGTGAATTTGAAGAAGTTGGTGAA
GTGATAAGATCCTTCATCTCTGACGAGGCTATCGTGATAGCTGGTACAGTTATTGATCCAGATATGTCTG
ATTCTATGAAAGTGACTGTTGTTGTTACTGGTATAGAGAAAGTTGCAATGAAAAGAGGCTTTGGTGTAGA
GAAGACATCTAGCCTACAACAAAGTGCTTCAAGTTTTTCAAATAAAACTTCTGCGCCTTTCTTAAGAAAA
GAGACTGAGGTTGTTACTGGCGCTAGTAATGCACCAAAAACTGATTCTGATGATGTAAATAAATCAGATA
TCCCTAGTTTCTTAAGAAGAAGATAA

>lcl|NC_006570.2_cds_YP_169250.1_165 [gene=lpxC] [protein=UDP-3-O-[3-hydroxymyristoyl] N-acetylglucosamine deacetylase] [protein_id=YP_169250.1] [location=204916..205776]
ATGATGAAACAAAAAACTATAGCAAAAGAATTCTCTGTAACCGGTGTTGGTTTACATTCTGGTGTAGATG
TTTCTATGACTGTTAAACCAGCTGATATTGATAGCGGTATAGTATTTCGTCGTGCTGATTTAACTCCAGT
TGTTGATATCAAAGTTACTCCATCTAGTATTAAAGAAGCTATCATGTGTACTCTTTTGACAAAAGATGGC
GATCAAAACCTATCAGTATCAACAATTGAGCACTTAATGTCAGCATTTGCTATGTTTGAGGTTGATAATG
TATTGATTGAGGTTAATGCTCCAGAGTTGCCAGTAATGGATGGTAGTTCATATGAATTTACACAGTTGCT
AAAACAAGTTGGTATCGTTGAGCAGAATTCTGCTAGAAAAGGTATCAAAATCTTAAAACCTGTAAGAGTT
GAGCATGAAGATAAGTTTGCTGAAGTTCTACCTAGTGATACATTAAAATATGAGTTTAAAATTCACTGGG
ATCATCCTGTAATTGCAGCGACAAATGATCATATAGTTTTTGAGTATGATCTTGATGAGTATATTAAGAT
GGTTTCAAAGGCTAGAACATTTGGCTTCTATGAGCAGCTTGCATATTTACATCAAAACAATCTTGCTAAA
GGAGCATCGTTAGATAATGCTGTTGGTGTTACTAATGAAGGTGTCCTTAACGAAGGCGGTTTGCGTTATG
ATGATGAGTTTGTCAGACATAAGCTTTTAGATGCAATTGGTGATTTTTATGTTGGTGGTTATATCTTAGG
TCATTTCAACTGTTTTAAGTCTGGACATACTCTTAATAACAAACTTCTACATGCTGTCTTTGCTGATAAA
GATGCGTGGGAATACATTTAG

>lcl|NC_006570.2_cds_YP_169251.1_166 [gene=dnaX] [protein=DNA polymerase III subunit gamma and tau] [protein_id=YP_169251.1] [location=complement(205790..207430)]
ATGTCATATCAAGCATTAGCAAGAAAATATCGTCCACAATCATTCGCAGAAGTTGCTGGACAACAACATG
CTCTTAACAGTTTAGTACATGCTTTAGAAACTCAAAAAGTTCACCATGCCTACTTATTTACGGGTACGCG
TGGGGTTGGTAAGACAACACTTGGTAGACTCTTAGCAAAATGTCTAAATTGTAAAACTGGTGTTACTGCT
GAGCCTTGCAACAAATGTGAAAACTGTGTAGCAATCAATAATAACAGCTTTATAGATTTGATCGAGATTG
ATGCTGCTTCACGTACAGGTGTTGAAGAGACTAAAGAAATCTTATATAATATCCAATATATGCCTTCACA
AGGACGCTATAAAGTCTATCTAATCGATGAGGTACATATGCTATCTAAGCAAAGTTTCAATGCTTTGCTA
AAGACTCTCGAAGAGCCACCAGAATATGTCAAATTTATCCTAGCTACAACAGATTATCACAAAATCCCTG
TTACGATACTTTCTAGATGTATCCAACTTCATCTCAAACATATCTCACAGGCTGATATCAAAGATCAACT
AAAAATAATTCTAGCTAAAGAGAATATAAATTCAGATGAGCAATCACTTGAATACATAGCTTATCATGCT
AAAGGCAGTTTAAGAGATGCTTTAAGCTTACTTGATCAGGCTATTAGCTTTTGTGGTGGTGAACTTAAGC
AAGCTCAGATCAAACAAATGCTTGGAATTATCGATAGTGAAGAAGTATATAGTATTATCAATGCAATTAC
TGATAATGATCCAAAAGCTATATTGCCAGCCATCAAAAATTTAGCACTTACAGAAAGTAGTGCTGATGTT
GTACTAGATAGAATTGCTGAAATTTGGTTCGCTTGCTGTATATATAGCTTTACCCAATCACTAGATGCTG
TTAATGATATTGATGTTGATATAATCAATAATATTTTGGCAAAAATATCAATCGAACAAGCACATTTTTT
GTATCAACTAACGATAACCGCCAAAAAAGATATTGCTTTAGCACCAAATTTCGAAACTGGTGTAACTATG
GCTATACTTAGACTTATAGCGTTTCAAAAAAAAAATCTAATTGATAAAACCCAAACCTCTAAGTCAAATA
TTAGCCCAATAGTTTCTAAAAATGATATAAATCTGCTTAAAAACACATTTAAGTCAGAACAACCTAAACA
GACTGTAAAAGCTGTTGTAGCACAAAATAATGATTCAACAGCTAGTGAAAATACTCAAGAACAATCATTA
GATAAAAAATGGTTTAATCTGCTTAATAGAATAAAACTGAAAGGCTTCACAAAAACGCTCGCTTTTAATA
GTCACCTTATTAGTGATAATAGTGAAACATTTGTAATCCATCTAAATGAAGATGCCAAAAAAATCCTTGA
GCTTGATCCTCAAAGCATAGCTAAACTTCAAGCAAGTATTAGTGAGTATCTTAACAATCCTAGTTTTAGG
TTAGATATCAAAAACCTAGCTATGGATAAAGTCTCAACTGATCAAAAGTCTCCTGCTGAAATTAAGCGTG
AAAATGCTATAAGTAAAATTCATAATGATGAGAATACAAAGCTTATTAAACAAGCTCTCGCAATTGATAT
AAAGGATCAAAATATAATTTTAACAGATTAG

>lcl|NC_006570.2_cds_YP_169252.1_167 [gene=prfB] [protein=peptide chain release factor 2] [protein_id=YP_169252.1] [location=207703..208680]
ATGGAGCTAGAAGATGGTTCTATTTGGGATAATCCAGAGTATGCACAAAACTTGGGTAAACAAAAAGTAG
AACTTGAGAACGTTGTACACAATTGTGAGTATATCTCAGAAACTCTAGAAACTTTAAGTGAACTGCTTGA
GCTTGGTGAAGAAGATGAATCTTTAATGCAAGAAATTGCTAAAGATACACAAAATGTTGCTAGTGAAATT
GAGAAGCTTGAGTTTCGTCGCATGTTTTCAGGTAAAATGGATGCTAATAACGCCTTTTTGGATATTCAGT
CAGGTTCTGGAGGTACAGAGGCGCAAGATTGGGCAGAGATGCTGATGCGGATGTATATGCGTTGGGCAGA

TAGTCACGGCTTCAAGGTAACCGTTGATGATGTATCAGATGGTGATGTTGCGGGTATAAAAGGCTGCACC
TTAAAAATAGAGGGAGAGTATGCTTATGGTTGGTTGCGTACCGAAACTGGTATTCATAGGTTAGTACGCA
AATCACCTTTTGACTCAAACAGTAAACGTCATACATCTTTTGCATCGGTGTTTATCTCACCTGAGGTTGA
TGATGATATTGATATTGAGATAAATCCTACTGATTTACGAGTCGATACTTATCGTGCATCAGGTGCTGGT
GGTCAGCATGTCAACAAAACAGACTCTGCTGTGAGAATAACGCATATTCCGACAAATATTGTGGTACAAA
GTCAAAGTGATAGATCTCAGCATAAAAATAGAGATAATGCCATGAAACAGCTTAAGTCAAAGCTATATGA
AATGGAGTTACAAAAGCGTAATGCTGAAAAGAATGCACTCGAAGACTCAAAGGCAGATATTGGTTGGGGT
AGTCAGATTCGTTCGTACGTGTTGGATCAGTCACGAATTAAGGATTTAAGAACAGGAGTTGAGAATACTA
ATACACAAGCGGTATTAGATGGTGATTTGGATAAGTTTATTGAGGCTAGCTTAAAAAGTGGGTTATAA

>lcl|NC_006570.2_cds_YP_169253.1_168 [gene=lysU] [protein=lysyl-tRNA synthetase] [protein_id=YP_169253.1] [location=208813..210543]
TTGGGGCTACTAATTGCTAATGTGCAACAAATAATTACAGGGTTTAAAATGAGTAGTAAATTAAAAGATT
TGATAAAAACAACGATAAAAGAATATTTAGAAGCTAATGATATCACAGTTAAAGATGCTATTAGAGGCAA
GATTAGAGAGCAGATTAGTGAGTTTTTAGGCGAGGTAAGCGAGATAAATGAGCAGGTTAAAGAACAAATT
TCAGCGCTTGTGAAAAAGCATGTAACAGAATTACAAGAAAGCTCACAAATTGCTTTAAGAAAAGAAAAGC
TAAAAACTTTAGCTCAGCAAAATAATGGCATAAGCCATCCAAATAGTTTCCGAAGAAATGCTGTTGCTAT
AGAGTTACAGGCTCGCTATGCTGATAAGACTAAGCAAGAATTAGAAGAGCTAGATAATAAACAACAATAT
AGTTTGACAGGTAGAGTAGTTCTACGCCGTGTCATGGGTAAAGCATCTTTTATAACACTACAAGATTATA
CTGGTAGAATCCAAGTTTATCTAAAAAAGAGTGACTTGCCAGATGGGCAATATGAAACTTTTAAGAATCT
ATGTGACTTGGGTGATATAGTTGGTATTACTGGTACTATGTTTAAGACAAATACTGGTGAACTTTCTGTT
GAAGCAAATCACTTTGAGATTTTGACAAAGGCAATTCGTCCTTTGCCTGATAAATTCCATGGTTTGGCAG
ATCAAGAAATGAGATATCGTCAAAGATATGTTGATTTAATTACAAATGAAAAGGCAAGAGAAGTCTTTAA
AGTACGCTCAAAAGTTGTGAACTTTATCCGTAACTATTTTGATAGACTAGATTTTATGGAAGTAGAGACG
CCGATGATGCATGTTTTGCAAGGTGGTGGTGCTGCTAAGCCATTTAAAACTCATCACAATGCTTTGGATA
TGCCTTTATATTTGCGTATTGCTCCAGAACTTTATCTAAAAAGACTAGTTGTTGGTGGTTTTGAGCGAGT
TTATGAGATAAACCGTAATTTTAGAAACGAAGGTGTATCTTCACGCCATAATCCAGAATTTACAATGCTA
GAGTTCTATATGGCGTATGCTGATTATAATGACCTTATGGATCTAACAGAAGATATGCTATCTAAACTTG
TACAGGAAGTAATTGGTAGCGAGATACTTGAGTATGGTGAGTATAAAATCAACTTTGGTGGTAAGTACGA
ACGAATCTCAATGGTTGACTCAATTGTTAAGTATAATGATGATATTACCAAAGAAGATTTAGTAACATTT
GAATCTGCTAAAAAGATTGCTGAGAAACTAAAAATAAAAGTTGAAGCTTACCATGAACTTGGACATTTGA
TAAACGAAATATTTGAAGAAACTGTCGAGCATCAGCTTATTCAACCGACATTTATTACAGATTATCCAGC
TGTAGTTTCACCATTGGCGCGTAGACAAGACGGTAACCCTGAGTTTACCGATAGATTTGAGTTCTTTATT
GGAGCACGTGAAGTTGCTAACGGCTTCTCTGAGCTAAATGATGCTGAAGATCAAGCAGAGCGTTTCAGAA
AACAGGTTGAAGCTGCGGCATCTGGAGATGATGAAGCTATGCCTTATGATAAAGACTATATCCGCGCACT
AGAGTATGGTATGCCTCCTACAGCGGGACAAGGAATCGGTATTGATAGACTGGTAATGTATCTAACAAAC
TCTCAATCAATTAGAGATGTGATTCTATTTCCGCATATGAAGCCTGAGTAA

>lcl|NC_006570.2_cds_YP_169257.1_172 [gene=glnA] [protein=glutamine synthetase] [protein_id=YP_169257.1] [location=complement(214073..215110)]
ATGAAAGTAATAACCGCTGAATACATATGGGTTGATGGTTCAGATCCTGTGCCTCATCTTAGATCTAAAG
CAAGAGTTTTACCATTTAAAGAATTTGAAACTCCAGATGAATTTCCAGAATGGAGTTTTGATGGTTCTTC
AACAAACCAAGCTACAGGAGATAACTCAGATTGTATTCTTAAACCTGTTAACTTTGTAATTGATCCACTA
AGAGATTATGGTTACTTAGTATTATGTGAAGTTTATAATCCAGATGGACAAACTCCGCATGCGACAAATA
ATCGTGCCAAGCTAAGAGCTCTATTAGCTAGTGCTGACTGCCAAGATATGTGGGCAGGTTTCGAACAAGA
GTATACAATGTTCAAAGATGGTAGACCACTTGGATGGCCTACTACAGGATTTCCTGGTCCACAAGGTCCT
TATTATTGTAGTGCTGGTAATAGCAAAGCTTTTGGTCGTGACTTAGTTGAGGCTCATATGCAGGCGTGTC
TAGAGGCGGGAATTTTATTCTATGGTATAAATGCTGAAGTTATGCCAGGGCAATGGGAATTTCAAATTGG
CTATAGAGGTGTTGATGGTGAGGACGCTGGTATATTAAATGTTGCAGACCATACTCACCTTGCTAGATGG
CTACTAGAGAGACTTGGTGAAGAATATGGTATTGATATATCTTTTGATAACAAACCTATCAAAGGCGACT
GGAATGGTGCTGGTCTTCATACTAACTTCTCTACGAGTAAAACTAGAAATCCTCAAACAGGTAGAGAAGC
TATTAAGAAGATATGTGCAGCATTAGAAAAAAATCATAAAAAAGATATCTTAAACTATGGTCACAATCTT
CATGAGAGATTAACAGGTAAACTAGAAACTTCTGATATGAATACTTTCAGTGTTGGAGATGCTGATAGAG
GATGTTCTATTAGAATCCCAAGACCTGTTGCACTTAAGGGCTATGGCTACCTTGAAGATAGAAGACCTGG
CGCAAATGCTGATCCTTATGTTATTGCTATGGCTTTAGCTAATGCTGCTATAAACTAA

>lcl|NC_006570.2_cds_YP_169258.1_173 [gene=holA] [protein=DNA polymerase III subunit delta] [protein_id=YP_169258.1] [location=complement(215230..216207)]
ATGGAATTAAGTTATTTTGAGCTACTCCAAAAAAATGATCTCACCGCCTATAAACTTTTTATCATAACAG
GTGATGAACCTCTACAAAAACACAATACTATTGAAAAAATAACTAATCAGTTTAAAACTAAAAAATTTGA
AATAAGCTATCATGATTTAAGTGAACAAAATATAGATGTTTTGTATAACGAAGTTGATAGTCTTAGCCTA
TTCAGTATTGATAAATTTATTCAATTCAATTTTGATAAGCCCCCACAAAAAAAACTCCAACAAACATTAG
TTGACAAGCTTATAAATGATGATGATAACGTTTACTTGCTTGTTTTTAGTGGTATGAAAAAGCAAAATAC
CTCAGCAAAGTGGTTTCAAAGCTTAGAGCATAAAGCTATTCATATTCGCATTTTTCAACCAAATTTAGAT

AATGCCATAAATATTATAGATTATGAAACACAACAACTCGGCTTAAGTCTGACAAAAGAAGCTACACAGC
TCTTAGCGCTAAAAACCGAGGGAAATTTGATAGCAACAAAACAAATCTTAAAATTACTCTCACGCCAAGA
TAGCCGAGTCTTTGATGAAAATACAATTCGTCCTTTTCTACATGAGCACGCCAATTTTGACGTATTTGAT
CTTTCTGAAGCAATTCTCTCTCAGCATAAAAGCAAAGCACTAAAGATACTTAATAGCATCCTGAACGAAA
ATGATAAGCCTCCTCTTGTACTCTGGGCCCTAAAAAGAGAATTAAGAATACTCTCCCAACTAAAAAACAC
TCAAATAACTTATCATCAAAAAATATTTAAAGATAACAATATTTGGTCAGCTAAACAGAAATTTTATATT
AGTTTAGCGAATAAGCTTAGTCCTGAAAAAATTTCAGCAGGACTGGAAAAATGCCTTGATACTGACCTAT
GTATCAAAGGTGCAAGAAAAGGCAATATTCAGCTTAAACTTAATGAAATTGTTTTTGATATTTTCTAA

>lcl|NC_006570.2_cds_YP_169266.1_179 [gene=FTT_0207c] [protein=ABC transporter permease] [protein_id=YP_169266.1] [location=complement(225323..226165)]
ATGGTCAATGCCATTATTGCAATGAGCTACCTTGTTTACAGGAGGATATTTGTGTTTAGTTATACCTTTA
TGCTATATGCTTTTATAGCAGGAACAATAATAGCGATTATCTGTGGCATTATTAGCTTTTTTGTCATAAT
TAGAAGATTATCATTTGCTTCACATGCTTTAGGACATATTAGTCTGACAGGAGCATCAGGTGCTGTGCTG
CTTAATCTATCAGCGATGTCTGGGCAACTTGCTATAAATCTTATTGCTGGATTACTTATGGGTGCTTTTG
GTGATAAGATTAAGAAAAATGATATTGCCATCGGTATTGTCCTAACTTTTTTCCTAGGTTTAGGAACTTA
CTTTTTATTCTTATATCAAAGTGGCTATTCTGGATCTGTAATGTCTATCCTTGTCGGAGATATACTCACA
GTTAGTCTTGAACAAATATATATTTTGATAGGTTTAGCAATATTTACTATAGTCCTACTCATCATAATTG
CTCGACCATTATTCATATCATCGATAGATCCGATATTTGCTGAGTCAAAAAAAGTATCAAATAAACTTTT
ATCTATCTTACTTTTTATATGTATTGCAATAACTGTATCTATGGCTTGCCAAGTCGTTGGGATTTTATTA
GTATTCTCACTCTTAATTGGCCCTGCTGCTATTGCAACTCAATGGGTTGACGGCTTTTATAAACCTATTG
CTCTAAGTACTTTAATTTCAGTATTAACAGTATGGTCGGGTATAGTAGCCGCCTACTACATTGATGTGCC
AATTAGCTTTTTTATTACAACTATTATCTGTATTTTGTACCTAATAAGTATACTAAAAAATAAGTTTCAA
TAA

>lcl|NC_006570.2_cds_YP_169267.1_180 [gene=FTT_0208c] [protein=ABC transporter ATP-binding protein] [protein_id=YP_169267.1] [location=complement(226107..226784)]
ATGATTAAATGCTCTAACTTGGTGATTGGCTATAATAAACCAATCACAGCTGCGCTAAACCTAGAGGTAC
CAACAAATGCATGGGTTGGTATCGTTGGTAAAAATGGTGTTGGTAAATCAACTTTTTTTAAGACCCTCTT
AGGTAAAATACCAACTATTTCTGGTTTAATAACTATTAATGATAATAAAATAGATGTCGATACTATTAGT
TATATTCCTCAAGAGCGAGAAATAAACTTTGAAGAGAAAACCTCAGGCTATACTCTTGTTAAATATAGTT
ATAAACCAAAGTCATGGGGACTACCATTATTTGATAAAGAATTTAAGGAAAAACTAGAATATCTTATCGC
ACTAACACAAACACAAGATTATATCCATAAGCCTTTTAAAAACCTTTCTGGCGGCCAAAAAAAACGCGTA
TATTTAGTACAAGCTTTGATTAATGAGCCCAAAATACTTCTTTTAGATGAACCGTTATCTGATCTTGACC
CAGATGCAAAGCAACAATTCTTAGCTTGCTTGAAAGAAATTCATAAGAAAGAAAATATCACTTTATTGAT
AATATCACATGATATGAAAGAGATTAGCACTCAACTAGATGCTTTTATTCATTTCAAGGATGGTCAATGC
CATTATTGCAATGAGCTACCTTGTTTACAGGAGGATATTTGTGTTTAG

>lcl|NC_006570.2_cds_YP_169268.1_181 [gene=FTT_0209c] [protein=periplasmic solute binding family protein] [protein_id=YP_169268.1] [location=complement(226786..227703)]
ATGAAAAAATACATACTAATAGCTTTAGCTGCTGTTGTTATTGTAGCTTTGGTAGCTATCAACCTTTTGG
CTGAAAGTGATAAGCCCCAAACAACAAAAGGCAGTCATGACATCTCTGTTGTAGCTGCGGAAAACCAGTA
TGGCAGTATCGCTAAGCTTATTGGTGGTAGTAATGTTAAAGTTACTAATATAATCGATAATGCTGATGGT
GATCCACATACTTTTGTCTCTTCTGTGAAAAATGCTAAGCTTTTAGCCGAAGCAGATGTAATTATCTATA
ATGGTGCTGATTATGACTCATGGATTACGCCTATACTAAAGACTAATAAAAATGCCGAAATCATTAAGGT
ACAAGACCTGATAAACTATCCACAAACCGAAAAACTTGGCATTAATCCACACCTTTGGTATGATCCTGAT
ACATTCCCAGCTTTGGCAGCTAAACTAAAAGATGTTTTTTCTAAGCAAGATTCTGTTGATAATAGCTTAT
TTGAAAAAAATCTTGAAAACTTTAACCATAAATATCAAAAAGTTTATGACCTAGTTAAACAAATAAAGCA
GTCAAGCTCAGGTACTCCTGTAACTGCTACAGAACCATTATTTGGCTATATGGCTAATGCACTAGGACTA
GATATGAAAGGCCTTGCATTTCAATGGGTGATAATGAATGACTCCGAACCTAGCCCTAAAATGATGATTA
ATTATCAGAAACTCTTTAACGATAAACAAGTTAAAGTGCTTTTCTATAACAAACAAGTCACAGATAATGT
TACAAGTGATGTCTTGGAAGTTGCTAAGAAAAATAATATACCAGTAGTTGGTATCACCGAAACTATGCCT
GTAAATGATGATGCTATCAACTGGATGATTGAAACATTACAAGCAACTGCTGTGGCATTGGATAAAGTAC
AAAAATAA

>lcl|NC_006570.2_cds_YP_169274.1_187 [gene=FTT_0219c] [protein=phosphate transport protein] [protein_id=YP_169274.1] [location=complement(235980..236975)]
ATGATTTCATCAGTGCTTATAGCCATTATTGTAATAGCATTGTTTTTTGAGTTTACAAATGGTTTTCATG
ATGCGGCAAATGTTGTTGCAACACCTATTGCAACTAAATCACTAACTCCATATCAGGCAATAGCTTTAGC
AGCATTTTTTAACTTTTTAGGTGCATTTTTTGGTACAGCAGTTGCTGCAACAATATCAAAAGGACTTGTA
GATACTAATGTGGTTACAGATATTGTTTTGATTTCTGCGTTACTTGGAGCGATAAGTTGGAACTTTTTTA
CTTGGAGCTTTGGGATTCCTTCAAGCTCATCACATGCTTTGATAGGTTCTTTAGTTGGCGCAGTGATTAT
CAGCTCAAGCTATCAGAATGTAAGCTATATGACGGTAGTTAATAAAGTTTTGATTCCAATGGTTACCTCA

CCTGTTATTGCATTCTTTTTGGCATTGATAATATGTATCGTGCTGCTAAATATTTTTATGCGATTTTTTA
GAGTTAGAACTACAAATAAATACATAAGAGAAATGCAGGTATTATCTACTAGTTTACTGTCTTTCTCTCA
TGGATCTAATGATGCTCAAAAAACAATGTCAATAATAACACTAGCTTTACTAAGTGCAGGACTTGTTAAG
ACTACTCAAGTCCCTGATTGGGTGATTATACTTTGTGGAGTTGCTATGGGATTAGGGACTTTATCTGGCG
GTAAAAAGATAATTAAAACACTAAGTGCTAAGCTATCAAAACTAGAACCTGTCAATGCTGTTTCTGCTGA
GTTAAGCTCAGGGATACTAGTTTTAGGGGCATCACATATAGGATTACCAGTTAGTACAACTCAGGTTGCA
TCTGGTTCTATTATGGGAGCTGGATATGCTGACGCTGGCGTAAATTGGAAAGTCGTCAAGAAAATGGCAA
TGGCTTGGATTCTGACTATTCCAGCATGTGTATTCGTCACTAGTGCAATATATACCGTAATTTATTATAT
CTTTGGTAGTTTCTAA

>lcl|NC_006570.2_cds_YP_169275.1_188 [gene=FTT_0220c] [protein=hypothetical protein] [protein_id=YP_169275.1] [location=complement(236993..237649)]
ATGCTGCGTAAGCTGGTTAAAAAATTGATTCCGAGTCAAGATAAAATCTTTTTTGATTTAATGATAGAAG
CAACAGAGGCTGTTGAAGACTCGGCTAGAATTCTTGATAAATTAGTCAAAGAAGAAGATAATTTAACTAT
TTCTGAATTATCTGAAGAACTAAGATTAACAAGAACAGTAACAGTTGAAGTAGCTAATAAAATCGATCAT
GAATTAGCTCGCTACTTTGTAACACCTGTTGATAGAGTAGAGATACATAATATCACTACTTTGCTTTTAA
AATTAAATCGAAGAATTGTTAAGATTCATCGATATATGCAGATCTTGATGGAAGAAGAGCGTGGCAATGT
AAATATATACTTGGCTAATAGTGTTGAAACACTACGTAAAATGACAAAAGTTTTAGATGATATGATGAAA
GCTTTTGTTAACGGCGATAATAAAGAGCTCAAAAATCTTTATATAAGGCTTACAGCTTTAGATGAGAATG
TTTTAGAAGAGTTAGGTCATGCGCTTAAGAAATTCTCTCATTATGAAGAAGGTGATGTAATTTTTATCAT
GAAAGTTAAAGATATATATAAGGCTATAGAAAATGCGATATCTACATGTACATCTGTAGCTGAGTCTATT
ATGAGATTATATGTTAAAGAAGTTTAA

>lcl|NC_006570.2_cds_YP_169281.1_194 [gene=orn] [protein=oligoribonuclease] [protein_id=YP_169281.1] [location=complement(243775..244311)]
ATGCAATCAGCAGATAACCTAATCTGGATTGACCTTGAAATGACAGGTCTTGATGTCGATAGTTGCAAAA
TTACCGAAATAGCTGCAATTATTACTGATAAAGATCTAAATATAATCGCTGAAGCAGAGCCAATTGCAAT
CTATCAGCCTGATGAAGTTTTAGCCAATATGAATGAATGGTGTATAAAAACTCATACTGAAACAGGACTT
ACACAAAGAGTCAAAGATAGTAAAATCTCTACAGAAGCTGCCGAGCAACAAATTTTAGAATTTATAAGAA
AGTTTGTCCCATACCAAAGTTCTCCTTTATGTGGTAATTCAATTTGGCAAGATAGAAGATTCCTTGCAAA
GTATATGCCAAACATAGATGAATACTGTCACTATAGAATGCTAGATGTCACTACTCTAAAACTGCTTAAT
CAGTATTGGGGCGATGGCAAAAGCTTTGAGAAAAAAAATACTCACAAGGCATTAGATGATATTCGCGAAT
CAATTGCCGAGCTAAAATTTTATCGACAAAAACTTTTATCAATCTAA

>lcl|NC_006570.2_cds_YP_169282.1_195 [gene=efp] [protein=elongation factor P] [protein_id=YP_169282.1] [location=complement(244314..244883)]
ATGGCTAGTTATAGTACTAATGAGTTTAAAGGCGGCTTAAAAGTTTTAATTGATGGTAACCCAATGGTTA
TCGTTGAGAATGAGTTTGTAAAACCAGGTAAAGGACAAGCATTTAATAGAGTTAAGCTAAAAAACTTGCT
TAATGACAGAGTAGTTGAAAAAACTTTTAAATCTGGTGAATCTGTTGAAGCAGCAGATGTAGAAGAACTA
ACAACTGTATATTCATATTTTGATGGTGATAGCTATGTATTTATGCACCCTGAAACATTTGAACAATATA
TGGTTTCTGAAGAAGCTCTTGGTGAAACAAAAAAATGGCTTAAAGATCAGGATGAATATCAAGTAATCCT
TTTTAATGGTCAGCCTATTTCTATTATTGCGGCGAACTTTGTAAACCTTGAAATTATCGAAACAGATCCT
GGTTTAAAAGGTGACACCGCTGGAACAGGAGGCAAACCTGCAACACTATCAACTGGTGCAGTTGTTAGAG
TTCCTCTTTTTGTACAAACTGGTGAGATTATAAAAGTTGATACAAGAACATCAACTTATGTATCTAGAGT
CAAGGACTAA

>lcl|NC_006570.2_cds_YP_169284.1_197 [gene=htrB] [protein=acyltransferase] [protein_id=YP_169284.1] [location=complement(245343..246266)]
ATGAATAATAACAAGTTTAGTCCTAAAAACTGGGGTATCTGGATTGTCGTTGGTATAATGAACTGCGGCT
CGAAGCTACCTTTATTTACACATAAATATATAGTTTTAACTATTGGTCTTATTATTAAACCTTTTTTAAA
AAGTCGTAATGATATCGCTTATGAGAACCTTAAGATAGCTTTTCCTGAGAAATCTAACAAAGAAATAAAA
AAACTAGTCAAAAAAAGTTATTATTCAATGGTGTTATCTGGAGCTGAAACAACCGCCGCATGGTTTCTCT
CTAAAAAAAGATTTAATAAAATTGAGTTTAAATGGGAAGAAGGCTCTAGGGAAAGATTCGAAAAATATCA
TAATGATCCTGATAAAAAACTAATTATCTTAGGTTTTCACTTTCATTGTATCGAGATCATCGGTAGATAT
ATGGGGCAAGAGTTTCCGCCATTTACCGTAATGTATCAAAAAAATGGTAATGACCTTATAGAAGACTTAA
TTAAAGAATATCGCGAAAAAAGCCTATATAAGTGTCTTGATAGCAAGAACTTTGTTTCTGTTATTAAAAG
CCTAAAAAAAGGCTATACAATGTGGTACGCTCCAGATCAGGACTTTGGTTTAGAAAGCACCGGCTTAGAA
AATTCGGTATTTGCTCCTTTTTTTGGTAAACTATGCTCAACTTTAACTGTAACTCCTTGGCTTGCCCAAA
AAACGGGAGCTGTTGTAATACCTGCATATTATGTTAGAGAAAAGTGTCTAAAAAAATATAAAATAGTCAC
TGGAGAGCCTTTCGAGTTTACAGGTGACGCCTATAAAGATGCTGAAATGACAAATAAATTCCTAGAAGAT
GCGGTAAGAAAATATCCAGAACAATATCTATGGCAGCATCGTCGCTATAGAACCAGACCAAATGGTGAGC
CACAAATATACTAA

>lcl|NC_006570.2_cds_YP_169286.1_199 [gene=yidC] [protein=inner-membrane protein] [protein_id=YP_169286.1] [location=complement(247233..248888)]
ATGAAAGCTAATCATATAAGAATATTACTGTTAGTCACAATAGCAATAATGTTTATCTCGTTGATGGGAA
AATGGGAGCAAACATTCCCTGCTGACAATACTAAACAGCAAACATCAGCCACACAAAATAATAGTCACTA
CGACAATGCAGATTCAAGCACAAATACTGATGTAACTACAACTGATGCAAAAAGCTCTTTAGCTAAAGAG
ACTAATTTTTCTAAGTATGATAATGCTAAAAGTATCACAATTAATACTGGAGTTTTTAAAGATGTAAAAG
TTAGTCTACTAGATGGTGCAATTATATCAGCATCTTTAAAAGACTACAGTATCAGTCTTGATGATAAGAC
TCCGATGAGTCTACTTACAGATAAATCAGGTTCTGAATATATTGCTAAGAGCACAATAGTTGTAAATAAG
CAACCTATTAGTGTTAATTTTGAAGACCAAGGCATCAAAATAGAGAATGGCAAACAAATCCTAACGCTAA
CTGGTAGTGCTGATGGCTTGCAAATAACTAGAACATATACCTTTGATGATACTAAATACAATATATCAGT
GTCACAAAACATAAAAAATACAACATCTGCACCAGTTAATGTAATTGTTGATGATTCATTTGCTAGAGAT
TTTGATCCTGCTGGTGATAGCTTTAGCTTATTAAATGCTCATAGCTATACTTTTACAGGTGTTGCATACT
CTACAGCTAAAGATAGCTTTAGAAAAGAATCCTTTAAAGATATATCAAAAACTAATGGTCAGCCAACAGT
AATAAATAGTGATGGTCAAGGTTGGGTTGCATTTTTACAACATTACTTTGTAAGTGCATGGATACCACAA
TCTACTAATGCAAAAATTTATTATAAAAACCTAAATGGTGATGTATTCGAAGCTGGAGCATTTACAGGTG
CGACAATAGCACCAAACCAGTCAGAAAATATTTCTTCTATTTTATATACTGGTCCAATAATAAAAGCCAA
TCTAGTAGACTTAGCACCAAATCTAGAAAAAACACTTGACTATGGAATGTTGTCTTTCTTCTCAGAAATA
ATTTTCTGGGTTATGAATCACATACATTCGCTAGTTGGTAACTGGGGCTTAGCAATTATTTTAGTAACAT
GTTTGATTAAACTTATTTTCTACCCTCTTTCTGCGAAGAGCTATCGCTCTATGGCAAAAATGAGAATGCT
ACAGCCTAGAATTAAGCGTCTGCAAGAAACATACAAAGATGATCGTCAAGCTCTTGGTAAGAAGATGATG
GAGTTGTACAAAGAAGAGAAAGTAAACCCACTTAGTGGCTGTTTGCCTATGCTTATACAGATTCCAATTT
TCATCTCATTATACTGGGTATTACTCGAATCCGTAGAGCTAAGACAAGCACCTTTTATATTCTGGATTCA
TGATTTATCGATGAAAGATCCATATTTTGTACTTCCAGTGCTAATGGGATTATCAATGTTTTTACAACAA
AAATTATCACCAGCTCCAGCTGATCCTATGCAGGCAAAAGTAATGATGTTTTTACCAGTAATATTTACAT
TCCTGTTCGCATCGTTCCCATCTGGTCTAGTATTATATTGGCTGACGAATAACCTAATCAGTATTTCACA
ACAGTGGATTATTACAAGACACTATCAAGCCACTCATAAAAAATAA

>lcl|NC_006570.2_cds_YP_169288.1_201 [gene=rnpA] [protein=ribonuclease P protein component] [protein_id=YP_169288.1] [location=complement(249121..249522)]
GTGGTAGAAAAGTTATCAGAAACAGAAGAGCTAAAGGTAGAGCAAAGCTTGCTGCATAATTTTTGCCTGA
CAAAGCAAAATATACTTGATAAAAGTGAGATCCAACAAGCTTTTGATAGCGTAGAGAATAAATTAAGCAC
GCTACACTTCACATTCCTTTTAGCTAAAAGAAATATAAAAGACCCTGGCTTGTGTGTGATCTTAACAAAA
AAAAACATAAAAAAAGCTACCAAAAGAAACCTTTGTAGAAGAATAATTAAAGAATCATTCCGCTTACATA
AGGATCTCTTAGATCATAAAAGCTTGATAGTGCTAAGTAAGAAAACAGCAGCCCAAGCAACTAAGGAAGA
GTTGTGGCAATCTATAACAGAATTCGAATGTTTTTTAAAAAAATTACACTAA

>lcl|NC_006570.2_cds_YP_169289.1_202 [gene=rpmH] [protein=50S ribosomal protein L34] [protein_id=YP_169289.1] [location=complement(249464..249598)]
ATGAAAAGAACATTCCAACCTTCAAACTTAAAAAGAAAGAGAACTCATGGCTTCCGTGCTCGTATGAAAA
CTTTAAGTGGTAGAAAAGTTATCAGAAACAGAAGAGCTAAAGGTAGAGCAAAGCTTGCTGCATAA

>lcl|NC_006570.2_cds_YP_169292.1_205 [gene=murC] [protein=UDP-N-acetylmuramate--L-alanine ligase] [protein_id=YP_169292.1] [location=251115..252470]
TTGAACAAAAAAAATATTATTTCTTGGAGTTGGCGGTATTGGAGTATCAGCTTTAGCTATCGCAGCTAAAA
GGCTTGGTGCACATGTTGCTGGTTATGATAGTGTCGCAAATAAACTAACAGCAAAACTTGAAGCTTTAGG
AATAGTAATATTTACTAGTCCTAATGGTGTTGATGTTGCAAATTTTGATATTGTTGTATACTCAAGCGCT
ATACTTAGTAGTCATCCGCTGTTATCACAAGCTCGAAGTCTAGGGATACAATGCTTACAAAGGGCAATGT
TCCTAGCTGTTTTAATGAAAGACTTTAGTTATAGTATTGCGATTACTGGTACACATGGTAAAACAACTAC
CTCAAGTGTTTTGGCAACATTACTTTGTCAGCTAGATAAGTATAGTAGTTTTATTGTTGGTGGAGTTGTT
AAATATGCAGATTCGAATATACAGGTTAATGGTACTGATAAATTAGTTATCGAAGCTGATGAAAGTGATG
CTTCATTTCTTTTTCTAAGCCCTCAGGTAGTGATAATTACTAATATTGATTTGGATCATATGGCAACTTA
TAACAATAGTTATCAAACCCTACTTGAGAACTTCACAGATTTTGTTAGTAAAGAAAGTGTAAAGAGTATT
TATCTATGTGTTGATGATCAAGGTTGTAGAGATTTATTAGCTAAATATAATCAATCAGACAAAAATGTTA
CATCCTATGGTTTTTCTATTAATGCTGATGTGCAAATATATGATTATCATATTATTGATGAAATTACTCA
TTTTAAAATTAGATATAAGGACGATGATTTAAGTTTTAAGCTTCAGCTACCAGGTAGATATAATGTCCAA
AACGCCACGGCGTGCATTATTGCTTGTCTTGATTTGGGCTTTAAATATGAGGATATTCGTAATGCACTAA
TTAAAGTTACTGGAGTAGCGAGGCGATTTGATCTCTACACTAAGGTAATCTCAGGACATCAAGTAACGGT
TATTGATGATTATGGTCATCATCCTGTTGAGGTCGCGAATAGTATAAGTGCAGTTAGAGATAGATATCCA
AATAAAAAGATTATTCATGTTTTTCAGCCACATCGATATACACGTAACAGAGATTTGATAAAAGATTGGC
CAAAGGCTCTTTCACTGGCAGATCAACTAATTCTATTGCCTACATACTCAGCAGATGAGCAGATTATCAA
GGGCGCTGAAAGTCAAGATATTGTTAAAGGATTATCTGGATATCTACTTGCTGATGGTTTTGATCATGCA
ATATATTTTTTAGAAAAGCTTGCTAATGAAAATACCGTTATATTGATTCAAGGTGCTGGAGACGTCACAA
ATTTGGTGGAGATATTGAGTGAATAG

>lcl|NC_006570.2_cds_YP_169295.1_208 [gene=FTT_0242] [protein=hypothetical protein] [protein_id=YP_169295.1] [location=254156..254719]
ATGTTACAGCGTGGATTATATACCTTAAAGGAGATGTTTAAACATCGTAAAAAACTCTACGGATTATCAC
AAGGATACCGTATCGATAGTATTATAAACCTATCAGCTAAATTGCTAAGGCAACAAGGAATTAAATATTT
AGCATTGGACTTTGATGGCGTTCTAGCAAGTCATGGTAAGTCCGAAATGCATCCGGAGGTTATGCTTTGG
TTTAAGGATTTTGTTACTGAATTTCCCGCGGACAGAATATTTATCCTCTCAAATAAGCCTACAGAAGCTA
GACTTAAGTATTTTAATGCTAATTTTCCTAAAATTAGGTTTATTGCTGGGGTCGCAAAAAAACCATATCC
AGATGGACTTAATAAAATCATCCAACTAGTAGGTTGCCAAGCAAAGGAGTTAGCACTTGTTGATGATAGA
TTGCTTACGGGCTGTCTAGCATGTTTAATTGCTGGTTGCTACCCAATACTTATCACTAATCCTTATATAG
ATACAGATAACTATACTAAAGAGGAAAGATTCTTTAAATTTTTGCGCTATAGTGAACAAAAAATCTTTCT
ATAA

>lcl|NC_006570.2_cds_YP_169311.1_222 [gene=hemC] [protein=porphobilinogen deaminase] [protein_id=YP_169311.1] [location=276043..276945]
ATGAAACAAATAACTATAGCTAGTCGAGAAAGTAAACTAGCATTATGGCAGACAAATTTTGTCAAAAATC
GTATCCAATTAGAGCTAAACATTCCTTGTGAAATAAGTACTATGAAAACACAGGGAGATATTATTTTGGA
TCAACCGCTAAACAAAATTGGTGGTAAAGCACTATTTATGAAAGAGCTAGAAGTAGCAATGCTTAGTAAT
AAGGCTGATATTGCTGTACATTCTCTTAAAGATGTTCCTTACCAATTACCGCAAGGTTTTTGTTTAGCAG
GTTTTATGCCAAGAGAAGATCCTCGAGATGCTTTTGTATCAAATAAATATAATTCTATTGATGATTTACC
TAAAGGAGCTGTTGTTGGAACATCTAGTCTACGGCGAAAAGCGCAGCTTTTGCACTATAGAGATGATCTT
GAGATTAGAGATTTAAGAGGCAATATTCAAACTAGACTGTCTAAGCTTGATAATGGTGATTATGATGCGA
TTATTTTAGCTAGTGCTGGACTTATTCGCTTAGAGTTAGTTGAGAGAATAACTCAGTTTATCCCTGTAGA
AATATCTTTACCAGCGGTAGGTCAAGGTATAGTAGTTATAGAGGCATTAGAGAGAGATAATGACCTTTTA
GAAAAAATACAAAAATTAAATTGTAGAGAAAGTTCTCGTGTAGCAACAGCTGAGAGAGCTTTTAATCAAG
AATTAAAAGGTGGCTGTCATGTTGCAATAGGTGCTTATGCAGAATTAGATAATAATCAGATAACCTTGAT
GGCAATGGTTGCAAGTAGTGATGGTAAGAAAATTCTCAAGCGAAAGATGATTGGGGATGATCCTACTAAA
CTTGGTAAATTGTTAGCTCAAGAGATGATAGCACTAGGTGCATATAAAATATTGGAGAGTTAA

>lcl|NC_006570.2_cds_YP_169317.1_227 [gene=FTT_0265] [protein=ABC transporter permease] [protein_id=YP_169317.1] [location=278751..280550]
ATGAAAAGGTTATATAGCACTAATATGTCAGCAAAGGTTTCTCGCACAAAGTGGGATATTTTAGCTTTAT
CAATTATACTTCTGATATTAAGTATCTTTGTATGGTCAACCTCTGACCTTGGCGGTTCTATTGATTATAA
AAGTCAGGCTAGTGTTCTTAAATACTCAGATATTTCGTTAAGTCTTTGGCTCTTGCCTTACTATACAGCG
GAAACAACAATTAGAATGTTTATAGGTTTAGTTATTTCACTACTTATAACATTTATATTTGGAACATGGG
CAGCAAAAAAATAAAAGAGCAGAAAGTATAATCATACCGCTTGTTGATATTTTACAGTCAATACCAGTTCT
GGGTTTCTTTGCAATTACAGTAACAGGATTTTTAGTTATATTTCCAACCTCATTATGGGGAGCCCAAGCT
GCGGTAATTTTTGGTATTATAACAGCACAAGCTTGGAATATGATTTTAAGTTTCTATCAATCCTTAAAGA
CAGTACCAAAAGAGCTAAGAGAAGCTGCTGATATGTACCAACTCTCAGCTTGGCAGAAATTCTGGAAACT
AGAAGTACCATTTGCAATGCCTGGTTTAGTTTGGAATACGATGATGTCAATGTCAGGAAGCTGGTTTATG
ATAGTAGCTTCTGAAACAATTATGGTTAATTTCAGTGCATCACAATCAATACCAATAAATTTACCTGGTA
TAGGTTCTTTTATTGATGCGGCTAATAATGCACAGAACTTTACAGCAGTTGGTGCTGCAATAGTTACTAT
GTTGGTTACGATTATCTTATATGATCAACTTTTATTTAGACCATTAGTCTCTTGGTCTGAGAAGTTTGTT
ATCGGTGATAATCCTTCAGAGACGCATAGTAAATCATGGTTTTTAACTATTTTGCAAAAGGCATATGTTG
TCAAAATTTTCACGACATTTTTAGCTAAATGCTCTAATAAGATTGTGAATATAAAGTTTTTTAAGAAAAA
CCTTGATAAAGCTTATAATCAAAAAGTTCATAGAAAAGCCGATAAGCATGAAAGTGTAACCAAGAGAATA
CTTTGGAATGTTATTATTATCTTAATGATATTAGCATTGCTATATTTTGTCTATCAAACAGTTTATGCCA
AAGATAAAGACATAGGTATTGAAGAAACATTTAAAGTATTTGTATATGGACTATTTACAGGAGTCAGGGT
TGCGGTACTGATATTGATAACTTCACTAATATGGGTTCCGATAGGAATATGGATTGGTTTAAGACCTAAG
ATAGCTCAAAAAGTTCAGCCATATGCACAAATGGCAGCAGCTTTTCCTGTCAACGTACTATACGGTGTAT
TTGGAACACTAGTGGTAATGTTTAACCTTAATTTTAATATATGGTGTATCCTACTTATGGCGCTTGGTAC
ACAGTGGTATATTTTATTTAATGTCATTGCGGGAGCTTCAGCTATACCAGAAGAGTTAAAGTTAGCTGCT
GAAAATATGCAACTAAAGGGTATTATCAAGCTTAAAAAGTTCCTTGTGCCTGCGGTGATGCCATATTACG
TCACCGGTGCTATAACAGCAGCGGGAGGTTCATGGAATGCAAGTATTATTAGCGAATACATAAACTGGGG
GAAAGATTCTGTAATTCAAGCTTCAGGAATAGGTGACTATATTACAAAATATACTAATATGCCAGGAGAT
CATACTTCAAATGTCCTTTTAGGTGTGATAGTGATGTGTATTTTAGTTGTAGCCTCTAACAAACTGTTCT
GGCGCAGATTATATAACTATGCGGAAAATCGTTTTAGTATGAATATGTAA

>lcl|NC_006570.2_cds_YP_169318.1_228 [gene=FTT_0266] [protein=ABC transporter ATP-binding protein] [protein_id=YP_169318.1] [location=280569..281885]
ATGACTAAGAAAATATTTACAGTAGAAAAAGTTAATAAAGAGTTTAATATCAAAGGTGGCCATTCTCTTA
AAGTCTTGGATAATATAAATTTTACTCTTTATGAAGGTGAAGTTGTTGCTCTATTAGGTAAGTCCGGCTC
AGGTAAGTCAACTTTATTAAGAATAATTGCAGGCCTTTTAAGTCCAACTAATGGTGATGTGCTCTATCGT
GGTAAAAAAGTCTCTGCACCTGTACCAGATATTTCTATGGTTTTTCAGAGTTTTGCACTGATGCCATGGT
TAACAGTTTTACAAAATGTGGAGCTTGGACTTGAGGCACGTAAGATAAGCTTAGAAGAGCGTCGACAAAG

AGCTCTAAAGGCTATCGATATGGTTGGTCTAGATGGTTTTGAGAATGCTTATCCAAAAGAACTGTCTGGT
GGTATGAAACAGCGTGTCGGTTTTGCTAGAGCTCTTGTACTTGAGCCGGACGTTTTATTGATGGATGAGC
CTTTTTCTGCACTTGATATTCTAACAGCGGAGAATTTAAGAGAAGACTTACTTGATCTATGGGAAAATAA
TGATGCTATGAAAGGTATTTTGTATGTGACTCATAGTATCGAAGAGGCAGTTTTGACAGCAGATAGGATA
ATTATATTTGGTAGTAATCCAGGATTTATCCGTGGTGAGCTAAAGATAAATATCCCACATCCAAGAAGCT
CTCAGGACCCTGTAGTTGCTGATCTTGTAGATCAGGTTTATCGTATGATGACTACAGCTCAGACCAAAGA
GCTAACAGAACGTATGAATAAGAAAACTGCAATGACTATCGGCTATCGTCTGCCGGATGTTGATATTTCT
GAGATGAATGGTCTTTTAGATGAGATGGCAGAGATTAAGAATGTTGAGGCGGTTGATTTGCCACAACTTG
CTGATGATTTACATCTTGATATTAATGATTTATTCCCAATCATTGAGATTTTATCAATCTTAAGATTTGC
TGAAGTTTCTGATGGTGATATCAAAATGACAGCGATGGGACGTAAGTTTATTGATTCAAACATTGATGAG
AGAAAATTTATTTTTGGACGCTTATTTTTGAAATATATACCATTAGCGCGTCATGTGGTTAAGGTTCTTC
GCGAAAGAGAAAGTCAGTCAGCTCCTAGAAGTAGGTTTTTAGCAGAGTTGGATGATTATTATCCGATGGA
TGTCGCTGAGCAAGTTTTTGACACATTTATTGACTGGGCTCGTTATGCTGAACTTATCTACTATGATGCA
AATACCGGAGTAATATCTTTAGATGAAAATGCTGCGGAATATATCAAGAAAATGTAG

>lcl|NC_006570.2_cds_YP_169322.1_232 [gene=lolB] [protein=lipoprotein releasing system outer membrane lipoprotein] [protein_id=YP_169322.1] [location=285583..286218]
ATGCTGAACACTATGTCAAAGTTAAAAATAGATACAAAGCGCAGATTTAGTTTACTTATTGCTTTAGTAC
TAATTATCTCATTATCATCTTGTGCTACAACGCAAACTAATGTTACAACTAAAACAGTTTTTAATCAAGA
GACTACTTACCATAACTTACTTAAGCTTAAAAAATGGCAAGCCAATGGTGTTATTGGTATTATCTATGAT
AATCAGGCTGAATCTGCTAATTATACATACTTACAAGATGGTGATAATTTTAGTATCAAACTTTATGGTC
CATTAGGAATAGGTAGCATTGAAATAAAAGGCGATACGAACAGTGTTTTACTAGCGAATAGTAAAGGACA
AAAGCTAACAGCAAAAGACGCTAAAACTTTGATGTTAGAGCAATTAGGCTGGTATGTGCCAGTAGAGGGT
CTTAAATACTGGATAAAAGCGATAGCAATACCAAATATTAGGCAAACATCCGAACTAAACACCAATAATC
TTTTAAGTAAACTATCACAAAATGGTTGGAGTATTAGTTATAGTAATTATCAACTAGTTGATTCTAAATA
TCCCTTACCAACAAAGATTAGGATGTCTAGAGATAATCTAACTCTAAAAATCGTTATAAAATCATGGCAA
ATATAA

>lcl|NC_006570.2_cds_YP_169323.1_233 [gene=ipk] [protein=4-diphosphocytidyl-2C-methyl-D-erythritol kinase] [protein_id=YP_169323.1] [location=286206..287033]
ATGGCAAATATAAAAGCTAAAAAATACTATAGTTATGCAAAGATAAATCTATTTTTGCATATATTAAACA
AACGTCCAGATGGTTACCATAATTTACAGACTTGGTTTACCTTTTTGGACTTAAAAGATCAACTAACTTT
TAGTTTTAATAATTCACGAGAAATTAATATTTCGAGTAATATTAGTATTGCTGCAAAACAAGATAATTTA
GTATATAAGGCTATCAAAAAATTTCAGCAAAGCTATAGAGTGCAAGATATAGGTGTTGATATTGAAATTA
AGAAAAAATATTCCAATGGGAGCAGGACTTGGCGGTGGAAGCTCAAATGCTGCTACTACACTTATAGCTTT
ACGTGATTATTATTTGCCGCAGTTGTCAAATGAGGAGATGATTCCATTAGCGGCAAAACTTGGTGCCGAT
GTGCCGATATTTGTTTATGGTAAATCTGCGTGGGCAGAAGGTATAGGTGAGATATTATACCATAAGGATT
TTAGTCCGCAGTATGCGCTATTGATTAAACCGGATATCCATATTAGTACAAAAGAGTTTTTTACAAGTGA
GGATTTAATTAAGTCATCGGTTCTAATATCTAAAGATTTAGGCTTTGATAAGAGCATTATGCATAACGAT
TTTGAGAATGTTTTTTATGCAAAATATCCAGAGTTTAGTCAATATCTAAAAGAGTTAGATAGTGATTTTA
GAATGACAGGTACAGGTTCTTGTTTTTATTTACTCTCAGCAGATAAAAAATAAACTTGAGCAACTTGCAAG
AAAAATTAATAAACCTCTTGACAAATGGCTAGTCAAAACATTAAACTATGTCTACTAA

>lcl|NC_006570.2_cds_YP_169328.1_237 [gene=cydB] [protein=cytochrome d terminal oxidase polypeptide subunit II] [protein_id=YP_169328.1] [location=complement(290541..291734)]
ATGTTATTAGACGTGTTACAAATTATCTCTTGGCTAGTTGTAGGAGTGCTAATATTTTTGGTAGCCGCTA
CAGTTGGTTTTGATTTTGGGGTAGGAATCCTTGCAAAATTCGTTGGTAAAGACGATTACGAGAAAAGAGC
TATCATAAATACCGTAGGACCTACATGGGATGGTAGCCAAGTTTGGTTTGTCACCGCTGGTGGTGCTATA
TTTGCAATCTGGCCTCAAGTATATGCTACTAGCTTCTCAGGATTATATATTGCAATTTTAGTAGTACTAT
GGGGACTTTTCCTTAGACCTCCTGCTTTTGAGTATAGAAAGAAAATAGACAATCCTAAGTGGCGTAATTT
CTGGGATTGGATGCTTGTACTTGGAAGTATAATCCCTATAGTTGTGATGGGTGTAGCTGTTGGTAATCTT
TTTTTAGGTTTTCCAATCTCTTATGATGATACTGCCAGACTGATTTATGGTACAGTAACAAATGGTCAAT
ATCAGTCGATGTGGATCACATTACTACATTTACTAACACCATTTGCACTATTATTTGGTATATTTGCATT
AAATATGGCACTAATGCATGGATCTGCTTATGCTAAGCTTAGAACTAGTGGTATTTTACGTGATAGGTTC
AGAAAGATTACTAATGCAACAGCAAGTGTATATATGGTTTTATTCATAATCGCTGCAATTTGGATAACTT
TTATACCTGGATATCAGTTTACTCCAGATGCTAGCTTAGCTCATCTATCAGATGCTTTAAATCATGCTTT
CACATCAGGTAAAGTAACTACTGACTATTCATGGTACTATAATTTCAACCATGCACATATTTGGATGTGG
TTTGCACCAGTACTAGCTTTTGTTGGCGCTATATTTGTTATTAGATTTAACAATCAAGACAGAGATGGCG
CTGCTTTCTTAGCTAGTATGGCATCTTTACTTGGTGCTGTTTTAACCGTTGGCTTTACTTTATTCCCATT
TATTATGGTATCCAATATTGGTGACTATATGTATAGTCTAACAGTCTACAACTCAAGTAGTAGCCAAACA
TCACTTATAGGTATATTATGCGCAGCTGTTATTATTCTTCCGATCATATTTAGCTATACATTCTTTGTCT
ATAAGAAAATGTGGGCTAATGGACGAAGAATATCAGCTGAAGAGATTAAAGCTAATTCACACGAAATGTA

CTAA

>lcl|NC_006570.2_cds_YP_169329.1_238 [gene=cydA] [protein=cytochrome d terminal oxidase polypeptide subunit I] [protein_id=YP_169329.1] [location=complement(291745..293523)]
TTGAGCTATAAGGAGAAAATAAATATGTTACCAACGCTCATGTCTGTTGACTTAGCAAGATTACAGTTTG
GTTTAACAGCATCATTTCACTTTTTATTTGTTCCTCTAACTTTAGGTCTAACGTGGATCTTATTCACAAT
GGAGCTAATGTACATAAGAACAGGTAAAGAAGTTTATAAAGATATGGTGAAGTTTTGGGGTAAATTACTC
GGAATAAACTTTGCACTAGGGATTATTACTGGTTTGACCATGGAATTTGAATTTGGTACTAACTGGTCAT
ACTACTCTCAATCAGTAGGTGATATTTTTGGTACACCACTAGCTATTGAAGGTTTAGCAGCATTTATGCT
TGAGTCTACATTTGCTGGTTTATTTTTCTTTGGTTGGGATAAACTTACAAAAAAACAACACCTACTTTCA
ACTTTTTGTTTAGCAATTGGCTCAAGTTTCTCAGCTTTACTTATCTTAGTTGCAAACGGCTACATGCAGC
ATCCAGTCGGCTCTGAATTTGTTGCTTCAACTATGAGAATGGAGACTGTTAGCTTATTAGATCTATTCCT
TAACCATACAGCCCAAACAAATTTTGGCCATGTGATGACTGCAGGATATACTACTGCTGCAATCTTTGTA
ATCGGGATAAGTGCATTTTATCTGATTAGAGGTCGTGATATAGCATTTGCTAAGAGATCTATCGCGATTG
GCTTAGGTTTTGGTTTAATTACATGTATAGTGGCGATTATCTTTGGCGATGCAAATGGTGTAGATGCTTT
TAGAGTACAACCACTAAAAATGGCTGCTATCGAAGCAGAATGGGATACTTCTAAAGCTCCAGCAGCATTC
AATGCAGTTGCGCTACCGAGTCAAAAAGAACAAAAAAATAATTTTGATGTTCCTATCCCTGCCGTATTAG
GTTTAATTGCAACTCACTCAACAGATACAGAAATTCCAGGAATCAAAGCTATACTCTATGGTAAAAAGAC
AGCTGATGGTACAAGAGATCCAAACCTTGCATACTACAGAGATATCAAAACAGGTCAAACTGCTGATGTT
TCACCTGAAGATGCTGCTGCTGATCCAAGCAAATATGAAAAAGTACCTTCTGCTTTAGTTATGATCAAAG
AAGGTGGTCTTGCATATGCTGACCTTCTAAAATGGCGTGAGACAGGACATAGTGGTGATACTCCTGATAG
TAGCTACACTAACTACAACAACCCTACTTATCAGAAGTACATGGGCTTTGGTAAAATGCTAGTACAAGCT
GCTCAAGAAAAATATGGTGTGGCTGATTCAGCAACAATTGCCAAAGCTGCAAATGATCCAGAATTAGTAA
GAGCTGTTGCTACTAATATGGTTCCTGATGTTGCTAGTGTTTTCTGGAGCTTTAGGATAATGGTATTCAT
AGGATTCTTTATGTTTACCTTAATAGTTGTTGGGCTAATTCTACTTGCTAGAAATGCTCTTACTAGCAAT
GCATTTAGTAGATTTATCCTAAGGGTTATGATTTGGTCAATACCATTACCTTATATCGCATGTATTGCTG
GCTGGTATGTAACTGAACATGGTCGTCAACCTTGGACTGTTTATGACCAACTACCTACAAGTATTAGTTC
TTCAGCATTAACTGCTGCTGACGTTGGAACATCAATGGCGATATTCTTCTTAATAGATACTGCGCTATTT
GCTGTAATGGTATTTTTAATGTTCAAATATGCAAGACTTGGTCCTAGCTCTCTAGGTACTGGCAAATATT
ATTTTGAACAAAACAAAAAAAGTAAATAA

>lcl|NC_006570.2_cds_YP_169348.1_258 [gene=valS] [protein=valyl-tRNA synthetase] [protein_id=YP_169348.1] [location=312388..315147]
ATGACTCAAGAAATAAATAAAAATTATAATCCAAAAGAAATCGAACAAGCAAATTACCAAAATTGGGAAG
CTTCAGGCAAATTTGCATGTGGTAATACTGACTCGAAAGATACATATACAATCATGTTACCACCTCCAAA
TGTAACAGGTACTTTGCATATGGGACATGGTTTCCAGATGTCATTGATGGATATTTTAATCCGTTATAAT
CGTATGTCAGGCAAAGATACTCTATGGCAACCTGGTACTGATCATGCGGGTATTGCTACACAAATGGTTG
TAGAGAGACAGTTAAATGCTCAAGGTATCTCAAGACATGATTTAGGGCGAGAGAACTTTGTAAGCAAGGT
TTGGGAATGGAAAGAGCTATCAGGTGGCACTATCACATCACAGATGCGTAGAATAGGTGCTTCTCCAGAC
TGGGATCGTGAGAGATTTACAATGGATAAGGGTCTATCTGATGCGGTTAAGAAGTGCTTTATTAAGTTAT
ATGAAGATGGTTTAGCGTATCGTGGCGAGAGATTGGTAAATTGGGATCCTAAGTTAAAAACAGCGGTTTC
AGATCTTGAGGTAGCTCAAGTAGATAAGCAAGGTTCACTTTGGCACTTTATATACCCAGTAGCTGATAGT
GATGAGAAAATTATAATCGCAACAACTCGTCCTGAGACAATGCTAGGTGATATGGCTGTTGCAGTGCATC
CAGAAGATGAAAGATATACTCACTTAGTTGGTAAGATGATAAATCTACCACTTACAGATAGACAAATTCC
AATTATCGCTGATGATTATGTCGAAAAAGACTTTGGAACAGGTTGTGTCAAGATCACTCCTGCTCATGAC
TTTAATGACTATGAAATGGGTAAAAGACACAATTTACCTATGCTAAATATTCTAACTGATGATGCGACAT
TAAATACAAATGTACCATCAAAATATCAAGGGTTAGATAGATTTGAAGCACGTAAGCAAGTAGTTGCCGA
TATGGAGGCTTTAGGTCTTTTAGATAAGATAGAGCCACATGCGCTAAAAGTACCAACTGGAGATAGAACA
GGAGAAATTCTAGAGCCATATCTAACTAAACAATGGTTTGTCAAAGCTGATGTGCTAGCAAAACCAGCGA
TTGAAGCAGTTGAAAAGGGCGATGTAAGATTTGTTCCGGATAATTGGAAAAATACTTATTTTGCCTGGAT
GAGAGATATTCAAGATTGGTGTGTATCACGTCAATTATGGTGGGGTCATAGAATTCCAGCTTGGTATGAT
GAAGCAGGTAATGCTTATGTCGGCGAAGATGAGGCAGATGTTAGAGCTAAATATAATCTAGCTGATGATA
TCGCTATTAAACAAGATGAAGATGTATTTGATACATGGTTCTCATCGGCATTATGGCCATTTAGTACATT
AGGCTGGCCTGAGCAGACTCCTGAGCTGGCAAAATACTATCCAACAAGCGTACTTGTAACTGGTTTTGAT
ATTATCTTCTTCTGGGTTGCTAGAATGATGATGTTTGGCATGTATTTTATGAATGATGTGCCATTTAGAG
ATATTTATATCACAGGACTTATTCGTGATAGCGAAGGGCAGAAAATGTCAAAATCTAAGGGTAACGTTTT
AGATCCTGTAGATTTGATAGATGGTATTTCATTAGATGAGCTTCTGAAAAAGAGAACTACTGGTCTAATG
CAACCACAAATGAAAGCTAAAATTGAGAAAGCTACTAAAAAAGAATTCCCTGAAGGTATCAGCGCTTATG
GTGCTGATGCGGTGAGATTTACTTATGCTGCATTGGCTTCTACATCGCGTGATATCAGTTTTGATACTGC
GAGAGTTGAGGGTTATCGTAACTTCTGTAACAAGCTTTGGAATGCTTCAAGATTTGTAATGATGAATCTT
GATGATTATAAAGTTTGTGATAACTATGAGTTAGGTGTGGCTGATAAGTGGATTTGGAGTGTGCTAAATA
CTGCTACTGCTGATGTACATAGACATCTTGCAAATTATCGTTTTGATTTAGTAACAAACACTATTTATGA
TCTTGTATGGAATAATTATTGTGACTGGTATGTTGAATTTGCAAAAGTTGCTCTAAAAGATGATTCACTA

TCTGAGCAACAAAAAAAATGGTGTTAAATATACGCTTACTAAGGTTTTAGAAAATATTTTAGCTTTAGCGC
ATCCGCTTATACCATTTATCACAGAGAGCATTTATCAGCAGTTAAAAGCACATTTAAATGATGCTAAAGA
TACAATTATGGATGTATCTTATCCTGTAGCAACTCAAGCTTTAGAAGCTCCAGAAGCTGAAAAAGCTATC
GTATGGTTACAAAATGTTGTTACAACTCTACGTAATATGCGTAGCGAAGTAGGTATCAAGCCATCTTTAG
AGATTTCTCTAATTGTTAAAGATGTTGCCGATAAGGATAGAGAATACTTAGCTCAAACAGAAGGGTTTAT
AAAAGCGTTAGCTAGAATAAATAATATTGAGTTTAATGATAATCCGCCAACATCTTTATCACAGATTGTC
GAAGGGCTTGAATTAAATATTCCATTAGCAGGTTTGGTTGATATCGAAGCTGAAAAAGCAAGATTAGATA
AAGAGCTAGATAAGCTAAAAGACGAAGTTGATAGAGTACAGAAGAAACTTTCTAACGAAAGATTTGTCTC
AAATGCTCCAGAGGCTGTAGTTGCTGCAGAGCAAGAAAAATTAGCTAAGTATCAAGAGTTATATGCTAAG
ACACTTGAGAAGAAAGAGGCTTTGGGATAA

>lcl|NC_006570.2_cds_YP_169356.1_266 [gene=gltX] [protein=glutamyl-tRNA synthetase] [protein_id=YP_169356.1] [location=321536..322942]
ATGATTACAACAAGATTTGCACCAAGTCCAACAGGGTTTTTACATGTTGGTGGAGTGCGTACTGCATTAT
TTAGCTGGTTATATGCCAAAAATAACAATGGTAAATTTATTCTAAGAATAGAAGATACTGATTTGGAGAG
ATCTACTCAAGAGGCGGTCGATGCTATTTTAGATGGAATGAGTTGGTTGGGGCTAAAAAATGATGGTGAG
ATCTACTATCAAACAAAGCGCTTTGATAGATATAAAGAAGTGATACAAGAACTTATTGCGGATGGTAAAG
CTTACTACTGTAGCTGTTCTAAAGAAAGACTAGAAGAGTTAAGAGAGTATCAGCAAGCAAATAATCTTAA
AACTGGCTATGATGGTAAATGTCGTGATGCAAACTATATTCCACAACAAGGTGAGAGTTATGTAGTACGT
TTTAAGAATCCTCAAGATGGAGTGGTCAGTTGGGATGATGCTGTCAAAGGTCGAATTTCAATTTCAAATC
ATGAGCTTGATGATATGATTATCCAAAGAGCAGATGGTTCACCAACATACAATTTCTGTGTTGTTGTTGA
TGATATTGATATGGCTATTACACATATTATTCGAGGCGATGATCATGTTAATAATACCCCTAAACAAATC
AATATATACAAAGCTCTAAATGCTAATGTGCCGATATTTGCCCATGTACCAATGATTCTTGGTCCAGATG
GGGCAAAGTTGTCTAAGCGTCATGGTGCTGTTAATGTTATGCAGTACCGTGAAGATGGCTATTTACCTCA
GGCGATACTTAATTATCTTGTTAGGCTTGGTTGGTCGCATGGTGATCAAGAGATTTTTTCTATCGAAGAA
ATGATAAAAGCTTTTAATTTAGAGCACATTAACGCATCACCATCACGTTTTGATTTTGAAAAACTTAAGT
GGCTGAACAAACATTATATCAAAGAGTCTAAGTTTGATGATATTCAAACAGAAGTTGAGTATCACTTTGC
TAAAACTGGTTTAGATATTAGTAATGGTCCAGATTTAAAAGAGCTTGTAGCTGTGATGGCAGAAAAAGTT
GATACATTAGTTGAACTAGCAGAAAAATCTAGTTATTTTTATAGTGATGATATCAGTTATGATGAAAATG
CTGTAAAAAAACATATCAAAGCTTCAACGGGTGAGATATTTGTTAAGTTATTAGAAAACTTTGAAGCTTT
GGATGCTCAGCAATGGCAAGATCCTGATGTTTTACATAATATAGTTAGTACAACAGCAGAGCAGTGCCAA
GTTGGTATGGGTAAAGTTGGTATGCCATTGCGTGTGGCTATTACTGGTTCTGGCCAATCACCAGATATTG
GTATTACTCTAAAACTTCTTGGTAAAAATAAAGTAGTTGCTAGACTTACTAAAGCACTTGAAGAGTTATG
CAAGTAA

>lcl|NC_006570.2_cds_YP_169361.1_271 [gene=folA] [protein=dihydrofolate reductase type I] [protein_id=YP_169361.1] [location=complement(327764..328264)]
ATGATCTCACTAATCGTTGCTTATGATAAAAACTTTGGTATAGGTAAAGAAAATACTCTAGCATGGAAAC
TATCAGAAGATCTTAAGAACTTTAAAAAAATAACAGAAAACAACTATATTGTTATGGGTAGAAAAACCTT
TGAATCGATTGGTCGCCCACTACCGAATCGTAAAAATATTATTTTAACTAGAGACAAAGATTACAAACAA
GATAAATGTTTAATAATAAATAGTACTCAGGATATTTTAAATTTTGCTGAATCAAAACCACACTACGAAA
TATTTATAATCGGTGGTGCACAGATCTATAAAGAATTTCTAAAATACGCAGATAGATTGTATATAACCGA
AGTTGATGCAGAAATGACTGATCTTGATGCTTTCTTTCCACAATGGGATAAATCAAAATATAAACGTATT
GGTCACAAACAATTCAAAAAAGATGATAAAAATGAGTTTGACTTTACATTTAGCGTCTTTGAAAAAAATA
AAAATTTGTAA

>lcl|NC_006570.2_cds_YP_169362.1_272 [gene=rpsB] [protein=30S ribosomal protein S2] [protein_id=YP_169362.1] [location=328496..329215]
ATGTCTTTAATGAAAGAAATGTTATCGGTTGGTGTTCACTTCGGGCACAAAAAGGCTTTCTGGAATCCAC
AAATGAAAGAATACATCTTTGGTATTAATCATGGTGTACATATCATTAACCTAGAAAAAACAGTTCCGCT
TTTCCAAGATGCGGTTAACTTTGTTGGTAAAACTGTTGCTAACGGTGGAAAAATTCTATTTGTTGGTACA
AAAAGACAAGCACAAGATATCGTTGAAGCTGAAGCTAAAAGATGTGGTATGTCATTTGTAAGCCATAGAT
GGCTAGGTGGTATGCTTACTAACTACAAAACGGTTAGACAGTCTATCAAGAGATTGGCACAACTAGAAAA
AATGAGAGAAGATGGTACTTTAGAATCTCTTACTAAAAAAGAGATGTTACAAAATATTAGAACTATCGAA
AAATTAGAGAAAGTTCTTGGTGGTATCAAAGAAATGGGCGGTTTACCTGATGCAATCGTTGTTATTGATA
GCAACAAAGAGCATATCGCTATTCAAGAAGCACAAAAGCTAGGTATCAAAGTTGTAGCAATCGTAGATAC
AAATTCAAACCCAGAAGGTATTGATTATATAATCCCAGGTAATGATGATGCTGTTAAGTCAATATCTTTC
TATATGAAAAAAATTTGCTGATGCTGTTATTGATGCTCAAGGTCTAGATAGAGCAGTTGAAGCAAAAGCTG
ATGAAGCCGCTCAAGCATAA

>lcl|NC_006570.2_cds_YP_169363.1_273 [gene=tsf] [protein=elongation factor Ts] [protein_id=YP_169363.1] [location=329237..330106]
ATGTCAAATATTTCTGCTAAATTAGTAAAAGAACTTAGAGAAAGAACTGGTGCAGGTATGATGGAGTGCA
AAAAAGCTCTAGTTGCCGCTGCTGGTGATATTGAAAAAGCTGCTGAAGAAATGAGAATTTCTGGTCAAGC

AAAGGCTGATAAGAAAGCTTCACGTGTTGCTGCTGAAGGTGTCATTGAAGTTTATGCTGCTGACGGTAGA
GCTATTTTGCTTGAGATTAATTCAGAGACTGATTTCGTTGCTAGAGATGAGACTTTCAAGAAGTTTGCTC
AAGAAGCTGTAAAAGCTGCTCATGCTGCTAATGCTAAGACTATCGAAGAAGTTTTGGCTGCTAAAACTTC
AAATGGTGAAACTGTTGAAGAAGTTAGAAAATCACTAATTGCTAAAATTGGTGAAAACATTCAAGTGCGT
AGAGTTAAGACTGTTGAGGCTGAGACTTTAGGTGCTTATATCCATGGTAGTAAGATTGGTGTGGTTGCTG
CACTAGAGGGTGGTGATGAAGATCTTGCTAAAGATGTTGCTATGCATGTTGCTGCTGCAAACCCTATGGT
TGTTTCAGGTGATCAGGTGCCAGCGGATGTTGTAGCTAAAGAAAAAGAAATCTTCACAGCTCAAGCAAAA
GAAAGTGGTAAGCCAGCTGAAATCATCGAGAAAATGATTGTTGGTAGAATTCGTAAGTTCCTAGATGAAG
TTGCTCTTTTAGGCCAAGATTTTGTTAAAGATCCTGCTATAAAAGTTGAGAAGCTAGTTAAAGATAAAGG
AGCTAAAGTAGTTAATTTCATCAGATTAGATGTTGGTGAAGGTATCGAGAAGAAAGAAGAAGACTTCGCA
GCAGAGGTGATGAGTCAAATTAAAGGTTAA

>lcl|NC_006570.2_cds_YP_169364.1_274 [gene=pyrH] [protein=uridylate kinase] [protein_id=YP_169364.1] [location=330110..330859]
ATGTCTAATGATTCGTCAGAATGTTCTCAAAAACTCCCTAAACTTAAGAGAATTCTTCTTAAGTTAAGTG
GAGAATCTTTATCTGCAGATCAGGGTTTTGGTATAAATGTTGAATCTGCTCAACCTATCATAAATCAGAT
TAAAACTCTTACTAATTTTGGTGTTGAGCTTGCTTTAGTGGTTGGCGGTGGTAATATTTTGCGTGGTGGT
AGAGCTAATTTTGGTAACAAAATTAGAAGAGCTACTGCTGACTCAATGGGGATGATTGCTACTATGATTA
ATGCTTTAGCTCTTCGTGATATGCTTATTAGTGAAGGTGTTGATGCTGAGGTTTTTTCAGCAAAAGGAGT
GGATGGGTTGTTGAAAGTTGCTAGTGCGCATGAATTTAATCAAGAACTTGCCAAGGGTAGGGTTTTGATA
TTTGCAGGTGGTACTGGTAACCCATTTGTAACAACTGATACTACTGCTAGTTTAAGAGCTGTTGAAATTG
GTGCTGATGCTTTGTTAAAGGCTACGACAGTTAATGGCGTGTATGATAAAGACCCAAACAAATATTCAGA
TGCTAAGCGTTTTGATAAAGTCACATTTTCAGAAGTGGTTAGTAAAGAACTTAATGTGATGGATTTGGGA
GCATTTACTCAGTGTAGAGATTTCGGTATTCCGATATATGTATTTGATTTAACTCAGCCTAACGCTTTAG
TTGATGCTGTTTTGGACTCAAAGTATGGTACTTGGGTTACTTTAGACTAA

>lcl|NC_006570.2_cds_YP_169365.1_275 [gene=frr] [protein=ribosome recycling factor] [protein_id=YP_169365.1] [location=330888..331445]
ATGATAAATGATATTCTAAAAGATGCTGAGAATAGAATGAAAAAATCATTAGAGGTTTTAGCTGATGATT
TGGCAAAAATTAGAACTGGTAGAGCACAACCCGATCTATTAGCACATGTTACAATAGATTATTATGGTGT
TGAGACGCCAATAACTCAAGCAGCTAATATTACTGTGCTTGATGCTAGAACTTTGGGGATTACTCCTTGG
GAAAAAGGTTTATCGAGCAAGATTGAAAAAGCTATATTGACTTCTGATCTTGGGCTTAATCCTACTAACT
TGGGTGATTCATTAAGAGTCCCTATGCCTGCTTTGAATGAGGAGAGAAGAAAAGAATTAGTTAAACTAGT
TAAATCTGAAACTGAAGCGGGTAGGGTATCTATCAGAAATATTCGTCGTGATGCTAATGGTGATATCAAA
GAACTTCTAAAAGAAAAAGAAATCACTGAGGATCAAGCTAAAAAAGCTGAAGATGATATTCAGAAGATTA
CTGATAAAATGATTGCTCAGGCTGATGCTCTTGCAGCTAAAAAAGAACAAGATCTAATGGCTGTGTAA

>lcl|NC_006570.2_cds_YP_169366.1_276 [gene=uppS] [protein=undecaprenyl pyrophosphate synthase] [protein_id=YP_169366.1] [location=331478..332251]
TTGTTTATTCTTTCTTATCATTTAGCTAACTTAATTTTTTTCATAATATTTATGACATCGGCTAAAGAAA
ATATTCTAAGGCATCTTGCTATTATTATGGATGGTAATGGTCGGTGGGCAAAGAGTAGATTAAAGCCGAG
AATATTTGGTCATAGAAATTCGGTCTCGAGTGTTGATGCAACTATAGAGTACTGTGTTGAGAATAATATA
GAAATGCTTACTCTTTTTGCATTTGGTCGAGATAATTGGTTAAGACCAGCTCAAGAAGTCAGTGATCTTA
TGGATCTTTTTTATAAAACTCTAAAAGATAAAACTCCAAAGCTACATGATAATAATATTGTTGTTACTGT
TGTTGGAGATCGCTCACGCTTGTCTAATAAGCTTATTGGTATGATTGAATATAGTGAGTCATTAACTAAG
TCAAATACAGGACTAAAGCTTAGATTGGCTGTTGATTATGCTGGTCGATGGGATATAGTTGAGGCTACTA
GAGCTATTGCTAGAGAAGTTGATATTGGTAAGCTTAGTGTTGATGAGATTGATCAGAACAGTTTCGCTAA
ATATTTGGTTGGGGGTAATATGCCTGTCGATTTACTTATTCGTACAAGTGGAGAAGTGCGTCTGAGTGAT
TTTATGTTGTGGCAGTTAGCCTATGCGGAGATGTATTTTACAGATATTATGTGGCCAGATTTTTCTAAGC
AAGAATTAACTAGAGCTGTTGAGTATTTTTATTCTCGTCAAAGAAGATTTGGCAAAAGTGGTGAACAAAT
TTAA

>lcl|NC_006570.2_cds_YP_169367.1_277 [gene=cdsA] [protein=phosphatidate cytidylyltransferase] [protein_id=YP_169367.1] [location=332265..333059]
ATGAAAGAAAGGATTGTGACTGGTATAGTTTTGGTCGCTGTGGTTTTTGGTTTTTTATTTTTTGCTTCTG
ACTATCTGTTTGGTGTTGGTGTATTTTTAGTTACTTTAGTATCAGCGTATGAATGGTTGAAACTTGCAAA
AATTGATCAGCAGTCAATATTTAAAAATCTTATTATATTTACGATAGTGGTTTTTGTGGTTGCACAGTTT
TTTATATATCTTCAATATATATTCCCAATATTTTGGTTGTATGCGATCTATAAATTAGCTAGTTATGAGC
GTCAGAAAATCGATGCAATAGCAACTAATGAAATGCTTGTGATGGGCGTATTTACTATATCTCCTTTTGC
TGCATCATTATATATATACTACATACTAATGGTGTTGCATGGATATTTATGTTTATCTTGGTAATTGCCGCA
GCTGATAGTGGAGCATATTTTACTGGCAAAGCTATTGGTAAACGTAAAATGTTACCACGACTAAGTCCAA
ATAAAACTATTGAAGGTTTATTGGGTGGTTTGATTTGTGCTGTTATTGTTGCAGTGATTTTTCTTGTCTA
TATGAATTTAAGTTTTGGACAGTATATTTATATGGTTATAGTATCTGCTTTAATTGCGTTGTTATCTGTT
GTTGGTGATGTTTTTGAGAGTATGATGAAGCGAATAGCTGGTGTCAAGGATAGTGGCAATATTTTACCTG

GGCATGGTGGAGTGCTTGATAGGTTGGATGGATATATGCCAACTTTACCAATATTTGTGCTGCTTGGTTA
TTTAGCGGGAGTTTTGTTTTTTAG

>lcl|NC_006570.2_cds_YP_169368.1_278 [gene=dut] [protein=deoxyuridine 5'-triphosphate nucleotidohydrolase] [protein_id=YP_169368.1] [location=333069..333515]
ATGAAGGTAGAGCTAAAAATTTTAAATAAAGAACTTATAAAAGAGTTACCCGGTTATGCGACTGAAGGTT
CAGCAGCTATTGACTTAAGAGCATGTATCTCTGAGAGTATTTATCTTAAATCGGGTGAATGTAAACTTGT
TGCAACTGGTATAGCTATTAATATTGCTAATCCAAATTATGCGGCAATGATTTTACCAAGATCTGGTTTG
GGACATAAAAAAGGTTTGGTGCTAGGAAATGGTACGGGGCTTATAGATTCTGATTATCAAGGTGAGCTTA
TGGTTTCTTGTTTTAATCGCTCACAAGAGACTATTGAAATAGAACCGCTAATGAGATTCGCTCAGCTTGT
AATTGTTCCTGTGGTACAAGCAAATTTTGAGATTGTTGAAGATTTTTCACAGCAAAGTGTCCGAGCTACT
GGTGGCTTTGGACATACAGGGGTTTGA

>lcl|NC_006570.2_cds_YP_169369.1_279 [gene=pgsA] [protein=phosphatidylglycerophosphate synthetase] [protein_id=YP_169369.1] [location=333519..334115]
ATGTTTTTTAATATCCCTAATATTCTGACTTTTGGTCGTTTGATATTAATTCCTTTTATAGTTATATGTT
ATTATTTTGAGTTTCCACACCATCATGGTATTACGGCGACTTTATTTTTACTTGGGGCAGCAACTGATTG
GTTGGATGGTTATTTGGCACGTAAATGGAAGCAAACTAGTAAGCTAGGTGCATTTTTAGATCCTGTTGCG
GATAAGCTTATTGTTGCTACGGCGCTTTGTTTGTTTATAGAGATGTATCCATATTGGTGGGCAACAATAC
CTGCTATTGTGATGATTTGTAGAGAAATTGTTGTTTCAGCTTTGCGTGAATGGATGGCTGAACTCGGTCA
GCGTAGTGTTGTTAAGGTTGGTGTTTGGGGTAAAGTGAAGACTGCAGCACAAATGGCAGCATTATTTATA
TTTTTAATCAAACCAGCTATAGATTTTAGACACTCTATAGACTATACGAGTTTCAATACTTGGTTTATAT
TCTTAGGTTTCTTAATGCTTTATGTTGCGGTTATACTAACTATTTATTCTATGTGTAACTATCTCTATGT
GGCTTTTAAATCAGTTTTTGGTGCTTCTGATAACTAA

>lcl|NC_006570.2_cds_YP_169370.1_280 [gene=rpsL] [protein=30S ribosomal protein S12] [protein_id=YP_169370.1] [location=334250..334624]
ATGGCAACTATAAATCAGTTGGTGAACAACCCTCGCAAGAGATCGGTTGTTAAGTCTAAAGTTCCTGCGT
TAAAGGCGTGTCCTCAAAGAAGAGGGGTTTGTACTAGGGTTTATACTACAACTCCTAAGAAGCCTAACTC
AGCACTTAGAAAAGTGGCTCGTGTAAGATTAACGAGTAGATTTGAAGTGACAAGCTATATTGGTGGTGAA
GGTCACAACCTGCAAGAGCACAGTGTAGTGCTTATCAGGGGTGGTAGGGTAAAAGATTTGCCAGGTGTGC
GTTACCACATTGTTAGGGGTGCTTTAGATACTTCAGGTGTTAATAATCGTAAGCACGGTCGTTCCAAGTA
TGGTACAAAGCGTCCTAAGTCTTAA

>lcl|NC_006570.2_cds_YP_169371.1_281 [gene=rpsG] [protein=30S ribosomal protein S7] [protein_id=YP_169371.1] [location=334663..335136]
ATGTCTAGAAGAAATAGAGCTCCTAAAAGAGATATTCTACCTGATCCTAAATATAAGAGTCAGGTTGTTG
CTAAGTTTGTTAACCATATAATGCTAAGCGGGAAGAAATCGATAGCAGAAAAAATAGTATATGGTGCGTT
TGATAAAATTAAAGCAAAAGATGCTTCAGCTAATGAAGTTGAAGTTTTTGAAAAAGCGTTGGAAAGTGTT
AGCCCAATGGTGGAAGTTAAGTCTCGTCGTGTTGGTGGTGCTACATATCAAGTTCCTGTAGAAGTTAGAC
CAGAGCGTCGTCAGACTTTAGGTATGAGATGGATCATCGATGCTGCACGTAAGAGAAAAGAGAATACTAT
GGGTGATAGAGTGGCTGCTGAAATTCTCGAAGCTGTAGAGGGTAGAGGTGCTGCTGTCAAGAAGAGAGAA
GATACTCATAAGATGGCTGAAGCTAACAAAGCATTTGCTCACTTTAGATGGTAA

>lcl|NC_006570.2_cds_YP_169372.1_282 [gene=fusA] [protein=elongation factor G] [protein_id=YP_169372.1] [location=335151..337265]
ATGCCTCGTAATACGGCTTTAGAAAAATATAGAAATATTGGTATCTGTGCTCACGTTGATGCAGGTAAAA
CTACTACTACAGAGCGTATTTTGTTCTATACTGGTTTATCTCATAAGATTGGTGAAGTGCATGATGGTGC
TGCTACTATGGACTGGATGGAGCAAGAGCAAGAAAGAGGTATTACAATTACTTCTGCTGCGACAACTACA
TTCTGGTCTGGTATGGATCAACAGTTTGAAAAACATCGTATTAACATTATTGACACTCCAGGCCACGTTG
ACTTTACGATTGAGGTTGAGCGTTCTTTACGTGTTCTAGATGGTGCGGTTGTGGTGTTCTGTGGTTCATC
TGGCGTTGAGCCTCAGTCTGAGACTGTATGGCGCCAAGCTAACAAGTACGGTGTTCCAAGAATTGTATTT
GTAAACAAAATGGATAGATCGGGCGCTGATTTTGAAAGAGTATGTGCTCAGATTAAAACAAGATTGAAAG
CAAATGTTGTTCCTGTGCAGCTAAACATCGGCGCTGAAGAAGACTTCAAGGGTGTGATCGACCTTATCAG
AATGAAAGCAATTATGTGGAATGAGGAAGACATGGGTCTTACTTATGAGCTTGTTGATATCCCTGCAGAT
CTTCAGGATAGAGCGGAAGAATTGCGTATGGAGATGATCGAGGCGGCTGCAGAAGCTTCAGAAGAGCTTA
TGGAGAAATATCTTGAGGGTGGTGAACTTTCTGAAGATGAGATTCATCAAGGTCTGCGTGCGAGAGTTCT
TAATAATGAAATCGTTCTTGCTTTCTGTGGTTCGGCATTTAAGAACAAGGGTGTTCAAGCAGTTCTTGAT
GGTGTGGTTAGATATCTTCCTGCGCCAAACCAGGTTCCAGCTATTAAATGTGAAACAGAAGATGGTGAGC
CAGCTTCTAGACCATCGTCTGATGATGCGCCATTTGCGGCATTAGCATTTAAGCTAGCTACAGACCCATT
TGTTGGTAACCTAACATTTATTCGTGTTTACTCGGGTGTGCTTAAGTCTGGTGATGCTGTTTATAACCCG
GTTAAGGGTAAAAAAGAGCGTGTAGGTCGTATCGTACAGATGCATGCTAATAAGCGTGATGAAATCAAAG

AAGTTCGTGCTGGTGATATTGCTGCATGTATTGGTTTAAAAGATGTTACAACTGGTGATACTCTTTGTGA
TCAAGAAGATGTAGTAATCTTAGAAAAAATGGATTTCCCAGAGCCGGTAATATCTGTTGCTGTTGAGCCT
AAGTCGAAAGCTGATCAAGAGAAGATGTCAATTGCTTTAGGTAAACTTGCAGCTGAAGATCCATCATTTA
GGGTTAAAACTGACGAAGAGAGTGGTCAAACAATTATTTCTGGTATGGGTGAACTTCATTTAGATATTGT
TGTTGATCGTATGAGACGTGAATTCAAAGTTGAAGCTAATGTTGGTAATCCACAGGTTGCATATAGAGAA
ACAATTAGATCAAAAGTCGAGCAGGAAGCTAAGTTTGTGCGTCAATCTGGTGGTCGTGGTCAGTATGGTC
ACGTTTTTGTCAGATTTGAGCCTTTGGATGAAGTTGATGAGAACGGTGAAGCTAAAGTCTTTAAGTTTGT
TGATGAGGTTGTTGGTGGTGTAGTTCCTAAAGAGTATATTGGTTCAGTTGCTAAAGGTATCGAAGAGCAG
TTGAATAATGGTGTTTTAGCAGGCTACCCAATGATTGGCGTTAAAGCAACTTTATATGATGGTTCATATC
ATGATGTTGACTCATCTGAGATGGCATTTAAGATTGCTGGTTCTATGGCACTTAAAGAAGGTGCTAAAAA
GGCTAATGCTTGTATCCTAGAGCCAATCATGAAGGTTGAGGTTGTAACTCCAGAAGATTACTTAGGTGAT
GTTATGGGAGACCTAAACAGAAGAAGAGGAATTATTGAGGGTATGGATGAAAACCCAAGTGGTAGAGTTA
TAAATGCTCTAGTTCCTTTAGCAGAAATGTTTGGTTATGCTACTAATGTGCGCTCTATTAGCCAAGGTAG
AGCTTCATTCTCTATGGAGTTTAAGAAGTATGCTGAAGTACCAAATAACATTGCTGATGAAATCATCAAG
TCACATAACTCATAA

>lcl|NC_006570.2_cds_YP_169373.1_283 [gene=rpsJ] [protein=30S ribosomal protein S10] [protein_id=YP_169373.1] [location=337286..337603]
ATGGCTATAAATAATCAACGTATCAGAATTAGATTAAAAGCCTTTGATCATAAGCTTATTGATATTTCTA
CACAAGAAATTGTTGATACTGCTAAGAAAACAGGGGCTCAAGTTAAAGGACCTATTCCTTTACCAGTGCG
TAAAGAGAGATTTACAATTCTTATTTCTCCGCACGTAAATAAGAAAGCAAGAGATCAATATGAGATTAGA
ACTCACAAGAGATTAATCGATATTGTTGAACCTACAGATAAAACTGTAGATGCGCTTATGAAGCTAGATT
TAGCATCAGGTGTTGATGTTCAGATCAGTTTAAGCTAA

>lcl|NC_006570.2_cds_YP_169374.1_284 [gene=rplC] [protein=50S ribosomal protein L3] [protein_id=YP_169374.1] [location=337707..338342]
ATGTCTTTAGGATTAGTTGGTCGCAAAATGTGGTATGACTCGTATTTTTACTGAAGATGGTGTTTCTATTC
CTGTAACAGTAGTCCAAGTTGAGCCTAACAAGGTTACTCAAGTTAAGACTGTTGAAAAGGATGGTTATAA
TGCTATTCAAGTAACTACTGGTTTTAAAAAGCGTTCAAATGTAAATAAGCCTATGGCTGGTCACTATGCG
AAAGCTAGTGTTGAGCCTGGTAGAGGTTTATGGGAGTTTACTGTTGATGCAGCAGCTGAATATCAAGTTG
GTTCGTCTTTTGATGCTACTATGTTCGAAGCAGGGCAAAAAGTAGATGTAAGAGGTGTTTCAAAGGGTAA
GGGTTTTCAAGGTGGTGTTAAGCGCCATAATTTTGCAACTCAAGATGCTACTCATGGTAACTCACTATCT
CACAGAGTTCATGGTTCTACAGGTCAAAACCAAACACCTGGTAGAGTCTTTAAAAACAAAAAGATGGCTG
GTCATTTAGGTAATGAGAACGTTACTATTCAGTCTCTTGAGGTTGTGAGAGTGGACGCGGAAAATGGTTT
ATTGCTTTTAAAGGGTGGTATTCCTGGTTCAGTTGGTGGAGATATTATCGTTACTCCAGCTGTGAAAAGT
TGGTAG

>lcl|NC_006570.2_cds_YP_169375.1_285 [gene=rplD] [protein=50S ribosomal protein L4] [protein_id=YP_169375.1] [location=338372..338995]
GTGGACTTAAATATAAAATCTTTGGCTGGTCAAGAGGCTGGATCTTTAGGTGTTGCGGAAGGTGTTTTTG
CGGCAGACTATAATGAAGCCTTAATTCACCAGGTTGTTGTTGCCTATATGGCAGGCGCTCGTCAAGGTAC
AAAAGCTCAAAAAACTAGATCAGAAGTTTCTGGTGGCGGTGCTAAACCTTGGAGACAAAAAGGTACAGGT
AGAGCAAGAGCGGGTACTATCCGTTCACCTATCTTCAGAAAAGGTGGTGTTACATTTGCAGCTAAGCCTA
AGAGTTATAAGCAAAAAGTGAATCGTAAAATGTATTCAGGCGCAGTTAAGTCGATCTTATCTGAACTATT
AAGATCAGGTAGAATGACTATTGTTGAAGAGTTGAAATTAGAAACTCCAAAAACAAGAGAGTTTAAATCT
GTGATTGACTCTTTAGGGGTTAAAGACGTGCTTTTTGTTGTTGGTGTAGAAGAGTTTAGTGAGAATTTAT
ACCTATCTTCTAGAAACCTTAAGAATGTGGCAGTATGTGATTCTGTGGAAATTAATCCAGTTTCTTTAGT
ATGCTTTGAGAACGTTGTTTTAACTAAAAAAGCTATAAAAGAGATAGAGGAGAAGTTAGTATGA

>lcl|NC_006570.2_cds_YP_169376.1_286 [gene=rplW] [protein=50S ribosomal protein L23] [protein_id=YP_169376.1] [location=338992..339291]
ATGAGTTCTCAAGAAAAATTATTAAAAACTGTTATAAGACCTCATGTTTCTGATAAAACTTACGGTCTTT
CAGATGCAAATTCAACTATAGTATTCGAGGTGGCTAGATTTGCAAATAAGCAAGATGTTAAGAATGCTGT
AGAGAAGCTATTTGAAGTTAAGGTTGAGTCAGTTAATATCCTTAATGTTAAGGGTAAGGCGCGTAGATTT
GGTCGTGTTGAGGGTGGAACTAAGGCATGGAAAAAAGCTTATGTGAAGCTTGCTGAAGGACATGATATCA
ATTTTGTTGGTGCAGAGTAA

>lcl|NC_006570.2_cds_YP_169377.1_287 [gene=rplB] [protein=50S ribosomal protein L2] [protein_id=YP_169377.1] [location=339313..340137]
ATGATTGAAATAAAAAAAGCTAAACCTACTTCACCTGGTCGTCGCCACGTAGTGAGCGTAAAGAATACAG
AATTACATACAGGTAAGCCATTTAAGGGTTTAGTAGAGGTAAAAAAAAGCAAGGCTGGTAGAAATAATAC
TGGTAGAATTACAGTTCGTCATCAGGGTGGTGGACATAAACAGCATTACCGTATAGTAGACTTTAAAAGA
AATAAAGATGATATAACAGCTAAGGTTGAGAGAATCGAGTACGATCCTAACCGTAGTGCAAATATTGCTT
TAGTTCTTTATGCTGATGGTGAGAGAAGATATATCGTTGCGCCTAAGGGTTTAAAGAAAGATATGTCTGT

TATTTCAGGTGAAAAAGTAGATGTTGCTGTTGGTAACTGTATGCCTTTGAGAAATATTCCTCTAGGTACA
GTAATTCACAATATTGAAATGAAGCCTAAAAAAGGTGCGCAAATGATCAGAAGTGCAGGTACTTTTGCTC
AGTTGGTTGGTAAAGATAATGCTTATGCTATTATCCGTCTAAGATCAGGTGAGATGAGAAGAGTGCTTTT
AGATTGTAGAGCGGTGATTGGTGTTGTTTCTAATTCTGAGCACAACTTGAAGTCTTTAGGTAAAGCTGGT
GCTAAGCGCTGGAGAGGTATAAGACCTACCGTAAGAGGTGTGGCGATGAACCCAGTAGATCACCCACATG
GTGGTGGTGAAGGGCGTACTTCTGGTGGTAGACATCCAGTCACGCCATGGGGTATCCCAACTAAAGGTTA
TAAGACGCGTAGAAATAAGCGTTCTAATAAGTTGATTGTTCAAAAACGTAAGTAA

>lcl|NC_006570.2_cds_YP_169378.1_288 [gene=rpsS] [protein=30S ribosomal protein S19] [protein_id=YP_169378.1] [location=340152..340430]
GTGCCTCGTTCATTAAAAAAAGGACCTTTTGTAGATCATCATCTTTTAAAGAAGGTTTTTGAAGCGCAAG
AAAGTAATTCTAAAAAGCCAATCAAAACATGGTCAAGAAGATCAATGATTGTGCCAGATATGATAGGTTT
AACTATAGCTGTACACAATGGTCAGCAGCATGTGCCTGTTCTTATGACTGAAGAAATGGTTGGTCATAAG
TTAGGTGAGTTTGTTGTTACTCGTAACTACCGTGGTCATGCAGCTGATAAAAAAGCTAAGAAGAAATAG

>lcl|NC_006570.2_cds_YP_169379.1_289 [gene=rplV] [protein=50S ribosomal protein L22] [protein_id=YP_169379.1] [location=340446..340781]
ATGGAAGTACAAGCTAAATTAAAATTTGCAAGAATCTCAGCGCAGAAGTGTAGATTAGTTGCTGATCAGA
TCAGGGGTCTGCCAGTAGAGCAAGCTATTAATCTTTTGACATTTAGTAATAAGAAAGCTGCTGTATTAAT
TAAGGGAGTTTTGAACTCTGCAGTAGCTAACGCTGAACACAATGATGGTATGGATGTTGACTCTTTAGTA
GTGTCGACTATCTTCGTTGATGAAGGTCCTACAATGAAGCGTTTTGAAGCTAGAGCAAAAGGTCGTGGTA
ATCGTATTCTAAAAAGAACTTCACATATAACTGTGAAAGTTGCTGAGAAAAAATAA

>lcl|NC_006570.2_cds_YP_169380.3_290 [gene=rpsC] [protein=30S ribosomal protein S3] [protein_id=YP_169380.3] [location=340796..341464]
ATGGGTCAAAAAGTAAATCCTAATGGTATCCGCTTAGGTTATATAAGAGACTGGCGTTCAACGTGGTATG
CTGACTCTTCTAGATATGCTACTAAACTTAATGAAGATATTAAGGTTAGAGAGTTTTTGCATAAAAAACT
TGCAGCAGCAGCAGTTAGTAAGATTCAGATTGAGAGACCTGCTCAAAATGCTAAAATTACAATTCATACA
GCTAGATCTGGTATTGTAATTGGTAAAAAAGGTGAAGATGTTGAGAAGTTACGTGCTGAAGTTCACAAGC
TGATGGGTATTCCAGTTCAGATAAATATTGAAGAGGTGCGTAAGCCAGAAATCGATGCTAAATTAGTTGC
TGAAAGTGTTGCTCAACAGTTAGAGAAAAGAGTAATGTTTAGAAGAGCGATGAAAAAAGCAATGCAAGCT
GCTATGAAATCAGGTGCTAAAGGTATCAAAATCATGGTTAGTGGACGTTTAGGTGGTGCTGAAATTGCTC
GTTCTGAATGGGCTAGGGATGGTAGAGTTCCTCTACAAACATTTAGAGCGGATGTGGATTATGCTACAGC
GGAAGCTTTAACAACTTATGGTGTTATTGGTGTTAAAGTTTGGATCTATAAGGGTGAAATCCTTCCAGGT
CAGATCGCTGAGAAGAAAAATAATAAAAAAAGGAGCTAA

>lcl|NC_006570.2_cds_YP_169381.1_291 [gene=rplP] [protein=50S ribosomal protein L16] [protein_id=YP_169381.1] [location=341468..341881]
ATGCTACAGCCTAAGCGTACAAAGTTTCGTAAACAGCAGAAGTTGCGTAATAGAGGCTTAGCTCACAGAG
GTAATAAAGTAAGCTTTGGTGAGTTTGGTCTTCAAGCGACATCTAGAGGTAGAATCACTGCTAGACAAAT
CGAGGCAGGAAGAAGAGCGATTAGCCGTCACATTAAGCGTGGTGGTAAAATTTGGATTAGAATCTTCCCA
GACAAACCTATAACACAGAAGCCTCTTGAAGTTCGTATGGGTAAAGGTAAAGGTTCAGTTGAATATTGGG
TTGCTCAAATTCAGCCGGGTCGTGTACTATATGAGATCACTGGTGTTAAAGAAGAATTAGCGCGTGAAGC
TTTTGCTAGAGCAGCTGCTAAGATGCCAGTGCAGACAACTTTTGTTGAAAAGCAGGTGATGTAA

>lcl|NC_006570.2_cds_YP_169382.1_292 [gene=rpmC] [protein=50S ribosomal protein L29] [protein_id=YP_169382.1] [location=341881..342081]
ATGAAAAGAAAAGATACTTTAAAAGATTATAGAGGTAAAAGTATTGACCAATTGCAAGAAGCGAAAATAG
AGTTATTGCAACAGTTATTTTCTCTTCGCATGCAGAAGGGTACAGGGCAATTAAAGAAAAATCACTTGTT
CAAAAGTGCTAAAAGGGATATTGCTCGTATTAATACAATAATATCAGAAAAGAATAAATAG

>lcl|NC_006570.2_cds_YP_169383.1_293 [gene=rpsQ] [protein=30S ribosomal protein S17] [protein_id=YP_169383.1] [location=342094..342345]
ATGAGCGATAAAATTAGATTGTTAGAAGGTAAAGTTTCTAGCGTAGCTATGGATAAAACTGTAGTTGTAA
GAGCTGAAAGATATGTTAAGCACCCTTTGTATGGTAAGTTCGTTAAGAAAACCACAAAGTATTATGTTCA
TGATGAAAATAATGAATGTAAAGAAGGTGATGTTATCAAGTTCAAAGAAACTAGACCATACTCAAAAACT
AAGAAGTGGTGTTTAGTCGATATTATCCATAGAGAAAAATAA

>lcl|NC_006570.2_cds_YP_169384.1_294 [gene=rplN] [protein=50S ribosomal protein L14] [protein_id=YP_169384.1] [location=342437..342805]
ATGATTCAAATGCAAACAGAACTCCAAGTTGCTGATAATAGTGGCGCTAAGAGAGTAGAGTGTATAAAAG
TTTTGGGTGGTTCTCATCGCAGATATGCATCTATAGGAGATGTTATCAAAGTAACTGTGAAAGAAGCTTC
TCCAAGAGGTAAGGCTAAAAAAGGATCTGTATATAATGCTGTTGTTGTTAGGACAGCTAAAGGTGTACGT
AGAAAAGATGGTTCTAAAGTTCGTTTTGATGGCAATGCTGCCGTGCTTCTAAATGCTAACGGACAACCGA

TTGGGACTCGTATCTTTGGCCCTGTTACTAGGGAGCTTCGTACTGAGAAGTTTATGAAGATCGTATCTTT
AGCACCAGAAGTATTATAG

>lcl|NC_006570.2_cds_YP_169385.1_295 [gene=rplX] [protein=50S ribosomal protein L24] [protein_id=YP_169385.1] [location=342827..343144]
ATGAATAGATTAAAAAAAGGCGATGATGTAATAGTTATCGCTGGTAAAGATAAAGGACGCAGAGGTGTTG
TTAAGTCATTCGCTAAAGGCGGTTCTTTAGTTTTGGTTGAGGGTATAAATATTGTTAAAAAACATATTAA
GCCTAACCCAAATAGAGGTATCGAAGATGGAGTTGTTGAGAAAGAGCTTCCTGTTGATGCGTCTAACGTT
GCTATCTTTAACCCAGCTACTGAAAAAGCAGATAGAGTGGGTTATAAGTTTGTTGATGAGAAAAAGGTTC
GCTATTTTAAGTCTAATGGCGAGCTTGTAGACTTATAG

>lcl|NC_006570.2_cds_YP_169386.1_296 [gene=rplE] [protein=50S ribosomal protein L5] [protein_id=YP_169386.1] [location=343156..343695]
ATGGCAAGATTAAAAGATTATTATCAAAAAGAGCTTGTTGCTAAGTTAAAGACTGAGCTTGGCTTAGATA
ATATAATGGAAGTACCTACTATTAAGAAAATTACTCTTAATATGGGTGTGGGTGATGCTGCAAAAGATAA
GAAGATTATGACTTTTGCATTAAATGATTTGACAGCTATTGCTGGTCAAAAGCCAGTTGTTACTAAGTCT
AAAAAATCAATTGCTGGTTTTAAAATTCGTGATGGATGGCCTATTGGTGCTAAAGTTACGCTACGTGGCG
ATCGTATGTATGAATTTTTAGATAGACTTATAACAATTGCTATTCCTAGAATTAGAGATTTTAGGGGGTT
AAGTGCTAAATCTTTTGATGGAAGAGGTAATTATAGCTTGGGTATGAGAGAGCAAATTTCTTTCCCTGAA
ATTGATTATGATAAAGTTGACTCTATCAGAGGTTTAGATATTTCGATAACTACTACAGCTAAAAATGATG
ACCAAGGAAGAGCTTTGCTTAAAGCATTTGGTTTTCCTTTTAAGTCTTAA

>lcl|NC_006570.2_cds_YP_169387.1_297 [gene=rpsN] [protein=30S ribosomal protein S14] [protein_id=YP_169387.1] [location=343714..344019]
ATGGCAAAAAAATCAATGATTCAGAGAGAATTAAAGAGAGAAAAATTAGTAGCTAAATATGCTCAAAAAA
GAGCTGAGTTTAAGGCTATTATTCTTGATATAAATTCTACTGAAGAACAAATATGGGAAGCTCAAATTAA
ACTGCAAAAGTTACCAGTAAACTCATCAGCTTCTAGAGTTCAAAGAAGATGTAAGGTTACAGGTAGACCA
CATGCTGTATACAGAAAATTCGGCTTATGTCGTAATAAGCTTAGAGAGTATGCAATGGCAGGTGATGTTC
CTGGTTTGAAGAAGGCTAGTTGGTAA

>lcl|NC_006570.2_cds_YP_169388.1_298 [gene=rpsH] [protein=30S ribosomal protein S8] [protein_id=YP_169388.1] [location=344036..344434]
ATGAGTATGCAAGATCCTATCGCGGATATGTTTACAAGAATCAGAAACGGACTTTCTGCTGAAAAAGAAT
TTGTTTCTGTACCATTTTCAAAGATAAAAATGGAAATCGCGAACTTTTTAGTTAACGAAGGTTATATTAA
AAGTTGTTCAAAAGGTACAACTTCAATGGGTCATCCTTCTATTGAAGTAGAGCTTAAGTATCATGCAGGT
GCTCCTGTTATTGAAATGATCAAAAGAGTTTCTAGACCAAGTTTGAGAATCTATAAGTCACACGCAGACC
TACCTAAGGTATATGGTGGTTATGGTGTTGCTATTGTTTCTACATCAAAAGGTTTAGTAAGCGATAGAAA
GGCTAGAGATCTTGGTGTTGGTGGCGAAATAATTGGCTACGTAGCTTAA

>lcl|NC_006570.2_cds_YP_169389.1_299 [gene=rplF] [protein=50S ribosomal protein L6] [protein_id=YP_169389.1] [location=344453..344989]
GTGTCAAGAATAGGTAAAAAACCTGTTGTTATCCCAAGCGGTGTTACGATTAATGTTGCTGCTGGTAACA
AGGTTGAAGTTAAAGGTGCAAAAGCAACTTTAAGTAAAACTTTTTCTACTGATGTGACTTTTAGTGTTGC
TGATAATGTTGCGACTATAACGCCAAATAATAACAGCAAAAATGCTGTTGCACAGTCTGGTACTGCAAGA
GCAATACTTAGCAATATGGTTGAAGGTGTTAGTAAGGGCTTTGAGAGAAAGCTAAAAATTATTGGTGTTG
GTTATCGTGCTAAAGCACAAGGTAACGAGCTTAATCTTACTTTAGGCTTCTCACATCCTGTAGTTTACAA
GTTGCCACAAGGTATAACAGCTGAAACTCCAGCTCCTACAGAAATTATTCTAAAAGGTGCTGATAAAGAG
CTTTTGGGCAAGGTAGCTTCAGAGATAAGAGAATATAGAAAGCCTGAGCCTTATAAAGGCAAAGGTGTTC
GTTACGAAGATGAGTATGTAGCGAAGAAAGAAGCTAAGAAGAAGTAG

>lcl|NC_006570.2_cds_YP_169390.1_300 [gene=rplR] [protein=50S ribosomal protein L18] [protein_id=YP_169390.1] [location=345012..345365]
ATGGATAAAAAAACTGCTCGTTTAAGTCGTAGTAAGCGTACTAGAATTAAACTAAGAGAGCTTGGTCATA
CTAGACTTTGTGTTTATAGAACACCAAGACACGTTTATGCGCAGGTGATTTCTGGTGATGGTTCTACTGT
ATTAGTAGCTGCATCTACTGTAGAAAAAGATGTTAAAGCAAAATGTAAATATACAGGTAATGTTGAGTCT
GCTGCAATTGTAGGTGAAATCATTGCTGACAGATGTAAAGAAAAGGGTATTTCACAGGTTGCTTTTGATA
GATCTGGATATAAGTATCATGGACGTGTTAAAGCTTTAGTAGAAGCTGCTAGAGAGCATGGTCTTCAGTT
TTAA

>lcl|NC_006570.2_cds_YP_169391.1_301 [gene=rpsE] [protein=30S ribosomal protein S5] [protein_id=YP_169391.1] [location=345393..345893]
ATGTCTAATGAAGTGAAAAAAAACGAAGAGCTGATTGAAAAGTTAGTTAGTGTTAAAAGACACTCAAAAA
CAGTAAAAGGTGGTAGAATTATGAGCTTTGCTGCTTTAACTGTTGTGGGTGATGGTAAAGGTAGAATTGG
TGTTGGTAGAGGTAAATCAAGAGAAGTGCCTGCTGCTATCCAAAAAGCTATGGAAAACGCTAAAAAGAAC

ATGGTATCAGTAAACTTAAATAATGATACTTTATGGTACCCTGTGATGTCTAACCATGGTGCTTCTAAAG
TATTCATGCAACCAGCATCTGCAGGTACTGGTATTATTGCTGGTGGTGCTATGCGTTCTGTTTTTGAAGC
AGTTGGTGTACACAACGTTTTAGCAAAAACATACGGCTCAACTAATCCAGCGAATGTTGTAAGAGCAACA
ATTGCAGGTTTGGCAAAAATTAAGTCACCAGATGAGATCGCTGAGAAGAGAGGTCTATCTGTTGAAGAGA
TCCAGGGGTAA

>lcl|NC_006570.2_cds_YP_169392.1_302 [gene=rpmD] [protein=50S ribosomal protein L30] [protein_id=YP_169392.1] [location=345900..346085]
ATGACTCAAGCTAAAACATTTAAAGTTACTTTAGTAAAAAGCCTAATTGGTCGTAAAGAAAACCACATAG
CTTCAGCTAGAGGTTTGGGCCTAAGAAAAATTAACCACACTGTAGAAGTATTAGATACTCCTGAGAATCG
TGGTATGGCTAACAAGATATATTATATGGTTAAGATAGAGGGGTAG

>lcl|NC_006570.2_cds_YP_169393.1_303 [gene=rplO] [protein=50S ribosomal protein L15] [protein_id=YP_169393.1] [location=346092..346523]
ATGAAATTAAATACACTTGCTCCTGCTGCTGGCTCAAAAAGCGCTCCAAAAAGACTAGGTCGTGGTATCG
GAAGTGGTTTAGGTAAAACTTCTGGTAAGGGTCACAAAGGTCAAAAAGCGCGCTCAGGTGGCTATCATAA
AGTAGGTTTTGAAGGCGGACAAATGCCTTTACAAAGAAGACTACCAAAATTTGGTTTTACTTCTGCATCT
AAAGGATATGTTGCTGAAATCAGACTTCATGAATTAAATAATGTGGTAGCTGATGAAGTCACATTAGATA
CCCTAAAAGATTTTGGTCTTATTAGAAAAGATATCAAGACAGTAAAAGTAATAGCTAGTGGAGAAATCCA
AAAAGCTGTTAGCCTAAAAGGTATAGCTTGTACAAAAGGTGCAAAAGAAGCTATCGAAAAAGCTGGCGGT
AAAGTAGAGTAA

>lcl|NC_006570.2_cds_YP_169394.1_304 [gene=secY] [protein=preprotein translocase subunit SecY] [protein_id=YP_169394.1] [location=346534..347859]
ATGTCAAAGTATAATAGTGCTTCTGGTACAACAGGTGAATTAAAATCTAGACTGATTTTTGTAGTCATTG
CTATCTTGGTATTTAGACTCGGAGTTTATATTCCTATACCAAATATAGATCCTACTAAGTTGGTAGAGAT
TATTTCAAATCAACACTCATCAACCGGTGGTTTGATGAGTATGTTTAATATGTTCTCTGGTGGCGCTCTT
ACTCAAATGAGTATCTTTGCATTGGGTGTGATGCCTTATATTTCGGCATCAATTATTTTTCAGATGCTTT
CAGCTGTATATCCAAAATTTATAGAGCTAAAAAAAGAAGGTGAGTCTGGTCAAAAGAAAATCACTCAATA
TACAAGATATTTAACTCTTGCTTTGGCTATAGTACAATCATTTGGTATTGTTGCATTTGTATTACATCAA
GATGGTTTAGTAACTACAAATAATATGGCGTTATTTTATTTGACGACTATTGTTTCTGTTACAACGGGTA
GTATGTTTTTGATGTGGCTAGGTGAGCAAATCACTGAAAGAGGTGTTGGAAATGGTATTTCACTACTAAT
CTTTTCAGGTATTGTTGCTAACTTGCCGTTTGAAATCTCAAACACATTATCACAAGCTAACCAGCATGTA
ATATCTTACTTGTCTGTATGGGTGCTTTTAATTTTGCTACTATTAGTTATTGCATTTGTAGTATTTATGG
AAAGTGCTCAGAGAAAAATTACAGTAAACTATGCTAAGAGACAGCAAGGTAGAAAAATGTTTGCTGCTCA
GACTAGCCACTTGCCTCTAAAATTAAATATGGCTGGGGTAATTCCAGCAATTTTTGCATCATCAATATTG
ATGGTTCCAGGTGTATTACTTGGTTGGTTATCTAACTATAATTCACTTAGTTGGTTGGCAGATGTTGCAG
AGATGCTGCAGCCAGGCAGTATAGTTTATACGGTAGTTTTTGCTGCGACAATTATCTTTTTCTGTTTCTT
TTATACTTCTTTGGTATTAAATCCAAAAGAAACAGCAGATAATTTAAAGAAATCAGGCGCTTATATTTCT
GGTGTGAGACCTGGTGAGCAAACAGCTAAGTATATAGATGCAGTTATGACTAGATTAACTTTGGTTGGTT
CATTATATATCACAGCTATATGTTTATTACCAATTTTCGTAGTGAAGTTCTTTGCACAAGGATTATCATT
TACATTTGGTGGTACGTCTTTGCTGATCGTGGTAGTTGTGATGATGGACTTTATGGCTCAAGTTAGATCT
CATATGATGTCAACACAATATGATTCTTTACTAAAAAAAGCAAATCTTAGTGGTAAGAGAAAATAG

>lcl|NC_006570.2_cds_YP_169395.1_305 [gene=rpmJ] [protein=50S ribosomal protein L36] [protein_id=YP_169395.1] [location=347883..347996]
ATGAAAGTTAGAGCTTCAGTTAAAAAAATGTGTAGAAACTGTAAAGTTATCAAGCGCAATAGAGTAGTTC
GTGTGATATGTACAGATCCTAGACATAAGCAAAGACAAGGATAG

>lcl|NC_006570.2_cds_YP_169396.1_306 [gene=rpsM] [protein=30S ribosomal protein S13] [protein_id=YP_169396.1] [location=348113..348469]
ATGGCTCGTATAGCTGGTGTTAATATTCCTGTTCATAAGCATACAGTGATAGGATTAACTTCAATTTATG
GAATAGGTAAAACAAGAGCGCAACAAATTTGCCAAACTTGCAATGTAGATCCAACTGTCAAAATTAAAGA
TTTATCAGAAGAACAAGTTGAATCTTTAAGAACAGAGGTTGCTAAATTTACGGTAGAAGGTGATTTACGC
CGTGAAGTTTCTATGGACATAAAAAGACTTATGGACTTAGGATGCTTCAGAGGTAGAAGACATCGTCGTA
GCCTTCCTGTAAGAGGACAAAGAACGAAGACTAATGCACGTACTCGTAAGGGTCCAAGAAAGCCAATTAA
GGCGTAA

>lcl|NC_006570.2_cds_YP_169397.1_307 [gene=rpsK] [protein=30S ribosomal protein S11] [protein_id=YP_169397.1] [location=348509..348898]
ATGGCTAAGTCTGTTAGATCATCAAAGAAAAAAGTAAAAAGAGTAGTGACTGATGCAGTTGCTCATATTT
ACTCATCTTTTAATAACACTATAGTAACTATTACAGATAGACAAGGTAATGCTTTATCTTGGGCGACTTC
TGGTGGTAGTGGCTTTAGAGGTTCGAGAAAAAGTACACCTTTTGCTGCGCAGGTAGCTGCTGAGAGAGCT
GCTGATATGGCTCTTGAGTATGGTGTAAAAAACGTAGATGTTTTAGTAAAAGGACCAGGTTCAGGTAGAG

ATTCTGCTGTTAGAGCTTTGAATGCTAAAAACTTAAAAGTAACAAGCATAACAGATGTGACTCCATTACC
TCACAATGGTTGCCGTCCTCCTAAGAAACGTCGTGTTTAA

>lcl|NC_006570.2_cds_YP_169398.1_308 [gene=rpsD] [protein=30S ribosomal protein S4] [protein_id=YP_169398.1] [location=348920..349540]
ATGGCTAGATATCTAGGACCAAAATGTAAGCTTTCTAGAAGAGAAGGTACTGACTTATTTTTAAAAAGTG
GTGTAAAAGCTAACGATGAAAAATGCAAAATGAATACTGCACCAGGTCAACATGGAGCAAGAAGAGCGCG
CCTATCTGACTATGGTTTACAGTTAAGAGAAAAGCAAAAAGTTCGTCGTATGTATGGTATTTTAGAAGGT
CAATTTAAAAAATACTATGTCGAAGCTAGCAGAAGAAAAGGTAATACCGGTGCTACATTGTTAGAGCTAT
TAGAATCAAGACTAGACAATGTTGTTTATAGAATGGGTTTTGCTGCTACACGTGCAGAAGCAAGACAACT
GGTAGTTCACAAAGGTATCATGGTGAATGGTCATACTTGTAACGTGCCATCTGCTCAAGTAAAAGCTGGC
GATGTAGTTGCAGTTAGAGAAAAAGCTAAGAAACAACTAAGAATTCAAAATGCTGTAGAACTTGCTAAGC
ATAGAAAAGAGCTTTCGTGGATCGATGTTAATACTGATTCATTAGAAGGTACTATGAAATCTTCACCAGA
TAGATCTGAGTTATCAGCAGACATAAATGAACAATTAATCATCGAGCTTTACTCTAAGTAA

>lcl|NC_006570.2_cds_YP_169399.1_309 [gene=rpoA1] [protein=DNA-directed RNA polymerase subunit alpha] [protein_id=YP_169399.1] [location=349598..350569]
GTGAGTAATAATAATTCAAAACTGGAATTTGTACCTAATATACAGCTTAAAGAAGACTTAGGAGCTTTTA
GCTATAAAGTCCAACTTTCTCCTGTAGAAAAAGGTATGGCTCATATCCTTGGTAACTCTATTAGAAGGGT
TTTATTATCTTCACTATCAGGTGCATCTATAATTAAAGTAAACATCGCTAATGTACTACATGAGTATTCT
ACTTTAGAAGATGTAAAAGAAGATGTTGTTGAAATTGTTTCTAATTTGAAAAAGGTTGCGATAAAGCTTG
ATACAGGTATAGATAGACTAGATTTAGAACTATCTGTAAATAAATCAGGTGTAGTTAGCGCTGGAGATTT
TAAGACGACTCAAGGTGTAGAAATAATAAATAAAGATCAGCCAATAGCTACTTTGACAAACCAAAGAGCA
TTTAGCTTAACTGCTACAGTGAGTGTAGGTAGAAATGTCGGAATACTTTCTGCGATACCAACCGAGCTTG
AGAGAGTTGGTGATATAGCTGTAGATGCTGATTTTAATCCTATTAAAAGAGTTGCTTTTGAGGTTTTTGA
TAATGGTGATAGTGAAACTTTAGAAGTATTTGTAAAGACAAATGGTACTATAGAACCACTAGCAGCTGTT
ACGAAAGCTTTAGAGTATTTCTGTGAGCAAATATCAGTATTTGTATCTCTAAGAGTACCTAGTAATGGTA
AAACAGGTGATGTATTAATAGATTCTAATATTGATCCTATCCTTCTTAAGCCGATTGATGATTTAGAGCT
AACTGTCAGATCATCTAACTGTCTGCGTGCAGAAAACATTAAGTATCTTGGTGATTTGGTACAGTATTCT
GAATCACAGCTTATGAAGATACCTAACTTAGGTAAGAAATCTCTCAATGAGATCAAACAAATTTTAATAG
ATAATAACTTGTCTCTAGGTGTCCAAATTGACAATTTTAGAGAGCTAGTTGAAGGAAAATAA

>lcl|NC_006570.2_cds_YP_169400.1_310 [gene=rplQ] [protein=50S ribosomal protein L17] [protein_id=YP_169400.1] [location=350614..351051]
ATGAGACATCGTAAGCAAGGTAGAAAGTTCGGTAGAACTAGTAGTCATAGAAAAGCTATGTTTAAGAACA
TGTCAGCTTCTTTGATTAATCATGAGCTTATCAAAACCACTTTACCAAAAGCTAAAGAGTTAAGAGCAAT
CGTTGAGCCTCTAGTAACTTTAGCTAAGAGAGAGAGCATAAGTTAAGACAAGAACTAGACACTAACTCAAAT
GAGTTCAAAGCACAATCAGTTGCTTTAAGAAGACAAGCTTTTGATTTCTTAAGAAATAAAGCTGCTGTAA
CTAAACTTTTTGAAGAGTTTGGTGCTCGTTATGCAGAAAGAGCTGGTGGTTATACTAGGATTCTTAAATG
TGGTTATAGATTCGGTGATAAAGCGCCTATGGCTTTCATCGAATTAGTTGACAGACCTCAAGTTGAAGAA
GCAGCTGACGAAGAATAA

>lcl|NC_006570.2_cds_YP_169412.1_322 [gene=poxF] [protein=phenol hydroxylase] [protein_id=YP_169412.1] [location=365352..366083]
ATGGCACTAGAAAAAATTTGAACTTGAATTAGTATCTTTTAAAGATATTACTGATAAGGTTAGACACTTTG
TTTTCAAAAGAACAGATGGTAAGCCTCTTGATTTTATTGCGGGACAATTTATTACGTTTCTGCTCACAGA
TGAAGATGGTAATATAAAGCGTAGAAGCTACAGTTTAGGATCATTACCTGCTGATAATATGCTTTTAGAG
ATAGGTATGACTTATGTTGAGGGCGGTATCGCCACAGATACTTTTTTTAATATGAAAGTTGGTGATACAG
CAGCTGCGATGGGACCAGCTGGTAGATTAGTACTAAAAAAAGATGAAGAAATCAGAAAACTAATTTTAGT
TGGTACTGGTACTGGTATTGTTCCATACAGAGCGATGTTCCCAGAACTACTAGAAAAAGCTGATAATACT
GAGATACATATTCTTTTAGGTGTGCAATATCGTAAAGATGCACTTTATCAAGATGATTTTATTAAGTTTG
CTAAAAAGCATCATAATATCCACTTTAAACTATGCCTAAGTAGAGAAACTCAAGATCTTAGAGATTATGA
AATTTCAGGATATGTACAAAACCAATTTGATAAAATAGGTTTAGATCCAGAAAAAGATGTTGTCTATGTT
TGTGGTAACCCAAATATGATAGATGAATCATATGAAATGCTTACACAAGCTGGCTTTAACGCCAAAAATG
TTCGCCGCGAAAAGTATATTTCTTCGAATTAA

>lcl|NC_006570.2_cds_YP_169413.1_323 [gene=rpmE] [protein=50S ribosomal protein L31] [protein_id=YP_169413.1] [location=366205..366420]
ATGAGACAAGAAATTCACCCTAAATATACAGAAGTAACTGTAACTTGTAGCTGTGGCAATACTTTCGTTA
CAAGATCAACAGCTGGAAAAAAAGAAATGAACATCGATATCTGTTCAGAATGTCACCCATTCTATACTGG
TAAGCAAAGAATCGTTGACACTGCTGGACGTGTTGATAAGTTCAAGAAGAGATTTGGCGGCATGAAAAAA
ATATAA

>lcl|NC_006570.2_cds_YP_169414.1_324 [gene=gshA] [protein=glutamate--cysteine ligase] [protein_id=YP_169414.1] [location=complement(366631..368136)]
ATGTACGATTTCAAAAAAATAAATAACCTACGCGGGATAGAAAGAGAAACTTTAAGAGTTACTGATTGTG
GTAATCTAGCAACTTCTAATCACCCTGATGGTTTAGGACATAAGTTGACTAATAATAGTATTACCGTTGA
TTTCTCTGAAAATTTGCTTGAGCTGATAACTAAACCGCATGATAGTATAGATAAAGCAATCGGTGAGCTA
TATCAACTTTCGGCATTTACTTTGGATAATATGCACAGTGATGAAATTATCCTTAATACTAGTATGCCAC
TATCTGCTAACGATAATGATATACAGGAAGCAGATTTTGGGAGCTCAAATTCGGGGCGAATGAAGCGTGT
TTACCGTAAAGGGCTATCTGCACGATATGGTAAAATTATGCAGATAATTTCCGGGATACATTATAATTTT
TCTTTTGATAAAGACTTAATTAGTAATATTGCTACTAACAAGCAAGTATCAATATCAGATATTTATTTTG
ATGTGCTTAATAACTATTTTGAGTTTATGTGGCTTTTACCGTATCTTTTTGGCGCTAGTCCTATATGTGC
TAAGACTTCTGTTAAAAATAAACCTGATTATTTATCAGTTTTAGATGATAAGTTTTATGTTGGTGAATAC
GCTACAAGCTTGAGAATGAGTGACCTTGGCTACACAAGTCCCGCACAAAAAGATTTAGCAATATCATATG
ATAACGTTAAGGCATATGTTAAAGATTTAATTCAAGCAACTGATGATACTTTTGCTGATTATAAGCGTAT
AGGCCTTTATAATTCTCAAGGGCAAAGAATACAGTTAAATGATGGTATTTTACAGATAGAAAATGAATAT
TACAGCGCAATTAGACCTAAGCAAATTGCTAAAAGAGGAGAGAGACCTGCTTGTGCATTATATAACCGTG
GTGTTGAGTATGTTGAGGTTAGAGTTCTTGATGTCGATCCATTTGAGCCGGTGGGTATAAGCAAAGATAC
AGCGCTTTTTGTTGAGGTGATGTTGATGACTTGTCTAGACAAAGATGCTAAAAAATATCATAAAGATATT
ATCAAACAGGCAAAACAAAATTTAACTGCAGTTGCAATACAGGGACGTAATCCACAACTTAAGCTTAAAA
AGCTTGATGATGATAGTGAAATACTTTTAAAAGATTATGCTTTAGAATTGTTTGATGAAATTGAGGCTGT
AGCTAAGAAAATGCCTAAAGAATACTTAGATGCTGTTGAAATTCAAAAGCGCAAAGTATTAGATATATCG
CAAACACCTTCAGCTAAAATAATAGAATTAGCTAGACAACATGGTTATAAAAAATTTATCTTAGATATAT
CTCGCCGAGTATCACAACAATTCAGAAGTTATGAGCTTCCAGCAGCAATTGTAGCTAAATTAAAAGACCA
AGCTGGCCAATCTGTTGCTGCAGAAAAAGAATTAGTTGCTAATGATAAAATTTCCTTAGATGAATATATC
AATAGATATTATAAATCTTCAAAGGGTTGTTGTTAG

>lcl|NC_006570.2_cds_YP_169417.1_327 [gene=yjeE] [protein=nucleotide-binding protein] [protein_id=YP_169417.1] [location=complement(370900..371310)]
ATGAAAAGTATACTAGTAAATGATGAAGAGCAAATGTATCAACTTGCCAAAGAGTATTCTCAACAGCTAA
AGCCTGGTCAGATTATCTATTTGTATGGTGATTTAGGTGCGGGTAAAACTACGTTTGTCAAAGGTATACT
CAATGCACTTGGTTATACTGGTAACGTTAAAAGTCCCACTTATACTCTAGTTGAGAGTTATGAGTTTGAC
AAATTTGATATTTATCATTTTGACTTATATAGGTTGGCTGACCCAGAAGAGTTAGAATGGATAGGTGCAC
GTGATTATTTCAATCAAAAAGATATTTGTTTTATTGAGTGGCCTGAGAAAGGTAAAGGTTTTTTACCATT
AAATACCACTAAGGTACATATAAAATATCTAGCTCAAGGTAGACAAGTAGATTTTTACTAA

>lcl|NC_006570.2_cds_YP_169418.1_328 [gene=folC] [protein=bifunctional folylpolyglutamate synthase/ dihydrofolate synthase] [protein_id=YP_169418.1] [location=complement(371288..372469)]
ATGAGCGTCGAAATTAAAATAGCTAAATTAATGGCTAAGCCTGATGCTCTCTATTGCGATCTTCTTGATT
TATCACTAATTCTTAGTAGATTTAATTTTGCTAAAAAGTTTAAAGTCATAACTATAGCTGGTACTAATGG
TAAAGGTACGACAGTAGCTATGTTAGAAGAGCTTTTAGTAACTAATAATAAGAATGTTTTAAGTCACACT
TCACCACACGTTTTTAAATTTAATGAAAGAATATCATTAAACAAACAGCCAATATGTGATAGTGTTCTTT
TGGAGATTTTAGAAAGATTAGAAGAATTGGCTCCTGAATATCGTCTCTCATATTATCAAATTGCTTTTTT
ATGCCTTTGTATCTATTCGCAAAGAGTTGAGCTTGATTATCTAATTTTAGAAGTTGGTATTGGTGGTAGA
TTGGATGCAGCAAATATAATTGATGCTGATATAACAGCTATTACAAATATTGACTTTGATCATTGTGAGA
TATTAGGTGATACTCTTGATAAAATAGGTTTTGAGAAAGCTGGAATATCAAGGCCACAAGTTCCACTTTT
TCTAGGTTCACAAATGCCGCAGAGTGTCTATGAATATGCACAAACGATAGGGGCAGTTATTTATCAAAAT
AGTTATGAGTATAGCTCAAGACAGTGTTTTACTCATAGTTATAATATAGCCATGGGGATAGCAGAATATC
TTTTTTATAGGATGCAGATATCATATATCCCAAATCTTGAGGATATAAGAGCAAGAGCAAGGTTTGCGAC
TCTTAAGAATGATTCGCTAAATAACAGTTATGTTGTTGTTGATGTTGCTCATAATCCTGCTTCAGTCAGA
CATTTATTTGAGCTATTAGAGAGCAAATTTGCAGGCAAAAATATTCGCTATGAGGCAATATTTGGTATCT
TAGCAACCAAAGATATACGCGAAGTACTTAATATAGCTAAACAGCATGTCTATAAGTGGGATGTCATAGA
TCTTAAATATCTCGACTCAAGAGCAGCTGATCTTGAAAAGATAAAACAAGAATTTAAATTACAACAGATA
ATTCGTGTAGATTATAACAAAGATTTAAGCAGTGTTTATCTAGCAAAAAAAGATACTCTTATGGTAGTGT
TTGGATCTTTTGTATTAGCAGGAGAATTTATAAGACATTATGAAAAGTATACTAGTAAATGA

>lcl|NC_006570.2_cds_YP_169419.1_329 [gene=accD] [protein=acetyl-CoA carboxylase subunit beta] [protein_id=YP_169419.1] [location=complement(372484..373392)]
TTGGAAATGAGTTGGTTAACTAGAGTAATTGGTAGAAGTCTTGGTTTAGAAGCGCAGAAAAAAGATATGC
CATCTGGAGTTTGGAGTCAGTGTCCTAATTGTGGTGTAACTCTATATTCAGAAGAGCTACATAACAATAA
GTCAGTATGTCCAAGCTGTAATTACCACTATAGAATTTCTGCTAGAAATAGACTAAACCTATTCTTTGAT
AGAGATAGTATGCAAGAGCATTTTGCAAATGTATCGCCTGTTGATATGCTTAAGTTTAAAGATACAAAAA
CTTATAAGGATAGATTAGCTCAGGCACAGAAAAAAACTGAAGAGCAAGATGCTTTAGTGGTAATGGAAGG
AACAGTTAAAGGATTTCCTGTAGTTGCAGCAGCATTTAACTTTATGTTCTTAGGTGGTTCTATGGGTTCT
GTAGTGGGTGAGAAGTTTGTTAGAGGTGTCAAGCTTGCTATTGAGAAAAAAGTGCCATTTATCTGCTTTA

CAGCAAGTGGTGGTGCTAGAATGCAGGAATCATTGTTCTCACTAATGCAGATGGCAAAAACTAGCGCAGC
TTTACAAAAATTAGCAGAAGCTAAACTACCATACTTGGTAGTTTTGACAGACCCAACAACCGGTGGTGTA
TCTGCTTCACTTGCTATGCTTGGTGATATCCATATCGCGGAGCCAAAAGCTTTGATTGGATTTGCTGGTC
CTAGAGTAATCGAGCAAACTGTAAGAGAAAAATTACCAGAAGGTTTCCAAAGAAGTGAATTTTTAGTAGA
GAAAGGTATGGTTGATATGATCGTTGATCGTAGAAACTTAAGAGGTGAAGTCGCTAAATTAATAGACAAA
CTAATGCCTAATCTCACTAAAATAAACTACTCTCAACCTTTAGAGTATAAGTCTGAACAACAAGCTTAA

>lcl|NC_006570.2_cds_YP_169420.1_330 [gene=ndk] [protein=nucleoside diphosphate kinase] [protein_id=YP_169420.1] [location=complement(373428..373850)]
ATGACTAAACAAAGAACTTTATCTATAATTAAGCCAGATGCTGTAGAGAAAAATGTAATTGGTGAGATCT
ATAGCCGTTTTGAGAAAGCAGGATTAAGAATTATTGCAGCTAAAATGAAGCATTTGTCAAAAGCAGAAGC
TGAAAGATTTTATGCAGTACATAAAGATAGACCTTTTTTTAGTGCGCTTGTTGAGTTTATGATTTCAGGT
CCTGTGATGATACAAGTTTTAGAAGGTGAAAATGCAATAGCAAAAAACCGTGAGTTAATGGGTGCAACAA
ACCCTAAAGAAGCTAAAGCAGGCACTATTAGAGCCGATTTTGCTGATAGTATCGATGCAAATGCTGTCCA
TGGCTCAGATGCAGAAGATACAGCAGCACAAGAGATAAGATATTTCTTTAGTGATACTGAGATTTTCGGC
TAA

>lcl|NC_006570.2_cds_YP_169421.1_331 [gene=pyrG] [protein=CTP synthetase] [protein_id=YP_169421.1] [location=complement(373923..375563)]
ATGAATTCTAACACTAAAATTATTTTCGTCACAGGTGGGGTAGTATCATCACTTGGTAAGGGTGTAACTG
CGGCATCTTTGGCTACTCTCTTAGAAAGTCGTGGTCTTAATGTAACAATGATGAAGCTTGATCCATACAT
CAATGTTGATCCAGGGACTATGAGTCCATTGCAACATGGTGAAGTTTTTGTAACCGAAGATGGCGCAGAG
ACTGATCTTGATTTGGGTCATTATGAGCGCTTTATCCGCAATAAGATGACTCAAGCAAATAACTTCACAA
CCGGTAAAGTATACCAGAGTGTGTTAAGAAGAGAGCGTAAGGGTGATTATCTAGGTGCTACTATCCAGGT
GATTCCACATATCACTGATGAGATCAAAAGGCGTATTTGTAGTGGTATTGCTGATGATGTTGATGTTGCG
ATTGTTGAGATTGGTGGTACTGTTGGTGATATCGAGTCACAACCATTTTTAGAAGCAATTAGACAACTAA
GAATAGAGTTAGGTAGAAATAGAACATTATTTGTGCATTTAACTCTTTTACCTTATATAAAAGTTGCTGG
TGAGATTAAAACAAAACCAACACAACACTCTGTCAAGGAGTTAAGAGGTATCGGAATTCAAGCTGATGTA
CTAGTTTGTCGTTGCGAGAAAAAATTTGATGATAGTGAAAAGCGTAAAATAGCACTTTTTACTAATGTTG
ATCAGGATTGTATATTTACAGCAGAAGATGTTGATACGATTTATGAAGTGCCACTTAAGTACAACCAGCA
AGGTTTTGATGCAAAGCTTGTTGAACTTTTAAACCTAAATGCTAAAGAAGCTGATCTATCCGAATGGCAA
AATGTTGTAAATACTATTAGAGATGTCAAAGGTGAAGTCACTATTGCGATGGTTGGTAAGTATGTTTCAT
TAACAGAAGCGTATAAGTCGCTAAATGAGGCATTGTATAATGCGGGTTATAAAAAAGGCGTTAAAGTAAA
AATAAAATTTGTTGATTCAGAAGATGTTAACGAGAATAATGTTGAGTCGTACTTCAAAGATGTTGCTGCT
ATTTTAGTACCAGGGGGGTTTGGTAGTAGAGGTGTTGAAGGTAAGATAATCTCAATAAAATATGCTCGAG
AAAATCAGATTCCTTTCTTGGGGATATGTCTAGGAATGCAGTTGGCTGTTATCGAATACGCTAGAAATAT
TCTAGGTATAAAGGACGCTCATTCTAGTGAACTAGAGCCAACGACAGCAAATCCAGTAATTGGATTAATA
ACAGAATGGCAAGCAGAAGATGGTACTGTTCATCAAAGAACACATAGTTCTGATCTCGGTGGGACAATGC
GTCTAGGTGGATATAAGTGTGTCTTAAAACAAGGCTCGCGCGCTAGAGAAATATACCAAGCTGATGAAGT
GGTTGAAAGACATCGTCACCGCTACGAGGTAAATAGTAATTATGTTGAGCGCCTTGAGGAAGCAGGTTTG
ATTTTCTCAGGAAGATCTGAAGATAATAAATTGATGGAGCTTATCGAAATTCCTCAGCATAAATGGTTTA
TAGCATGTCAGGCGCATCCAGAGTTTACTTCTACACCAAGATATGGACATAAATTGTTTGAATCTTATAT
CCAAGCAGCTATCGAAAATTCTAATAATTAG

>lcl|NC_006570.2_cds_YP_169425.1_335 [gene=gdh] [protein=glutamate dehydrogenase] [protein_id=YP_169425.1] [location=complement(382014..383363)]
ATGAATGCTCAGAAGTACATTGATAGCGTAATTGCTCAAGTAGAAAAAAGAGATGGTCATGAAAAAGAAT
TCATCCAAGCTGTAAAAGAAGTATTTTCTACACTTAAACCAGCTCTAGAGCATAATCCTAAATATATCGA
AGAAAATATTCTAGCACGTATGGTTGAGCCTGAAAGAGGTATTTCTTTTAGAGTACCATGGGTTGATAAG
GATGGTAATATCCAAGTAAACCGTGGTTACAGATATCAGTTTAATGGCGCTATCGGTCCTTTTAAAGGCG
GGATTAGATTCCACCCAAGTGTATACTCTGGTATTATCAAATTCTTAGGCTTCGAGCAAGTTTTCAAAAA
CAGCTTAACTACTCTACCTATGGGTGGTGGTAAAGGTGGTGCTGACTTTGACCCTAAAGGCAAAACTGAT
GCTGAAATCATGAACTTCTGCCAAAGCTTTATGATTGAATTACAACGTCATATTGGTCCAGATATCGATG
TCCCAGCTGGTGATATTGGTGTTGGTGGTAAAGAAATCGGCTATATGTATGGACAATACAGAAGAATTCG
TGCATGTTTTGAGAATGGTGTATTAACTGGTAAATCGCTAGAGTCAGGCGGTAGCTTAATCCGTCCTGAG
GCTACAGGTTATGGTGCAGTATTCTTCTTAGATGAAATGCTAAAGCATGATGGTGAAACTTTACAGGGTA
AAACTGTAGTAACATCTGGTTATGGTAACGTTGCTTGGGGTGTATGTAAGAAAGTTGCTCAATTAGGCGG
TAAAGTTGTTACAATATCTGGTTCAAAAGGTTTTGTTCACGATCCTGAAGGTATTACAACTGATGAGAAA
ATTGAATTCTTATTAAAAATCCGTAACGGTGAAAAAACAATGCAAGACTACTCTAAAGAATTCAATGCAA
GCTGGCACGCAGGTCAAAAACCTTGGGGTATAAAAGCAGATATCGCTATCCCTGCAGCTACTCAAAACGA
GATAGATGTTGAAGATGCTCAAAAACTTATTGATTCTGGTGTTAAGTATGTTGTTGAAGCATCTAACATG
CCAACAACTAATGAAGCAATTGAATTCCTTATGAAGAAAGGTATAATCCTAGCTCCAGGTAAAGCTGCTA
ACGCTGGTGGTGTTGCAGTTTCTGGTCTAGAAATGAGCCAAAACTCAGCTAGACTATCTTGGTCAGCTGA
AGAAGTTGAATCTAAACTTCAACAAATCATGGCTAACATTTTCCATGCTTGTAAGTTTGCTAGTAGTAAA

TACAACCTTGGCTACAACCTAGTTGCTGGTGCAAACTTAGCTGGTTTTGAAAAAGTAGCAGAAGCTATGA
TTCAACAAGGTAGATACTAA

>lcl|NC_006570.2_cds_YP_169429.2_339 [gene=psd] [protein=phosphatidylserine decarboxylase] [protein_id=YP_169429.2] [location=complement(385993..386844)]
ATGAGAGATAATTTATTTATATATTTACAATATCTACTTCCTCACACTTTAACATCACGTTTAGTTAGTA
AACTTGCTGACTCAGAAAATAAAATTATTAAAAACCATCTAATAAAACTTGCTATTAAGAAATTCAATAT
TAATTTAGTTGAAGCTAAAGAAACCGATATAAGCAAATACAAATCTTTTAATGATTTTTTCATAAGAGAA
CTTAAAGATGATTTAAGACCCATTTCTAATGATAAAAATGTTATCTCCTCTCCTGCAGATGGTGTATTAA
GTCAATTCGGCACGATAACTGATAATAGTCTAATTCAAGCCAAAGGTAAACTCTTTTCTCTAGAATCATT
AATTGCTAGTAGTTCAACAACTAGCTTCACAAAATTTGCAACTATATATCTTTCACCAAAAGATTATCAT
AGAGTACATATGCCTATAGATGGAAAGCTTACAAAGATGGTTTATATTCCAGGCAAACTTTTCTCGGTTA
ATAAAATAACGACCAGTAAAGTTGATAATCTCTTTGCAAAAAATGAACGTCTAATCTGCTACTTTGATAC
AATAATTGGAGAAATTGCAGTAATTTTTGTCGGTGCTCTTTTAGTTGCAGGTATAGAAACAGTATGGCAT
GGTAAAATTGCTCCGAACTACTACAAAGACATACAAACTTGGGACTATAATTCTGCTAAATTTAATATCA
AATTCAATAAAGGCGATATACTTGGATGGTTTAACTTCGGCTCAACAGTAATTATTTTAACTTCTGGAAA
TAATGTCAGTTTCAAATTTGAAGAGAATAAAAATAATATTAAAATACAAGTAAATCAAGATTTAGCTTTG
ATTACAGAATAA

>lcl|NC_006570.2_cds_YP_169432.1_342 [gene=glmU] [protein=UDP-N-acetylglucosamine pyrophosphorylase/glucosamine-1-phosphate N-acetyltransferase] [protein_id=YP_169432.1] [location=388990..390357]
ATGGGTTTATCAGTTGTTATTTTGGCTGCAGGCAAAGGTTCAAGAATGAACTCAAACAAACCAAAGGTTT
TACAAACATTAGCAGCAAAAACGTTGATTGAGCATGTTGTAAGTTCTGTTGAGAAACTAAACCCAGATAA
TATAGTTGTTGTTACAGGGCATCTAAAAGAGCAAGTTGAAGATGCATTGCAAGGAAGAAATATAACATTT
GTCTATCAGCAGCAGCAACTTGGTACAGGTCATGCGGTATTACAGGCATTACCTTACTTAAAAGAACAAA
AAGTTCTTATTCTTTATGGGGATGTACCTTTAATTTCAACTGAAGTTTTAGAAAACCTTGTTGATACTAC
CAATGATGATGACCTTGGCGTCTTGACCGCGTTTGTTGAGAATCCTCAAGGTTTAGGTAGAATTGTTAGA
GATAAGTTTGGTGCAGTTACAGAGATTGTTGAAGAAAAAGATGCGAATGATATCCAGCGTCAAATAAAAG
AAATTAATACTGGAATTTATTGTGTCCATAAAAATTTACTGCAAAAATGGTTGCCAGAGATAAAAGCAAA
TAATGTCCAGAAAGAATATTATTTAACAGATATAATCACATTTGCAAAAGCCGATCATGTTTCTATAAAT
GTAACTCATCCAATTAATGAGTTTGAAATTTTGGGTGTTAATGATAGAACACAATTAGCTAGTCTTGAGC
GAGTTTGGCAAAGAAATGTTGCTGAAAAGATTATGGCTAAAGGTGTAAGTATTGCTGATCCGAATAGATT
TGATGTGCGTGGTAATCTTGATGTTGGTAAAGATTGCTGGATAGATATTAATGTAATAATCAAAGGTAAT
GTTAAGCTTGGTAATAATGTTGTCATAGGAGCTAACTGTATACTTAAAAATTGTATTATCGAAGATAATG
TTAGAATTAAATCTAACAGTATGGTCGATGGTTCTATTATTCGTGAAGGTGCAATAGTTGGTCCTTTTGC
ACGTGTTAGACCTGAATGTGATGTTAAAGAGGGTGCTGTTATTGGAAACTTCGTTGAAGCTAAAAAAACG
ATTCTTGGCAAAGGTTCAAAAGCATCGCATTTAACTTACCTTGGTGATAGTGAAATTGGTGCTAATTGTA
ATATAGGCGCTGGAGTAATAACTTGTAATTATGATGGGGTTAATAAACATAAAACAGTTATAGGGGATTA
TGCGTTTATTGGATCTGATTCACAATTAATAGCTCCAGTTAACATTGGTCAAGGTGCAACTGTTGGTGCT
GGATCAACTATTGTAAAAGATGTTCCCGCTGATAATCTTGCAATTTCAAGAGCAAGACAGCGTCATATTG
ATACTTGGCAGAGATCCGTCAAGAAAACAGATAAATAA

>lcl|NC_006570.2_cds_YP_169433.1_343 [gene=glmS] [protein=glucosamine--fructose-6-phosphate aminotransferase] [protein_id=YP_169433.1] [location=390378..392216]
ATGTGTGGAATAGTAGGTGCTAACTCTACAAGAAATGTTACTAATATCTTAATTGAAGGTTTAAAAAAAC
TAGAGTACAGAGGTTATGATTCTGCTGGTTTGGCAATAATTGATGATAAAAATAATATAGATATATGTAA
AGAAGTTGGTAAAGTTATTGAACTAGAGAAATCTGTACATAACTTAGCTAATTTTAAAGGAGATATAGGT
ATTGCTCATACTAGATGGGCTACTCATGGTAAACCATCTAAGAATAATTCTCACCCTCATGCTTCGGAAA
GCTTTTGTATAGTCCATAATGGAGTCATAGAGAACTTTGCTGAGCTTAAAAAAGTTCTTATTAATGATGG
TTATAAATTTAAGTCAGATACTGATACTGAGGTTATCGCACATTTGCTACAAAAAGAATGGCGTGATAAT
TTTAGCATAGTTGATAATATTAAATATATTATGGCTATGCTTAAGGGAGCATATGCCGTAGCAATAATCT
CACAAAAATTCTCTGATAAAATTGTTGCGGTGCGTTCAGGTTCGCCACTTGTAATTGGTGTGGGTATAGA
TGAGAATTTTATTTCATCAGATGCATTATCATTATTACCAGTTACAAATAAATTTTCTTATCTTGATGAA
GGTGACATTGCAATTATTTCTAAAGACAATGTTGAGGTTTTTGATAATAATGGTGCAGCAAAAAATCTTG
AGGTTGAGGAGTATAATTACTCTTCATCAAGCGCCTCTAAAGATGGTTATAAGCATTATATGCTCAAAGA
AATATATGAGCAGCCAGAGGCAGTTTCAAATACTATCTTAGCATCATTAGCTGATGGTGAAATTAGTCTG
GATAGTTTTGATAAAAGAGCTAAAGAATTATTTGAAAAAACCAAACATATTTGTATAGTTGCATGTGGAA
CTAGCTATAATGCTGGGATGACAGCAAAGTATTGGATTGAAAAATATGCAAAAGTTCCATGTAGTGTCGA
AATAGCAAGTGAGATTAGGTATAGAGATAATGTTGTGGTTGATGGTTCTTTGTTTGTCAGTATTTCTCAA
TCTGGTGAAACAGCAGATACTCTAGAGTCACTTAGAAAGAGCAAAAAGCAAAATTATGTTGGCAGTATGT
GCATTTGTAATGTGCCAAATAGTTCGCTTGTGAGAGAATCTGATATTGCTTTTATGACAAAAGCTGGTGT
TGAAATTGGAGTGGCTTCAACCAAGGCATTTACAACACAGTTGGTGGCATTAGCAATATTTACATTGGTA
ATTGCTAAACTCAAAAATAGTTTAACAGATCAACAGATAGCTAAATATACTGAAGAACTTAAAAATATCA

GAGCTTTGGTTATGGGAGCCTTAAAACTAGATACTGAAATAGATCAGATAAGTGAGTATTTTTCTGATAA
AGAGCATACTATCTTTTTAGGAAGAGGATTATATTATCCTATAGCTATTGAAGGGGCCTTAAAACTTAAA
GAGATCTCTTATATCCATGCTGAAGCATACCCATCAGGAGAGTTAAAGCATGGTCCTCTAGCTCTAGTTG
ATAAGAATATGCCAATAGTTGCAGTTGTGCCAAATGATGAATTATTAGATAAAACCTTATCTAACTTACA
GGAAGTACATGCTCGAGGCGGCAAGCTAATTCTTTTTGTTGATAAAGCTGTTAAAGAAAGAGTTAACTTT
GATAATAGTATTGTGCTAGAGTTAGATGCAGGACATGATTTTAGTGCGCCTGTGGTATTTACGATACCGC
TTCAGCTGTTGTCATATCATGTGGCTATAATCAAAGGAACGGATGTTGATCAACCTAGAAACTTAGCTAA
ATCTGTAACCGTTGAGTAA

>lcl|NC_006570.2_cds_YP_169438.1_348 [gene=map] [protein=methionine aminopeptidase] [protein_id=YP_169438.1] [location=393921..394691]
ATGAGTCAAATAATAATAAAAACTCCTCAAGAAATAGAAAAGATGCGCGTTGCTGGTAGACTAGCAGCAG
AAGTTTTAGAGATGATTACTCCTTTTGTTAAAGAAGGAGTTACAACCGCAGAGCTTGATAAAATTTGTCA
TGAATATATAGTCAAAGAGCAAGATGCATATCCAGCGCCGCTTAATTACCATGGGTTTCCTAAATCAATT
TGTGCTTCAATAAATCATGTTGTTTGTCATGGAATTCCAGCTGATAAGAAGCTTAAAAATGGTGATATAT
TAAATATCGATATTACAGTTAAAAAAGATGGCTATCATGGTGATACAAGTAAGATGTTTATGATAGGTGA
GCCATCAGTCATGGCTAAAAAACTAGTAGAAGTTACTCATGAATGCTTATGGAAAGGTATAGAAGTAGTT
AAACCTGGCAATCATTTTGGCGATATCGGCGCAGCTATAGAAAAGCATGCTAAGAAGTTTGGTTATTCGA
TAGTTGATGCTTTTTGTGGCCATGGGATAGGTGCTAATTTCCATGAACCACCGCATGTTATGCATCATGG
TAAAGCTGGTACAGGCGCAATGTTTGAAGAAGGAATGATATTTACAATCGAACCGATGATAAATATCGGT
AAAAGAGCAGTGTCAGTGCTAAAAGATGGTTGGACTGCTGTTACAAAAGACCGTTCACTCTCAGCACAGT
GGGAGCATACAATTTTAGTTACAAAAGATGGTTATGAAGTCCTAACACTAAGAGAAGAAGAAAAAAACTA
G

>lcl|NC_006570.2_cds_YP_169439.1_349 [gene=FTT_0394] [protein=hypothetical protein] [protein_id=YP_169439.1] [location=394693..395541]
ATGCCAAAACAAGTAACTCAAAAACTGGTAAATCAAAAGTGTGATCTTTTAAGATCTCAAAATGAAGAGA
TTACTGTCAGTAAAGTTAGAAAGCTGATTGGTGAGGGTGTATCTATTATTGATTTAGTAGAAAAAGTTAC
TTTGTATAAAGAAGATAAAAAACAAGCTTTAGAAGTTGCAGAGCAAGAAATACTAGAGCCTAATCAACCA
GTACGTGATGAGCTGCTTGAAATCATAAGAGCTAGTCTAAAACAGTTTGACGTTGATAGAGATGATATTG
CTTTTAGTTTACGTAGTGATATTATGCAATATATTCAACAACAAATATCTAATAATATCTCTAAGCTTAA
GCATAAGCAAGCTGAGCTTTCTAATAAGAACGATAGCTTAGAAATTTCTAATATTAGTTTAGATCGTCGC
TATAAAGAATTACTCGAAAAATATAATCAAATCAAAGAAGAAGCGTATAGTTTAAAGCAAAATTATAATT
CAAAATCCATGAAGTTTTTAGAAAAAGAAACAACAGAGAAAATGCTTTTAGCTTGGGAAGACTTTAAGGG
TATCAAAGAGCAATTGGTTAGTTTGAAAATGTATTCTAAGGTTGCTGCATATGATAAAAGTGGTGTTATT
GTCATTAAGTTTCCAGCAACAGATTTTTTGACTCAAGAGTGCAGAGCAGGAGTAAGCAGATACCTAAAGG
CAAAAACTGTATTTGATTATAGTATCCAAGCTTGGGTTCTCTCTGGCTTTAGGGATATTCTAAAAACTTT
AGATTTTTTACAAAGAAATAAGTTTGTATTCTCCAAAGAGTTAGAAACTATTGCTTACTTAAGAAGACAG
AAGAGTTAA

>lcl|NC_006570.2_cds_YP_169442.1_352 [gene=mtn] [protein=5'-methylthioadenosine/S-adenosylhomocysteine nucleosidase] [protein_id=YP_169442.1] [location=398658..399344]
ATGAGAAAAATTGCAATATTAGGAGCAATGGAGATAGAAATTTTACCAATTCTAGCTAAGTTAGATAACT
ATGAAACTATAGAATATGCAAATAACAAATACTATCTAGCAAATTATCAGGATAAAGAGCTTGTAATAGC
TTATAGTAAAATTGGTAAAGTATTCTCAAGCTTAACAGCAACGATAATGATTGAGCGCTTTGGTGTTGAG
GCATTATTGTTTAGCGGTGTTGCTGGGGGGCTACAAGATTTGAAAGTAGGTGATATGATTGCTGCAACTG
CTACAGTACAACACGATGTTGATATCACCGCATTTGGCTATCCATATGGTAAGATTCCTATATCGGAAGT
TGAAATTAAAACATCGGCTAAACTTTTAAAACAAGCACAAAATGTTGCTAATGAACTTGGTCTTAATCTA
CATACTGGTGTTATAGCTACAGGAGATCAATTTGTTCATTGTGCAGAGAGAAAAGATTTTGTAATCAAAG
AGTTTGATGCAAAGGCTATAGAAATGGAAGGAGCAAGTGTTAATCTTATCTGTAATGAAATGGGTGTGCC
AAGTTTAATTCTAAGAAGTATTTCTGACACTGCTGATGGCGATGCACCTGAAAATTTTGATGAGTTTGCT
AAGATGGCTGCTAAAAGATCCGCAAACTTTATAATGCAAATACTTAGTAATATATAA

>lcl|NC_006570.2_cds_YP_169445.1_355 [gene=slt] [protein=soluble lytic murein transglycosylase] [protein_id=YP_169445.1] [location=401240..403216]
GTGATTAATAAAAAGTTCATAACAAGTTGTTTATTGATTTTATCTAGTACTATTGGTTATAGTCTCACGA
CACAACAAGTTGAATATTCTCAAAAAGCTATAGATGCATTAGCTAAAAAAGATTATAAATCATATTATTA
TTATAAATCAAAACTCAAAGATACTAGCATTTATCCATACTTACAATATAAAGAAATAAGTACAGATCCA
GATATTTTTCAGCAAACAACAATAGATGAGTATTTTAAACAAGACAATAACAGCTATTGGCAAAATCGCC
TTAGCGATGATTTAGCGCAATATTATGCTCAAAAACAGGAATGGAAACTCTTTGATAAATATTATAAAGG
TGACTTGAGTATATCTGGTAAGTGTTGGAGTATGCAAGCAGAATATGAATCAGGTGACAAAAACAAAGCA
TTAAATGAATATGGTCAACTTTGGCAGAATCGAGTATATATGCCTGCTGCATGTAATCCAATGCAAAAAT
ATTGGGATAATTCTGACTATAAACCAAGCAGCTATCTAACAACTAAAGCATATACTCTTGCTTTTGCTAA

TAAATTTGATGATAGCCTGTGGCTATTAAATACTTATGTTAAAGATAACAAAGATTATCTTAATTATATA
ACGGCATGGAAACAGGCTACTAAAGATCCACGTAAATTGGATAGCTTCATTAATAGATTTCATAACTATA
ATCATTTTGACAAAGTATTTGTTGATATTTCTAGAGACCTAATAAGAAAAGATGTTGAAAGCTACGCAAA
AGTATGGAATAACCTAAAAAATAAAAGATATCTAAGCACGAAAGTGAAGCAACAAACTATCTCAGCAATA
GCAGTAAGCTTTGCAAGGTCACAATCGCCACAAGCACAACAGTGGCTAAGCAAAGTAGATAAAAATTATC
TTGATACAACAGCTTGGGAGTGGTTGTTAAGAGTTGATCTATATAATGAGAACTTCAAAGATTATATACA
AACATACAATCAGCTACCTAAAAATTCTCAACAAGACCAAGCATGGAGATATTGGCTAGCATATAGTTAT
CAACAGACAGGCCAAAAAGCAAAAGCTGAAGATATATTTGAGAGTTTGACTAAGACTCCTCTTGATTATT
ATTCATTCTTAGCGGCTGATAAGCTTGGTAAGCCATATAACTTTGGTAATGATGTTGCTACAGCATTAAC
TAATAGTGAAACTAAAAAACTACTAACAGAAGATACTACTGTGCAGGCTATTGACTTATATCAGATCGGT
CAATATAAAGATTCAACAAGTATCTGGCAATGGGCAATTAGAAATAAGCTTAGAGATAAACAGATTGACG
AGATTAAACAACTTGCAAGATTAGCTGAGGATAAGCAAATGTATTATGCAGCTATATTCAATATGTCAGT
TATTGGTAGTTATAATAGTATTGACATGCTCTTTCCGAAAGCTTTTATAAATATAGTTAATCAAAATGCT
CAAAAATTTGCTATTGATAAAGATCTTGTTTTATCAATTATGCGTAAAGAATCACTATTTGATATTAGCG
CGGGTTCTTCAGCTGGAGCAAAAGGATTGATGCAGGTTACAGAACCAACTGCAAAATTTATAGCTCAAAA
ATATAAATTATCTCTAGTTGGTGATAGCTCTCAAGGTATGACTAGTCAGATATTTATCCCAGAGAATAAT
ATTAAACTTGGAACAGCTAATTTATATTTCCTAGAGAAGCTTTTTGATAAGAATCCTGTGTTAGGAATAG
CAGCTTATAATGCTGGTCCTGGTAATGTTGCTAAGTGGCTAAATGAAAATGAGGTTCCAGCAGCAATTTG
GATAGAGAATATTCCATTTGGTGAAACACGTCATTATGTAAGAAAAGTTCTAATGTATATGATTGTATAT
AATAATTTTGTGTTTAAAGATAAAAAAGATCATATTAGTAATTTCTTGGGTTATAAGATATCTGATAAGC
AAAGTTTTAGAAAATAA

>lcl|NC_006570.2_cds_YP_169447.1_357 [gene=dnaE] [protein=DNA polymerase III subunit alpha] [protein_id=YP_169447.1] [location=404585..408064]
ATGATTTCTCATCTTAGAGTTCATACCGGATATTCTGTTGTTGATAGTACAGTGCGCCTAGGTGATCTTT
TTGCAAGAGCAAAAGATAAAAATATTGTTGCAGTAGCTCTTACTGATGTATGTAATTTATTTGCTGCAGT
TAAGTTTTATAAACAAGCTTTAGCTAGTGGGATTAAGCCAATCTTTGGAGTTGAGCTAAAGGTCGATACT
GAATTTGGCATATGTGATTTAGTATTATTAGCGGAGAATAATCAAGGTTATCAAAATATTGTTAAACTAG
TCTCAAAAGCATATCAAGAAGCTGATAGGTTTGGTTCAATTCCTCTTATACCTAAATCTTGGCTTAGCGA
GGTAAAACTAGAAAGTGTCATTTGCTTAAATGGTGGTCAACAAGGAGAGTTAGGCAAAGCTATCTTAGCT
AAAGACTATATCAAAGCAGATGAGATTATTAAACAAAATATTGAGATATTCGGGACTAATAACTATATCA
TTGAGATTCATAAGTTAGAGTATGAGAATGAAGGTTTGTATAATGAAAAAGCTCTAGAATACGCAAGTAA
GTACAATCTCATAGCTGTAGCAACAAACTTAACTGTATTTATGGAAGCAGATGATTATGATATTCATGAA
ATTAGAGCCTGTATCAATGAGAAGACAACAATATTAGATGAGTCGCGTAAATCTAAATTTACTAAAGAAC
AATATCTAAAATCAGCTGATGAAATGTATGAAACTTTCTCTGCACTGCCAGTACTTGTAGATAATACACT
AGCAATTGCTAAGCGTTGTAATGTCACATTTGAATTAGGTAAGCCGTGTTTACCGACTGTCAATATTCCA
GCTGGTTTAACAGAAAAAGAGTACTTTTCAAAACTGTGCTATCAAGGTTTAGATAAGCGTTTAGAAAAAA
TTCTAGAAAATAGGTCAGCTGATAAGCATGAACATATCATAAAAGTCTATAAAGATAGGCTACAACGAGA
AATTGATATCATTTGTGATATGGGTTTCCCAGGATACTTTCTGATTGTCGAAGATTTTATCCGCTGGTCA
AAAGAAAATGATATCCCTGTTGGACCAGGTCGCGGTTCTGGTGCTGGTTCATTGGTGGCGTATTCTTTGT
TAATTACTGATATAGATCCATTACCTTATGGATTACTTTTTGAGAGGTTTTTGAATCCTGAAAGAGTATC
AATGCCAGATTTTGATATAGATTTTTGTATTCAAGGTAGAGATAGGGTTATCAAGTATGTTGAGCAAAAA
TATGGCAAAGAGAGTGTCGCACAGATTATTACCTATGGTACGATGGCTGCTAAAGGTGTTGTGCGTGATG
TTGTACGGGTAATGGGCCAAAGTTTTGGTTTTGGTGATAGGATTGCGAAGTTAATACCAGAAACACCGGG
GACAACATTTAAGAAAATTCTTCATGAAGGTGAGACATTGTATGAGGAAGTGCAAGCAGATGAGGACGTT
GCCGAAATTATCGAGAAGGCTCAAAAACTTGAAGGTTTGCCGCGTAGCTTAGGTAAGCATGCTGCTGGAA
TTGTGATATCACCGACAAAAATTTCTGATTTTGCACCTGTGTATTGTGAGAATAAGGGTGGAGATATTGT
AACGCAATTTGATAAAGGTGATGTTGAAGATGTTGGTCTGGTTAAATTTGACTTTTTAGGACTAAAAAAC
TTAACGATCATCAATAATACTGTTAAAAGCATTAATGCTAAAAGACCAGCTGATCAAAAACCTTTAGATA
TCGCTGATATACCTTTAGATGATCAAAAAACATTTAAGCTGTTACAAGCTGGTAATACTACGGGGATATT
TCAGCTTGAGTCTCAAGGTATGCGTCAGATTGTCAAAGACCTTGGTACTTCTAATTTTGAGGAGATTATT
GCTCTAGTAGCGTTATATCGTCCGGGACCGATGGAGAATATCCCAACATTTATTGATCGTAAGCATAGTA
GAAAACAGATTACTTACTTGCATCCGTTACTTGAAGGAGTACTAAAAGAAACTTATGGTATTCCAGTATA
TCAAGAGCAGGTTATGCAGATGGCACAAAAACTTGCAGGCTATACTCTTGGTGGAGCAGACTTATTACGC
CGTGCTATGGGTAAGAAAAAACCTGAAGAGATGGAGAAACAGCGTAAAATCTTTAAAGAAGGTGCCGCTA
AACATCATAATATTGATACCAAGCTTGCTGACGAGATATTTGACCAAATGGAGGCTTTTGCTGGTTATGG
TTTTAACAAATCACATGCTGCTGCATATGCCTTGATTGCGTATCAAACAGCTTGGTTAAAAGCACATTAT
CCAGATGAATATATGGCTGCCCTTATGTCTGGAGATATGGGGAATACTGATCAGCTCGTTAAATTTATCC
TAGATTGTAAAAATATGAGTATCACAGTTCTAGCACCTAATGTAAACAAAAGTGTCTATGACTGTATAGC
TGTCTCAAAAGGAACGATCTTGCTTGGCTTAGGAGCTATCAAGGGGCTTGGTGGTGAGGCTATCAAAAGT
ATACTTACAGAGAGAGAGATATAGCTGGAGAGTTTAGCTCTATATTTGATTTATGTCGCAGGGTTGATTTAC
GCAAAGTTAATAAAAAAGCGCTTGAAGCACTATGTTATGCAGGCGCTATCAAAGATATCTCTAAAAATAG
AGCAACAGCATTTAACTCTATCGAAAAAGCCATCAAAAATGCTGGTTATGTCAATGAGATGAATGCTGCT
GGGCAGGATGATTTATTTGGTTTTACAGAGCAAGAGAGTGATACTGATGCAGAACTAGAGAGAGAATGTA

TAGTAGAAGAGTGGAATCTCAAAGAGTTACTTATTAATGAGAAAAAAGCTTTAGGAATGTATTTCTCAGG
ACATATTATTGACGAGGAGAGCCACTGGCGCAATCATGTTAGCTTTAGCGACCTTGAAAAAATTCAACAA
CCTAATATGGATGGTAATTCCGTAAGAATTATCGCAAGTATGATAACTCCACCAGCAAGACGTAAAACTA
AGACTGGTCGAGTTTTATATATTATCAATATTGATGATGAGTTTGATAGAGCTGATTGTTTGATTAGTGA
AGAGGTTTTTGCTAGTGTTAAAGATAGTATCGAGGTTGATGATATTGTTGTTGTAGAGGGCAAAGTTAGT
CGTGATGTGCAACGAGAATGTAATAAGCTAAGTGTAGATAAAGTTCAACCAATATCACAATATATTGATG
AAAATTTTAGTAAGGTTGAACTAACATTAAGAAGTCAAAATATAAATCCTACTAATCTCAAAAAAGTTCT
CAGTAATCTCAAAGAAAATATAGCCTCAACCAATATCCAAAAACAAAATATTTTAGAAATAACGGTTTCT
CTTGGTGATGTTGATGGTAAGTTTGATTACTTACAAAAGCCATTTTCTATCTACAAATTTGTCAATGATG
TTAGCAAGCTAATAGCTGAGGGTAGCACAGTTAGTTTGAGTGGGGTTTAA

>lcl|NC_006570.2_cds_YP_169449.1_359 [gene=lolC] [protein=lipoprotein releasing system membrane protein] [protein_id=YP_169449.1] [location=409479..410741]
ATGTTTAAAAGCTTACCACTATTTATCGGATTAAGATATATCCGTGCTAAGAAGCGTAACCGGTTTATAT
CTATTATATCGGCAATATCATTTTTGGGTATTTCTCTAGGTGTGGCTGTACTTATCACAGTGATGTCGGT
TATGAATGGGTTTGATCAGCAGATCAAAAGCAAAATTCTAATGATGGTACCACCTTTAAAAGTGTATCAA
TTAGGTGGGGAAGTTACTGATTGGCCAAACTTAGCTAAAGAAGTTGAGAAAAGTACTCCAAGTGTTACTG
CAGTAGCTCCGATAGTAGAATCACAAGGTTTACTTAGTGCAAATAGTGGTAGTAGCACGACAGCATTTGT
ACAAATTCAGGGTATAGAACCTAAGTATCAAACCAAAGTACTACCTATAGCTGAGCATATTGTCGATGGT
AAGCTGTCATCGTTAGATGATAATCAAGGTTATAATATAGTTTTAGGTAGTGTCTTGGCTGATAATTTAG
GTGTTAAAGTTGGTGATAAGGTGACTTTAATCGTACCAAAGATTAGTCTAACGCCGGCTGGGATGATCCC
AAGAATTAAGCAGTTTAGAGTGTCTGGAATATTCTCTGTGAGCTATCAATATGATGCTTATTATGCGATG
ATTAATATTAAGGATGCACAAAAAGTTTTTGAGACGGGTAATTCAGTATCATCTCTTCAACTAAGTGTAA
AAAATATCTATGATGCACCACTTGTTAAAGACAAACTCAATGATGGTGCCATTCCTCCTTACTATTTCAC
TCGTGATTGGACGGATGAGAATAAATCTTTCTTTGACGCCTTAAAAATGGAAAAAACGATGATGTTTTTT
ATCCTATTACTAATAATTACTGTCGCTGTATTTAACTTATTATCATCTTTAGTTATGGTTGTCACTGATA
AGCGTAGTGATATAGCTATTCTTAGAACTATGGGGATGTCATCGCGACAAATTATAACGGTATTTATATA
CCAAGGTTTTATTATTGGTTTGATAGGTACAGTTATTGGTGTATTGCTTGGTATACTTCTTTCAACTTAT
GCCACAGAGATAGTTAATTTTATCCAGAATCTTACTGGTAAGCAATTTTTGAGCGCAAGTGTTTATCTTA
TAAATTATATCCCATCGGAGCTTATGTGGTCAGACGTTATAAAAGTTACTTTGGTTTCTATGTTTTTAAG
TTTCTTAGCGACACTTTATCCTGCTTGGAGTGCTTCTAAGGTTCAGCCAGTGGAGGCTTTAAGATATGAA
TGA

>lcl|NC_006570.2_cds_YP_169450.1_360 [gene=lolD] [protein=lipoprotein releasing system ABC transporter ATP-binding protein] [protein_id=YP_169450.1] [location=410734..411429]
ATGAATGATGTTGTTTTAAGTTGCAAAAATGTTTCAAAAAAATATACAGAATTTAAAACTGATATAGCTA
TCTTAAAAGATGTAAACCTCGAGATTAAAAAAGGTGAAAAAGTTGCTATCCTTGGATTATCCGGATCAGG
TAAGACTACATTGCTAAATGTTTTAGGTGGACTAGATAAATGTAGTGCCGGTGAAGTATATTTGATGGGT
GAGAGGTTTGATAATCAATCTGTTAATAAGCGTGCAAAAATGCGTAATAAGCATCTAGGATTTATCTATC
AATTACATCACCTATTACCAGAATTTACAGCTATTGAGAATGTAATGATTCCATTAGCTATCACTAAAAA
ATATACTAAAAAAGAGTCAATAAAACTTGCTAATGAAATTCTCAAAAAAGTTGGTCTTGATCATCGTGCT
GATCATAAACCGGCTGAACTTTCAGGTGGGGAGCGCCAAAGGGTAGCTATTGCTAGAGCATTAGTTACAA
ATCCAAATTGTATTCTTGCAGATGAGCCTACTGGTAACCTAGATAGTCAAAGATCAGAGAGTATATTCGC
ATTGATGCAACAGCTAAGTGACGATTTTGGCACAAGTTTTGTGATTGTCACCCATGATGAGAAATTAGCC
AGCCGGATGAATAAAATTTATCGCTTAGTAGATGGTGAATTAGAGTTAGTTATAAATTCTAACTAG

>lcl|NC_006570.2_cds_YP_169463.1_373 [gene=glyQ] [protein=glycyl-tRNA synthetase subunit alpha] [protein_id=YP_169463.1] [location=433015..433923]
GTGAAAATTTTAATTAGTATGCTTACATTTCAAGAAATTATTTTAAAACTACATCATTATTGGGCATCTA
AAGGTTGTGCAATTGTTCAGCCGTTGGATATGGAAGTAGGTGCTGGAACTTTTCATCCAGCGACAACTCT
ACGCGCGATAGGTCCAGAGCCATGGACAGCTGCGTATGTTCAGCCATCAAGAAGACCTACAGACGGTCGT
TATGGTGAGAACCCTAACCGTACGCAACATTATTATCAATACCAAGTTGTTATGAAGCCATCTCCAGATG
ATATCCAAGAGTTATATCTTGGTTCACTTAGAGAGCTTGGCATAGATCCACTAGAGAACGATATTCGCTT
TGTTGAAGATAACTGGGAGTCGCCAACTTTAGGTGCTTGGGGACTAGGTTGGGAAGTCTGGTCAAATGGT
ATGGAGATTACTCAGTTTACATATTTCCAACAAGTAGGTGGACTTGAGTGTAAGCCTGTGATGGGTGAGA
TCACTTATGGTCTTGAGCGTCTAGCTATGTATATTCAAAATGTTGATAGTATGTATGATATTTTATGGGC
TAATACGCAAAATGGGCCTTTATATTATCGTGATGTGTTTTTGCAGAATGAAGTTGAGATGTCGACTTAT
AACTTTGAAGAGGCAAATGTCGAAGAACTTTTCAAACAATTTGATTTATTAGAGAAAGAAGGTTATCGAT
TAGTTGAAAAAAATCTGCCGATACCTGCTTATGAGTTTGTTTTAAAAGCTTCACATACTTTTAACTTATT
AGATGCGCGTCATGCAATATCTGTTACAGAAAGACAAGGCTATATTCTAAGAGTTAGAAAATTAGCTCTA
GAAGTAGCTAAAGAGTACTATAGTGCTAGAGAAAAATCAGGTTTTCCAGCTTTTAAAAAAGACAATTGA

>lcl|NC_006570.2_cds_YP_169464.1_374 [gene=murE] [protein=UDP-N-acetylmuramoylalanyl-D-glutamate--2,6-diaminopimelate ligase] [protein_id=YP_169464.1] [location=434134..435573]
ATGAAACGTATAAACCAAATATTAAAATTTCTAGATATTAAAACTTTATCTCAAAATAATTTTCAAATTG
AGTCTCTATATTTAGATAGTCGTAAATGTGATAAAAAATCAGTGTTTGTAGCTCTCAAAGGTCTATCAAC
TGATGGTAATAAATATATTGATAGTGTTTTAGCTAAAGGTGTCAGTTTAGTATTAACAGATAGTCATGAA
TTTGAAGACCAGAAACGTGTTTTTTATATTCAAAATTTAAAAGAGAAACTATCGACCCTTGCAAAATGGT
TTTATGATTATGAAAAACCACAAAATATAATAGGTATAACTGGCACAAATGGTAAAACTTCTATCTCTAG
TTATATTGCTCAATTTTTAGATTTAAGTGCACAAAAAACGCTACTTTTAGGTACAAATGGCAATGGCATT
TATCCTGACTTAAGAGAGAGTACACATACGACATTAGATATATTGTCTTTGTATAAAACTATTTCATATT
ATAAAAATTATCAAAATCTTGTGATGGAAGTATCTTCTCATTCATTAGACCAAAAGCGTGTTGAAGGCTT
AGATTTTGATGTTGCTATATTTAGTAATCTTAGCCATGATCATCTTGATTATCATAAGACTATGGATAAT
TATTTTGAAGCTAAGGCAAAATTGTTTCAATTCAAAAGACTTAAAAAAGCTATAATAAATATAGATGATG
AGTATGGTCAAAGACTGTGTAATATGACACAGGTACCGGTTATTACAGTAAGTCTAAAATCTGAATATGC
TGATGTCTATATTAAGCCTAAATCTATAGAAGGTATTAAAACTAAGTTTGAAATGTTTTTTAATGGTAGG
TTTGTTGGTGAATATACAACTAAGCTTATCGGCGATTTTAACTTGATGAATTTGGGCTTGAGCTTTGCAT
CACTTGATGGTGTGTTAGATTGGGATTGTATATTAGATCTAGGAAATATCAAACCAGTGAAAGGTCGAAT
GGAAGTGATAGAGCTAGATAATGATATAAAGATAGTTATTGATTATGCGCATACTCCAGATGCTTTAGAA
AAGGCTTTACAAACCTTAAATAGCTACAATCCAAATAATTTATGGTGTATTTTTGGCTGTGGTGGTAATA
GAGATACTTCCAAAAGACCTATAATGGCACAAATAGCGGAAGAATATGCTAGCAAAATAGTAATTACAGA
AGATAATAATCGTTTTGAGAGTATTGAAAATATATTCAGTGATATTAGAAAAGGATTTAAGTATCCGCAA
AAGCATATTTTTATCACCTCTCGAGAACAAGCTATTAGGTTTAGTATAGAAAATGCTAATCAAGGAGATA
TTATTTTATTAGCAAGTAAAGGGCATGAGTGTTACCTTGATAAAAATGGTGCTAAAGAGTATTTTGATGA
ACGAGAAATCATAAAAAATATGCCGGAGCATTCTCTTTGA

>lcl|NC_006570.2_cds_YP_169465.1_375 [gene=murF] [protein=UDP-N--acetylmuramoylalanyl-D-glutamyl-2,6- diaminopimelate-D-alanyl-D-alanyl ligase] [protein_id=YP_169465.1] [location=436365..437723]
ATGATAAAATCACTAAAACAATTAGCAATCCAAGCAGGCTTAGAATATTTGGGTGAAGATGTATCAATAC
AAACAGTAGCTATAAATTCAAATGAAGTAAGACAAGATTGCTTATTTGTTGCAATTGTAGCTAATCGTGA
TGGACACGAGTTTATTCCAAGTGCTATTGCTAATGGCGCTAAAGCTATTTTAGTTTCAAAAAAACAAGGT
TTAGATATTCCACAAGTAGTTTGTGGCAATACTATCAAAGGCTTACGGGCTTTAGCTAAAGAATATCGCA
AATCACTAACTATGCCAATAATATCTTTAACTGGGAGTTGCGGTAAAACATCTGTTAAAGAAATGATTGT
AACTCTTCTTGGTGAGAGAAAAGTTCATTTTACTCAAGGTAACCTAAATAATTATCTTGGTGTACCAATG
ACAATATTAGAAACACCGCATGATGTCGATTTTGCAGTGATTGAGGCAGGTACAAATGTTGGTGGTGAGA
TAAAAGCTGCAGCTGATATTATCCAACCAAATATAGCGATGATTACAAATGTTGGCGCTTGCCACCTTGA
GAACCTAAAAACGCTGGATGGTGTAATGATAGAAAAAGGGGAGCTTCTCAAAGCTTTGCCAAGAGATGGT
TTTTGTATCGTAAATCTAGATGATGAAAGAATTCCAAATTATGCTAAGCTGCTAGAGTGTAAAAAGATTA
GCTGCTCAATGACTAATCCAAAAGCTGATATTTTAGTATTAGATTATAGGGCAACATCAAATAGTTATGC
TTTTAAGATTAGAATTTTTAGCCAAGAGTATGATTATCAACTACCAAACATTGGCAAGCATAATTTGTTT
AATAGTGTCTTGGCTATTGCTAGTGTAGTTGCTGTGGGGCTTGAGCCTAAGGATTTCTTACAAAATACAC
AAAATATCAAAAACTATAAAGGTAGATTTTCAACTGAAAAACTCAGTGATAAACTAACTCTTATAGATGA
TACATATAATGCAAGTGCTGGCGCTGTGCAAGCTGCAATTGAAGATTTGGCAGAATTTGATGGTAAAAAA
ATATTGGCTATTAGTTCAATGCGTGAGCTAGGAGATGAAGCTGAAAATTATCATCGAAAAATGGGGCAAT
GGCTTAAAAAGGCAAATTTAGATAAGATATTTTTATTTGGTGAGAAAAATTTAATAGATTATGTTTTGGC
AGAATATTCAAACCAGAATGCTAAATATTATGAATCTAAAGACGAGCTAAATAATGATTTAGCTAAAATA
TTGGATGAATATAAGTCACAAAGTACTAAGCTAACAGTCAAAGGGGCTAGAAGTTTTAAGATGGAAGAAG
TTGTCAAATTTGTTAAGGAAAACTTATAA

>lcl|NC_006570.2_cds_YP_169476.1_385 [gene=lpxH] [protein=UDP-2,3-diacylglucosamine hydrolase] [protein_id=YP_169476.1] [location=complement(449760..450476)]
ATGGCTCATAATAAAGATATTTATCTAATTTCGGATCTTCATCTAAACGCTAATCATGCTGAGATGGCTG
ATCTTTTTAAAAAGTTTTTAGATAGCATTACATCTACACAAAACCAGCTTTTTATTCTTGGTGACTTTTT
TGATTATTGGATCGGTGATAATCACAGAGATGACTTTTATCATAAAATCACAAATTGGCTAAAAGAAGCT
TCAGATCAAGGTTTAGAAATATTTTTCATGTATGGAAATCGTGATTTTTTGATTGGCAGAAAATTTGCTA
AACAAAGTGGCGTGACTTTAATTAAAGATCCATACTATATAGATATCTCTAAGCAAAAAATACTTTTCTC
GCATGGAGATCTATTTTGTACAGATGATAAGAGTTACCAAATTTACAGAAAATGGATAGCATACAACCCT
ATCCTTAGATTTATTTTTAGAAGATTACCTTTATTTATTAGAGAATACACCGCGAGAAATGTGCGCAAAG
CAAGTTATGTAAAAAAACCGTAAAAATCCGAATGTTGATGTAACAACTAAAGGCATTGAAAAATATCGCAA
AGACTATGACATTATCATTCATGGTCATACGCATAAAATGGCTGTACATATAGCTGATAACTATACTAGA
TATGTACTTGGCGACTGGTTCAAAGATGGTAACTATATAAAAATCTCAAAAAATGGTGAGATTATGCAGG
TTAGAGCTCTAAACTAA

>lcl|NC_006570.2_cds_YP_169486.1_395 [gene=glnS] [protein=glutaminyl-tRNA synthetase] [protein_id=YP_169486.1] [location=complement(463699..465345)]
ATGACTAGTGAAATTAAAGTAAATAAAACTAATTTTATAAAAAATATCATTAAGAAAGACCTCGAAACAA
ACAAAGTCTCAAGTATTTTGACGCGTTTTCCTCCTGAGCCTAATGGTTATCTTCATATTGGCCATGCAAA
ATCAATATGTCTTAATTTTGGTATTGCACAAGAATTTGATGGTAAGTGTAATCTACGCTTTGATGATACT
AATCCTGATAAAGAAGACATTGAGTATATAAACGCAATCCAAGAAGATGTCGAGTGGCTTGGTTTTAAAT
GGGAAAATCAACCACGTTTTGCCTCTGAATATTTTGATAAGATGTATGAGCTAGCTATATTGCTTATAAA
AAAAGGCAAAGCTTATGTCTGCGATTTGTCAGCAGAAGAAATACGCGCGTACCGTGGCACGCTAAAAGAG
CCAGGAAAAAATAGTCCATACAGAGAACGCAGTATCACTGAAAACCTTGAATTATTTGAAGAGATGAAAA
ATGGTAAATTTGCTGAAGGTAGTAAAACTTTGAGAGCAAAAATTGATATGTCTTCTGGTAATATCAACCT
TAGAGATCCTGCCTTATACAGAATAAAATTCTCACACCACCCTAAAACTGGTGACAAATGGTGTATTTAT
CCGATGTATACATTTGCTCATCCACTTGAGGATGCAATCGAAGAAATAACTCATTCGTTATGTACTTTGG
AATTCCAAGATCAAAGACCATTCTATGACTGGGTTATTGAGGAAACAGAATTTAGTATAAAACCACAGCA
AATTGAATTTTCAAGACTTAATCTTAATTACACAATTACAAGTAAGCGAAAACTAAAATATTTGGTTGAT
AATAAACTTGTCAATGGCTGGGATGACCCACGTATGCCGACTATAAAAGGCTATCGCCGTAGAGGTTATA
CCCCTGAGTCTATACGCAATTTCTGTGAGATGATAGGAATATCTAAACAAGATTCTGTTATTGACGTATC
GGTTTTAGAAGATGCTGTAAGAGATGATTTAAACAAAAGTGTCTTAAGAAAAAAACGTTGTCCTAGATCCA
ATAAGAGTAAGCATAAAAGATATGCCAAATCATCATTTAGATGTACCAAACCACCCACAAGATCCAGAAT
TTGGACGCCGCGAGATCACAATATCTTCACAAATATTCATTGAAAGAGATGACTTTGTCTTCAAGCTAGA
AAAGATATGAAAAAACTTAGTCCTAATGGTAGAGTGCGTTTACTAAATGGCTATGTAATTGAGTGTCAA
GAAGTAATTACTGATACTAGTGGCGAAGTCATAGAACTAAAATGCTCTTATCTACCAGAAACTTTAGGTG
GTAAAAAACCAAATGATGGTATCAAGCCTAATGGCATTATCCATTGGGTTGATGCTAATAATTGCCTTGA
TGCTGAAGTTAGGATTTATGATCGCTTATTTAATGATGAAAATCCAGCAAATGCTGATCGAATTGAAGAT
GTTTTAAATCCAGACTCATTACAAGTTATCAAAAATGCCAAAGTTGAAAGATCTCTTGAAAGTGTCAAAG
CTGAAGAACGCTTTCAGTTTAACAGAGTTGGTTATTTTATTGCTGACTTAAAAGATTGCTCTAACGACAC
ATTAGTATTTAATCATATTGCAACTCTTAGAAACTGA

>lcl|NC_006570.2_cds_YP_169487.1_396 [gene=pckA] [protein=phosphoenolpyruvate carboxykinase] [protein_id=YP_169487.1] [location=465490..467082]
TTGTTAATGGATCCTTCAACCATAGATGAGTATAAGTATCTATATACTTGTGAAAATGTGCATAAAAATC
TCTCTCTCCAAGAATTAGCAAAATACGCACATAATAGTGATGGTTTTATTTGCTACGATAATAAAACTTT
AGTAGTTGACAGTGGTGAAATCAAGGGTCGACTTCCTGATGATAAATATATTGTTGAGACTAAATATGCC
AAAAAAAATATTTGGTGGAGTGAAAATGGCTCAGATAATAAAAGATTAAAAAGAAAAAACTGGAAGCTGA
TTAAACAAAGATTATGTCAAGAGGTAGCAACAAAGGATCTTTTTGTTGTAGATGGTTTTTATAATCATGA
TGAGAGATATACCATAGCAGTTAGACTAATAACTACCCAAGCTTCAGCTGCATATTTTTTTAAGTTGATA
TCGATAACTCCGAGTGAAAAAGAATTACAGTCATTTGAGCCACAATGGGTAATAATGCATTCCCCTCAAA
CACAGATAGAAGATTACCAAGACCTTGATTTAAATTCTTCAAAAGTAATAGCAACTAACCTTAAGCAGCG
AGAAAGTATATTGGTTGGGACTATGTATCTTGCTGAGATAAATAAGGTATTGTTGTCGATTATGAGCTAT
TATCTCTTGCTCAACGATATTGGCGTATTTTACTGTGCTGTCAGTGTTGATGCTGAAGCTAATAGTACAA
TATTTTTTGGTTTGTCAGGTAGCGGTAAAACAACGTTAGCTTTAGACCAAAATAAGAGTCTTGTTGCAAA
TGAGGCTATTGCTTGGACAGAAATGCGTGGTGTGTATAGTCTAGAGTCAGGTTTGACAATTAAATCAGCA
AGTTTTAAAAAAGACGATCCGCGTATCAAACAAGCATTAGCTGGGGACCTTTTGATAGAAAATCCTAACT
TTGATGATAGTCACAATATTATATTTGGTGAGAAAAATAGTTCACAAAGTAATACTTATGTAACTTTCCC
ACGTGAAAATTTTGTTAATGTTATAAACACTGATAACCCTAACACAATAATTTTTCTTGTCAAAGATGCA
AAAGGTGTATTACCAAGAGTTGCAAAACTTAGTAAAGGTCAGGCAATTTACTATTTTTTATCTGGATATA
CTTCTACATCTATAGGAGTAGAGGCAGGTGTTACAGAGCCTAAACCAGAGTTTACAAGTTGCTATGCTCA
GCCATTTCTATTACTTAAGCCAACTAGATATGCAAGTATATTGCGTCAGCGTTTGAAGCATAGTAATGCT
AAAATTTATATGATAAATGTTGGCTGGATTGAGGGTGATTATAAGACAGGTAGAAGGGTTCCTGTTGAAG
AGACAAAACTTATTGTTAACTACTTATTGACTAAACCAGAGAATGTTAGCTTTAAATTTACACGTCAAAA
ATATTTTAACTTTAAAGCTTTAACTTCAATAAATGATAATGGTCAAGAGCTTCAACTTACCAATAATTGG
AGTGATAGTGCTGAATATAAAAAAGAATATAAGTCTTTAGCAAGAGCTTTTATCAAAAACTATGAGCAGT
TTGAAAATGATGATTTTGCACTAAAATATAAAAAATTTGAACCAATTATTTAG

>lcl|NC_006570.2_cds_YP_169488.1_397 [gene=mraY] [protein=phospho-N-acetylmuramoyl-pentapeptide-transferase] [protein_id=YP_169488.1] [location=467157..468254]
ATGCTGATTTATCTTTTTGAATGGTTAAGTCATTATTTCAAAGGCTTAGAAGTTTTTAGTAGTTATATAT
CTGTTAGAATAATTATGATTTCGATAACATCGTTACTCATTACTCTTGCACTAGGTAGACCTATGATCAG
CTGGTTACAAAAAATGCAAATAGGTCAAATTGTTAGAGATGATGGGCCACAAAGTCATTTCTCTAAAAGA
AACACTCCAACAATGGGAGGCGTTTTGATCCTTTCATCAGTTATTATTTCCTGTTTGCTTTGGGGGGATT
TGACAAGTATATATTTATGGATTTTAATTTTAGTGGTTATTTTCTTCGGAGCGATTGGTTTTTTTGATGA
TTATTTGAAGCTAGTACTTAAGCATCCAAAAGGTTTAAGAGCTAAGTATAAGTTTGCTTTGCAATCAATT
TTCTCAATAGTCTTGGCTATAGTTTTATTTTATCTATTGTCTAAGAATGGCCAGATGAGTTTGTCGATAC
CATTCTCAAAGAGTCTTTATATTCCAATAGGTATCGTAATATTCGTAGTTTTGGCGTTTTTTATTATTAA
TGGCAGTAGTAATGCTGTCAATCTTACTGATGGGCTAGATGGTTTGGCTATTGTACCTGTAGTACTTGTG

GCTGCTGGGTTAGGTATTTATGCATATATTGAAACTAATAGTACTTTAGCGAATTATTTATTATTTAATT
ATTTAGGTAATCCTGGACTTGCTGAGGTGGCGGTATTTTGTGCAGCTGTTTGTGGGTCTGGTTTAGCATT
TTTGTGGTTCAATTCCCATCCAGCTGAAGTATTTATGGGTGATGTTGGTTCTTTGACATTAGGTGCTGTG
CTAGGAGTAATTGCAGTGATGGTACGTCAAGAGTTGATATTTTTTATCATGGGGTTGTTATTTGTAGTAG
AAGCTCTATCAGTAATGTTACAAGTAGGCTCATATAAGCTTAGAAATGGTAAGAGAATTTTTAGAATGGC
GCCTATTCATCATCATTTTGAATTGAAAGGCTGGCCAGAAACAAAAGTTGTGATACGATTTTGGATAATT
TCTTTGATACTTTTCTTAATTGGTTTGGCAGCTATCAAGGTTAGATAA

>lcl|NC_006570.2_cds_YP_169489.1_398 [gene=murD] [protein=UDP-N-acetylmuramoylalanine--D-glutamate ligase] [protein_id=YP_169489.1] [location=468254..469504]
ATGTTTAGTTTTTATTTTAATGATAACAAGATAACCAAACTATTAATGGTTGGTTATGGTTCTACTGGTA
AGTCTGTTTGTGATTTTCTTGCGAACTTTATAGATATTACAGTTGATATATCGCAGAATGATGATGAGTT
TGTTAATTATGATTTAAACAGTTATGACTTAATTACAGTCAGCCCTGGGATTCCTCTTAATAAATCTCCA
TATAGAGCCCTAACTAAATTTAAAGATAAAATAGTCAGTGATATTGATATATTCTATCAGTATATCAAGG
ATACTAAAGCTAAAACAATTGCTGTTACAGGATCAAACGGTAAAAGTACAGTAGTAACAATGACTGATTT
TGTCCTCAAGGATCTTGGTTATAAGAGTATTCTTGTTGGTAATATTGGTACCCCAGCTTTAAACAAAATA
GGTGAGAAATTTGATTACTGTGTTGTTGAAGTATCAAGTTTTCAGATAAATTTATTTAATTGTGTTAGAT
TTGATCTAGGTTGTATTATTAATGTTTCACCAGATCATTTAGATAGATACCAAAATTTTGAGCAATACAA
GCAGTCAAAACTTAATTTGGCAAAATTCAGTAACGATTTTTTTGTTTATGATGTGCATAATGGTATTAAG
TATGCTGGCGAATATCAAATAATAAGAGGCGCTATCTACCGTAATTCAACAAAGCTATTAGATATTGTTG
AGACTAAACTTTTTGGTGAACATAATTTAGAAAATATTATTGTTGTGCTAAATATCCTTGATAGGTTAGG
TTTAGATATTAATCAAGCAATAGACTCTATTAAAAAATTTAAAGGCTTAGAACATCGCTGTAAGATTGTA
AAGAAAGTAAATAGTACAACTTATATAAATGATTCAAAAGGTACTAATGTTGGAGCTACAATAGCTGCTC
TTAATAGTATAACAAATTCTAAAAATATCATATTATTATTGGGTGGTGTAGCAAAGGGTGGTGATTTTAG
TTTGATGATAAAATCACTAGATAAATATGTTAAATATGTCTATATATATGGAGCTGATAAAGAATATATT
GAAAGCTATATCAAAGGCTACTGTAAATATCAATTATGTAATAATATGAAACAAGCTTTCGAATTGTCTA
GTCAAAAAGCCAATTCCAATGAGATAGTGTTATTATCTCCAGCATGTGCAAGCTTTGATGAATTTAGTGG
TTATGCACAGCGCGGTGAAGTTTTCAAAATCTTGTTGCTCAGCTAGAGCAAAAAAGTTAG

>lcl|NC_006570.2_cds_YP_169490.1_399 [gene=ftsW] [protein=cell division protein FtsW] [protein_id=YP_169490.1] [location=469514..470719]
GTGTTATATAGATTAAAACTATTGTTGAGTGGCAAAAACTCAAAAAAAGAACGTGTAAGAGCCAAATTAG
AGATTGATATTTCAATTGTCTTTATAATGCTTGGCCTACTTACTTTTGGTTGGGTTATGGTGACTTCTGC
ATCTATGATTGTTGCTTTAGATGACTACAATAATCCTTATTTTTATTCTATCAGACAGGGTTTTTTTGCA
ATTATTGCAATTTTTTTATTTTTATTGGCTTTATTAGTACCAACTAAGAATTATGAAAAGAATTATAATG
CTTTTTTCTTTGTGATGTTGATCGTCTTAGTTGCTGTACTTGTGCCAGGAATTGGGAAAAGTGTCAATGG
TGCGCGACGCTGGATACCTTTGCTAATTATTAATATCCAAGTTGCAGAATTGGCTAAACTTTTGGCGATT
ATATTCTTCTCAGGTTATATTGCTGAAAATCTAAAAAAGATGGCTAATTTTAAAGAAGGAATTCTTAGAC
CAATTACTTTATTGGGTTGTATCGCAATATTGTTATTAATGCAGCCAGATTTTGGTTCTACAGTTGTTAT
ATCAATATGTGTTATGGGAATGCTGTTTGTAGCAGGTAACAAAGTGCGCTGGTATGGTTTACTTTTAGGT
ACTATGGTTATGATGTCTGCGATGCTAGTAATTATATCACCATATCGAATGCACAGAATTACAGGGTTTT
TACACCCATGGGAAAATGCCAATGGCTCTGGCTATCAGCTTGTTCAAGCATTGATAGGTTTTGGACGTGG
TGGCTGGTTCGGTGATGGTCTCGGGAATGGAATTCAAAAGCAATTTTTCTTACCAGAAGCACGTACTGAC
TTTATTACTTCTGTGATTGCTGAGGAATTAGGAGTTGTTGGTTTGATGGTTCTACTTGCAGTGTACCTTT
TTATTGTATTTAGAGCTATGAGTATTGCTAAAATGGCTTTTGAACTTAATAGATATTATCAGGCTTTTCT
TGCTTATGGTGTTGGTTTTTGGATTGCTTTTCAAGTTTTTGTTAATATTGGTGTTAATACAGGACTACTC
CCTACTAAGGGTCTTACATTACCACTGATAAGTTATGGGGGAAGTAGTTTATTAATTATGTGTTATACGC
TAGGTGTCTTGGTTAGAGTTGACTTTGAAAACAAACTTTTAGCAGATACCATTAATCCTCGTTATATTTA
TAAGAAAGTAAAATAG

>lcl|NC_006570.2_cds_YP_169495.1_404 [gene=yccK] [protein=anaerobic sulfite reductase subunit] [protein_id=YP_169495.1] [location=complement(475119..475433)]
GTGGATAACTACAACACAGACGAGCACGGCTTTTTACTTGACTTTGCAAGTTGGGATATGGATTTCTGTA
ATTTAGCGGCTGCACAAGAAAATATCTCCCTTACACAAGAACATATATTTATTATAGAATTCTTGCGTCA
TTTCTATCAGCAAACTAGCAAATCACCAGCAATTAGAGAGCTTGTAAAAGCTTTAAAAGAAAAATATGGC
GAAAAAATAGGTAATAGCTTATATTTACAAACTCTATTTCCAGTATCACCGGCAGTACAAGCAGCCAAAT
TAGCTGGTTTACCAAAGCCTAAACGATGTATTTAA

>lcl|NC_006570.2_cds_YP_169496.1_405 [gene=sspA] [protein=stringent starvation protein A] [protein_id=YP_169496.1] [location=475519..476151]
TTGATGAAAGTTACATTATATACAACGAAGTATTGTCCATATTCTCTTAGAGCAAGAATTGCTTTAGCAG
AAAAGAAAATGTCAACAGATATCGTAGAGGCTGGCGACTTAGAACCAGCCATGATCAAGAAAATAACTCC
TAATGGTGTTTTCCCTGTTTTGATGGAGAAAGACTATAGCATCAATAATAGAAAAGCATTGCTAATTTAT

ATTGATGAAAGATTTCCTGCTCCAAGCTTGCTACCAAATGTAGTTAATGAGCGCATAAAGATACGCTTAT
CGCTTGATAAAATTGATAATGAGTGGTATCCAGTATTAGATCAAATTCGCAAACACAGATCAGATCAGAA
GATGCTTGAATCAATGTTTAAAGACCTAAAAGAAAGTTTATTGGCAATGGAAAAAGCTTTTACTGGCTCA
GAGTTTTTTATATCTTCTGGCTTTACTTTAGCAGATTGTTATATAGCTGCTTTGATTATATGTTTAGAAG
CAGAAGGATTTATTATTGATGATGAATATGGGGCAATTTATGAGTATAAAAAAAGACTCTTTGCTAGAGA
TTCGGTCAAAAAAGCTAACATAAAAGGCGGCGCTGGTGAGTCATTACTCAAAACTCTAAGAACTCATAGA
TAA

>lcl|NC_006570.2_cds_YP_169498.1_407 [gene=holB] [protein=DNA polymerase III subunit delta'] [protein_id=YP_169498.1] [location=477190..478101]
GTGTTATCTCTAACCACACATCAGCAATTAATAGATAATTTTCTTGAGCAAAAAGCTAAGCAAACGTTGC
GTCATGCTTTTATCTTCAGAGTAGAGGACGCTGTATTACTTGATAGCTTCATCAATTCTTTGTGCCAACT
ATTGTTAGGGGAGAAAATACTTAGTTATGATGACTCGCCTTATATCAATATAGCAGCGATTGAAAATGAT
GAAATTAAAGTCGCAGAAATAAAAAAAATAATCAAAAATTGTGAACTGACCGCACATAATAATTTAGCAA
AGATTATTATAATCCAGGGACTCGATTTATTGAACGAATCAGCGGCTAATGCGTTGCTAAAAACATTAGA
AGAACCAACACAAAATACTTTTTTTTTGATGTTTACAAGAAATTATAGTGATGTTTTAGCAACTGTCAAA
AGTAGATCTTTGGTGTATGATATTAAATTTACTCAAAAGATAAGTATAATTACCTAAGTTATACGTTTG
ATATGTCAAAAGATGCTATTGAAAAGTCATTACAAATGACGCGTAATGATATAAATATAATTGCCAAGAT
TAAACTTGAGCAACATTTTTGGCAACTGCGAAATAACTTAATGAAGGTTTTAGCTAATCAGGTTAATTTA
AACGTGTTTCTCAAAGAAGTTAATCCACATTTTAAGGATACTCTTTATTGGTTAACTAGTATGATTATAG
ATGTTTATTGTTATAAGCTTGATGAGCAAAACCAAGGTATCGCAAACTATGATAAACTAGCAATAATAAA
ATATCTTGCAGCTAAATTTGATGCTGATTGTATATATAAGTTGTATTCTAAGGCGCTAGAGGCACAAAGT
TACTTTGTAAAATTTAAAAATGTAGACAAAGAATTAATCTTAGAAAACTTAATATTAGAAATTATAAAAT
AG

>lcl|NC_006570.2_cds_YP_169499.1_408 [gene=yhbY] [protein=RNA-binding protein] [protein_id=YP_169499.1] [location=478112..478390]
ATGGATGTAAAACAACAACAAAAATTAAAAGCTCAAGCACATAGCTTAAAACCTGTTGTACTAATGGGTG
AGAAAGGCCTAACAGAAAATGTAATCTTAGAGATTGATTTGGCTTTGGCATCACATCAATTAATAAAAGT
AAAAGTCGGTCGTTTGCCTAAAGAAGAGAAGCAACAAATTGCTAGTGAAATTACTCAGGCGACAAGGTCA
GAGCTTGTACAGATTATTGGTAATATTCTAGTCTTATACAGAAGAAATCCAAACAAAGAAAAAATATAG

>lcl|NC_006570.2_cds_YP_169500.1_409 [gene=hemB] [protein=delta-aminolevulinic acid dehydratase] [protein_id=YP_169500.1] [location=478398..479372]
ATGAGCTTTCCAATATCACGTCCACGTAGACTTAGGGTTACACAAGCTTTTCGCGATATCGTTGCTGAGA
CAACTTTAAGCCTTGATGACTTAATGTATCCAATATTTGTTGTGCATGGTCAAGGAGTTAAAAAAGAAAT
TTCAAGTATGCCAAATCAATATCATTGGTCAGTTGACATGCTTGATGAGTTAGTAGATCAAGTTGTCAAG
GCTGGAATAAGGAGTCTAATGATATTTGGCGTGCCAAAAATCAAAGATCTTGTTTCATCGGAGAATTATG
ATCCTAATGGAATAACACAGCAAGCAATTAGAAAGATTAAACAACTTGCTCCTGAGCTTGTGATAGCTAC
AGATGTATGTATGTGTAGTTTTACACCACATGGTCATTGTGGAATTTTAGATGAGCATGATTATGTTGAT
AACGATCAAACATTAGAAATATTACAAAAAACGGCTGTTTCTCATGCTCAAGCTGGAGCAGATATTGTAG
CACCAAGTGGGATGATGGATGGTATGATAATTGCGATGCGTCAGGCACTAGATGAGGCAAGCTTTGAGAA
TGTGAGTATTATGTCATACTCAGTTAAATATGCATCGGCATATTATGGACCATTTAGAAGTGCTTGTAGT
TCGTCACTTAAGGGAGATCGTAAAACTTATCAGATGGATTATCGCAATAAGAAAGAGGCAATCTGTGAGG
CGCTTGCAGATATTGAGCAGGGTGCAGATTTTATTATGGTTAAACCAGCTTTGAGTTATCTGGATATTGT
CAATGAGTTAAGTCATATTATAGATTTACCAATAGCAGCATATCATGTCAGTGGTGAATATGCGATGATA
AAAGCTGCTGCTAGTGCTGGTTTAGTTGATGAAAAAGCAATCACTATAGAAACACTCATATCAATGAAAA
GAGCAGGTGCTAAAGTGATACTTACATATACTGCGTTGGATGTGGCTAATTGGTTAAAGGTTTAA

>lcl|NC_006570.2_cds_YP_169504.1_412 [gene=argS] [protein=arginyl-tRNA synthetase] [protein_id=YP_169504.1] [location=complement(481989..483734)]
ATGAATATAGAAAATTATTTATCAGAAACTCTTGCAAAGGTGTTCCAAAAGCTAGGTTATGCAGAAAGTT
TTGCTAAAGTTGTAACCTCAACTCGTGAAGATGTTAGACATTTCCAGTGTAATGGCGCTATGCCATTAGC
TAAATTCGCCAAAAAACCGCCTTTGGCAATTGCCGAAGAGATAGTCGAGCATATTGATGCTGAAGATATC
TTTGCCAAATTAGAGGTTGCTAAACCAGGCTTTATCAATATAACTTTAGCACCAAAATTCTTAGCTGATA
CTACAAATAGATTTTTAAATTCTAATAAATTTGGTGTACAAAACAACTTACCTAATAGGAAAGTTGTGCT
TGATTTTGGTGGTCCAAATGTTGCTAAGCCGATGCACGTTGGACACATTAGATCTGCACTTTTAGGTGAT
GCCTTACAAAGAATTCATCGCTTCTGTGGTGATACGGTAGTCTCTGATGTACATTTAGGTGATTGGGGTA
CGCAAATGGGTATGCTTATAGAAGAAATAAAACTACAATCGCCTCAACTTGTATATTTTGATGAAAATTA
TACAGGTGAATATCCTACGGAATCACCAGTAACTGTCCAAGAGCTAGCAGAAATCTATCCGCGTGCCTCG
AAGAGGTGTAAATCTGATATAAATGAAATGGAAAAAGCTAGATTAGCTACTTTTGAATTACAACAAGGTC
GTAGAGGTTATGTTGCACTTTGGCAACATTTTGTGAGAATCTCTATCGATGCAGTTAAAAAAGATTTTGA
TAGTTTAGATGTGCATTTTGACTTATGGTTAGGTGAAAGTGATGCTAATAAATTTATCGATGAAATGATT
AGTTACTTTCAAGCTAATAATTTTATCTATGAAGATGAAGGTGCTTGGGTTATTGACACAAATAAGGATG

GAGTACCACCTCTAATAGTCATCAAAAAAGATGGTGGTGTAATGTATGGTACTACGGATCTAGCTACTTT
ATGGCAGCGTAGCAAAGATCTTGATCCAGATGAAATAATTTATGTAGTTGACAAAAGACAATCTCTTCAC
TTTAAGCAAGTTTTTAGCGTTGCTGAACGGACAAAAGTTGTCAGCGAAAAATGCAAACTTAAACACGTGG
CTTTCGGCACAGTCAATGGTAAAGATGGACGCCCTTTCAAAACCCGTGAAGGTGGCGTAATGCATTTAGC
AGATTTGATTTCTCAAGCAAAAGAATATGCCAAAAATAGAATGCCTGATGAGAATGATGATAGCATTATT
AATCAAATAGCTATGGCAACAATTAAATTTGGTGACCTAATCAATAACTATGCTAATGATTATTTCTTTG
ATTTAGAAAAGTTTGCACAGCATGAAGGTAAAACAGGTCCTTATCTTTTGTATACTGTAGTTAGAGCAAA
ATCGATCCTTAGAAAAATATTTGGTGACAACTATGATATCAAATCTCTAGCAAAAGATTATAAAGTTGTA
AATGCTCATAATGAATACGAAGAAAAACTCCAACTTCAACTAATACAATTTCCTATCGCTGTACAAAGAG
CTTATGAAAATTCGCAACCTCATCATATTTGCGAGTATGCTTACTCATTAGCTAATAGTTTTAACAAATT
CTACGTTAATTGCCCTATTAATAATCTTGACGATGAGTCATTAAAAAAAGCAAGAATTGCACTATGCATG
GCAACTGTCAAAGCTATGACAATCGCCTCAGATTTAATAGGCATCTCTATTCCCGAGAGAATGTAA

>lcl|NC_006570.2_cds_YP_169505.1_413 [gene=ostA1] [protein=organic solvent tolerance protein] [protein_id=YP_169505.1] [location=483860..486466]
ATGTTAAAGGGGATTCATAAGTATCTATTGATGTGTTTTGGTACTGTATTGTTTACAGTGCAAGCCAATG
CTGCACGGATAATGAGCAATAATCCTATTAAAGAAGATTGGCAATGTAAGGTAGTTGATGGTGAGTGGAG
TTGTAAACGAGCAAAGAAACCAAAAAGTGTCTTTGATAAAAAGCTTACTAAAACTGAAAAAGAAAAAGCT
CTTGCTGATGATCTGGCATGGGTCAAGAAACCATCATATTTTGTTGGTGGATACTATAGCAATGATAATC
AGTTTACCAAAGCTTTGTGTGAGTCTAAAAAGACAGATCTTAGTTATGAGAAGTCTGAGTTTGACAATTA
TGGTACATTAATAGCGTCAGGAAATGTCCAGGTGCTACAATGTGATCAGGAATTGTATGGTAATAATGCA
ATAATAAATTTAAATAGTAATAATAGTGCTATAAGATCATTAGTGATGGCTGGAGATGTAATCGTTAAGC
AACCATCGACAGGTATAGTCATCCGTACTACAGAGTTAGATGCTGATATGAATAATGGTACTTATAGTAC
TGGTGAAGCATATTTTAGATTGGCGCGTGAAATGCCAAAAACTAGAATATATGATAAAGAACATTTTAGT
GGCTACTTGCGTGGTTATGCTAAAACATTCAAAAAAGAGTCATCAGGAGATATAGTACTTTCTGATGGAT
ATATTACTTCTGGCGATCCGTATGATAATGCTTGGAAGATTACTGGTAATAATATAGATATTGATACCAA
CACTCATATGGCGTATGTCAAAAATGGTTATTTTGAGATTCAGGATATTCCAGTAATGTATATACCATAT
TTTTCACATCCAATAGATGATAGAAGAAGATCTGGTTTCTTATATCCAGGTTTTGTACAAAATGCTAACT
CTGGTATTGGGATATCTGTACCTTATTATTTTAACCTTGCGCCTAATTATGATTTGATGTTACAAAGTGT
TATATGGTCTCAAAGAGGTATCATAGAAAATGGTACTTTCCGTTACATGACTAAATATTTCCAGGGTCAA
TTTGAGGGTTCATTAGTACCTTATGATTTTAAAGAAGGAAAAATGCGCGGCTCTTTTACTTTGTCAACTA
CAGGTCAGTATGAAAATATAAACACAAATTTTAAGTATGAATATGTTAGTGACCAAAACTATTATAATGA
TTTCTCAGCAGGAAATGTTAATTTAGTTACTAAGACATTACTGGATAGAGAATTCGATCTAACTTACACT
AATGACTATGTTGATTCTGGGTTAACAGTATTAGACTATGGTGTAGTAAACCCTTTATTAACCGTTGATA
ACACACCTTATGCTAAGCTACCTGAAGTTAAGTTAAATCTTACGTCGGATGGTTATACACCAGATTATTT
GACTTTAAGTGCTCAAACTCTTAATACATTTTTTTATAAAACTGCTGGGCCAGCTAATACTAATCCTGGT
GCTCCTCAAGGAACAAATGTTAATGCATTTAGAGCTTATGAATCTCCAAAAATAGCATTCAACTTTAATA
AAACATGGGGGTATCTAAATCCTTCATTAGAGGTGCCTATTCGTTATTATCAACTAAAAAATAGCCCTAC
AGATACGATACAGTTTGCTAATAGTAGTGTTACTAGTGTACTACCAATATTTAATATTGACGCTGGGGCA
TATTTTGATAAAGATTATACTAATGAAAATGGGACGTATACATCAACCTTACATCCTAGACTATTCTATA
CTTACATACCGTACCAAGATCAAACAAATATACCATTATTTGATACAAGTTTACAAAATGAACAATATAT
GCAGATGTTTCAAGTAAATAGATTTACTGGTTATGATAGAATCAACAATGCTAATCAGCTAACATATGCG
ATAGAAGCTTCAACAACTAATCAAGATAATGGTACTACTCTAGCGTCTGCTAAAATTGGTCAGATGGCTT
ATTTTGCTGATAGGAAAGTTAATTTGTGTCAAGGAAATTCTGCTTGTCCGAATCCGGGTTTAATGGATCC
TTTTTCAACAGATACTTTCTCTCCAATTATGAGCTCATTTGAGTTTCAAGTAATGAAAAATATTTACTTA
TCAGCTCAAGTGAATTATAGGGTTAATCAACAAAACGTTGACTATCAAGTCTATCAATTGTCATATAAGG
ATGAAAATGAGAATATTTTTAACGTTTCATATAATAATATTGCAAATAACTGGAACTCATTGACTCAACA
GCAAATAGCAGAAGGTGCTAAGCCTCAACCACAAGAAACTATTACTCTTTCTACAGTGTTAAATATTACT
GATCATTGGGGTATTGCAGCGCTATGGAACTATAATTTCCAGCAAAAACAAATAGCTAATATATTTGCAG
GGCTGCAATACAATGCCAAATCTTGGGCGGTTAGAGCATTATGGCAGAAGACCGCGTATACTAACCAAGA
TCCTAATAATCCAACATTACTTGGCCCACTAGTTAATACTTATATGTTTGAATTTGAACTAAAAGGTCTA
GGAGGTATTGGCAATACTAGTGATATATCTTCACGTTTACAACAAATAAATGGTTATCAAGTAGGAGAGT
GGGGAAACGGTATATAA

>lcl|NC_006570.2_cds_YP_169506.1_414 [gene=surA] [protein=peptidyl-prolyl cis-trans isomerase] [protein_id=YP_169506.1] [location=486448..487869]
GTGGGGAAACGGTATATAATGAAGAAGCTAATAACAATCTTTTTGTTGATGCTAATGTTAAATAATGCTT
ATTCAGATATCTCTTCATCTATGTTTCAAAATGCTTTTAACTCGGGTATTGATGCAACATCTCCGGTATC
AATGAATGTATCAGATAAAAAAATATCTTGTGAATAAGACGGTAGCGATAGTAAATAGTAGACCAATTACA
TCTTTTGAGCTGGATCAAGAGCTGGCTAAGCTAGAAGCTATGCAGCCAAATTCAGCCTTTAATACAGATC
CTCTAAAAAGACAAGCATTACAAGATTTGATATCTCAGAGTGTTTTATTACAGCTTGCAGAGCGCAATAA
TATTATGATATCTAATCAACAACTAGATAGTGCTATACAAGATATTGCAGCAAAAAATGGCGTTTCAGTA
GAGTCTTTAAAGCTTAATGTCGAAGCAGCCGGAATGTCTTTTGATAGTTATAAAAAAAGAATAAGAGATC
AGTTGATGATAAGTCAACTACAACAGCAGGCTATAGCACAGCAAGTATATGTCTCACCAGAAGAAATACA

AAAATATATTAAAAAACATCAAAAAGAATTTGATAGGGAAATGTCGCCTGTTAAATTATATACACTTAAA
AATCTAATTGTAGCTTTGCCAGATTCTAAAAAAGCACGCCAGAAGAAAATAGATTTGTTTAAAAAACTAG
CTCTTGCAGTTAATGATGGCAGTATTGATTTTTCTGAAATTGTCAAACAGTTTTCCCAAGCGCCAAATGC
AGTTTCCGGAGGTATAGTTAGTCAACAGGTTAAATTTGATTCAATACCAGATATATATAAGGAATATATT
AAAGAACTTAAAAATCATCAAGTTTCACAACCATTTATAGTTAATCATACATTACAGATGATATATATCT
ATAACATTGATGAAAAAGCACCAATTTTAAGCAAAAAAGTAACAAAATATTATGTTTACGCAATAGAAAT
TAAGCTTGATGGTGGTATGAATGAGGACGGTGCAAAAAGTTCACTTGAAAGAGCAAAACTTGCTATTGAG
AGTGGGCAAGAATTTACTAAAGTTGCGCTGAAATATAATCAAGATTACGATCATCCAAATGGTAATTTTA
AATGGGTATCAGAGCTTGATAGTCCGCCTTCACTACCTCCTGCTGCGTTTGCACAGCTTAAGCAATTAAA
AGAAAATGAGTTGTCAGAGCCTTTTCAAGCTGATGGTAGAACTTGGATGATTATCAAATATACCAAAACT
AAAGAGTATGATGCTGCTGAGCAGCTTAAAGAACAAAAGGCTCTAGAAGCAATATTTTCTGAAAAAGCTC
AAGAGATTTATAAAACTTGGTTAACGTCGATGAAAGATGATGCGTATATTGAAATACTTGAAGATGATTT
AAAAACACCTGAACTTTACTAA

>lcl|NC_006570.2_cds_YP_169510.1_418 [gene=accB] [protein=acetyl-CoA carboxylase, biotin carboxyl carrier protein] [protein_id=YP_169510.1] [location=489944..490417]
ATGGATTTATTAAAAGCAATTGATAGAGTGGCTGAGATTCTTAACTCAAGTGATATAAAGGAAATCAGAA
TTAAAGATGGTGGTTCAAGCATTTTTATGACAAAAAATAATACAGCTGCTATTACAAGTGTAGTTTTTGC
TGCACCAGTTGCTAGCAATGTTGCTTCAGCAGCTCCAGCAGTAGCTACTGCTGCTACTTCGGCAGCAGCT
CCTAAAGTAAATCTGGCTGAAGAAATAAGCGGTGAAGAGATCAAGTCTCCAATGGTAGGTACTTTCTATG
GCGCATCTTCACCTGATGCTGCTCCTTATGTTAAAGAAGGTCAAGAAGTCAAAAAAGGCGACGTATTGTG
TATCATCGAAGCAATGAAAATCATGAACAAAATTGAAGCAGAAAGAGCAGGTAAAATTGTTAAAATCATC
GCTAAAGATGGTGAGCCTGTTCAATTTGATCAACCTCTATTTATTATTGAATAA

>lcl|NC_006570.2_cds_YP_169511.1_419 [gene=accC] [protein=acetyl-CoA carboxylase biotin carboxylase subunit] [protein_id=YP_169511.1] [location=490482..491837]
ATGATTAAAAAAGTACTGATTGCCAATAGAGGTGAAATAGCTCTTAGAATTTTAAGAGCTTGTAGAGAGT
TGGGAATTAAGACAGTCGCTGTGTATTCTACAGCTGATGCTAACCTTATGCATGTCAAACTAGCTGATGA
AGCAGTTTGTATAGGTCCTCCTGCTCCTAATCTTAGTTATCTAAATATCCAAGCTATTATAACTGCCGCT
GAAATTACTAATGCTGATGCGATACATCCAGGTTATGGTTTCTTATCAGAAAATGCAAAATTTGCTAAAG
CTGTTGAAGAAAGTGGCTTTATATTTATTGGTCCACGTGCAGAAAGCATAGAGATAATGGGCGATAAAGT
TGAAGCTATTAGATATATGAAAAAAGCTGGTGTTCCATGTGTGCCAGGTTCAGGCGGTCCATTAGGCAGC
GATGAAAAGAAAAACTTAGAAATTGCTGAAAAAATCGGCTATCCAGTTATTATTAAAGCTGCTGGTGGCG
GTGGCGGTCGCGGAATGAGTATCGTTAGAAAGAAAGAAGATCTTATTAGCGCTATTTCTCTAACAAAGAG
TGAAGCGAGAATAGCTTTCAATAATGATATGGTTTATATGGAAAAATTCTTAGAAAACCCTCGCCATATT
GAAATTCAAGTTTTTGGTGATGGTGAAGGTAATGCCGTATATCTATTTGAAAGAGATTGTTCTACTCAAA
GAAGACACCAAAAAGTTATTGAAGAAGCTCCAGCAATCGGTCTTTCCGACGAGGAAAGAAAGCGTATTGG
TGAGCAGTGTGTTAGCGCATGTAAGATATTAAAATATCGTGGTGCTGGTACTTTTGAGTTCTTATATGAG
AATGGTGAATTCTACTTTATTGAGATGAATACTAGAATTCAAGTGGAGCATCCTGTTACTGAGTCAATAA
CTTCTACAGATCTTATCAAAGAGCAAATCAGAGTTGCTAAGGGTGAAGGTCTAAGTTGGAAACAAGAAGA
TATTGCTATAGTAGGTCATGCAATTGAGTGTAGAATAAACGCCGAAGATCCTGAAAGAATGATTCCTTCA
CCTGGTAAGATCGATATGTATCATCCGCCAGCAGGACCTAGAGTGCGTGTGGACTCACATATATATTCAG
GCTATGTTGTTCCGCCAAATTATGACTCAATGATAGCTAAAGTTATCGTACGTGGTCATAATAGAGAAAC
AGCATTACAGAAAATGCGCGCAGCTTTAGAAGAGATGGTGATTAATGGTATTAAGACAAACATACCTCTT
CATCAAGAAATTCTAAATAATGAAGATTTTATTAAGGGTGGAACTAATATCCATTTCTTAGAGAAATTCT
TAGAACAAAAAAACAAAGCTAAATAA

>lcl|NC_006570.2_cds_YP_169515.1_423 [gene=birA] [protein=transcription repressor BirA] [protein_id=YP_169515.1] [location=complement(494680..495462)]
ATGAAAAATCACCGCCTCATAATAACACAACTTAACAAATATATAGATGATATAAAAGTTGAATATTTTC
CAACTATAGACTCAACAAATGACTATTTCTTAGCAACAAATTTCACACATAAATATCATTTTTGTTATGT
GGATAAGCAAACAAAAGGTCGTGGTCGTCGTGGCGATAAATGGATTTCTGAAGATAAAGATAATATCTAC
TCAACATTAGCGTTTCATTGTGATTTTGCAATCACAGCTGACTCACTTAAAAGTGTCAAAATAGCTCTAG
GAGTCTTAGCAGCTATCAAAAAATATATTCCCAAAAATCTACAACAATACCTAAAGATAAAATTACCAAA
TGATATTTATTTTCAAGATCAAAAACTCGCTGGGATACTAATAGAAACAAAAAATATCAAAAAAGATAGT
TTTGACATTATTATAGGAGTAGGTATAAATGTTAATATGACAAATACTGATGAAAATATTGATCGCGAGT
GGACATCTTTATCAAACATAAATAATAAACAATTAAACTCTTCTAGAATCATAGTTGATTTAGTTAAAAG
CATTATTGATAGCTTTGATATGAATGATGCCACTGCACTAGCACAATTAATTAGTTATGATTATATTTTA
GATAAGAGAATTACTTTTAACTATGCAGATCAAAGTTACCAGGGTATCGCCAAAGGTATTTCTGAGGATC
TGAAACTAAAAATTATAGATGAAAATAATAATTATTGTGAATTTGAACTAGCTAATATTAATAAAATTAG
AGTTATAAAATGA

>lcl|NC_006570.2_cds_YP_169522.1_430 [gene=FTT_0484] [protein=hypothetical protein] [protein_id=YP_169522.1] [location=503405..504124]

ATGAAAAAATCAAAAATCTTAGTTTCGATAGCAATTGCAGTTTTTGGAGCTGCAGGAACATCTTTTGCAG
CAGAAGCTGATAAGTCTAGTTCAATAGATAAACTATTACCAGAAGGTTACTGGGTTCAGTTTGATGAGGA
TAACGATGCTGGTCGTGGTATGCCTAATGGAATTATTCATACATATTTTGCTAAGAATGATAAGTATGGT
AAAAAAGGTACATTACAGATGGAAATCGTAGTGCCACTAATGTATGTTGACTCTTCTGGTAAGCCTACAA
AGCCAAGAGCTACTTGCTATAATTGTTCTAATGGCTCTTATAATGGCTTTAATTACAGGGGTGAAAATGC
TCCTCTAGAAGGTTTTGTATTTGCTGGCAATATGCAGCCTGAAGCTGGGACAGCTGTTGCTAACCAAAAG
AGTGATATCTATAAAAATGGTGGAGTAATAAATCCAAATGATGGTAAGGTTTATGCTTCAGAGGCTCAAG
TCCAAGATGGTGGAACTACTATGTTTGCCAAAGCCGCTTATCTTGTATGGGGTAAAGAGTTAGGTAGCAA
ATCTGCGCATTGGAAGAGAATAACAAAAGCTGACTATGAAAAAGTCAAAGCAGATTGTGGTGTAACAGCT
GATGGTCAGTATACTAATAGTGATGTAAAAGTCACTGCTACTTGTACTAATTACCCAGTTGCTCAGTTTG
GAGTTAAAAGTCCCGTCTAA

>lcl|NC_006570.2_cds_YP_169545.1_450 [gene=gyrB] [protein=DNA gyrase subunit B]
[protein_id=YP_169545.1] [location=530401..532818]
TTGTTAATGTCTGAGAATAAAGCTTATGACTCATCGAGTATTAAGGTACTTAAAGGTTTAGATGCTGTTA
GAAAAAGACCTGGGATGTATATTGGTGATACCGATGATGGTAGCGGCTTACATCATATGGTATTTGAGGT
TGTTGATAATGCTATCGATGAAGCCTTAGCTGGTTATTGTGATGATATTAAAGTTATTATAAATAAGGAT
GGCTCTGTATCTGTATCAGATAATGGTAGAGGTATTCCTACAGATATTCATAAAGAAGAAGGGCGCTCTG
CAGCAGAGGTTATTTTGACAGTATTGCATGCAGGTGGTAAGTTTGATGATAACTCTTATAAAGTATCAGG
CGGTTTACATGGTGTTGGTGTATCAGTTGTAAATGCGCTTTCAGAACAATTAAAGTTAACAATTTATAGA
AATGGTATCGAGCACTATCAAGAGTATGTTCATGGAGTACCACAGTTTCCATTAAAAGAAGTTGGTTCAA
CAGATAAGACCGGAACACTAATTACTTTTAAACCGAGTAAAGAAACGTTTTCATTTGTTGACTTCGATTA
TGATATCTTGATGAAGAGAATTCGAGAGTTATCTTTCTTAAACTCTGGCGTCAAAATTGAACTTATTGAT
AAAATTAATAATAGGTCAGAAATATTTAAGTATGATGGTGGTATAGTTGCTTTTGTTAAGTATTTGAACA
ATAGCAAAAAACCTATTCATGAAAATGTTATAGCTGTAAATGGTATCAAAGATGATATTCAAGTAGAGCT
TGCTTTGCAATGGAATGACTCATATAAAGAATCAATTTTTTGCTTTACAAATAATATCCCACAAAGAGAT
GGTGGTACGCATTTATCTGGCCTTAAGGCAGCTGTTACTCGTACGATGAATAATTATATTGAATCAGAAG
GTTTAAATAAAAAGCTAAAAGTTACTTTAACAGGTGAAGATACGCGCGAAGGTTTAGCTGCAGTTTTATC
TGTGAAAGTTCCTGATCCAAAATTCTCATCACAAACTAAAGATAAACTAGTATCATCTGATGTTAAATCA
GCTGTAGAGTCATTAGTTAATGAAAAACTACAAGAGTTTCTTTTAGAAAACCCTAAAGAAGCTAAAATTA
TATGTGAGAAAATTTTAGACTCAGCAAAAGCTCGTGAAGCTGCTCGTAAAGCCCGTGATATGACGCGTCG
TAAGGGTGCTTTGGATATTGCTGGCCTACCTGGTAAGTTAGCAGATTGCCAAGAAAAAGATCCGGCATTG
TCTGAAATTTATCTTGTTGAGGGAGACTCTGCAGGAGGTTCAGCAAAGCAGGCTCGTGATCGTAAAACGC
AGGCGATTTTGCCACTAAAAGGTAAGATTCTCAATGTTGAGAAAGCACGTTTTGATAAAATGCTTGGTTC
TCAAGAGGTAGCTACGCTCATTAAAGCTCTAGGTTGTGGTATAGGTGCGGAAGATTATAACCCTGATAAA
ACTAGATATCACAAAATTATCCTAATGACGGATGCTGATGTTGATGGCTCGCATATTAGAACACTTCTTT
TGACATTTTTCTATAGACAAATGCCAGAGCTTGTTGAGAGAGGGTATTTATATATTGCTCAACCACCTTT
ATATAAGGTAAAAAAAGGTAAGCAAGAAACATATCTAAAAGATGAAGATGCTTTGGCAGAATATCTTGGT
AATATAGGTCTAGAAGGCGCGTGTATCTATTTAAGTAATGGCAATGTTATTTCTGGTCAAGTTTTGGCTA
ATTATTATGAGCTTTATCAGAAGTCACAAAAAGTAATTAAAAAATATACAAAAACGTATCCTGAGAAGCT
TCTTAGAGTTATGGCATATGGAACTAAGTATGTTGATGAAAGTGCGGATATCTCTGAGTGGTGGCAGAAA
ATTGTTGAAAATTGTAATCAAAAGGCTTTAGCATATGAGCGCTTTAAGCTTATAGAAACAAAAGATATAG
GTGAGGATGGTAAAGAGAATATATCATATGGAGTTAATCATTATATTAATGGTTATGATACTGACTATAT
TGTTAAGAGTAGTTTCTTTAGTACTAAAGATTATGAAGATCTAGTTACATATGGAGATGTTTTATCGGAT
ATATCTTTTGAAGGAGCTTATGTAGAGAGAGGTGCTAAAAAAGAATATGTTGATGATTTTGAATCAGCAA
TTGATTGGTTGCTTAAAGAAGCTAGAAAAGGTAATGATGTACAGCGTTATAAAGGTCTAGGTGAGATGAA
TCCTGGTCAGCTTTGGGAAACTACTATGGATCCTGATAATAGAGTTCTTCTACAAGTATCAATAAAAGAT
GCGGTTGAAGCTGATGCTTTATTTACTACTCTGATGGGTGATGAGGTTGAGCCACGCAGAAACTTTATTG
AGTCAAATGCTCTTAATGTGGTTAACTTAGACGTTTAA

>lcl|NC_006570.2_cds_YP_169561.1_462 [gene=grxC] [protein=bifunctional
gluaredoxin/ribonucleoside-diphosphate reductase subunit beta]
[protein_id=YP_169561.1] [location=complement(553228..554460)]
ATGGTCGAAGTAAGAATATATACTAAAACAAATTGTCCTTTTTGTGATCTTGCAAAAAGTTGGTTTGGTG
CTAATGATATCCCTTTTACGCAGATATCACTTGATGATGATGTCAAAAGAGCAGAATTCTATGCTGAAGT
AAATAAGAATATTCTTCTTGTAGAAGAACACATCCGAACCGTACCACAGATATTTGTTGGCGATGTACAT
ATCGGCGGTTATGATAATCTTATGGCTAGAGCTGGTGAGGTGATTGCACGTGTTAAAGGTTCATCGTTAA
CTACTTTCTCAAAAACATACAAGCCTTTTAACTATCCTTGGGCTGTTGATCTTACTGTAAAACATGAAAA
AGCTCACTGGATAGAAGATGAAATTGATCTTTCTGAAGATGTCACAGATTGGAAAAATGGTAAAATAACC
AAAGTTGAGAAAGAATATATTACTAATATTCTGAGGCTTTTTACTCAGTCTGATGTTGCAGTTGGTCAGA
ACTATTATGATCAATTTATTCCTTTATTCAAAAATAATGAAATAAGGAATATGCTTGGTTCATTTGCAGC
TCGTGAAGGTATTCATCAAAGAGCATATGCGCTGCTAAATGACACTCTAGGCTTACCTGATTCAGAATAT
CATGCCTTCTTAGAATATAAAGCAATGACTGATAAAATTGATTTTATGATGGATGCAGACCCTACTACGC
GTCGTGGTTTAGGATTATGTCTTGCTAAGACGGTATTTAATGAAGGTGTAGCTTTATTTGCATCTTTTGC

AATGCTGCTTAACTTTCAACGCTTTGGTAAAATGAAAGGCATGGGTAAAGTAGTAGAATGGTCAATTCGT
GATGAATCTATGCATGTCGAAGGTAATGCAGCACTATTTAGAATTTACTGTCAAGAAAATCCTTACATAG
TTGATAATGAATTCAAAAAAGAGATCTATCTAATGGCTAGTAAAGCTGTAGAACTAGAAGATAGGTTTAT
TGAACTAGCTTACGAACTTGGTACTATCGAAGGACTCAAGGCTGATGAAGTTAAACAATATATCCGTCAT
ATCACTGATAGACGCCTAAATCAACTTGGACTAAAAGAAATCTATAATATTGAGAAAAACCCACTAACAT
GGTTAGAGTGGATACTAAATGGTGCAGATCACACTAACTTCTTCGAAAACCGTGTCACCGAATATGAAGT
TGCTGGTTTAACAGGTAGTTGGGACGAGGCTTATAGCGCCTAA

>lcl|NC_006570.2_cds_YP_169562.1_463 [gene=grxA] [protein=glutaredoxin]
[protein_id=YP_169562.1] [location=complement(554467..554727)]
ATGAAAGTAAAAATATATACTAGAAATGGTTGTCCATATTGTGTTTGGGCAAAACAATGGTTCGAAGAAA
ATAATATAGCTTTTGATGAAACTATAATTGATGATTATGCTCAACGTTCTAAGTTTTATGATGAAATGAA
TCAAAGTGGCAAAGTTATCTTCCCTATTTCAACTGTGCCACAAATATTCATAGATGATGAACATATAGGT
GGCTTTACAGAGCTTAAGGCTAATGCTGATAAAATTTTAAACAAAAAATAG

>lcl|NC_006570.2_cds_YP_169563.1_464 [gene=nrdA] [protein=ribonucleotide-
diphosphate reductase subunit alpha] [protein_id=YP_169563.1]
[location=complement(554731..556503)]
ATGTCTAATCAAAGCTATCTTGGCATTAATATTGATTTATCTAGAGATCGTGAGCTTTCAGAACAAGCTC
AACAACTACTGACAAACTATTATTGCTTACAAAATGAACCCTCTCCTCAATATGCTTTTGCACGAGCTGC
TGTTGCTTATTCTTTTGGGGATATGAACCTAGCTCATCGCATTTATGATGCTGCTGCTAAAGGTTGGTTT
ATGTTTGCTTCACCTGTGCTTTCTAATGCACCACTTCCAGGTGTAAAAGTAAAATCTTTACCTATTAGTT
GTTTTTTATCTTATGTGCCAGATTCATTAGAAGGTCTTATCGAGCATTCATCAGAACTTAGATGGTTATC
AGTAAAAGGTGGAGGTGTTGGAGGACACTGGTCTAGTGTTCGCGCGGTATCTGATAAAGCTCCAGGACCT
ATCCCATTTATGCATACAGTTGATGCTGATATGGTTGCCTATAGACAAGGTAAAACTCGTAAAGGTTCAT
ATGCTGCATATATGGACATATCACACCCTGATATAATTGAATTTTTAGGTATCCGTGTGCCAACAGGTGA
TGTAAATAGAAAATGTCTAAACCTACATCATGCTGTAAATCTGACTGATGATTTTATGCAAGCAGTTGCA
AATGACCAAGACTGGAAACTTATTGATCCAGATGACAAAACAGTAAGAGATGTTATCAAGGCTCGCAAAA
TTTGGGAAACCATATTAGAAACTCGTTACCGTACAGGTGAACCATACTTAAACTTTATTGACACAGCTAA
TAGAGCTTTACCAAAATCTCAAAAAGACCTGGGCTTGACAATCAAAGGTTCAAATCTATGCAATGAGATT
CATTTAGTTACAGATGAAAAACGTACAGCAGTATGCTGTTTATCTTCTGTAAATTTAGAAAGATACGATG
AATGGAAAGATACCTCACTTATCAAAGATTTAATTAGATTTTTAGATAACGTATTACAGTTTTTCATAGA
TCATGCTGGTGATGAAATCTCTAAGGCTAGATATAGTGCTTCACGTGAGAGAAGTCTAGGTCTAGGAGCT
ATGGGGTTTCATTCTTATTTACAGCTTCATCGTGTACCTTTTGAGTCTCAAAGAGCAAAAGAAATTAATG
AAGAGATATTTAAGCGTATCAAAGAACAAGCGCTAGAAGAAACTCTAATACTTGGTGCTGAAAAAGGCGA
AGCGCCAGACATGGCAGGAACAGGACGTAGAAATGCTCACTTACTTGCTATAGCTCCTAATGCCAATAGT
TCGCTTATCTTGAATACTTCTCCAAGTATTGAACCTTGGAAAGCAAATGCTTTTACCTCTAGAACAAGAG
TTGGTTCACATCTTAATAAGAACAAATATCTAGAACAAGAACTAGAAAAAAATTGGTAAAAATACTGAAGA
AGTTTGGTCAGATATTATTACTAATGGTGGATCAGTTCAACAACTAGATTTTCTCAGTAAAGAATTAAAA
GAAGTATTTAAAACTGCTATTGAAATGGATCAAGATTGGTTAGTCTATCTAGGTGGTGAACGCCAAAAAT
ATCTATGCCAAGGACAATCATTAAATGTATTTTTCCCAGCGGGAGCATCGCGTGGATATCTTCACAAAGT
ACACTTTAATGCTTGGAAATATGGTTGTAAAGGTTTATATTATCTAAGAACAGAGACTTCAAATAGAGCT
GAGAATATTTCTAAAAAAGTCGAGAAGAAAAGACTTGTAGAGTTCTCAGAAATTAAGCAGTCTGAAAATG
AGTGTATCGCTTGTGAAGGATAA

>lcl|NC_006570.2_cds_YP_169564.1_465 [gene=mdh] [protein=malate dehydrogenase]
[protein_id=YP_169564.1] [location=complement(556707..557666)]
ATGGCTAGAAAAAAAATAACACTTGTTGGTGCTGGTAATATTGGTGGTACACTAGCTCACTTAGCACTTA
TAAAACAATTAGGTGATGTAGTCCTTTTTGATATAGCTCAGGGCATGCCTAATGGCAAAGCACTTGATTT
ACTACAAACTTGCCCTATCGAGGGAGTTGATTTTAAAGTCAGGGGTACAAATGATTACAAAGATTTAGAA
AATTCTGATGTTGTTATTGTAACTGCTGGTGTTCCGAGAAAGCCTGGCATGTCTCGTGATGACCTACTTG
GTATTAATATCAAAGTAATGCAAACAGTCGGAGAAGGTATCAAACATAACTGTCCTAATGCTTTCGTAAT
ATGTATCACAAATCCACTAGATATTATGGTAAATATGTTACAAAAATTCTCAGGCGTACCAGATAACAAA
ATCGTTGGTATGGCTGGTGTTTTAGATTCTGCTAGATTTAGAACATTTTTAGCAGATGAACTAAATGTAT
CAGTACAACAAGTACAAGCATATGTAATGGGCGGTCATGGCGATACTATGGTCCCTCTAACTAAAATGTC
AAATGTTGCAGGTGTGTCGTTAGAGCAGCTAGTTAAAGAAGGTAAACTTAAACAAGAGCGTTTAGATGCT
ATAGTATCGAGAACTAGAAGTGGTGGTGGCGAAATTGTTGCTTTACTTAAAACAGGTTCAGCATATTATG
CACCAGCTGCTGCAGGTATTCAAATGGCTGAAAGCTTCTTAAAAGATAAAAAGATGATTTTACCTTGTGC
TGCAAAAGTAAAAGCTGGTATGTATGGCCTTGATGAAGATCTATTTGTTGGTGTACCAACTGAGATTTCT
GCAAATGGTGTCAGACCTATTGAAGTAGAGATTTCTGATAAAGAAAGAGAACAATTACAAGTATCTATAA
ACGCTATAAAAGATCTTAATAAAGCAGCTGCTGAAATTTTAGCAAAATAA

>lcl|NC_006570.2_cds_YP_169566.1_467 [gene=FTT_0537] [protein=ubiquinone
biosynthesis protein] [protein_id=YP_169566.1] [location=558345..558980]

ATGAGAAAGTTATCATTTTTAGATAGGGTTATCGAGGAGCTTGATAGTTATGCTAGATTTACAAAGGTAC
CATTAAACCCAAGTAAAAAATCTCCTAGTTCTGATACTATAGATGGAAAGTTATCTGAAATTGAAAAGAA
ACATTCAGCTGGTTTGATGAGAGTTGATTACACTGGTGAGATTTGTGCTCAAGGCTTATATAGGGGCCAA
GCTAGTGTAGCTAAATCACCACAAACAAAAGAGCATTTATATCATGCTGCTGCTGAAGAATATGATCATT
TAGCGTGGTGTGGTGAGAGACTTCAAGAGCTAGGAGCTAGACCTAGTTTACTTAATCCATTTTGGTATTG
GACATCATTTGGTATTGGTGCAGTTGCAGGGTCTATTAGTGATAGCTTGAGTTATGGTTTTGTAGTTGAG
ACCGAAAAGCAAGTGATGAAGCATCTTGATTCACATCTAAAAAGTTTGCCTGTGAATGATAATCGCTCGC
GAGAAATTCTCAAACAGATGTATATTGATGAGTCAGAACATGCTGTCGAAGCTGAGAAAGCAGGTGGTAA
GAAGTTACCGAAAACAGTAAAAGCTATTATGAAATTACAGTCAAAAGTAATGACTACTCTTGCATATAGA
TTTTGA

>lcl|NC_006570.2_cds_YP_169575.1_475 [gene=dnaQ] [protein=DNA polymerase III subunit epsilon] [protein_id=YP_169575.1] [location=566858..567550]
ATGTCACGACAAGTTTTTATAGATACTGAGACAACTGGCTTTGATTATAAGATTGGTAATAGAATTATTG
AATTTGGTGCAGTTGAAGTTATTGATAGAAGGATAACAGGTAATAATTTACATTTTTATTGTAATCCAAA
TTATGAAGTTGAAGCTGGAGCTTTAGCTATTCATGGTCTTACAAATGAATTTCTAGCAGATAAACCTCTT
TTTGAGGATAAAGTTGATGAGATGATTGAGTTTTTAAGAGGTGCTGAAGTAATCATTCATAATGCAGCTT
TTGATGTGCCATTTATAAATTGGGAACTTAGTCTCTTAAAAAATAATAAATACGGAACATTAGAGCAAAA
TGTTGCTAAAATAGTAGATAGCCTTGATTTAGCTAGAAAAAAACATCCATTACAAAAAAATAATCTTGAT
GCTCTATGTAAAAGATATCAAATTAGGAATGATCATCGTACCTTTCATGGTGCATTATTAGATAGTGAGC
TGTTAGCGGATGTTTATCTAGCTATGACAGGTGGGCAGACTAATCTAAGTCTGCAAACAGCCAAAACTGT
GAGCAAAAATAGTATTGATATTGATGTCAATAAACTAAACTTACGTAATGCTGAAGATAGTATTAGCGAT
ATTTCTGCACACCATAGTTATCTTAGTAATTTGCTAAAACTAGAAGAAGATGCAAAATGGTAA

>lcl|NC_006570.2_cds_YP_169583.1_483 [gene=FTT_0557] [protein=AhpC/TSA family protein] [protein_id=YP_169583.1] [location=575197..575721]
ATGACTAAAAAAGTACCTAATGTAACTTTTAAAACAAGAGTAAGAGATGAGAGTATTGGCGGTTCAAATC
CATTTAGATGGCAAGATGTAACATCAGCTGATATTTTTGATAATAAAAGAGTAATTGTATTCTCATTACC
AGGAGCATTTACACCAACTTGTTCAACTTATCAATTACCAGGTTTTGAAAATAACGCAGCTAGATTCAAA
GAGCTAGGTATAGATGAGATTTATGTTTTATCTGTAAACGATAGCTTTGTTATGAACAAGTGGATTCAGG
TTCAGGGAATTCAAAATATTAAACCGATTCCAGATGGTAATGGTGAATTTACTGAAGCTTTAGATATGCT
TGTTGATAAATCAAATATTGGTTTTGGCAAAAGATCTTGGCGCTATGCAATGATTGTAAATAATGGTGAA
ATCGAGAAAATGTTTGTTGAGCCTGGTAAGCGTGATAATGCAGATAATGATCCGTATGGTGAGTCTTCTC
CAGAAAACTTATTGAAGTATTTAGAGTCTAAGTAA

>lcl|NC_006570.2_cds_YP_169585.1_485 [gene=cmk] [protein=cytidylate kinase] [protein_id=YP_169585.1] [location=complement(576352..577017)]
ATGAACAATTCTAAGATTATTACAATTGATGGACCAAGCGGCGTTGGTAAAGGTACTTTAGCGAAGGCTC
TAGCAAAATACTACGACTTTAAACTATTAGATAGCGGCGCCATTTATAGACTAGCTGCCCTACACTGTTT
TAAAAATAATGCTAGCCTTGAAAATGAAGATGATGTTTGTAAAACTTTAAGGAATTTAGACATCAGTTTT
AAAATTGAAGATGATTTAGTTAAGGCATTTTTATCTAATCAAGATGTAACTAAAGATATACGTACTGAAC
AAATAGGAATGCTTGCTTCAAAAGTCGCTGCTTACCCTACTGTTAGAGCTATACTTTTAAACAAACAAAG
AGAATTTGCTACTGAACAAGGTTTGGTTGCTGATGGTAGAGATATGGGAACGGTAGTTTTTCCACAAGCT
CAATATAAATTCTTTCTCGATGCTAGTACTGAGATTACAGCCAAACGCAGATATGACGAACTAAAAACCA
AAGGTCAAAAGCCTAACTTTGAAAAAATTTTAGCTGATATTAAACAAAGAGATTTTCAAGATCGTAATCG
TAAAGTAGCGCCACTAAGACCCGCAAATGATGCTATAATAGTAGATACTTCGCAACTTTTTATAAAAGAA
GTATTTGATAGTGTTATTAAAAAAATAACTATTTAG

>lcl|NC_006570.2_cds_YP_169594.1_494 [gene=FTT_0569c] [protein=hypothetical protein] [protein_id=YP_169594.1] [location=complement(586508..587530)]
TTGAAAATTTTAAAATACTTTTATGGATCGTTCTTCGTCACCCTAATTGGTATAGTTATTGCGGTTTTTA
TTTATCCAAATGCTCCACTAGAAACAATATACTCGGTTTTAATATTAGCAATACTTGAAATATCACTTAG
TTTTGATAATGCTGTAATCAACGCAAAAATTCTTGGGCAAATGTCTCCAAGGTGGCAAAAGATTTTTATA
TACATAGGTCTACCTATAGCGGTATTTGGAATGCGGTTACTATTCCCAATTTTATTAGTAAGTGTGACAA
GTGGTATCAATTTTATGAATGTAGTCACTCTTGCTTTAGATAACCCGCAACAATACCAAGCAATATTAGA
GCACTCTATGCCGTATATTTGTAGCTTTGGCGGTAGTTTCTTACTTATGGTATTCTTAAACTTCTTTCTA
AGTGAAAATAAAGGACATCATTGGATACCTCTTATTGAAAACAATATTATTACAAAAAAAAATTCGTAACT
ATGATGGTGGCTATATCCTTTTGGCAGTTATCATTGGAGTTATCACAATCTACTATAGTGATCCAAACTA
TCAAGGTAGTTTAGCTATTGCTTTTCTCTTGGGTATAGTAGTTCATGAGAGTATTGGACTACTTAACTCA
TTATTTGACACTGCTAAAGTTAGTACTACAGATGTTGCTCGTAATGGTTTAATTGGCTTTATTTACTTAG
AGATAATAGACGCCTCATTTAGTTTTGATGGTGTTATTGGTGCATTTGCGATCACTGCAAATATTATTAT
CATTATGATTGGTCTAGGTATTGGTGCGATGTTTGTGCGATCTTTGACTATACTATTTGTCGAAAAGAAA
ACTCTTGCAAAATATATCTATCTAGAACATGGAGCACATTATGCAATTGGCTTCCTTGCAGCAGTATTAC
TACTAAAAATATTTATGCATATCCCAGAGTGGTTTAGTGGTTCAATTGGTATACTAGTACTTACGCTAGC

ATTTATTCATTCTGTTATATCTCATAAAAAATTACATAATTAG

>lcl|NC_006570.2_cds_YP_169602.1_502 [gene=csdB] [protein=selenocysteine lyase] [protein_id=YP_169602.1] [location=596297..597520]
ATGTATGATGTAAATAAAATTAGGCAAGACTTTCCATTTTTAGCACAAAAAATAAATAATAAATCGGTAC
TTTTTTTTGACACTGGTGCCTCAGCACAGAAGCCCCAAGCTGTTATTGAATGTGTAGCTGAAGCATATGC
TTATAATTATGCAAATGTACATAGAGGTGTATATTCTCTTAGTCAAGAAGCTAGCGAGAAGTATGAAAAT
GTCCGTCAAATTGTGCAAAAGTTTTTAAACTCTAAATCAGCAGATGAAATTGTAATAACAAAAGGAACCA
CAGAGGCTATTAATTTAGTTGCTAGCTCTATCGGTAAGGGGATTATTAGGTCTGATGATGAAATAGTTGT
CACAGAAATGGAGCATCATGCTAATTTTGTACCATGGCAGATGCTTTGCGAGGACAAAAACCTAGATTTT
AAAGTGGCTGCGGTCAAGGACAATGGCGAGTTAGACGTTGATAATTTATTAGCTTTAGTTACAGCTAAGA
CTAAAATTTTAGCAATTACATTATGCTCTAATGTTTTAGGAACAATCAACCCTGTGAAAGAAATTATCAA
ACAAGTTAGAGAAATTAACCCAAATATAATTGTTTTGGTAGATGGTGCTCAAGCTGTTATTCATACCAAG
GTTGATGTTCAAGATTTAGATTGTGATTTTTTTGTCTTCTCTGGGCATAAGTTATATGGACCTACTGGGG
TCGGCATTTTATATGGTAAATATGAGTTGCTAAAACAACTTCCTCCTTATAATTATGGTGGTGATATGGT
TGACGAAGTAACAATAGCAAAGACTACATTTGCGCTACCACCATATAGGTTTGAAGCTGGAACACCAAAT
ATAGTTGAAGCTATTGGACTTGGTAGGGCTATTGAGTATGTTGATTCAATCGGCATGATTAACATTGAAA
AACATGAGCAAAAATTATTAGAATACGCGACAGCTGAGCTTAATAAAATCGATGGTCTAACTATATTTGG
TCAGGCAAAACACAAAGCTGGAGTTATAACTTTTGATATTCAAGGTTGTAATGCTGGGGATATTGGTGAG
CTATTGGCAATTAAGGGTATATGTGTGAGAACTGGTAAGCATTGTGCTCATCCTTTGATGTATCGAATGG
GAGTTACCTCAACAGTGCGTATGTCTTTTGGGATGTATAATACTTTTGAGGAAATAGATTTGTTCATAAT
AGCGTTGAAAAAAGTAATATCTCAATTAAAATAA

>lcl|NC_006570.2_cds_YP_169603.1_503 [gene=FTT_0579] [protein=HesB family protein] [protein_id=YP_169603.1] [location=597541..597897]
ATGGTAGAAGTTTTTGATCCAAATGCTAGTAGCATTTTAGAAGTTACAGATGCTGCAGCTAAACATTTCA
AGAAACATCTAGACAAGCATGATGGTTGTATAGGTATTTATGTCGGAACTAAGGTTATGGGTTGTTCTGG
CTTAGCTTATGATGTTGATTTTGTTAAACAGCAACCACAAGATACACAGAAAGTACAGCAGCATGGCATT
AATTTCTTTGTATCTAATAAGTCTATGGATTTTCTTAATGGGCTAAAAATTGATTATGTTAAGCATGATT
TTGGATTATATAAGTTAGAATATACAAATCCAAACGAATCAGCACGTTGTGGTTGTGGTGAAAGTTTTAC
AGTTTAG

>lcl|NC_006570.2_cds_YP_169605.1_505 [gene=coaD] [protein=phosphopantetheine adenylyltransferase] [protein_id=YP_169605.1] [location=598474..598962]
ATGAATAAGATAGCAATTTATCCAGGTACTTTTGACCCAATTACCAATGGTCATGTTGATCTAGTAGAGC
GTGCATTAAATATCTTTGATGAAATAGTTGTTGCAGTATCTACAGCTTATGGTAAAAATACTCTTTTTGA
TATTCGTATCAGAGAGCAAATGATAAAAGAAGTATTCAAAGATAATCAAAGGGTCAAAGTTGTAAGCTTT
CAGGGTCTACTTGTTGATACAGCAGTCAAACATAATGCTTGTGCTATAGTTAGAGGTCTCAGAGCAGTAT
CTGATTTTGATTATGAATTTCAGATGTCAAGTATGAATAATAAATTAAACAGTGATATTCAAACTATATT
CTTAACTCCGAGTGAGAAGTTTTCATGTATTTCCTCGACATTAGTTAGGGCTGTAGCGATACATAATTAT
AAGCGTGTTGATGAGTTTGTACCTGAATGTGTCTTTCGTGAGATAAAGCTTAAATATTCTAAGGAATAA

>lcl|NC_006570.2_cds_YP_169611.1_511 [gene=aroA] [protein=3-phosphoshikimate 1-carboxyvinyltransferase] [protein_id=YP_169611.1] [location=607810..609087]
ATGAAAGATTTTATCCTTCAAATAAAATCACAGATAAAACAGCAAGTGTATTTAGATGGCTCAAAGAGTA
TCTCAAATAGATCACTGATAATCGCAGCGATGGCTCAAGGTCAAACAAAATTTGAGAATTTGCCTAATAG
TGCTGATGTTTTAGCATGTATTGCAGCTCTAAAGGAGTTAGGTTGTCAGCTAGAACATAGCTGGGATTCA
AAAACTTTAGTTATTCAAGGTTGTAGTGGAGTATTTGCGAACTTAGATGTCAAGATATTTTGTAATGAGT
CTGGTACTTTAACAAGATTTATAATACCAATGTTAGCAGTGCAGTCTACAGGTAAATATTACGTATATGC
CAAGCAAAGAATGATGGATAGACCCTTAGCAGATCAACTAAAACCGCTTGAAAAGTTAGGTATGCTAGCA
AATTACCATCAAAAGGCTTATGCTATGCCTTTGACAATAATAGCTAAATCTTTGGATGGTGGTTATATAG
AAGTTGATGGCGAGAAGAGTTCGCAATTTGCCTCAGGTTTGCTGATGGCAGCACCATTTATGCACCGTGG
CTTAAGATTAAACTCTATCACAGACCACAAGCAACCGTACTTAGATATGACAACTAAAGTTATGGCAGAG
TTTGGTGTGACAGTAGATATTGATGAAAATATCTATACTGCCAACAAATCTCAATATATCTCTCCTAGTA
ATTATGTAGTTGAACCAGATGTTTCGACAGCTTCATATTTTTGGGCATTTGCAGCTATCACCGGTTCAAC
TATCAAGGTTATGCATGTTACTAAAAACTCTAAGCAAGGTGATATTAAATTTTTAGAAGTACTTGAGAAA
ATAGGCTGTCAAGTTAATTACTACAATGATGGTATAGAGGTTACAGGTAATAATCAGCTTCGTGGTATAC
AAGTTAATATGCGTAATTTCTCTGATACTTTCATGACGCTTGCAGCTATTGCTTGCTTTGCTAAAGGCGA
TACGCATATTTCAGGTCTTAGTCATACACGTGGTCAAGAATCAGATCGCATTGCAGCTATGGCAGAGGGA
CTTAGCAAGCTTGGTATCTATGTGGAGACTACTCAAGATAGTATTTTAATCTCGCCAGCTAGAAGTAAAT
TTAAGCCAGCTGAAGTTGATAGTCATAATGATCACCGTATCGCTATGTCTTTAGCACTTTTAGGCTTAAA
ATATCAAGGTGTAATAGTAAATAATGCAGCGGCTGTAAGCAAAACTTGCCCAGATTATTTTGATAGAATG
AGAGCATTGGTAGGTTAG

>lcl|NC_006570.2_cds_YP_169615.1_515 [gene=cynT] [protein=carbonic anhydrase]
[protein_id=YP_169615.1] [location=611223..611909]
ATGAGTGATATTTCTGAGTTGATAAAAGGCAATAGAGCATGGGCTGAAGAGATCAAAAAAACAAATCCAG
GTTTTTTTGAAACTTTATCTAAAGGTCAGTCTCCAGAATACCTTTGGATAGGTTGTTCAGATAGTCGAGT
ACCTGCAAACCAAGTTTGTGGTTTGATTCCAGGTGAGGTTTTTGTCCATCGTAATGTTGCAAACGTTGTT
TCACTTACAGATTTAAACTGTTTGTCAGTGTTACAGTTTGCTATTGAAGTCTTAAAGATTAAGAAAATAA
TAGTATGTGGTCACTATGCTTGTGGTGGTGTTGAGACTGTTGTCAAAGATAAGAGCTATGGTCTAATTGA
TAACTGGCTAACGTCTATCCATGAAGTTAAAGAGCAAAACAAACAGTTTATTGAAGAGTCATTATCTTGT
TACAAAGATAATCAAGAAGAATATATGAAGAAAAAAGTAGATATGATGTGTGAGCTAAATGCACTTCATC
AAGCGCTAAATCTTTGTAAAACTACAGTTGTTAAAAATGCGTGGGCAAAAGGGCTTACCTTTACTATACA
TGCAGCTATATACGGTATTGGCGATGGTAAGCTTTATGAAATTGGTGGTGGTGTAGGCTCAAGAGCAGAG
ATGGATACTACTTATGCTCAAGCTATTAAAGACATCAAATCTAGATATTGTAGATAA

>lcl|NC_006570.2_cds_YP_169618.1_518 [gene=rubA] [protein=rubredoxin]
[protein_id=YP_169618.1] [location=complement(613746..613916)]
ATGGAATATAGAAAATACATTTGTATCGTTTGTGGCTTGATTTATGATGAAGCTGAAGGATGGCCAGAAG
ATGGTATCGAACCAGGTACCAGATGGGAAGATGTCCCGGAAGATTGGGAATGTCCTGACTGTGGTGTTAG
TAAAGATGAATTTGAGCTTCTAGAAGAATAA

>lcl|NC_006570.2_cds_YP_169621.1_521 [gene=FTT_0598c] [protein=sodium-dicarboxylate
symporter family protein] [protein_id=YP_169621.1]
[location=complement(616158..617438)]
TTGCGTATGGCAATTTTACTTATAATTTCTTTACTAATTTTAGTATATCTTCACCTAAAAAAAATTAGCT
TTAATTTCCGGACTATACTCGCATTAATAATCGGTGTTGTTATTGGGATTATCTACAATTCTACTGATTA
TTATAGCAATAGCTTCATACAAATTAGCAATATATTGGGTGATGGCTATATTTCTTTACTTAAGATGTTG
ATTATTCCAATTGTATTAACATCAATAATTCACTCAATAATTAACCTAAAAAACTATGAGGGCTCTTATG
TTATAAGATTTGCCTATAAAACTATAGCAATACTTTTAATCTTAACTGGTATAAGTGCTGCAATAGGCGC
TAGTGTTGCTATTATTATGCATTTAGGACAGGGCATAGATATCGCATCTATCACAGGTAATGTTGCCAAA
AACATCAAGACCTCGAGTATATCCGAGACTATTCTAGGCTTCTTACCTGATAATATTTTCCATCAAATGG
ATAACAATAATGTCATGGCTGTAGTAATATTTGCGATACTTCTTGGTTTTGCAATGCTAATTGCACATCG
TGAAGATAGTAAGTTAGCTGCTCCATTTATCAGTTTTATTGATTCAGCATTTTTTGTGATAAAGAAATTA
GCTAGAATGATTATTGCTATAACACCTTATGGTGTACTTGGTCTGATGGTACAAATGAGTATTGAGCTTG
ATAAGAATAGTATCTCAACGGTCTTATACTTCATTTTAACTTGTTATATCGCATTAATAATTGTTCTAAT
AATGCATATTATCCTTCTAGTTTTATTTAGGACCAATATTGTTAGATTCTACAGAAGTATTTGGAAAGCC
ATGCTCGTAGCTGCTACTTCTAGATCAAGTATGGGCACACTACCATTATCAATTGATGGTCTAAATAAAT
ATGGTACTACTAGTAGTATTGCTACATTTGCACCAACTATGGGTACAACTCTAGGAATGAATGGCTGTGC
TGGTGTATTCCCCGCAGTATTAGCAATTATGGCTATGAATGCTACTGGAGTGGATATTACTTTCTCAACT
GTAGTATTAATTTCTCTAATTTGTATGCTAGCTTCACTTGGTGTCTCTGGGATACCTGGTACAGCTTTTG
TTGCAGCTGGTGTGGTTTTCTCTTACTTTGGCTTGCCATGGCAACTGATAGCTCTAATTATAGGAGTTGA
TGCTATTATAGATAGCTTTAGAACGCCTCTTAACATTCATGGTACTATGACTACAGCTATTATTGTTGAT
AAAACTACTAAAGCTTCTTAG

>lcl|NC_006570.2_cds_YP_169628.1_528 [gene=ispG] [protein=4-hydroxy-3-methylbut-2-
en-1-yl diphosphate synthase (flavodoxin)] [protein_id=YP_169628.1]
[location=625340..626557]
TTGGGTTCTTATATGAATAAAACAAGTGTTGTAAAAGTTGGAAATGTTTTGATAGGTGGTGATAATCCAG
TTGTTGTTCAGTCAATGACAGATACATATACTGCAGATGTTGAAAAGACAGTTAAGCAAATCTTAGCACT
TCATAAAGCAGGTAGTGAAATTGTTAGAATTACAGTTAATGATGAGTCAGCTGCTGCTGCTGTACCTGAG
ATTGTTAAAGAGCTAGCAAAGCATGATTGTCATGTGCCTTTGGTTGGTGATTTTCATTATAATGGTCATA
CATTACTTAGTAAATATCCAGAATGTGCGAAGGCTTTGGCAAAATATCGTATAAATCCTGGTAATGTTGG
TTTTGGTAAGAAAAAAGATACTCAGTTTGCAGAGATTATAAAAATTGCAATAGCAAATGATAAGCCTGTG
CGTATAGGCGTAAACTGGGGAAGCTTAGATCAAGCTTTATTAGCAAGGCTAATTGATGAAAACAATGCTC
AAGAGAATCCACTAAGCTTACAACAAATAATGCATAAGGCACTTATAACTTCAGCTTTAGAAAGTGCTAA
ATATGCAGAAGAGCTGGGATTAGCAAAAGATAAAATTATAATCTCATGTAAAGTAAGTGAAGTACAAGAT
TTAATTGCAGTATATCAAAAACTAGCTAAAGAGTGTGGTTATGCTTTACACCTAGGATTAACAGAAGCTG
GTATGGGTACTAAAGGTATTGTTGCTAGTGCTGTGAGTTTAGGAATTTTATTACAGCAAGGTATAGGTAA
CACTATCAGAGTTTCATTAACTCCAGCGCCAAATGCTCCACGTACAGAAGAGGTGCGTGTATGTCGTGAA
ATCCTTCAAAATCTAGGCATGAGAACATTTACTCCAAGTGTAACATCTTGTCCAGGTTGTGGTAGGACTA
CTAGCTCGTTTTTTAGAGAACTAACAAGCAAAGTTAAAGATCATTTGGATGAAAAAATGCATACTTGGAA
AGAGCAATATCACGGCGTAGAAGCAATGAAAGTGGCTGTAATGGGCTGTGTTGTTAATGGTCCAGGCGAA
TCAAAAAATGCTGATATTGGTATAAGCCTACCAGGTAGTGGCGAGTCGCCTGTTGCACCAGTTTTCATCG
ATGGTAAAAAAGCACATACTCTAAGAGGGGATAACATTTCTGAAGAATTTATAGAGATTGTAGAAAATTA
TGTTAAAAATCGTTATGGTAAAAAATAG

>lcl|NC_006570.2_cds_YP_169637.1_537 [gene=FTT_0616c] [protein=hypothetical protein] [protein_id=YP_169637.1] [location=complement(635666..636172)]
ATGAAGAGAAAACTAAAAATGGATAATCTAAATATAAATTTCATCAATGATGACGAACACCCCATACCTA
GTCAAGATTTACTGCTAAAATGCCTACAACTTGTAGCTAACAAACATCATATCAGTCATGCTGAAGTTAA
TCTAAATATTGTCTCGAATGATGAAATTCAACAAATAAATAAACAGTTCCGCAATAAAGATAAACCAACA
AATATCATTTCCTTTGAATTTGAAAAGCCTCAAGGCTTACCTGATGATATTGCTAATGATTTCTTAGGTG
ATATTGTAATAGCGCCAGCGGTATTAGAGAATGAAGCCAAAGAGCAGAACAAAGAGCTCAATGACCACTG
GCAACATATTTTTATCCACGGTTTGTTACACTTGCTTGGTTATGATCACCAAGATGATCAAGAAGCCGAA
GTGATGGAGAATTTAGAAATACAGCTACTAGCACAGCTAGGAATAGCTAATCCATATATTGAACAAGAGA
ATCAAAATGGCAGATAA

>lcl|NC_006570.2_cds_YP_169648.1_548 [gene=hupB] [protein=histone-like protein HU form B] [protein_id=YP_169648.1] [location=647057..647329]
ATGAACAAGAGTGAATTAGTAAGTGCTATAGCTAAAGAAGCAGATGTTACTAAAGAAGTTGCTAGCAACA
CTCTAGATGCTACTATTGCGGCAGTAACTAAAGCTTTAAAAAATGGTGATAGCGTAACTCTAGTAGGTTT
TGGTACTTTTCAGGTAAAAGAAAGAAGTCCTAGAGAGGGTAGAAACCCAAAGACTGGTGAAACTATTAAG
ATCCCTGCTTCTAAAGTTCCTAGCTTTAAAGCTAGTAAGGGTCTTAAAGACGCTGTAAAATAA

>lcl|NC_006570.2_cds_YP_169650.1_550 [gene=miaA] [protein=tRNA delta(2)-isopentenylpyrophosphate transferase] [protein_id=YP_169650.1] [location=648872..649798]
ATGAGCAAATTAATCTATGGTTTGGCTGGACCAACAGCTTCTGGTAAAACTTCATTATCAATATTGTTAG
CTAAAAAAATTAATGCAGAAATTATTAGTGTTGACTCATCTCTTGTTTATAAAGGTATGGATATTGGTAC
AGCCAAGCCAACTTTACAAGAGCAAGATGGTATTAAGCATCATCTTATAGATATTATTGAGCCAACAGGG
AATTTTTCGGTTGCTGACTTTATATCCAGTGTTAATAAACTCAAAAAAGAAATTTGGGCTAGAGGAAGAG
AAGTCTTACTTGTTGGTGGGACAATGCTCTATTTTAAAGGTTTGATAGAGGGTTTATCTGCGTTACCAGA
ATCTCAAGCCGAGATTAGAGAAGCACTAGAATACCAGAAAAAAGCTAAAGGATTACAATATCTTCATCAG
CAACTAAATGAAATTGATCCACAATCTGCTCAAAAAATTAACCCTAATGACCAGCAAAGAATCTTTCGAG
CTTTAGAGGTGATTATGATAAGTGGAAAAAAATACTCTGAGCTTGTTAAAACATCTAAAGTTGGTGGTTT
GGATGAAGATCTAAAATTATGTGCTTTAGTGCCAAATGATAGATCAATATTGCATAAAAATATTGAGTCT
AGATTTAGACAAATGCTTGATCAAGGATTTTTGGATGAAGTCCAAAATTTACACAAAAATCCTATGCTTA
CTAAAGAAACCACAGCTATTAGAAGTGTTGGATATCGTCAAGCATGGGAATATCTTGATGGTGATATTAG
TTATGATGAGTTTGTTAAAAAAGGAATTGTAGCCACTCGCCAACTTGCTAAGCGTCAACTAACATGGATT
CGTAATTGGCAGAGTTCTATAAATATAGTTGCTATGGAGAACGAAACTAAAGAGTTAGATATTTTAAAAT
ATTTTGGTTATAAATAA

>lcl|NC_006570.2_cds_YP_169657.1_557 [gene=engB] [protein=ribosome biogenesis GTP-binding protein YsxC] [protein_id=YP_169657.1] [location=655448..656041]
ATGAATTATAGTAAAGCAAAATATATAATGGGTGCGGCAAAGGTTTCACAGCTTCCAGAAGATACTGGTG
TGGAAGTTGCTTTTGCAGGACGTTCAAATGCTGGTAAATCAAGCGCATTAAATACTCTTACAGATCAAAA
GGGTCTTGCTAGGGTAAGTAAAACACCTGGAAGAACACAACTTATTAACTTATTTGATTTAGGTAATAAT
AATAGATTGGTAGATTTGCCTGGCTATGGTTATGCAAAAGTTTCAGAAAGTATTAAGCGTCAATGGCAGA
GTGAGATGGAGAACTATTTGACATCACGTAAATGCTTAAATGGTATAGTACTTTTAGTAGATTTACGTCA
TGAGTTAAAAGAATTTGATTCTCTAATGATAGAGATGGCGATATCTTTTGACTTAAACTTACATATATTA
CTGACAAAGGCTGATAAGTTGAATAATAAAGAACGAGCTCAGGCAAATAGAATGATAGAAAGTTTTCTAA
AAACCTTTGTTTCTACAGATAAAATATCATATCAATTATTTTCATCATTAACTAAGATGGGACTAGATAA
GTTTAAAGAAAAGCTTGATACTTGGTATCAATAA

>lcl|NC_006570.2_cds_YP_169664.1_564 [gene=FTT_0646c] [protein=hypothetical protein] [protein_id=YP_169664.1] [location=complement(667306..668565)]
ATGAGAAATTCTATAAAAATTTCAAAAGTAAATATTAATTTTAATCAGCCTAAAGGTTTACATATCATTG
CTTTTATGATTTTTTGCCTATGTTTATGTATTGCTCCTTTTATGACTATATTAAATGGCTATGGTAATAT
CAACTTCTTCTATAATCGTAATGATCATCTTTTTGTGATATTTTTTGCTGGAATTTTTGGCTGTATTAGT
AACTATATTTTTGGTTCAACTAAATCAATTATTCATGGCTTACAATTCATAATAGTAGCAATCGTTTTAT
CTTTTATTCACAATATGGTTTTATTTAGTTGTGCTACATTATGGATTGGTCTTGCAATAGTTATCGTTAA
TCTACTTTTTAACTTAAGTGCTTTTTATCTAAAGACAGATATTCGCAGAATATATGGTTTTATTGGTGTA
TACTCAAGTGCTTTACTTGGAATTGCTACGGGAATTATTATTTACTTCTTAGTTCTTAAAAATATGTACC
ATTTTAAAATTCTTTACCTTTTAATAATAATATTTTTATTATTATTTTTCCTTAAAAACAGTTATAAGCT
TAATACCTCATTAACAAGCCAAACAGAAAGGATCAACATAAGCTTTTATGGCGTACTGTTGATTTTCTTT
ATATTTGCATTTATACTTTTTTATCTGCTTGTTAATCTAAATGTTTTTAGCAGTCTAAACTTAGTTATAT
TACCCTTATCACTAATATACTTACATGTTATTACGGTTACTAATAACAAAGAGAACGGTAAAAATCTCTT
AAAATACATTTACTTTAGCCTAACACTTATAGTCATTAACAAAATCTTTTATCTTAGTTTTCTCCGATAC
GAAGATGTAGTAGATCCAATATATCTAAATTCTGCTATTAGTACTTTTTTATTTTTACTAGCTGAATATT
TGTTATGCATAATAATATATTTTGGCTGGAGGTTTAAATTTATAACTCTGCGCATTGAGTCAATCACTAA

CTCACATATCATCAAAACGATGTTGTATATCGAAGCTATCCGAGTGTTTATCTTGATAACAGCTATTTTT
ACAAATAATATTCTTATCAGTAATATAATCCTTATATTTGCTACAATTTTATCTTTGGTTTTAAATATTT
TTATCGTGCCAATATATTTTTCTCTTGGTAAAATACTCGCCGGTGGCAAAAATGAAATTATTACTACAAC
TCTACTATATCTAATATTCTCGTCACTAATACTTGTATCTTTCTTGTATGATATTTCTATTAGTTTATAA

>lcl|NC_006570.2_cds_YP_169671.1_571 [gene=lipA] [protein=lipoyl synthase] [protein_id=YP_169671.1] [location=673236..674219]
ATGAAAGAAATATCTGGTATAAAAGTTAAAGTTGAAAGTGGCTCTAAGTACACTACCGATCATGGTTTTC
ATGCTGTCAAAGATGGTATTAGAAATAAGAAAGAAAATGCTGTGCATGTACGTAAACCAGATTGGTTGAA
AGTGCAAAAACAAGATTCTAAAGAATACCTAAAAGTTAAATCCATTACAAAAAAACATAAACTTTCAACA
GTTTGTGAAGAAGCAAGGTGCCCTAATATTAATGAGTGCTGGTCTCATGGTACAGCAACAATTATGTTAA
TGGGGAGTGTATGTACTAGAGCTTGTAAATTTTGTTCTGTAGATACAGGTAATCCAAAAGGTTGGTTAGA
TAAAGATGAACCTATGAATGCTGCTGAAAGTGTAAAGCTCATGGGACTTGAATATGTTGTATTGACATCT
GTAGATCGTGATGATTTAGAAGATGGTGGTGCTGGACACTATGCAGCGACTATTACAGCTATCAAAAATC
TTGATGAGAATATTAAAGTAGAAGCATTGACTCCGGATTTTGCTGGTATTAACGAAAATATTGATAAAAT
CATAAATACAAAAGTAGATGTAATTGCACAAAATATCGAGACTGTAGAGCGCTTGACTCATCCAGTACGT
GATCCACGTGCAGGGTACTGGCAAACATTAAATTTCTTAAAATATGTTAAACAAAAATCACCAAATGTGC
TTACTAAAACTAGTATTATGGTAGGTTTAGGTGAGACTGATGAAGAAATTTACAAAACTATGGATGATGC
GCGAAGTGTTGGAGTTGATATAATAACGCTTGGGCAATATATGCAACCAACAAAGCATCATTTGAGTGTT
GAGAGGTTTGTGACACCACAACAATTCGAAGAGTATCGCAAAGTGGGTCTCGAAAAAGGCTTCTTAGAAG
TGGCATCAGGGCCTATGGTAAGATCAAGCTATAGAGCTGATAGAGTTTTCAAAAGGAATAATTTAGATTT
GTAA

>lcl|NC_006570.2_cds_YP_169675.1_575 [gene=ruvA] [protein=Holliday junction DNA helicase RuvA] [protein_id=YP_169675.1] [location=677567..678220]
TTGATGATAAGTTTTATAAAAGGTGTATTGATAGAGAAAGATCCAACAGCTTTGCTTATTGATGTAAATG
GTATTGGTTATGAAGTTTTTGTACCGATGACAACATTTTATACATTAGGTGATATTGATAGTCAGGTTAG
CCTTTATACACATTTTGTAGTTCGTGAAGATGCTCAGCAACTCTATGGGTTTAAATCAAAAGTTGATAAG
AAAGTTTTCCAAGAGTTAATTAAAGTCAATGGTATAGGAGCTAGAACAGCTATTGCTATTTTATCTGGTA
TGGATTCAAAAACTCTATTACATTGTATTGAAAATAAAGATTATGCTTTATTAGCTACAGTGCCAGGAAT
TGGTAAAAAAACTGCTGAGCGTTTAGTTGTAGAAATTTATGATAAGTTATTAAAAATGGCTAATGAGATT
TATGCTCAGACTTCAGGTACAACTACAACTAGTCAAGATTCACAAGCACAACAGGCACCAACATCTGTAG
TATTAGCAAACTCAATATTTAACGAATCCGTTGATGCATTATTGGCATTAGGCTATAAGCAAAAAGATGC
TGAAAAAATGGCTCGCTCTGCTATGGGTGATGCAACTACAGTAGCAGAGGTAATTCGTAAAGCTCTGCAA
GGATCAATAAAATCAAAAAGATAA

>lcl|NC_006570.2_cds_YP_169690.1_589 [gene=prsA] [protein=ribose-phosphate pyrophosphokinase] [protein_id=YP_169690.1] [location=692007..692975]
ATGTCAGAAGATTTGATGATTTTTAGCGGTAATGCTTCTAAAAAGCTTGCTAGTGAAGTAGCTAAAGAGC
TAGGTGCAACTCTTGGTAATGCAACGGTTGATAGGTTTAAAGATGGCGAAATACATGTTGTCCTAAACGA
GAATGTGCGTGGTAAGGATGTATTTGTAATCCAATCAACTTGTCCACCATCTGATAATTTGATGGAACTT
ATTCTATTAATAGATGCGCTTAAAAGATCATCAGCAGAGAGGGTAACAGCAGTATTACCATATTTTGGCT
ATGCTAGGCAAGATAGAAGATCAAAATCAGCGAGAGTGCCTATATCTGCTAAAGTTGTCGCAAATCTTCT
TCAAGCTGTTGGCTTAGATAGGATATTATCAGTAGATATTCATGCTGAGCAAATCCAAGGATTCTTTGAC
ATACCATTTGATAATGCTTTTGCAACTAAAATATTCCTAGAATATGTGCGTAAAAATCCAGAGAAATATC
AAAATATCAAAATAGTATCACCTGACATGGGTGGTGTGGTTAGAGCTAGATCTGTAGCTAAAAACTTAGG
TGTTGAGATTGCTGTAGTTGATAAAAGAAGACCTAAACCAAATGTTGCAGAGGTTATGAACATAATTGGC
GAAGTTGATGGCAAACATTGTATACTTGTTGATGACATTATGGATACTGGTGGCACAATGTGTCAAGCAG
CAAAAGCATTGATAGAGAAGGGTGGAGCTGCTAAAGTATCAGCATTTTGTATACATCCATTACTTTCTGG
CGATGCGATTAAGAATATCGAGGATTCAGCAATTGATGAGCTCATAGTTACTGATTCTATACCTCTTAAA
CCTCATGCTGAAGCATGTAGCAAAATCAAAGTCATAACATTAGCACCATTACTTGCTCAAATTGTTGAAA
AAACTAATGGAGAGGAATCAGTTAGTGATATTTTCCGTATTGATGGCTTAGTTGATTAA

>lcl|NC_006570.2_cds_YP_169691.1_590 [gene=rplY] [protein=50S ribosomal protein L25] [protein_id=YP_169691.1] [location=693075..693365]
ATGGCAAATTTTGTTCTTAAAGCTGAAAAGAGAGAAGACTTAGGTACTGGTGCGAGCCGCCGTCTAAGAA
GAGCTGGTAAAATCCCAGCTGTTATATATGGTGGTGAAAAAGAAGCGGTATCTGTATTACTTGATCATGA
TAAAGTACTACACTCAACAGAAGACAAAGCGTTTTTCTCAAGTGAGATAACTTTGGATATCGATGGTAAA
CAAGAAAAAGTAATTATCAAAGCATTACAAAGACATCCATATAAAGTTAAGCTTATCCACGCTGACTTTA
TGAGAGTATAA

>lcl|NC_006570.2_cds_YP_169696.1_595 [gene=pth] [protein=peptidyl-tRNA hydrolase] [protein_id=YP_169696.1] [location=complement(697710..698285)]
ATGCCTAAAATAAAAATGATTATTGGCCTAGGTAATATAGGCAAAGAATATCAAGATACGCGCCATAATG

TTGGTGAATGGTTTATTGCTAAAATAGCTCAAGATAACAATCAAAGCTTTAGTTCTAATCCTAAGCTTAA
TTGTAATCTAGCTAAAGTTAGCATTGATTATAATAATGTAGTACTAGTATTTCCTACAACTTATATGAAT
AACAGCGGTTTAGCTGTTAGCAAAGTCGCTAATTTTTATAAAATCGCGCCAGCAGAGATACTTGTAGTAC
ATGATGAGCTAGATATAGATTCTGGAGAAATACGTCTAAAAAAAGGCGGAGGTCATGGTGGTCATAACGG
TCTAAGAAGTATTAACCAACACTTAGGCACTAATGACTACCTACGCCTTAGAATTGGTATTGGTCATCCT
GGTCATAAATCAAAAGTAGCTAACTATGTATTATCAAATCCATCTATAGCTCAGAAAAAAGATATAGATA
GTGCTATTGATAATGGTATCTGTTTTTTAGATGATATAATAAATTACAAATTAGAACCTGTAATGCAGAA
GCTACATACAAAATAA

>lcl|NC_006570.2_cds_YP_169700.1_599 [gene=sthA] [protein=soluble pyridine nucleotide transhydrogenase] [protein_id=YP_169700.1] [location=complement(700878..702293)]
GTGTGGAGTAAAACCATGGAATATAATTACGATATTATTATTATAGGCAGTGGTCCTGGTGGTGAAGGGG
CTGCGATGAAAGCAACTAGGAACGGACAAAAAGTAGCTATCATCGAAGATGACGCTATTGGTGGTGGTTG
TAATAACTGGGGAACAATTCCAAGTAAAGCTCTAAGACAGCTATCACGTGAAGTTTGGCACAACAAGAAG
AATTTCGATTTCCCAGAGATGCTTGATACTGCTTATGAAATAGTTATCAAACAAAGAGAGATTAAGAGAA
ATCGTTTCGCTAATAATGAAATTGATGTTTTCTATGGTTTTGCTAGTTTTATCGACAAGCATAAAATAAA
AATTTCACGTAAAAATGGTTCAACTGAAATCATTACGGCAAAAAAATTCATTCTCTCTACAGGATCTCGT
CCATATCATCCTGATGATATTGACTTTACTCATCCTAGAATTTTGGATAGTGATAAGCTTCTAGAGTTGA
AAGACAAAAATATCAAATCAATTACTATTTATGGTGCTGGTGTAATTGGTTGTGAATATGCATCCATACT
TGGAACACTAGATATTCAGGTAAATCTTATCAACACTAGAAATAAACTAATGTCGTTCCTTGATGATGAA
ATTATCGAAACGCTAACAAATCACTTTACAGTTAATCAAAGAATTAATCTAATACATAATGAAACTTATA
AAAGCATCAAAGCAAGAGGAGATAAAGTAGTTACTACTCTTAACTCAGGTAGAATTATCGAATCTGATTA
TGTCTTGTTTGCGCTTGGCCGATCAGGTAATACAAATGGCCTTAATTTAGATAAAATTGGAGTTGAGTAT
GACCCACAAAGAGGTCTTGTTAAGGTTAATGACAATTATCAAACTACTCAAGAGAATATATATGCTGTTG
GTGATGTTATAGGTTTTCCTTCACTTGCATCATCTGCTTTTAACCAAGGTAGATTTGCAGCTACGCATAT
TATAGATGGCTCTTGTAATGACAAATTAGTCGAGGATATCCCAACAGGGATCTATACCCGCCCTGAGATT
AGTTGTATTGGAAAAACAGAAGAGCAATTAACTGCTGAGAATATTCCATATGAAGTTGGTAGAGCATATT
TTAAAGATTTAGCTCGTGCACAAATTTCAGGAAGTGAAACAGGGATGTTAAAGATACTTTTCCATAAAGA
AACTCTAGAGATCCTTGGCATTCACTGTTTTGGTCATAGGGTATCTGAGATTATCCATATTGGTCAAGCT
ATCAAATCAATGCCAGGCAAACACAATACTATCAGATACTTTTTGAACACTACATTTAACTACCCTACTA
TGGCTGAGGCTTATCGTATTGCTGGTATTGACGGTCTTAATAAGCTTAAACCTAAAAATAAACAATTTGT
CCCAGAACACCAATAA

>lcl|NC_006570.2_cds_YP_169706.1_605 [gene=lplA] [protein=lipoate-protein ligase A] [protein_id=YP_169706.1] [location=complement(708031..708933)]
ATGCATATATACATATCTCAAAGTAACGATATCTATTTTAATTTAGCTTTTGAAAATTGGTTATTCTTGG
AGAAGCTGCATCAACAAAAAATTTTATTCTTGTGGCAGAATTCTCCTTGCGTTGTTATTGGTAGAGCCCA
AAATCCTTGGCTTGAGTGCAATCTTGAAGCTATGGATAATGATAAAATACCAATGATTCGTCGTCAAAGT
GGTGGCGGTACGGTTTATCATGATTATGGTAATCTTAATTACACTATTATCAGCACTAAAAAAGATCATG
ATATCAAAGCAAATTTAGAACTAGTTTGTAATGCTATTAAAAAACTAGGCATTGATGTATATCCCAACCG
AAGAAATGATATTGTTCTTGATCATCACAACTATACATATAAAATATCAGGTAGTGCCTTTAGAGAAAAA
AAAGATCGAGCTTTTCATCATGGTACTCTATTGATTAATGCAAATACAAAAAAGCTTTATGATTATCTTC
ATCAGCCAATAGATAAATCTCTCGATACTAAAGGAGTTAAATCTCACCGTTCAAAAGTAATCAATCTATC
TGAAATAAAGCATGATATACAAACTCAGGATATAACTAGATCTTTCATTAAAAGCTTTAGAAGTATTGAC
CTAAGTTTAATCAATGAAGAAACTCCATTAGAAAATAAAGAGCTTATAGAAAAAGAAATTGAAATCTTAA
AGGATTGGCAATGGCGTTTTGGTAAAACTTTGCCTTTTACAAAAACTTATACAAAAGCAAGTGAACAAAT
TAAAATCAAAATTGAATCTGGAATTGTTACAGAAGTTAGGAATGTTTATAAAAATACAAATCTTGCTGAT
AAGAATATTCGCTTTGAAAATAGATATGATTTTGATTTCTTTAAGAAAATTCTTACAGAATAA

>lcl|NC_006570.2_cds_YP_169707.1_606 [gene=tyrS] [protein=tyrosyl-tRNA synthetase] [protein_id=YP_169707.1] [location=709046..710236]
ATGTCGAGCATAAAAGAGACTTTAGAAATTATCAAAAGAGGGGCGGATGAGGTCCTAATCGAAGAAGAAT
TAATCAAAAAATTACAAAAACATAAGCCATTAATAATTAAATTTGGTTGCGACCCAACTGCTCCAGATAT
TCATTTGGGACATACTGTAGTTATCAATAAGCTAAAGCAATTACAAGATTTGGGACATAAAATTCACTTT
TTGATCGGGGATTTTACAGCTCAGATAGGTGATCCTACAGGTAAAAATGCAACAAGACCGCCATTAACTG
CCGAAGAAGTTGCGGCAAATGCTGAAACTTATACTAAGCAAGTATTTAAGATTCTTGATAAAGACAAAAC
TATTATTCGTCGTAATGGTGATTGGTTTAACAAAATGTCTGCTAGTGAGATGATCAAACTAGCTTCTAAA
TCAACGGTTGCAAGAATGCTTGAAAGAGATGATTTCTCAAAAAGATATAAAGGCGGACAGTCGATCTCAA
TACATGAGTTTTTATATCCATTAGTACAAGGATATGATTCTGTAGCTATGAATGCTGATATTGAGCTTGG
AGGTACAGATCAAAAATTTAACCTACTAATGGGTAGAGAGCTGCAAAAGCAGCAGGGTCAAGAACCACAA
GTTATTATCACAATGCCACTTTTAGAAGGTTTAGATGGCGTCAAAAAAAATGTCTAAATCAAGCCAAAACT
ATATCGGTATCGAAGAACCAGCAAATGAAATCTTTGGTAAGATAATGTCAATTTCAGATGAATTTATGTG
GCGCTATTATGAATTACTAAGTTTCAAATCTTTAGAAAATATCGCTAAATTAAAGCAAGATGTTGCAGCT

GGAGCTAATCCGCGCGATATTAAGATAGAGCTGGCAAAAGAGTTAATTGAAAGGTTTCACTCTAAAGAAG
ATGCTGAGAGTGCGCACCAGGATTTTATTCAAAGATTCCAAAAAAATCAGATACCTGATGATATAAATGT
TGTAGAATTAAACCAAGAGCTACCGATAGCAAACTTATTAAAAGAAGCAGGCTTAGTTGCAAGTACTTCT
GAAGCTAATCGTATGATTCAGCAAGGTGCTGTTAAAATCGATGGTGAAAAGCTTAGTGATGCTAAGATTA
TTTTTGCTAAAGGAACAAATAATGTTTTCCAAGTAGGAAAACGTAAATTTGCAAAAATTATTATAAAATG
A

>lcl|NC_006570.2_cds_YP_169712.1_611 [gene=FTT_0696] [protein=hypothetical protein] [protein_id=YP_169712.1] [location=715529..715879]
ATGATGTTAACAAATCGTCAAATAAGGGTAAGATTATTTGAATCACTTAAAAATAGTTTCTTTAAAAAAA
CAGTTGGTATTTCTTTTGCATTATTATTTATATTGCTTATTACTGCATTTAGTCTTATAGTTGTGCGTTT
TGAATATAAATTACAATTAAATGAGCAAAAAAACCTAATTCTTGAGGATACTCGTTTAGATGAACAATGG
AGTCAGATTGTATTAGAATATAGTTCTTTAGCAACTCCTACAGCTGTGGAGAAGTTTGCTCAAAAAGAAA
AAATGACCTTGCCAACACGTAAAACAATAGGCTTTTTGAATGAGCAAAAAGAGGAGTTAAATAATGAGTA
G

>lcl|NC_006570.2_cds_YP_169713.1_612 [gene=ftsI] [protein=peptidoglycan synthetase] [protein_id=YP_169713.1] [location=715872..717566]
ATGAGTAGTTATCGACCAAAACTCCGGCACTTTGTTGTAATTATTTTACTGTTGCTAAGTTTTATTGTTT
TATTTATAAAGCTTATTTATATGGAAACTATCCAATATCCTAAGCTTAAGCAAGAAGGCGATAATCGTAG
TGATAGAAGTATAGATATAAAAGCATACAGAGGTATAATCCTCGATAGAAACGGTAATCCTTTGGCGATA
AGTACACCTGTTGATACAATTTGGGTTGATCCTTTTTATATTAGCGCGGATAGTCCTGAATTAAATAAAG
TCATGAATATTTTAAATCTACCTGAAAAAACTAGAGAAAAGATAAAAAGTCAAGTTAAGGTTAGAGAAGG
AAGAAGTGGTTTTGTCTACATTGAGAGAAAGGTTCAACCATATTTATCACAAAAAGTTAAAGACCTTGAT
ATTACCGGTATTCATGTGATACGAGAGTTTAAACGCTATTATCCAATGGCTGAGGTAGCTTCTCATATTG
TAGGTTTTACTAATGTTGATGGTAAAGGGCAAGAGGGTTTAGAACTTGAGTTTAATAAGTTTTTAAGTGG
TCAAGATGGTTATTTTGAATATAAAAAAGATCTTCATGGTGGGGTTGCTTCTAAGATTGAGGATAAATAT
GTTGAGCCTAAGAATGGTCGTAATCTGCAGATAAGTATTGACTCTAGATTGCAATATATTGCGTATAAAT
ATCTCAAAGAAGGGGTTATTAGGACTAATTCTGAAGCAGGTTCTGTGATAGTTGAAGATATTCATATTGG
TGAGATATTAGCAATGGCAAATTACCCTTCGTATAATCCTAACAGCATGGCTGATGCCTATCCTGATAGG
AGAAGAAATAGAGCTATTACAGATGTCTATGAGTTAGGTTCGGTAATGAAAACTTTTGCAGCAGCGACAG
CTCTAACTTATGGTGATAATGTAACTTCAGATGAGCCTGTAATTGATACTCACCCTGGATTCTATCGTAT
CGGTAAAAACACCGTAAGAGATGAACGAGATTATGGTGATATTAATCTAAGACATATCTTAATGAAGTCT
AGTAACGTTGGTGTCTCGAAGATGATTTTAGGTTTGACAGAGCCAAGTATATTAGAGTCTTCATTAAGAA
ATTTTGGTTTTGGTAGTAAGACTGGAATAAAACTACCTGGTGAAAGAGATGGTTATGTTCCAACTAAAGA
TAAGTGGGGTGATTTCCAACTAGCGACATTATCTTTTGGTTATGGTATGAATGCTACTGATTTGCAGCTA
ATAGCTGGTGTATCCGCAATTGCTAATAATGGTCAATATATCAAGCCAACTATCCTAAAAAGAAGACCTG
GAGAAGAGATTGAAACAAGACCTATCATATCTGAGAAGAATTCAAAAGAAATGATAAGTATGATGCAGTC
TGTTGTTGAAGACCTGGGAGGAACTGGTTCTAAAGCACAAATTCCTCTTTATCATGTTGCTGGCAAAACA
GGTACAGCTAGAATGCTTTCTGGTGGAAATTATGGAGCAAAATATTTGGCAAGTTTCGTTGGTATTGTTC
CAGCAACTGATCCTAAACTAGCTATAGTTGTGACAATCAAAGATCCTAAAGGAGATCAATATGGTGGCGG
TTCTGTAGCCGCGCCAGTTTTTGCTGATGTTGCATTAAATAGCTTGCAAATACTAGGTGTTAAGCTAGAT
AAGATTGAAAATTAA

>lcl|NC_006570.2_cds_YP_169714.1_613 [gene=rpsO] [protein=30S ribosomal protein S15] [protein_id=YP_169714.1] [location=717672..717938]
ATGTTAACAGCTCAAGATAAGCAAAAAATAATTAAAGAAAATCAATTAGCAGAAAGTGATACTGGATCGC
CAGAAGTGCAAGTAGCTCTTTTAACTGCTAGAATCAATGATTTACAAGGTCATTTTGAGGCTCATAAAAA
AGATAATCACTCAAGAAGAGGTCTTTTAAGATTAGTAAGTCAACGTCGTAAGTTATTAGATTATCTACAT
GATAAAGATGTTGAAAGATACCGTAGTCTAATCAAGAAATTAAATATACGTAGATAA

>lcl|NC_006570.2_cds_YP_169715.1_614 [gene=pnp] [protein=polynucleotide phosphorylase] [protein_id=YP_169715.1] [location=717995..720076]
GTGAAAATATTTAGAGAAGTTTTTGAACTAGGTAACAAAGAAATAATTCTTGAAACAGGTGGTATGGCAC
GTCAAGCAGATGGGTCAGTGACTGTAAGTTGTGGGAATAATGTTGTATTGGTGACAACAGTAGTTAAAAA
ATCAGTAGCTGATGGTACTGACTTTTTTCCTTTATCTGTGCATTATTTAGAGAAAACTTACGCTGCTGGT
AAAATACCGGGTGGTTTCTTAAGAAGAGAGGGAAGACCTTCAGAAGAACAAATTCTTATTTCTAGGCTAA
TAGACAGATCTATAAGACCATCATTTCCAGATGGGTTTTTAACGAAATTCAAATCGTTGCTACAGTTTT
ATCTTATGATGGTGCTTTTTCACCTGATATACTAGCTTTAATAGGTGCTTCGGCATCTTTAGCAATATCT
GGAGCACCTTACGATGATGTTGTTGCTGGTGTAAGAGTGGGTTATACAAATGGTAAGTATATTCTTAATC
CAAATAAACAGGATTTGAGAGATTCAGATCTAGATCTAGTAGTCTCTGGAACAGATGATGCTATTTTAAT
GGTTGAATCTGAAGCTAATAGCCTTCCAGAATCTGTTATGCTTGGTGGTATCTTATATGCGCACAAGCAT
TTAAAAACTATTATTAACTCGATTAATAGACTTGCTAAAGTAGCATCTAAACCTCGTATAGAATATTCTA
TATATCAAATCAACAAGTTTCTTAAATCACAAATTAAGTCACAATTTTTTGGTGAAATTAAGAATGCCTA

TACTATAGCGTCAAAGCAAGAAAGAAACTTAAAACTTAATGCAATTAGAAAAAATGTTCTTGAGTATATT
TTCTCTAGTGATGTTGATGGTAACGAGTATACTGAAAAAGAAATATTAGAAGCTTTCCACGATATTGAAA
AAGATCTTGTAAGATCAAATATTCTTGAGGGTAAACCAAGAATAGATGGTAGATGTACAGAGACTATCCG
ACCTATCAATGTCAAAATAGGTGTTTTACCGGGGGTACATGGATCTGCTTTGTTTACACGTGGTGAGACT
CAAGCTTTAGTTGTAACTACTTTAGGTAGTGATAGAGATGCACAGCTTGTGGAATCTTTAGATGGCATAG
AGAAATGTCGTTATATGTTGCATTATAACTTTCCACCATATTCTGTGGGAGAGTGTGGTATGGTTGGTAT
GGCTCCAAAGCGTCGCGAGATTGGTCATGCTAACTTAGCTAAGCGCGCAACTCAAGCAGTATTCCCTAAT
GAAGAAGCATACCCGTATGTTGTTAGAGTTGTCTCTGAAATTCTAGAGTCAAATGGTTCAAGCTCTATGG
CGACAGTATGTGGTTCTTCTTTATCAATGATGGATGCTGGTGTACCAATTGCTGAGCCAGTAGCAGGTAT
TGCTATGGGACTTATTAAAGATGGTGCAAAATATGCTGTGTTATCAGATATTCTAGGCGATGAAGATCAT
TTGGGTGATATGGATTTTAAAGTTGCTGGTACTAGATATGGTGTTACAGCATTACAGATGGATATTAAAA
TCAAAGGCATCTCAAGAGAAATTCTTGAGCAAGCATTAGAGCAGGCAAGAGCAGGTAGATTACATATTCT
AGGAATCATGAATGAAGTAATAAAAGAGCATAAAGAAGCTGTATCTGATGTGGCGCCACAAATTCATGTG
ATGAATATAAATCCTGCTAAAATTAAAGATGTCGTTGGTCGAGGAGGTGCTACTGTAAAAGGTATAGTTG
AGAAGACTGGTGCACAAATTGATACTAGTGATTCTGGTGAAGTTAAGGTTTTTGCTAAAGACAAAAAATC
TATGGATATGGCTGTAGCAATGATAGAAGAAATCGTTGCAGAAGTGGAAGAAGGGCAAGTTTACAAGGGC
AAAATAGTTAAGCTTTTAGATTCTGGAGTATTTGTTAATCTTCTTGGTAGTCAAGATGGATATTTACCAT
TCTCTGAAATTGAACAAGCTGGCATGAAAACTAACTCGTTAGTAGAAGGTCAAGGCTTAGAAGTTTTAGT
TCAAAATATCGATAGAGGTGGTAGAGTTAAACTTTCTCTTGTAGCGAGGTAA

>lcl|NC_006570.2_cds_YP_169716.1_615 [gene=FTT_0700] [protein=iron-sulfur cluster insertion protein ErpA] [protein_id=YP_169716.1] [location=720082..720432]
ATGAGTGAAGTAGTTCAAAGCGTTGATCCGATAAACTTTACAGAAGCCGCTTCTTTAAAGGTTAAAGAAC
TAATTGAAGAGGAGGGTGATAATTCTCTTAGCCTTAGAGTATACATCACCGGTGGGGGCTGTTCTGGGTT
TCAATATGCTTTTGCTTTTGATAATGAAGTAAAAGAAGATGATATGGTTATTACTAAAAATGGTGTTCGC
CTTTTGGTAGATTCTATGAGTTTTCAGTACTTGGTTGGTGCTGATGTTGACTACAAAGATGATGTCGAAG
GTGCATACTTTGTAATTAGAAATCCAAATGCGAAAACTACTTGTGGCTGTGGTTCATCTTTTTCTGTATA

>lcl|NC_006570.2_cds_YP_169724.1_623 [gene=eno] [protein=phosphopyruvate hydratase] [protein_id=YP_169724.1] [location=727627..728997]
ATGTCGTCACAAATAAAACAAGTTTTTGCCAGACAGATATTAGATTCGCGTGGTAATCCTACAATTGAAG
TAGAAGTGGTTTTGGAAAGTGGTGCTTTTGGTCGTGCTGCTGTACCTTCTGGTGCTTCTACCGGAATTAG
AGAAGCTCTAGAGTTAAGAGATGGTAACAAAGCCCTTTTTCTAGGTAAGAGTGTATATAAAGCTGTTGAG
AATGTTAATACTAAGATAGCTCAAGCAGTCAAAGGTTTAGATGCATTAGATCAAAGGTTAATTGATAAGA
CTATGATTGAACTAGATGGTTCTGAGAATAAGAAAAATCTAGGTGCAAATGCAATTTTAGGTGTTTCACT
AGCTACTGCTAGAGCTGCTGCATCACATCTTAGAAAACCTTTTTACCGTTATCTAATGGATGTCAAAGAA
TATCTAATGCCAGTACCAATGATGAATGTTATTAATGGCGGTTCACATGCTGATAATAATGTTGATATGC
AAGAATTTATGATTGTTCCAGCTGGTTTTGATACTTTTTCAGAAGCTCTAAGATGTGGTACAGAAGTTTT
CCACATACTTAAAAAGGTTCTAATTGCTGATGGTTACAGTGTCGCTGGTGTTGGTGATGAGGGCGGTTAT
GCTCCTGATCTACCGTCAAATGAGGCGGCTATAGAGGCAATATTAAAAGCAGTTAAAGAAGCAGGTTATG
AGCCTGGTAAACATGTATTTATAGCTTTAGATCCTGCAAGTAGTGAGTTTTATAAAGATGGTAAGTACGA
ACTTAAGTCAGAGAATAAGTCATTAACAAGTGAAGAAATGATTGATTATTATGCTGCTTGGGTTGAGAAG
TATCCTATAGTATCTATAGAAGATGGACTTGCAGAAGAAGATTGGGCTGGTTGGAAACTTTTAACTGAAA
AACTTGGTAACAAGGTACAGTTAGTCGGTGATGATTTATTTGTTACTAATCCAAGTATCCTTGCTAAAGG
TATTGAAAAAGGTATTGCTAATTCAATTTTAATTAAGCTAAATCAAATTGGTACTTTGACAGAAACTTTC
GAAGCAATGGCAATGGCTGGTCAAGCAGGATACACTTGTGTGGTATCGCATCGTTCTGGTGAAACTTCTG
ATACAATTATTGCTGATTTAGCAGTAGCTACATGTTCTGGACAAATTAAGACAGGGTCATTATCTAGATC
TGATCGTATAGCTAAGTATAACCAGCTGCTTAGAATCGAAGAAGAATTAGGTGAAAATGCAATTTACCCA
GGGATAAAAGCATTTGTATTTAATTCAGATGAAGAAGTAGAAGAAGTTGTTCAAGAAATTATTGTAGAAG
ATAGTGAAGCTGAGAAAGTTGTAGTTCAAGTAGAAGAATAA

>lcl|NC_006570.2_cds_YP_169725.1_624 [gene=ftsB] [protein=cell division protein] [protein_id=YP_169725.1] [location=729005..729295]
ATGGATATCAAATCTAACTCTTTTTTTTATATTTTCATTTCTGTAGTTTTATTACTAATAGCAATATTGC
AATATGATCTGTGGTTTAGTAATACAGGCTTTATTAAGTATCAAGCACTAAAAAAATCTGTAATTAGCCA
GCAAAAAGAAGTAAAGCATAAATCTCAGACTAATGTACAATTATATTCTGAAGTGGTTTCACTACGTCAA
AATAGTGAGGTGCTTGAAAGCTTAGCTCGTGAGAATATGGGCCTAATCAAGCAAGGAGAGGTTTTTTATA
GTGTCAAATAA

>lcl|NC_006570.2_cds_YP_169726.1_625 [gene=ispD] [protein=2-C-methyl-D-erythritol 4-phosphate cytidylyltransferase] [protein_id=YP_169726.1] [location=729285..729974]
GTGTCAAATAAATACGTAATTATTCCAGCTGCTGGAATAGGTACTAGGATGCAGTTAGATATTCCTAAAC
AATACTATAAACTTAATAATGGTAAGACTATCCTTGATAATACCTTAGTAAAGTTTATTGATAATCCTTT

GTTTGATAAGATCTTTGTTGCAATTGCTGCTAGTGATAATTTTTGGAATAATTCGTTATATTATAATCAT
GACAAAATAGTAGTTTGTAATGGTGGAGAGACTAGATTTAATAGTGTTTATAATGCGCTAAAGGTCATTG
ATGAACGCAAAAATGATGATTGGGTTTTTGTCCATGATGCTGCCAGACCTTGTGTTAGTATTGATAGTAT
TATAGATTTGTATGAGCAGACTAAATCATCACACTCACAAGCAGGTATACTTGCTGTGAGAGCGTATGAG
ACAGTTAAGCAAGTTACAAAGAATATAGTTGTCAAAACACTGGCTCGTAATAATATTTGGCTTGCACAAA
CACCTCAATTATCTAGGCTTGGACAGTTAGAGAAAGCTTTTGATTTTTGCTATTCAAATAATCTTGTTGC
TAAAGTAACTGATGAAGCATCAGCTTTAGAAATGTTTGGTATAAATCCGATTGTTGTTGAATGTTCAAAG
AAAAATATTAAGATTACAACTAAAGATGATTTAGAATATGCTAATTGGCAGTTAGGTTAA

>lcl|NC_006570.2_cds_YP_169727.1_626 [gene=sdaC1] [protein=serine transporter] [protein_id=YP_169727.1] [location=complement(729979..731241)]
ATGAAAAAACCAGATTACTCAAATACTACAAAAGTCGATATCCAGTGGGTATTTACGTTATTTGGTACTG
CAATAGGTGCAGGGTTACTTTATCTACCTGTTCAAGCAGGGGATAGCGGCCTTTGGGCTTTAGTAACAGT
ACTTATTTTTGCTTTACCTTTAACGTATTACTCACATAAAAATATGTCAAATATTGTACTTTGCACAGAT
AATGGTGGTATTACAGATGTATTTACCCACAATCTTGGTAGATTTTTTGGTTTAACCTGCGTAGTGCTAT
ATTTCTTTGCAATATTCCTAAATATGCCAATGTACTCTATTGGTCTAAATAGCGAACTAAGTAATTTTCT
TTTAAACTACAATATTGTAAAAACCAATTTATCAACACATATATGGTTTAGTTTTAGTATTTTAGCCGTT
TTATTAATAATAGTTTCTTTAGGTATCAATATAATTCTAAAGTTTATGCAGCTTATAGTTATATTATTGA
TTATATTAGTAGTGACACTGTCCATATATATAATACCTTATTGGAACTTTGAGTTTATAACTGATAGTCA
TTTTGATACTGTTGGTTATATTACAGGTGTATTAATGGTATTACCAATCTTGATATTATCGATGAATCAC
TCACCTGTTATTTCTAATCTAGTAATTTTTTATCGTGATTATGTAAAAGTTGAGCGATCACAAGAAAAAT
ATAAAGTTTATAAAATACTTAAAATTAATGCTCTAATTCTTTTTATCTTTGTACTGCTTTTTGTAACATC
TTGCCTACTTAGCACTACTATACCAGACCTTAATAGGGCTAATGCAAATAATCTAACAATAGTAACTCTA
ATACAAGAGCAGCATCATAGTACTTTATTGAATATTTTAGCACCAATGATAGTTTTCACTGCTATTATTA
GTTCATTTATAGGCTGTTACATAGGTTCAAAAGAAGCTTTGAAATATCTATTTAAATATTTCTTTAAAAA
TATTTATAAAATAGAATTTTCTGATTCATTAATAAATAAAATCTGTGTTGGGCTAATATTTATAGTTCTC
TGGATATGCACAATATGTAATTTTAAAATCCTTAATATTATAGGCATATTAGTTGCGCCTACTGTGGCTT
TTCTACTATATGTTTTACCTGTAATAATAATTTACAAAAATATCCAATGTAAAGATTATAGACGCGTAAT
ATTAGACTCTATTTTATTTATAATGGGATTAATAATTATATTTGGCCATGTAATTGGTTTACTTCTAAAA
TAA

>lcl|NC_006570.2_cds_YP_169754.1_649 [gene=FTT_0743] [protein=hypothetical protein] [protein_id=YP_169754.1] [location=766572..767387]
ATGAATAAAACTATAAATTTACATAAAATGAAAACTTCTCTAGATCCGGCAAATAATGCTCGCTACGAAT
TAGAAGATATTTGTCTAAATGATTATATTGGTCAGCAGCTAAAACTTGAATTTCTTGATGAAATTAATTG
TGTCGCTTGTGGAGCCAAAACAAAAAAAAGCTACTCACAAGGGTATTGTTTTATGTGTATGCGAAGATTG
CCTGAGTGTGATATATGTATCGTTAAGCCAGAGTTATGTCACTTTGCCGCAGGGACATGTAGAGATTCAA
GCTGGGGCGAAGAAAATTGTATGAAAACTCATATAGTATATTTAGCAAATACTGGAGATATCAAAGTTGG
TATTACTAAGCTAAAAAATATTCCATCACGTTGGATAGATCAGGGTGCTAGCCAGGCTATTCCAATTTTT
GCTGTCGAAAGTAGGCTAATATCTGGTCTGGTTGAGGTCGCTATCAAAGAGCATATATCAGATAAAACAA
ATTGGCGTAAAATGCTCCAAGGTGAGCCTGATAATAATATTGATTTTGTGACTTTAAGAGATCAACTTAT
CGAAAAATCTAGCCAAAAAATAACTGAGATAAGAACCAAATATGGTCAAAATAGTGTTGAGCCAGTAGAA
GCTGAGATACAAAATATAAATTATCCTATCCTTAAATATCCAACCAAATTAAAATCTTTCAATCTAGATA
AAGATCGTCTAATTGATGCAAAACTCATTGGTATAAAAGGGCAATATCTAATTTTTGATAGTGGTGTGAT
AAATATCCGCAAGTTTAGTGGCTATAAGTGCATTTTATCTGCGTAG

>lcl|NC_006570.2_cds_YP_169774.1_665 [gene=glyS] [protein=glycyl-tRNA synthetase subunit beta] [protein_id=YP_169774.1] [location=782778..784862]
ATGAGTAAAATTACAAAAGATTTTTTATTTGAATTGGGTACTGAAGAGTTACCACCAAAAGCACTAAGAA
ATTTAGCCCAGTCTTTATTAGTCAGTGTTGAAAGTCAGCTAAAAGAGGCAGAAGTTAGTTTTGGTGATAC
TAAATGGTTCGCTTCACCAAGAAGGTTATCTTTTATAATCAAAGGTCTAGCAGAGTCTCAACAAGATGTA
GTTATTGAAAAGCAAGGACCGTTGGTAAGTATTGCTTATAAGGAAGGTGAACCTACTCAAGTAGGTTTAG
GTTTTGCAAAATCTTGTGGTGTTGAGCTTGATGAATTAGAAAGGGTAGCTACACCAAAAGGTGATAAGCT
TTTTTATAAAACAGTACAATCTGGTCAAGCAACAGTTAATTTATTGCAAGAAATTATTACCAAAGCGCTC
AAACAATTACCAATTTCAAAGATGATGCGTTGGGGCTCTTCAAATGTTGAGTTTGTAAGACCTGTACATT
GGGTATTAGCATTATATGGTAACGATGTTGTCGATATAGAAATTTTAGGACATAAAGCTGCTAATATTAC
ATATGGACACAGATTTCATCACCCTCAAGCTATAGTAATTGATAATATTAGTGATTATATCAAGCTACTT
GCTGATGCTATGGTGATTGTAGATTGGCAACAGAGCAAACAAATGTTAGTTGAACAAGCTGAAAATATTG
CTAAAGAAAATGATTATCAAGTAGTTCTAGACAATGATTTAGTCGAAGAGGTTTGTGCTATAGTTGAGTA
CCCAAACGCAATGTTATGCAGTTTTAACAAGGATTTCCTAAGAGTTCCTCAAGAAGCGTTAATATCTGCT
ATGGAAGAACATCAAAAGTGTTTTGCTCTATTGGATAATCAGGGTGATTTGGTTGCTAATTTTATTACAA
TTTCAAATATCCAAAGTAAAAAGCCAGAACTAGTAACATCAGGTAATCAAAAGGTAATGAATGCTAGGCT
AGCAGATGCGGCTTTTTTCTATGATACTGATTTGAAGACTTCTTTAGAACAGTTGTTGCCTAAGCTTGAG
CATGTAACTTTCCAAAGTAAACTTGGTAATATGTATCAGAAAGCTCAAAGAATAGCTAATATAGCACAAC

AGTTAGCAGAACTAGGTAATTTTGATTCTCAACAAGCACATAGGGCGGGTCTTTTAGCTAAAGCTGACTT
AATTTCAAATATGGTGTTTGAGTTTACAGATTTACAAGGAATTATTGGTAAATATTACGCAAAAGCTCAT
GGTGAAACAGATACAGTAGCCGAGGCAATAGAGCAACAATATTGGCCAAAGTATTCTGGAGCGGAGTTAC
CAAGAACTAATGTTGCTGCATGTGTAGCATTAGCTGAGAAATTAGATACTTTAGTAGGTATATTTGCAAT
TGGTCAGAAACCAACAGGAAATAAAGATCCATTTGCATTAAGAAGATCAGCTATAGGTATCTTGCGCATA
CTAAGAGATACAAGTGTTGATATATCTTTAGAAAAGATTATTGATATAACTTTAGAAAGTTATAAAAAAA
TTAATAATTTAGAGTTTAATACTGATGTAAAAACAGAAGTAATATCTTTCTGTCTAGATAGACTAAAAAA
CCTTTATAAAGAAGAGGGTATTGCTGTAGATATTTTTGAAGCTATTAATAATACTAATTATGATTCGATC
AAAGATTTTGCTGCGAGAGTAGAAGCTGTAACTAAGTTCTACAATTCAGATAAAGCTCAGAGTCTAATAG
CTTCAAATAAGCGTGTAGCTAATATTCTTAGCAAGAATGCTACAGATAAAGATTACTATTATAATATTGA
ACTTGCTAAAGCAGCTGCTAATGAATATGAGTTAGCACTTGCTTATAGTATCGAAGAGGTTGCTGCTGAT
CTTCATAAGTACCTTAATAATCGAGAATATAGTTATGCTCTAGAGCTGTTAACTTGTCTTGATAAAGTGA
TTAGTGAGTTTTTTGAGAATGTAATGGTAATTGATGAAGATATTAAGATAAGAAAAAATAGACTTGCTTT
GCTTGTAAACCTGCATAAGATGTTCATTGGAATTGCTGATATCTCTAAATTATAG

>lcl|NC_006570.2_cds_YP_169779.1_670 [gene=secA] [protein=preprotein translocase subunit SecA] [protein_id=YP_169779.1] [location=788287..791007]
ATGTTAAGTTTAGTACAGAAAATAATAGGTAGTCGTAACGAAAGATTTATAAAAAAAGTTTCTAGAATAG
TTCAAAAAATTAACTCTTTAGAACCTGAGTTTGAGAAACTTAGTGATGAGCAATTAAAAGCAAAAACTTT
TGAATATCGCGAAAGACTTGCAAACGGTGAAATATTAGACAATCTTTTGCCAGAAGCTTTTGCAACCGTT
AGAGAAGCTGGAAAGCGTACTAAAAATATGCGTCATTATGATGTTCAGCTGATAGGTGGTATAGTTCTTC
ATCAAGGTAAAGTTGCTGAGATGAGAACAGGTGAGGGTAAAACTTTAGTTGCCACATTACCAGCTTATCT
AAATGCTTTGACTGGTGATGGTGTACATGTAATTACAGTTAATGATTACCTTGCTAAGCGTGATGCTGAG
CTAATGAGTGATATTTACGAATTTTTGGGAATGTCTGTAGGTGTAATAGTTGCTGATTTAAATCCCCAGC
AACGTAAAGAAGCTTATGCATGTGATATCACTTATGGAACAAACAATGAATTTGGTTTTGATTATCTAAG
AGATAATATGGCTTATGAAAAAGAGCAGCAAGTTCAAAGAAGCCGTAACTATGTAATCATAGATGAGGTT
GACTCAATTTTGATTGATGAGGCTAGAACACCACTTATCATATCAGGTGCCTCAGATGATAGATCTGAGA
TGTATAACCTTTTCAATAGATTAGTTCCGTACTTAGAAAAGCAAGAAAAAGAAGAAGTTGAAAATGAACA
AGAGCAAAGAGATTTTTATGTAGATGAAAAATCCAAAAATGCTTATCTAACTGAAAAAGGTTATGCAAAA
ATTGAGAATATGCTCAAAAAAGAAGGTATTCTTGAAGAAGATGATAACCTTTATAGCCCTCACAATATTA
CAAAAATGCATTACTTAAATGCATGTCTAAGAGCTCATTCGTTGTATCAACTTAATATTGATTATATTGT
GAGAGATCAGGAAATAGTTATTATTGATGAAAGTACTGGTAGGGCAATGCCTGGTCGCAGATGGTCAGAT
GGTTTGCATCAGGCAATAGAGGCTAAAGAGGGCGTCAAAATTAATGCTGAAAATCAAACAATGGCATCTA
TTACCTTCCAAAATTTCTTTAAATTATATAACAAAATTGCTGGTATGACTGGTACTGCTGATACTGAGGC
ATTTGAGCTTCATTCTATCTATGGTTTAGAGGTAATTATTATACCAACTAATAAACCGATGATTAGAAAA
GATCATCATGATGAGATATATGGTAGTGTAAGAGAGAAATTTGATGCTATAGTTGAGGATATTAAGGAGA
GAATTTCAAAAGGTCAGCCGGTACTAGTTGGTACAGCATCTATTGAAGCATCTGAAGTATTATCAACGTT
GTTGAAAAAGAAAAAAATCAGACATAATGTTTTGAACGCTAAACAACATGAGAAAGAAGCTAGTATTATT
GCCATGGCTGGCTACCCAGATAATGTGACAATCGCAACGAATATGGCAGGTCGTGGTACAGACATTATTT
TAGGCGGTAATTTAGAGGTTGAAATAGCACAGCTTGAGGATCCTACACCAGAAGATATTGCTCAAATAAA
AGCAGAATGGTTAAAGCGTAATGAAGCTGTTAAGAAAGCTGGTGGACTATGTATCATTGGTTCAGAAAGA
CATGATTCCCGCAGGATCGATAATCAGCTAAGAGGTCGTGCTGCTCGTCAGGGTGATCCTGGTGAGAGTA
AATTCTATCTATCTATGGATGATAATCTTTTGCGAATTTTTGCTTCTCAACGTATGGCAGAAAGGGTTAA
AAAAGGTCTAAAAGGTGGTGAGTCACTAGCTTTTGGGTTTATGTCAAAAGTTATATCAAAAGCACAGGGT
AAGGTAGAGAGTTATCATTTTGATATCCGTAAGAATCTATTAGAGTATGACAATGTTGTTAATACACAAC
GTAAAGTTATTTATGAGCAAAGACAGTCATTCTTAGAAGCTGAAGATGTTAGTGATATTCTTGCTGATAT
TCGTATTGATGTGGCTGAGCAGTTGTTTCATGATTATGTACCTGCTGGTTCCATGCATGAACTGTGGGAT
CTTGAAGGCTTAGAGAAGGCACTTAAATCTGACTTTATGATTGAGCTTGATCTACAGAAGCTTTATGAAG
AAGATGATAGCTTAGGAGAAGAAGATCTTAAGAGGCTAGTAAGAGAAGCTATAGAAATTGAATTTGTTGA
AAAAACCAAGAATTTAGATTCAGGCGCTGTGAGACAATTTGAGAAATTCTCATTATTACAATCTCTTGAT
ACTCATTGGCGTGAGCATTTGAGTTCAATAGACCATTTACGTAATAGTATAAATCTACGTGGTTATGCTC
AAAAAGATCCAAAAAATGAGTATAAAAAAGAAGCCTTTGAGCTTTTCTCAACTATGCTAGATAATTTCAA
ATATGAGGTTATATCTTCACTTGCTAAGATTAGAATTGCTACTGAAGAAGAAACGCAAAGAGCTCAGCAA
GAATGGCAAGAATCTATGAGTGATATTAAAGCTGAGCATGAAAGTGTGATTGATAACAATCAAAGACATG
ATGAGGATGAGCAAGAAGAGGCTCCAAAAGTTAAGCAAGTTAGAAGAGAAGGTCCAAAAGTTAAAAGAAA
TGATCCATGTCCTTGTGGCTCAGGTAAGAAATATAAACAGTGTCATAGTAAGGTTGAGTAA

>lcl|NC_006570.2_cds_YP_169780.1_671 [gene=rplU] [protein=50S ribosomal protein L21] [protein_id=YP_169780.1] [location=793076..793390]
ATGTACGCGATAATTAAAAATGGCGGTAAGCAATACAAGGTAAAAGAAGATGAAGTAGTTAAGCTTGAGA
AGTTTGACCTTGGTATTGGTGAGAAAGTTGAGTTTGATACAGTTTTAATGGGACAAACTGCAGCAGGAGA
AGTTAAAATAGGCGCTCCAACTGTAGCTGGTGCTAAAGTTGTTGGTGAAGTTGTTGAGCAAGGTCGTCAT
AAGAAAGTTAAAATTATGAAGTTCCGTCGTCGTAAGCACAGCATGAAGCAACAAGGTCACCGTCAGTATT
TTACAGCGGTTAAAGTTTCATCTATTAGTTTATAA

>lcl|NC_006570.2_cds_YP_169781.1_672 [gene=rpmA] [protein=50S ribosomal protein L27] [protein_id=YP_169781.1] [location=793421..793675]
ATGGCTCACAAGAAAGCTGGTGGTAGTACTAGAAACGGAAGAGATTCAAACCCTAAGTATTTAGGTGTTA
AAAGATATGGTGGTGAGTTTGTTAAAGCAGGTACAATCATCATACGTCAAAGAGGTACTAAAACTCATCC
TGGTGTAAATGTTGGTTGTGGTAAAGATCATACATTATTTGCACTTAAAGATGGTACAGTTAAGTTCCAT
ACTGGTGGCGCTTTAAATCGTAAATTTGTTTCAATCGAAGAATAA

>lcl|NC_006570.2_cds_YP_169789.1_680 [gene=fabI] [protein=enoyl-ACP reductase] [protein_id=YP_169789.1] [location=801553..802335]
ATGGGTTTTCTAGCAGGAAAAAAAATATTAATCACTGGACTTTTAAGTAATAAGTCAATTGCATATGGTA
TTGCTAAAGCTATGCATAGAGAGGGAGCCGAGCTTGCTTTTACTTATGTTGGACAGTTCAAAGATAGAGT
GGAAAAATTATGTGCAGAATTTAATCCAGCTGCAGTTTTGCCTTGCGATGTGATTTCTGATCAAGAGATT
AAGGATTTATTTGTAGAGCTAGGTAAAGTTTGGGATGGTCTAGATGCCATAGTTCATTCTATAGCTTTTG
CACCGCGTGATCAGTTAGAAGGTAACTTTATTGACTGTGTAACTCGCGAGGGTTTTAGTATCGCTCATGA
TATTAGTGCCTATTCTTTTGCAGCATTAGCTAAAGAAGGTCGTAGTATGATGAAAAATCGTAATGCTTCT
ATGGTAGCACTTACTTATATTGGAGCAGAAAAAGCTATGCCAAGTTACAATACTATGGGAGTTGCTAAAG
CATCTCTAGAAGCTACAGTTAGATATACAGCGTTAGCTTTAGGTGAGGATGGTATCAAGGTAAATGCTGT
ATCAGCTGGTCCTATCAAAACTCTGGCAGCTTCTGGTATATCAAACTTCAAGAAGATGCTTGATTATAAT
GCTATGGTTTCTCCACTTAAGAAAAATGTTGATATTATGGAAGTTGGTAATACTGTAGCGTTTTTATGTT
CAGATATGGCAACTGGTATCACTGGAGAAGTTGTCCATGTTGATGCTGGATATCATTGTGTGTCTATGGG
TAATGTTCTTTAA

>lcl|NC_006570.2_cds_YP_169796.1_687 [gene=rpe] [protein=D-ribulose-phosphate 3-epimerase] [protein_id=YP_169796.1] [location=807002..807670]
ATGAAACATATTCAAATTAATCCTTCTATACTCTCTGCAGATCTTGCCAGATTAGGGGATGATGTCAAGG
CAGTTTTAGCGGCAGGGGCTGATAATATTCATTTTGATGTTATGGATAATCACTATGTGCCTAATCTGAC
ATTTGGACCGATGGTGCTCAAGGCTTTGAGAGATTATGGTATAACTGCTGGCATGGATGTTCACCTTATG
GTTAAACCTGTAGATGCTTTAATTGAGAGTTTTGCCAAAGCTGGAGCAACTAGTATTGTTTTTCATCCAG
AGGCGAGTGAGCATATTGATAGAAGCTTACAACTAATCAAATCTTTTGGCATTCAAGCTGGGCTTGCTTT
AAATCCTGCTACAGGTATAGATTGTTTGAAGTATGTTGAGAGTAATATTGATAGGGTGCTGATAATGTCA
GTAAACCCTGGTTTTGGGGGACAAAAATTTATTCCAGCTATGCTTGATAAGGCTAAAGAAATTTCTAAAT
GGATTAGCTCAACTGATAGAGATATTTTGCTAGAGATAGATGGTGGCGTAAATCCGTATAATATTGCTGA
AATAGCTGTATGTGGAGTAAATGCCTTTGTTGCTGGTTCAGCTATTTTTAATTCTGATAGCTATAAGCAA
ACTATTGATAAGATGAGAGATGAGCTCAATAAAGTCTAA

>lcl|NC_006570.2_cds_YP_169808.1_699 [gene=FTT_0801c] [protein=carbohydrate/purine kinase pfkB family protein] [protein_id=YP_169808.1] [location=complement(819627..820733)]
ATGAAAAATTTCAAAGCTCTAACAATTGGTGGTGCAACTCTTGATACAATTATTGAATATGAAGAAATGT
TTACCATGAATATGCAAAAGAAAGACACTATTCAATCTTTTATGCTTCTTGAAGAAGGGGCTAAAATTGA
AGTGACTGATCAAAAATCCTTCTCTGGTGGTGGTGCAACTAATGCTGCCGTTTCATTCAAAAAACAAGGT
ATAGATGTCAGTTTTTTTGGCAAAATTGGTAAAGATATCGCTGGTGAACAAATTACACAAGAACTAGAAA
ATCATGGTATAGATACTTCTAATATTCGTTACTCTAATAAATATGGTACTGCTACTTCATATGTAGTACC
TACGTTAAGTGGTGATAGAACAATTTTTGCTTATCGCGGTGCTAACAAAGATATTCTAAAAGATGATCTA
CCATCACAAGCAATTATAGCGAGTGATTTTATATATATCACATCATTGAGCAAATCATCCGCGGCAAGAT
TACCCGAGATAGTTAAACTCGCGGCAGATAATAACACTAAAGTTGCTATTAATCCTGGCTCTAGTCAGCT
AAGCGTTGGTGAGAGTTTTATCAAAGATTCGATGTTTGGTATTGATATTCTTGTGCTAAACTTCCAAGAA
GCACAAAAAATTGATGTTGTCGTTATTATCTTCTGATGATAAAGACATTGTCGAATCTAAAGATAGATTAG
AAACACCTCTTTGCGAAGAAAAAAGTGACTTTTTAAACTCTACTTTTAGACTCAAAGATTTCTTTAGAAT
CTGCCTAGATCTAGGAGTAAGAATAATAGTAGTTACAGATGGCGCTAATGGAGTCTATGCTGCAACTAAG
GAAAGAATATATCATAGTGAGTCACTACGTATCAAAAATGTCATAAATACACTCGGTGCCGGTGATGCAT
TTAGCTCAACTTTCTGTGCAAATATTTACAAAGGTTGTAGTATCGAAGATTCTATAAAATTTGCTTTGAT
CAATGCAAGCCATGTAATCCAACACTCAGATGCAAAATCAGGTTTACAAACATCAAAAAGCCTTGAGAGA
ATAAAATCAAAAATGAATTGTGGTTTGGATAAAAAAATTACAGTTACTTCTTGGTAA

>lcl|NC_006570.2_cds_YP_169815.1_706 [gene=spoT] [protein=guanosine-3',5'-bis(diphosphate) 3'-pyrophosphohydrolase] [protein_id=YP_169815.1] [location=827816..829939]
TTGCTAAATATGTTTTGTTTCTATGACCTTAATCAATTAATTTCTCAATATCTTCCTCATCGAGAAAGGC
TAAAGATAGCTCAAGCATTTATATTTGGTGCTGATGCTCACGAAACTCAAGTTAGAAGCTCAGGGGAGCC
TTATTTTACCCATCCTGTTGCTGTAGCATGTATACTTGCTGAGCTTAGGATGGATGTTGATACAATAATT
GCAGCTTTATTGCATGATGTCGTAGAGGATACTGAGTATACAGTTGACGATATTAGTAATATTTTTGGTA
AAAAAGTTGCTCAACTTGTCGAAGGAGTAACTAAACTTACTCAAATTAGGCATAAAAACCGAGCGGAACA

ACAAGCAGAAAACTTCCGCAAAATGTTACTATCTGTGACAAAAGATGTTCGTGTAATATTTATTAAGCTA
GCTGATAGGCTTCATAATATGCGTACATTAGCACCACTAAAACCTGAGAAAAAGCGTCGAATTTCTAAAG
AAACTTTAGATGTGTTTGCACCTCTTGCTCATCGACTAGGTATCAATACTCTTAAAGAACAGTTGGAAAC
CCTTGCGTTTGAGGGCATGTATCCTTATCGTTATCATATTTTAGAAGAAAAAGTAAAAAAAGTAGAAAAA
AATAAAGAAAAAGTCTTTTACGAGGTAAAAGAAGCATTAGCAGAAAAGCTTAATGACTTAGTCTCATTAG
AAGATATCAAAGCACGTAAGAAAACTTTGTACAGTATATATAACAAAATGCGTAAAAAGGGTATATCTTT
TGATGAAATTATGGACATGTACGCTTACAAAATTATTGTTCCTAATAGAATAGATTGTTATGTTGCTCTT
GGTAAGGTTCATGAGTTATATAAGCCAATACCACAAAAATTCAAAGATTATATTGCAACACCAAAAGCAA
ATGGTTATCGTTCATTACATACGGTTGTCTTAGGGCCGTACAATATTCCATTAGAGATTCAAATAAAAAC
AGAACAAATGGATCGTCAAGCCGAATATGGTATTGCAGCTCATTGGAGTTATAAGATCGGTGAGAAAACT
GACAAAGCATTACAAAGATGGCTTAAGAAAATTTCTGATATTAATGTGTATACTGCGAGCTCTGTAGAGT
TTTTAGAAAATGTCAAAACAGATATTTTTAATAATGATGTGTTTGTATTTACTCCTCAAGGTGAGATTGT
TGAATTACCTATGAATTCAACTTGTATAGATTTTGCCTACTATATACATACTGATATTGGTAATAAATGT
ATTTCAGCTAAAGTAAACCGTAAGTCTGTTCCACTTAACTACAGATTAAAGCAGGGTGATAATGTTGAAA
TTATTACTTCAGCAATTGCTGATCCTAATCCGGCATGGTTAAAATTTGTTGAAACTAATAGAGCTAAATC
AGCTATCAAGGACTTCTTGAAACAGCAATATCGTAATATTGATTATATTCGTGGCAAAGATATAGTCGAA
GGTGAACTTAGACTTTTAGGTGTTGAACTAAGGGATGTTCCAAGTGAAATTATAGATGGAGCATTATTTA
ATTATGAAGGAATAGATACAATAAATCGCTTCTATCTAGATACTGGCTTAGGTCTTATTGATCCAAATAA
CTTTATTGATTTCGTTGTTAAGCATTTTGAGGATATGCGTAACGCTTATAAAAAATCATCTTTATCAAAA
TTACAAATTAGATATGGTGATGATCCACGGATTGCTGATTGTTGTTTACCTCTGCCAAATGATGAGATAG
TTGGTATTGTTAACGAGCATGGCAAAGTTGAGGTCCATCGAAAAAGCTGTAATGAGTTGTATACAAAAAT
TAAGGATAATCAAACTAAAGAGATACAGGCAGATTGGTTTACAAATAGTGATGATGATCCTAGCTTTAAA
GCTAGACTTGCAGTTACACTCAAAAATATTCCAGGTTCGATAGCTAAAATTACAGCAACTTTAGCTCGAG
AAGGTGTTGATATTAGAAGTTTTGATATGATATCTGTAGATAACAAACAAGCTAAACTGTCATGTGTTGT
AGTAGTTAGAAATAGAAGAGAACTATATTTATTGGTTCGTTTAGTAAGAAAGTTAGATGTTTGTTTAAAT
ATCGAAAGAATATTAAATAAGTAA

>lcl|NC_006570.2_cds_YP_169818.1_709 [gene=murG] [protein=undecaprenyldiphospho-muramoylpentapeptide beta-N- acetylglucosaminyltransferase] [protein_id=YP_169818.1] [location=complement(830897..832012)]
ATGAGTCTAGAAAATAAAAACATAATTATAACAGCTGGTGGTACAGGTGGTCATATATACCCCGCACTGG
CAATAGCTGAATTGCTCAGACAAAATAAAGCCAATGTGACTTGGGTTGGCACTCCTAACAATATGGAGGC
TTCAATAGTTCCCGAGTATTTTAATATTCAATTTATTAAATCATCCGGTGTTAGGAGAAAAGGGATTATA
AAAAAAATTACTTTTCCGCTAAAGCTTGCCTACAATACCTTAAAATCGCGTAGTCTATTAAAAAAGCTTA
AAGCAGATTTAGTGATTGGTTTTGGTGGTTATGTTTCTGGGCCAATATGTCTAGCAGCTGCTCAAATAAA
TATCCCAGTAATAATTCATGAACAAAACGCTAAAATAGGTCTAACAAATCGTATATTAGCTAAATTTGCA
ACAACTATATGTCTAGCTTTTGAGATAGAAAATCTCCATAAACAATTTAGCTCCAAACAATTAGCAAAAA
CAAAAATTGTCGGCAACCCAGTCCGTAAAGAGATTGTAGCACTTAATGATAAAGCAAGAATTTACACAGA
CTCATCAACATTAAAAATATTAGTTTTAGGTGGTAGCCAAGGCGCTAAAGCAATAAATGAAATCATTCCT
AAGTTAATTCAGAAATCAAATGAGCAAGGTATAAATATAAAAGTTTGGCACCAAACAGGTAAACTATCAC
TCGAAGAAACTAAAGATGCTTACAAAGATATATCACAAAACCATATCAAAGATATTGCTGCTTTTATTGA
TGATATGGCCATAGCATATAATTGGGCTGATTTAGTAATTTGTCGAGCTGGCGCATTAACTGTATCAGAG
TGTGCTATTGCTGGATTGCCAGCGATATTTATCCCTTTACCATCAGCAGTTGACGATCATCAATTTTTTA
ATGCTCAAAATATCGTCAATAATAATGCGGGATTTTGTTTAAGACAACAACAGATGACTTTGGAAAATTT
ACTCGCTATAATAAAACCTCTTAATCAAGATAGATCTAAGCTTGAGCAAATGTCAAAAATGGCAAAAAAA
ACATTGATAAAAAACTCAAGTGAACAAATATTAGATTGTGTAAAAAAAATCTTAAACAACAAATAA

>lcl|NC_006570.2_cds_YP_169824.1_712 [gene=thrS] [protein=threonyl-tRNA synthetase] [protein_id=YP_169824.1] [location=835100..837004]
ATGATAAATATCAGATTTCCAGATGGTTCGATAAGAGAATTTGAAGCCGGTGTTAATTCTCTAGATGTTG
CTAAATCAATATCTCCAAGCTTAGCTAAAGCTACAATGGCTGCGTATATTGATGACCAGCTCAAAGATGC
AAAAGATGCGATTAACAGTAACTGTGAATTAAGATTGATAACAGTAAAAGATCCAGAGGGTCTCGAAATT
CTAAGACATTCTTGCGCTCATCTTTTAGCTCATGCTGTCAAAGAGTTGTATCCAAATACTGAAGTAACTA
TAGGTCCGGTAGTTGATAATGGCTTTTATTATGATTTTTCTTTCAAAGAGTCTATAGGTGAAGCAGATTT
ACCTACTATCGAAAAGAAAATGAAAGAATTAGCAAAAAAGTCTGCTCCTATTAGTTATAGAGTGGTTCCT
AAAGCTGAAGCAATTGAGTTTTTTAAGGCTCAAGGTGAGAACTATAAGGTTGAAATTATTGATAGCATTG
CTGATGAGCAGATGAAAATATATACCCAAGATAATTTTAGTGACTTGTGTAGAGGTCCTCATATTCCAAA
TACATCAGTATTAAAAGCATTTAAGTTGACAAAACTAGCAGGAGCTTATTGGCGAGGTAACTCTGATAAT
GAAATGCTAACTAGAATCTATGGCACATGCTGGGCAACTAAAGAAGATTTAGAGCAATATTTGAATATGC
TTGAAGAGGCTGAAAAGCGTGATCATAGAAAAAATTGGTAAAGTTCTTGATCTATTTCATTTTCAAGAAGA
CTCACCAGGCATAGCTTTTTGGCATGATAATGGTGTGAGAATATGGCGTCAAGTAGAAGATTATATGCGA
GCTTCTAATAATAAATATGGCTGTAGTGAGATTAGAACCCCACTTATTGCAGATTTTAGTTTGTGGCAAA
AATCTGGTCACGCATCTAAGTATGCTGAGAATATGTTTGCAACAAAGTCTGAGAACAGGGATTTTGCTAT
CAGACCAATGAACTGTCCAACTTGTGTACAGGTTTATAATACAAAGCTACATAGTTATAGAGATCTACCT

ATAAGGATGGCTGAGTTCGGTATAGTACATAGGAATGAACCATCTGGATCGCTACACGGGTTATTAAGAG
TACGTAGCTTTACTCAAGATGATGGGCATATTTTCTGTACCCCTGAGCAGGTTGAAGAAGAAGTGATTTT
GATGGTACAGCAGTGTTTTGAAGTATACAAAGATTTTGGTTTTAATGATTTTGCGGTCAAGATTGCACTT
AGACCTGAAAATAGAATTGGAGATGATGAAACTTGGGATAAATCTGAACAGATGCTCAAAAATGCTCTTG
ACGCTAATAATGTTAGTTATGAACTACTTCCAGGTGAGGGTGCATTTTATGGTCCTAAGATTGAGTTTCA
TCTCAAAGATGCAATTGGTAGAAGCTGGCAGTGTGGCACTATACAACTAGATTTTTCAATGCCACAAAGG
CTAGGAGCTACATATATTGACAAGAATGGCGAGAAACAGGTGCCAGTTATGCTTCACAGAGCTATCGTTG
GTTCTTTGGAGAGATTTATAGGTATGCTTATTGAGCATTATGCTGGAAATTTACCATTGTGGTTAGCTCC
AGTCCAAGTTGCTGTGATGGGAATTAGTAATAATCAGGATGACTACTGTAAAGAAGTATTTATAATGCTT
GAAAAAAATGGTATCCGCGCTAAATTAGACTTGAGAAATGAGAAAATAGGGTTTAAAATACGTGAGCATA
CTCTTTTGCGTGTACCATACCTTGTTATCCTTGGTAAAAACGAGCAAGAGCAAAAGATTATCACAATAAG
AAAGCATAGCGGCGAAGATCTAGGACAGATGTCTGTAGATGATTTTTGTGCTTTTTTAGATAAGCAAATA
CAGGCTAAAGAATAA

>lcl|NC_006570.2_cds_YP_169825.1_713 [gene=infC] [protein=translation initiation factor IF-3] [protein_id=YP_169825.1] [location=837080..837547]
GTGCGTCTAGTTGGTGTTGATGGTCAACAAATAGGTGTTGTATCTATCAATGAGGCTTTAGCGTTGGCTG
AAGAGGCTGATGTTGATTTGGTTGAAATGGTAGCAAATGCTAATCCGCCAGTGTGTCGTTTGATGGACTA
CGGTAAGTACTTGTTTGAACAAGGCAAGAAAAAAGCACAAGCTAAAAAGAACCAAAAGCAGACTCAGGTG
AAAGAGGTGAAGCTCAGACCTGTGACTGATGTTGGGGATTATCAGGTAAAACTACGCAACCTGATAAAAT
TCCTTGAAAAAGGCGATAAAGTAAAAGTCACATTGAGATTTAGAGGTAGAGAAATGTCACATAAAGAGCT
GGGTATGGAAATGCTTCAGCGTATGGCAAATGATGCTGCTGAATACGGTGTGGTGGAACACCAACCTAAA
CTGGAAGGTCGCCAAATGATTATGGTTTTAGGACCAAAGAAAAAGTAG

>lcl|NC_006570.2_cds_YP_169826.1_714 [gene=rpmI] [protein=50S ribosomal protein L35] [protein_id=YP_169826.1] [location=837590..837787]
ATGCCAAAGTTAAAAACTAAAAGTGGTGCTGCTAAGCGCTTTAAAAAAACTGGTAAGGGTGGATTCAAAC
ACCGTTGTGCAAACCGTGCGCATATCAACACTAAAATGACAACTAAGAGAAAGCGTCATTTAAGAGGTAT
GAACCAAGTAGCTAAAGTTGATACTACTAGCTTAGTTCAACAAATGCCTTACGCGTAA

>lcl|NC_006570.2_cds_YP_169827.1_715 [gene=rplT] [protein=50S ribosomal protein L20] [protein_id=YP_169827.1] [location=837823..838179]
ATGTCAAGAGTAAAAAGAGGCGTAACAGCACGCGCACGTCATAAGAAGGTTTTAAATCAAGCTAAAGGCT
ACTACGGTGCTCGTTCTAGAGTATATAGAGTAGCTAAGCAAGCTGTTATTAAAGCAGGTCAATATGCTTA
TAGAGATCGTAAAGTTAAGAAAAGAACATTCAGATCTTTATGGATTGTTCGTATCAATGCTGCTGCTAGA
CAACACGATATTAGCTATAGCCAACTAATCAATGGCTTAAACAAAGTAGGTGTTGAATTAGATAGAAAAG
CGCTAGCTGAATTAGCAGTATACAACAAAGATGCTTTTGCTGCTGTTGTAGAAAAAGCAAAAGCTGCTTT
AGCTTAA

>lcl|NC_006570.2_cds_YP_169838.1_726 [gene=ispH] [protein=4-hydroxy-3-methylbut-2-enyl diphosphate reductase] [protein_id=YP_169838.1] [location=849840..850796]
GTGAAGATATTACTAGCTAATCCCAGGGGCTTTTGTGCTGGTGTAAGTCGTGCTGTTGAGACTGTAGAGA
AAGTTTTAGAAGTTGAGAAGTCGCCTGTATATGTGCGTCATGAGGTCGTACATAATAAGGTTGTTGTAGA
TTCTCTTAAGAAAAAGGGTGTGGTTTTTGTCAAAGAAGTTGATGAAGTACCAGATGACGCGGTATGTATC
TTTAGTGCTCATGGAGTTTCTCTAAAGGTTGAAGAAGCTGCTGCCAAGAAAAATCTAGTACTTTATGATG
CAACATGTCCTTTAGTTACTAAGGTACACAGAGGTGTACGTTTGGCAAGTAATAACGATGCTGAATGTAT
CTTGATTGGTCATAAAGGCCATCCAGAAGTTCAAGGTACTATGGGGCAGTATCGTAGCAAAAAAGGCGCG
ATTTATCTTATAGAAAGTGAAGAAGATCTTAATAAATTAACAATAAAAGATCCAGATAATCTATACTACG
CCACTCAGACTACTTTATCAGTAGATGAGACACAGGGGATAATACAGGCTTTAAAAGACAAATATCCAAA
TATTAAGGGACCTAAAAAAGAAGATATTTGTTATGCAACCCAAAACCGTCAAACAGCTATAAAAGCCATG
CTTAAACATATTGATGTTTTAGTTGTTGTAGGCTCACAAAATAGCTCTAACTCTAATAGACTAAAAGAGC
TAGCAACCTTAGAAGGCATAGATGCTTATCTTGTTGATAATCCTAAAGACGTTGATAAGTTATGGTTTGA
TAATAAAAAAGTCTGTGGTGTCAGTGCCGGAGCTTCTGCACCAGAATACTTGGTTCAGCAAATAATTAGT
CAAATATCTAAAGTTTGTTCTACAGAGGTTGAGGAGTTTGAGGGTATTAAAGAAGAAGTCTATTTCCCAC
TACCAAGACTTTTAAAGCAAAAGATTGGTACAGGTAAGGTAGAATAA

>lcl|NC_006570.2_cds_YP_169840.1_728 [gene=FTT_0835] [protein=CDP-alcohol phosphatidyltransferase] [protein_id=YP_169840.1] [location=851169..851990]
ATGATTATGTGGTTATTAGAAAATATAGACATGAAAAAATCAAAATATATACTACCTAGTTTGTTTACTA
GTGCTAGTTTATTATTTGCTTTCTTAGCAATTATAGCAGCCTTCAATGGTAATTTTGTCTCGTCTGCAGT
ATACATGCTTTTAGCAGGTTTTGCAGATGCATTTGATGGTAGGGTTGCTAGGTATACACACACTCAAACT
GAATTTGGTGCTGCTTTAGATAGTCTTGCTGATGTGGTTTCATTTGGATCTACTCCAGCCTTAGTTATGT
ATTTTTGGAGTTTGCATAATATTGGTGCTCTAGGCGCAGCTATTTCATTTTTGTACTTGCTTGCTGTAGC
ATTAAGATTAGCTAAATTTGATACTATGCCTGCTGGTGATAATTCAGAGGAAAATATAATTGAGCGTCGT

TTATATTTTTATGGAATGCCATGTCCAGCTGGTGCTATAACTATATCAGGATTAATCTGGATGGGGCAAA
GAACATTTGTTGGTGATTATGCTTTTATAACGGTAATTTTGACTGTTTTTACAGCTTTATATCTTGCTTT
TATGATGGTTAGTGATATTAAGTTTAGAAGCTTTAAAGATAGTGATGGTAAAGGTAATATTAGTAAATTG
TATGTAATTTGTTTTATTTTGATTATTCTTATGTTATTTACGATGCCTGATAAATTACTTTATTTGATTA
TGATAGGTTATGCCTTATCAGGTCCGATATCTCATTACAGATATAAAGCTAAAATTAAAAATAGTGATCT
AAATGAAGAATCATCGGTTGATGGTAAGAAAATAATAGATATTGAGAAGTAG

>lcl|NC_006570.2_cds_YP_169842.1_730 [gene=tolQ] [protein=TolQ protein]
[protein_id=YP_169842.1] [location=852360..853079]
TTGGGAATATTTATGGATAATTCTATTTCTTTAGTTGAGCTAGTGCTGCATGCAAATTTTATAGTTCAAC
TTATTATGCTGGCTTTGGTTGCAATGTCTGTATATTCTTGGGCAATAATGCTTGAAGTTAATAACCGTGT
TAAAAAATATCGTAATGAACAGGTTCAGTTTGATAAGCTATTTTGGGCAGGTCATCATATCCAGAAGTTA
TATGATTATTATCTACAACATAAAGAAAATATCTTTGGCAAATCAATAATATTTTGTTCTGGTCTAAGAG
AGTTTAATAACCTTAAGGATACTTGTATGTTAAGGGGAGACACTATTCTAGAGGGTATGGAAAGAACAGT
TAGTATAGCGATAGCACAAGAAGCTAAAGAGCTAGATAGAAAACTACCGGCACTAGCAACAATAGGAGCT
GTGGCACCTTATATTGGTCTAGTTGGAACTGTATGGGGAATTATGTCATCGTTTAATACTCTAGGTGGTG
TTGAGCAAGCTACAATCTCTGTGGTCGCGCCACATATTGCTGAAGCTTTAATAGCTACTGCATTAGGTCT
TTTTGTTGCTATTCCTGCAGTTATTGGTCACAGCAAATTATCTAACCAAGTGGATGATATTTTATCAAGC
TATGAGTCATTCCAAGATGATTTATGTATTTTACTTCTAAAAGAAGCACATAGAGATGAAATTCAACATC
AAAATCAAGAAAGCTTGTAA

>lcl|NC_006570.2_cds_YP_169843.1_731 [gene=tolR] [protein=TolR protein]
[protein_id=YP_169843.1] [location=853089..853535]
TTGCTTATGAAAAAAAGAAATAAAAGATTTTTTAGAAAACAGCGACCTATGGTACAAATCAATGTAGTGC
CTTACATTGACGTTATGCTTGTTCTACTAGTAATTTTTATGATCACAACACCGATCTTAACTCAGGGTGT
AAAAGTTGATCTACCGAAAGCACAATCTGAAAAGATACCTTCAAATGATAGTAAACCAATAGTTGTTACG
GTAAATAAGCAAGGTGAGTATTTTATTAATCAAGGAGTTAATGATCCTAAGACAGCTTTAAGTTCAGGTG
CATTAGCAAATGCTGTGGTGAGTTTATCACAGCAAAATCCTGGTAAGCCTGTTTATGTAAGGGGTGATAG
TAGCGCAAGTTATGGTGAAGTTGTAAAAGCTATGGCTCTTATTCAAAAAGCAGGCATTGATAAAGTGGGG
TTAGTCACAGAAGATGGCAAATCTTAA

>lcl|NC_006570.2_cds_YP_169844.1_732 [gene=tolA] [protein=hypothetical protein]
[protein_id=YP_169844.1] [location=853522..854433]
ATGGCAAATCTTAATTATCATAAATTTTTACGCTTTTGTAATAAGCAAATAGATGAGAACCCATTTTTAG
TTAAGGCTATATTAATCCATATTGCTTTGATAATTCTGCTATATATTTTGTCTTTTGTTAGCAGTCTAAA
GTTTGAAAAAACACAGGCATCTTTGACTGCACAAGTTTCAAATATGCCAAAGAAATTTGAGATTATCCAA
GCCACTTCAATAAGTAGTAGCGAATTAAATAAACAAATATCTGCTTACGAAAATCATCAACAAGAGTTAA
AACAGGCTAAAGAAGATATTAAACAAGCTAAGTTACAAGCTCTTAGAAAACATCAGCAACAATTAAAAGA
GAAGGCTGAGGCAGAAAAAAAAGCTAAACAACAAGCTATCTTAGAGGCTAAGAAAAAAGCTCAGCAAGAA
GCCCAACGTCAAGCTGAGCAAGAAAAGCAAGCAAAACTTGAAGCAGAACGCAAGGCAAAAGCAGAGGCGG
AGCAAAAAGCGCAACAAGAGTTACAACGCAAAAAAGAGCAAGAGCTTAAGGCAAAGCAACAAGCTGAAGA
AAAAGCTCGCCAAGAGAAACTGGCAAAAGCTAGAGCAGAGGCAGAAGCAGCCGCTAGGAAACAAATTGAG
CAGAATCAGGCGCAATCAGCTATTTCTAGCTACATCGCTGCATATCAAGATAGGGTTGGAGCTAACTGGA
TTAAAGATTCTTGTAGAGGTATATATGATTTACCTCGTGCAATTATTAGAGATGGTAAATTTATTAAACT
TACTGGTACATCTGGTAATTATAGATGTGATCAGTCTTTAATTGATGCTATTAAAAATACTACGCCACCT
ACAATTACAAATAATGTGGCAAGAAAAACTATACAAACAGAAAATATAAGCTTTATATTTAAACAAAGTT
AA

>lcl|NC_006570.2_cds_YP_169845.1_733 [gene=tolB] [protein=TolB protein precursor]
[protein_id=YP_169845.1] [location=854433..855752]
ATGAGGAATGGTATGAGGAAAATCATTGCAGGCGTTTTTATATTTGTTTTTTTGATCTCTAATCTATATG
CAGACTTGGTTGCAGAGGTGACTACTGGAGTAATTCAAAAGCCGTTAGTTACGGTTGTTAGTGATAATGT
TGTTGATCAGTTTCCGCAGCAGGTAAATTCTGTAATAGTTGCAGACTTAAACCATAATGCTAAATTACAA
GCTAATGATACAATTAAATATGAAATTAAGCAAAAGCAAAATATTCCTTGGAAGAGCCTAAAATCTGACT
ATGTTGTATTAACAAAATATACGAATAACTCATATAATAATTATACTGTAGAAGTGCAGATTCTAAAGAG
AAATGACACAAGTTATTTACAAGCGATTACTTATAAAAATATAAATGTTTCTCTAATGAGAACATTGGCA
CATAAGATTTCTAATTATGTTTATCAAAAGCTTACGGGCAATCAGGGCTTCTTTTTAACTAAGCTTGCAT
ATGTCAAAGTAAGTAATCCATATGCAAGATATGGTAGGTTATATGAGCTTATTATATCTGACTATGATGG
TTATAATAAGCATGTTGTACTAAGACAAACTGATAACCCAATAGCAACGCCTTCATGGTCAAATGATGGT
CGTTATATTGTTTATTCTAGTTATAGTGGTGGTAGTATGGGAGTATATACTTTAGAAATAGCTACTGGTA
AGGTAACACGTATAACTAACTATAAAGGTATAAATAGCTCACCTTCGTTCTCACCAGATGGTAAAGAAAT
TGCCTTAGCGCTATCAAAAGGCTATTCTGATCAGACTAATATCTACATCATGAACTTATCTACAAAAGCA
CTAAAGAGAATCACTATAAATGGTATAAATACAGCACCAAAGTTTTCTCCAAATGGTCAAAGCATAGTGT
TTACATCTGATAGAGAAGGCAGACCAAATATTTATGTAGCATCAGTAAACTCTAAATATCCACAATCATC

AATATTAAGTACAAAAATACATCAGGCATATGAGCCAAACTATACTCCAGATGGCAAAAATATAGTATTT
ATGAATCAAAGCTCACGCACAAGCGGTACGCAAATTGCTGATTTTAATTTAGCTAATGGAAGTGTAACAA
ATATTACAAATGGTAAAGCAGACAGTTCACCTACAGTTTCGCCATATGGTGATATGGTTGCTTATATATC
AACCAACACTAGAGGTTATAGCTCATTAGATATGGTATCATTAGATGGTGATAATCATTTCAATATTGAG
ACTGCTGATAATGGTAATATTTTAATACAATCACCAAGTTGGTCGCCAAAAAACTTTTAA

>lcl|NC_006570.2_cds_YP_169847.1_735 [gene=FTT_0842] [protein=peptidoglycan-associated lipoprotein] [protein_id=YP_169847.1] [location=856026..856649]
ATGATGAAAAAAAGGATAATCAACTTAGCAGTTATAGGTTCTATGCTTATGTTGGCAAGTTGCTCTAGTA
CTAGACCAGATAATAGTGATTTAATCAAAGATAAATATGCTGGAGTAGATAGCTCACAAGCTTTAGAGAT
GTCTTCGCAGATTTATGGTTCTGATAAGCTAAGCTCAGATCAAGTTGAGCAAATGAAAAAAGAACTTATG
AATATTAATTGTAGATCAGTATATTTTGGTTTTGATAGCTATAATATTACTGATGACGCTAAAGAATGCC
TTGATAAAACGGCAGACTATTTAATAGCTCATCCAGATCAACCAATTAAATTATCTGGTAATACTGACCC
AAGAGGTAGTGAGAAGTATAACTTTAACCTTGGTCAAAAACGTGCTGAAGCTGTGTATAATTACCTACTA
GGCAAGAATGTTAATAAAGATCAAATCTGTGTAGTAAGCTATGGTAAGCTAAAACCAGCTGCAGAGCCAA
CGCAGTTCTATGATGAGTTCTGTAAAGATGGTGTAAATGATGCATGTATGTACAAGGCATCAGAGAAAGC
TTTCTACTTAGACAGAAGAACAGAGATTGATTTTGGTGCTAAATGTGATGAGAGCAATTCATAG

>lcl|NC_006570.2_cds_YP_169858.1_746 [gene=ubiA] [protein=4-hydroxybenzoate octaprenyltransferase] [protein_id=YP_169858.1] [location=complement(868531..869385)]
ATGAATAAACAACAACTAAAAGCATATTTCATGCTAATGCGACTACATCGCCCTATTCCGATATTACTAA
TCTTGTGGCCAACACTTACAGCATTAGTCTTAGCAAGTCATGGACTACCAGATATTAGCTATCTAGTTAT
TTTCACTATCGGTGTAGTAGTAATGCGTACTGTAGGTTGTATTATCAATGATATTGCTGATGTCGATTTT
GACAAACATGTAGCTCGCACAAATACTCGACCTTTAACAAGTGGACAATTAAGTATTAAAAATGCTATTT
GGCTTTGTATTTCTCTTACATTAGTTGCATTTATATGTGTATTATTTTTGAACTTATACACGATTTTATT
ATCATTTGTAGCACTATTCTTAGCAATACTATATCCGTTTTGTAAACGATTTTTTGCGATACCTCAACTA
ATACTCGGATTAGCATTTAATTTTGGTATTTTTATGGCTTTTTCTACAATACAAAACCAAATACCAGTCG
AAGCGTGGATATTTTATATAGCTACGATATGCTGGACAATAGCTTATGATACTATCTATGCACTAGCTGA
CCGAGAATTTGATTTAGAGATTGGTATAAAGTCATCTGCTGTTTTATTTGGTAATAAAGTATTTAGATAT
ATCTTATTATTTAATTTCTTATCTTTACTTCTTTTAATAATCCTTGGTATTTATTGTGATTTTAATAGTT
TTTTTATTTAGGTGTTGTTATTTGTAGTTTATTTTTCGTTAGAAATTATTTTTTATATAAAAAATTAGG
CATCACTAACTGTATAAATGCTTTTTCTGCTAATCATTGGATTGGCTTAATAATTTTTATTATAGCAGTG
ATACAATATATATAA

>lcl|NC_006570.2_cds_YP_169861.1_749 [gene=FTT_0859c] [protein=hypothetical protein] [protein_id=YP_169861.1] [location=complement(870754..871077)]
ATGTCAGAACAACAACCACGTAAAAAACTATCATTAGGCGATAGAAAAAAACCATCTACAACTAGAGTAT
CTAGTCTAAGTGTTAACCTTAAAACTAAACTACAAGCAAAAACAGAAGATAATATACAAAACTTCTTTAA
AAATACGAATAAAGAACTTCGGGAGCTATATCAACAAAGAAAAACTCTCGAAGAGCAAATCAAACAAATA
GAGCAAAGACTATTGTTTGCCAAGAGTGAACCATTGCAAGATTTTTTATTTGAATTAAAATCAAAAGATT
TTGAACTACTTGTAAAAATTAATTCTTTTATAAATAACCTGTAG

>lcl|NC_006570.2_cds_YP_169876.1_764 [gene=aroC] [protein=chorismate synthase] [protein_id=YP_169876.1] [location=complement(884781..885839)]
ATGTCAGGAAATACTTTTGGTAAAATTTTTACAGTAACCACTTGTGGCGAGAGTCATGGCGATTCACTTG
CAGCTATTATTGATGGCTGTCCTAGCAATATACCTCTTTGTGAGGCAGATATTCAATTAGAACTAGATAG
ACGCAAACCAGGCCAATCAAAATTCACCACTCAGCGTAAAGAGCCTGATGAAGTTAAGATAATTTCTGGT
GTTTTTGAGGGTAAAACTACAGGTACGCCTATCGGTCTGATAATAAAAAACCAAGATCAAAAATCAAAAG
ACTATAGTGAAATAAAAGACAAATTTCGTCCTGGTCATGCTGATTATACCTACTTCAAAAAATACGGTAT
TCGTGACTATCGTGGTGGTGGTAGATCATCAGCAAGAGAAACAGCAATGCGTGTGGCAGCTGGAGCAATA
GCTAAGAAAATACTCAAACATTACGGCATCGAGATCTATGGCTTCTGCTCACAGATCGGTAGTCTTAAGA
TAGATTTTATTGATAAAGATTTTATAAATCAAAATCCTTTTTTTATTGCCAATAAAAATGCAGTTCCAGC
TTGCGAGGATTTAATTCACAGCATTCGCAAGCAAGGTGACTCTATTGGTGCTGAGGTAACTGTAGTAGCA
ACAGGTCTAGAGGCAGGATTAGGTAGACCAGTTTTTGATCGACTTGATGCTAGTATTGCTTATGCAATGA
TGAGTATAAATGCCGTCAAAGCAGTCAGTATTGGCGATGGCTTTGACTGTGTTGCACAGAAAGGTAGTCA
ACATCGTGATGAAATTACCCAACAGCAAGGCTTTTTATCAAACCATGCTGGAGGAATTTTAGGTGGTATA
TCAACAGGTCAAGATATAATTGCTAAATTAGCTTTCAAACCAACATCAAGTATCCTACAACCAGGTAAGA
GTATTGATGTACAAGGTAACGATACTACTGTTATCACCAAAGGTAGACATGATCCTTGTGTTGGCATCAG
AGGTGTACCAATAGCTGAGGCGATGTTAGCACTAGTTTTAGTTGATGAACTCCTAATTACGCGATCTTAT
AGGGATTAG

>lcl|NC_006570.2_cds_YP_169889.1_777 [gene=folD] [protein=bifunctional 5,10-methylene-tetrahydrofolate dehydrogenase/5,10-methylene-tetrahydrofolate cyclohydrolase] [protein_id=YP_169889.1] [location=900247..901095]
ATGATTTTAATCGATGGTAAGTCTCTCTCAAAAGACCTTAAAGAGAGATTAGCAACTCAAGTTCAAGAAT
ATAAGCATCATACAGCAATCACCCCAAAACTTGTCGCAATAATTGTTGGTAATGATCCGGCAAGTAAGAC
ATATGTTGCCTCAAAAGAAAAAGCCTGTGCCCAAGTTGGTATAGATTCTCAAGTGATTACACTACCAGAG
CATACTACAGAATCAGAACTTTTGGAGCTGATCGATCAGCTAAATAATGACTCTAGTGTTCATGCGATTT
TAGTGCAGCTACCATTACCAGCACATATCAACAAAAACAATGTAATATATAGTATAAAACCAGAAAAAGA
TGTCGATGGTTTTCATCCTACAAATGTTGGTAGACTACAACTTAGAGATAAAAAATGCTTAGAATCTTGT
ACACCTAAAGGTATCATGACTATGCTAAGAGAGTATGGTATAAAAACAGAGGGGGCCTATGCAGTGGTTG
TCGGCGCAAGTAATGTTGTTGGTAAACCAGTATCACAATTGCTGCTAAATGCTAAAGCTACAGTGACGAC
TTGTCATAGATTCACAACTGATCTTAAATCACATACTACAAAAGCAGATATCCTAATTGTTGCTGTTGGT
AAGCCTAACTTTATCACAGCTGATATGGTCAAAGAGGGTGCTGTAGTTATTGATGTTGGTATTAATCATG
TTGATGGTAAGATAGTTGGTGATGTTGATTTTGCTGCTGTAAAAGATAAGGTCGCAGCAATTACCCCAGT
ACCTGGAGGGGTTGGACCAATGACAATTACTGAACTGTTATATAATACTTTTCAGTGTGCTCAGGAGTTG
AATAGGTAG

>lcl|NC_006570.2_cds_YP_169894.1_782 [gene=purK] [protein=5-(carboxyamino)imidazole ribonucleotide synthase] [protein_id=YP_169894.1] [location=905875..906972]
ATGAAAATAGGTATTATCGGAGCTGGACAACTTGCCAGGATGCTAAGCTTAGCAGGTACACCTTTAGGTT
TAGAGTTTCATTGTCTTGGTAAAAATGGTGATTGTGCTGAAGAAGTTGTAAAAACTGTTACAGATATTGA
GCTAACAAAGGTAAATGATGTTGTAGCTTGGGCAAAGCAGTTTGATGTGATAACTTTTGAAAATGAAAAT
ATCTCTCATGAGCTTATCAAAGCTATAAATCATGAGGTGAGTGTGTATCCATCAGCTAAAGCTATAGCTA
TTTCTCAAGATCGTTTACTTGAGAAATCCTTTATGCAAGATCATGGTATTGCTACTGCAAAATTTGTAAA
TATCGATAGTTTGGCAAAATTGCAAAGTGCTGTTGATGATCATGGTCTGCCAGCTATACTAAAAACTCGT
CGATTTGGCTATGATGGTAAGGGCCAGTTTGTGATTAGATCACAAGAGGATATTACTAAAGCATGGGATG
TGCTCAAGGATGCTCCGGATGGGCTTATTTATGAGGCTTTTGTTGATTTTGATTATGAAGTTTCACAAAT
ATGTACAGCTGATCTAAAGGGTAATATCGCCTTTTATCCATTGGCTAGAAATACTCATAAGCAGGGTATT
ATCGTTGAATCAGAAGCACCTTTTGAAAATGTTGTACTTGCTGAAAAAGCACAACAAATCGCTAAAATTC
TAGTCAAGGAGTTTGCATATGTTGGTACTTTGGCAATTGAGTTTTTTGTCAAGGGTGATGAGCTAATAGT
CAATGAAATAGCGCCACGTGTGCATAATAGCGGTCATTGGAGTATTGATGGCGCCGTCACATCACAATTT
GAAAATCATGTGCGTGCTATTGCCGGATTGATTTTAGGTGATACAACTAGTCGTAAAACAGTTATGCTAA
ATTGTATCGGTGGTATGCCGGCAACAAAAGATTTAGCAGCTCTAGATAGAGTTAAAATTCATAGTTATAA
CAAGGAGCCTCGTAAGGGACGTAAAGTAGGTCATTTAAACCTTAATTTAAATGATGAAACTGATGAGTAT
CAGCTACTTCAAGTTAAAAAATTAATTGCTCTTTCTGAAGAAATTTAG

>lcl|NC_006570.2_cds_YP_169897.1_785 [gene=FTT_0900] [protein=hypothetical protein] [protein_id=YP_169897.1] [location=909502..909873]
ATGATGACTTTACAAGAAGAAAAAATACAAGCGCCAGTATTTTTTAAAGAATATGTCAAAGGTAGGTTTA
TACTTAATATAGGTGAGTATAATCATCCATTGATTTTATCAGCAACTCAAGTGCTTGAGTATCAAGATAA
AATTGATGATATCCAAAGTATCAAAAAAAGTCATCTTGATCTTATCTTAGCGACTAATCCTGAAATAATA
CTCATAGGTACCGGTGAAAAACAACTTTTGCCTCCGCTTGAGATAATTAATCAAATCGCAAAAGCTGGTA
AAAGTGTTGATTTTATGGCGAGTGATACTGCTTGTAAGACATATAATTTGCTTGTTAATGAAAATCGTAA
TGTTAGCTGTATCATCATTTAA

>lcl|NC_006570.2_cds_YP_169903.1_791 [gene=topA] [protein=DNA topoisomerase I] [protein_id=YP_169903.1] [location=complement(913713..916007)]
ATGGCAAAAAAATTTAGTAATCGTAGAGTCTCCAGCCAAAACTAAGACTATAAAAAAATACCTTGGTAACG
AATTTGAAATATTAGCATCATTTGGACACGTACGCGAAATACCTTCCAAAGATACTTCTATCGATGTTAA
TGATAATTTTAAGATTAAGTTCACAACTAGTGAAAAAAGCAAAAAACATTTAGATGCAATCAAAAAAGCA
GCTAAAGATGCTCAAAATATCTATCTCGCTACTGACCCAGATAGAGAAGGTGAAGCTATATCATGGCATG
TACAAGAAGTATTAAAAAGTGCACGCTTACTTAAAGATAAAAATATCTATCGTGTCACATTTAACGAAAT
CACAAAATCAGCAGTTACAAATGCTATAGCAAATCCAAAAGAGTTATCTATGGATTTAGTTGATGCTCAA
AAAGCACGCCAAGCACTAGATTTTCTGGTTGGCTTTAATATATCTCCGCTATTATGGCGTAAAATCACCA
GTGGCTTATCAGCAGGAAGAGTACAAAGTCCTGCTCTAAGAATGATTGTCGAGCGTGAAATAGAGCGTGA
AAACTTCATCAAACAAGATTATTGGAGTCTAACTGCAGATACTTTCAAACAAAAACAAATATTTGCAAAT
TTGCTTGAATTTAATAATAATAAAGTTGAACAATTCACTTTCACAACAGCAGAAGATGCTGAAAATGCAA
AAGCCCATATCCTAAAAGATGCTAATGGTTTTTTGATAGTTGATGGCATTACTGAGAAAAAAACACGGAG
AAACCCATATCCTCCATTTATAACATCAACACTACAGCAAGAGGCTTCAAAAAAACTCGGCTTTACCGCT
AAAAGAACAATGTCTACTGCACAAAAATTATATGAAGGTATTGATCTAGGCAATGGTGAATCCGTTGGTC
TTATCTCGTACATGAGAACAGATTCAACCAACCTTTCTAACGATGCTCTTAATGATATTAGAAACTTTAT
TCAAGCAAAATATGATAAGGATATGCTACCAGCTAAGCCACGAGTTTTTGAGAAAAAATCACAGAATGCG
CAAGAAGCACACGAAGCTATCCGTGTCACAGCTGCGAATAGAACTCCAGAATCAATAAAGCAGTTTTTAA
CTAATGATGAATTTAAACTCTATAGCCTAATCTACAATCGAACTATTGCTTGTCAAATGAAACATGCAAC

TTTAAATAGTACATCAGTAGATTTAATTACCGAAAATACTAAACATAGGTTTAGAGTTACAGGAACTGTT
ATAGTAGATGCCGGATTCTTGAAAATTTATAATGTTGAGAAAGATGAGGATGAAAAAGACTCAGATGATG
AGCAAACTTTACCAAAATTTGAAAAAGGTGAAAAAATAATACTAAATGATGTAATTACTAAAGCTCACTC
AACAGAGCCACCACCTCGCTATACCGAAGCATCTTTGGTTAAAGCATTAGAAAAATATGGTATTGGTAGA
CCTTCAACCTATGCAACAATCATCTCAACTTTACAGCAACGTGAGTATGTTGAGGTAGACAAACGTCGTT
TTATCCCTACAGATAAAGGTCGTATCGTAAATAAATTTCTAACTGAATATTTCAAAAAATATGTTGAGTA
CTCTTATACCGCAGGCTTAGAAAAAGAGCTTGATGAAATTGCTCATCACAAAAATGATTACCTAAGTGTA
TTAAATAATTTCTGGCATCCATTTATTGAGAGAATCAATAAGATTTCTGAAGATGTCTCACGCAAAGATG
TTGTGCAAGAAGAGCTAGAGGAAGACTGTCCTGAATGCGGTAGTAAATTATCACTTCGACTTGGTAAAAA
TGGTAGATTTATTGGCTGTACAAACTACCCAACTTGTAAATACACCCGTCCAGTAGATAGTGATCCTAAA
GAAGTCATCAAAGAAGAACCTATAGTCGTAGAAGATAGAAAATGCCCTAAGTGCAATTCTGATTTGCATA
TCAAACAAGGTCGCTATGGTAAGTTTATTGGTTGCTCAAATTATCCTGAATGCAAACATATGGAACCTCT
TGAAAAACCCAAAGATACTGGTGTAGTTTGTCCTAAATGTAATAAAAATCATATTGTTGAGAAAAAATCT
CGCAAAGGTAAAGTTTTCTATGCTTGTGCTGGCTTCCCTAAGTGTAAAAATGCTTATTGGTATCCACCAA
TAAAAGAAGAGTGTCCTAAATGCCACTCACCAATACTGTTACACAAGATAACCAAAAAAGATGGCGAACA
GAAAGCTTGCCCTAATAGCGAGTGTGACTATGCTGTAGCATTTGAAACTAAATAA

>lcl|NC_006570.2_cds_YP_169910.1_798 [gene=FTT_0913] [protein=hypothetical protein] [protein_id=YP_169910.1] [location=920784..921251]
ATGTCCAAGAAAATTTTTACCCTTTGCTTGGCGAGTTTGGTGCTTGCATCATGTACTTTCAAAAGTGATA
TAGAAGATAATATTACCCATTATGAGAAATTGACAAGATATAGCATGATTAAACAACTTGATTTTAGCAA
ACACACAAATGATATTGATGGTGCGAGATTTTTAGTGGCAAATTATCCGGATCAGGTTATTCGTCTTAAT
GGTACTGATTTTGATAATAAGTATGATAAGTTAGTTAGCTATTTAAAAACTAATGGCTATACAATTATGG
TGAATGAGAAATTACCTTTGACTGCTACAATTATCGCTGAAATTAATAAGCCTCTTCCTGATGTAAAATA
TATTGGGATTATTCTGCAACAGGATTCGAGGATGGATCAGACAATCTATAATATATCTTATGGTGGTAAT
GAATTTAAAGCACGTCAATTTTATACTGATTACAAAAAAACATTATGA

>lcl|NC_006570.2_cds_YP_169911.1_799 [gene=lspA] [protein=lipoprotein signal peptidase] [protein_id=YP_169911.1] [location=complement(921246..921731)]
GTGAATTTGCTTAGACCAAAATTGAAATATTTTATTTTAGCAATTTTAATTATTGCCGCTGACTTATACA
CTAAGTATTTAGCAAATACATACCTAGAATTTGCTCAATCTCTTAAGATAACTAGTTTCTTTAATCTTAC
GTTGTTATACAATCATGGCGCAGCATTTAGTCTTCTAAGCAATGATCAAACATCATGGCAAATGATAATG
TTTTCTACAATCTCTCTAATTGCTGCTATTGTACTTATTTATCTAATTATTAAACAACCAATTACTGAGA
AAATTAATTTGTTCTCTTTTGCTCTTATTCTAGGTGGTGCTTTAGGCAACTTTTACGATCGTGCTTTTCA
AGGATATGTAATCGATTTTTTAGATTTTCACATAGGTAATTATCACTGGCCGTCATTTAATATAGCTGAT
TCAGCTATCACATGTGGTGTTGTAATTTTGATAGCAGCTTCACTATTTACTAAAAAGAAATCATAA

>lcl|NC_006570.2_cds_YP_169912.1_800 [gene=ileS] [protein=isoleucyl-tRNA synthetase] [protein_id=YP_169912.1] [location=complement(921718..924525)]
ATGAGTGACTATAAAGACACCTTAAATCTACCAAAAACATCTTTTTCTATGAAAGGTAACCTAGCTAATA
AAGAACCAATGATCCTTAACAAATGGGAAAAGCAAGGTATTTATAAGAAAATTAGAGAACACTTTGCTGG
ACGCGAGAAATTTGTTCTTCATGATGGTCCTCCATATGCTAATGGTAGTATTCATGTTGGTCATGCAGTT
AATAAAATTCTCAAAGACATAATCATCAAATCAAAGACATTAAGTGGTTATGATGCTCCTTTCACTCCTA
CTTGGGATTGTCACGGTTTGCCAATTGAGTTACAAGTCGAAAAAAAACATGGTAAAGCTGGTCAAAGTAT
ATCAGAAGATGATTTTAGGAAAGAATGTCGTAAATATGCTAAAAAACAGGTAGAAATCCAAAAGAAAGAT
TTTAAAAGACTTGGTGTTCTAGGTGACTGGGAACAACCATACCTGACAATTAATTTTGATTATGAAGCTA
ATATGATTAGAACATTAGCTAAAATTATCGAGAATGGTCATCTAAGCAAAGGATTTAAACCAGTTCATTG
GTGTACTGATTGTGGTTCGGCACTTGCTGAAGCTGAGGTTGAATATGCTGATAAAGTTTCTCCTGCTATC
GATGTTAAATTTAAAATTAAAGACAAAGATAAACTTGCTCAAGCATTTGGCTTAGATTCACTAAATCACG
ATGCTTTTGCAATTATTTGGACAACTACTCCTTGGACACTTCCTGCTAACCAAGCAATTGCAGTTAATAA
CCAACTAAATTATAGCTTAATCAAAATTGAAGATTTTTATATTATCTTGGCTGAAAATCTAGTTGAACAA
ACACTTAAAAGATACGCAATAGAAAATGCTCAAATAATTGCTACAACAACAGGTAACAAACTAACTGGAA
TCATGGCTGAGCACCCTTTTTACAGTCGTCATGTACCAATTTTACATGGCGATCATGTCACTGATGATTC
TGGTACAGGTTTGGTACACACAGCTCCTACTCATGGTGTTGATGATTTTACCCTAGGAAAAGAGCATAAT
TTATCTATGGAAATTTTTGTCAAAGGTAATGGTTGCTACAGCGAGAATACCAAACTATTTGCCGGAGAGT
TTATATTTAAAGCTAATGATAGAATTATTGAGTTACTTGGCGAGAAAAAACGCCTAATGAACTCTGATAA
AATTAAGCATAGCTATCCTCACTGTTGGCGTCATAAAACTCCACTGATGTTTAGAGCAACACCACAATGG
TTCATTAGCATGGAAAAACAAGGACTGCGCGATAAAGCTCTACAAGCAATCAAAGAAACTAGCTGGGCAC
CTAGCTGGGGACAAGCACGTATCGAAGGCATGGTCAAAGATAGACCTGATTGGTGTATATCACGCCAAAG
AACTTGGGGAGTTCCTCTGCCTTTATTCATCCATAAAGAAACTGAAGAGCTACATCCAAATACTATTGAA
ATACTACACAAAGTTGCTGAGAAAATTGAAAAAGACGGTATTGAAGCATGGTTTAATGCTGATGACTGTG
AATTCATCACAGAAACTGCTCAATACAAGTCAGTCAAAGATACCTTAGATGTTTGGTTTGATTCTGGCTC
ATCTAGTATGTGTATATTAGACTTAGATAAACGCCTAAGCTATCCAGCTGACTTATATTTAGAAGGATCT
GACCAACATCGTGGTTGGTTCCAAACATCGCTTTTAGTAGCAATGTCTGCTAAAGGTAGTCAACCATATA

AAGAAGTTTTCACACATGGCTTTGTAGTAGATGAGCATGGTCGCAAGATGTCAAAATCTTTAGGCAATGT
AACATCTCCTCAAGACATTTATAATACTCTTGGGGCTGATATCTTACGCCTGTGGACAGCCTCAACTGAT
TATAAAAGTGAAATGGCTGTGTCTGATCAAATACTAAAAAGAACTGCTGATACTTATCGTAGATTACGTA
ATACTGCTAGATTCTTGCTTTCAAACTTAGATGGATTTAATCCTGTGACTGATATTATCGAATTTGATAA
ACTAGTTAAGCTAGATCAATGGGCGATTGCAAAGACTAAAGAGTTCCAGGATAAGATTATTGAAGTTTAT
GATAAATATCAAACCCACACAGTCGCACAATTAATACATCATTTCTGCTCTATTGAGATGGGAAGTTTTT
ATCTAGATATCATCAAAGATAGACAATATACTGCAAAAACTGATGGACACCCACGCAAATCTGCGCAAAC
AGCTATTTATCATATAGTTCATGCTCTTGTAAGATGGATGGCGCCTATACTTTCATTTACTGCTGATGAA
ATTTGGGATGCTACACCAAAAACTACTGATCTGCCAATCCAACTTTGTGAATGGTATACAGGGCTTAAAT
CTTTTGATCAAGATGCTGAACTAGATCTAGAATACTGGGCAAAAATCCAAGAAATTCGCTCTGAAGTAAA
TAGAGTTCTTGAAATTAAAAGAAATGAAGATGTTATCAAAGCCTCCCTAGAAGCAGAAATTACAATTTAT
GCTGATAAATATAATTATAATCTATTAGAAAAACTTGGTAATGAGCTTAGATTCCTACTAATATCATCAA
AAGCAGACCTAAAAGTAATTGAAGAAAGCACAAGTAGCTCAATCGCTGCCAACATACCAGGTTTATTAAT
TGAAATAACCAAGATAGAAGAGCCTAAATGTGAAAGATGTTGGCACCGTAGCTCGACTGTTGGGGATAAT
CCTCAATACAAAGATATTTGTAGTCGCTGTGTTGAGAATATAACTACAGAAGCTGGAGAGTCGCGTGAAT
TTGCTTAG

>lcl|NC_006570.2_cds_YP_169913.1_801 [gene=ribF] [protein=riboflavin biosynthesis protein RibF] [protein_id=YP_169913.1] [location=complement(924618..925538)]
ATGAAAATTATTACAAACCTAAATAAAACCAAAGATTTTCTACCTAAAGCAATAGCTATTGGTTCTTTTG
ATGGTGTACACCTTGGGCACCAAGCAATAATAAAAAAACTCCTAACTATAGCTAAAGAGAACAACCTTGT
ACCGTATATCTTATTCTTTGAACCTTTACCTAAAGAATTTTTTCTCAAAGATAAAGCTCCATTTAGAATA
TATGATTTTAGAAACAAAGTAATCAACATACATAAACTTGGTATCAAACATATAATTTGTCAAAAATTTA
ACACAAAATTTGCAAATATCACTGCAAATGAATTTATCGAAGAGTTTCTTGTCAAGAAACTAAACACCAA
ACATATAATCGTTGGTGATGACTTTAAATTTGGCAAAAACCGTGGTGGCGATTATGCGCTTTTAAATCAA
TACTCACAGACTCATGATTTTAACGTAGATAAAGTTTCTACTTTAAATCTAGATAACCACCGTATCAGTA
GTAGCGATATTCGCCAAGCTCTTACAAACCATGATTTAATAGAAGCAAATAAACTTTTAGGTGAATCACT
AAAGATTAACTCGCGTGTCATACATGGTCAGAAAAAATGGTAGAAAAATTGGCTTTAATACTGCTAACCAG
AAATTACCTAAGAATTCTGCTCTAAAGGGGGTTTATCTGACTAGAGTTTTTATCGATGATGTGATTTTCT
ATGGAGTAGCTAATGCCGGTACCAGACCAACTATCGATGGTAAAAACAACCTACTTGAGACTCATATCTT
TAACTTTAATCAAGAAATTTATGGTAAACATATAACTACAGAGATAGTAAGTTTTATTAGAATAGAAATG
AAATTTAACTCTTTTGAAGAATTAAAATTGCAAATATCCAAAGATATTCAAACTGCAAAAAAACTAATAA
GTACATTATAG

>lcl|NC_006570.2_cds_YP_169914.1_802 [gene=maeA] [protein=malate dehydrogenase] [protein_id=YP_169914.1] [location=925689..927503]
ATGGAAAGACGTGAAAAAAGAATAAGAAAATTAAGAAATAAAAGCGGTAAAGTTTTTGCAATAGAAACTA
ACCTTACAGGTAGACAACTATTAAACAATAGGGTTTTGAATAAGGATGTTGCTTTTAGCCAAGAAGAAAG
AGTTGCTTTTGATCTTATCGGCTACCTACCAGAAAAAGTTGAAAGTCTCGAAGAGCAAGCTATCAGAGTA
AGAAGACAGCTTGATTTGAAACCTAATTCTCTTGAAAAATATGTATTTTTAAATAGGCTTCATGACTTAA
ACACCACGCTATTTTATCACTTTGTGCGTGAGAATTTAGAAGAAATAATGCCAATTATATATACACCAAC
TGTGGGCGAAGCTGTCCAAAAATATAGTAGCTCATTTAGAAAGCAAAGTGGTTTATTTATCTCTATAAGT
CATAAAAAGCATATTGCTAGAATACTAGAGCGATATGAGTATAACAGTATTGACTTAGTGCTTGTTACCG
ATGGTGAAGCAGTGCTTGGAATTGGTGACCAAGGGATTGGTGGTATGAATATCAGTATCGGTAAGATTAT
GGTTTATGTTGCTGCAAGTGGTATAGATCCAGCTAGAGTTTTGCCGGTACAGCTTGATATGGGTACAAAT
AATGATGCTTTACTTAATGCTCCTGGTTATCTAGGTGTACGCTTGCCAAGAGTCTCTGGTGAAACCTATG
ATGAGTTTATTGAAGAATTTGTTACCCAGGTTAAAGCTAGATTTCCAAATGTGTTTTTGCATTGGGAAGA
TTTAGGTCGTGATAATGCGACACGTATTTTAGAAAAATACAAAGATAAGTTATGTACATTTAATGATGAT
ATTGAGGGTACGGGCATTGTTGCAACAGCTAACTGTATAGCTGGTGTGAAAACGGCAAGCACTATAAGAA
TAAACGCTGGTGAGATAACTGTTGAAGAGGCATTAAGTGGTATATGTGATATCAAAGTAGTTATTTTTGG
TGCTGGTAGTGCTGGTTGCGGTATCGCTAGACAGCTTGCTGATGTGATCGCTGATCGAGCGGGGGTATCT
ATTGAGAAAGCCAGAAGTAGTATCTATCTAGTAGACAGATATGGTTTAGTTTGTGATAGATTAAGAGCAA
AAAGAATTACACCAGAGCAGAAATTATTTGTCAAAACTAGAGAAGAGATTGATAAATGGAAGGTTGAGGA
TTTTAACTATATTACACTTGAAGAAACAGTTAAAAATACAAAATGTGATATTTTAATTGGGACATCTGGT
CAACCAGGATCATTTACTAAAGAAATTATAAAAACTATGGCAAAAAATAATAACTATCCTATTATTATGC
CACTATCTAATCCTACATCTCTTTGTGAAGCATTACCTGAAGACATTATTAAGTGGACAAATGGTAAGGC
TCTAATTGCTGCAGGTAGTCCATTTCCAGATGTGACATATAATGGTCGTGATTACCGTATTTCTCAGGGG
AATAACGCCTTTATATTCCCAGGTTTAGGTTTAGGCTCAGTAGCTGTGCATGCACGAGTTCTTACAAAAG
GGATGATTAGAGCTGCTAGTTATAGATTAAGTGAGTTATCACCGATGGTAATAAATGAGGATATTACTCA
GCCATTGTTAGCTAGGATAACAGATTTAGTTGATGTTACAAAAGAAATTACTAAAGCAGTAGCTAAACAA
GCAATTCTAGAGGGTGTTCATGGTGTTGATATAGATCTAGAAGATTTAACAGAAACTGAGCTTGATGAAG
AGATTGAGAGACTAATTAACCTATCATCATGGACACCGACGTATGCACCATATATGCCAATTTAA

>lcl|NC_006570.2_cds_YP_169920.1_808 [gene=FTT_0924] [protein=hypothetical protein] [protein_id=YP_169920.1] [location=935772..936170]
ATGGGACGATTTCAACAAAACTTGATGTATGCGGTTATCTTGGTGATAATTTTTGCAGTTTGTGTGTATT
TTTTTACTTCAAATAATGAAGCTGAAACAAAAGGAGTCTATCTGCCTAAATACAGTGCTGAACTACCACC
AACTGATCCTAGTCAAGTTAGAGTTTATAATCTACAATACCAGAGTGATACACAAGGAAATATTGGTCAA
GTCAGAACCTCAACTCATGTCAGTAATGAAAAAGATTTTCAAAAACTTTGTGATAAAAACCTTAAAGAAG
CTATCAAATTAGCAGCTCAACATGGTGCTCATGAGATCAAATATATTTGTCTTTATCCAGAAGGGCAAAT
CAATGAGTTAAGTAGTGTTCAATTACGTGGATACGCTTTTAGAGATTAG

>lcl|NC_006570.2_cds_YP_169921.1_809 [gene=fmt] [protein=methionyl-tRNA formyltransferase] [protein_id=YP_169921.1] [location=936175..937116]
ATGAAAAAACTAAATATAATTTTCGCTGGAACTCCTGATATTTCAGCTCAAGTTCTAAAAGACTTATATA
AATCACAACATAATATCCAAGCTGTACTTACGCAACCAGATAGAGCAAAAGGTCGTGGTAAAAAAGTACA
GTTTTCACCTGTTAAAGAAGTTGCATTAGCAAATCATACTCCAGTATTTCAGCCATTATCTTTTAAAAAG
AATCCTGAAGTACTCGAGCAAATCAAACAACTAAAACCGGATGTAATAGTTGTAATAGCTTATGGAATTA
TTGTTCCACAAGAGTTTTTAGATATACCAAGGTATGGCTGCTTGAATATACATGTATCACTATTGCCTAA
ATGGCGAGGAGCTGCGCCTATTCAAAGAGCTATCCAAGCTGGTGATACAAAAACGGGGGTCTGTATAATG
CAGATGGATGCTGGCCTTGATACTGGAGATATTCTAAATACTTTAGAAATTGAAATACAAGAAACTGATA
CTTCACAAACACTTCACGATAAATTTGCTAAGTTATCAATTAAGCCATTACTAGAAACTTTAGAAAAGAT
TGAAATAATTAAGCCCGAGCCACAACAAGGAGAGCCAACTTATGCACATAAGATTACTAAGCAAGAGGGA
TTAATTGATTTTACAAAATCAGCATGGCAAATTAGTTGTCATATTAGAGCATTTACACCGTGGCCAGGAG
CATATTTTATATTAGATGATGAAGCAATAAAGGTTGGTGAGTTTGAGATACTTTATCAAAATACGGATAA
TAGAAAAGCAGGTACAATTATAGATATTTATAGAAGTGGCTTTGATATTGCGACAAGTGATAAAATTATT
AGATTTAGACAATTACAATTTCCTAATAAAAAAATGCTTAATATTGTAGATATTTTAAATGGAAAAGATT
TAGATAAATATATTGGATACAAACTAGGATAA

>lcl|NC_006570.2_cds_YP_169922.1_810 [gene=gshB] [protein=glutathione synthetase] [protein_id=YP_169922.1] [location=937120..938094]
ATGAAAGTTGGATTTATAATAGATAACTTAAACTCTTTTAATATTTCAAAAGATAGCACTTATATGATGC
TACATGCAGCCCAAGATAAAGGCTGGGAGATTTATACCTTTTATCTTAATGATTTATCAATAATTAATGG
TAAGCCAAAAGGTGATGCACTTAAGATAAAAATTCATAAGACAAAACAAGATTGGTATGAGATTTTATCG
CAACGCCATGATTTTTCATTACTAGATCTAGATTGTATCTTTATGCGTAAAGATCCACCATTTAATATGG
AATATATTTATGTAACTTATATGCTTGATTTAGCTAAAAAAAAATGATGTTTTGGTAGTCAATAATCCCCA
AGCACTTAGAGATTTTAACGAAAAAGTAGCTATTTCAAATTATCCCAAATTTGCGCCGCATACCTTGATC
ACCAGAAGCTATAAACAGATTAATGAGTTTTATGAAAAGCATAAAGATATTATTGTCAAACCTTTAGATG
GTATGGGTGGTAGTTCTATCTTTAGAATTAAAGAAGGTGATAAAAACAAAAACGTAATATTAGAAATACT
TACTCAGCATCAAAGCCGTTATATAATGGTGCAAGATTATCAAAAAGCTATCAAAGAGGGTGATAAGAGG
ATACTTATAGTTAATGGTGAGCCAATTAAGTATCTTTTAGCAAGAGTGCCTAGTGATAGCGATAATCGTG
GCAATCTTGCAGCTGGGGCTACAGCAGAAGTTAGAGAACTCCAAGATAGTGATTATAAGATAGCCAAAAA
GGTCGCTAAGAAGCTCAAAAAAGAAGGCGTGATGTTTGCTGGTATTGATGTGATTGGCGATAAATTAACA
GAAGTTAATATTACTAGTCCAACAGGTATCCAAGAGATATACAAAGCTACAAAAATAAACGCTGCTAGTT
TACTAATGCAAGCAGTTGAGAAAAAAATAAATAAGATGAGACAGGAACACGAAAATGGAGAATAA

>lcl|NC_006570.2_cds_YP_169923.1_811 [gene=hemL] [protein=glutamate-1-semialdehyde aminotransferase] [protein_id=YP_169923.1] [location=938084..939379]
ATGGAGAATAAATCAAACTCTCAAATTTTATTTGCAGAAGCACAGCAATATATACCAGGTGGAGTAAACT
CTCCAGTTAGGGCATTTAAGAGTGTTGGACAAGAATTTCCAAGATTTATAAAGTTTGCAAAAGGCGCTTA
TCTATATGATGTTGATTGGAACAAATACATAGACTATATTGGATCATGGGGACCGATGATTTTAGGTCAT
TGTGATGACGATGTTTTAGAAGCAATACAATGTCAGGTCAAAAATGGACTAAGCTATGGAGCACCATGTA
AGCAAGAGGTTGATCTAGCTAAAAAAATAATTGAGCTAATGCCAAATATTGAGCAAGTAAGATTTGTAAA
CTCAGGTACTGAAGCTACTATGAGTGCAATCAGATTAGCAAGAGCATATACATGTAGAAATAAAATTATT
AAATTTGAAGGCTGTTATCATGGTCATGCTGATGAGTTTCTTGTTGCAGCTGGTTCTGGTGCGTTATCTC
TAGGACAACCAAACTCTCCTGGAGTGCCGGAAGATGTTGTCAAAGATACTTTAGTAGCTAGTTTTAATGA
TATGGAGTCTATTCAAGCACTTTTTGAAAAATATAAAGATGAAATTGCTTGTATTATCATTGAGCCAATT
GCTGGTAATATGAATATGATTTTCCCACAAGATGACTTCTTAGCCAAACTTAGAGCTATTTGTGATCAAA
ATAGTAGTTTATTGATATTTGATGAAGTGATGACTGGTTTTAGAGTTGCTTTGGGTGGTGCGCAAAGTAT
CTATAATGTTAAGCCAGATTTGACAACTTTGGGTAAAGTTATTGGTGGTGGTATGCCAGTTGGGGCTTTT
GGCGGACGTAAAGAGATTATGCAAAAAGTTTCTCCAGCTGGACCAGTTTACCAAGCAGGGACACTATCTG
GAAATCCTATTGCGATGACAGCAGGTATCAAAACTTTAGAAAAAATCTCACAACCAGGGTTCTTTGATGA
GCTTGGAGCTAAAGCACAAAAGCTAGTAGATGGTTTAAATGAGGCTGCTAAAGCCTATGATTTTAATTTT
CATGCAAAATGTCTGGGCGGAATGTTTGGTTTATTTTTCTGTAGTGACAAAATTGCAGTAAATACATTTG
TAGATTTAGGCAAAACAAACCTTAAGATGTTTAATCAATTCTTTGCATATATGCTTGATAATGGTGTATA
TTTAGCGCCATCAGCTTATGAAGCAGGTTTTATTTCAATAGCCCATAGTGATGAAGATATTGAAAAAACC
ATTTATCTTGCTAAAAAATTTTTTCAAGAGAATTAG

```
>lcl|NC_006570.2_cds_YP_169930.1_818 [gene=bioB] [protein=biotin synthase]
[protein_id=YP_169930.1] [location=complement(948397..949338)]
ATGACATTACAACAAATCAAAGAAATTTACTCAAGACCATTAACAGAATTAATTTTACAAGCTTTAGAGA
TTCATAATAAAAATTTTGGTAATGATATCGAGTTATGCTCACTAAAAAGTATCAAAACTGGTACTTGCCC
AGAAGACTGTAAATATTGTCCTCAAAGTGGTCATTATAATACTAGTATCGAAAAGCATAAACTATTAGAT
AAAGATAGTATATTGGCAGAAGCAAAAAAATGCTAAAGATGCTGGATCTAAAAGGTTCTGTATGGGCGCTG
CTTGGAAACACATTCCTAAAAAAGACTTTGATCAAGTTGCTGAGATTATCACTGAAGTCAAAAACCTTGG
CTTAGAAACATGTGTGACTCTCGGAAGTATTAATGCTGATGAAGCAACTAAACTAAAGCAAGCAGGATTG
GATTACTACAATCATAATCTTGATACTTCAAGAGAATTTTATCCGGAAATAATTACCACACGTAAATTTG
AAGAAAGAATTGAAACTATTAGAAATGTTGCTAACGCCGATATAAATGTCTGTTGTGGTGGTATATTGGG
TATGGGCGAATCTCTAGATGATAGATTTAATTTATTACTAGAGTTATTACAACTACCTGCTGCTCCAAAA
AGTATTCCTATCAATACATTAATACCTATTAAAGGAACTCCTTTAGGAGATAAGTATACAAATGCACAAA
TTGATAGTTTTGAATTAGTAAGATTTATTGCAACTACAAGAATACTATTTCCACAAGCACGCCTAAGGTT
ATCAGCAGGTAGAGAAAATATGTCTTTGGAAACTCAAACTCTATGTTTCCTTGCTGGTATTAACTCAATA
TTTTATGGTAATAAGTTATTAACAGAAAATAATGCTACTGTTAATTCTGATAATTTTTTATTAGCTAAGC
TTGGCTTAAAATCAAATGCTGAATTATGTTAA

>lcl|NC_006570.2_cds_YP_169935.1_823 [gene=folK] [protein=2-amino-4-hydroxy-6-
hydroxymethyldihydropteridine pyrophosphokinase] [protein_id=YP_169935.1]
[location=complement(953366..954631)]
GTGCAATATATTATAGGAATTGGAACAAATATTGGTTTTACTATCGAAAATATTCATCTTGCAATAACTG
CACTAGAATCGCAACAAAATATAAGAATCATCAGAAAAGCAAGCTTATACAGTAGTAAAGCTGTCCTTAA
AGAAGACGCTCCTAAAGAATGGGATATTAGATTTTTAAATACAGCTGTAAAAATTAGCTCTTCACTAAAG
CCTGATGAACTTTTAGTACTCTTAAAAGACATAGAATTAAAAATAGGTAGAGACCTAAATGCTCCTGCAT
GGTCACCTCGAGTAATTGATTTAGATATTCTTGCTGCTGAAGATCTAATTTTAGAAACAGACAAACTTAC
TATTCCTCATAAAGAATTAATTAATCGTAGTTTTGCGTTGGCTCCTTTATTAGAATTATCAAAGGGTTGG
CATCACCCTAAATATGTTGAATGGGATCTTAATATAAGATTAAAAGAATTAGGTGAGATAGTAAAACTTA
AACAAACTCTTGCAAATACAATACGTATGGGTATAGTTAATCTGTCAAATCAATCTTTTTCAGATGGTAA
TTTTGATGATAATCAACGTAAATTAAACCTTGATGAGCTAATACAAAGTGGTGCTGAAATTATTGATATC
GGAGCTGAATCGACTAAGCCTGATGCCAAGCCTATATCGATTGAAGAAGAATTTAACAAATTAGATGAAT
TCCTAGAATACTTTAAATCACAACTGGCAAATTTGATTTATAAACCATTAGTCAGTATTGACACACGCAA
ACTAGAGGTAATGCAAAAAATTCTCGCTAAGCATCATGATATTATCTGGATGATAAATGATGTTGAATGT
AATAATATTGAGCAAAAAGCACAGCTTATAGCTAAATATAATAAAAAGTATGTTATAATTCATAATTTGG
GTATTACAGATAGAAATCAATATTTAGATAAAGAAAATGCTATAGATAATGTTTGTGATTATATTGAGCA
AAAAAAGCAAATTCTTCTAAAACATGGTATAGCACAACAAAATATTTATTTTGATATTGGCTTTGGTTTT
GGTAAAAAATCAGATACCGCTAGATACTTATTAGAGAATATCATCGAGATAAAAAGAAGATTAGAATTAA
AAGCATTAGTTGGTCACTCACGTAAGCCATCAGTTTTAGGATTAGCAAAAGATAGTAATTTAGCAACTTT
AGATCGAGCTACAAGAGAGCTTTCAAGAAAATTAGAGAAACTAGATATTGATATTATAAGAGTACACAAG
ATTTAA

>lcl|NC_006570.2_cds_YP_169936.1_824 [gene=folB] [protein=dihydroneopterin
aldolase] [protein_id=YP_169936.1] [location=complement(954633..954986)]
ATGAAACAATCTTTATTTTTAAATGATGTTAAAATATATGTGAGTCTTGGTTGCTCAGGAGAAGAAAGAG
CCTACAAACAAATGATAACTCTTGATCTAGAGCTAGAATTTAGCCAAAATTATAGAGCTAGTGATAGTGA
TAATCTTGAAGAAACTATTTGTTACTACACTCTAAGAAATAATATCCAACAATTTTGTGATAGTATCAGC
TGTAATCTTATAGAGTATTTAGCTAAACAAATTTATCTATTTATTCAAAAAAACTATAGTGATATAACTA
TCAAATACTTGAAAATTAACAAAAAGCCCCCAGTTTCTCAAATAGAGTCAGCTTGTTTTATAATTAGGAA
CTAG

>lcl|NC_006570.2_cds_YP_169941.1_829 [gene=folE] [protein=GTP cyclohydrolase I]
[protein_id=YP_169941.1] [location=complement(963398..964018)]
TTGATGATGAAAGATAAATATTGCTCTAAATTAGGTAAAAGTGTCCAAGAACATCTCATCAAGTTAGGTT
TAGAGCAACCTAGAGAATTTAACCTTGATAATGATAATAAAATAACCATAATCACCAAAGCATACCGACA
AATCTTAGATGCTCTCGGCTTATACACAGATGAATTTGAAAAAACTCCATTTAGAGTAGCGAGGATGTTT
ACTCAAGAAATATTTAATGGACTTGATTATGCTAATTTCCCTGCTTGTGCTTTATACGAGAACGAGTTTA
ACTATACTGGTGTGCTAACTCAAAAAAATATAACTATAATGTCATTTTGTGAGCACCATTTTGTACCTTT
TGAAGGTACTGCAGAAGTTTCTTTTATTCCTAAAAATAATAATATTATAGGGCTTTGTCGTATAAATAGC
ATTTGTGACTTTTTTTCCAGACGCCCACAAATACAAGAAAGAATGACCGCTCAAATATTTGAGGCCTTAA
AATTCATTTTATCGACAGAAGATGTTAGTGTGAAAATTAAGGCAAAACATGCTTGTGTCTCACTTAGAGG
GGTAAATAATCAAAATTCTCAAACATATACACAAATGGTTGGTGGAGTTTTTGCTAAATAA
```

>lcl|NC_006570.2_cds_YP_169954.1_842 [gene=ffh] [protein=signal recognition particle protein, Ffh] [protein_id=YP_169954.1] [location=complement(975474..976850)]
ATGTTTACTAGTTTATCAGAGAAATTACAATCGTCTTTTAAAAAGATAAAAGGCCAAACCTCTCTAACAG
AGGAGAATATTCAATCAGCGCTGCGTGATATTAGGGTATCACTTTTAGAAGCTGATGTTGCCTTGCCAGT
AGTTAAAAAATTTATAGCTAATATTAAAGAAAAAGCTATTGGCGAAGAAGTCAAAAAAAGTCTTACCCCA
GATCAGACTTTTATTTCTTTTGTCAAAAAAGAAATTGAAAAAGCACTCGGTGAAGAAGCTGTACCAATAA
ACCTAAAAACTCAGCCACCTGCTGTGATATTAATGGCTGGTTTACAAGGTGCTGGTAAAACAACATCTAC
AGCTAAGCTGGCTAAATACTTAAAAGAGCAACATAATAAAAAAGTCATGGTAGTTAGTGCCGATGTCTAT
CGCCCTGCTGCTATCGACCAGCTAAGAACTTTAGCAAATAGTTTAAATGTTGAGTTTTTTAAATCAGATG
CTTCACAACAGCCAGAAGATATTGTAACAGCTGCGATAAAAACAGCAAAAACTAAACTCATAGATGTGCT
TATCATCGATACTGCTGGTAGATTGCACATAGATAATGATATGATGGATGAGATTAAACAAATCCACAAA
ATTGCTAAACCAATCGAAACGTTTTTTACAGTTGATAGTATGACTGGTCAAGATGCTGCAGTTACAGCTA
AAGCATTTAATGACGCTTTAGAGTTAACTGGCGTGATTTTGACAAAAACTGATGGTGATGCTAGAGGTGG
TGCTGCTTTATCGATTCGAGAAATCACAGGCAAACCAATCAAGTTTTTAGGTGTCGGTGAAAAGACTGAT
GCTTTAGAGCCATTCCACCCTGATAGAGTTGCTTCTAAGATTTTAGGTATGGGCGATGTTCTTAGCTTGA
TAGAGAGTATAGAACAAAAAACAGAAAAAAAATCTGCTGAACAACTAACTAAAAAACTTAAAAGTGGTAA
GAGCTTTGATTTAGAAGATTTCAAAGCACAAATCCAACAGATGAAGAAAATGGGTGGTGTAGGTTCAATT
ATGTCTAAACTGCCAAATATGCCAGCAAATTTGCCAGGTGACGTTGGTGATGATATGTTTAAAAAAATTG
AGGCTATGATAGACTCGATGACTCCACTTGAACGTAAAAAACCAGAACTTATCAAGCATAGTAGAAAGCA
GCGAATTATTAAAGGATCTGGAACTACAATTCAAGATCTTAATAAACTACTTCAGCAACACACGCAAATA
AAAAAAATGATGAAAAGCGTTATTGGTAAAAAAGGTGGTATGGCAAACTTAATGAAACGTATGTCTGCTA
TGCAAGGTATGGCGAATATGCCAGGTCTTTTTGGTAAAAGAAAATAA

>lcl|NC_006570.2_cds_YP_169956.1_844 [gene=infA] [protein=translation initiation factor IF-1] [protein_id=YP_169956.1] [location=978193..978411]
ATGGCGAAAGAAGATTGTATAGAAATGGAAGGCGTTGTTTTAGAAGCACTTCCAAACACAATGTTTAGAG
TTGAACTTGAAAATGGACGTATAGTAACAGCTCATATCTCAGGTAAAATGAGAAAAAATTATATCCGTAT
ATTAACAGGTGATAAAGTTGTAGTTGAAATTACTCCTTATGATTTAACAAAAGGTCGCATTAAGTTCCGC
AGCAAATAA

>lcl|NC_006570.2_cds_YP_169957.1_845 [gene=FTT_0968c] [protein=amino acid antiporter] [protein_id=YP_169957.1] [location=complement(979373..980788)]
ATGACAGTAAATTCATCAGCAAAAGAAAAAAATTGGTTTAATTTTACTAGTATTGTTAATGACAGGAGCTA
TTGATAATATTAGAAATCTTCCATCTACAGCCACTTCAGGAACATATATATTCTTCTTTTTTGCAGTAGC
TGTTTTTTTATTTTTAGCACCAGTTGCATTAGTATCTGCAGAGATGACTACGACATATACTGCTAAAGGT
GAAGAAGGAGTATATGGTTGGGTTAAAAAAGCTTTTGGCCCTAATGTTGCGATGTTAGCTGTATGGTTCC
AATGGATAAATACTCTTATCTGGTTTCCAAGTATTTTGACTTTCCTTGCAGGTACAATTGCATACTTATT
TAATCCCGATTTTGCACAGAATATAAAATTTACAATTATATTTATAACTGTAGTTTTTTGGTCTTTGACA
ATTCTTAATCTTAAAGGTCTAAGAGTATCGGCAATTTTCGCTAGTACTTGTACTTTTTTAGGTATGGTAA
TACCGATGTTATTAATGGTGCTATTTGCATTAATTTGGTTACTCAATATCTATGACCTTAATATTCATTT
TCATCTAAACAATCTAATTCCTAGTTTTACATCTACAGATTCATGGATGGGACTAACAGCGATTATAGCT
TCTTTCTTAGGACTTGAGTTAGCAACCGTGCATATTAGAAAAGTTATAAATCCTAAAAAAACATTTCCTC
TAGCACTTTTTATTTCAGTAATATTTATAATCTTTACTATGGTTTTAGGTGCTTTAGCTGTTGCGATTAT
ATTTCCACAGTCCCAAATTGATGTTGTTCATGGTACTATCAAAACTTTTAAAGTTTATTTAGAGAGTTTG
GGAATACCTGTATTTTTTTATTATATTTTGGGCTTGATGGTTTTTGTTGGTTCAATAGGATCAATGATTA
ACTGGATGATTTCACCTGCAAGAGGATTACTTCAAGCAGCCGATGATCATTTTTTACCAAATATGTTAGA
TAAAACAAATAAGCATGATGTACCAAGTGACATATTAATCCTTCAGGCTATAATTATGACTATAATATGT
CTGCTTTTAGAATTAGTGCCTTCAGTTCAAGCTTATTATTGGTTGCTTACAGCTCTTAGCACACAAATAT
ATTCTTTGATGTATCTAATGATGTTTTTTGCCGCTTTAAAACTAAAACTAACAAATAACCAAACTGTAAG
AAATACCGATGATTTTCATATCCCTGGTGGGAAAATCGGTATGTCTATTGTTTGTGTTTTAGGTATTATA
GGCACTATTTTGTGTGTGATCGTTGGCTTTGTCCCACCAGATAATTTGTATAGTAATCCGCTAGAGTTTA
TTGAAATGCTGTCTGTATGTTTTGTATTATCAATTATACCTGTAGTATTCTTTATCATATATAGAAGAAT
TAAATTAAAAAATTAA

>lcl|NC_006570.2_cds_YP_169958.1_846 [gene=trkA] [protein=potassium transporter peripheral membrane protein] [protein_id=YP_169958.1] [location=complement(980853..982226)]
ATGAGAATAGCCATTTTAGGAGCTGGGCAATTAGGCGTATATTTAACCCAAAGGCTTAGTTTAGACCATC
AAGTTTCTGTTATTGACCTAGATGAAGAAAAACTTGGTTTTATTTCATCAGCATTTGATGTCCAAACAAT
CATTGGTGATGTTACCAAACCAAACATTATGATGGAAGCTAATTTTAAAGATACTGATATGATAATAGCT
GTGACATCAAATGATACTACAAATATAGCCGTATGCGATATGGCTTATAAGTTGTATAAAACACCATATA
AAATAGCCCGTATTCGTGACACTGAATATAATAGATTTCCTAAACTTTTAAATAATATAGATTTAGTCAT
AAAGTCATTTTTTGAAACAACAAAAAGATTAGAACAATTAATCTTCTTGTCTGGAGCATATTTTATATCA

AGTTTTTTTGATAAGCGTGTTCAGATTGTTGGTGTTGAGGTTTCCTCAGACTCCCCTCTTGTAGGACTTG
CAGTTAAAGATATATATCTAGGTTTAGGTGATATCAAAGTTGATATTATCTCTGTATATAGAGGTAATGA
AAAATTAGATATTGATGATATTAATGTCTTAGTAAAGCCTGGGGATAGAGTTATGTACCTTTCCGAAAAA
GCATATTCATCACAGATACTCTCAATATTTCAACCTAAAAAGACCAATATCAGAAAAATATTTATAGCAG
GTATAAACTATGCAAGTATCACTTTAGCAAAATCTTTGGAGAGTAAAGGATATATTATTAAAATGATAGA
CCCAAGTGCAGAAAAATGTGAGTTTGCTTTGAGTGAATTGTCTAAATCAACAATTTTACATTATAACCCT
GTTAATAATAACCTACTGGTTGCTGAAGGTATAGATGAGGCAGATATGTTTTTTGCTTTGACAAACTCTG
ATGAAATAAATATTATGTCATCAATTTTGGCAAAAAAACTTGGTGCTAAAAAAACAGTCGCTACCGTTAA
TAGTTCTGAATACTACGATATTACAAGGGATCTAAAACTAATTGATATTTCTATTTCGCCACATAATTTT
TCATATACAACAATCAAGGCATTTTTAACTCAAGTTGATATGCTAAGAATGTACGAAATCGAAGATAGCG
AAGAAATGCTTGTTGAGCTTAAAGTCCATGGTCAAGAAAACATGTCCACAGTTATTGGTAAAAAGATTAA
TGACTTAAAATTACCTCAAGGTCTAGAGATTATTGCAATAATGAAAATTGATAATATTCCAAGGTTTTAT
GCCGATAGTTTTCTAATACAAGATCAAGATAGATTAATCATCAAAGTGGATAATAAAAATGCTCTACAAA
CCTTAGAAAAACTTTTTCAAGTAATGCCATTATACATCGCATAA

>lcl|NC_006570.2_cds_YP_169960.1_848 [gene=FTT_0971] [protein=cysteine desulfurase] [protein_id=YP_169960.1] [location=982796..984241]
ATGAGTGAAAATTTAGATAAAATCATTGAGCAAGACTATGAGCATGGTTTTGTTACTAATATTGAAGCAG
AAACAATTGAGGCTGGTCTTAATGAAGATGTAATCCGTTTGATATCTGCAAGAAAAAACGAACTTGAGTT
TTTATTGGAATGGCGCCTCAAAGCTTATCACAAATGGCTAGAGATGAAGTCACCTAAATGGGCAGATTTA
AATTATCCTCCTATAGATTTTCAAGCTATTAGTTATTACTCATCACCCAAGTCACTTAAAAATCATCCAA
AAAGCTTAGATGAAGTTGATCCAGAGATCATTGAGACTTACAACAAGCTTGGTATTCATTTACATGAGCA
AGAAATGTTAGCGGGCGTCAGAAATATTGCTGTAGATGCGGTATTTGACTCAGTGTCTGTTGTAACAACA
TTCAAAGAAAAATTAGCAGAAGCTGGAGTAATATTTTGCCCAATATCAGAAGCTGTACAAAAATACCCTG
AACTTGTACAGAAATATCTGGGATCAGTAGTTCCTCAAGGAGACAATTTCTTTGCTGCGCTTAATTCAGC
AGTATTTAGTGACGGGTCATTCGTGTATATCCCTAAAGGTGTAACTTGCCCAATGGAATTATCAACGTAC
TTTAGAATCAATGCTATGAACACAGGGCAGTTTGAGAGAACTTTGATTATAGCAGATGAGGGTAGTTATG
TAAGCTACTTAGAAGGTTGTACTGCACCAATGCGTGATGAAAACCAACTTCATGCTGCAGTTGTTGAGTT
AGTGGCACTAGATGGTGCTGAGATTAAATACTCGACAGTACAAAACTGGTACCCAGGTGATAAAGATGGC
AAAGGTGGAATTTATAATTTTGTAACTAAAAGAGGGGTCTGTCATAAAAATGCAAAAATTTCATGGACAC
AAGTTGAAACAGGTTCAGCTATTACATGGAAATATCCTTCTGTAGTTTTGCGTGGTGATAACTCAATTGG
TGAATTCTATTCAGTAGCATTAACTCGCCATGCTCAGCAAGCTGATACAGGTACAAAAATGATTCATCTT
GGTAAAAACACCAAAAGTACAATCATTTCAAAAGGTATATCTGCAGGTAAAGCATCACAAGCATACAGAG
GTTTAGTAAGGATTTCTCCAAATGCTGCTAATGCAAGAAATTTTTCACAATGTGACTCTTTATTAATTGG
TCATAATTGTGAAGCACATACTTATCCATATATAGAAAACAAAAGTAACTCCTCACAGATAGAGCATGAA
GCTACAACCTCTAAGATTTCTGATGATCAACTCTTTTATTGTAAGCAAAGAGGTTTATCAGAAGAAGATG
CGATCGCTATGATTGTAAATGGCTTCTGTAAAGAGGTATTTAAAAAACTTCCACTTGAGTTTGCTGTTGA
AGCTCAGAAGCTTATGGAAGTCAGTCTAGAAGGTGCAGTTGGTTAA

>lcl|NC_006570.2_cds_YP_169961.1_849 [gene=FTT_0972] [protein=ABC transporter ATP-binding protein] [protein_id=YP_169961.1] [location=984280..985029]
ATGTTGTTAGAAATTAAAGATTTACATGTAAGTGTTGGTGAACAAAAAAAACAAATACTAAAAGGATTAA
ACTTAACAGTTAAAAAAGGTGAAGTCCATGCAATTATGGGACCAAATGGAGCTGGCAAAAGTACTTTAAG
TAATGTTTTAGCTGGTAAAGATGGTTATGAAATTACTCAAGGTTCAATCACATTTGATGGTAAAGATTTA
AATGATTTAAGTATCTCAGAAAGAGCTGCAGCCGGCATATTTTTAAGCTTACAGTATCCAATTGAAATCC
CTGGAGTTAGTAATGTGCAGTTCCTTAAAACCGCTTTAAATAGTATTAGAAAGCAAAATGGTGAAGATGA
AATTGATGCAATTTCTTTTATGAAAAAACTAAAAGAGAATATGCAGGTATTAAAAATTGATCAAAAATAT
ATGTCACGAGGAGTTAATGAAGGCTTCTCAGGTGGTGAGAAAAAACGTAATGAAATGCTGCAACTTATGA
TGCTTGAGCCAAAATTAGCTATACTAGATGAAACTGATTCTGGTCTAGACATAGATGCTCTACAAGTAGT
ATCACAAGGGGCAAACAGTATGAGATCAGCAGACAGAAGCTTTTTAGTAATCACTCACTACCAAAGACTT
CTAGATCATATTCAACCAGATTTTGTCCATGTTTTAGCAGATGGTAAAATAGTTAAGACTGGTGGAAAAG
AGCTTGCTCTTGAGTTAGAAGAGAAAGGTTATTCTTGGTTAAACAGCTAA

>lcl|NC_006570.2_cds_YP_169962.1_850 [gene=FTT_0973] [protein=hypothetical protein] [protein_id=YP_169962.1] [location=985033..986187]
TTGCTAAAAAATGTTAATAGATAATAAATCTCTGCCTACTACTAAACAAGAAAGCTGGAAGTATACAAATA
TTGCTTCTATTTATGAGAAGAATAATATTAAAGAATTATTGATAGAGTCACCTAACTCAAAAGACTATCT
TGAAGGCTTTAAGTTTGACACACAAGAGAATGTTGTGATAATTCTTGATGGCGTTTTAGCTATCGATTAT
AACAAAAAATTAAACCATATTAGTGCCTTAGAATTACATAAAGATGACAGAAATATGTCGAGGTTAGCTA
TAGAAAATTCTAAACATTTTGGTATCAATATAGCCAAAGATACTAAAGATTACTTAAGCCTAATCTTCAT
AAATACTGATATGGCAAAAGATAAATTAACAAATATATCTCTAAAGCTTGATGTTGATATGTTTGCTAGT
CTAGATCTAGATATTGATTTTGTAAATCTTACTGAAAATTCTGCGATTAATTTATACTTTGATATAAATG
TTGCTGAGGCTGCAAAAGTCAATTTTACAAATAATTCTAATAATCCAAATAATTCTAAGTTAATTACAAC
AGCAAACTATTTGATAAATTTAGATAGAGCTGCTGAGTTTAACGGTTTTAATCTTCTTAATAAAGATGCT

TTATTAAGAAATGATTTTGTAGTTAATCTCAACAAGCCTCATTCAAAGTTTGATGTTAGAGGACTATACT
TAATAAATGATAGTGCTATTGCCAACACTTGTTTTTTGGTTAATCATAATGCTTCGCATACTTATAGTAA
TGTCAACTTTCGCGGTGTTGCTAATGGTAATGCTAAGGCTTGGTTTAATGCTAAAGCAATAGTTAATAAG
GGTATTGAACAAATTCAAGCATATCAAAATAACAAAAATATTCAATTAAGCAATAAAGCTGAAATAAATA
CTAAGCCGGAGCTAGAAATTTTTGCTGATGATGTTGTGTGTACTCATGGCGCAACGATAGGGCAATTAGA
TAAAGACGCACTATTTTATTTACAATCTAGGGGATTAGAACTTCATGATGCTCAGCACTTACTATTAGAA
AGTTTTGTAAAATCTCAACTCACAAGTGATGATTTTCCTTTTGAAAATGAAATCAAAGAGGAGATAGTTG
AGTCTTTGAAGGATATTCTCCATAGTATCATTTAA

>lcl|NC_006570.2_cds_YP_169974.1_861 [gene=FTT_0986] [protein=hypothetical protein] [protein_id=YP_169974.1] [location=995821..996231]
ATGTTTCAATCATTGATAGCTATTGATTATGGTAAAGCACGCATTGGTATATCTAGTGGTCAAATGATTA
CTAAAACCGCCACACCTATAGGCACTGTTGAAGCTTATGATGGTGTTCCTAACTGGATTGAACTTGATAA
AATCATCAAATGCTGGAATCCTTCAGATATCATTATTGGCCTACCATTAGATACTCAGAATTTTGAAACA
GATATTACTAAATCTGCTAAAGATTTTGCCAAAGAAGTACAGCAAAGGTATCAAAGAAAAGTTCATCTGA
TTAACGAAGCTTACTCTACTCGTGAAGCTAGATGGCGTTTGGAAGAAGTCAAAAGTAAAAAAGTCTCTCA
TATTAAGGTAGATGCTCTGGCAGCCTGTGTAATTTTAGAAACATGGATGTCTGAAAATTAA

>lcl|NC_006570.2_cds_YP_169978.1_865 [gene=leuS] [protein=leucyl-tRNA synthetase] [protein_id=YP_169978.1] [location=998929..1001370]
ATGAATGAATATAATTTTAGTGATATAGAAAAATCGACACAAGAGTATTGGCGTAAAAATGATACTTTTA
AAACAATAGAAGATAATACTAAAGAAAAATTTTATTGTCTTTCAATGTTACCCTACCCAAGTGGTACTCT
ACACATGGGACATGTAAGAAATTACACGATAGGTGATGTCATAGCTAGATATCAAAAAATGCAAGGTAAA
AACGTCCTTCATCCTATGGGTTGGGATGCCTTTGGTCTACCTGCAGAAAATGCAGCGATTAAGCACAAAA
AATCACCATACGAATGGACAAAAAGTAATATTGCTTACATGAGATCGCAGTTTGACTCTCTAGGCTTTAG
TTTTGACTGGTCAAGAGAGATTACAACTTGTGATGAAGATTACTATAAATGGGAGCAATGGTTTTTTATT
CAGCTATACAAAAAAAGGTTTAGCATATCGTAAAAATTCAGTCGTTAACTGGGATCCGGTTGATCAAACAG
TTTTAGCAAATGAACAAGTTGTTGATGGTAGAGGTTGGAGATCTGGTGCATTAGTTGAGAAAAAAGAAAT
TCCTCAATGGTTTTTGAAAATTACCGATTATGCTGACGAGCTTTTGCAAGATATCAACAAATTAGATAAT
TGGCCAGAGGCTGTTAAAACTATGCAAATTAACTGGATTGGTAAATCCAAAGGTTTAACAGTTAAGTTTA
AGGTTAAAGACTCTAATCAAGAAATAGAGGTTTTCACAACTCGTCCAGATACTCTTATGGGAGTAAATTA
TCTTGGAATAGCTCCTGAGCACCCTCTTGCTCTTAAAGAAGCTAAGTCTAATTCTCAATTGGCAGCATTT
ATCGAAGAGTGTAAAAAAACTTCAACCATGGAAGCTGATCTTGCTACTCAAGAAAAGAAAGGATTTAAGA
CATCTATTAAAGTGATTCATCCTATTTCTGCTGAAACTATAGATGTTTGGGTAGCTAATTTTGTGCTTAT
GGGATATGGTTCTGGTGCGGTTATGTCTGTGCCAGCTCACGATCAAAGAGATTGGGAATTTGCACAAAAA
TATAATATACCATTAAAACAAGTTATAGAGTCTAATGATAACAAGTTAAAAATTGATTTAGAAAAACAAG
CTTTCACAGAAAAAGGTATTTTGATTAACTCAGGTGAATTTGATGGTCTAAACTTTAAAAATGCTTATCA
GGCTATTAAGAAATATCTTACAGAGCAAAATAAAGGTTATGAGACTACTAATTTTAGAATTCATGATTGG
GGTATTTCACGTCAAAGATATTGGGGTTGCCCTATTCCTATGATTCATTGCGACGATTGTGGTGCTGTAC
CAGAAAAAGAAGAGAACTTACCAGTAAGGTTACCTACTGATGTAGCCTTAACAGAAGCAGGCTCACCACT
TAAAGATATTCCAGAGTTTATAAATGTAGCTTGTCCAGAATGTGGTAAACCAGCAAAGCGTGAAACTGAT
ACATTTGATACATTTTTTGAGTCATCTTGGTATTATGCAAGATATACTTGCCCTACCGCTAATCAGATGC
TTGACCAAGAAGCTAACTATTGGTTACCAGTTGATAAATATATTGGTGGTATTGAGCATGCTATTATGCA
TTTATTATATGCAAGATTCTTCCATAAATTAATGAGAGATCAAGGTTTAGTAAAGTCTGATGAACCTTTT
AAGAACCTTCTTACGCAAGGGATGGTGTTAAAAGATGGTGCAAAAATGTCTAAGTCTAAAGGAAATATTG
TAGATCCTCAAGAGCTTATTGATAAATATGGAGCAGATACTGTAAGATTATTTAGTATGTTTGCTGCACC
GCCTGAACAATCACTTGAATGGTCTGAAACAGGTGTCGAAGGAGCAAATAAATTTCTACGCAAAGTTTTT
AATTATGCCGAACTAAATAAAGTTATATTTGCCAAAAATATAACGCTAGAGTCTCAGAAACTTACAAAAG
AAGATAAAAAAGCACGTTTTGAAATACACTCTAACCTAAAGCAAGCTATTTTTGATTTTGATAAGAGTCA
GTTTAATACTGTAGTATCTGCTTGTATGAAAATTTTAAATACTCTTAATAACTATGATAACCTTTCTGAA
AGTGTAAAGGTTGAGGGATTTAGCATTTTATTAAGAATTCTAGCGCCATTTACTCCTCACTTATGTCATT
ATCTATGGCAACAATTAAATTTAGGAGAAGATATACTTCATACTAGCTTCCCAACAGTTGATAATAATGC
TCTAGAAAAAGATGAGTTTCTTTTAGTAGTACAGATTAATGGTAAGTTAAAAGCTAAACTAGAATTAGAT
GCTTCATTATCTTCAAATCAAGTTGAAGAAGTAGTGTTAGCTGATGAACATGTCAAATCATTTATAGATA
ATAAACAAGTTGTTAAAGTAATTTATGTACCACAAAAACTTATTAATATTGTAATTAAATAA

>lcl|NC_006570.2_cds_YP_169982.1_869 [gene=FTT_0994c] [protein=MRP like protein] [protein_id=YP_169982.1] [location=complement(1003789..1004649)]
ATGATAAGAATCGAAAATGTTGTCAAAAGAAAAGTTCAACAAGGGCAAAAACTTTTACCTAATATTAAGA
ATATAATTTTAATTGCTTCTGGAAAAGGAGGTGTTGGTAAATCTACTGTAACAGCAAATCTAGCAGTTTG
CTTTGCAAAGATGGGAGCGAAAGTTGGTATTTTAGATGCTGATATTTATGGTCCAAGTCAGCCAACATTA
TTTGATCTAAAACAAAACCCTAATACTACAGATAAGAAAAAGATTATCCCTTTGGAAAAATACGCAGTTA
AGATGATTTCTATAGGGAATCTGATAGATCCTGAATCAGCAGTAATTTGGCGTGGTCCTATTGTATCTAG
AGCGTTGATGCAACTTTTGAATGATACAGACTGGGGTGATATAGATTATTTATTTTTAGATTTACCTCCT

GGAACAGGTGATATTCAGTTAACAATATCAAAAAATATGCCAGTTACAGGAGCAGTGATTGTTACAACTC
CTCAAGATTTATCATTAATTGATGCTAGAAGGGCTTTAGCAATGTTCCAAAAAGTTGATATCAAAACTCT
AGGTGTAGTAGAGAATATGAGTTATTACATTTGTCCTAAATGTGGTAATAGTGAACATATTTTTGGTGAG
GATGGTGCTCATCTGTTGTGTGGTAAAAATAATATTGAGTTTTTAGGTAGTTTACCGCTACATAAGGATA
TTCGTGAGAATGCTGATAATGGTAAACCTTATGTTAGTCTAGACAAGGATGATAGTATTAATACGAGTTA
TATGACTGTAGCTGAAAATATTTTAAATCAAATTGAGAAGTTACCTAAGGCAAGTAGTTTAGATTCTATT
GGTGTTAAATTAGAAAATTAA

>lcl|NC_006570.2_cds_YP_169988.1_875 [gene=pheT] [protein=phenylalanyl-tRNA
synthetase subunit beta] [protein_id=YP_169988.1]
[location=complement(1012720..1015092)]
ATGAAATTTTCACACAATTGGTTAAATGAATATCTAGGTGATACTCAGGATAGCCAAAATCTAGCAGATA
CTTTAACTTTAGCTGGTTTAGAAGTAGATGCAATTGAACCGGTAGTCGCAGAAAAAGTTAGTGGTGTAGT
AGTTGGACAAATTAAAACCATTAATAAACATCCTGATGCAGATAAATTAAATGTTTGTAGTGTCGATGCA
GGTGAAGATGAGTTGCTAACGATAGTTTGTGGTGCAAGTAATATTTACGAAGGTATGAAAGCTCCTGTTG
CAAAAATTGGTGCGGTACTACCAGGTAATTTTAAAATCAAAAAATCTAAACTGCGTGGTCAAGAATCTTT
TGGGATGATGTGCTCGGAAGAAGAGCTTGGCTTAGCTGAAAAAGCTGATGGTTTAATGGATTTACCTATA
GATGCTCCGGTTGGTACTGATATAAATAAATATTTAAATTTAGATGATAATATTATCGAAGTTGATCTAA
CACCAAATAGAGCAGATTGCCTAAGTGTATATGGTATTGCTCGTGAAGTATCAGCATTAACAAAAACTGA
ACTTAAAAACTTAGAAATACCAGAACCTAAAGTGGTGATAGATGATACTAAAGAAGTCAATATAACTGCT
ACAGATGCTTGTCATGCATACTATGGCTGTATTATCAAAAATGTCAATAATAAAATTCAAACACCTTTGT
GGATGGTTGAAAAACTAAGAAGAAGTGGTATTGGTAGTATTTCATTTTTTGTTGATGTAACAAACTATGT
AATGCTACTTACTGGTCAACCAATGCATGCATTTGACTTAGACAAGTTAGAGGGTAGAATTAATGTCCGT
TATGCAAAAAATAATGAGGAGCTAACACTACTTGATCAAACACAAGTTAAATTGGATTGTGATACTTTAA
TAATAGCAGACGATAAGAAAGCTCTTGCTATAGCTGGTGTTATGGGCGGCTTAGACTCATCAATTACTGA
TAGTACTACTAATATATTTTTAGAAAGTGCATTCTTTGTACCAGAAAAAATTGCTGGTAAAGCGCGTAAA
TATAATCTTCATACAGATTCATCACATAGATTTGAGCGTGGTGTTGATCCACAATTAGCTAAAAAAGCTA
TGAAAATAGCTATTAGATTAATAAACGAAATAGCTGGTGGTGAAGTTGCACCAATTCATGGAGCTGAGGA
TTTAGCAAACTTAAACAAACAAATCAAAATAAATCTTTCTATTAAGAAGCTAAATCACGTGTTAGGTACA
AACTTTGATATAGAATATGTAACAGAAGTACTTAAAGCATTACATATGGATGTTGCAACAAGTTTTGATG
GTAATTGTATAGAGGTAATACCTCCTTCATATCGTTTTGATTTGGAAATTCCTGAAGATTTAATTGAAGA
AGTTGCTCGTATATACGGTTATTCAAAACTTCCAGAAACTATGCCTAAATATGCAGCAGCTAAAACTAAT
ATCTCTGAAACATACCAGTCTTTAGATACACTAAATATGCGTTTGATAGATAGAGGCTATCATGAGACTA
TTAACTATAGTTTTATAGATCCAAAATTTGATGAGTTTTTCTTTGCTGATAGAGGTATTGCAATTCAAAA
TCCTATTTCACAAGATTTATCTATAATGAGACAATCTTTGATACCTGGTTTGATTAATACTTTTAAGGCA
AATACATCACGTCAACAAAATCGAGTTAGAATATTTGAGAAGGGTGCTTGTTTTAAACTTCAAGATAACC
AAAGAATCCAATTTGATAGAATTGCAGGGTTAGCGTATGGAGAGCTTTTAAATATTAATTGGTCTAATTC
TAAGAAAGTAGACTTTTTTGATGTTAAAGCAGATGTTGAAGCATTATGTAATGATCTTACAAGCTTAAGT
TTTGAAGTTTGTAATGATATTAATTGGCTGCATCTAGGTCAATCTGCTTATATCTTAGCTAATGGTAATA
AGATTGGTGTAATCGGTGTAATTCACCCCACAGTTTTAAAAAATTTCCAAATCAAAGCAAAGGCTCCTAT
AGTTTTTGAATTAGATTTAGATGTTTTGATTAAAAGACAAATACCAAACTTTACTAAAATTTCTAAGTAT
CCCTCAGTATCGAGAGATATATCATTCTTAGTTGATAAATCTGTACTTGCTGGAGATATTATCAAAGCTA
TAAAGGCTTTAAATATAAATATCTTAAAAGATGTAAGTATTTTTGATATATATGAATCACAAGATAGTGA
TAGAAAGAGTATTGCTCTAAATATGCTTTTCCAAGATAACTTACAAACTTTAGATGATAAGGTTATTGTT
GAGAGTATAGATAAAGTTCTAGAAGCTCTGAAAACTAAATTTAATATTGAACAAAGAGTTTAA

>lcl|NC_006570.2_cds_YP_169989.1_876 [gene=pheS] [protein=phenylalanyl-tRNA
synthetase subunit alpha] [protein_id=YP_169989.1]
[location=complement(1015113..1016126)]
ATGCAAATAGTCGAGCAAATGAAAGATAAAGCTCTTGCTGAGCTAAATCTTGTCAAAGATAAAAAAACTT
TAGATGATATTCGAGTAAAGTACCTGGGTAAAAAGGGTGAATTAACTGAGATGATGAAGTTAATAGCGAC
ATTACCTAATGATGAGAAACCAAAGCTAGGACAAGCAGTAAATATTGCAAAACAAGCTTTACAAGAAGCG
ATCAATTTAAAGCTAGCTAATTTTGAAGAACAAGAATTAAATGAAAAACTAGCTCAGGAAAAGATTGATA
TAACACTAAGTGGAGTAGGGCAAAATCAAGGCTCACTTCATCCTGTAACAAAAACACTTAATCGAATTGA
AGCATTTTTTAAACAGAATGGTTTTGCTATTGAATTTGGTCCTGAGATTGAAAGTGATTATTATAATTTT
GAAACTTTAAATATCCCTTCTCATCATCCAGCTCGCGCTATGCATGATACTTTTTATATTGATGAGACTC
ATGTTTTGAGAACGCACACCTCTGGAGTGCAAATTAGAACTATGGAAAAACAACAGCCACCAATTAGAAT
AATTGCGCCAGGTAGGGTTTATCGTTGTGATTCGGATATTACTCATACACCGATGTTTCATCAAGTAGAA
GGTTTACTTGTAGATAAGGATGTTTCTTTTGCTGATCTTAAAGGCTTGTTGCATGCTTTTCTTAACTCAT
TTTTTGAAAAAGATCTAAAAGTAAGATTTAGACCATCTTATTTCCCATTTACTGAGCCTTCAGCAGAGGC
TGATATCGAATGTGTAATGTGTGATGGTAAAGGCTGTAGAGTATGTAAGCATACTGGTTGGTTAGAAGTA
CTTGGCTGTGGTATGGTGCATCCTAAAGTTTTAAAAGCTGGAAATATAGATTCTGAAAAATATCAAGGAT
TTGCTTTTGGCATGGGTGTTGAAAGATTATCTATGCTTAGATATGGTATAGATGATCTAAGAATGTTTTT
TGAAAATGATTTGAGATTTTTAAAACAGTTTTAA

>lcl|NC_006570.2_cds_YP_169998.1_884 [gene=ruvB] [protein=Holliday junction DNA helicase RuvB] [protein_id=YP_169998.1] [location=complement(1023158..1024204)]
ATGATCGAAACAGATAGAATAATCTCAGCTAATACAGCTCAAACCAATGATGAAAATGTTATAGATAGAG
CTATTAGACCAAAAACTTTGGCAGAGTATGAGGGTCAACCAGCTGTACGCGAACAGATGGAGATTTTTAT
TCAAGCAGCAAAAGCACGTAAAGATGCTCTTGATCATACACTTATATTTGGACCTCCTGGCTTAGGCAAA
ACAACCTTATCAAATATTATTGCCAATGAGATGGGGGTTGAGCTTAAGCAGACAAGTGGTCCAGTTCTAG
AAAAAGCTGGTGATTTAGCTGCACTTTTGACTAATCTTGAAGAGAATGATGTGTTATTTATTGATGAAAT
TCATCGCTTAAGTCCAGTTGTAGAAGAGATTCTCTATCCTGCTATGGAGGACTACCAACTTGATATAATG
ATAGGTGAAGGTCCAGCAGCGAGATCTATAAAAATTGATTTACCGCCGTTTACATTAGTTGGAGCAACTA
CAAGAGCAGGGCTTTTGACATCACCTTTGCGTGATAGATTTGGTATTATCCAGCGACTAGAGTTTTATTC
GATAGATGATTTGTCAAAAATTGTTTATCGTTCAGCAAAGTTATTAAATCTAGATATTACTACTGATGGC
GCAATGGAGATTGCAAAACGCTCAAGGGGAACACCTAGAATTGCTAATAGACTTTTGCGTAGAGTAAGGG
ACTACGCTCAAGTTAAAGGTTCTGGAGTAATCTGCTTTGAAATTGCTGATAAAGCTTTGAGTATGCTTAA
AGTCGATCCTGTTGGTTTTGATCATATGGATCATAGGTATTTGCTTACATTAATGGAAAAATTTGCTGGT
GGACCGGTGGGACTAGATACAATGTCAGCAGCTTTAAGTGAAGAAAAGGGTACCATTGAGGATGTTATTG
AACCTTATTTAATTCAACAGGGCTATATTATGCGTACAGCTAGAGGACGTATTGCGACTCTATTAGCATA
TAACCATTTCAAATTAAAAATCCCAGATAACTTAAGTGCAGATCAACAACAAACTCTATCAATATAA

>lcl|NC_006570.2_cds_YP_170003.1_889 [gene=dxs] [protein=1-deoxy-D-xylulose-5-phosphate synthase] [protein_id=YP_170003.1] [location=complement(1026713..1028560)]
ATGTCAAAATATACTATTTTAGATAAAATAAATACCCCATCTGATCTTAAGCTAATTCCTGAGAGTCAGC
TCAAAATCTTATCAGCAGAGTTAAGAGCTTTCTTAGTAGATACTTTAGATGTAAGCGGTGGGCATTTTGC
AAGTAGTCTTGGAGCTACAGAGTTAACAGTTGCTTTGCATTATGTTTATAATGCGCCATATGATAATATC
GTGTGGGATGTAGGGCATCAGACATATATTCATAAGATTCTTACTGGCAGAAAAGATAAACTTGTCACCA
TTAAAAAAGATGGCGGTATTTCAGGCTTTCCTAAACGCAGTGAGAGTGAATATGACACCTTTGGAGTTGG
CCACTCAAGTACTTCTATAAGCGCTGCTTTAGGTATGGCTATAGCAGACAGGCTACAAGGCAAATCTTCT
AATACTGTAGCTGTAATTGGAGATGGCGCTATAACTGGTGGTATGGCTTTTGAAGCCTTAAATCATGCTG
GCGGTATCAAAGAAGATATTCTTGTTATACTCAATGATAATGAAATGTCTATTTCTGATAATGTTGGCGG
ACTTTCAGCACATTTTAGTAAAATTATTTCCGGTGGTTTTTATAATTCTATACGTGAGAAAGGTAAGGAA
GTATTGAAAAATATTCCACCAATATTTGAGTTTGTCAAAAAGGTTGAAACTCAAACCAAGGGGATGTTTG
TACCAGCAAATTTCTTTGAAGATTTAGGCTTTTATTATGTTGGGCCTATCGATGGTCATGATGTTACAGA
ATTAGTTAAAACCCTAAGAATTCTAAAAGATCATAAGGGTCCAAAACTTTTACACGTGATTACTAAAAAA
GGTAAAGGTTATACAAAAGCTGAATCAGATCCAATTAAATTTCATCATGTTGCACCAAGTTTTCATAGTG
GCGAGAATATAACTACCAAGATTTCTAAACCAACTTATTCAAATATATTTGGCGATTGGATTTGTCAAAA
AGCTGCTAAAGATAAGCGTTTAGTTGGAATCACGCCAGCGATGAAAGAGGGTTCTGACTTAATTAGGTTT
TCACAACTATATCCGCATAGATATTTTGATGTAGCGATTGCTGAGCAACATGCAGTAACTTTTGCTGGTG
GCTTAGCTTGCCAAGGATTAAAGCCAGTCGTGGCAATATATTCAACATTCCTACAGCGAGCTTATGATCA
AGTAATACATGATATAGCTTTACAAAATTTAGATGTACTATATGCTGTAGATAGAGCAGGTCTAGTTGGT
GCTGATGGCGCAACTCATGATGGTAGCTTTGATTTAGCATTTATGCGCTGTATTCCTAACCATGTGATTA
TGACTCCAAGTGATGAAAATGAAGCTTATCATATGCTAGAGTTTGGCTATGAGTATAATGGTCCAGCAAT
GGTACGTTATCCACGTGGTGCTGGTATTGGCGCTGAAATTACTGGTAGTTTAGATCTAGAATTAGGTAAA
GCAAAAATTGTTAAACAAGGTTCAAAAATTGCAATTTTAAATTTTGGAACTTTATTGCCTCTAGCCAAAC
AGCTAGCTGAGAAGTATCATGCTACAGTTATAGATATGCGTTTTGTCAAACCACTTGATGAAATTATGCT
TGATAAAGTATCTCAAACTCACGAGATAATATTAACTTTAGAAGAGAATTGTATAGCTGGGGGCGCAGGT
TCTGCTGTTAATGAATATTTTGTAGCTAAAGATCTTAGCAATAAAATAATAGTTAGAAATTTTGGACTTC
AAGATAAATTTCTCAATCATGGTACAAAAGATTTATTATTAGCTCAAAGTAAGCTTTGTGTTGAAAATAT
ATCTCAAGAGTTAGATAAACTAATTTAA

>lcl|NC_006570.2_cds_YP_170004.1_890 [gene=guaA] [protein=GMP synthase] [protein_id=YP_170004.1] [location=complement(1028642..1030192)]
ATGACAGATATACATAATCATAAGATTTTGATTTTAGATTTTGGCTCGCAATATACACAACTTATTGCTC
GTAGAGTAAGAGAAGTAGGTGTTTTTTGTGAAATTTTCCCTCATGATGTAGCAGCTGATTTTATCAAAAA
TTATCAAGCAAAAGGAATAATTTTATCAGGTGGTCCTGAGTCAGTTTATGATTCTGATGTCAAAGCTCCT
GAGATAGTTTTTGAGCTTGGAGTACCTGTTTTGGGTATTTGTTATGGCATGCAAACAATGGTAATGCAGC
ATGGTGGCGAAGTAAAAGGAGCTGATCAAAGTGAATTTGGTAAGGCAATTATAAATATCCTAAATTCAAC
AAATAACATATTTTCAAATATGGAGCATGAGCAGCTAGTTTGGATGAGTCATAGTGACAAAGTCACTCAA
ACTGGTGAGCATTTTGAGATAATTGCTTCTTCAACAAATGCTCCAGTTGCGGCAGTTGCACATAAAAATA
AGCCTTTTTTTGGTGTGCAGTTTCACCCAGAAACTACTCATACAGAAAATGGTAAGCAAATTATTGAGAA
TTTTGTCGTTAATATCTGCGGATGTGATACTCTGTGGAATATCGAAAACATTATCGAAAATGATATAAAA
GAAATTAAACAAAAAGTTGGTACTGATAAAGTAATTTTAGGGCTATCTGGAGGTGTTGATTCATCTGTTG
TAGCTGCGATATTACATCAAGCTATCGGCGATCAGCTAACTTGTATATTTGTAGATACTGGTTTACTGCG
CCTTAATGAGGGTGATCAAGTTATGCAAGTATTTGCAGAGCATATGGATATTAATGTTATTCGTATCAAT

GCAAAAAATAGATTCTTAGATGCCCTAAGGGGTATCTGTGATCCAGAACAAAAACGTAAAATTATCGGTA
AGCTATTTGTTGATATTTTTGATGAAGAGGCTGCTAAGATAGAGAATGCTAAATGGCTTGCGCAGGGCAC
TATCTACAGTGATGTAATTGAATCAGCTGGCAATAATCAGTCTAAAGCGCATGTAATTAAATCACATCAT
AATGTTGGTGGCTTACCAAAAGAAATGAAACTTAAGCTTTTAGAACCATTAAGAGAGCTTTTCAAAGATG
AGGTTCGTAAATTAGGCTTAGGTTTAGGATTACCTTATAATATGCTTTATAGACATCCATTTCCTGGTCC
TGGTCTTGGAGTACGTATATTAGGTGAAATAAAGAAAGAGTATGTAGAAACGTTACAAAAAGCTGATGCT
ATATTTACAGAAGAGCTCTATAAACATAATTTATACCATGATGTTTCTCAAGCTTTTGGGGTGTTTTTAC
CTGTCAAATCGGTAGGTGTAGTTGGTGATCAGCGTAGATATGAGTATGTAATAGCTCTAAGAGCTGTCGT
TAGTATCGATTTTATGACTGCAACGTGGGCTAATTTACCTTATGATTTTCTGTCACTAGTTTCAAATAGA
ATTGTAAATGAAGTAAAACAAGTATCAAGAGTTGTGTATGATGTGACAGGAAAACCACCTGGAACAATTG
AATGGGAATAA

>lcl|NC_006570.2_cds_YP_170006.1_892 [gene=truA] [protein=tRNA pseudouridine synthase A] [protein_id=YP_170006.1] [location=complement(1031880..1032656)]
ATGAAAAACTACCTACTACAAATCGAATACTTTGGCAAAAACTACTGTGGTTGGCAAAGACAATCTCATT
CACCAAGTGTGCAAGAAGAGCTTGAAAAAGCACTTTCAAAAATAGCAAATCAAAATATAGAAGTTACTTG
TGCAGGGCGAACTGATACAGGCGTGCATGCAACTTCACAGATTGTTAATTTTTATTCTAATGCAGATAGG
CCTTTAAGCGCATGGCAGCGTGGGGTAAATGCTTTGCTACCTCAAGATATTAAAATATTAGCAGTTCAAC
AAGTTGATAATAATTTTAATTCTAGGTTTACAGCTATAAATAGAACTTACAATTATATTATCTACAATTC
TGCAACTAGCTCACCAATATTCGCGGAACATTGCTTATGGGAGAATAGAGAACTTGATATAGATAAGATG
AATCAAGCTTGTGAATATCTTCTTGGCGAACAAGATTTTAGTTCTTTTAGATCATCACAATGTCAGTCAA
ATACACCATTTAGAAATATCCAAAAAGCTGAGTTTATCAAGCAGGGTAGTTTTATTGTTTTTGAGGTTGT
TGGTAATGCTTTTTTACATCATATGATTAGAAATTTAGTAGGCTCATTATTAAAAGTAGGCTTAGGTTTT
GAATCTCCAGAATGGATTAAAGTAGTATTAGAAGCTAAAGATAGAACACAAGCAGCTGAGACAGCTAAAG
CACATGGTTTGTATTTTGTCGGTGTTGAATATCCGGAATTTAGCTTCAAACGGCAAATAATTAAATTGTT
TTGTTAG

>lcl|NC_006570.2_cds_YP_170009.1_895 [gene=yhbG] [protein=ABC transporter ATP-binding protein] [protein_id=YP_170009.1] [location=complement(1035146..1035877)]
ATGGAAAGATACACATTAGAAGCTAAAAGATTAGGCAAAAAATATGGTTCACGCTGGGTTGTGAATAATG
TCTCTATGAAAGTTTCAACTGGTGAAATTGTTGGTCTTTTGGGCCCAAATGGTGCCGGAAAGACAACATC
ATTCTATATGATTGTAGGCTTAGTTGCAGCTACACGTGGTAAAGTACGCATGGGTCAAGAGGATGTAACA
AAAATGCCGATTCACCTGAGAGCTAGAAGAGGATTAGGCTATTTGCCTCAGGAAGCTTCGGTTTTTAGAA
AGCTAAGTGTCGAGGACAATATTGTCGCAATTTTAGAAACTCGTAAAGATCTAAATAAAATTCAAATCGA
AGAAAAACTCAATGAGCTTTTAGACGAATTTAGTATACAGCATATTCGTAAAAGTTTAGGTATGAGTTTA
TCTGGTGGTGAGCGTAGAAGAGTTGAGATTGCTCGAGCATTAGCTATGGATCCTAAGTTTATCCTACTTG
ATGAGCCTTTTGCAGGGGTTGACCCAGTTTCGGTGATAGAGATTAAAGAGGTTGTCCGTCATCTTAAAGA
TAGAGGTATAGGTGTTTTGATTACAGATCATAACGTACGCGAGACGCTAGATATTTGTGAAAGAGCTTAT
ATTGTAAATGCTGGTAATATGCTTGCAGCGGGAACTCCAGAAGAAGTTCTAGCCGATGAAACAGTTAGAA
AAGTATATCTAGGTGAAGATTTCAAATTATAA

>lcl|NC_006570.2_cds_YP_170010.1_896 [gene=FTT_1025c] [protein=hypothetical protein] [protein_id=YP_170010.1] [location=complement(1035951..1036784)]
ATGCGACAAGCTAGGCTATTTATTATAATTTCTCTTTTAATTGCGTCAAACGCGTATTCAAATATCGAAG
AAAATAAGATTGATTATAGCTCTCCTATATATAACTCTTCTATACAGGAAGATAATGCAACTGATGAGGA
AAAAGAGAATAACTCCGATGATAATAACCTAAAAGAGTATGGTCCTGTAACTATTTGTGCAAATAATGCT
GTTTATGACGATAATAAAGGAGTACTAACTTATTTAGGCAATGTATTTGTAATGCAAATTCATAATAAGC
ATATTTTATGTCATCAGCCAAATAACTTAAAAAAAGGTGTAAGTTATTTCATAAGAGACAATACTTTACC
ATTTAAACAACTACAACAAAAATGGTTAGAGCAAGCAAAATTATTATGTTCTCAAGAACAAGAGTGTAAT
TTCATCTCTGGACAAAAGCTAATTATAAATTTAGACAAAGATAGAAAAATAAAGACTTTCACAATGCTTT
CAGAAGGTGATGAAAAATCACGATTCTATACATTTCCGACTAGTACTAATCCTAACTATAGTAGTTCAAA
AACTGTAACTAGAGGGCCTGTTGAAGGAAGTTCTAAAAAGATTGTTTATGATGTTACTGATAAGCATTTA
GAGCTTTATAAGAAAGCTATAGCTTATCAAAATGACAATGTATATCGTGGCGAAAAGGTGATTTTTGATA
TAACTCATGACTTAATATCCATACCTGGTAGTGTCGATAGAAGGTCAACAATAATATTGGATGGTCTTCA
AAACCAAACAAAAATTGATACTGGCCTTACACCTATTAGTCAGTACAAAAATAAACCAAATTAA

>lcl|NC_006570.2_cds_YP_170011.1_897 [gene=FTT_1026c] [protein=hypothetical protein] [protein_id=YP_170011.1] [location=complement(1036771..1037397)]
ATGAAGTTTTTTACAAAATATTCCTTATTTGCGAATGTACTCTCTATAATTGTAATTATTTTTTCAATGC
TGTATATAAGCTATAATGCTCTAGATGGCGGTAAACCGCTCAAAAATATACCACAAAAAAATCGTGTTGA
ATTAAAAGCCTTTGATTTTAATTACAATAAATATGACTCAAGTGGTAACCTTGCAATGAGTTTTTTTGCT
AAAGAGCTGCAACGTTATCTTAATCAAGATTTGTATATGACAGATATTACCGAAAAAAGCTATGATAAAG
CCACAGAAAAGCTTGATTGGCAAGTACAAGCAAAACATGCTCAGCAATTAGCAAATCAGAATCTGATACA
CTTATATGATGGTGTTAATGCTATTATGATTACAAAAAAATCAGCAGCTAATACTCAAAAGACTTCTGAT

AATGATTCAACACCAGATAAGATCTATATAAAAAGCTCTGAAATGTTTTATAATTCAAGTTCTAAAGATT
TTTATAATAATAGATTTACTAAAATGTATGATCCTAAAACTGGTAACAACACCACAGGAACTGGTGTAAA
GGGAAACTCTGAGACTAAAATTATAGAATTAAGCCAAAATGTAAGGAGCTATTATGCGACAAGCTAG

>lcl|NC_006570.2_cds_YP_170013.1_899 [gene=ppa] [protein=inorganic pyrophosphatase] [protein_id=YP_170013.1] [location=complement(1038044..1038565)]
ATGCTAAAGAATATACCTTGTGGAAAAGATATTCCTAATGATTTTAATGTTGTAATAGAAATACCTCAAG
ATAGCGATCCTATAAAATATGAATTTGATAAAAATAGCAACATGATAGTTGTTGATAGGTTTATGTCATC
TACTATGAGATATCCTTGTAATTATGGTTTTGTGCCAAATACTCTTTATGATGATGGAGATCCTATCGAT
GTATTGGTTTTAGCGCCATATCCTTTAGCAGTTGGTTGTGTAATAAACTGTAGAGCAGTTGGTGTGTTTA
AAATGGAAGATGATGGTGGTGTTGATGCTAAAGTTATTGCAGTACCTAGCTCTAAGTTAACTAAAGAATA
TGATCATATTAACGATGTTGATGATTTGCCGGTATCTTTAAAGCAAAAAATTGAGCATTTCTTTACACAT
TACAAAGATTTAGACTCAGGTAAGTGGGTTAAAGTAGAAGGCTGGGATAATGCTGCCTTTGCTAGAAAAG
AAATCGAAAAATCTGTAAAAAACTACAAATAG

>lcl|NC_006570.2_cds_YP_170014.1_900 [gene=dacD] [protein=D-alanyl-D-alanine carboxypeptidase] [protein_id=YP_170014.1] [location=1038722..1040056]
ATGAAATTGATAAAAATAGCTTTATTAACAGCTAGTATAACAGCGGGGGTAGCTTTTGCTGCGCCTAATA
TATCTGCCTACTCTGATCCTTATTTCAATGGAGCTAATGGACTCGCGCAGAAAGATATTATTATAAGACC
TGCAAACATTGAGCTAGATGCTCCAGCTTGGGTAACGATGGATTATCGTACTGGAGATATTGTTAGTGAA
AAAAATATGGACGTCAGAAGAGCCCCTGCAAGCTTAACTAAAATCATGACTTCCTATATAGTTGCTAGTG
AAATAAAAGCTGGTAATCTAAGCTGGGATACAATGATCCCAATTAGTGAAAATGCTGCTTCTACAGGTGG
TTCAAAGATGTATGTTAAAGCCGGTGCTAAGGTATCTGTAAGAAATCTTGTAACTGGTATGGATGTAGTG
TCTGGTAATGATGCTACTATTGCTCTTGCTGAATATATCGGCGGAACTACACAAGCCTTTACTGATTTAA
TGAATCAAACAGCAAAGGCTATTGGCATGAATAATACTCATTTTGCTAATCCAGATGGACTTCCAGGTGG
TGAGCAATATACTACTGCTCATGATATGGCGTTGCTTGCAAGATCATACATATATAATTTCCCAGAAGCA
TATAAAGTATACGACGATAAGGGCTTAGTTTGGAATGCCACAAAACAAGACTCGGTAAGTATTGCTGATC
GTAAACAGTGCTTACCTAAGTTTGATCGCGCTACTGGAAATGTCATAGAGAGCTATACGGTCAAAGATCT
TGATGATCAAGCTAAAGATAAATGTAATAAGCTTTTTCCTAAAGGTGATAATTTCGTATTACAAAATAAT
AGAAATAGATTACTATTTACATTTGATGGGGCTGATGGTATGAAGACTGGTCATACTGATGCTGCTGGAT
ACTGTCTAGTTTCATCTGCTAAACAAGATGGCGAGAGATTTATTTCTGTAGTTCTTGGTACTACTAGTTC
TGCTAAGAGAGATTCAGAATCAGCTAAATTACTAAGATATGCTCTGAGTAAGTATGAAAATGTACTTTTA
TACAAAGCAAATTCTCCAGTTACAATTAGTGCTGATAATATCCCTAATGCAAAAGCTGGACAAAAGTTAA
CTGTAGCTTCAAATCAAAATATTTATAAAACAGTACCTAAAACTTATGTCCCTTATCTAAAGCAAGGTAT
AGAATTTAATCCAAATTTGAACGCTCCTATTAAAACAGGTCAAACTGTAGGTAATTTAGTGATTACACTA
GGTGATACTAAGGAGGAAATTGCTAGTATACCTGTAGTTGCTATGAATAATGTTTCACAAAAAGGTTGGT
GGTAA

>lcl|NC_006570.2_cds_YP_170015.1_901 [gene=FTT_1030] [protein=hypothetical protein] [protein_id=YP_170015.1] [location=1040089..1040373]
ATGTCTGAAAGTAATAATCACAATCAACAAGAAACTTTTTTTGAGTTTCCTTGCCAATTTCCAATAAAAA
TAATGGCAAATCCTCAGAAAGAAACTGTTGAGTTTATTCTAAGTGTTTTTGAGAAATATATACCAAATCA
TAGTGAAATAGACTTTAATACCAAAGAAAGTAAAACTGGTAAATATATTTCTATAACTGCGATTTTTACC
GCAGATAGTAAAGAACAGCTAGATAATATCTACAAAGAAATTTCAGCACATCCAGAAGTTCACATGGTTT
TATAA

>lcl|NC_006570.2_cds_YP_170016.1_902 [gene=lipB] [protein=lipoate-protein ligase B] [protein_id=YP_170016.1] [location=1040383..1041003]
ATGAATAATATTTACCAAAAAGACTTGGGTTTACAACAATATACCAAAGTGTTTGATGATATGCTTGAGT
TTACTTCAACACGAACTCCTGAAACTAACGATGAAATATGGCTAGTTGAACACCCTGCTGTTTTTACTCA
AGGTAAACATGGTAAGCCAGAACATATTCTAAATTCGCATAATATCCCAATAGTTGCAACTGATCGTGGT
GGTCAGGTTACTTATCATGGTCCAGGTCAAGCAGTGATATATTTTCTTCTTGATATTAAGAGAAATAAGC
TTGGTGCCAAAAAGTTAGTAACAACTGTTGAGCAAGCTTGTATCAATATGCTTGATAAATATTATAATCT
AAAGGCTCATATTATTGATGGAGCTCATGGTATATATATAAACAATCAAAAAATTGCCTCTTTAGGCTTA
AGAATCAAACAAGGTAAAAGCTATCACGGTATTGCTATAAATACTAATATGGATCTTACACCATTTAGCT
ACATTAATCCCTGTGGTTATAGTGGTTTAAAAATGTGTCAATTAGCTAATTTCTATCAAGAAGCTGATAT
TAAGAAAGTTCAGCAGCAATATACTGCTGAGTTTGTAACTTTGCTAAATAACTCTATCTGA

>lcl|NC_006570.2_cds_YP_170018.1_904 [gene=rpoD] [protein=RNA polymerase sigma-70 factor] [protein_id=YP_170018.1] [location=complement(1046441..1048174)]
ATGACAAAAGAAGATTTGCTCTCTGATTTAAAAGATTTGATGATTGATGGTAGGGAAAGAGGATATTTAA
CTAGAGCCGATATTTTAGATGCTCTACCAGGAGATGTTTCAGAAGATCCTAAAATATATGAAGAAATTGA
GGCTATACTTATTGACGCTGGTATTGATGTCTATGATAGAACTCCTCAGATAGAAGAAGATGATGAAAGA
AAGGTTAGTGAAGCTAATCTTGATGATTTAAAAGGTAAAACTTCAGATCCTATTCGTATGTATATGCGCG

AAATGGGTATTGTTGATCTTTTAGATAAAAAAGGCGAGACGGATATCGCTATTAGGATCGAAGAAGGTAC
TACAGAAGTTTTTAGTACGATTTTAAGCTATCCGATAGTAATTAAAACTTATATTGAGCGTTTCCGTGAG
CTAGAAGAAAAAGCTATAGATTATATGAATTCCCAAGATATTGAGAATGAGCCTGTAGCTAGATATATTC
GTTTTAATGAAGTTATGGCTGGTTTTAGTGATGAACAGGTAACTGAAGAAAAGGTTGCTGAAGATGATCA
TGAGGAAAAAATTGATACTCAAAGGGCTTATGAATTCTTTACAAATTTAGAGAAAATGTTTGAAGAATAT
CAAGATAAGCCTAACAAAAAACTTTATACTAAAATAGTTAAGGAATTTGATAATTTAAGATTGTCAACAT
CACATTTACAAAAGTTGGTTGATTATATTAGATTACCTTATGCTCGAGTTAAAGAATTCGAAAGAAAAAT
TTTAAGACTCTGTGTTGAAAGATCAAAAACACCACGCCAAGAGTTTATTAAAGTTTATAAAGTTGGTTCT
CTAGAATGGTTGGAGCCATTAACTAAAAAGTATAAATTTACTGAGAATACTATTAGAGAAATAAAAAATT
TAACTAAGCAAATTAATCAATTCCAAATTTTAATGATGATGGATATTGAAGAGCTTAAACAAGTCAATCT
TGAGATTTCAAGGAGTGAAGCTAAAATTACTCAAGCTAAAAAAGAGATGATAGAAGCAAACTTAAGACTA
GTTGTTTCTGAAGCTAAAAAGTATACCAATAGAGGCTTACATTTCTTAGATATTATCCAAGAAGGAAATA
TTGGCTTAATGAAAGCTGTAGATAAGTTTGATTATCGTAAAGGCTTTAAGTTCTCGACATATGCTACTTG
GTGGATACGTCAAGCAATTACTCGTTCGATTGCAGACCAAGCTAGAACTATTCGTGTACCTGTACATATG
ATTGAGACAATTAATAAAGTAAATAGAATTAAACGTCAGATTCTGCAAGAAAAAGGGCGTGAAGCTACAG
AAGAGGAGATTATCGAGCATACTCCTAATATGACTAAAGAGAAGCTGAAAAAGATTCTTAATATTTCACA
TACACCAATTTCAATGGAGAGCCCAATTGGTGATGATGAAGATTCTACAGTGGGCGATTTTATTGAGGAT
AAAAGTAACTATTCTCCGATAGAGGCAGCTAATTTAGAAAATTTACGCGAAGCAATAAAGGAGCTTATTG
AAACTGGCTTAACTGAGAGAGAGGCTAAAGTTCTAATGATGCGTTTTGGTATTGGTATGAATACTGATCA
TACTTTAGAAGAAGTAGGTAAGCAGTTTAATGTTACTAGAGAACGTATTAGACAAATTGAGGCTAAGGCT
CTTAGAAAGCTTAAACATCCTTCTAGATCGGCATTTTTGAAAACATTTTTGTAA

>lcl|NC_006570.2_cds_YP_170019.1_905 [gene=dnaG] [protein=DNA primase] [protein_id=YP_170019.1] [location=complement(1048194..1050011)]
ATGGCGAAAAAAGTCTCGAATAGTTTTATAAAAGAGCTTGTAGCTACAGCTGATATCGTTGATGTTGTTT
CAAGATATGTTAACTTGAAAAAAACAGGCAAAAACTATAAAGGTTGTTGTCCTTTTCATAATGAAAAAAC
ACCATCATTTTTTGTTAATCCAGAGAAAAATTTTTATCATTGTTTTGGCTGTCAGGCATCTGGAGATGCA
TTAACTTTTGTTAAAAATATTAATAAACTTGAGTTTATTGATGCTGTAAAAAACCTTGCTGAAATAGTTG
GTAAGCCAGTTGAATATGAAAATTATTCTCAAGAAGATATCCAAAAAGAACAGCTATACAACAAATGTAT
AAGCTTTTTAGCAGCTGCTCAAAAGTATTATCGTTGGAATTTGGGTAACTCTGTCACCAAAGATAAGGCT
ATAAACTATCTTAAAAAAAGAGGTATAGATAGTAGTTTGGCTAAATTCTTTGGTATAGGATATTCATCCG
AAGGTTGGAATAATATTACTGAACTAGTAAAGTCTATAAATATTCCAGAAGAAATATTAGTTGATACTGG
GCTTGCTATAAAAAAATGATAAGGGCAATTTATATGATCGTTTTCGCGGTCGTGTTATGTTCCCTATTAGA
AATATTCAAGGCAATGTAATTGCTTATGGCGGTAGAGTAACAGAGGACTCTGATGGAGTTAAGTATATAA
ATTCACCAGAAACTTTAGTCTTTCAGAAAAATAATATTTTATATGGACTCTATGAGTACCGTGAAAGAAA
AAAACAATATCCAGATTTAATAAATCAAAGCCTTGTAGTAGTTGAAGGTTATATGGATGTGGTTGGTTTA
GCACAGCATGGTTTTTATGCTGCAGTAGCTACATTAGGCACCGCATTCTCACCAAATCACGCTAAGATTT
TATTTCGTGAAACTAGCTCAGTAATTTTGTGTTTTGATGGTGATGAAGCAGGGCAAAAAGCAGCACTTAG
AACTATAAAGATTTTGTTACCAATGCTAGATGGTAATAAAAAACTAAAAATTTTAACTCTTCCAGATAAG
GATGATCCTGATGATTATATTAAGAAATATGGTTTAGAAAGATTTCTCACTGCTTTAGATAATTCTCTAG
CTGTAGCAGATTTTGTAATTGATAATCTTATTCAAGGTAAGGATCTGCGTAAAGCAGAAGCAAAAGCGGA
AGTTTTAGAGAATTTAAAAAATTTTTTAGCAGATGTAGAAGATAATATTTATTCAGAAAGTATTACTGCT
ACTATAGCTGATAAAATTGGTATAAAAGTTGAGCAATTTAAGAATCTACTTAAGATACGTAAGCAAACTA
CACTAAACGTAAATAGACAGCAAAATATTCAGCAAAAGAAACTAGCAAAGAATCTATTGCTTGAAGAGTT
TGTCCTTGCGGAATTATTTGTTAATATCGCGGATTTTCGCATTTTACAACATACAAACGATTTTGAAATA
TTTGCTACTTCGAAAAATCTTGATATCTTAGCAAAAAGCTTGAAAATTTTAAAAGAAGACTCATCTAATC
AAATTGAAGCTGTTATACTTATACAGCTATTGGCAGAGGACTATCCTGACTATAGAGAGTATTTTTTTGA
GTTGTTAAGTTATGGTATTCGCAATACTCAAAAAAAAATATGCTGAAGACAAATATCAAGAACAAATGTTC
GTTATGCTAAAAAGAGTAGAAAACTCTAGTGTAAAAAAAAGATTAAAATATTTAGGTTCATTGTCCTTTA
GGTCGGATGTCCAGGAAATGGAGCGTAAGTATTTAGTAGCAAAATTAGGCAACAGTAATATAATATAA

>lcl|NC_006570.2_cds_YP_170021.1_907 [gene=rpsU] [protein=30S ribosomal protein S21] [protein_id=YP_170021.1] [location=complement(1050539..1050736)]
ATGCCAAGCGTTAGAATTAAAGAAAGAGAACCTTTTGATGTCGCTCTTAGAAGATTTAAAAGATCTTGCG
AAAAGGCTGGAATAGTATCTGAACTACGTCGTAGAGAATACTTTGAAAAGCCAACTTGGGCACGCAAAAG
AAAAAAAACTGCCGCTGTTAAAAGAGCTCATAAGAGCAATATAATAGTTAAAAGGTAG

>lcl|NC_006570.2_cds_YP_170022.1_908 [gene=dacB1] [protein=D-alanyl-D-alanine carboxypeptidase] [protein_id=YP_170022.1] [location=1050992..1052395]
TTGAAAAGAAAAACTAAGTTTTTTACGGCATTCTCTATATTACTTATAAGCATAGCAACTACTCAAGCAT
CTACGCGTAGTGAGGTTCAATATCTTGTAAAAAAATATAATCTCTCTGATGCTAAAATTGCTATAGCAAC
TCAAACAACTAATAATGGTGATCAATTATATGCCTATGCCCAAAATAGATTAATGACGCCAGCAAGTACT
AATAAGGTTTTTACAATAGTTGCGGCACTTTTTACGATACCTAGTAATTTTAGATTTACAACATCAATAA
TGTACCCTTCCGATAGAGTAAAAGATCATACTCTGTATGGAGATATGTACATAAAATTTACTGGTGATCC

AGCACTAACTGGTAGTCAGTTAGCCACATTAATAAAAAAAATTAAAACTGAGAAAGATATATCTAAAATA
ACTGGTGATGTTTACCTTGTTGGAGTTTTCTCTGGGCCTTATATCCCAAATGGTTGGTCTAAAGAAGATA
GTACATTCTGTTTTGGTGCTCCAGCATCAAGCTTCACACTTAATAGAAATTGTACAGTCATAAAATTAGT
AAAAAATACTAATAGTTTAACAACAAGAATTGTTGAATTAAGTAACGCAAGTAATATCACTATAAAAAAT
ACCGCAAAATATACTAGTGCATCAAGTGCAACTACGATAGAAATGAATAATGATAATGTTTTATATATTG
GTGGATACTTATCAAGAGCTGCTGAAAAAATGTTTAAATTAGCAATTAAAAATCCCGCGCTTAAAACATC
AGATACCGTAAATGACTTCTTAAATTCTGACGGTATTAAACATGGTAACGTTATTATAGCTGGTAGTGTT
CCTACTGGTTATACTGAACAGATTACAACAAGGTCTCAAACAATCGGACATTTTATTGATCAAGCCCTTA
AACATTCTAATAATCTATATGCAGAGACTATTTTAAATACTTTAGGCTTAAAAGAAAAAGGTATTGGCTC
AACTAAAGCAGGTACGGAAGCTGTTCAAAGTATACTCTACTCAAAACTAGGCTTGGATACATCAGCTTTA
ACAATGTATGACGGTTCAGGTCTATCACATCTAGATAAAGTAACTCCAGAATTTATGGTCAACTTTTTGA
CCAAAGCTTATAATAGTCAGATTGGTAAAGAATTCTATAACTACTTATCAGCATCTGGTATAAGCGGTAC
AATTTCATATAGAATGGGTGGTAAACTACTTGGACGAGTACATGCTAAAACTGGTACTCTATCCGGAGTA
TCAACGTTATCAGGCTATTTATTAACAGCTAAAAACCACAGAATCAGTTTTTCGATTATGCTTAATAATC
TTAAACCCTCTGATCGTTATAATGCACGAAGATTCCAAGATAAACTAGTTGATGTCTTCTATAGGAATTT
ATAA

>lcl|NC_006570.2_cds_YP_170027.1_913 [gene=ispB] [protein=octaprenyl-diphosphate synthase] [protein_id=YP_170027.1] [location=complement(1055079..1056056)]
ATGCAACAGCTAAAAGATATTCAGGCGCTTATTAAAGAGGATATACAAAATAATAATCAATTCATAGTTG
ATTCTCTTTGCTCAGATGTCGTACTTATCAATCAGATTAGTCACTATATAATAAATAGTGGTGGTAAAAG
ATTAAGACCTCTGTTAGTAATGTTATTTGCTAGAGCCTTAAACTATAATGGAGATAAACATTTAGCATGT
GCTGCAATTATTGAGTTTATTCATACAGCAACTCTTCTTCATGATGATGTTGTTGATGATTCGCAATTGC
GTCGTGGTAAACAAACAGCAAATAATGTCTTTGGTAATGCTGCGAGTGTTTTGACTGGAGATTTTCTCTA
TTCTAGAGCTTTTCAGATGATGGTAAGTTTAGATAATATGCAAATTATGCAAATATTAGCTGATGCAACT
AATAAAATCTCAGAAGGAGAGGTGCTACAACTACTAAATGCGCGTAATAGCGAACTTAGTGAAGAAGAGT
ATATTAAAGTGATATATTGCAAAACAGCAAAATTATTTGAAGCTGCTTGTGAATTAGCAGGTGTAATTAG
TCTAGATAAACAAGATTATACTAAGTACCAAGGTAGGATTAAAAACTATGGTGTTTATTTAGGTAATGCT
TTTCAGATTGCAGATGATGTTTTAGATTATGTATCTGATGCAGAAAGTTTAGGTAAAAAATATTGGTGATG
ATCTTGATGAAGGCAAAATGACTTTGCCAACAATCTACGCACTAGCTAATGTTACTGAACAACAGCAACA
AATTCTAAAAAAAGCTATTGAAAAGGGCGAGTATAGAATTGATGAAATCATAGCTATGGTCAAAGATAGT
GGTGCTGTAGATTATTCTTATAAAGTGGCTTGTCAGTATGCTGATCTCGCTAAACAACAAATAGATTTTT
TACCAGATTCAGAATATAAGCAAGCTATGATTTTATTATGTGATCTAGCAGTAAATAGGAAGAATTAA

>lcl|NC_006570.2_cds_YP_170033.1_919 [gene=FTT_1051c] [protein=hypothetical protein] [protein_id=YP_170033.1] [location=complement(1061830..1061994)]
ATGAACGAGATGAAGATCAAAGCTGCGAGTGTTTGCAATGATTCAAAAACCATAAAAAAAACATGGCAGA
CACCTATGTTATCGACACAACACCAGGAAAGTGAAAGCATTGAAGGAAAAGCCCCAGCTGGCAATGAACA
ATTTGGTACGCAAGGTCTGAGTTAA

>lcl|NC_006570.2_cds_YP_170036.1_922 [gene=FTT_1055c] [protein=hypothetical protein] [protein_id=YP_170036.1] [location=complement(1064854..1065279)]
ATGAGCTTTGCAAGCCAGATAGAAGAAATTTTTGGTGTAGATATTTGGGAATTAAAACCACAATATAAAA
CTTCGCAACAAAATCAAACTACTAATGATGCTCAACAACAAAATGAAATTATTACAGCAGATCTAAATGA
TTTAGAACTTATCTATACAAACGAAATTACAAGTTCTAAAATTATAAATATTCTTATATCTACTAAATTG
AATTTAACTTTTCTAAAAAATATTGCTAATAGTCTATTTTTTAATTCAAAAGTTAGTATTTATAAATCAA
ATGATATTAGTTCTTTTGAAAAACTTGGAGGTATTAATCTCAATGAAAAAGATCTGTTTACTAACAATAT
TGACCTATTAAGTATTCAAAATAAGAAATATATATTATCAAAGTTATATAAGTATGCAGATTTCTCTTCT
AGATAA

>lcl|NC_006570.2_cds_YP_170037.1_923 [gene=rluB] [protein=23S rRNA pseudouridylate synthase B] [protein_id=YP_170037.1] [location=complement(1065279..1066094)]
ATGCGTAGAGCAAATCAAAATAATGATAAAAATCCTGAAAGATTACAAAAGCTTTTAGCAAAATATGGTA
TTGGATCTCGAAGAAAAATAGAAGAGTATATAGAGCAAGGTAGAGTTAAGGTAAATGGTAAAGTCGCTAC
GCTAGGAGATAAAGCTAGTGAAGCTGACAAAATCAGTTTCGATGGTAAAGCTCTACACTCATATGGTCAG
CCAATGACTAGACCACGAGTAGTCATTTATCATAAAAGAGAAGGTGAAGTTTGTACTAGCAAAGATGAGA
AAGACCGCCAAACAGTCTTTGATTCATTACCAAAATTAGCAAAGTCACGTTGGATAATGGTAGGACGCTT
AGATATTAATACTACAGGTTTGTTGCTTTTTACTACAGATGGTGATTTGGCAAATAGATTGATGCATCCT
TCTTACCAAATTGAGAGAGAGTATGCAGTGCGTGTATTTGGTCAACAATTATCTGATGAAACTATAAATA
AGCTTAAAGAGGGCATACAGCTAGAGGATGGTATGGCTAAGTTTAATAGTATAAAATTCTCAGGTGGTGA
GGGTGCTAATCTTTGGTATTATGTGACACTTTCTGAAGGACGTAATCGAGAAGTAAGAAGAATGTTTGAA
GCAGTAGGTGTTACTGTTAGTCGACTAACGAGAATTAGATTTGGTGATATAATCCTTCCTAAATTTGTTT
CGCGTGGCAAAACATTAGAACTAAATCCATCAGAAGTTAATCGACTTAGAAAGTCTGTTAAGTTAAAAGA
GTATAGTTTTCCTAAGAAATTAGTTGAAAGGTTAGAGAAAAAATAA

>lcl|NC_006570.2_cds_YP_170040.1_926 [gene=dnaB] [protein=replicative DNA helicase] [protein_id=YP_170040.1] [location=complement(1068125..1069522)]
TTGATGTCTATGGATTATCAGTTTAAAGCTGCTGAAACGACTTACTCTCTAGAAGCAGAAAAAGCTATCT
TAGGGAATATATTACTGTACAATCAAAATATTGAGTTAGTTGAAGATTTTCTTTTGATTGATGATTTTTT
TGATAAAAGACATAAAACCATCTATAAACAGATTGTCACGCTTAACCAAGCAAACACTCCGTTTGATGTA
CTAATTTTAAGTGAATACCTTGCCACCGAGGGACTTTTAGAACAAGCAGGTGGTGAGGCTTACATCATAG
ATTTAGCTGCTAATACACCTTCAATATCAAATATCAAGACGTACGCTAATATCGTAAAAAATAAGGCTAA
GCTTAGAAGTTTACAAAATAGTGTAAATGATATCGTTCAGAAGATATATTCAGCAGATTCAAAAAATCCT
GATGAAGTTATTGATTACGCTGAGAGCAGAATACTTGATGTAGCTAAAGAGCGTGAAACACTTACCAAAG
GTCCTGAATCGATAAAGTCTGTAATCCCAAAGCTTGTAGATAGAATGAGTGCTATAGTTGATTCTGGTAG
TGGTCTAACAGGGCTTTCAACAGGTTTTATAGATCTTGATAAAATGACATCAGGATTACAAAGAGCAAAT
ATGGGTATCATAGCAGCTCGACCATCTATGGGTAAGACTGTTTTGGGTATTAACATCGCTCAAAATGTCG
CAAAAATTGCTGATAAGCCAGTATTAGTTTTTAGTCTAGAGATGCCGTCAGAAGATATTGTAACAAGGAT
GTTAGCTTCACAGGCGCGTGTAGAGATGAATTTATTTAAAGAATGTAATAGGTTAAATGATGCGCATTGG
GTTAAAATCACCAGCGCTATGAAAACTTTAAGTGAAATGCCACTATATATTGATGATACATCAAGTTTGA
CTCCAGCCGAGATGCGTTCAAGAGCGCGAAGATTATATAATGAGCATGGTGGTTTAGCAATGATCTTGAT
AGATTATCTACAGCTTATGAAAATCCCAGGTTATGAGACTAACCGAACACTAGAGGTATCAGAGATATCC
CGTTCACTGAAGGCTTTAGCCAAAGAGTTGGATATACCAGTCATAGCACTATCGCAACTAAATAGAGCAG
TAGATGATCGTAAAGATAAACGACCAATGATGTCAGACTTAAGAGAATCAGGAGCAATTGAACAAGATGC
TGACTTGATTATGTTTATTTATCGTGATGAAGTTTATAATAAAGATAAAGAAGATAATAAAAATCTAGGT
GAAATAATAATAGGTAAGCAGCGTAATGGTCCTATAGGAACTGTGCATGTGCGCTTTGATGGTCAGTTTG
CTAGTTTTGCTAATTTAACTAATGAAAATGATCATATTTTACCTGGTGATATAGGTTATAACGAGTAA

>lcl|NC_006570.2_cds_YP_170042.1_928 [gene=rpsR] [protein=30S ribosomal protein S18] [protein_id=YP_170042.1] [location=complement(1070084..1070302)]
ATGAGTCGTCGTAAAGTTTGCCGTTTCACTGTAGAAGGTGTGAAAGAAATAGATTATAAAGATGTTAATA
AGTTAAAAGCTTATATTACTGAAACTGGTAAAATCGTACCAAGTCGCGTAACTGGTACATCAGCTAAGTA
TCAAAGACAGCTAGCAACAGCTATCAAAAGAGCTAGATTCTTAGCATTATTACCATACTGTGATCGTCAC
TTTAACTAA

>lcl|NC_006570.2_cds_YP_170043.1_929 [gene=rpsF] [protein=30S ribosomal protein S6] [protein_id=YP_170043.1] [location=complement(1070318..1070653)]
ATGAAACATTATGAAGTCGTTTTAATGATCCACCCTGATCAATCAGATCAATTAGATGCAATGCTTGGCA
AATACCGTGGCATAATCGAAGAAAAAGGCGGCAAAATCCACAGATTTGAAGACTGGGGACGTCGTCAATT
AGCTTACCCTATCGAAAAACTTCACAAGGCACACTATGTACTATTTAATATTGAGTGTCCAACTGAATCT
CTAGAGAAGCTTCAAGAATCTTTAAGATACAACGATGCTATTTTACGTCGTTTAGTTATCGCTACAAAAG
AAGCTATAACAGAACCATCAGTAATGATGGAATCAAATGAGAAAGAAGTAATTTAA
>lcl|NC_006570.2_cds_YP_170044.1_930 [gene=hemF] [protein=coproporphyrinogen III oxidase] [protein_id=YP_170044.1] [location=1071331..1072257]
ATGCAAGAAAAAATTTCAAAATTTGAAGATTTTCTAACACAACTTCAACAAAATATTACTACAGCTCTAG
AGCAGCATGAAACAAATGCTGCAAAATTTATTTCTGACAAATGGCAAAAACCAGATACTCACGATCAAAA
ACTTAAAGGCTATGGTAACTCTATGATCATAGAAGGTGGCGAAATATTTGAAAAAGGTGTAGTAGCTTTT
TCTAGAGTCCACGGTAGTGAGCTACCACCATCTGCTACAGCAAAAAGACAAGAATTAGCTGGTAAATCTT
TTATAGCTACAGGGCTTTCATTAGTTATCCACCCCCGCAATCCTTTTGTGCCAACATCTCATGCTAATTT
TAGAATTTTTATTGCTGGTGCTGATACTGATAATCCAATATGGTGGTTTGGTGGAGGTTTTGATCTAACA
CCATACTATCCTTTTGAGGAGGATGCGATTCACTGGCATCAAACAGCAAAAAATATTTGTGACAAGCATG
ATAAAACCTATTATCCAAAATTCAAAAAATGGTGTGATGAGTATTTTTATCTCAAACATCGTGATGAGTG
TCGTGGGGTTGGTGGTTTATTTTTTGATGATTTAAATGATAAATCTTTTGATGAATGCTTTAATTTTGTA
ACTGACTGTGCAAATTCTTATTTAGATGCTTATATTCCAATAGTAGCACAAAGAAAAAATATTGAATATT
CACAAAAGCATAAAGATTTTCAACTTTATCGTCGTGGTAGATATGTTGAATTTAACTTAGTGTTTGATAG
AGGTACGATATTTGGCTTACAAAGTGGTGGGCGAACTGAGTCGATACTCTCATCAATGCCACCAATAGCA
ACTTGGAAATATAATTGGCAACCTGAACTAGGTTCTGAAGAAGAAAAAGTTTACCAATATATAAAACCTC
GCGATTGGATTAAATAA

>lcl|NC_006570.2_cds_YP_170067.1_949 [gene=FTT_1088c] [protein=hypothetical protein] [protein_id=YP_170067.1] [location=complement(1097857..1098603)]
ATGACTTTTCAATACACTAATTTTAAAATTTTAGAAGTAAGTGGTGTAGACACCAAAAAAATTTTTGCAAG
GGTTAACTACAGCAGATTTAAATGGATTATCAATTGATAATGATATTTTGTTGACAGCTTTTGCTAACTT
AAAAGGACGAATTATTTCACTGTGTTTTGTTAAGTTTATATCCAATGAGAAACTACTCTTGTCAGTTGAG
CAAGAGGTTTTTGAAAATCTACTAGCGTGGTTGAAAAAGTACGGGATGTTTTCCAAAGTTTCTTTTAATC
CTAATGATGATTATGCTTTGTTTTTCACAAAAACTGGCTTTTTAAATCATGATATTTTGACTAAAGGTTC
TTTAACTTCTGAAATGACTTTTGAGCAAATTCAAAAAGAGAATATCATTAATAAACTTGCAACCATAAAT
GCGGCTAATTTTGAGAAGTTTCTTCCTGCTGAGTTGGATTTAGATAATGTTGATAAGGTGGTTTGCTATA

CCAAAGGTTGCTATATGGGGCAAGAAGTTATAGCTAGGATGCATTACAAAGCTAAATTAAAAAAAGAACT
AGCAGTAGTTAAATCTGAGTCGGATATTGATGATTTTGATTTAAAGGATAGTGAAGGTAAACCTCTAGCT
AATGTTGTTAATAAAGTATTTGTTGATAACCAGTGCTATATGCTAGTTGTGTTTCATAAAGAAGCTTCTG
AGCAAGAGTATCAATTAGATGATGGCAAAATTATAACTAAATGCTAG

>lcl|NC_006570.2_cds_YP_170075.1_956 [gene=alaS] [protein=alanyl-tRNA synthetase] [protein_id=YP_170075.1] [location=complement(1105416..1108013)]
ATGATTACTACTAAAGAGTTACGTAATAAATTTATAAATTATTTTGAGTCTAAAAATCACTCACATCAAC
CCAGCTCATCTTTAATCCCATTTGGTGATGATACTTTATTATTTACAAATGCTGGGATGGTGCAGTTTAA
AGATGTTTTTCTTGGAATTGAAAAAAAGGATTTTTCGCGTGCGGTGACAGTACAAAAGTGTTTGCGTGCT
GGTGGTAAGCATAATGACCTTGATAATGTAGGCTATACAGCTCGACATCATACATTTTTCGAAATGTTAG
GTAATTTCAGTTTTGGTGATTATTTTAAGAAGGAGGCTATTAGTTTTGCTTGGGAATTTTTGACTAAGGA
AATTAAACTTCCTGTAGAGAAGCTATGGGTAACTATATATGCCAGTGATGATGAGGCTTTTGATGTCTGG
CATAAGCATATTGGTTTAGCTAAAGAGAGAATCATCCGTATTGACTCAAGTGATAACTTTTGGTCAATGG
GTGATACTGGTCCATGTGGTCCATGTACAGAGATCTTCTATGATCATGGTGAAGATGTTGCTGGAGGATT
ACCTGGTACTCCGGAACAAGATGGTGATAGATATATCGAAATTTGGAATATTGTATTTATGCAATATAAT
CGCCATGCTGATGGTTCTACTACAGATCTCCCTAAACCATCTGTTGACACTGGAATGGGCCTAGAGAGAA
TCTCAGCAGTGTTACAAAATGTCCATAGTAACTATGAAATAGATTTGTTCCAAGCTTTAATCAAAAAAGC
TCAACAAGTAACACATGCAAAAGATATAAACTCACCGTCACTAAAGGTTATTGCGGATCATATTCGCGCA
TGTGCTTTTTTAATTGCTGATGGGGTTTTACCTGCTAATGAAGGGCGAGGCTATGTTCTAAGAAGAATTA
TTCGTAGAGCTATTCGACATGGCAATAAAGTTGGAGCGAAAGAGATATTTTTCTATAAATTAGTCGCGGA
GCTTGTAAGCCAGATGGGTGAAGTTTATTCGCAATTAATTGATAAAAGAGAGTTGATTGAAAAAACACTT
ATAAAAGAAGAAGAACTTTTCTTGAAGACTATTGAAAATGGCATCAAAATATTTGATGCTGAAATAGAGA
ATCTAAAAGATAATACAATATCTGGGGAAGTAGCTTTTAAGTTATATGATACCTATGGTTTCCCTTTTGA
TTTAACTGCTGATATGGCAAGAGAAAAAGGCTTGAAAGTTGACGAACAAGCATTTCTTGCACAAATGCAG
ATCCAAAAGCAAAGATCAAAAGAAGCTGGTAAATTTAATGTTGATTATAATAGTTTGATTAATTCGCAAG
TTAAATCAGAATTTAGAGGATATTCGACACTGATAGAAGACGCAAAAGTTTTGGAGATCTATCAAGATGG
TCAGTTAGTTGCTAGTACTTCTGAACAAGTATCAGCTGTTGTTGTTTTGGATAAAACTCCTTTCTATGCA
GAGTCAGGGGGACAGGTAGGTGACAAAGGTATACTTGAGGGCATCGGCTTTGAGTTTGTAGTTGAGGATG
TCCAAAAGTCTGGTGAGGCGATTTTACATATCGGTAAGTTAGTAAAAGGTCATTTAAATTTAAATGATGA
GCTAACTGCACGAGTAAGTGATAAACCTAGACTTGCTACAGCTGCTAATCATAGTGCTACACATTTATTG
CATAAAGCTTTAAAATTAGTTTTAGGTGGTCATGCTGAACAAAAAGGTTCACTTGTTGATGAAAATAGGC
TAAGATTTGACTTTACTCATGACAAGGCTATTTCTCGTAGTAAAATAGAGCAAATAGAGCTTTTGGTAAA
TCAGCAAATACGTGCTAATTATCCAGTAACAACTATTGAAACATCGCAACAAAAAGCCAAGTCACTAGGA
GCAGAGGCTTTATTTGGTGAAAAGTATGGTGATATTGTACGTGTGATATCTATGGGTGATTTTTCTATAG
AACTATGTGGTGGTACTCATGTTGCCTATACTGGAGATATAGGTTTATTTAAGGTAACTTCTGAAGGGAG
TATTGCCTCAGGAGTTAGAAGGATAGAAGCAGTTACAGCAGATAAAGCTATAAGACATACTTTTACCAAT
GAAAATAAAATCATAGCGATTAAAGATAGTCTCAAAGCAAATGATACTAATTTGATAGATAAAATAAAGT
CAATGCTTGAACAGATAAAAAATCAAGAAAAGCAAATAGCTAAACTAAAAAAAGAATTGTTATCAGGCTC
TAGTAATGATATCAAAGAGACAAATATTGGTGATATAAAAGTTGTTGTCGCTAATGTTGATGGCGTTGAT
GTCAAAACATTACGTAATAAAATTGATGATTATAAATCAAAAAATACTAAGGTAATTGCAGTTCTAACTA
CAACTAATGCAGATAAGGTACAGTTTGTAATTGGAGTTAGTAATGCTTTAACAACTTTAATTAAGGCTGG
AGATATTGCTAAAGAGCTAAGTAGTCATATTGATGGTAAAGGCGGCGGTCGTGCTGATATGGCTCAAGGT
GGTGGTAATAACTCTGCGAATATAGATCAAGCTTTATCTCAAGTAGAAAAATTTATTTTAAATAATATAA
AAGAGTAG

>lcl|NC_006570.2_cds_YP_170086.1_967 [gene=rpoH] [protein=RNA polymerase factor sigma-32] [protein_id=YP_170086.1] [location=complement(1122307..1123185)]
ATGGCTAACAAAAAATTATTACCAGCTACGAAGACGAAAACTTTACCAGTTGTATCTGATAATAATCTCA
GTGCGTATTTAAATTTTGTCAATACTTTACCAGTATTATCACTAGAGCAAGAGCAAGAGCTAGCAAGACG
CTATAAATATAAAAAAGATCTAGATGCTGCACAGCAACTTGTGTTATCACATTTACGCTTTGTTACTAAA
ATAGCAAGAAATTTTTCAGGTTATGGACTTTCAATAGCGGACCTTATCCAAGAGGGTAATATTGGTCTTA
TGAAAGCTGTAAGTAAGTTTGATCCAGATCAAGGTGTTAGATTACTTTCATTTGCAGTTCATTGGATTAA
GGCGGAAATGCATGACTATGTCCTAAAAAACTGGAAGATAGTCAAGGTTGCTACCACAAAAGCACAGCGT
AAATTATTTTTTAATCTTAGAAGTAGTAAAGACAAAATTGGTTGGTTAAGCTCGGAGAATATCAAAGAGT
TAGCTGAAGAGCTAGGTGTCAAAGAAGAAACTGTCATTGAGATGGAGAAAAGAATGTGTCAGGGGGATGC
AAGTCTTGATTTACCATACACTGATGATGACGGTGAGCAAACTTCGCAGCAAAGTTTGTATCTAGAGGAT
AAGTCTTCAAACATTGAGCATCAAGTTGTTCAGCAAGATTACTATGATAATTTCAAAGCGATTGTAAAAG
ATGTTTTAAGTGGTTTTGATACACGTACAAAAGATATTATCATGTCGCGCTATCTACTTGATAACAAGGC
AACTCTTCAGGATCTAGCTGCTAAATATAATATATCAGCCGAAAGAGTACGCCAAATTGAAGAAGATGCT
CTAGCTAAGCTTAAAAAAGCTATCAAGAATCGCTCTTAA

>lcl|NC_006570.2_cds_YP_170087.1_968 [gene=FTT_1113c] [protein=hypothetical protein] [protein_id=YP_170087.1] [location=complement(1123209..1123739)]

ATGGTGTATAATTATAATGAAGAATTAATAAAGGAGCATTAATGAAAAAATTAGTTTTAGCAGCTTGTA
TGCTTGCTTTTGGTGTAAGTTCTAGCTTAGCTATGTCTGATAGTAAGCCGGTACAACCTCCAGCACAGAA
GCAAGATGTTGGTCATCCACATAAGTTGGTAGATATCTATAGCAAAGATGATCAAAGCTCTAAGGTATCA
GCTCAAATAACCCTTGAGAACCAAGATAATTATAATATATTCTATTGTAAGCAAAATAACTGGTGTGAAG
TTGTTGATAAAAATAATGGTAATACTGGTTGGATCAATCTGGATAAACTTAAACAAGCTCAAGAAAAATT
TGCTAAGCATGTACATAAGCAAAATACAATAAAAAGGTTAGAAGAGTATACTAAGGTTCAAGATCAAAAA
ATATCTCAATTACATGCTATGATGACGCAAATGCGACAAGAGTTTGCGTATGTATTAGAGCAGCAACAAG
CTCAAATTAATCAGCTAAAACAAGCGTACTACTATCAGTAG

>lcl|NC_006570.2_cds_YP_170088.1_969 [gene=secF] [protein=preprotein translocase subunit SecF] [protein_id=YP_170088.1] [location=complement(1123771..1124715)]
ATGGAATTTTTTAAACAAAAGACAAGCATAGATTTTTTAGGAATTAAGAAATATACAACTGTTTTTTCTG
TATTAATGATTGTTGTGTCATTATTTTTTATCTTTACTAAAGGTTTAAATCTTGGCTTAGACTTCACAGG
TGGTTATCAAGTACAAATTCAGACATCTACTAAGTCACAAGATTCTGAAACTATGACTAAAGAGTTAGCC
AAAGCTGGTTTTGAACATACAACTATTACTACTTTTGGTGATAATAATAACTTTCTCATTAAGTTTGCTC
CTGATGAGGTTAACACAAAAGCAAAAAGCTTAGAAGATGCACAACAATATCTCAAGCAGCAAGTCGAAAA
CTCGTTAGATGCGCAAGTTCAAAGTGTTAATTATATTGGTCCACAGGTTGGTAAAGAGTTAGCAAGTAAT
GGTGTTTTAGCAATTATAGTAGCGATGGTATGTATTTTGATATATATCAGTGCTAGATTTGAGATGAAGT
TTGGTATTAGTGCTTGTATTGCATTACTTCATGATCCTATAGTTATCTTAGGCATATTTTCGGCATTTCA
GTTAGAGTTTGATTTAACTGTATTAGCTGCGGTACTAGCTGTGATTGGCTACTCATTAAATGATACCGTT
GTAATATATGATAGAGTGCGTGAGAACTTCAGAAAAAATGCGTAATGCTAGTGTTGTAGAAGTTGTTAATA
GAAGTATTAATGATACTTTATCAAGAACTATTTTGACTTCTGGTCTAACAATGCTAGTTGTTGTAGTGCT
ATATTTGTTTGGTGGTAGTTCAGTACATAATTTCTCATTGGCTCTAATCTTAGGAATAGTGGTGGGTACA
TACTCATCTATATACGTTGCTGGTGTGGTAGCTGTTGCGCTAGGTTTAAATAGAGAATCATTACTTCCGA
AGCAAGTATCTAAAGAAGATATTCCTATTCTATAA

>lcl|NC_006570.2_cds_YP_170089.1_970 [gene=secD] [protein=preprotein translocase subunit SecD] [protein_id=YP_170089.1] [location=complement(1124734..1126671)]
ATGAGTAACAACAGAAGTTTACCTATAAATCAGTTTCCGCTGTGGAAAAATCTTTTGATCGTAATTATCT
TAGCATTAGCAATTTTTTATGCTTTGCCTAATGTTTTTGGTAAGAGCCCAGCTTTGCAAATATCACAAAA
AGATGGTGATGTGACTACACAGTTATTAGTAAGTGTAGAGAACACGCTAGCTAAAGATAAAATTAGTTAT
CAAAAAGCTGATATAGCTGATGACAAAAGTAATATTGCAATAACTTTCAAGGATGTCGAAGAGCAGCTAA
AAGCTAAAAAAGTACTAAAAGACAGTTTGAGTGATGACTATATTATTGCGATGAATATGCTCTCGAACTC
ACCAAATTGGTTATCAGCATTAGGTGCTAACCCTATGAATTTGGGACTAGATTTGCGTGGTGGGATGTAC
TTGATGTTAGAAGCTGATACAAAAACATCTATTGATGCTCAACTAGATAATTCTTTGAGCATAATTCTAA
GTGCTGCTAAGGATAATAGTATAAATATATCAAGCTCAACAAAAGCTGAAGAAAAATCAACTATCAAAGC
ACCACAAAATTATTTTGACAATGGTTTTGTTTCGATAACTTTAGCAAATTCTGTTGATGTTGATAAGCTT
AAACAATATTTAAGTACTGATTTCATCAAGACACAAGACCCTAATATAATTTACACAAACAAGGGTAATA
CTGTATTTATTTCTTATAATAGTGCTAAGATTCTTCAGCTTAAGCAAGATGCGATATCGCAGGTTGTGAC
AGTTATGCGTAATCGTATCAACGCATTAGGTGTAGCTGAAGCATCTGTTGCTCAGGCAGGTGATAATCGT
GTAGTGATTGAAATACCAGGCTTACAAGATGCTACCCAAGCTAAGCAAATTTTAGGTGGTACTTCTACAG
CAAGTTTCTATTTAGTAAATCCAGTTGCAGATAGATTAGCAGCTGAAGAGCAAGGTTATAAAGTTTATTC
TCTAGATAATGGTCGAGGCTATCAAAGCTACTATAGCTTAAAAGGAACTGCTATTGCTGGTGGTGCTGAT
ATTATCGGAGCTAGCCCATCTATAGATCGTCAGACTGGAACACCTATAGTCATGGTTGAGCTAGATAGAA
GTGCAGCAAGCCATTTTAGACAAATAACTGGTAAGAATATTGGTAACCCAATGGGTGTAATGCTTGTTAA
TACCACTTACGAGAAAGTCAAAGATAAAGATGGTAAAGAGAAAAAATGTAGTTAGCAAAACAGAAAAATTA
ATCAATGTTGCTACAATCCAATCTGCGTTAGGCTCACAATTTCAGATCACTGGTCTAAATCAAAAAGAAG
CAAATAATCTAGCTTTAATGATCAAATCAGGAGCGTTGCAGGTACCTGTACATATCGTCCAAGAGCAGCA
AATAGGTCCAAGCTTAGGTAAGGATAATATTGGAAAAGGTATGCTCTCTATCGTGATAGCACTTATAGCT
GTTGTAGTATTTATCTTAGTTTATTATCGTGTTTTCGGGATTATAGCTAACATTGCGCTAGTGATGAACT
TAATCTTAATTGTAGCGGTGATGTCTATTATCCCTGGAGCTACTCTGACATTACCAGGTATTGCTGGTAT
CGTCTTAAATTTAGGTATGTCGATAGATGGTAACGTGCTAATATTTGAACGTATTAGAGAAGAAATTCGT
GCTGGTATGCCTCGTCAAAGTGCAATACATATTGGTTATGAGAAAGCATTTACTACAATTGTAGATTCAA
ATATTACAACCTTGATTGTTGCGGTGATACTTTTCTTTATCGGTAGTGGCGCAGTCAAAGGTTTTGCAAT
TACATTAATGATAGGTATTGTCACATCTATGTTTACATCTGTTACTGTTTCAAGAGCGATGACAAATTTT
GTTTACGGTAAGAGAAAGAAACTAGAAAAAATCTCTATAGGCATATAA

>lcl|NC_006570.2_cds_YP_170090.1_971 [gene=yajC] [protein=preprotein translocase family protein] [protein_id=YP_170090.1] [location=complement(1126728..1127084)]
ATGAGAAAATTATTATTGTCACTATCTGCAATCGTATTATTTGTGTCAACTTCATTTGCACAAGGAGCAG
AGCAAGCTGCTGGTAGTCCGTTAAGCTCAATCTTAATGCTAGTGGTATTTTTTGCTATATTCTGGTTCCT
ATTAATCAGACCTCAACAAAAGAAAAACAAAGAATTGCGTAAGATGCTATCTGAGCTATCAAAAGGTGAT
GAGGTTGTAACTAACGGTGGAATGGTAGGCAAAATCGCTAAGATAGATGAAACATTTGTAGATTTAGAAG
TTGCAGATAATGTTACTGTTAAGATTCAAAGAAATGCTGTAGCAAATATTTTACCTAAAGGTGCTACTAA

AGCTTAA

>lcl|NC_006570.2_cds_YP_170096.1_977 [gene=ygiH] [protein=hypothetical protein] [protein_id=YP_170096.1] [location=1133213..1133827]
ATGAATTTTTTGAATTTCAGTATTCTAATATTTGCTTACTTGTTAGGATCTATAAACAGTGCAATTATCG
TATGTTATATATTTAGGTTACCATCACCTCGCAGTGTTGGTTCTGGTAATCCTGGTATGACAAATGTTCT
TAGAATCGGTGGCAAAGTTCCAGCTGCAATTACGCTAATATTCGATATTCTCAAAGGTTTAGTCCCAGTA
GTTATTGCTAAAGTCCTAACCGGTAATGAGTTTATAACCGCATGTACAGCACTTTATGCAATTCTTGGTC
ATATTTTTCCGATATTTTTCGGTTTCAAAGGTGGTAAAGGTGTTGCAACTCTTATAGGTACACTTTTTGG
TTTTAGCTGGATCTTGGGCTTAATCTTTGTAATCACGTGGTTATGTGTAGCTATAATTACACGCTACTCA
TCATTATCAGCTTTAGTTGCTACCGTTATAGCGAGTTTTTCAGTAATATTTACATCAGACCTACAAGTAG
CTGCACCGTTTCTAATAATAGCGATAATAATACTTGTAAAGCATAAAGGAAATATTCAAAGACTGATTAG
TAGACAAGAAAGTAAAATTGGTGACAAAGCAAAGGCAAAAAATGATTCAAATTAA

>lcl|NC_006570.2_cds_YP_170101.1_982 [gene=ispF] [protein=2-C-methyl-D-erythritol 2,4-cyclodiphosphate synthase] [protein_id=YP_170101.1] [location=1138435..1138914]
ATGTCATTTCGTATAGGTCATGGTTATGATGTGCATAAGTTCACTTCAGCAAAGCAAAATATCATTATTG
GTGGAGTCGAAATTGCTTATCATCTAGGACTTGAAGCTCATTCTGATGGTGATGTACTAATACATGCTCT
TTGTGATGCAATTCTTGGTGCTTTAGGACTAGGTGATATAGGCAAACATTTCCTGGATACGGATAATCAA
TTTAAAAATATTGATAGTAAATTTTTCTTAGCTGAAATAAAAAAAATGCTTGATAAAAAACAATATTCCA
TAAGCAATATTGATTGTACTATAATTGCTCAAGCTCCTAAGATGCTTCCACATATTGAAAAAATGAGAGC
TTGTCTAGCAAATATTCTTGAAATACAAATTAGCCAGATAAATATCAAAGCTACTACAACTGAAAGATTA
GGATTTATTGGTAGAGAAGAAGGTATTGCAACCCATGTGGTTTGTTTATTATATAGATAA

>lcl|NC_006570.2_cds_YP_170102.1_983 [gene=FTT_1129c] [protein=hypothetical protein] [protein_id=YP_170102.1] [location=complement(1138919..1140604)]
ATGATTCCTGAAGGTTATTCACGTAGGCTAGTTGGCCCTAATTTGTTCTTTAAAGAAACTGGGACAGTGT
TAGATGTGCCATTAGTAGACAATAGAGATGAGTTGACAAAACTTTTTTATCAAGAAGCTAATAGAATTTT
ATTAGCTCTTGATTGGCAAGATATTAAAATCACTCATAAATTTTTTAATAATGGCGTTAGATTTGCGATG
ACAGCTCCTGTAGACATAACTATGCCTGCCTGTGATGTCATTGATTTTATCTGGCTATCTACTCGAGAGG
GTTTTGAGACCGGAGTATTTAAAACTATTGAGGAGGCAAAGCGCAAGCTTATTCCATTGATAGATGAGGA
TAAAAACCTTACATATCGTAAGCTGTATGAGTTAGCTAAATCCAAAGGCTTTAATGCTTTTAGAGACAAG
AATAAAGCTTTTATTGGTTCTGGTAGAGGTTGTTATGAGTTTGATTTAGATAATGACTCGATAGATGACA
TTCCGTGGCAGGATATTTATGATATTCCCGCAGTTATTGTTACAGGTACAAATGGTAAGACAACTACAGT
TAGACTTACAGATTATATTTGTCGTGTAGCAGGTAAACTTACAGGATATACCTCTACAGATTGGGTAAAA
GTAAATGATCAACTAATTGATGAAGGTGACTATTCAGGACCGACTGGTCATCAATTTGTTCTGACAAATA
AAAAAGTTGAAGTAGCTCTTTTAGAATCTGCAAGAGGCGGCTTACTAAAAAGAGGTCTGATAGAAACTTA
TGTAAATGCTGCAGCTGTGACAAATGTTTCAGCTGATCACTTAGGTGAGGATGGTATTGAGACGGTTGCA
GAACTTGCTGAGGCTAAATCGATAGTTTTTCGTGCTTTAGGTCAAGGTTCACATGGGATTATCAATCTTG
ATAATTCATATATGAAAGAAAGATTTGATAAGCTTAGCTGCGCAAAAAATAGTGGTTACTCAGAACCCACA
ACAGCATGATATGAAGTATTATTTGTCAAAAGCAGATTATGCCTGTATAGTCGAAGATGGTAATTTTGTC
TGGGTTGAAGCAAATTCTAAAAAAGTAATATTGCCAGTTATAGAAGCACCATTAACTGTTAGAGGCTTTG
CTAAACATAATATTGAAAATGCTATGATTGCTATAGCCTTATCGTTTAAGTTAGGCATTAGTTTTGATGT
CATAGAAAAAGCGCTTAGAAGCTATTCGAATGATCCAAAAGTCAATAGAGGTCGAGCTAATGTTTTTGAA
TGGGATAATAAAGTTGCTATACTTGATTATGCTCATAATGAAGCTGGAATGGAAGCCCTTTTAAATATGG
TCAAAGCCTATGATAAAGGTGGTAAGAAATATTTGATGATTGGTACAACTGGTGATCGTAAGTATTTAAT
CTCTGGTATTAATGATATAGTTCTTAAACATAATTTGGACTTTATTGTTATAAAAGAAACAGAAAAATAT
TTGCGTGGCGCTAAGCCTTTGGAGTTGCCATTATTGATTCGTAAGGATTTGGCAGATAAAGGCTATGATA
TTTCTAATACATATATTTCTCATGGTGAAATTGATGGAGTTAAGTTTTTAGTTGAAAAGCTAGAAAGTAA
CGATATGGCAATATTCTGTTGTCAAGCTGAACTTGAAGAGGTAGCAAACTATCTTGAAGAGTGTGCAAAA
AAATAG

>lcl|NC_006570.2_cds_YP_170109.1_991 [gene=hemH] [protein=ferrochelatase] [protein_id=YP_170109.1] [location=1150231..1151232]
ATGCAACAATATAGCTGCAAATATAACAAACAAGCTATTCTATTAGTCAATCTTGGAACTCCAGACAATT
ATGATACTAAATCTATAAAAAGATATTTAAAAGAATTTCTATCTGACCCTAGAGTAATAGAAGCCAACCC
TGTATTATGGAAAATAATCTTAAACTTGATCATTTTACCAATTCGTGCTAAAAAAAATGTTCATACTTAT
AAAACTGTTTGGAATAAACAACATAACAAATCTCCACTATTATTCTACACAGAAAATTTAGCAGATAAAT
TGGATAAAAAACTAGATAACTATATCGTTGATTATGCTATGCGTTATGGCAATCCAAGTATAGAAAGTAA
AATTAAAAGCTTACAAGATCAAGGCGCTACTGAAATAATAATATTTCCACTGTATCCCCAATATTCTGCT
ACTACTACAGCAACAGTATATGATGAGGTTTATAGAGTTTTATCAAAACTAAGATGGCAACCTACTATTA
AGGGAATAAATCCTTATTATGATAATAAGTTTCATATTCAGACTATTTCTCAACAGATAAAAGAACATCT
AAAAAAGCTAGATAGTACTCCTGATACTGTTTTGTTTTCATTTCATGGTTTACCAAAAGAATATTTTGAT
AAAGGCGACCCTTACTACTGTCACTGCTACAAAACTTATCGTTTAGTCAAAGAAGAATTACAAAACGAAT

ATCCTAATATAGATTTTGAACTATCCTTCCAATCTCGCTTTGGACCTAAGAAGTGGTTAGAACCATATAC
TACCGTAAAGTTAGAAGAATTTACTAAGCAAAATAAGAGTGTAGTCGTTATTGCTCCAGGATTTAGTGCT
GATTGTTTGGAGACATTAGAAGAGTTAGCAATTTCTGAAAAAGAAAATTTCATTAAAAAAGGTGGTAAAG
AATTTAGTCTAATACCATGTTTAAATGATTCTAATCAACATGTCGATATGTTATACAATATAATAGATGA
GGAAATATGTCTAAAAAAATAG

>lcl|NC_006570.2_cds_YP_170114.1_996 [gene=dfp] [protein=4'-phosphopantothenoylcysteine decarboxylase] [protein_id=YP_170114.1] [location=complement(1157636..1158811)]
ATGAGTAATAAGATTTTATTTGGGATCACTGGCAGTGTATCAGCTTTTAAAACTATAAATTTGATAAGAC
TTTTTATTAAAAGTGGTGTTGAATGTAGAGCTATATTTACTAAAGGTGCACAGCAGTTTATAAAGCCTGA
ACTTTTAGTAGCTTTAGGCTGTGATGTTTATACTGATGAAAAACTAGATATGTTATCTTATCAGCAAAGT
ATGGCACATATAAATTTATCACGTTGGGCTGATAGGATATTTATAGTTCCTGCTTCAGCAAATACTATTG
CAAAATTAGCTTATGGTTTAGCAGATGATTTATTAAGTCAAACAATATTAGCTAATGATGATAATTCTAA
GGTATATATAGCTCCTGCTATGAATGTAAATATGTGGCAAAACCAACTTACTCAAGATAATATTAATAAA
CTTCAAACTCTAGGATTTAATTTGATATTACCTGATAAAGGTCTTCAAGTTTGTGGAGATATTGGTAGTG
GTAGATTACATGAGCCGGAAGTCTTGTTTGATTTATTGAGTGTTTCTCAAGATTTCAAAGCTAAAAAAGT
AGTGATAACAGTAGGAGCAACTGTAGAAGATATTGATGGTGTTAGATATTTATCAAATTATAGCTCTGGT
AAAATGGGCTTTGCTTTGGTAAGAGAGTTACTTGCAAGAGGAGCGAGTGTTGTAGTATTAAAAGCAAAAA
CTACAATAAGCTTTGATATTAAACATCCTAATTTAGAAATTATTAATACCAAAAGTGCTGATGATATGAA
CCAAGCAATGCTTGAAAAAGCTAAAGATAGTGATATTTTTATAGGCTGTGCTGCAGTTGCTGATTATAAG
ATCAAAAATAAATTTACTAATAAAATTAAAAAAACTGATGATACATTAACTTTAGAGTTTATAAAAAATC
CTGATGTTTTAGCTAATTGTAAAAAAACATACCCAAATATCTTTGCTATAGGTTTTGCAGCAGAATCACA
AAATATAGTAGATTATGCTCAGGCTAAACTTGTCAAAAAGAATCTAAATATGGTAGTTGCAAATTCAACT
GAAGTATTTGGTAATGATAACTCAAGTGTAACAATATTATCTAAGCATCAAACTAAACAATATAACAACA
AATCAAAGTCAGAAATAGCTAAGTTAATATTAGATTTTGCTAAGGAAATAATATGA

>lcl|NC_006570.2_cds_YP_170119.1_1001 [gene=FTT_1152] [protein=hypothetical protein] [protein_id=YP_170119.1] [location=1166334..1166717]
ATGGCTATTAGTCAAAATGTTATAAAAATACTTCAAGATATTGATGAACTAGATTTATTCAGCAACATCT
CAGATGAATGGTCAACTCTTCTCAATGAGTCTGATGATAATATTAAAATCAAAAAATTATATTCCGCATT
AGTTAAAGAAAGTATTCGCCATGAAACTGCTGAGAGGTTAGCTAAAGATGCTAAGTCATATTGTGACCTT
GTCCAAGAGCAAGCTAAACAACGAATATCTGATCTGAAAGAAAGTTTAGAAAGCCAAATCACTTTTCTAA
CTCAACAAATCAAAGACCTAAAAGTTGAGTCTGCAAAGAATCTAGACTATTATAGAAATGAACTGAAAAA
AGCTAATCGAAGCTTGATCGATAATGAAAGCTAA

>lcl|NC_006570.2_cds_YP_170122.1_1004 [gene=aroK] [protein=shikimate kinase I] [protein_id=YP_170122.1] [location=complement(1168142..1168672)]
ATGATAAGAACAAAAAATATTTTCTTAATTGGTCCAGTTGGTGCTGGAAAATCTACTATTGGTAAGCAGT
TAGCAAAACAGTTAAAGTTAGAATTTATCGATTCTGATGATGTTATTGAAAAAAAATGCGGCGTTGATAT
TAACTGGATCTTTGATCTAGAAGGTGAAGAAGGTTTTAGAAAGCGTGAAAGAGAAGTAATTGCTGAGATT
TTAGCTGAAAAACAAAATATAGTTTTAGCTACCGGTGGTGGTGCTATACTTGATCCAGAAACTAGATCAC
TACTGTCATCACGAGGTAAGGTTGTCTACCTTGAGGCAACAATTGAGCAACAGCTTGAGAGAACTTCTAA
AGATACTAAAAGACCATTATTAAGAGTTGATGATAAAAGACCAGTTCTTGAGCAGTTAATGGCTGAAAGA
GAGCCATTATATAGAAGTATCGCTGATGTGGTTGTGGAAACTAATGGTGCAACAGTTAAAAATATTGTGA
ATAAAATATCGACATTTTTAGTAGAAGAAACTATCCTGTGA

>lcl|NC_006570.2_cds_YP_170128.1_1010 [gene=adk] [protein=adenylate kinase] [protein_id=YP_170128.1] [location=1173477..1174133]
ATGCGTATAATACTTTTAGGAGCACCTGGAGCCGGTAAAGGAACTCAAGCAAAGATAATTGAACAAAAAT
ATAATATCGCTCATATTTCTACTGGAGATATGATAAGAGAAACTATAAAATCAGGTTCGGCACTTGGTCA
AGAATTAAAAAAAGTTCTTGATGCTGGAGAGTTAGTTTCAGATGAGTTTATTATCAAAATTGTCAAAGAT
AGAATTTCAAAAAATGATTGTAACAATGGCTTTTTATTAGATGGTGTCCCTAGAACTATTCCACAAGCAC
AAGAATTAGACAAGTTAGGTGTAAATATTGACTATATAGTTGAGGTAGATGTGGCTGATAACCTTCTTAT
AGAGAGAATTACAGGTAGGAGAATACATCCAGCATCTGGTAGAACTTATCACACTAAATTTAACCCACCT
AAAGTAGCTGACAAAGATGATGTAACCGGTGAACCACTAATAACTCGTACAGATGACAATGAAGACACAG
TTAAACAAAGACTATCTGTATACCATGCTCAAACCGCCAAGTTAATCGATTTTTACAGAAATTTCTCATC
AACGAACACTAAAATACCAAAATACATTAAGATAAACGGCGATCAAGCTGTTGAGAAAGTGTCTCAGGAT
ATTTTTGATCAGCTTAATAAAAGATAA

>lcl|NC_006570.2_cds_YP_170130.1_1012 [gene=era] [protein=GTP-binding protein Era] [protein_id=YP_170130.1] [location=complement(1177905..1178798)]
ATGAAAAAATGTGGCTACATTTCAATTATAGGTAGACCTAATGTCGGTAAATCTACTTTGTTAAATAATA
TACTTAAATATAAAGTAAGTATCACTTCACGTAAACCTCAGACAACTAGACATCAAATTACAGGTATTAA

GACGCTTGGTGATACTCAGTTTATCTATGTTGATACTCCAGGTATACATATCAAAGAACCTAAAGCTATA
AATAAATTTATGAATAAAGCAGCAACAACAATGTTTAAAGATGTTGATGTAATTTTATTTGTAGTTGAGA
TGGGTAAATGGACAGAGCTAGAGGATAATATTGTTGAGAAATTAAAGCACTCAGAAATTCCGATATTTTT
AGTTGTTAACAAGGTTGATAAGAAAAAATCATTAGAAGCAGCTATGTTTATCGAATCTATTAAAGAAAAG
CTAAGTTTTTATGATGTGATTTATGTATCAGCTAAGCAGGGACACAACATCAATGAGCTAGAATCAAGAA
TTGAAAAGCTTTTGCCAGAGTCTGAATATTTTTTCTATGAAGAAGATCAAATTACCGATAGAAGCATTAA
ATTCATGGTAGCTGAAATTATCCGTGAGAAGATTATGCGTACTATAGGTAGCGAGGTGCCATACCAAATA
GCTGTAGAGATAGACAGCTATAAGGTTGATCAAGAAAAAAATATTGTCTATATCTATGCTAGTATACTTG
TTGAGAGAGATAGTCAAAAAGGTATTGTGATAGGTGCTAAAGGAGCCAAACTCAAAAAGATTGGTACAGA
CTCACGTATAGATATCGAAAGGTTAGTTGGTATGCAGGTTAACCTTAAAACTCATGTCAAAGTCAAAAGT
GGCTGGTCAGATGATGATCGCGCGTTGAAGTCACTTGGCTATGATCTAATCTAA

>lcl|NC_006570.2_cds_YP_170131.1_1013 [gene=aspC2] [protein=aspartate aminotransferase] [protein_id=YP_170131.1] [location=complement(1178912..1180105)]
ATGGCATCGCTTAACAAAAAGATTCAAAATGTTTCGACTTCACCAACTAATGCTATGGCGGCATTAGCAA
AGCAAATCAAAGATCAAGGAAATGATGTAATCTCTCTTGCTATCGGAGAACCTGGATTTAGTACCCCAGA
TATTATCAAAGCTGCTGGTATCGAAGCAATAAATAAAGATATTACTAAATATACAAATGTCGATGGTCTT
AAAGAACTGCGTGAGGCTATAGTAGCTCGTTACAAGCGTGAATATGGTATTGAGTTTGCTGCAGATCAAG
TCTGTGTTACCTCTGGGGCTAAGCATAGTTTGCATAATATTTTTAACTGTATTTTAGAAGCAGGTGATGA
GGCGATTTTCTTTGCACCGTATTGGGTATCATATCCAGATATGATAGCACTTACAGGAGCAAAACCAGTA
GTTGTCGAAACTAAGTTTGAGAATAACTTTGAGATAGATGTTACAGACTTAGAAAAACATATTACAGCAA
AAACTAAAGCTGTGATTATCAACTCACCTAATAATCCTACCGGTTTGATCTACTCTAAAAAATGTATCGA
AGATTTAGCAAATTTACTTATAAAGTATCCAAATATTTGGATTATTGGTGATGATATTTATGACCAACTT
TACTTTAAGGATAGAGTTACATTGATAACTGAAGTAGCACCTGATTTAGCTGATAGATATGTGATCGCTA
GTGGTGTGTCAAAAAACTTTGCGATGACTGGTTGGCGTGTGGGTTTTACAATAGCGCCAAAGCTTTTAAA
TGATGCACTGAAAAAATTCCAGTCACAATCTGCTACTTGTGCTTGCTCGATATCACAATATGCTGCAATT
ACTGCTATGAATATGCCAGCACAAGATTTACAGTATTTTGTTGAATCTTACAAGCAAAAAGCACAATTCG
TTACTAAGTATCTAGAAGCTATGCCATATGTTGATGTAAAGAGTGCTGAAGGTACTTTTTATCTTTTTCC
AGATCTTAGAAAATTACTTGAGCATACTAATTTTAGTACTGATGTCGAGCTATGTAACGCACTACTTAGA
GAAGAGTATGTAGCGATGATGCCTGGTGTGGCATTTGGTTTATCAGGTTTTGCTAGGATAAGTTGTGCTA
ATGAAATGCCAGAGCTTGAAGAGGCTATGACTAGAGTAGCCAGATTTATTAATAAGCATGTTTCAACAAA
GTAA

>lcl|NC_006570.2_cds_YP_170133.1_1015 [gene=FTT_1167c] [protein=glycoprotease family protein] [protein_id=YP_170133.1] [location=complement(1180925..1181563)]
ATGAATTTTTTATTATTAGATACATCAAGTAAGTATTGTTCAGTGGTGTTATCAGCCGCTGGTGAATTGT
ATAATGATACGCGTGAGATACCACGCCAACACAACAAATATCTACTTGAAATGATACAGGGAGTTTTCGC
TAAGGCGGCGGTTAATATCAAAGATTTAGATTTTATAGCTTATGGCGTTGGACCTGGTAGTTTTGTTGGT
GTTAGGCTTGCTGCAGCAGTTTGTCAAGGTTTTGCGGTTGGTCTAGATATACCAGTGATTGGCTTTTCTA
GTATGTTTGCATTGGCAAAAAGTGTCACGACTGAATCTCAAAAAGTAGCTGTTATTCTTGATGCAAAAAT
GGATGATTTTTTACCTTGGACTTTATGATAAAGATACAGATCAGATAATTACAGAGAATGTCTATAAATTA
GAGGAATATTCTCAAGATCTATATGCCGGTTATCAACTAGTAGGTGAGTCTATCGCAGAACTACAACTAA
AGAATGATGATTTTAAAATAGATGTTGCTAATGTAGTAGAATATGTCTATAAACAATACCAAAAACAAAA
ATACGATGGTACTTTAACTCAAGAAACATTTCCAGTATACCTCAGAGGAACTAGTCATTGGCAGGCTAAA
AAGGAGTAG

>lcl|NC_006570.2_cds_YP_170135.1_1017 [gene=FTT_1169] [protein=GTP-binding protein] [protein_id=YP_170135.1] [location=1182763..1183635]
ATGTTACATTGGTTTCCCGGGCATATGCATAAAGCCACCAAAGAATTTCGCAAAAAAATGCCTTCGATTG
ATATCGCTATCGAGATAGTTGATGCACGTATACCAGACTCTAGTAGTAATCATGTTTTAGAGCAGATTGT
TGGTGACAAGCCGATTATAAAAGTTCTCTCAAAAAATGATCTGGCAGATACTACAATCACTAAGCAATGG
CTGGACTACTATAAGGGTAGTGCCATTGCAGTAAATACTCTTGAGGATAAAAAATATTGTCAAAAGAATTC
TTGATTTAGCACAAAAAAAAATGTCCACAAAGAGGCACTGTGCTAAAACCTATCAGAGCTATTATTTTTGG
TCTGCCAAATGTAGGAAAATCTACAATGATAAATAAACTTGCAGGTCGTAAAGTTGCTAAAACAGGTAAT
GAGCCAGCCGTCACAAAACTACAGCAACGTATCGATATTAGTAAAACTTTTATGATATTTGATACTCCAG
GAATTATGTTTCCTAGTCCAAAAAGTGAAAATAGCGCTTTTAGAATCGCAGCTATAGGCTCCATCCGTGA
TACTGCGATGGATTATGAAGGTACAGCTTGTTATTTACTAAACTTTTTTAGAGAGAAATATACAAAAAAC
TTCTTAGCTCGCTACAACTTCATTAGCGAAAAAGATTTTTTAGAGAGACATCCTCAAGAGATACTCAAAG
ATATCTCAATTGCTAAAACTAATTCAAATCTCAAACAAGCTGCCAAAAATATAGTCCATGATTTCCGTGC
TGGACACTTTGGCAAGATCTCGTTAGAAGATCCACAAACTATCGAAGCCGAAAAGCAACAACAAATACTA
CTAGAGCAACAACAACTACAACAAAATCTCTAA

>lcl|NC_006570.2_cds_YP_170147.1_1027 [gene=FTT_1185c] [protein=hypothetical protein] [protein_id=YP_170147.1] [location=complement(1201508..1201939)]

ATGAATAAGGTTAATAAATCTGCAGTTGTAAATTATAGTGCCGCACAGATGTACGAGCTTGTAAATGATA
TTAGAAGCTACCCTAAATTTCTACCGATGTGTTATGACATTGAAATTTTTGAGCAAACTGAAACAGAGAC
AAAAGCATCATTGAAAATAAAATCTGGATTTGTGAAGCTTGATTTTGGTACGCATAATACAATGGTCAAA
AATGAGCATATTCATCTCAACTTAATGAATGGTCCATTTAAAAGCTTAACTGGTGATTGGAAGTTTGAGC
CAATAGATGAAGACTCATGTAAGGTTTCATTAGATATGGAATTTACCTTCGAAAATAAATTTGTCGAGAT
GGCTCTTGGACCAGTTTTCCGTGGCTTAGCAGATAAGATGTTAGGCGCTTTTTGTAAGCGTGCAGAAGAG
GTTTATAAATAG

>lcl|NC_006570.2_cds_YP_170148.1_1028 [gene=smpB] [protein=SsrA-binding protein]
[protein_id=YP_170148.1] [location=1202014..1202487]
ATGAGTAAACATAAGGTTTCTCCAGCGACTATTGCTAAAAATAAAAAAGCCTTGCATGATTATACTATTT
TAGAGAAATTTGAAGCCGGAATAGTTCTACAAGGTTGGGAAGTAAAAAGTATTCGCGCTGGTAAAGTACA
AATGGTTGATAGTCATGTGCATATTAAACATGGTGAAGCGTGGCTTTTTAATTGTCTAATCACACCTTTG
CTATCTGCCTCAACTCATGTGGTTGCAGATGCTGCAGCAACACGCAAGCTACTTCTCAATCGTCGTGAGA
TTAATAAGATTATGGGTAGAATTGAGCAAAAAGGTTTTACATGTATACCTCTTTCTATGTATTGGAAAGG
TCCACGAGTAAAAGTAGAAATAGCATTAGCTCAAGGTAAAAAAGTTCATGATAAGCGCCAAGCACAAAAA
GACAAAGACTGGGCACGCGAAAAAGATAGATTATTTAAAAAGGCTTATAAATAA

>lcl|NC_006570.2_cds_YP_170156.1_1035 [gene=murI] [protein=glutamate racemase]
[protein_id=YP_170156.1] [location=complement(1214431..1215228)]
ATGCTTGATAATAGACCTATAGGTGTTTTTGATTCGGGAATCGGAGGTTTGACAATTGTTAAAAACCTCA
TGAGTATATTACCAAATGAGGATATTATCTATTTTGGTGATATAGCTAGGATACCTTATGGGACAAAATC
GCGAGCTACGATTCAAAAGTTTGCAGCACAAACAGCAAAGTTTTTGATTGACCAAGAGGTCAAAGCAATC
ATTATTGCTTGTAATACTATTTCAGCTATTGCTAAAGATATAGTCCAAGAGATTGCTAAAGCAATACCGG
TAATAGATGTAATAACAGCTGGTGTAAGCTTAGTAGATAATTTAAACACAGTTGGTGTAATTGCTACTCC
AGCAACCATCAATAGTAATGCATATGCTTTACAAATTCATAAAAAAAATCCTAATATTGAGGTGTATAGT
AATCCTTGCGGTTTATTTGTATCAATGATAGAGGAAGGCTTTGTTAGTGGTCATATAGTAGAACTAGTAG
CTAAAGAGTACCTTAGTTATTTTCATGATAAGAATATTCAAGCCCTGATTTTAGGTTGTACACATTATCC
AATTATCAAAGAAAGTATTGCAAAAATTTTAGATGTAAAACTTATAGATCCATCATTACAAGCTAGCAAA
ATGCTTTATTCGTTACTTTTTGAGAATAAGCTCTTAAACACTACTAAATCTAATCCAGAATATAGGTTTT
ATGTAACTGATATTCCTTTGAAATTTAGATCAGTTGGTGAGATGTTTCTGCAAACAGAAATGCAACATCT
TGAGATAGTAAGTTTAGATAGCTACTAA

>lcl|NC_006570.2_cds_YP_170164.1_1043 [gene=gidA] [protein=tRNA uridine 5-
carboxymethylaminomethyl modification protein GidA] [protein_id=YP_170164.1]
[location=1223517..1225400]
ATGATTTATGATTATGGTTATGATGTTATAGTTGTTGGTGGTGGTCATGCAGGTGTTGAAGCTGCTTCAG
CGTCAGCTCGTATAGGTGCAAAAACTCTGCTACTAACACACAATATTGATACTATTGGACAAATGTCTTG
CAACCCTGCTATTGGTGGTATTGGCAAAGGTCATCTAGTCAAAGAGATAGATGCTATGGGTGGTGTCATG
GCAAAGGCTATCGATATGGCTGGTATCCAATTTAGAATACTTAATTCCCGTAAAGGACCAGCTGTACGCG
CCACAAGAGCACAAGCAGATAGATTATTATACAAAAAAGCTATAAATTCTCTTATCAATAATCAAGAAAA
CCTTGATATTTTCCAAGACTCTGTAGATGATTTGGTTGTTGAAAATAATACTGTATGTGGCGCTATTACG
AAAACAGGTATTACATTTAGAGCAAAAAAAGTAGTACTTACTGTTGGTACGTTTTTAGGTGGCAAAATAC
ATATTGGTAAAGTCTCTAATGCTGGTGGTAGAGCCGGAGATCAGCCATCAAATGCACTAGCTGCCAGACT
TAGATCATTACCTTTTAGAGTAGATAGACTTAAAACTGGTACGCCTCCTCGTATAGATAGACGCTCAGTA
GATTTTAGCGTTATGGAAGTCCAACATGGTGATAATCCTACTCCTTACTTCTCATTTTTCTCAAAAGGTA
AGATAGAGCATCCGAGACAAATACCATGCTATATTACTTACACTAATAATGAAACACATAAAATTATCAC
TGATAACCTTGATAAATCAGCGATGTACAGTGGTTTAATAGAGGGTATTGGTCCACGCTATTGTCCTTCT
ATTGAAGACAAAGTAGTTAGATTTGCTGACAAAGAAAGACATCAAATCTTTGTTGAACCAGAAGGTTTAA
ATAGTATCGAGTTGTACCCAAATGGTTTATCAACTAGTTTACCTTTTGAAGTTCAATGCAACTATATCCG
CTCAATTAAAGGTTTTGAAAAAGCTTTTATAATGCGCCCAGGCTACGCAATTGAATATGATTTTTTTGAC
CCAAGAGATCTAAAACCAACACTAGAAACTAAGCATATCAAAAATCTATATTTTGCTGGTCAAATTAATG
GTACTACAGGCTATGAAGAAGCTGGTGCCCAAGGTTTAGTAGCTAGTATTAATGCTGCTATTAGCATAGA
TAGTGACAAATCATGGTATCCAACTCGTGCTGATAGTTACATCGGCGTATTGATTGATGATCTGATTACT
AAAGGTACAAAAGAGCCATATAGAATGTTTACCTCACGTGCCGAGTATAGACTTATTCTACGTGAAGATA
ATGCTGATTTACGCCTTTCTAATAAAGCTTGTGAACTAGGACTTTTAAGCAAAGAAGATCAACAACACTT
TATCAGCAAGAAAAATGCTATAATTGAAAATATCGCAATGATGAAAAACACTTGGATAGGCCCACAAACA
CAAAAAGCTCGTGATCTAGAAAAATTCTTAGATAAGAAAATGACACGTGAAAGTACTTTATTTGACTTAC
TCAAAAGACCAGAAATAGATTACAGTAAGTTACAACAGATATCTGAGCTAAATCTAAACTTACAAGATGA
CGCTGTCATCGAACAAATAGAAATTTCAGCAAAATACTCTGGCTATATTGAGCGCCAAAATAAAGATATT
GAAAAAACAGCAACTTTTGAACAAAAAGCTATCCCAACAGATTTCAACTATTCGCAAGTTAAGGGATTAT
CTAATGAAGTTCTACAAAAATTGACAGAACAAAAGCCTACTACTCTAGGCGAAGCATCACGTATCCCAGG
TATAACTCCTGCAGCTATATCATTGTTGACTATATATATGAAAAAAACTGGGTTTATAAAATAG

>lcl|NC_006570.2_cds_YP_170166.1_1045 [gene=FTT_1207] [protein=hypothetical protein] [protein_id=YP_170166.1] [location=1225818..1227188]
ATGAACATCAAACAAACTTCAACACCTCTAGTTACAGGTCTTGTTTCTGCCTCGATTTGCTTTAGTTTTG
GCTTTTTTACAAATGTTTTTAAATACAATGGTCTTGAATTTTTTTATGCTTATATAGCTTTTCTTATACT
ACTCTGTTATCCGATGAATATAGCTGCGCTATATTTCCAAAAAGCTTTTCCTAACTTAAACTCACACAGT
AAACTAGTCTATAAAATTACTGGAAGCTCTAAGTTCAGACCAATAAGTATTTTATTAACTGGTTGTATGG
TAATTTTAGTAGCATTGATAATGTTTGACATAGCGACTTATGTTTTAGATTTTTTTGACAACATTCCAGC
TATTGATCGCTTAAGCGATGAAAACCTTAAATTTAATAGTAATCTACCTATATATGTTTCACTACTTGTG
GTATTTATTGTAATTCTTCTAATGTTTATTCTAGCTGATAAGAGAAGGCTGAATTTAAATGAAACTCTAA
AGACTAGTGCTCATGTATCTTTATATTTAGTTACTATCTTAATGCTAATAGTTATATATTCACCACAAGG
CATGTTAGGCATAAAAGACTTCCTCTTAGACTTAAATTACCAAAAAATTAGTCAACTAAGACAAATGTTT
GCTTTAGCGCTAATGTATGCAATTTTAAGTAATTTTATATCGATAGCATTTTATAAAAATATAATAAATA
TAGGCGATGATAATTACAGTAAACTTAAGACTAGCGCTTTTAAGAGTATTTTCTACAATATAATTTTCTC
ATTTATTATTTGCGTGACTATATACGCTATTTTAGGCAATTACAGAAGCTATTTACAACCAACTGAAGGT
ATACAAATAACAACAGTTTTCAAAATTATTAAATTCAATTCACCAATGTATTATTTACTTCTTGAAGTAA
TATTTACAACTCTTAATTTAGTTGTTTTTGTTGCTGCCTTAAAATACATTTTTGAAATTGGATCTAAACT
TTATACAAAGCTTTTAGTTCTACTTATACCATTTATTATGGCTATCACATTTATAGAATCGGGTATAGCT
AATATAGACTTCTCTAAAATGTTTGGTCTACATTTAGTGATTATTTTTATCTTTTTATTTGATGTATTTA
TAGTTGGCTGGATCTATGATGCTCAAAAGCTTAGTTATGAAATACTCAAAAACACAAATACTAGACTTTC
ACTGATCTTTAACATTATGCTGAGAATAATAATACCTTTTATCTGTATTCTTGTTACAATTGGATATATA
TTCTTACCTATGCCAATAATATGGCAATTTATTGCTACTTTAGCATGTATGATTATCTATATTGTCAAAG
GAAGTATTTTTAGTAACATATTTAGCAAGAGAAAGTTTTAA

>lcl|NC_006570.2_cds_YP_170167.1_1046 [gene=rpiA] [protein=ribose-5-phosphate isomerase A] [protein_id=YP_170167.1] [location=1227190..1227864]
ATGTTTTTTAATAAAAAAAATAACCAAGATGAGTTAAAAAAGCTTGCCGCAACTGAAGCTGCAAAAAGTA
TTACTACAGAAATTACTTTAGGAGTCGGTACAGGAAGTACTGTTGGATTTTTAATCGAAGAACTTGTAAA
CTATAGAGACAAGATCAAAACTGTAGTATCAAGCTCGGAAGATTCAACCCGCAAACTCAAAGCGCTTGGT
TTTGATGTAGTTGATCTTAATTATGCTGGTGAGATTGATTTATACATTGATGGTGCAGATGAGTGCAACA
ATCACAAAGAACTCATCAAAGGTGGTGGTGCGGCTCTGACACGTGAGAAAATTTGTGTGGCTGCTGCTAA
GAAATTTATCTGTATCATTGATGAATCAAAAAAAGTTAATACATTAGGTAATTTTCCTCTTCCTATAGAA
GTTATCCCAATGGCAAGAAGCTATATAGCACGTCAAATAGTAAAGCTTGGCGGTCAACCAGTATATAGAG
AACAGACAATTACAGATAATGGTAATGTTATTCTGGATGTATATAATCTAAAAATTGATAACCCTCTAAA
ACTTGAAACAGAACTAAATCAAATCACAGGCGTTGTCACTAATGGCATCTTCGCTTTAAAACCTGCTGAT
ACTGTGATTATGGCTACAAAAGATAGTAATATTGTTGTACTTTAA

>lcl|NC_006570.2_cds_YP_170175.1_1054 [gene=visC] [protein=monooxygenase family protein] [protein_id=YP_170175.1] [location=complement(1235593..1236828)]
ATGAGCGTTAGAAATATTCAAAAAGATGCTGTGATTGTCGGTGGTGGTATGGTAGGCTTAAGCCTTGCTC
TAGCATTGCACCAAAATGGTTTGCAGGTTGCGCTTGTTGAGGCAAAAGAGATTAATTATAAGTCTTTGAG
TGCTGATAGAGTTGAAACTAGAGTTAGTGCTATAAACCATACTTCGAAAAGTTTTTTACAACGATTAGGT
GTGTGGCATAGTATCAAAAATAAGCGTATATCTCCATATTATCAAATGAGAGTTTGGGATGATATTCCTA
GTGAGAGTATAAATATTACAGCTGAAGAAATAGCTGAGCATAGTTTAGGCTGTATTGTTGAAAATGATGT
AATAGTTGAAGCGCTACTAGAGAAGATCAGAGATACAGGTATCGAGATCTTTGCTAATCAAAAAATACAT
AAAATTCAAAGAAATGGTAATACTGAAAAGCTCTTTCTACAGGCAGTCATTCCGGACTCTTGCTCTGATG
GTTCTAGTCAGAATAGTGATGACAAGTATTTATCAATTGAAACTAGTCTAATAATTGGTGCAGATGGTGC
TAACTCTTTTATTCGCGACTATTTTAATTTTGAAACCAAAGTTAAACCATATAAACATACTGCAATAGTT
GCAACAATTAAGCTTGAAAGAAATCACCTGCAAACAGCATATCAACGTTTTTATGATAAAGGTGTATTGG
CATTTTTACCCTTAGAAAATTCAAACAAAGCTTCAATAGTTTGGTCTGTAAAGAGTGATTATGCTAATTT
TCTGATGAGTTTAACAGATGAGAAATTTGAGCTAGAACTTACTAAGGCAATTGATAATAGTTTTGGTAGT
CTTAATTTACTATCAAAAAGATTTAGTTTTGAGCTAATAGAGCGTCATGCTAAAAGTTATATACAAAATA
ATGTAGTATTGGTAGGTGATGCAGCTCATACAATTCACCCATTAGCAGGGCAAGGGGTAAATATAGGTTT
TAAAGATGCTATTGCATTGACTGAAATTTTGACTGAGGCTTTTACAAAAGGTCGCTTGATTGGTCATATT
TCAACATTAGATAAATATCAGCGTGAGCGTAAGCTTGATAATACTAAGATGCTAGCTTTGATGAAGACAT
TTAAAGAAGTCTTTGCTAGTGATAACCAATATCTCAAAAACGCGCGCAGGGCAGGATTTGAGTTTGTTGA
TAAAAATAGAATTGTTAAAAGTATAGTTGTGAAGCAAGCTTTATAA

>lcl|NC_006570.2_cds_YP_170176.1_1055 [gene=ubiH] [protein=2-octaprenyl-6-methoxyphenyl hydroxylase] [protein_id=YP_170176.1] [location=complement(1236818..1238029)]
ATGAATGCTAAATATGATGTGGCAATTGTTGGGGGTGGATAGTTGGTTTATTTACTTCTTTAGCTCTAG
CCAAGACTGGATGTAAAATTATCCATATTGAAAAAGATCAGCTGCAAGTAAAAAATGATAATAGAAGTAT
TGCGGTATCTTATTCATCAATAGCTTTTTTAAATACACTTGGTTTGTGGGATAAGGTTGCAAGTAAAACT
CAAGCTATTAAAAAAGTTCATGTTTCAGATAAGGGCAGATATGGACGAGCTGAGATTTTTGCTAAAGATG

AAAATTTGCCATTTTTAGGAGCTATTGCGCCGATGCAGGAGCTTCTGACAGTTGCTTTACAAAGTGTTGC
TGCTAATCCAAATATTATCAAATCTTTTGAAACAAATGTTATTGACCTTGCTAAACATGATGATAAGTAT
TCTTTAGTAGTCAAGCAACAAGAACAGACTAAAACTATACGAGCACAGCTGATAATAGCTTGTGATGGCG
CTAATTCAAGCTTAAGAAAAATGCTAAATATCACAGCTAAGACAACTGATTATCAGCAAGATGCGCTTGT
TTTTGATATTCAAACAGAGCTTGATAACAATAATACAGCTTTTGAAAGGTTTATGACTGATGGTGTACTA
GCGATGTTACCTAAAGTAAAAACAACTATGGGCTGTGTTTGGACTATTGATAGAGATAATTCAAAAGCAA
AGCTAACCTTAGACAATAAAGAGTTTGAACAGTTAGTCCAAGATCGCTTCGGCTATAGGTTAGGACAGAT
TAAACTTACCTCAAAGCCTGCGGTTTTTCCTTTATACCTTGTCCAAGCAGAGCAAGTCTACAAAAACAAT
GTACTTTTTTTTGGTAATGCATTGCATTTTTTACATCCAGTATCCGGTCAGGGAATGAACCTAAGTATTC
GTGATATTGGTTTTTTGTATGATTTGCTTGTAGAGAGTGATTTTTCTCAGAACTCAATTACAGCTGTTTT
AGCAGAGTTTGCCAAAGTTAGAAAACCAGATCATGATAGAACAATTTTTGTCACACATGGATTTGTAAAA
TGGTTTGTATCTAATGATACTAAATTTGTTGCAAGTAGAAATGCTGGTTTACATCTGTTACAAAGAAGTA
AGTTAGCTAAAAAAGTTTTATCAAGAGTTATGATGGGCAAGCTTAGCAAAGGTTCAACTCTGATGAGAAA
GGTGGTCGAGGATGAGCGTTAG

>lcl|NC_006570.2_cds_YP_170177.1_1056 [gene=FTT_1219c] [protein=hypothetical protein] [protein_id=YP_170177.1] [location=complement(1238038..1238574)]
ATGAAAAATGAAAAACCTAGTTTTGAAGATGTCGCTGAAGCATTAAAAGTAATGCAAGCTCTAAGTTCAG
CTTCTGAAGCTCATGGACTGCTTTGTGCTTTGTTTAGTTTTGGTGCTGAGGTTAAATTTACAGCATGGTC
AGATTCTCTAATGACTAAGCCGATTGAAGAGGGTGATTTAGTTGCAAGTTCAGCTTTAAAAACTATGAAA
AAACTTTATGACTATACCAAGTCTCAGTTCGATGAAAAAGGCTTAAGTTTTGATTTGTTTATCCCAGCTG
ATGATGAGCCTTTAAGCTACCGAGCTGAGGCTCTGACATATTGGATAAGAGGATTTTTATCAGGAGTAGG
CTTATTTGGTTTGGATTTTGAGAACTCAAAAGATAAAGAAATCAAGGAAGCAATTAGCGATCTGATGAAA
ATTTCATATATGGATTATGAAGCTTTAGGTGAAGATGAAAGTTGTAAAGAAGATTTTATCGAGTTGCTTG
AGTATACAAAAGTAGCAGTTTTACTTATTGATAGTGAAAAAATTTAG

>lcl|NC_006570.2_cds_YP_170183.1_1062 [gene=FTT_1225c] [protein=hypothetical protein] [protein_id=YP_170183.1] [location=complement(1241886..1242215)]
ATGTATAATGATTTGACTCGAGAACTTTTAAGACAAGTTAAATTTGAAGATGGAATTATCTTAGCGGAAC
AGACTAAATATAGCGTTAGTGATAGTTTTTTAACGGTTGAGATTTATATTTGTGATAAAGGGGTATCATA
TAGAGTTTATGGCGATGCGTATATTTTAGCGATGCTCAAGTGGCTTCAGCTTAGCTTGCTAAACAAGCAA
AATCTAAGTCAAATTTCTTTAGAAAAACTAATAGCAGATTTTGATTTGCCACAAGTTAAGTATAGGGATG
CACTACAAATAATTAAGCTTATTGAGAAAATAAATGCAGCAGCTATATGA

>lcl|NC_006570.2_cds_YP_170184.1_1063 [gene=iscS] [protein=cysteine desulfurase] [protein_id=YP_170184.1] [location=complement(1242208..1243383)]
ATGAGTCTTATTTATTTAGATTATGCTGCGACAACTCCTCTTAGTCAAAGTGTCAAAAACTCTATGTTAA
ACTCTATAACTAGTGATGATGATTTTTTTAATTCTGGATCATTGACATATCAACAAGCAGAAATTGTTAG
TAATAAAATTGAACAAGCAAGGGCTACAATTGCACAAACTCTAGAGGTATTACCAAGGGAGGTCATTTTT
ACATCGGGAGCAACAGAGTCAAATAACCTTGCGATAAAAGGAGTAGCATATGCATATAAAGATAGAGGCA
GACATATAATAACATCAAAAGCTGAGCATAAGGCAGTATTAGATGTATGTAAGTTTTTAGAGACACAGGG
GTTTGATGTTACATACCTTGATGTTAACCAGTTTGGTGAGGTTGATTTAGAGCAGCTTAAAAAAGCTATC
ACGCCACAAACAATACTTGTAAGTTTAATGGCTGTAAATAATGAACTCGGGACAAAAAAATAATCTTATAG
AAATTGGTAAAATAACCAAGCAAAAGGGCGTGATGCTGCATGTTGATGCTGCCCAGGGTTATGGCAAAGT
TGATATAGATATCAAAGCGATGAATATTGATCTATTATCAGTGTCTGGGCATAAGTTATATGCACCAAAA
GGTGTTGGTTTTTTGTATCTAAGATCGAAACGACCAAAAGTTAGGCTTGTAAAGCAAATACATGGAGGGG
CTCAAGAGTTTAATTTACGCGCAGGGACATTAGCAACCTATCAGATATTTGCATTAGCTATAGCCGTTAA
AGAGATGTTTGCTAAAAAACAACAAAACTTTGAATATGTGTTAGAAATGCGTCGAGTTTTTTTAGATATT
ATTCAGGATTTGGCTAGTATAAAAATTAATACAGATCTTGAGAATAGTTACCCAGGTATTTTAAGTGTGA
CATTTTTAGGAGTAAAAGGTGAAACGCTATTAGCATTAGTTGATGGGGTTTGTATGTCAATGGGCTCAGC
GTGCAATTCTCAGGCAGTTGAGCCTTCACATGTGTTAAGTGCCATAGGTTTGACAGCAACTCAAGCAGAG
TCAACACTTAGAATATCTTTTGGGTTACAAACAAATAAAAAGCAAGTTATTCAAGCGGCAAATTTGCTAA
AAGAAAAAGTACAACTTTTACGAGCTTTATCTCCTCAAGGAGAAGTAAATGTATAA

>lcl|NC_006570.2_cds_YP_170185.1_1064 [gene=rne] [protein=ribonuclease E] [protein_id=YP_170185.1] [location=1243633..1246290]
ATGAAAAGAATACTAATAAATAGCAAGAGTGGCGAAGAAACAAGAATTGCCACCCTAGATAATGGCAAAT
TAATAGATTTAGACATTGAGAGCGTTGATAGAGAACAAAAAAAAGCAAATATTTACAAAGGTTATATTTC
AAGAATTGAACCAAGTTTAAATGCTATATTTGTTAACTATGGTGAAGAAAAAAATGGTTTTTTACCATTT
AAAGAAGTATCAGAGTATTATCTAAAAGATGTTCCAAACGGTGAAAATATCGCACCTTTGCTTTCAGAGG
GGCAAGAACTAATTGTTCAAATAGATAAAGAAGAACGAGGCGATAAAGGTGCAGCATTAACTACATTTAT
TACCTTGGCTGGTTCGTATATGGTGTTATTGCCAAATAATCCTGAGGGGGGTGGTATTTCCCGTCGTGTC
GAAGGTGAAGATAGAGAAAGATTAAAAAAATATCTCAAAGAATTAAATATCCCAAAAAAATATGAGCGTAA
TAGCAAGAACTGCTTGCGTTGAGTGCTCTTTTGAAGAATTAAAACATGATTTTGATACTCTAGTTGAATT

ATGGGGATCTATTTCACAAGCTTACCATAGAATCAAAAAACCAGCTTTATTACATAAAGAAAGTGACATT
ATTGTAAGGACAGTTAGAGATCACTTAAAAGAAGATGTTAAAGAAATCATCGTTGATTCTAAAGAGTGCT
TTGAAGATGTCAAAAGACAGCTTAGTTTACTTAGACAAAGTTTTGATATAAACAAAGTAAAACTATACAA
CGAAGAATTACCATTATTTGCTCAGTTTGGTATTGATCAACAAATAGAAAATGCTTATAAAAGAGAAATT
CGCCTACCATCCGGCGGTTCTATCGTCATAGATACTACAGAGGCACTTGTAGCAATAGATGTAAACTCAT
CTCGCGCTAACAAAGCCGAAGATGTAGAAACTACAGCATTTAAAACTAACCTAGAGGCTGCGGAAGAAGT
TGCTCGTCAACTTAGAATTAGAGACTTAGGTGGTCTTGTTATAGTTGACTTTATTGATATGTCTTTCTAC
CCGAATCGAAAACAAGTCGAAGAAAAACTAATGGAAGCACTACAACAAGATAGAGCACGCATCCAAATGT
CACGCATTTCTAAACTTGGACTTGTTGAGATCTCAAGACAACGTTTAAGTTCTTCAATTAATGAAAGCGT
AATGCAAAAATGCCCACGTTGTGAGGGTCATGGTTTTATCAAAACTACGCAAGCTACAGCACTTACCATC
CTACGTAAAATCAGAGCAGAAGCCATTAAAGAAGATACCAACGAAATTCGTGTCCAAGTTCCTGTAGACA
TCGCAGCATATATACTAAATGAAAAAAGAGATAGTATTGTTGAGATTGAGAGAATTTCTCAAGTTAAGGT
TATGATTATTCCTAACTTTAATATGGAATCGCCAAAGTTTCAAATGCAGAGAATCTGGGGTACTAGCTAC
AAATCAAATCGTACAAGCTCAGAATTAATAGAAGATGTTTATAATATTGAAATTCCTAAAGCTGGTAAGA
AAAAAATTGCGGCTGTGGATTTAAATCAAGCTATTGAAAATCAAGCTGCTAGCAACACTCAAGAACAAAA
GCCTCAAACTGATGCTCAAGTAGATATAGTTACACAAAATGATAAGAAACAAGATGATGTAACTCAACAA
GCTAAGAAAAAAGGCTTCTTTGCTCGGCTTTTTAGCGGTATATTTGCAACTGATTCAAAAGCTGCTCAGC
AAGCTAATAACTCAGCTCAAAAACAACAAAACAAAGAACAGAATTTCCAAAGGCAGCAACAACATAATAA
GGCGCAAAAAGATAATAATCAACGTAATGAGCGTAACAATAGCAACAACAAAAATGAACGCAATAAAAAT
AGAAACAATAAAGGAGCAAAACCACAAATAGCTCAAGAAAATAAACCACAAAAAGACAATAGCCAGCGTA
GTGATCTCAATGCTAATGGTAACAGTAACAATGATAAAAATGAGCGCAATAAAAATAAATCAAACTATGA
TAAGTTTGATAAAAAAATCAAATAACCGTAACAGCAGAAGCAATAATTTAAATCTAAATAATGCTGAAGAA
GTTATTGATATTACGCAACTTAAGTATCAAAATCAAACAGCTATTGCAAAGACAGATAATATCAAAAAGG
TGATTTCGAAGAATCCTGATGAGTTTATCTCTGTAATGGTTAAAGATGTCTTAGAAAACTATGATAGTCT
AAAAGACGATGGTGCTACAAAGATAGCTACCCAGGAGAAAATTAAAACTAACAAATACTTAAAGTTTGAT
ACTGTATCTGTAGATACTGAAATAGCTCTACTTAATGAAGTCGCTAAATCAAATCAAACTGTTGGAGTTG
AGCAAATCACAGCTGAAACAGAAGAATCTTTAAGTATCGTTGATAAAGTTGATGACATAACAATACCAGA
AGTTGTAGTAGAGCAAGAAAAGCCAGCGAAAGTAGTTAAAGAAACTAATCAAACTAGTGAAGCTAAACCT
AAAAAAGAAAAGCAAACTACTACAAAAAAATAATAAGAAAGAAAAACCAGCTGAAAAAGAGATGAAACAAA
ATCAAGCTAAACCTGAATATATAAACTATTCACCAGCGATTGATTTTGAATCTCAAGCACTAATATAG

>lcl|NC_006570.2_cds_YP_170186.1_1065 [gene=lgt] [protein=prolipoprotein diacylglyceryl transferase] [protein_id=YP_170186.1] [location=1246305..1247111]
ATGCTACAATATCCTCATATTAATCCAGTTGCATTGCAGCTTGGTCCTATCAAGATTCATTGGTATGGTT
TGATGTACCTACTTGGTATTTTTGCCGGCTGGTACTTAACTAGATATAGAGCAAAAGTTAAACCATGGGC
ACCTATCAAACCAGAGCAAGTTGGCGATTTAACTTTTTATGTTGCACTAGGTGTTATCCTTGGTGGTAGA
ATCGGCTATATTATCTTCTATAATCTTCCTTATTATTTCCATAATCCATCACAAATGTTTTTCTTATGGG
ATGGTGGAATGTCATTCCATGGTGGATTTATTGGGGTGCTTATAGCATTTGCCCTATTTGCACGTAAAAT
TGGTGCAAATTTCTTTGATTTGGGTGAGTTTGTTGCACCAGTTATACCAATTGGTTTAGGTGCTGGTAGA
ATAGGCAACTTTATCAATGGCGAGCTTTGGGGTAAAGTTACAGACTCTCCATTAGGTATGGTTTTCCCAA
CTGGAGGTCCTTTACCAAGATATCCATCACAGCTATTCGAGTTTTTCTTCGAGGGAGTTGTCTTATTTAG
TGTTCTATGGCTTGTTACCATCAAGAAAAGACCCCGTTATCTTGTACTAGGACTTTTCATGTTTTTATAT
GGTTGTGCTAGATTTATCTGTGAATTTTTTAGACAGCCAGACCCGCAGTATGGCTATATATTCTTTAATT
GGATGACTATGGGGCAAATATTATCAATCCCAATGATATTACTAGGAGCGGTAATTTTAATCGCAGTATT
TATTAAAATAAGGAAGAATAAATGCAAGAATATCTAA

>lcl|NC_006570.2_cds_YP_170187.1_1066 [gene=thyA] [protein=thymidylate synthase] [protein_id=YP_170187.1] [location=1247096..1247920]
ATGCAAGAATATCTAAATTTCCTTAAGTATATCAAAGAAAATGGCGTTTTAAAGGGTGATAGAACCGGTA
CTGGAACAAGAAGTATCTTTGGCTATCAAATGCGTTTTGATCTTCAAAAAGGCTTCCCATTAGTAACTAC
AAAAAAAATTCATATTCCTAGTGTTGTTCATGAATTATTATGGTTTTTAAGTGGTAGTACAAATATCAAA
TATCTTAATGATAACAATGTTAGAATTTGGAATGAGTGGGCAACTGTAGATGGCGAACTTGGTCCTATTT
ATGGCAAGCAGTGGCGTGATTTTAATGGTCAAGGTATAGATCAAATTGCCGATGTAATTCAGATGCTAAA
AACAAACCCAAACTCACGTAGGATTCTAGTTTCAGCGTGGAATCCATGTGTTGTTCCATCAGAAAAAATC
TCTCCACAAGAAAATGTTGTAAAAGGTAATTCAGCCCTACCACCATGCCATGCAATGTTTCAGTTCTATG
TAGCAAATAATAAACTATCATGCATGCTTACGCAAAGAAGTGCTGATGCTTTTCTTGGAGTGCCATTTAA
CATTGCAAGTTATTCACTACTGACAAATATGGTTGCACAACAATGTAATCTTGATGTTGGTGAGTTCATC
TGGTCAGGTGGTGATTGTCATATTTACAATAATCATATTGAGCAAGTCAATGAACAGCTTAGTCGTGAGC
CTTTAGCTTTACCAACACTAAAGATTTTAAGAAAACCAAACTCCATCTTTGATTATAAATACGAAGATTT
TGAATTTGAGAACTATAACCATCACCCTGCCATCAAAGCAAAAATATCTGTATAA

>lcl|NC_006570.2_cds_YP_170189.1_1068 [gene=mesJ] [protein=PP-loop family protein] [protein_id=YP_170189.1] [location=1249168..1250364]
ATGTCTATAAGCAAATCTCTTGTTTTAAATGAAATAAAAAAATTCTCGCCTTCTCATATTATTATTGGTT

ATAGTGGTGGCGTTGACTCAAGTGTTTTACTAAATATTAGCAAAGAATTAGATATTCCACTGATTGCAAT
TTATATTAATCATAATCTTCATCGTGACTCATTAAAATGGCAAATTCATTGTCAACAAACTTGTCAAAAA
TATAATCTACAATTTATTAGCCACTCATTAGATAAAGTTCCTAAGGGTGAGAGTTTTGAGGCTTGGGCAA
GTAAACAAAGAATGGCATTTTTTCAAAAAATAATGCAGCAGTACTCTAAGCCACTACTTCTATTAGGACA
TCACCAAGATGATCAAGCCGAGACATTCTTAATTCAAGCCATACGTGGCTCGGGATTAGCAGGCTTAGCA
GGTATCCCACATTATAAGGAACTTCATCATGGAGGTGTATTACGACCATTATTAAAATATAGTAAAATTG
AGATTGAAGGGTTTGCTAAACTAAATAATATTTCATATATATATGATGATAGTAATGAAGATATAAAATA
CCGCAGAAACTTAATTCGCAATCAAATAATCCCGATACTACAGCAAGTAAATCCAAATATCAGTCAAACC
CTCTCTCGTAGTGCAAATATTTGTGCAGAAAGTAATAATATTTTGCAAAAGCTCCTTACAGAAAGATTAC
AATCGATATCTCAAGATACTAATCTAATAATTAGCGAGTTAATAAAATTAGATGATGATATTCAAAAAAA
TCTTCTGCATCTTTGGTTTAAACAAAATACTCAACAAAGTCTTAAAAGTAAGCAAATAAAAGAGTTACAT
CTAGCTGTCAATAACCCCTCAACAGGTTGGCAAATCGATATAAGTAACTATTATCAAATTCACATCCAAT
ATAATCAGCTTATAATAAAGTATCCAACAACTATCAATGATATAAGCAAAGAAGATATCATAAGTTGGCT
TAGCAAAAATCTTAATGAAGAAATCGATCTAACCAAAATAGTAATTCGTGATAGAAAGCCTGATGATAAA
TGCAAATATCGTGGCAGAAATAAGCCTAATAAACTCAAAATACTCTTTCAAGAACTACAAATTCCCACCA
CTGAAAGATCAAAAGCAAAAATAATTTTAAAAGATCAGCAAATAATCGCTGTATATCCTTTTTTTATTTG
TGGGTAA

>lcl|NC_006570.2_cds_YP_170199.1_1078 [gene=glyA] [protein=serine hydroxymethyltransferase] [protein_id=YP_170199.1] [location=1260815..1262068]
ATGTTCAGCTTTGAAAAAAATAGCCTAAAAAATACCGACAAAGAGATTTTTGATGCTATAGAACTTGAAG
TTAAAAGACAACATGAACATGTTGAACTTATAGCATCAGAAAACTATGCAAGTCCTGCAGTAATGGAGGC
ACAAGGGTCACAACTTACAAACAAATATGCCGAAGGCTATCATGGTAAAAGATATTATGGTGGTTGTGAA
TTTGTTGATATCGCTGAGAAACTTGCCATAGAGAGAGCACAGCAACTATTTGGGGTTGATTATGCAAATG
TTCAACCACATTCAGGCTCTCAAGCAAATGCCGCTGTATATAATGCCGTTTTAAAACCAGGTGATACTGT
TCTTGGTATGGATCTAGGTGCTGGTGGACACCTAACACATGGTAGCAAAGTTAATTTCTCTGGAAAAATT
TATAATTCTATCCAATACGGCTTAGATGAAAATGGTGATATAGACTACAAGCAAGTAGCACAACTAGCTA
AAGAGCACAAACCAAAAATGATAATAGCCGGCTTCTCAGCATTTTCTGGAATAATTAACTGGCAAAAGTT
CAGAGAAATAGCAGACTCCGTAGATGCCGTACTTATGGCAGATATAGCTCATGTAGCAGGTCTAGTAGCA
GCTGGAGTATACCCTAACCCATTCCCATATGTTTATGTTGCAACTACAACTACTCACAAGACATTAAGGG
GACCTAGAGGTGGATTAATACTTTGTAATAATAATCCGGAACTTGCAAAAAAAATTCCAATCAGCTATTTT
CCCTGGTATTCAAGGTGGTCCTTTAATGCATGTAATAGCAGCTAAAGCAGTTGCATTTAAAGAAGCATTA
GAACCAAGTTTTGTAGACTATCAAAAACAAGTATTAAAAAATGCTAAGGCTATGGAAAAAGTTTTAAAAC
AACGTGGTATTAATATAATCTCTGGTGGAACTAGTAATCATCTACTTTTACTTGATATTACAAATACTGG
TTTCTCTGGTAAAGAAGCTGAGGCGGCTCTAGGTAGAGCAAATATCACTGTTAATAAAAACTCTATCCCA
AATGATCCCCGTTCTCCTTTTGTCACTAGCGGATTAAGGATTGGAAGCCCTGCTATAACGACGAGAGGTT
TCAAAGAGAAAGAATGTGAGTTAGTCGCTAACTTATTAGCAGATGTAGTATTTAATTGTGGTGATGAAAA
AGTTGAAAATGAAACTGCTGCTAAAGTTTTAGATCTTTGTGATAAGTTCCCTGTTTACAAGTAA

>lcl|NC_006570.2_cds_YP_170202.1_1081 [gene=yfiO] [protein=lipoprotein] [protein_id=YP_170202.1] [location=complement(1265424..1266248)]
ATGAAAAGGTTTTTATATTTAATAATAATTACATTTATGTTATTGCTATTGAGTTCTTGTGGTCCTAAAA
AGGATAGTGAGCTACCACAAGTCTACACAGGTTATACCGCTAGCTTTATTTATGCTAAAGCTCATGAGCA
AATGCAGAATCAAAAATATTTTGATGCAATTAGATCGTATAAGTCATTGGTGGCACAGTATCCATTTACA
CCATTGGCAGAGAAGGGTATGGTTGATTTGATATATGTTTATTATATGGATGATGAGTCAACTATGGCGC
TTGCATTAGGTCAACAGTTTATCAAGATGTATCCATATAGTATATACAAAGGGTATGTTTACTACATGAT
AGGTGTTGTAGGCTTTGAGGATGGAAGGGGTATGTTACAAACTTATGCGCCATATGATATGAACTATCAT
GATCCTACTGGATATCAAGATGCATATACTAATTTCGAAAAAGCTATTCAATTAGATCCTAATGGTAGTT
TTGTTCCAGATGCTAAACGCAGAATGGTATTCATAAATAATATTATCGCAAGGCACTATGATGATATCGC
TCATTTTTATTTTAAGAGAGGTGCTTATAATGCAGCTATTGATAGAGCTTCTCAAGTGATAAGAAATTAT
CCACAAAGTACATCAACAGAGGATGCTTTGGTCTTAACAATTAGAGCTTATAATAAGTTAGGCTTATATG
ATCAAGCTAAAGCAAATATTCGTGTACTTAAGAAAAATTATCCTAAAAATAAATTTATTAAAAACCTTCG
TCCGGATGGTACAGAAGAGCCAAGTTGGTATCAAAGATGGTTTGGCTGGTTATAG

>lcl|NC_006570.2_cds_YP_170225.1_1104 [gene=dnaK] [protein=molecular chaperone DnaK] [protein_id=YP_170225.1] [location=complement(1290709..1292637)]
ATGGGAAAAATAATAGGTATAGATTTAGGTACTACTAACTCTTGTCTTGCTATTATGGATGGCAAGACTG
CTAAAGTTATTGAGAATGCTGAAGGACATAGAACAACACCTTCAGTTGTGGCATATACTGATAGCGGTGA
AATATTAGTAGGTCAAGCTGCTAAAAGACAAGCTGTAACTAACCCTGATAATACATTCTTTGCTATCAAG
AGACTTATAGGTCGTAAGTACGATGATAAAGCTGTACAAGAAGATATTAAAAAGAAAGTACCTTATGCGG
TAATTAAAGCTGATAATGGTGATGCTTGGGTTGCTACTAAAGAAGGCAAAAAAATGGCTCCACCACAAGT
TTCTGCAGAAGTTCTAAGAAAAATGAAAAAAACAGCAGAAGACTATCTAGGTGAACCAGTTACAGAAGCT
GTAATTACAGTGCCAGCATACTTTAACGATAGTCAAAGACAAGCTACAAAAGATGCTGGTAAAATAGCAG
GTCTTGAAGTTAAAAGAATTATCAACGAGCCTACAGCGGCAGCGCTGGCATATGGTGTAGACTCTAAGAA

AGGTGAGCAAACTGTAGCGGTGTATGACCTAGGTGGTGGTACATTCGATATCTCAATTATTGAGATTGCT
GATGTTGATGGCGATAACCAAATCGAAGTATTATCAACCAATGGTGATACTTTCTTAGGTGGTGAAGACT
TCGACTTGGCTTTAATGAACTATCTAATTGACGAGTTCAAAAAAGAGCAAGGTATAGATCTTCACAATGA
TAAGCTTGCTTTACAAAGAGTTAGAGAGGCTGCTGAGAAAGCTAAAGTAGAATTATCTTCAGCACAACAA
ACTGATGTTAACCTACCTTACATCACAGCAGATGCTACTGGACCTAAGCACTTAAATATCAAAGTAACTA
GAGCTAAGTTTGAGTCTTTAGTTTCTGATCTTGTAATGAGATCACTTGAGCCTTGTAAGAAAGCTCTTGA
AGATGCTGGTTTAAGTAAGTCTGATATTACAGAAGTATTACTAGTGGGTGGACAAACTCGTATGCCTCTA
GTACAAGAGAAAGTAAAAGAGTTTTTTGGTAAAGAGCCACGTAAAGATGTGAACCCTGATGAAGCTGTTG
CAGTTGGTGCGGCTATTCAAGGTGGTGTATTAGCAGGTGATGTTAAAGATATTCTTTTATTGGATGTAAC
ACCGCTTTCTCTAGGTATTGAGACTATGGGAGGTGTTATGACTAAGCTTATCGAGAGAAATACTACGATT
CCTACTAAGAAGTCGCAAGTATTCTCAACAGCTGAAGATAACCAGCCTGCGGTAACTATTCATGTACTTC
AAGGTGAGCGTGAAATGGCTTCTGCAAACAAATCTTTAGGTAGATTTGATCTGGCAGATATTCCACCAGC
GCCACGTGGTATGCCACAAATTGAGGTTACTTTTGATATAGATGCTAACGGTATATTAAATGTGTCTGCT
AAAGATAAAGCTACTGGTAAAGAGCAAAATATTGTGATTAAGTCTTCAAGTGGTTTATCTGAAGAGGATA
TCGAAAAAATGGTACAAGACGCTGAAGCTAATGCAGAAGCAGATAAAAAGTTCCATGATTTAGTTACTGC
TAGAAATACTGCTGATAACTTAATTCATAGCTCAAGAAAAGCAATTCAAGAACTGGGTGACAAAGTAACA
GCAGCAGAAAAAGAAAAAATCGAAGAAGCTTGTAAAGAGCTTGAAGCAGCAACTAAAGGTGATGATAAGC
AAGCGATTGAATCTAAAACTAAGGCTCTAGAAGAAGCATTTGCGCCAATAGCTCAAAAAGCTTATGCTGA
GCAAGCTCAAGCTGCTGTTGCCCAAGGTGGTGCTAAAGCTGAAGAACCTAAGAAAGAAGAAGATGTTGTT
GATGCTGACTTTGAGGATGTTGAAGACGACAAAAAATAA

>lcl|NC_006570.2_cds_YP_170226.1_1105 [gene=grpE] [protein=heat shock protein GrpE] [protein_id=YP_170226.1] [location=complement(1292738..1293325)]
ATGAGTAAGCAAGAAAAAAGTAATGTAGAAGATAAAAGTCTTGATATTGAGACAGCAGCACAAGTAGAAA
CAGCACAAGAATCTGCTAGTGGAGCTTTAGAAGAATTATCTGTAGAAGAACAGCTAGAAAGAGCAAAAGA
TACTATAAAAGAACTTGAAGATAGTTGTGATCAATTTAAAGATGAGGCTTTAAGAGCGAAGGCTGAAATG
GAAAACATTCGTAAAAGAGCTGAAAGAGATGTATCAAATGCACGCAAATTTGGTATAGAGAAGTTTTCTA
AAGAGCTTTTGCCAGTAATTGATAGTATTGAGCAAGCATTAAAGCATGAGGTTAAGCTTGAAGAAGCTAT
CGCAATGAAAGAAGGTATTGAGTTAACAGCAAAAATGCTGGTTGATATACTTAAGAAAAATGGTGTAGAA
GAGTTAGATCCAAAAGGAGAGAAATTTGACCCTAATCTACATGAAGCTATGGCAATGATTCCTAACCCTG
AATTTGAAGATAATACTATTTTTGATGTTTTTCAAAAGGGTTATATGTTAAATGGTCGTATTGTTAGAGC
TGCAAAAGTTGTTATAGTAAAAAATTAA

>lcl|NC_006570.2_cds_YP_170227.1_1106 [gene=mltA] [protein=membrane-bound lytic murein transglycosylase A (MLT) family protein] [protein_id=YP_170227.1] [location=1293414..1294580]
TTGGTATTTTTTTATATGGATATTAAAAAGTAAAAGAAACCTTAAAAAAAATAGTTATCATAGTTAGTT
TAATACTTATAACTGGGTGTGCTAATATTACTGCGCAACGATATTTATCAAAAAAATATTCAAAAAATAT
AGATTATAAAAAATCAAGCTTCGAAGACTTAGCCAACTGGGATAGTGCTGATCAGCTTGAGTCTTTTAAT
ACTTTTAAAAAATCTTGTGAAAAGATATTAGAAGAAAATAAACTAGAATATTCAAACTGGATAAATATCT
GCCATAAGGTTATAAATACAGATTTAAAAACTAAACAACAAGCTAAATTATTTTTTGAGCAGAATTTCAC
TCCATACCAAATAATATATAAAGGAAAAGATACAGGTTTATTTACAGGCTATTATGAACCTTCGATGAAA
GGTAGCTTAGTCAAAACTATGGAATATACCGTACCTATCTATCGCACACCTGATAACTTAGTAAAAAAAC
CTAAAGATGATGATAGTTTTTCTTTCGGAATGTATCAAGATGGTAAATTTGTTCCTTATTACTCACGTGA
AGAAATATCAAAAGGTGATCTACTTCCAAAAAAAGATGTTTTAGTATGGGTAAAATCAAAAGTTGACCGA
ACATTTTTACAAATCCAAGGCTCTGGTCGCATCGAAACTGACAGTGGTGACATTCTTATAGGTTACGATA
GCCAAAATGGTCATGAATATAAGCCCATAGGTAAATATTTACTTGATCACGGTTATATGAGTGCTACGCA
AATGTCTATGCAAGCGATAAAAGCATGGCTAGATGAGAATAAAGATAAAATCGATGATGTACTAAACTAT
GACCCTTCTTTTGTGTTTTTTAGATACATTGATCGCAAAAAATGCTGTTGGTGCACAAGATGTTGAACTAA
CACCTGGATATTCTCTTGCTGTTGATAACAAATACTATCTGTATGGCGTACCCTTATGGTTAGAGACAGA
TTATTTTGCTGATAATCATGATGATACAAAACCTCTTGATAGGCTAATGATAGCTCAAGACACAGGTGGT
GCTATAAAGGGCGCTATCAGAAGTGATGTATTTTGGGGACATGGTAAGCAAGCAGAATTTAACGCCGGCC
ATATGAATAACCGCGGCAAACTATGGATACTTTTACCAAATGACTGA

>lcl|NC_006570.2_cds_YP_170228.1_1107 [gene=FTT_1272] [protein=hypothetical protein] [protein_id=YP_170228.1] [location=1294573..1295757]
ATGACTGATACAAAACATTTTGATACTATTATAATTGGTGCTGGAATTTCAGGTATATCTTTAGCACAAC
GATTAACTGCTGCATCTGTAAAAAAATTGTGTCTTTGAGGCTAATAAGGTCGGCGGTTGTATTGACTCTCA
ACAATACGAAAATTTTTGGTTTGAGATGGGAGCACACACCATATACAACTCTTACAATAAAACTATAGAA
TATATCCACAGTAACTCTCTTGGAAAAGATATCCAACCAAGAAAAAAGCTACCATTCTTATTTGTTCAAC
CAAACAATAAAATTCAAAGTATTTTTATAAATATTAATCCATTTACAGCAGCTCTTAGTTTTTTGAAAAA
CAGAAAAGTCTCAAAACAAGATAAAACTGTTAGCGAATATGCCACAAAGCTATTTGGCAAAAAAAAATTAT
AGTAAAACTTTGAAGTATTGTTTTGACGCTGTACTATCCCAAGATAGTCAAGAGTTCCCGATGGAGTATT
TATTTAAGAAATATGATAGAGATACGACACTGCCACGAAGTTTTACTCTAAAAAAATGGTTTAGCTGAACT

TTTCAAAAACCACAACGAAAATGTTATTAAAGAGACTGTCATAAAGATAACAAAACAGCAAAAATGGCAC
ATACATACAAAACACGGCGAATATACTTGTGAAAATCTTTGTTTGGCAACACCATGGAATGTAACCGAAT
TACTATTAGAAAAAATATTACCTAATATAGCAAAACATCAATATAGACCTACAATGTCGAATCTAATAAG
CGTAGGAATTGTTACAAATAAATCAAGCTTGAAACATATTAAAAACTTAGCTGGCTTAATCGGTAAAGAA
CAATTTTTTTACTCAACAGTCTCTAGAGATGTGATAGATAACCCAAACTATAGGGCAATTGTTTTTCACT
GTCGTGATGAATTTTCTCAAGAAGAATTACTTGATAAAATTATAGAATTACTAAAAATAAAACCAGATCA
TATCATATACACTTATACAAAAAACAATACACTACCTTGCTATCATCGTAAGCATAGTGCTTTTATAGCA
GATCTTGAAAAAGAATTACAAAATCAGCCAAATCTATATATTAGTGGTAATTTTTTTGACAGACTAGCTA
TAGAAAATTGTATTAGAAGGTCAAATGAACAAGCAGGTAAAATTATTCAAAATAAAATCCCTTAA

>lcl|NC_006570.2_cds_YP_170229.3_1108 [gene=rplM] [protein=50S ribosomal protein L13] [protein_id=YP_170229.3] [location=1295869..1296324]
TTGACAACAATAAATAAAGGAAAAAAGATGAAAACGTTTACTGCAAAACCATCAAATATCAAAAGAGAAT
GGCTTTTGATTGATGCTACAGATAAAACTTTAGGTCGTCTAGCTACTGAAGTAGCAATGATCCTAAGAGG
AAAAAATAAACCAGAATATACTCCTCATATGGATACTGGTGATTATGTTGTTATCGTAAATGCTGAAAAA
GTAGCTGTAACTGGTAACAAAAGAAAAGCAAAAACTTATTACCATCATACTGGTTATATTGGCGGTATCA
AATCAGTATCTTTCGAGAAGCTAATAGCGACTCATCCAGAAAGAGCTATTGAAAAAGCTGTTAGAGGGAT
GCTTCCTAGAACTCCACTAGGTCGCACTATGTTTAAGAAGTTAAAAGTTTATGCAGGTGAAGCTCACCCA
CATACAGCTCAACAACCTAAAGCTCACAATATTTAA

>lcl|NC_006570.2_cds_YP_170230.2_1109 [gene=rpsI] [protein=30S ribosomal protein S9] [protein_id=YP_170230.2] [location=1296340..1296729]
ATGTCAGAATATAATTATGGTACAGGTCGTCGCAAAAGTTCTGTAGCTCGTGTATTTATGAAAAAAGGTA
CTGGTCAGTTTATTGTTAATGGTCTACCATTAGAGCAATACCTATGTCGTGAAACGGACTGCATGGTTGT
AAAGCAACCTTTAGAACTAACTAACAACACTGATAACTTTGATTTCAAAGTAACTGTAAAAGGTGGTGGT
ACTACTGGTCAAGCTGGTGCTATCCGTCTTGGTGTTACTAGAGCTCTAATTGAGTATGATGAAGAACTTA
AACCAGCTCTTCGTGAAGCAGGTTTTGTTACTCGTGACCCACGTAAAGTTGAGCGTAAGAAATTTGGTCT
TAGAAAAGCTCGTAGAAGAAGACAATTCTCTAAGCGTTAA

>lcl|NC_006570.2_cds_YP_170231.1_1110 [gene=mglA] [protein=macrophage growth locus subunit A] [protein_id=YP_170231.1] [location=1296789..1297406]
TTGCTTTTATACACAAAAAAAGATGATATCTATAGCGATATAGTCCGCATGATCCTTCTTATTAAAGGAG
CTAATGCGAAAATTGTAGATGTTTCTAAAGAAGAAAATTCAAAACATCTAGAAGAGCTAAATATCATTAC
ACCTAATGGTAATATACCTACGCTTAGCACAGATGATTTTGCAGTGTATAGGCTTAGTGTGATTATAGAA
GCTATAGAGGATCTATATCCCTTTCCTCCGATGTTTCCAGTATTTCCAAAACAGCGAGCTAATGCAAGAA
TATTGTTAGAATATGTTAATAAAACGTTTCTGCAAAATATTATCAAATTACAAAGCCCTGATTTGGATGA
AAAACAAGCTAACGAAATAAAAATGCTAATGCAAAGGGATATAATAAGCACTTATAAGAAAATAGTTAGT
GAAAGAGAAGTAAATGCAGAAAGTAATCCAGATGCTCAAAATATAAATGTATTGACTCTGATAATAACTT
TCGTTTTTTATTATTTCATTAAATTAAAGATCTCAATACCTACCAAAGATAAAAACATTATCAAAGAGAT
CAAAGAATTACTTAGCGAACCTAACTTTATAAAAACTATCAAAGCAAAAGGAGCTTAA

>lcl|NC_006570.2_cds_YP_170236.1_1115 [gene=ptsN] [protein=PEP-dependent sugar PTS system family protein] [protein_id=YP_170236.1] [location=complement(1300377..1300832)]
ATGAATTTAAAAGCTCTAATTGATAAAAAAAATATTATCTTAAATTTGAATATTGAGTCTAAAAAACGCT
TAATCGAGTTTTTTGCAAATAGAATAGCAGACACTTACCCTGATGTGAGTGAAGATCTTGTGCTTAAAAA
TATTTATAAACGCGAAAGAATAGGTAATACTTATATTGGTAAAAATATTTACATTCCTCATTGTCGCGTT
GAGAATCTTATGACTACTAGGTTGATTATCGTGACATTAAAGAATAGTTACTATGATGATTCTGTCAACG
ATGATATAAAGATAGCAGTTGGTGTTTTTTTTCCTGACAATATATCTACAATCCATATGGAACTGCTAAA
GCAATTAGCTTTATATTTGAAACAAGATAAAACGCAGCAGTATTTTCAGCAAGCAGAAAATTCCGAAGAT
TTGTATAATTTAATTATTAATACTAGTAATGAATAA

>lcl|NC_006570.2_cds_YP_170237.1_1116 [gene=yhbH] [protein=sigma-54 modulation protein] [protein_id=YP_170237.1] [location=complement(1300869..1301165)]
ATGAATATTCAAATTACTGGTAGACATGTGGAAGTTACTGATTCAATTAAAAACTATGTTAATGAAAAAG
TAGGTAAGGTTGAGCACTATTTTGATAATATCACTTCGACTAAAGTTATATTAGATGTTGAGAAAGATCA
TCAAGTAGCAGAGGCTATAGTTACAGTTCCTGGTAGTGAATTTGTTGCAAAGGCTGAAGACAAAGATTTA
TATGCAGCTATAGATATGCTTGAAGATAAGTTAGCACGTCAGTTAAAAAAGCACAAAGATAAAATGAGAT
GTAATCACGGCGAATAG

>lcl|NC_006570.2_cds_YP_170239.1_1118 [gene=trmE] [protein=tRNA modification GTPase TrmE] [protein_id=YP_170239.1] [location=1301535..1302887]
ATGTACACAAAAGATACAATAGTTGCAATAGCAACTCCTCAAGGTAATGGCGGTATAGGCATAATCCGAA
TATCTGGGATAGATGCTCTAGCAATAGCTGAGAAACTAACTAAAAAACAGCTAAAACCTCGTTATGCAAC

ATTCTGTAATGTCTATAATGATAATGAAATAATAGATCATGGTATCATAATATTCTTTAAGGCGCCTTTG
TCATATACAGGTGAAGACGTTGTAGAAATTCAAGCTCATGGTAACCCGTTTATATTGAATTTGATTATCA
AAGCAGCACTTAACTGTGGTGCCAGAATGGCAAAGGCAGGTGAATTTACTGAGAGAGCATTTCTGAATAA
TAAACTTGATTTAGCTCAAGCAGAAGCTGTTGCTGATATTATTAATGCATCATCAGAAATAGCAGCTAAG
TCGGCAGCTAAATCACTTCAAGGAGATTTCTCTAAAGAAATAAATAATCTTTTAGAAAAACTTATTTATC
TAAGAATGTATGTAGAAGCGTCTATTGACTTTCCAGAAGAAGAAATAAACTTTTTAGAAGATCAAAAAAT
TCATTCTAGTTTAGAAGAAATATATAAGGTTATCTTAGCGGTAAAAAATAGCTGTAAACAAGGTGTTATT
CTTGCTGAGGGAATTACATTAATATTAGTAGGCAAACCAAATGCTGGTAAGTCAAGCCTTTTGAATGCTC
TTGCAGGTAAAGAATCAGCTATTGTAACATCAATAGCAGGTACAACTAGAGATATTGTCAAAGAACATAT
TCAAATAAATGGCGTTCCTATGCATATCATTGACACTGCAGGGCTACGTAATAGTGATGATATCATCGAA
AGTGAAGGAATCAAAAGAGCTATTAAGAAAATTCAAGAAGCTGATCAAGTACTATTTGTAACTGATGACT
ATACGAATAGCCAAGTTAAATTTAGTGATATAAAAGAGATAATTCCAGAATTTTATGATCAAATTCCTAA
AGATATCGATATTACATATGTACATAATAAGATAGATCTCCTTAAAGAAGTTCCACACAATCATGCTAAC
CACATATACATATCAGCCGAAAATAACATTGGTATTGATAAACTAAAAGAACATATTCTCAATAAAGTTG
GTTATACAAATCAAAATGAAAGTATTTATACTGCACGTGAAAGACATGTTACAGCGATAAATAATGCTTT
TGAGCATATTAAACTTGCTAAAGAACAATTAGAACTTGGTAATGGTGAGCTTCTAGCTGAAGAGTTGTTA
ATTGTTCAAGAATATCTTAATTCAATTACAGGTGAGTTTAGTTCTGATGACTTATTAGGTGAAATATTCT
CAAGCTTTTGTATCGGCAAATAA

>lcl|NC_006570.2_cds_YP_170242.1_1121 [gene=cbs] [protein=cystathionine beta-synthase] [protein_id=YP_170242.1] [location=1305421..1306344]
ATGCTATCTTTAATAGGAAAGACTCCTATAATAAAACTTAAAAATATAATAAACAATCATAATCTTTATG
CTAAGTGTGAATGGCTAAACCCAACTGGTAGTATCAAAGATCGTGTTGCGAAATATATCTTGGAAAACTT
AATAAAAAATAAAAAAATAAAACCTCAACAAGCAATTATCGAAGCAAGTTCAGGCAATATGGGAACATCT
TTAGCTGCAATTGGTAAATTATACAAACATCCTGTGTACATTACATGTCCTGAGAAAACAGGACAAATAA
AAAGAGAAATGATCAAAAGTTTTGGAGCAAACCTTACAATTTGTAAAAACACATCAGATCATACGGATCC
AGATTTTTATGTAAATAAAGCTAAACAATTAACAGAAGATTTAGATGGAATATTAGTTAACCAATATGAT
AATTTATTAAATACAGAGTGTCATTACAAAACTACAGGTCAAGAAATAGTGGACTATTTTTAACACAAA
ATGTTGATATTGACTACTTTATAACGGTTGGGGGATCTGGCGGCACTATTACAGGATGTGCAAAGAAGAT
AAAAGAGTTTTTTCCAAAAACACAAGTTATTATGCCAGACCCTTATGGATCTGTTTATTATGATATTTTT
TATTATGGAGCACCTATTAAAGAAAACATACATAGCTATAAAGTAGAAGGTCCTGGTAACCCTGTCTTCT
GTAAATCAATGGATTTTGCATATATTGACGAAATAATACAATTTAGTGATAATCAAGCAATACAAGCATG
TCATGAGTTAGCTAGTGAACAAGGTATTTATGCTGGACATAGTAGCGGTGCTAACTATTTCATTGCTAAA
AAACTACTCGAAAAATTACCACAACATCAGAGCTATAACATCCTTATAATGGTATTAGATAGCGGTATGA
AATATGCTTTTTAA

>lcl|NC_006570.2_cds_YP_170245.1_1123 [gene=metG] [protein=methionyl-tRNA synthetase] [protein_id=YP_170245.1] [location=1314025..1316049]
ATGCGAAAGATACTCGTTACTAATGCTCTGCCATATGCTAATGGTGACTTACATTTAGGTCACATGCTCG
GCTATATACAATCCGACATCTGGGTCAGATTCCAAAAACTACAAGGCAATCAATGCATATTTGTCTGTGG
TAGTGATACTCATGGTACGCCAATAATGCTCAAAGCCAAGAGTCTTGGAATTACTCCAGAAGAGTTAGTT
ACAAAATACTCTAATAGACACTTACAAGATTTTACTGATTTTGAAATTAATTTTGACAATTATCACTCTA
CTCATAATTCTCTAAATAAAGAGATTGTTGAAGATATATATAATAAACTTAACAATAAAAATCTTATTTC
AAAAAAAGCAATAGCTCAAGCTTATGACCCTGAGGCTAAAATGTTTTTGCCAGATAGATTTGTCAAGGGA
ACTTGTCCAAAATGTAAAGCTGAAGATCAGTATGGTGATAGCTGTGAAGTTTGTGGTGCCACATATGATC
CTACAGAGCTTATCAATCCAAGATCTGTAATATCTGGTCAATCGCCAATACAAAAAAACTCTGAACATTT
CTTTTTTGACTTACCCGCATTAGAAAAAAATATAAAAGATTGGATAGAATCTAATACACTCTTACAACCA
GAAGTTGCTAACAAACTAGCAGAATGGTTTGAGCAAGGCTTACAAAGTTGGGATATCTCGCGTGATGCGC
CATATTTTGGCTTTGCAATCCCAGGTACAAATGAACAAAAATTCTTCTATGTGTGGCTAGATGCTCCAAT
GGGCTATATTGCAAGCTTCAAAGATTATTGTAATAAGAATAATATTAATTTTGGTGATTTCTGGGGAGAT
AGTTCTAGTGAAAGTGAGCTTTATCATTTTATTGGTAAAGATATTATTTATTTTCATACACTATTTTGGC
CAGCAATATTATCATCTACTGGTTATAAGACACCTACTAGTGTATTTGCTAATGGTTTCTTAACTGTAAA
TGGTAAGAAGATGTCAAAATCTCGAGGGACGTTCATTCAAGCTAGAACTTACCTAGATAATTTAGAGCCT
AGTTATTTAAGATACTACTTTGCGTCAAGATTGACATCCCGCATTGATGATATAGATTTGAATCTAGAGG
AATTTGTTACTAAATCAAATTCTGATATAGTAGGTAAGGTAGTCAATATTGCCAGTCGTTGTGCTGGATT
TATATACAAAAAATTTGATGCTACCTTGTCTGGAGAGATTTTTGATCCAGAATTAGAAAGTGAATTTAGT
AAAAACCACGATGCTATAACACAAGCTTTTGAGAAAAGAGAGTTTGCCCACGCTGTGAGACTGATAATGG
CATTAGCCGATAAAGCTAATCAATTTATAGATTACCATAAACCTTGGCAACTAGCTAAAGAAGAAGGACA
AGAACAAAAAGTTCATCAAGTCTGTTCACAAGGTATAAATATGTTTAAGGTTTTAATCGTTTATCTTAAG
CCTATTATTCCTAGCATTGTTGCTGAGGCAGAGAGATTTTTAAATATACAGTTCATAAGCTGGGCTGATG
CTCCAAAGTTTTTAATAAATCATAAAATAGATAAATTCAAACCTCTAGCCACTCGTATCGAAAAAGAAAA
AGTAGACAAAATTTTAGAGGATACAAAGAAAATGTTAGAAAATGAACAATCTCCGCAATCTAAAAAAGAA
GAGCCTAAACTAGATATCGCAGCTGAATGTACTTTTGATGATTTTATGAAAGTAGATTTGCGTATAGCTA
AAATTACTGAAGCTTCACATGTTGAAGGTGCGGATAAATTACTTAAACTAATATTGGATCTAGGTGGTGT

CACCAAACAAGTTTTTGCTGGTATCAAGTCTGCTTACAAACCTGAAGATTTAATTGGCAAGCATACTATA
ATGGTAGCAAATCTAGCTCCTAGAAAAATGAAATTTGGAATGTCTGAGGGCATGGTTTTAGCTGCTGGAG
ATGGTAAAGGTATATATATACTTGAGCCACATGAGGGTGCCCAACCAGGTATGCGTGTCAAATAA

>lcl|NC_006570.2_cds_YP_170251.1_1129 [gene=ubiE] [protein=menaquinone biosynthesis methyltransferase] [protein_id=YP_170251.1] [location=1320592..1321344]
ATGTCTAAAGAAAATAAAACAACAGATTTTGGTTTTACACAAGTGCCTTGGGAAGAAAAACAAAAAAAGG
TAGCTGGGGTATTCCATTCAGTAGCAGCTAAATATGACTTGATGAATGATCTAATGTCATTTGGTATTCA
TCGCATCTGGAAAAAACAAACCATTGCTAAATCTGGAGTTCGTAAGGGTGATAATGTCTTAGATCTTGCT
GGCGGCACAGGTGACTTAGCATATAAATTTTGTCAAATGGTTGGTCAACAAGGTAAAGTAATCTTGAGTG
ATATTAACTCCTCGATGCTTGAAGTAGGCAAAGAAAAACTTACAAACAAAGGTTGTGTTGGCAATATCGA
ATATGTCCAAGCTAACGCTGAGTGTCTACCTTTCCCTGATAATTATTTTGATTGTATTACGATATCATTT
GGACTTAGAAATGTTACAGACAAAGATAAAGCGTTAGCATCAATGTGTCGAGTTCTAAAACCAGGTGGTC
GCTCGTTAGTTCTAGAATTCTCTAAGCCAATCATACCTTTACTTTCTAAAGTTTATGATGAATACTCTTT
CAAAGCACTACCATTTTTAGGTAAAATTATCACACAAGATGCCGAAAGCTACAAATACCTTGCTGAATCA
ATCCGCAAACATCCTGATCAACAAACACTAAAACAGATGATGTACGATGCTGGATTTGATAACGTCGAAT
ATCAAAATATGACCGGTGGAATTGTAGCATTACATATTGGATATAAATATTAA

>lcl|NC_006570.2_cds_YP_170252.1_1130 [gene=FTT_1297] [protein=hypothetical protein] [protein_id=YP_170252.1] [location=1321328..1321933]
TTGGATATAAATATTAAGATGCTAAAACTAGTAAACACAGCCTTAAGTCTTCTGCCTAAATTTGATCCTC
AGGTAAGTGCTCTACTGTTACCCATAAATGGTAAAGCCCTAAGTGTCAATATTACTGATATTGACTTAAT
CATTACATTAGAAGTAGAAGATTCTAAAATATATGCTAGCAACGAACTTACTAAAAACATTTTAAAAGGT
AAATTGGCTTATATTTTGGAGCTTATTTTCAATAAAAATCTACAAGAGTTAATAATAGCTGAGAAACTTG
ATTATCAAGGTAGTCTCAAAGATCTTAATGCTTTTAATAAATTTTTGAATGCTATTGATATAGATCTAGT
TTATAGAATATCTGAACTCACCAGTCCTGAATTTGCTGGAATTGTAGCTAAACCTTTTCAAAAAGCAAAA
CAATACTTAAAAACATCTCGACAAGAGACTATTGTCGATATCAAAGATTTCCTAACAGAGGAGAAAAAGA
CACTGATATCTCAAAATGAGATTAATATCTTTTATCGCCAAGTTCAAGAGCTAAAGCAAGCTACTGATAG
AATAGAAGCGAAGCTAAAATTATTAGAAGGCTTAAACAATGATTAA

>lcl|NC_006570.2_cds_YP_170253.1_1131 [gene=ubiB] [protein=2-polyprenylphenol 6-hydroxylase] [protein_id=YP_170253.1] [location=1321926..1323584]
ATGATTAAAAAATTTCTAAGGCTTATCTACATTTTTTATGTTATAAACAAGTACTGTCTACTCAATGAAC
CTATCAGAGCGACAAAAATAAAAACTCTAAGAGCCTTATTATTGCTAAATCCATTTTACTACTCTCGAAG
AGTACGCAGACTTGAGCATGGTGTGCGTATTAGAGAGGCTTTAGAAAAACTTGGACCTATTTTTATCAAA
TTCGGTCAAGCATTATCAGTTAGAGCCGATTTACTACCGCCAGATGTAATAAAAGAAGTTTCAAAACTTC
AAGATAACGTTCCGCTATTTGATAATAAAATAGCGGCTGAACAAATTGAAAAAGCTGCGAAAAAACCTAT
CAATGAGATTTTTAAAAGCTTTGAAAGTTCTCCATTAGCTTCGGCATCTGTAGCTCAAGTGCATGCTGCT
ATATTGCAAAATGATGATAAAGTAGTAGTCAAAGTATTACGTCCAGGCATAGAGAAAATTCTTAAACTTG
ATACCTCTTTGATGCTATTTTTTGCAACTTTACTTAGTAAACTTAAAGAAATAAGAAGATTTAAGCCCGT
AGAGATTGTCAAAGAGATAAACCAAAGCTTTTTTGATGAGCTTGATCTAGTCCGTGAAGCATCAAATGCT
TCTCAAATTCGCAGAAATTTTGAAGACTCTGCCATACATTATGTACCAAAAATTTATTGGGAATATACTA
GTTCTACTGTGATGGTGATGGAAAGAGTTGGTGGTGTAAGAGTTTCAGACATAGAAACCCTAGATGCTTT
AGGAGTTGATCGTCGTCTATTAGCACAGCGCGGTGTAGAAATTTTTTATTCACAAGTATTTGATGACTGT
TTTTTTCATGCTGATATGCATCCTGGTAATATGTTTATTGATGTATCAAATCCTGCTGATCCTAAATATA
TTTCAATAGACTTTGGTATCGTTGGCACTCTTAACCGCGATGATCAAAGGTATCTAGCTGGTAATTTTCT
AGCATTTTTCAAAAGAGATTATCGCAAGGTTGCAGAGCTTCATATAGAATCTGGCTGGGTTCCAAGCGAT
ACGCGTGTTGATGTACTTGAATCAGCAATTAGAACAGTTTGTGAGCCAATCTTTGAAAAACCTATGAAAG
AGATATCACTAGGGTACACATTGATGCAACTTTTTGCAGTAGCTCGTCGTTTTAATATGAATATTCAACC
TCAACTCACACTTCTACAAAAAACTCTTTTTCATGTAGAGGGACTTGGACAAAAACTTTGCCCTGAATTA
AATATTTGGGAAACATCCCGACCAATCCTAGAAAAGTGGATGAAGGAGCAAATGGGACTAAGAGGTTTCT
ACCATCGTTCAATGGAAAATATGCCTAGAGTTAGTGATAAACTACCAGAATTACCACGTATGGTTTTTGA
TATATTACAACAAACACAAATCAATCTAAAAAATACTACTACATCTACACAATTTAATAACTTAAAAGAA
CCTAAGAAAAAATATCGATTTGCATTAGGCTGTGGACTAGTTTTGACAACTATTGGAGTAATATATACTT
TGAATAAAGATACAACTCCTTTAATTAAACTACAAAACTTTATTAGTAATTATAGTACTAGTTTTATAAT
TGTTGGAGTTGCATGCTTGATTTACTATAGTTTTAAAAAGGAGAAATAA

>lcl|NC_006570.2_cds_YP_170256.1_1134 [gene=FTT_1302] [protein=hypothetical protein] [protein_id=YP_170256.1] [location=1325075..1325905]
TTGATGATTTTTGTTATAATCTTCATACCTAATTATATTAAGACAGACTCTTATATGTTTAATTATAATC
TTTTTCTTAATAGACTATCAAAGAGAAAATTATACCCTAAATCATTTATAAAAAATGAAATTGCTCAAAG
ACTACTTAAGCGTTTGGAGTTTATAAAACTAGATCCTAAAGATATCTTAGTCACTGGTTATAGTGATAGT
GATTATCTTGAGAGATTACAAAAGCGCTTTCCTAATGCTGATATTCATACCAGTCAAAACCTTAAACAAC
ATTTTGATATTATTTTCTCAAACTCAATTATTCACATAACAGATAATTTATCGCAAGAACTAGATGATTA

TTATCAACTACTTAACGATAATGGTATTTTACTTTTTTCGACTTTTGGTGATAAATCATTTGCGACTCTC
AAAGAGGCTGTTACAAGTGTTAGTAATTATAAGCATACTAATACTATGATTGATCTTCTGACTTGGGGCA
ATACCCTACAAGCAAGTCAATACAAAACTCCAGCGATTGAGTCAGACCTTATTACTTTTACTTACGAGAA
TATAAATACTCTTTTCGAAGATATAAGATATCTAAATGAACCTCTAGCTGATACAAATATGCAGTTTGGA
CTAACTGGTAAAAATATGTGGCTTAGATTTGTTGAAAAATTTAAACAAAATTTACAGCTAGAAATAGAAG
CTCTGTATGGTTATGCTGTACGTAAGGCACAAGATAACACTTTAAAATCTCGGGCAAACCCAAATAGAAT
AACTTTAGAAGAACTAAAAAAACAAATAGCTGATTTTAAGAAAAACTCACAAAATGATTAG

>lcl|NC_006570.2_cds_YP_170258.1_1136 [gene=murB] [protein=UDP-N-acetylenolpyruvoylglucosamine reductase] [protein_id=YP_170258.1] [location=complement(1326861..1327709)]
ATGTCAGAGTATATATCTTTAGAGCAATATAATACCTATCGTATTAAGTCTTTTGCTAAATATGTTTATT
TTCCTACTAATAACCAAGAGCTATTGGATATTGTTAATAATCATAATAAATTATTTTTCCTTGGAAACGG
TAGCAATGTTATTTTTTCTAAAGAGTATTATGATGATGTAGCATTTGTGATTTTTACTAAAAAATTCAAC
TCTTTTAACATTATTGATAATTATGCTAGTGTCCAAGCTGGAGTATTATTACAGGATCTTGCATTTGCTA
CCTATAATGCCAGCTTAAGTGGTATTGAGACTTTTTATGATGTGCCAGCTAGCGTTGGTGGGGCATTGAT
TATGAATGCTGGTGCATATGGTGATGAGATATATACTTGTGTCAAAAGTGTCACAATACTTGACCTTAAT
ACTAAACAGATAAAAAAATATCTAAAAAAAGATATAGAGTATGGTTATAGATACTCTATATTTAAATATA
TGAAAGATATTTGTATTTTATCAGCGGAATTTGAGTTTGAGTACAAATCAAAACAAGAAATCAAAGCAAA
ACTAGATGATATTTATTCACGCAGATTGTCTAATTTACCACAAAAGCCAACAGCTGGGAGTGTTTTCAAG
CGACCACAAGCAAATATGCCTGTGGGAATTATGGTAGAGCAGCTAGGATTAAAAGGTAAACAAATTGGTG
ATGCGCAAATTTCTCCAAAACATGGAGGTATAATAGTGAATAATGGTAATGCAACTGGTCAAAATATTTT
AGATCTTATCGAGTTTATCAAACAACAAATTTTAGAGCATTATAATATCGAGCTGCATGAAGAGCAAATT
GTTATTTAA

>lcl|NC_006570.2_cds_YP_170259.1_1137 [gene=murA] [protein=UDP-N-acetylglucosamine 1-carboxyvinyltransferase] [protein_id=YP_170259.1] [location=complement(1327717..1329051)]
TTGCAGATAGAAAAAATAAGAGATCAAAAGATGAAGGCAGTAAGAGTAAAAAAGTTAAGTAAGCTTGCTG
ATGAGATAACTATTAACATTAGTGGTGCAAAAAATGCTTTGTTACATCTAATTTTTGCAAGCTTAATTCC
GGATACTAAAACAAAATTTACTAATGTTCCAATAACTCTTTTAGATTATAAAGGGGCTAAGGAGATCTTA
GAAAATGTTGGTGCTAAAGTCATTGAAAAGGATGAAGAAGTAACTATTGATACTGCAGCTATTTCAAATG
AAACTTTAGAACTATGTGGGGAAATGACATCTAAAACACGTTGCTCTTTGATGCTTTTAGGTTCATTGCT
TAAGAAAAAAGGTCGTGTTAAGATTGGTTTTCCTGGTGGTTGTAGTTTTAGTGAAAAAAGACCTTTTGAC
ATACACTTAAATGGATTAGAAGCATTAGGTGCTGAGGTTAAGTTGGCTGATGATCATATAGAGGTAATTT
ATAAAGAAGAAAAAAACGCAGAGTTTAAAATGCCTTTTCCATCGGTTGGCGCAACTATGAATTTACTAAT
GTACGCCGTGACAGGTAATTCTGAGATTGTACTTGAAAATGTTGCTTTAGAGCCTGAGGTTGTTACATTA
ATAGACTATCTTAATCAATGTGGTGCAAATATTGATTTTGATGCTGATAGCAGAAAAATAAAAATCTTAG
GTATTATGAGATTAAATGGCTGTGAGTTTGAAATTATTTTTGACCGTATTCAAGCTATGACCTATGCCGC
TATGGCGTATCTGTACAAAACAAACGTTACAATTACAAATATTAATACTCAAGATACATACAGTATCAAA
AAGCCTTTGGAAAAAATTGACTAATGCAGGAGCTAAATGGGAGTATAACCAAGCTAATCGTAGTATTAAGT
TTTTTGGTAAGGATAGTTGTATTAAAGGAGTTGATATAATTGCTGCACCTTTCCCACATTTTCCTACCGA
TTTACAGCCAATATATGCAGTAATGTTATTTATGGCAAATAGCTCTAGTACTATTCAAGATACAGTTTAC
CCTGAGCGCATAAATTATGTGTATCAAATTCGTAAAATGGGTTTTAATATATCTATAGATAATACTCTTA
TAAAAATAAATCCACTTAAAAACCTAAATGATATACGTCCAGCAGTGATGAGTGTCAAAGATTTACGTGC
TGGTATGGCATGCTTAATGGCTGGATCATTATTAGATGAATTTTCGACTATTAATAATGCTCATCAGATA
TTTAGAGGTTATAATAACCTTATAGAAAATATGTCTCATTTTATGCGAATAGAGATTCTAAATGATAATG
TTTAG

>lcl|NC_006570.2_cds_YP_170260.1_1138 [gene=engA] [protein=GTP-binding protein EngA] [protein_id=YP_170260.1] [location=complement(1328990..1330387)]
ATGTCTTTTTTAGTTGCAATTGTAGGTAGAGCCAATGTTGGTAAATCTACTCTTTTTAATGTTTTGACAA
ATTCGTACGATGCCTTAGTATTTGATTTTGAGGGTGTAACTCGTGATCGTCAATACGGACAAGCAAAGTA
TGATGATTTAGATTATCTAGTAGTTGATACTGGAGGTATTTCAGATAAAGATGTGGGTTTTGATGAGTTT
ATGGCTAAACAATCACAGATTGCTATAGATGAGGCGAATTTAGTTTTCTTTGTGGTTGATGGCAGATCTG
GATTGACAACCGGAGATGAGTACGTTGCTAGCCTTTTACGCCAGAAAGATAAGAAAGTAGTAGTTGTGGT
AAATAAGGTTGATGGTACCGATGAAGAAGCTGCAATGGCTGAATTTTATAGTTTTGGTTTTGATAAGGTA
TTTGCAATATCTGCAGCACATCGTAGAAACACGCAAAAGCTAGTTGATAAGTTTCTAAAAAAAACATTAA
ATGAATACTATCAAGATTATACTCAAACACAAGAGCATAAAGAACAACAGCGCCATGGTATCCATTTTTC
ATTAATTGGTAGACCTAACGTTGGTAAATCGACACTTACAAATAGAATGCTTGGCGAAGATAGGGTTGTG
GTATTTGACATGCCAGGCACTACAATTGATAGTGTCAGTATCCCATTTGAACGCCATGGTCAGAAGTATA
CTATAGTTGATACAGCTGGTGTGCGCAAAAGAGGTAAGGTAAAACAAACTTTGGAAAAGTTTTCGGTAAT
AAAAACATTACAAGCAATACAAGATTCAAATGTAGTAGTTGCAGTAGTTGATGCAAGACAAGGTATCTCA
GATCAAGACCTGAGTCTGATACATTTTGCTATTAAAAATGGTAGAGCATTGGTCTTAGCTGTAAATAAAT

GGGATGGTATGACAGAAGAAGATCGTATTCAGGTTAAGCAAGATCTTAAAAGAAAGCTTTTTTTCCTGCA
AGATTATGTCGATATACATTTTATCTCAGCATTACATGGTACTAATGTTGGGCATGTTTTTGAGTCAATT
GATACTGCATATGCATGTGCAAGTAAAAAAATAACTACAGCTGATGCTACACGTCTAATGCAGCTTGCGG
TAGAGGCACACTCACCGCCAATGGTAGGTAAATTTAGAATTAAGCTTAAGTACGCTCATGTTGGAGGACA
TAATCCTCCTGTTATTGTAATACATGGTAATCAAGTAAGTAGACTGCCAAACTCATATAAAAGATATTTA
GAAAATTTCTTTAGAGAAGCCTTAGATTTTCGTGGTACGCCAATAGTATTTGAGTTTAAGCAGTCAGAAA
ACCCTTTTGCAGATAGAAAAAATAAGAGATCAAAAGATGAAGGCAGTAAGAGTAAAAAAGTTAAGTAA

>lcl|NC_006570.2_cds_YP_170263.1_1141 [gene=hflB] [protein=ATP-dependent metalloprotease] [protein_id=YP_170263.1] [location=complement(1332931..1334847)]
ATGATTAAAAATATTATTTTTTGGATTTTAATCATTGGTGGGATGCTACTGCTTTTCAATGGTATTAATG
ATACAAATGGGTCATCTAAAAATATAAATTATTCGACATTTATTTCTAAGTTAAAAGATAATCAAATTAG
CGTTATAGATGTCGATGGTAGAACTATCACGGGTAAAACCAATGAAGGTGAGAGTTTTGTAACATACGCA
CCATTACTAGATGGTAGTTTAGTTAATAAATTAGAAGATAGTAATGCTATAGTCAAAGCAAAAGCTCCAG
AAAAGCCTAATATATTCTTGGCATTTTTGCTTAACTGGTTACCAATGCTACTAATTTTTGGCTTTTTCAT
TTATATGATGGTAAAAGCTGGTGGCGGTAGCAAAGGCGGACCTTTCTCTGTTGGTAAGAGTAAAGCAAAA
TTACTAGGTGAAGATGAGATTAAAGTAACTTTAGATGATGTTGCTGGTGTTGATGAAGCCAAAGAAGAAG
TTGCTGAAATAGTTGATTTCTTACGTGAGCCAAAAAAATATGAGAAGATTGGCGGTAAGATTCCTAAAGG
CGTATTGATGGTAGGACCTCCTGGTACCGGTAAGACATTATTAGCTAGAGCTATTGCTGGTGAAGCAAAG
GTTCCATTTTTCTCAATTTCAGGCTCTGATTTTGTTGAGATGTTTGTTGGGGTTGGTGCATCGCGTGTTC
GTGATATGTTCGAACAAGCTAAAAAGAAAGCTCCTTGCTTAGTTTTTATCGATGAAATAGATGCAGTAGG
CCGTCATCGTGGCTCAGGTATGGGTGGTGGTAATGATGAGAGAGAACAAACTCTTAACCAAATGCTTGTT
GAGATGGATGGTTTTGCCGATAATGAGGGTGTAATAGTAATAGCAGCTACTAATAGACCAGATGTTTTAG
ACAGAGCATTACTAAGACCTGGTAGATTTGATAGACAAGTTACAGTTGGTTTACCAACAGTAAAAGGACG
TGAAGCGATACTAAAAGTGCATATGAAAAAAGTTGCTCTAGGTGAAGATGTCCGTGCTGATTGGATAGCA
AGAGGCACACCAGGATTTTCTGGTGCAGAACTTGCAAACCTTGTTAATGAAGCGGCTTTATTTGCTGCAA
GAGAATCTAAAGATAAAGTTTCGATGGCTGATTTTGAAAAAGCCAAAGATAAAATCTTAATGGGTTCTGA
AAGAAGAAGTATGGCAATGACTGAAAAAGAGAAAAGGCTTACGGCTTATCATGAAGCGGGTCATGCTATT
ATTGGTCGTTTGATGCCAGAGCATGATCCAGTTTATAAGGTAAGTATCATACCTAGAGGTAGAGCACTTG
GTGTAACAATGTATATGCCAGAAGGTGATACTGTTAGCCAAAGCAGACTAGTGTTGCGTGGGCGCTTATG
TAGTATTTTGGGTGGTAGAATAGCTGAGGAGCTTATTTTTGGTTATGATCATGTAACTACTGGAGCTTCT
AATGATATTCAAGTGGCTACTGATATAGCGCGCAATTATGTTGCTCGTTGGGGCTTATCTGATACTATGG
GAACTATCCTGTATGACGTTGAAGATGAAGGACCTTTTGGCGGTAGTGGTGGCAAGTCAGTAAAACTTTC
AGACTCTACTATCAGAGAAGTAGATACTGAAGTGCGTAAACTAATAGCTACAAGTTATGCTAAGGCTAAG
CAATTGTTGGAAAAGAATATCGATATCCTTCATGCTATGGCTGATGCACTAATGAAATATGAGACTATAG
ATGCACTACAAGTTGATGATCTAATGGCTAGACGTCCAATGCGTGAGCCAGGTGAATATGGTGATAGCTA
TAAACCAAAGGATGCGGGTAAGGTTATAGCACCAGTAGCGCCTAATGAAGATGAGTCAGAAATAGCTGAT
GATTCTAGCCTTAAAGAAGATCTTTAA

>lcl|NC_006570.2_cds_YP_170268.1_1146 [gene=pgi] [protein=glucose-6-phosphate isomerase] [protein_id=YP_170268.1] [location=complement(1340444..1342066)]
ATGCTATTTTGTGATGATTCTAAAAAATATTTGAAAGAGCAAAATATAAACTTAAAAAATGAATTTGACA
AAGATGATAAAAGAGTTGAGAAGTTCTCTCTAAAACATCAAAACATATATTTTGATTATTCAAAAAATCT
TATTAATGATTATATTTTAAAGTCTCTTTTAGAGTCTGCAGAAAAATCTAGTCTTAAAGATAAAATTAAA
CAGATGTTTAATGGTGCTAAGATTAATTCTACTGAACACAGAGCTGTTTTACATACGGCGTTGCGCGATT
TGTCTAGTACGCCATTAATTGTTGATGGGCAAGATATTCGTCAAGAAGTAACAAAAGAAAAGCAGCGTGT
AAAAGAGCTTGTTGAAAAAGTGGTATCTGGTCGATGGCGTGGATTCTCTGGTAAAAAGATTACTGATATT
GTAAATATTGGTATTGGTGGCTCAGATCTTGGACCTAAAATGGTGGTTAGAGCATTACAACCTTATCATT
GTACAGATTTAAAAGTTCACTTTGTTTCAAATGTTGATGCTGACTCGCTATTACAAGCACTACATGTTGT
TGATCCTGAAACTACTTTGTTTATCATAGCTTCAAAATCATTCTCAACTGAAGAGACTTTGTTAAATTCT
ATTTCAGCTAGAGAGTGGTTGTTAGATCATTATGAGGATGAAAAAGCTGTAGCTAACCATTTTGTTGCAA
TATCGAGTAAGTTAGATAAAGTTAAAGAATTTGGTATAGATTTAGAGCATTGTTATAAAATGTGGGATTG
GGTTGGCGGTCGCTATTCATTATGGTCATCAATTGGGATGTCGATAGCTTTTGCTATAGGTTATGATAAT
TTTGAGAAATTACTAGCTGGAGCGTACTCAGTTGATAAACATTTTAAAGAAACTGAATTTAGCAAAAATA
TTCCTGTGATTATGGCATTGCTGGCAAGTTATTATAGCTGTACGTATAATAGTCAGTCACAAGCGTTACT
TCCTTATGACGAGAGACTTTGTTATTTTGTTGATTATCTTCAGCAAGCAGATATGGAAAGTAATGGTAAA
TCAGTAAATATTGCTGGTGAAACTGTCAATTATCAAACTGGAGTTGTACTTTGGGGTGGTGTTGGTACTA
ATGGTCAGCATGCTTTTCATCAACTTTTACATCAAGGTAATATTTTTATACCCGTTGATTTTATTGCTAT
TGCAACTAGTCATCATAATTATGATAACCATCAGCAGGCTTTACTTGCTAATTGTTTTGCACAGTCACAA
GCACTAATGTTTGGACAATCATATGATATGGTTTATAATGAGCTGTTAAAATCTGGTTTGAATGAAACTC
AAGCAAAAGAGCTTGCGGCTCATAAGGTTATACCAGGTAATAGACCAAGTACAACAATTTTACTTGATGA
GCTAAGTCCATATAGTCTTGGTGCCTTGATTGCTTTGTATGAGCATAAAATATTTGTTCAGGGTGTGTTA
TGGGACATCAATAGCTATGATCAGTGGGGTGTTGAGCTTGGCAAAAAACTTGGTAAAAATATATTAAAAG
CTATGAATGATGATTCATCTGATGAGTATCAAAATTTAGATGATTCAACTAGACAGTTAATAGCGAAGGT

AAAAAAATAAATAA

>lcl|NC_006570.2_cds_YP_170269.1_1147 [gene=FTT_1316c] [protein=thiamine pyrophospokinase] [protein_id=YP_170269.1] [location=complement(1342053..1342706)]
ATGTCTGAAGCGATACTTTTTCTAAATGGTAAAGTAGATTTGTGTTTTTGTGAGAAATATATCGAAGATA
ATTTTGCAAAACTTGATATATTTTGTGCTGATGGGGCTTATCAAAAAGTTAAAGAATCATTACATTTAAA
TTCAAAAATAAAAAAAGTAATTGGAGATTTTGATTCATTTACTTTATCAGAAGATGATTTGTTTTTAATT
GATACTGATCAATATTCGACTGATTTTGAGAAATCTTTGAATTATTTAATCTCTCTTGGGGCTTTAAAGG
TTTTTGTATTTGGCGCTTCTGAGGGTGAGATGGATCATTTTTTATGCAATATTAGTATTGCTAAAAACTA
TAAGCAAAAGATAGAGATTGAGTTTATAGATATTTATTCAAGATACTTCTTTATACCTAAAAAGTTTGCT
GTAAGCGGTGTTGTTGGTAAGATGTTTTCTGTTATGCCTTTTGGATATGCAGAAAATATATATTATAATG
GTTTAAGGTATCCTTTAAGCGGCGAGAGCTTAAGTATAGATACTAATACAGGGGCAAGAAATTATGCTAC
AGAGGATAAGGTTGAGATATCTTATTCAAGTGGAGATATTTTGTTGTTTATATCACATAGAAAATATAAG
GATAGATTAGATGCTATTTTGTGA

>lcl|NC_006570.2_cds_YP_170270.1_1148 [gene=guaB] [protein=inosine-5'-monophosphate dehydrogenase] [protein_id=YP_170270.1] [location=complement(1342772..1344232)]
ATGTTAAGAATTACTCAGCAGGCAATCACTTTTGATGATGTATTGCTATCACCTAGATATTCAAATGTTC
TTCCTCATCAAGTTGATTTGAAGACAAATATAACTAGAGATATTCAGTTAAATATTCCTTTAGTATCTGC
AGCTATGGATACGGTTACTGAGTCACGCCTAGCTATTGCGATAGCCCAGGAAGGTGGTATTGGTATTATT
CATAAGAATATGTCTATTCAAGCCCAAGCCCAAGAAGTTAAAAAGGTAAAAAGATTTGAAAATGGAATGG
TGATTGATCCCATTACCATTAAACAAGAAAGCTCAATTAAAGAAATCATGCAACTAGCTAAAGAACATAA
TTTCTCTGGTTTTCCCGTTGTTGATGATAATAATAAAATCATAGGTATTGTTACAAGGCGTGATTTTAGA
TTTGCAAAAGATTTAGATGAACCTGTTAGCTCTATTATGACACCTAGAGAAAAACTTGTAACAGTGCCTG
AAGATGCTTCTCAAGGAGCGATTAAAAAGAAACTTCATGAGCATAAAATTGAGAAGTTGCTTGTAGTTAA
TGAACAAGGCGAGTTGGTCGGTCTTATTACAACAAAAGATATCGAAAGATCACAAAATAAACCAAATGCT
TGTAAAGATTCTCTTGGGCGTTTGAGAGTTGGCGCTGCTGTTGGAACCGCTGCTAATACTAAAGAGCGTG
TTGCAGCATTGGCAGCTGAAGGAGTCGATATTATTGTTGTAGATACTGCTCATGGACATTCACAAGGTGT
GTTAGATACAGTTAAGTGGGTTAAAGAGAACTATCCACATATTCAGGTTATCGGTGGTAATATCGCTACA
GCAGAAGCTGCTAAAGATCTAGTCAAAGCTGGTGCTGATGCTGTCAAGGTTGGTATTGGACCTGGCTCAA
TCTGTACTACCAGAATTGTTGCTGGTGTTGGTGTACCACAAATTACAGCGATAGCTAATGTTGCAGAGGC
ATTAAAAGGAACTGGAGTGCCTGTGATTGCAGATGGCGGTATTAGATATTCCGGTGATATTGCAAAAGCT
ATAGTTGCTGGAGCATCTGTAGTTATGATTGGAGGGCTTTTTGCAGGTACTGAAGAATCACCAGGGGAGG
TTGAGCTTTTCCAAGGTCGCTCTTATAAATCATATCGTGGTATGGGTTCTCTTGGTGCTATGGAGAAAGG
CTCATCTGATAGATATTTCCAAAGTGAAACAGAGGCGAAAAAGTTTGTTCCAGAAGGCGTTGAAGGCAGA
GTACCATACAAAGGTTTATTATCTGCAGTGATTCATCAGCTAATAGGTGGTCTGAAATCTAGTATGGGCT
ATACTGGCTCAAAAGATATCCAAACAATGCGAACAGAACCGACATTTGTACAAATTACAGGTGCTGGATT
TAATGAATCGCATGTACATAACGTCACGATTACTAAAGAACCACCTAATTACCAGTCTTAA

>lcl|NC_006570.2_cds_YP_170272.1_1150 [gene=FTT_1319] [protein=YjgP/YjgQ family permease] [protein_id=YP_170272.1] [location=1345938..1347020]
TTGATTCTTGAAAAAATATTACAATAAAGATATAGTTAATACCTTTACATCTATCACATTATTTATAATAT
CTATAGTATCAGCTAATCTACTAATTAGACTCTTCCAAGAAGCCTATTCACAGGGTTTAGGAATCGATTC
AATTGTTAAATTTGTTATATTAACAATACCAGAAAATGTAAGCTTAGTTGCTCCTATTGCAATATTTCTG
GCAATAGTCATTTGTTTTGGTAAATACTTCGCTAATAATGAAATGTTTGTAACTATAGCTGGTGGTATCA
CTTGGATGCAAATAGTCAAAAATACTTTAAAGCCTGTAGTTGCATTGACTATAATAACGCTTATAACAGT
GATGTATTTAAACCCTCTTTCAAAACAAACTTTAGATATTTACAGAGCTTCTTTATCTGCCAAAGCTCTT
CTATCATCAATAACTGATCAAAAAATAATCAAAGCCCCTGATGGTAAAATTATTTATATTGGTAATAAGT
CAGGAAATACTTTGTCAGACGTTTTTTTATATCAAAATACTCCACAAGATGGCGAGTATAAAGTTATGAC
TGCACCAACTGCAAAAAATTGTCTCTGATAAAACCGCTGCTTATATAGATTTTATGAATGTGAATATTTAT
ACAAAAAAATTCCCAAAACTCTGAGTATAGCTATGATAATGCTAAAAAAGCTATATATACGATATTTGATA
ATTCTGACCGTGATTATAATCATGATAGAGTAGATAGACTATATATGCATACTCTAATAGAAAACTTTAA
TGATAAAAATAATGGTACTGCATATAAAGCAGAATTTTTGGGACGCATTAATAACGCAATATCAGTGATA
GTATCTTCACTTTTAGCTCTTGCTCTTTGTCGCCTACGACCAAGGCAAAATAAATATGCTAAGCTTTTAC
CATCAGTAGTTGTATTAGCTATATACTTATGTACAAATATGTTTATTAATACTCTTATGGCAAATAGTAG
TGTACCTGTATGGATAGGTTTTTGGTTGCCTCATATCTTTTTTACAATTTTTGCTGTTAGAACAATTAGA
AAAGATAATGGATCTTCTAAGAGGGAAAAAATAA

>lcl|NC_006570.2_cds_YP_170273.1_1151 [gene=FTT_1320] [protein=YjgP/YjgQ family permease] [protein_id=YP_170273.1] [location=1347020..1348081]
ATGCTATTGAATCGTATCGATCGTTATATTTTTAGGACCGTATTTAGCAGCTTTCTAATAGTTAGTATAA
TTTTCTGTATTTTATTTTTTATCTTTACATACTTAGCTCAAGTAAGTAACAATACTGCGAATGTAAGTAA
TTTTGAACTTATTATCAATACATTAATTCAACTTCCAGGTATCTTATATACACTACTACCGGCATGTGCG
ATGGTTGGTGCTCTTATGGGTTTAAGTTTACTTGCAAATAATTCAGAGATAATTGTACTTAGATCTTTTG

GTCGTTCTACAGCTCAAATCGCTAAAGGTGTAATTTTAGTTGGTCTAATAGGCTCTGTAACAACTATGGT
TTTTGGTGGCTACATAGCCCCAATTTTACAACGCCAAGTAGATACTAATATGGTCACATACAATACTCAT
GATCTATGGTTTAAAACTCCTGATGGCTTTATGAATATTGCTAATATAGAACCTACTAATGGCAAAGCTT
ATGGGATCAGAAAGTTTATGGTGCAAGATAATAAGGTTAGAGAAATCCGTTACGCAGAAAGTGCAACTTA
TATTAACGATGCTTCAGCTAATGTTTTTAATATAAGAAAAATTATTTTTCCTGCTAGTGATGGTCAAAAA
CATATTGATATAGTCACAAATATGACTAACGCTGTATGGTCAAATCCATTACCTATTTCAGTAGCCAAAG
TCATTACCATAAATGATAATAATTATTTAAATTTCTCTCAACTTACCAGCTATATGCTTTCAAATAGTCA
AGCTAAAGATTCGATATCTTTAAAATTTTGGCAAGAAGTTTTCCAGCCATTATCTTTGATGATACTAATT
CTACTTGCCGTGCCTCTTAGTATTGGTTCAACGAGATCATCAACACTTATACTTAAGCTTTTACTTGGAG
CTTTTTTTGGTTTTGCATTTTTTATAATAAACCAAATATTTGGTCCTATTGCACTAATTTTACACTTACC
ACCAATACTTGGTGCTGCAGGGCCAACTGTTATAGCGCTAATATTGTTAATATATTTATTTATAAAATCA
AAAGAAACATAA

>lcl|NC_006570.2_cds_YP_170281.1_1160 [gene=gpmI] [protein=2,3-bisphosphoglycerate-
independent phosphoglycerate mutase] [protein_id=YP_170281.1]
[location=1357287..1358825]
ATGAAAAAAACTACTCTTTTAGTAATCCTAGATGGCTGGGGTTATAGTGATAGTGACTATTTTAACGCTA
TCAAAAACGCAAACACTCCAACTTGGGATAGTATTTGGCAAGAATTTCCTAAAACACTTATCAATGCCTC
TAGTTTGGAAGTAGGCTTACCTAGAAGCCAAATGGGCAACTCTGAGGTTGGTCATGTAAATATAGGCTGT
GGTCGAGTGGTCTATCAAGAATTAACAAAAATTGATAAAGCTATCGAAGAAAAAACCTTTGGTGATAATA
AAGCAATCTGTGCAGCTATTGACAATGCAATCAAAAATGACTCTAATCTACACCTAATCGGGTTACTCTC
ACCAGGTGGTGTTCACTCTCATGAAGAGCATATTTTTGAGATGATAAAAATCGCCAAACAAAAAGGTATC
AAAAGATTATATTTACATGCATTTTTAGATGGTAGAGATACTCCTCCACGCTCCGCAGAAAAATCAATTA
AAAAAGCAGATAAACTACTACAAGATCTAAACTTAGGCTATATCGCTAGTGTTTGTGGTAGGTATTATGC
AATGGACAGAGATAATCGCTGGGATAGAGTAGAGAAAGCTTATAATGCTATTGTTAACGCTAATGCCGAT
TTTATCTATGACTCAGCACTAGAAGCACTAGAGCAATCGTATGCGCGTGATCAGTCTGATGAGTTTGTTA
TACCTACTTGCATCAAAAAAGATGGACATCTTGTAAAAGTCCAAGATAATGATAGTGTTATCTTTATGAA
CTTTAGAGCAGATAGAGCTAGAGAAATTTCACATGCTTTTACTGATGAAAGCTTTGATCATTTCCCAAGA
AAAAAACATTTGAATATAAACTTCACTACGCTTACAGAATACGATTCAAAACTTAAATGTGCAGTAGCAT
TTCCACCTGAGCAGCCTATAAATACTCTTGGCGAAGTATTAATGAAAAATCATAAAACTCAACTAAGAAT
CGCTGAAACTGAAAAATATCCACATGTAACATTCTTTTTTAATGGTGGTAGAGAAGAACAATTCGAAGGC
GAAGATAGAATACTTATTCCTTCACCAAAGGTAGCTACTTATGATTTACAACCAGAAATGTCAGCTCCAG
AGGTTACAGATAAGTTAGTCGCAGCTATTAATAGTGGTAAATATGATTGTATAGTATGTAACTATGCTAA
TTCTGATATGGTTGGTCATACAGGTAATTACGAAGCAGCTATGCAAGCAATTGAGTATCTTGATAAGTGT
ATTGCCAGATTAAAAGATGCCATACTTGAGCATGATGGTAACATGTTTATCACGGCAGATCATGGTAATG
CTGATATGATGGTTAATCCAGAAACACAAAAACCTCATACAGCTCATACTACAAATCTTGTGCCATTTAT
ATATGTAGGACATAAAAAAGCTCAAGTTGCTTTAGAGCATGGTAAACTCTCAGATATTGCACCAACTCTA
CTTAATGTCATGGGTATAGCTCAACCTAAAGAAATGACTGGTAAAACTATTTTTAACTTTGAAAAATAA

>lcl|NC_006570.2_cds_YP_170282.1_1161 [gene=serS] [protein=seryl-tRNA synthetase]
[protein_id=YP_170282.1] [location=1358840..1360120]
ATGCTTGATGCTAAATATATTAAGGATAATTTACAACAAGTTGCTGAAAAGCTTGCAACTAGAGGTTATC
AATTTGATATAGCCGAATTTGAAGCCCAAGAGCAAAAAAGAAAACATCTTCAAGAAAGAACTCAGGATCT
ACAATCACAACGTAATACTATTTCAAAAGAAATAGGCCAAAAAAAAAGCTAAAGGTGAAGATACTAGTGAT
ATCTTTGCTAAAGTTAATCAAATTAATGAAGAACTAAAAATTATTGAAAAAGAGCTCAAAGACCTACAAG
ATACTATAAATCAAACACTATTATCAATGCCAAACCTTCCTGCTGATGACGTCCCTGTTGGTAAAGATGA
GAATGATAATGTAGAAATAAGAAGATGGGGAACTCCACGTGAGTTTCATCCTGAAGCACCTGCAAAAGAT
CATTCTGATATTGGTGAAATCCTCAAAATGATTGATTTTAAAGCCGCTGCTAAAGTTACTGGCAGCCGTT
TTATGGTCCTGAAAAATAAGATTGCTAAACTACACCGCGCTCTATCACAGTTTATGCTTGATCTGCATAC
AGAAAAACATGGTTATGAAGAATTATATGTGCCATATCTAGTCAATAATGATAGTTTATATGGTACTGGA
CAATTACCAAAATTTGCTGCTGATCTTTTTAAGCTTGAGGGAGATTTTGAATATAGTCTAATACCTACAG
CAGAGGTGCCAATCACTAATCTAGTAAGGGATGAAATTTTAGATACAGAAACTTTACCTAGATACTATAC
AGCTCACACTCCTTGCTTTAGAAGTGAAGCTGGTTCATATGGCCGTGATACTAAAGGTATGATTCGTCAA
CACCAGTTTGAGAAAGTCGAGTTAGTACATATCACTACTGCAGATAAAGGTGAAGAATCTCTAGAATTAC
TCACATCACATGCTGAAAAAGTCTTACAAAAACTTAACTTACCATATAGAGTGATGAAACTATGTACTGG
TGATATGGGCTTTAGCGCTAAAAAAACTTATGATTTAGAAGTATGGCTCCCATCACAAAATACTTATAGA
GAAATATCTTCATGTAGCTGGTGTGGAGATTTCCAAGCTCGTCGTATGAAAGCTCGACACAAAAATCCAA
GTATGAAGAAACCAGAATTAGTCCATACTCTAAATGGTTCTGGTTTGGCTGTAGGTAGAACATTATTAGC
TATTATTGAAAATTATCAACAAGAAGATGGTTCTATTATGGTTCCCGATGCTCTTATCAAGTATATGGGC
GGAATTTCAGTTATTAAGTAA

>lcl|NC_006570.2_cds_YP_170284.1_1163 [gene=FTT_1332] [protein=polysaccharide
biosynthesis protein] [protein_id=YP_170284.1] [location=1361207..1362466]
TTGATTAAAAAAATACTTTCTAATCATATTCTATCTAAGTCTTTACAAACTATGACAGTACAGGCACTAG

GTCAAGGATGTGCCTTTATTACTGCTATTCTTTTAGCCAGACATCTAACAATTAGCGATTATGGTTTTTA
TATTTTTGGCGTAACAGTTGCAACTATTTTAGCTGTAATCGCAACAATGGGTGGCGGTGGTATCTTAGCA
CGCACTTGGGGTAAATCAGAATTAGTCGATAAATTTCAAAGAAACAAAGAAACTTTTTTAGTTCATAACT
GGTATTTCAAGCGCGGCTTCACTATAGTAATTATAATAATTGCTATAATATTTTTTTATAACCATTCTAA
ACACCATGATAACTCAATAGAAAATTTTACATTATTATTTGCTATACCATTTTTTATGGCAAATATATTT
CAATCTTTTTTTGTTGCCAGAAGAGTCGTTATCCTTGCTAACTTCCTTCAACTTAGTCTGCGAATTATAA
TGCTCTTGTTGACTATAGTTTTTATGTCTTTGTACATAAAAGATACTGCTTTGCTTATTGGTACAATGAT
GATTATAATGACAATATATACAACATCTATTTGGCTTAGCCAAACATCAAAATATACTTTCACCAGCTCA
AAGCCAATCGGTAGTAATATCTCATTTGCTCTAATGCAATGGGGATTATTATTACTATCACAAATCGATA
TCATAATCTTAAAAGTACTAGCAACTCCCGCAGATATCGCTCTCTACGGTGTAGCACTACAACTTGGCGC
ATTAGTTATTTTTGTATTAAATGCGGTTAATTCAAATGTCCTATCACAAATTGCTGATGATTATAAAAAC
TGCTCTAGAAAAGAATTTCAAAATAAAATAACCTCTTATACTAGAATTATCTTTATACTCTCAATATTTG
CTATTTTAGGAATTATTATTTGTGGATATCCAATTACTTTAATGTATGGTAAACAATATACCGCATCATA
TTTTATCTTTTGTATATTAATGGTTGGGCAAATAGTAAATGTTCTATCTGGTTGCGTTGCTACGATTCTA
AATATGGCAGGATATGAGAAAAGTACATGTTTTGCTTTCTATATTGCACTTGCTATTAATATATTGCTAG
GAGTAGTTTTTACGCTTTATTGGGGTGTATATGGTCTTGCAATTGCCTCAAGCTTATCAATGATATATTG
GAATATTCATCTACTTTACAAAGTAATTACACAGATAAAAATTAACCCAACAATATTTATAATTAGATAA

>lcl|NC_006570.2_cds_YP_170287.1_1166 [gene=cydD] [protein=cysteine/glutathione ABC transporter permease/ATP-binding protein] [protein_id=YP_170287.1] [location=1363584..1365365]
ATGCTTTCAGATGCTTCACAAGAAGATAAAAAAGTAGCTAGGAAATGGCTCAAACAAATATCATATCCCG
CTAAGAGATGGATTAAATTAACTATATTGATAGCTTTTTTAAGCGGCTTACTACTAGTTGGACAATTATA
TTTATTAGCTCATATTTCTTATGCTGCATACATCGATAAATCTAACTTTGGTCAACTAAGTAACTACTTT
ATAGTTATTGTTTTAATAGTCATAATTAGAGCAGCTCTTGCTTGGTTACGAGAAATTGTCAGTTATAAAG
CCGCGACAATTGTCAAAAAGCAAATTAGAGAAGATATCTTGGCTCATGTAAACCAACTCGGTCCAATTCA
GTTAAACAAAACTTCTAATGCAAATATAATTACAAGTGCGATGGAACAAGTCGAAGGACTAACTGGGTTC
CTAACAAAATTTCTACCACAAATAACTTTATCAGGACTATTACCTTTAGCTGTTTTAGTATTTATATTCC
CTCAAAGTATTGTCTGTGGAATCCTTCTACTAATAAGCGCACCACTTATTCCACTTTTTATGGTATTAGT
TGGTTTAGGCGCTGAATCTGAAAGCCAAAAGCATTTCAAAGCACTTGCTAGAATGAGTCTAACTTTTCTA
GATACGCTCAAAGGTCTAACTACACTTAAGCTTTTTAATAGAAGTAAAAACCAAAGCCAATCTATTTTTC
TAGCTTCTGATAACTATAGAATCCGTACGATGAAAGTTCTTAAAATTGCCTTTTTATTATCGGCTGTATT
AGAGCTTTTTGCCGCAGCTTCTATTGCATTAGTAGCAATATATCTAGGTATGGGTTTCATAAATACAGGC
TCAGGGAATAATATTTGGTGGTCAATCGATAATATTACTTTACAAGGTGGCTTATTTATACTACTACTTG
CTCCAGAATTTTTTATGCCATTACGTGAGCTTAGTACACATTATCATGCCAAAGCAGAAGCAGTTGGAGC
TGCTCTAGAAATCGCTAAAATATTTGAGATGCAACCATCTCATCAAGAAAGAAACGAAATATTTAGCGAT
AAAGTAAACTCTATAAACATCAAGGATCTGACTGTTAAATATGATGATAAAATAGCTCTTGATAATATTT
CTCTAGAAATTAAAGATAAGGATAAAATAGCTATAGTGGGTGCCAGTGGAGCTGGTAAAACCACACTAAT
AAACACTCTACTTGGTTTTATAAAATATCAAGGTAATATCTTAGTTAATAACAATATTGAGCTAAGAAAT
CTTGAAGAAAAAACTTGGCTTAGAAATATCTCGTGGCTAGGACAAAACTCATCATTATTCAAAGGTTCTA
TCAAGGATAATTTGCTATTGGCTAATAGCAACGCTACAGATGAACAAATAAATCAAGCTTTACAAAACAC
TGATTTAAATGAATTTATAAGCTCTCTTGCAAATGGTTTAGATACGGAAGTTGGCGAACAAAATATAGGA
GTATCTGGAGGTCAAGCGCAGAGACTAGCTCTTGCTCGTGCATATCTAAAACCTCATGATATCCTAATTC
TCGATGAGCCTACAGCAAGTCTAGATAAAGATAGTGAAGAAAAAATTATCAACTCACTTAAATCAAACTG
GAATGATAAAACTGTGATTATGCTAACTCACAAACTAAGCTTCCTAGAGTGTGTTGACAAAATTGTAGTC
TTAGCAGATGGAAAAATAGTTGAAACAGGAGCATTTGAAGAGCTAGTTAGCGATCAAAATAGTGAATTCT
ATAACTTCTATAGAAATGAGGTAACAGCATGA

>lcl|NC_006570.2_cds_YP_170288.1_1167 [gene=cydC] [protein=cysteine/glutathione ABC transporter permease/ATP-binding protein] [protein_id=YP_170288.1] [location=1365362..1367011]
ATGAGAAATTTAATACCATTTATAAAGCTGTTTAAAAATCAAACTCAATGGATGCTCTTGGGTACCCTAC
TTGCATGGTCAGCAATACTTATGGGCATAGGATTAATGTCACTATCTGGTTGGTTTATCTCATATACTGG
TTATTTAGCTACTACTACCTATGCTATTGCAACTTCATTTAACTATTTTTACCCTTCTGCTGGTGTACGC
TCTTTTTCACTTGGTAGAATACTAAGTAGATATGGTGAAAGAATACTAACACATGAAGCAACTTTTAGAA
TAATCACAGATATTCGTGTCTGGTTCTATCAAAAGCTTGAACCACTTGCACCTTCTCATTTATACAAATA
TAAAAGTGGCGACTTACTTACACGTCTAGTCAATGATATTGGTGCATTAGATAATCTATATATTCGTATA
ATCTCTCCAACTGTTGTATTTGTCTTAGCAAGTTTAACTATAGCTATATTATTTAGTTTCTTTAGCTTAA
GCCTTGCGCTACTGACATTTATAGCATTGCTGTTAATAGGCTTTGTAATACCGTTGGTTAGTAGTCTATT
AGCCATGAAAAAAACTCAAGATCTTAATCACACTAGCGCTGAATTAAAAACAAATATCACAGAACATGTT
AACTCTCTAGCAGAGCTTAAAATCTTTGACTTAGAAGGTAAGCATCTTGAAAACATTAAAAAGCAAAATT
CTGAACTACTAAACCAAGAAGAGAAAATCAGCTTTATAAGTGGTTTTGGTAGTGCTTTAATGACTTTGGC
ATTAGGCTTAACTGTAGTTATGGTAACAGTCTTTGCAGTTAAACTAACTCAAAATGGCTATATAAGCGGT
GCATTTATTGCTTTGATATTTTTGGCAATAATGGCGATGTTTGAGTCAATTATGCCATTACCATTAGCAT

ATCAGTATTTAGGTAAGACAATTTCTGCTTCAAAAAGGATATTAAATATTACAGATGCTAAAGCAGATGT
TGAATATCCAACAAACTCTAATATTAGCTTAGATAACTATAGCATCCGCTTCAATAATGTTGATTTTGGC
TATAATGCAGAGCAATCTGTACTTAAAGATTTTTGTTTAGAAATCAAACAAAATGAAAAAATAGCGCTAT
TCGTACCAACAGGAAAAGGCAAATCTACTATTATCAACCTACTTGCTAGGTTTTGGGATGTTAATAGTGG
CGAAATCACTATCAGCGATAGAAATATCAAAGAATTTAACGAAGATCAACTTAGAAACCTAATGACTGTG
ATTAATCAGTCTCCGCATATTTTTAATACAACTATTAGAGAGAATCTAAAGATTGCTAAAGATAATGCTA
CTGAAGAAGAGCTTCTAGAAGCTCTAGAAAAAGTTGAATTAAGAAAACATGTTGAATCACTACCAAAACG
TCTTGATACTTGGACTGGTGAGCTTGGTAGACAATTATCAGGCGGTCAACAAAAACGCCTAGCTTTAGCG
CGTGCATTCTTACAAGATAAGCCTATACTTATCCTAGATGAGCCTACTGAAGGACTTGATAAAGAAACTG
AAAGATTAGTTTTTGAAAATTTAGTTAAGCTTATGCAAAATAAGACTGTAATATTTATTACTCATAATGC
TAAGCTTTTGGAAAGTTTTGATAAAGTTGTTAGGCTATAA

>lcl|NC_006570.2_cds_YP_170315.1_1194 [gene=pyk] [protein=pyruvate kinase] [protein_id=YP_170315.1] [location=complement(1411283..1412719)]
ATGAGAAGAACAAAAATTTTAGCTACTCTTGGTCCTGCTAGTGAATCAAGAGAAGCTCTAACAGAGATGA
TTAAAGCAGGGGTTAATGCAGTTAGATGTAATTTTTCGCATGGCTCTGCTGAAGATCATCGCAAAAGAGT
TGATTTAATTCGTCAAATCGCAAAAGAGCAAGATACACATGTTGGTATTTTAGCTGATTTACAGGGACCA
AAAATCAGATTATCAAAATTTAAAAATGGTTCCGTAGAAATTAAGAAAGGTCAAAAATTTACACTGGATG
CTGATCTTGGTATTAATGATGGTGATGAGAATACTGTAGGGATTGACTATAAAGAACTCATTCAAGATGT
AAAAAAAGGCGATATACTACTTGTAGATGATGGCAAAATTGTCTTAGAGGTTGATTCTGTAAAAGGAAAT
AAGGCAGTCACTAAAGTTGTCGTTGGCGGTAAAGTTTCTAATAATAAAGGCATCAATAAGAAAGGTGGCG
GACTTACAGCACCTGCACTTACTGAGAAAGACAAAGAAGATATAAAAATAGCTGCGATGCTACAAGTGGA
TTTCTTAGCGGTATCATTTGTCAGAGATGGTAAGGATATGGAGTATGCACGTCAACTTGTCCACGAAGCC
GGTTGGAGACCTGCTATGGTGGCTAAAATTGAGCGTGCAGAGGCTGTCTTGGAAGATAACCTTAAGAGTA
TTGTTGATGCTTCTGACCTTGTAATGGTAGCGCGTGGTGATTTGGCTGTTGAAATTGGTGATGAGAATGT
GCCAACTGTACAGAAGCTTATTATCAGTACAGCTAGACGAAATGAAAAAGGTTCTATTACTGCTACACAA
ATGATGGAGTCGATGATAGAGAACTCTTCACCAACTCGTGCTGAAGCATCTGATGTTGCTAATGCTGTTT
TTGATGGCACAGATGCTGTAATGTTATCAGCTGAAACAGCGGTAGGTAAATATCCTGTTGAAACAGTTTC
TGCAATGTCAAGGATATGTAAATCTGCTGAAAAAAGTAAGTATACACATATTTCTAAAAAGCAAAAAATA
GCTGATTGTGATAGGATTGATCATGCTGTATCTGTTGCAGCAGTCAAAATAGCTAACGATATTGGAGCTA
AAGGATTAATTGTATTGACAGAGGGTGGTAATACTTCGCGTTGGATGTCACGAATAAATACTGATTTACC
TATTTACGCATTATCAAGAAACGCAACAACTTTAGGTGCGATGACATTATTTAGAGGTGTGGTACCAATA
TACTTTGATTCAACTAGAATGTCTAAGTTGTATGTAAATAGATCAGCTTGCATGGAGCTAGAGAGCAGAA
ATTTAGCCAAAGATGGTGATATTTTTGTGCTTACTAGTGGCGATAGTATGGGTGTACATGGAAGCACTAA
TAAGATTAAAGTGATAATTGCAGGTCAGGTAAGATAA

>lcl|NC_006570.2_cds_YP_170316.1_1195 [gene=pgk] [protein=phosphoglycerate kinase] [protein_id=YP_170316.1] [location=complement(1412735..1413913)]
ATGAGTTTTCTAACATTGAAAGATGTTGATTTAAAAGATAAAAAAGTCTTGGTTCGTGTTGACTTCAACG
TACCTGTTAAAGATGGTAAGGTAACAAGTAAAGTTAGAATTGAAGCGGCTATTCCGACTATTCAATATAT
TCTAGATCAAGGTGGAGCTGTGATTCTGATGTCACACCTTGGTCGTCCTACAGAGGGTGAGTATGATTCT
CAGTTCTCTCTAGAGCCTGTTGCTAAAGCTTTATCTGAAATTATCAATAAGCCGGTTAAGTTTGCTAAAG
ACTGGTTAGATGGTGTTGATGTTAAAGCTGGTGAAATTGTCATGTGTGAAAATGTCCGCTTTAATAGTGG
TGAGAAAAAAATCAACAGATGATTTATCTAAAAAGATTGCAAGCTTAGGTGATGTGTTTGTTATGGACGCT
TTTGCTACAGCGCATAGAGCTCAAGCTTCGACCTACGGTGTGGCTAAATATATTCCTGTAGCATGTGCAG
GGATATTGTTAACTAATGAAATTCAAGCATTAGAAAAAGCTTTAAAATCGCCAAAAAAACCAATGGCAGC
AATAGTTGGAGGGTCTAAGGTCTCTACTAAACTATCGGTGCTAAATAACCTTCTTGATAAGGTTGAAATT
CTTATTGTTGGTGGTGGTATTGCTAATACTTTCATAAAAGCAGAAGGTTTTGATGTTGGTAATTCATTGT
ATGAACAAGATCTTGTCGCTGAAGCAACAGAGATTTTAGCTAAAGCTAAGGCTTTAGGGGTTAATATTCC
TGTGCCAGTTGATGTAAGAGTTGCTAAAGAGTTTAGCGAAAATGCCCAAGCAATCATTAAAAAGGTTTCC
GATGTTGTTGCGGATGAGATGATTCTAGATATAGGTCCAGAATCTCAAAAGATAATTGCTGAGCTGTTAA
AATCAGCAAATACTATCTTATGGAATGGACCTGTTGGAGTATTTGAGTTTGATAACTTTGCAGAGGGTAC
AAAAGCTTTATCTTTAGCTATAGCACAATCGCACGCTTTTTCAGTAGCTGGTGGTGGTGATACTATTGCT
GCGATAGAAAAATTTGGCATTAAAGACCAAGTTTCTTATATATCAACAGCTGGTGGAGCATTCTTAGAGT
TTCTTGAAGGCAAGAAATTACCAGCTATAGAGATACTTAAAGAAAAAGCAATCAGGTAG

>lcl|NC_006570.2_cds_YP_170317.1_1196 [gene=gapA] [protein=glyceraldehyde-3-phosphate dehydrogenase] [protein_id=YP_170317.1] [location=complement(1413932..1414978)]
ATGTTTAATAAACTTTCGCAAGATAATATCTTAAGGAGATTAAAAATGAGAGTTGTAATTAATGGCTTCG
GTAGAATTGGTCGTTTAGCATTTCGTCAAATGTTTGGTAAAGATAACATTGAGATTGTCGCGATTAATGA
TTTAACAAATCCTGAGATGTTAGCACACTTATTGAAATATGATTCTGCTCAAGGTAGATTCTCAAAAGCA
GCTGTAACAGTAGTAAAAGAAAGCTCTATAGTTGTCGATGGTAAAGAGATTAAAATATATGCTGAAAAAG
ATGCAGCTAATCTACCTTGGGGCGAGTTAAATGTCGATGTTGTACTAGAGTGCACTGGTTTTTATGTATC

AAAAGCAAAGTCACAAGCTCATATTGATGCAGGTGCTAAAAAAGTAGTTATTTCAGCGCCAGCTGGTAAT
GACCTACCAACAGTTGTCTTTGGTGTTAATCATGACATCCTCAGCGCAGATGATAAAATCATATCAGCGG
CTTCTTGTACAACTAATTGTCTAGCTCCTATGGCTAAAGCTCTTCATGATCTAGCAACCATTGAAAGTGG
TTTTATGACCACTATTCATGCATATACAGGTGACCAAAATACTTTAGATGCTCCTCATGCGAAAAATGAC
TTCCGTCGTGCAAGAGCTGCTGCTGTAAATATTGTACCTAACTCAACTGGTGCTGCTAAAGCTATTGGTT
TAGTGATTCCTGAACTTGCTGGTAAGTTAGATGGGGCTGCTCAGCGTGTCCCTGTTGCTACTGGTTCTTT
AACTGAGCTTGTTGCTGTCGTATCTAAGAAGGTAACAGCTGAAGATGTTAATGCTGCGATGAAAGCTGCT
GCAAATAAATCTTTTGGTTACACAGAAGAAGAGCTAGTGTCTAGTGATATCATCGGGATTTCTGAAGGAT
CGTTATTCGATGCAACTCAAACTAAAGTTACTTCACTAGGTGATAAATCTCTAGTTAAAGTAGTATCTTG
GTATGACAATGAAATGTCTTATACAAATCAAATGGTTAGAGTAGTAGAGTACTTCGGAGCTCTATAA

>lcl|NC_006570.2_cds_YP_170318.1_1197 [gene=tktA] [protein=transketolase] [protein_id=YP_170318.1] [location=complement(1414971..1416962)]
ATGTCTTTATCTATTCCACGCGAGTTTTCAAACGCTATAAGATTTTTATCTATTGATGCAACACTTAAAG
CTAAATCAGGACATCCTGGAATGCCTATGGGGATGGCTGATATTGCCACAGTGTTATGGACAAAGTTTCT
TAAGCACAATCCAAATAATCCACATTGGATAAATAGAGATAGATTTGTTCTTTCAAATGGGCATGGCTCA
ATGCTTTTATATTCTCTGTTACATCTGACAGGTTATGATCTATCTATTGAGGATATTAAAAATTTTAGGC
AGTTACACTCAAAAACTCCAGGGCACCCTGAGTATGGCTATACGCCAGGAGTTGAGACAACAACTGGTCC
ATTAGGGCAAGGAGTAGCAAATGCTGTGGGTATGGCTTTAGGTGAAAAGCTATTATCAGATAGGTATAAT
ACACCTGATTTAAAAGTTATAGATCATCACACTTACGTGTTTTTAGGTGATGGATGTTTGATGGAGGGTG
TTTCTCATGAAGCATGCTCATTAGCGGGGACTCTAGGTTTAAACAAATTAGTAGCATTTTGGGATGATAA
TAATATTTCGATAGATGGCGATACTAAGGGTTGGTTTAGTGATAATACACCTGAGAGATTTAGGGCGTAT
GGTTGGCATGTTATAGAAAATGTTGATGGTCATGATTTTGTTGCGATTGAAAAAGCTATAAATGAAGCCC
ATTCTCAGCAGCAAAAACCAACTTTAATCTGTTGTAAAACTGTAATTGGTTTTGGCTCACCTGAGAAAGC
TGGAACTGCCTCTGTGCATGGTTCGCCTTTAAGTGATCAAGAAAGAGCCTCAGCTGCTAAAGAGCTTAAC
TGGGATTATCAAGCTTTTGAAATACCTCAAGATGTTTATAAATACTGGGATGCTAGGGAAAAAGGACAAG
CTTTAGAAGCAAATTGGCAAGGGCAATGGAATTTATTTAAAGACAGTCCTAAGTTTGACGAATTTGAACG
AGTTTTAAGTAAAGAGTTGCCAGTTGGATTAGAAAGCGCTATTAATGACTATATAGCTTCGCAATTGAGT
AATCCTGTCAAAGTGGCAACACGTAAAGCTTCACAAATGGTGCTTGAAGTCTTGTGTAAGAATATGCCTG
AGATGTTTGGTGGATCAGCAGATTTGACAGGATCAAATAATACTAATTGGAGTGGTTCAGTTTGGCTAAA
TAATACTCAAGAAGGCGCAAATTATCTTTCTTATGGTGTTAGAGAGTTTGGTATGGCTGCAATAATGAAT
GGTCTAAGCCTTTATGGTGGTATCAAACCTTATGGTGGTACATTCTTGGTATTTAGCGATTATTCAAGAA
ACGCGATAAGAATGTCAGCGTTGATGAAACAGCCTGTAGTTCATGTTATGTCGCATGACTCAATTGGTTT
GGGAGAAGATGGACCAACTCATCAACCTATAGAACATGTTCCGAGTTTAAGACTTATACCTAACTTAAGT
GTTTGGCGACCTGCTGATACAATTGAAACAATGATAGCATGGAAAGAGGCTGTAAAATCTAAAGATACGC
CAAGTGTAATGGTACTTACACGTCAAAACTTAATGCCAGTAGTGCAAACACAGCATCAGGTAGCAAATAT
TGCTAGAGGTGGTTATCTAGTTAAAGATAATCCTGATGCTAAATTAACTATTGTTGCTACTGGCTCTGAA
GTTGAGCTTGCTGTCAAAGTTGCTAATGAATTTGAAAAGAAAGGTATAAAATTAAATGTTGCTTCTATAC
CGTGTGTAGAAGTTTTTGCTACTCAAGCTCACGAGTATAAAAAAACAGTAATAAAAGATGATATCCCGGC
AGTTTTTGTTGAGATGGCGCAACCTGATATGTGGTATAAATATATGCCAAAAGCAGGAGGGGAAGTAAAA
GGAATCTATAGCTTTGGTGAGTCAGCACCGGCTGAAGATTTATTTAAACGTTTTGGATTTACTGTAGAAA
ATATTAGCAATATTGTTGCTAAATATGTTTAA

>lcl|NC_006570.2_cds_YP_170320.1_1199 [gene=rpmF] [protein=50S ribosomal protein L32] [protein_id=YP_170320.1] [location=1417649..1417831]
ATGGCTGTACAACAAGTTAAAAAAAGCAGATCAAAAAGAGATATAAGAAGATCTCACGACTCTTTAACAA
ATCCTACTCTATCTACTGATAAGTCAACAGGTGAATTACACTTGAGACATCATGTATCACCTAATGGTTT
CTACAAAGGTAGAAAAGTAGTAGATACTAAATCTGAAGACTAA

>lcl|NC_006570.2_cds_YP_170321.1_1200 [gene=plsX] [protein=glycerol-3-phosphate acyltransferase PlsX] [protein_id=YP_170321.1] [location=1417930..1418976]
ATGGGTTACAAAATATCTATAGATGCAATGGGTGGAGATCATGGTTTAAACACTACTATCCCAGCAGCTC
TTGAAGCAGTAAAAAAAGACTCTAATTTGCAAATAGTATTAGTTGGAGATCATCATAAAATTAAAAGAGC
TTTAGACAGATATTCAAAAGTCAAGAAAATAAAACTACCAGTTTTACAAAGAATAGCTATTCATCACGCT
AGTGAGACAGTTGGTATGGATGAATCACCTTCTATTGCTGTTAGAAAGAAAAAAGACTCTTCTATGCGCG
TAGCTATCAATCTTGTCAAAGATCGTACTGTTGATGCCTGCGTCAGTGCTGGTAATACTGGTGCGCTAAT
GGCTACATCAAAATTTGTATTAAAAACTATCAATGGTGTTGATCGCCCTGCTATTGTTTACGCATTACCT
GCATTCAATAGAGAAACTAAGCAACTTAGTAAAACTTATATGCTTGACCTTGGTGCAAATGTCGTTTGTA
CGTCTGAACAACTTTTCCAATTTGCAATCATGGGATCAATATTAGCAGCAAGTTCAAAAGGTATTGCTGA
ACCTAGAGTTTCATTACTAAATATTGGTGAAGAAGAAATGAAAGGGTTAGATAATATTAAAAATGCTGCA
AAACTATTACAAGGCTGTGATTTCATTAATTACAACGGATACATTGAGGGTAAATACATTTTTGATGATA
CTACCGATGTAATAGTCTGTGATGGCTTTGTTGGTAATGTCTCTCTAAAAACAATGGAAGGAAGCTTAAG
ACTTATAGAGTCTCTTATCAAAAAAACAATACAAGAAAGCTCCTTATTAATGAAAATCCCTATAGTAATG
GCATTACCAATATTCAAAAAGATGAAAAAAGGTATGAATCTTGATAGCTTTAATGGTGCATCACTATTAG

GTCTAACTGGAATTGTTGTTAAAAGCCATGGTGGTGCAAGTGCCAATGCTTTTGAGACTGCAATTTATGA
AGCTATCAAAGAAATTAAATACAATATCCCTAAAACAATTCAAGAATCTTTAGAAAAAGTTCTTTAA

>lcl|NC_006570.2_cds_YP_170322.1_1201 [gene=fabH] [protein=3-oxoacyl-ACP synthase] [protein_id=YP_170322.1] [location=1419073..1420044]
ATGTTTGCACAAATATTAGGTACAGGAAGTTACTTGCCTGAAAAAATCTTAACTAATGAAGATATAAGTA
AATTTGTTGATACTTCAGATGAGTGGATCAAACAAAGAGTCGGCATAGAAAGACGCCATTGCGCCAGTGA
AGCAGAAACGACAAGTTACATGGCTACACATGCAGCTAAAAAGGCTCTTGAAGCGGCACAATTAACTGCT
AACGATATTGATATGATAATTGTTGCAACAAGTACCCCTGATTTCATAATGCCTTCAACAGCGTCAATGG
TTCATCAGAATCTACAAATAGATAATTTCAAGGTACGTTGTTTTGATATATCAGCTGCTTGTAGTGGGTT
TGTTTATGCTTTGGATATCGCTAAACAATATATAGAGACAGGTGTTTCAAAAAACATTCTCGTAATTGGT
GCTGAAAAAATGACGCGGGTCCTAGATTGGAATGACCGTTCAACATGTGTACTTTTTGGTGATGGTGCTG
GTGCTGTTGTAATTTCAACAAGCCAAGAAAAAAAGATATTATCATCTTTACTTTTTACAGATGGCTCATG
CTTAGATATGCTTAATGTCCCAAATAATTTACCAACTTCAAGAGGACAAGCTATAAATATTGATCCTTAC
TTAATAATGGAAGGTAATAAGGTTTTTAAATTTGCTGTTTCACGCTTATCTTCATTAGCTGATGAGTTAA
TACAAGAAGCTGGAATAAAAGCTAGTGATATTGATTGGCTTGTACCTCATCAAGCAAACTATAGAATACT
AAACTCAACTGCCAAAAAAATTGACATGCCAATGCCTAAAGTTGTTACAACACTACAAGATCATGGTAAC
ACTTCTGCAGCATCTATCCCATTAGCCTTAGATCATGCTGTAAGAACAAATCAAATTAAACCTGGCGATA
CAATTATCTCTGAAGCATTCGGAGCTGGATTTGTCTGGGGTGGTTTTATCGCGAAGATATAA

>lcl|NC_006570.2_cds_YP_170323.1_1202 [gene=FTT_1374] [protein=malonyl CoA-ACP transacylase] [protein_id=YP_170323.1] [location=1420092..1421012]
ATGTCAAAAACAGCTGTAGTTTTTCCTGGTCAAGGTTCACAAAAACTAGGGATGCTCCAAGATTATTATG
AAAATTTTGAAACGTTTAGAAATATAGTCGATGAAGCTAAAGAACACCTTGGCTACGACTTATGGAATAT
TATTCAAAATGATGAAGAAACTCTAAATAAAACAGAGTTTACCCAGCCAGCATTACTTGCAACTAGTTAT
GCAATATATGAAGTCTTAAAAGAGCAAAAGCCAGACTTAAAAATAGCATACTTTGCAGGACATAGTTTAG
GTGAATACACTGCCCTACTTGCTGCTGGATGTATTTCATACAAAGATGCTTTACAACTTGTATCTACACG
TGGCAAATTAATGCAAAATGCTGTTACTGACAAAGAATGTGCTATGAGCGCAATTCTAGGTTTATCAAAT
GAGGATGTAATCAAATCTTGTCAAGAAGCTAGTGATGCTGGAATTGTTGAAGCTGCAAACTTTAACTCAA
CAGGACAAGTTGTCATCTCTGGGGAAAAAGCCGCTGTTGAGAAAGCTAATACAATAGCTAAAGAAAAAGG
TGCAAAACGCGCGCAGATACTTGCTGTTAGCGTACCTTCACATTGTTCTTTAATGAAGGATGCTGCAGAT
AAATTTGAAGCAGAGTTAAACAAAGTAGAATTTAAAGAGCCTACTACCGCTGTTGTACAAAACTTTGACG
CCAAATCACACGCAAATCCAGCTGAAATAAAAACTGCTGTTATTAAACAACTATACAAGCCAGTACTTTG
GACACAATCTATCGAAGAGCTAGTCAAACTTGGAGTCACAGAAGTTATCGAATGTGGTCCTAACAAGGTC
TTATCTGGACTAATCAAAAGAATAGATAAATCAATAGATATAAAAGATACAAACAGTATTGATAGTTTAG
AAAATATTTAA

>lcl|NC_006570.2_cds_YP_170324.1_1203 [gene=fabG] [protein=3-oxoacyl-ACP reductase] [protein_id=YP_170324.1] [location=1421026..1421769]
ATGTCTTTGAATGAAAAAGTTGCTCTTGTGACAGGTGCAAGTAGAGGTATTGGTTTTGAAGTTGCTCATG
CATTAGCAAGCAAAGGTGCTACTGTAGTAGGTACTGCAACTAGTCAAGCTTCTGCAGAAAAAATTTGAAAA
CTCAATGAAAGAAAAAGGATTTAAGGCAAGAGGATTAGTCTTAAATATTTCAGATATTGAAAGTATTCAA
AACTTCTTTGCTGAAATAAAAGCTGAAAATCTAGCAATAGACATTCTAGTCAACAATGCTGGTATTACTC
GTGATAATTTAATGATGAGAATGTCAGAAGATGAATGGCAGTCAGTTATAAATACTAATCTAAGCTCAAT
CTTTCGTATGTCAAAAGAGTGTGTACGTGGGATGATGAAAAAAAGATGGGGCAGAATTATCTCTATAGGT
TCTGTAGTTGGCTCAGCTGGTAACCCAGGACAAACCAACTATTGCGCAGCTAAAGCTGGTGTAATAGGTT
TCTCAAAATCTCTTGCTTATGAAGTTGCTAGCCGTAATATCACAGTAAATGTTGTAGCTCCAGGATTCAT
AGCTACAGATATGACTGATAAACTAACTGATGAGCAGAAATCTTTTATCGCTACTAAGATACCTTCAGGA
CAGATAGGAGAACCAAAAGATATCGCTGCAGCAGTAGCTTTCTTAGCTTCTGAAGAGGCGAAATATATAA
CAGGACAAACTCTTCATGTTAATGGTGGTATGTATATGGCTTAA

>lcl|NC_006570.2_cds_YP_170325.1_1204 [gene=acpP] [protein=acyl carrier protein] [protein_id=YP_170325.1] [location=1421865..1422149]
ATGAGTACACATAACGAAGATTCTAAAAAAAATAATGCCGATGAAAAAGCAAAAATATTTTCTAGAGTTA
ACCATATAATAGTTGAGCAATTAGGCGTTAAAGAAGAAGATCTTAAGCCAGAAGCTTCTTTTATCGATGA
TTTAGGTGCGGACTCATTAGACACAGTAGAGCTTGTAATGGCTCTAGAAGAAGAATTTGATACTGAGATT
CCAGATGAAGATGCTGAGAAAATCAGAACTGTTAAAGACGTTTACGACTATATCGAATCTAAAGATGTAG
GTTAA

>lcl|NC_006570.2_cds_YP_170326.1_1205 [gene=fabF] [protein=3-oxoacyl-ACP synthase] [protein_id=YP_170326.1] [location=1422229..1423488]
ATGAAATCTAATCGTAGAGTAGTTGTTACTGGCTTAGGCATGGTAACACCTTTAGGAAATGATGTCCCTA
CGACTTGGGCTAATATCTTAGCTGGCAAAAGTGGAGTAGAAACTATAACTGGTTTTGACACACCTGCTCC
TGATATTAGCGAGTTTAAAGTTAGATTTGCAGCAAGAATTAAAAATTTTGATGTAGATGCACTTGTAGGT

AAAAAAGACGCAAAGAGAGTAGATCCTTTCTGCTACTATGGTATTGCCGCTGCTAATGAAGCTCTTAAAG
ATGCTGGAATTGATAAAGTATCTGAAGAAGATTCATATAAGTTTGGCGTATGTGTTAGTTCTGGTATCGG
TGGTATCGAGACTTTAGAAACTACTAAAGCTGTAATAGATACAAAAGGACCTTCTAAAATTTCACCTTTT
TGTATACCATCCTCAATCGTGAATATGCTCTCTGGAATCATTTCTATAAATCATGGCTTACGTGGGCCTA
ATATTCCAATCGTTACTGCTTGTACTACAGGTACTCATAATATTGGTATGGCTGCTAGATTAATAGCTAG
TGGTGATGCTAATACTATGCTTGCTGGTGGCTCTGAAAAAGCAAGTAACGCTATTGGTATGGGTGGATTT
GCTGCTGCTAGAGCATTATCAACGCGCAATGATGATCCACAAGGTGCATCACGCCCTTGGGACAAAGACA
GAGATGGCTTTGTACTTGGCGATGGTGCTGGTGTTGTTGTCTTAGAAGAATATGAAAGAGCTAAAGCTCG
TGGCGCTAAGATTTATGCTGAGGTTGTGGGTTTTGGTATGTCTGCTGATGGTTATCATATGACTATGCCT
TATGCTCCAGGTCAAGAAAGATGTATTCAGAATGCTTTAGCTGATGCTGGACTTGAAAATAATCCTGATG
CTATAGACTACGTTAATGCTCACAGTACTTCAACTCCACTAGGAGATGTTCAGGAGTCACAAGTTGTTGA
AAAAGTAATTGGACAATATAGAAAAGATCTAGTTATGAGCTCAACAAAATCAATGACAGGACATTTACTT
GGTGCAGCTGGTGCTATTGAATCAATATTTAGTGTATTAGCAATAAGAGACCAAGTAGCTCCACCAACAA
TAAACCTACATAATTTAGATGATGGCTGTAACTTAGATTATGCTGCAAATGCTGCTAAGAAAATGAAAAT
TGATTATGTGCTAAATAACTCTTTTGGTTTTGGTGGCACTAATGGTTCTGTAATTTTCAAAAAAATCTAA

>lcl|NC_006570.2_cds_YP_170332.1_1212 [gene=ligN] [protein=DNA ligase] [protein_id=YP_170332.1] [location=complement(1431189..1433225)]
ATGACTCCAAACGAATTTTTTTCTATAAAATATCATATTTTAGCTAAAGCAGAATTAAAAGCTTACATAG
ACAAATTAGCTGATTATCTTAGTCAACAGAGCTATTTGTATCATACCTTAGATAAGCCAATTATTTCTGA
CTCTGATTATGATAAGCTGTTTAGATTACTTCAAGATTTAGTTAATGACAATCCCCAATTTAAGCCGATA
AATTCAGTATTAGATCGTGTTGGAGGTGAGGTTTTAGCAGGGTTTGAAACTATCAAGCATAAAAAGAAAA
TGACATCTTTGGCAAATGTTTTTAGTCTAGAGGAGTTGCGTGATTTTTATGACAAGATAGAGTATGACAT
TGAGCTTGAATGTGAACCAAAAATGGACGGTCTAGCAATCAGCATTTTTTATAAAAATGGTAAGTTTGAT
TATGCTGTTACACGTGGTGATGGTATTCAGGGTGAAAAAGTTTCAGAAAATGTTAAAACTATCCGCAATG
TCCCACTTAAACTAAATACTTCAAACCCTCCAGAAGAACTAGAAGTTCGTGGTGAGATTATCTTAGATAA
GCAAAGTTTTCTATCTCTTAATGAGTATATGCAAACTCATGAGAATAAAACTTTTGCAAATCCGCGAAAT
GCAGCAGCTGGAAGTATTCGTATGCTTGACTCAAAAGTTGTAGCAAAGCGACCACTTAAGCTTTATAGCT
ATGGTATTGGCTATTTTTCTAAAGACTTTGTATATCCTGAAACCCAGTTTGAACTAATGCAGCTACTCCA
AAGTTTTGGCTTTACAATTAGTGATAATATGTTTTTGGCAAAAAATTTCTCAGAGGTTGAGGAGTATCAC
CATAAGATGAGTCATCAGCGTGCTGATTTAGCTTATGATATTGATGGTTTAGTTTTTAAGGTTAATAATA
TTAAACTACAAGATACAATTGGCTACACTGCTAGAGGACCTAAGTGGGCTATAGCATATAAGTTCCCAGC
TGAAGAGGTGGAGTCAGAAGTGCTAAATGTAGAATTCCAAGTTGGCAGAACAGGAGCAATTACGCCAGTA
GCAAGACTTAAGCCAGTCGCAGTTGGCGGCGTAATAGTTTCAAATGCAACCTTGCACAATATCAATGAAA
TTAAACGCAAAGATATCCGAGTTGGTGATAGAGTAATCGTACGTAGAGCAGGAGATGTAATTCCAGAAGT
GGTCAAAAGCTTACCACAATATCGTAAGTCTGATGCACAGATGGTTGAAATGCCAACAAATTGTCCAGTT
TGTGATTCAAAGATTGAGAATGTTAATGATCAGGCTATATATCGTTGTACAGGAGGATGGCATTGTCAGG
CACAAACTACTGAGCGCTTGAAGCACTTCGTCTCACGTAAAGCAATGGATATTGATAAGCTTGGAGCTAA
ACTAATTGAACAACTTGTTGCTGCAAATTTAATTAAGTATCCTGCTGATATTTACAAACTCAATTTTGAG
CAATTGACAGGCTTAGAAAGAATGGCTGCTAAATCATCACAAAATGTCTTAGACTCTATCACAAAAAGTA
AAGAGCCAAGTTTAGCAAGGTTTATTTTTGCAATAGGGATTAAAGATATCGGTGAAGTATCATCAGACGC
TTTAGCGAATCATTTTGGTAGTCTAGAGAGCTTCCGTGATGCTAAGTTTGAGGAGCTAATAGAGATTAAT
GATATTGGTGAGATTATGGCTAATAATATTGTTTCATTTTGGCATGATTCTCTAAATATTAAGATTGTAG
AAGAGTTTTTAGCTATTGGGATTAAGATACAAAATCCAGTAAAAGTTGAACACGCCTATAACGAGAGCTT
CACAGGTAAAACTGTTGTTATTACTGGGTCTTTTGAGAACTATGGTCGTACAGAACTTACACAGCTTCTT
AAGTCAATCGGGGCAAAAGTTACCTCAAGTGTTTCTAAAAAAACTGACATGGTTATTTGTGGCGATAATG
CTGGTAGCAAACTTACAAAAGCCCAAGAATTAGGTGTAGAAGTTATTCTTGAAGATAATCTGAAAGATTT
ATTATGA

>lcl|NC_006570.2_cds_YP_170353.1_1233 [gene=FTT_1409c] [protein=hypothetical protein] [protein_id=YP_170353.1] [location=complement(1458812..1459228)]
ATGGAAAATCAAGTTGTACAAAAACAGCAAGAATTAGTCGAAGAGCTATCATTTTTTGAGGATTGGGAAG
ATAAGTATGATTATGTTATTTCATTAGCTAAGCAGCTACCAGAATTCCCTGAGGAGAAAAAGACCGAAGA
AAATTTAGTCAAAGGTTGCCAATCACAGGTTTGGTTTGATAGTAATATAGATCAAGGTAAGCTAAATTTT
ATAGCTACAAGTGATGCTTTGATAGTCTCTGGTTTAATAGGAATGCTTTTAAGAGTTTATAATAATGCTA
CTCCTGCAGAAATACTAGCTTCAAATACTGATTTTATTAAGCAGATAGGTTTTGGTAATAATTTAAGTAC
AACACGTGCCAATGGCTTAAAATCAATGCTTGATTATATATATGCTACAGCTAAACAAAATCAGTAA

>lcl|NC_006570.2_cds_YP_170355.1_1235 [gene=proS] [protein=prolyl-tRNA synthetase] [protein_id=YP_170355.1] [location=1463060..1464757]
ATGAAAGCAACACAAACTCTAATTGCTACGACAAAGGAACTACCAAAAGAAGCAGTTCTAATTAGTCATC
AATATATGCTCAAAGCTGGTCTTATCAAGAAGCTAGCTTCTGGTATCTATACATGGATGCCTCTAGGTCT
AAAGGTACTCCAAAAAATCCAAAATATAGTACGTGATGAGATGAATAAAGCTGGAGCAAGCGAACTACTT
TTACCAAGTATACTACCTTCAGAGCTTTTACAAGAAACTCATCGCTGGGATAAATTTGGTCCAGAACTTT

TAAAATTACATGATCGACATAATAGAGATTTCTGTTATGGTCCAACACATGAAGAACCAATTGTTGATAT
GGCTAGAGATACTATCAAAAGCTACAAACAATTGCCTTTAAATCTTTATCAAATCCAAACTAAATTCAGA
GATGAGATTCGCCCTCGTTTTGGTGTAATGCGTGCACGTGAATTTATAATGAAAGATGCTTATTCATTTC
ATGAAAATAGTCAATGTCTACGCAATACTTATAATACAATGTACGCAACCTACTGCAATATCCTTGACAA
AATAGGCTTAGCTTATCGCCCTGTTAAAGCTGATACCGGAGCTATAGGTGGTGATAATAGTCATGAGTTT
CAGGTATTAGCAAATGCTGGTGAAGATATTATTTGCTATAGTAATGGTTCTGACTATGCTGCAAATATTG
AGTTAGCAACTTATGCAAAATCTGATCTAAGCAAAAGAGTAAACTCTCAAAATACCATTGAGAAAATACA
CACTCCAAACATCAAAACTATCGAAAAACTCTGTAAAGAGATGAGCTTTGATATTAAAAAGACTATCAAA
ACTATGGTTATCAAAGATGCCGGAGGAAATTTCTTTGCTTTAGTTATCCGAGGTGATCATGAATTAAACG
AAACTAAGATTAATAAGCTTGATCAAATTATTGCACCATATACATTAGCTACTAAAGAAGAGATTTTTTC
AATATTTAATGCTAATCCAGGCTCGCTTGGTATATATAACTGCCCTATCAGTATAATCGCTGACTACAGT
GCAATTGCTATTACAGATCTAGTTTGTGGCGCTAATGAAGATGATTATCACTTTACAAATGTAAACTGGG
ATAGAGATGTAACTAACTATCAAATCGCCGACATTAGAAATGTAGTAACTGGCGATATTTCCCCTGATGG
TAAAGGCACTCTAGAACTTACAAATGGTATCGAGGTTGGTCATATTTTTGAACTTGAAGATGTCTACTCA
AAACCAATGAATGCAAATATAATCGGTCAAGATGGTAAATCTAAACCTATGCTTATGGGTTGTTATGGTT
TTGGAGTGTCGCGTGTGATGGCAGCAGCAATTGAGCAATCTCATGATGAAAATGGGATTATATGGCCTGA
GTCTATTGCTCCATACCAAGTTGCTATACTACCTATCAACTACAATAAATCTGACAAGATTAAAGAAGTA
GCAGATAAACTTTATCAAGACTTGCTTGGAGATGGTATTGATGTCCTATTAGATGATCGAGGTGCTAGAC
CTGGCGTGATGTTCGCTGATGCTGATCTAATTGGTTACTCTCATCATGTAGTAATTGGCGATAGACTACT
TGAGCAAGGGCTGATTGAATATAAGAATCGCAAAACTCAAGAAAAACAAGAGATAACTATTGCAGAGCTG
ATCAAGATTTTAAAATAA

>lcl|NC_006570.2_cds_YP_170361.1_1241 [gene=nusB] [protein=N utilization substance protein B] [protein_id=YP_170361.1] [location=complement(1468913..1469338)]
ATGAAAACTACCGCTAGAGCTCGAAATAATGCTCGTTTATATGCCGTACAAGCTTTGTACCAAAAGAAAA
TCGCTGATAATACATTCTCTGAGCTTAAGATTCAGTACTATGCTGATAATGCTGATAGGCATTATACTGA
TTGGGATCTTTTCTATAGACTTATAGATGCAGTTAAGACAAATCAAGACACTATAGACAAATATATCAAA
GAAAACTCAAGTAATGGTGTTGAATCAATCAATTATGTTGACTATGCAGTATTGCAAGTAGCTATAGCAG
AACTGATAGAGTGTCTTGAGAATCCATATCAAGTGATTATAAAAGAGTATGTTGAGATATGTTACAGCAT
GGGTACTGAAGAAGGTTATAAGTTTATTAATGCAGTATTGCAAAATCTAGCTAAGTCGATTAGATGTGAA
GAGTAA

>lcl|NC_006570.2_cds_YP_170373.1_1253 [gene=ppnK] [protein=inorganic phosphate/ATP-NAD kinase] [protein_id=YP_170373.1] [location=complement(1479294..1480184)]
ATGACTTTTAAATATCATAAGGTTGCGATTGTTGGTAAGCATTATAAAAAAGAAGTAAGTCAAATGGTTG
AAACTTTATATGCTTATTTACAGCAACAAGGCTTAGAAATAATTATAGAAAATGATACAGCAGCAGATAC
TTCACTTGTAAATGTTGCTATTGCTAGTCTAAAAGAGATTGCATTAAGATGTGATGTTGCGATAGTGGTT
GGAGGTGATGGTAATTTTCTTAAGGCATCTAGACTTTTGGCTTTGTACAGTAATATCCCAGTTATTGGTA
TAAACAAAGGCAAACTAGGATTTCTGACAACTCTTGCTGCAGATGATAATGCTCTAAAGAATGATCTTTA
TGCGATACTGAAAGGTGATAGTTCAGTAACAAAAATGAGTATGCTAAAGTATCGTGTTGATAATAATTTG
CGTGCACCATTAGAAGCCTCAATTGCCTTAAATGAGATAGCTATAACAGCTAGTAGAGGTTTGATGTTTG
GTTTGAAAGTTTTTATTGATGGTAGGTACGCTTTTGACCAAAGAGGTGATGGGCTTATTGTTTCTACACC
TACTGGTTCAACAGCACATGCGATGTCAGCGGGGGGACCAATTTTAAATCCTAATCAAAATAGTGTAGTT
TTGGTACCAATATGTTCACACTCATTAAACAGTAGACCTTTAGTTATCTCAGATGAGAGTGTTATTGATA
TTTATATAACTGATTATAATGATCCTGAATCAGTCTTAAGTATTGATGGTAGACATGATACTATCCTCAA
AGCACATCAGAAAGTAACTATCCAAAAAGCGCGAAAGAAAGTTACAGTATTACATACAAAAGATTATAAC
TATTATGATACGCTAAGGGAGAAGTTGGGATGGAGTAAAGTTCTGTTTTAG

>lcl|NC_006570.2_cds_YP_170380.1_1260 [gene=rpoA2] [protein=DNA-directed RNA polymerase subunit alpha] [protein_id=YP_170380.1] [location=complement(1488773..1489729)]
ATGGCATTAGAAAATTTACTACATCCTACTAATATTAAAATTGATGAATATGCTAAAAATGCTACTAAGT
TTTCTTTTGAAGCCCTAGAGAGAGGGGTAGGTTATACACTTGGGTTTGCTCTTAAGCAAACTATGCTTTA
CTCTATTGCTGGTGCATGTGTTACTAGTATAAAAATTAATGATGGTAAAGTTACTTCTTTAGAAGATGTG
ATTCCATGTGATGAGACAGTTGCTGATATTATCCTGAACGTTAAATCTCTGCCTGTAACTTTAGCAGAAG
GTGTTGAAACGGGGACAATAACTTTTGAACTATCAGGCTCGGAAGAAGAAATTTTTTCAGAAGAAGCTAA
GCTATCTGAAGGTTTGGCAATAACTGAAGAAGTATTTATCTGTAGTTACAATGGTGGTAAAAAGCTAAAA
ATAGAAGCTAAAGTAGAAAAAGGCGTAGGCTTTAGACCTGCACAAGATAATTTTAAGGATGGTGAGTTCC
TTTTAGATGCGACTTTCTCACCTGTTGTTTTTTGTGATTTTGAGATTAAAGATGCTCGTGTTGGTAGACG
TACTGATCTTGATAAGCTTGAGCTTAATATTAAAACAAATGGTAATGTGAATTGCGAGGAAGCTTTAAGA
CTAGCAGCTACTAAAATCCAAAATCAATTAAGAAATATCTTAGATATTGAAGAGATTAATAAGGGTATTT
TTGTAGAAGATCCTACTAAAGATATAAATCCTATTTTATTAAAGCATGTTGAAGAATTAAATCTAACAGC
TAGATCTTCTAATTGCTTAAAAGCTGTAAATATTAGACTAATTGGTGAGTTAGTACAGAAGACAGAAAAC
GAACTTCTTAAAGCACCAAATTTTGGTAAAAAATCATTAACAGAGATCAAAGACAAGTTGTCTGAGCTTG

GTTTATCTTTAGGAACTCTTATTGAAAATTGGCCTCAAGATTTATAA

>lcl|NC_006570.2_cds_YP_170384.1_1264 [gene=rho] [protein=transcription termination factor Rho] [protein_id=YP_170384.1] [location=1492777..1494039]
ATGAACTTAAATGAATTAAAGTATAAATCTGTTAATGAGCTAATGGATATAGCTCAAAGTCTAGATCTTG
AGTCACTTCGTGCCAGAAAACAAGAGCTGATCTTCTCAATTCTAAAGTACCACGCTGATAAAGGTGAAGA
CATTTATGGTGAAGGTATTTTAGAAGTCCTACAAGATGGCTATGGCTTTCTAAGATCTTCTGATAGCTCG
TACTTCGCCTCTCCAGATGATATCTATGTTTCACCTGCTTTTATTAGAAAACTAAATCTGCGTACTGGTG
ATAGTATTGTCGGTAAGATTCGTCCGCCTCGTGAAAATGAGAAATACTTTGCTGTCAAACACATCGATAG
TGTTAACTTTGATTCTCCAGAATTAGCTAGAAAGAAAATTTTATTTGAGAACCTGACTCCTGAGTATGCT
AAAGAAAGGCTAACTATGGAAATTGGAAACGGCTCAAATGAGGATATTACTGCTAGAGTTATAGATTTAG
CGGCACCATTTGGTAAAGGTCAGCGTGGCTTGATTGTTGCACCACCTAAAACTGGTAAGACAATCATGAT
GCAAAATATCGCAACGTCTATTGCCAAAAATCATCCAGAGTGTAATCTAATCATGCTACTAATTGATGAG
CGTCCTGAGGAAGTTACCGAAATGCAACGCTCCGTGCGAGGTGAGGTAGTTGCATCTACGTTTGATGAAC
CAGCTGCACGCCATGTCCAATTAGCTGAAATTGTAATCGAAAAAGCCAAAAGATTAGTTGAGCACAAACA
AGATGTAGTAATCTTACTTGACTCAATCACAAGACTAGCTCGTGCATACAATACCGTATCTCCAGCGTCT
GGTCGTGTACTATCTGGTGGTGTAGAGGCAAACGCCTTACAAAAACCAAAAAGATTCTTTGGTGCTGCGC
GTAATACTGCCGAAGGTGGTAGCTTGACAATCATTGCAACTGCTCTTGTCGAAACTGGTTCAAAGATGGA
TGAAGTTATCTTTGAAGAATTCAAAGGTACAGGTAACATGGAGCTTCATCTTGATCGTAAGATTGCTGAG
CGCCGCGTCTTCCCTGCTATTAGCTTTGATAGATCTGGTACACGTAGAGAAGAACTACTTACTACTCCAG
AAGAGCTACAAAAACTTTGGGTGCTGCGTAAGATTCTTGGTGGTATGGAAGATGTCCAAGCTATGGAATT
CTTAACAGAGAAAATGAAAGGCTCACTAACTAATGAAGAATTCTTTGAAACTATGAAAAGAGGCGCTATT
TAG

>lcl|NC_006570.2_cds_YP_170388.1_1268 [gene=wbtL] [protein=glucose-1-phosphate thymidylyltransferase] [protein_id=YP_170388.1] [location=complement(1500602..1501486)]
ATGAAGGGAATAATTCTAGCTGGTGGCAGTGGTACAAGGCTATATCCACTTACCTTGGGTGTTAGCAAAC
AGCTGCTACCTGTTTATGACAAGCCATTGTTATACTATCCACTATCTGTGCTTATGCTTGCAGGTATTAG
GGAGATATTAATTATCTCTACAGTGCGTGATATCTCACTTATCCAAGAGCTTCTTGGTGATGGTTCACAA
TTTGGTATACAGTTGAGTTATAAAATCCAGCCATCACCAGATGGGCTTGCTCAAGCATTTATTCTTGGTG
AGGAGTTTTTGGCGGGTGACTCAGCTTGTTTGATATTAGGAGATAATATCTACTATGGTCAAGGTATGAC
TACAATGCTAGAGTCTGCAAGAGCACAGTGTGGAGGTCCAGCTGGTGGCGCTTGTGTTTTTGGTTATTAT
GTTAATGATCCGCATAGATATGGTATAGTCGAATTTGATAAGCAAAAAAATGTAATTTCGGTAGAGGAAA
AGCCACAGAATCCTAAGTCACACTATGCTATCACAGGTTTATATTTTTATGATAATAATGTTGTTGAGTA
TGCTAAACAAGTCAAACCATCTGCACGTGGTGAGCTAGAGATTACTTCACTTAATGAGTTATATCTAAAA
GAAAATAAGCTAAATGTCGAACTCTTAGGGCGTGGCTTTGCTTGGCTTGATGCTGGTACGCATGATTCAT
TGCTAGAGGCAGGTCAATATGTCGCAACTATTGAGAAAAGACAAGGGCTTAAAATTGCATGTTTGGAAGA
AATTGCATGGCGTAAAGGCTTTATCTCAACACAACAAGTTCTAGCTCAAGCTGAAAAACTTTCTAAGACA
GAGTATGGTCAGTATCTGAAGAATTTAATTAAGGATGGTTTATAA

>lcl|NC_006570.2_cds_YP_170389.1_1269 [gene=wbtK] [protein=glycosyltransferase] [protein_id=YP_170389.1] [location=complement(1501511..1502371)]
ATGATTAAAGTTTCAGTATGTGTGATGACATACAATCAAGAAAAGTATATTGGTCAATGTTTAGAGTCTT
TGGTTACTCAAGAGACTGATTTTGACTTTGAGATAATCGTTGGAGATGATTTTTCTACAGATGGTACAAG
AGATGTTATTCAAGAGTATCAAAAAAAGTATCCGGATATCATAAAGCCAGTTTTTAGAGATAAGAATGTG
GGAATTACTGAAAATATTAAAGAAATCTATTTTGTTGCAAATGGTGAGTATATAGCTCATATGGATGGTG
ATGATTATGCATTGCCTGGTAAACTTCAAATTCAGGCTGATTTTTTGGATAATAATCCAAGATGTACGGG
AGTTTTTCATAATATAAATATACTCTATCCAAATGGTAATATACAACATAGTAGGTTTGCTTGTTCAAAT
AAGAGTATATTCAATTTATCAGACACTTTACGCGGAGTTGCTGTTGGTGCAAATAGTTCAAAAATGTTCA
GAACATCGGTTTTGGATGATTTGATTTTACCGGATATAGAGCTTCTAGATTATTATTTTCATGTTATAAC
AGCAGAAAAAGGTTATTTAAGTTTTTTAAATTCTAATGAATCCTATAGTGTGTACAGAAAAGGTATTGGT
ATCACATCTAAGTCTAAGGAAAAAATCTATAATACTTATGCTGGATTATTTGAATATTTTTTGGATAGAT
ATCCTGAAGAGAAATTAAATATTTGTATCCCTGTTGTGCAAATGATAATTTCGGCTATTAAAGGGAGATG
TTTTATTAGTGCTATTCGTCTATTCAAAATTTTAATTAGATCAAGATGTATTCCATTAGTAAGTTGGTTT
AAATATAGATTTGAAAAATAA

>lcl|NC_006570.2_cds_YP_170390.1_1270 [gene=wzx] [protein=O-antigen flippase] [protein_id=YP_170390.1] [location=complement(1502364..1503851)]
ATGAGCCTTAAAAAAAAATACAATATCAAATTATATAACACAACTATATACTAGCTTAATTGGTATTGTTA
TACTTCCTTTGTATTTACAACATTTAAGTCATGATGCATTTGGTCTGATTGGTTTTTTTACAGTTTTTCA
AACGTGGTTACGGTTGTTGGATGTTGGTATAACACCAACTTTATCAAGAGAAGTGGCTCATGTTAGAGGT
AGTACTGATGACTATCATTACTTACGCAAGTTGGTTAGATCGTTAGAGCTATTTTTCATTATTGTTGGTG
TTCTGGTATTTATTGTAATTAGTACACATTCAAGGTATATATCCACCTCTTGGTTACATATAGGCTCGCT

AGATGCTGATAGTGTAAGTGTATGTATTGCACTTATGGGTTTAATGTTTGCATTAAGATGGGTGTCTGAT
CTATATGGTGGTGGTTTGCGTGGCTTTGAAAGACAGGTTCTTTATAATAATTTAAGTATCATACAAACGA
CACTACAGTTTATTGGTGGATTATTATTTATCTGCTATGTGTCTACTAATATTATGTATTATTTTGTATA
TCAGACAATAATTGCGATACTATATCTAGTATGTATTGCAATTGCATTTTATAAAATACTACCATCATCA
TTTAGCGTGGGTTTAAGGTTTGATTTTAAAATAATTAGAAAAGTGCTTCCATTTGCACTAGGCATTGCAT
ATTCTACAACAGTTTGGATTATTGTCACTCAATCTGATAAATTAGTGTTCTCACATGTATTACCATTATC
TGAGTATGGTTATTTATCTTTATTGATAGTGATATCTAGTGCTGTTACGATATTGTCCTCTCCGATTAGC
ATAGCTATTCAGCCTAGAATGACAATGCTATTAGCCCAACAAAATGTAAAAGGAATGGAAAGCTTATATT
TAAAATCATCCTTGATCTCAATTACTTTTTTATCTGCTGTAGTAACATGTGTTTTGATGTATTCTCATCA
GCTGTTGCAGTCATGGACAGGAAGTATGGAAATTGCTAATTGGGGTAGTAATATCTTAAATATATATGTT
TTATCAGCATCTATTATTTGTATAATATCATTTCAATATTTTTTACAGTATGCTTATGGTAAGTTAAAGC
TACATAATACATATAATACAATTAGTTTAGTATTTTTTGCTCCTATAGTTATATATACTGCTTATAATTA
TGGAGTGTATACTACAGCACTATTATGGCTTGGATATGCTATAGTGGGGCTGATAATCTGGATGCCTATT
GTACACCATGTATTTGCTAAAGGTATCAATAGGTATTTTTTTATAAATTTAGCAGTTATTACTATAGTAT
GTTTTTTATTATCGTTAATATTTAAGGGTTGGTATATTTATCCAAGTAAAATTGGGTTGGTAGAATTAAT
ATTGATTGGGTTTGCATTTTTATTTATACAAATTTGTATAGAGTATGTTTTGTTTCGGTACAAGGTTTTG
AGGTGTATAGATGATTAA

>lcl|NC_006570.2_cds_YP_170391.1_1271 [gene=wbtJ] [protein=hypothetical protein] [protein_id=YP_170391.1] [location=complement(1503856..1504581)]
ATGAAAAAAATATTTGTTGTTACAGATAATAGAACTATTCTAAGTGATTTTAAAAATATCATTGGTAGTA
AAAATGATGTAGAGGTTGATTATTTTTGTAGTTTCAAGAGTCAAACTTCTTTTGCCAAAGAAATATATAA
CAGTGAGATTAAGCCAATAGATATGAAAAAAAAATGGCAATGATCTTATTGGTAAGTATGATTTAGGTTTT
TCTTGTCATTCGAAACAATTATTTCCAGCAAAATTAGTTAATTCAGTATTATGTATAAATATTCATCCTG
GACTTAATCCATATAATAGAGGGTGGTTTCCACAGGTCTTCTCTATTATAAATAAACTACCTATAGGAGC
AACTATTCATGTGATGGATGAAGAGATAGATCATGGAGATATAATCATTCAGGAAGAAGTTGAAGTTAAT
TCTTTCGAAAACTCTTTTGATGTTTATGCTAAAGTTCAAAAAAAAGAAGTTGAGTTGTTCACTAAAGTCA
TAGATGATATTTTGAATAATAAGTTCACTCGAATCAAACCTAACTCCGAAGGCAACTATAATTCAATTCA
TGATTATAAAAACATGTGTGAAATTGATTTAGATAAAATAGTAACAATGCGGGAAGCAATTGACTATCTA
AGGGCTATGACACACCCTCCATATAAAAATAGTTATTTCATTGATGAGCATGGAAATAAAGTATTTGTTG
CTCTTGAACTTGAAAAGATAAGTTAG

>lcl|NC_006570.2_cds_YP_170392.1_1272 [gene=wbtI] [protein=sugar transamine/perosamine synthetase] [protein_id=YP_170392.1] [location=complement(1504578..1505660)]
ATGAGTAAAGTAAATGTAACAAAACCATACTTACCAGATATAAATAAATATAAAAGCTATGTAAATAAAA
TATACAAAAATGGATGGCTTACTAATAATGGTCCGTTAGTGCAAGAGCTAGAAAAAAGACTTGCAAAGTA
TCTAGGTGTTAAAAAATATAGTTTTAGTATCAAATGGTACAATTGCATTAGAAATCGCGTATAGAGCGTTA
GGAGTCAAAGGAAGTGCAATTACTACTCCATTTTCATTTGTTGCTACTACATCTTCATTGGTTTCTAACA
ATGTAAAACCAGTGTTTGTTGATATTGATGAGAATACTCTAAGTATAGACGTCTCTAAAATTAAGTATGC
TATTGAAGAGGATACTTCAGCTATTGTGCCAGTTCATGTGTTTGGAAATGGTTGTGAAGTTGAAAAAATA
GACATGCTGGCTAAAAAACATAACTTAAAAGTTATTTATGATGCAGCACATGCTTTTGATGTTAAGTATA
AGGGTGAGAGTATATTAAACTATGGTGATATTTCGACATTAAGTTTTCATGCAACAAAGATTTTTCATTC
TATTGAAGGAGGTGCGCTTATCATTAATGATGATAGTCTTGTTGAAAAAGTTCGTTATTTCATTAATTTT
GGTATAGAAAGCTCAGAATCAATACCTTACTTAGGTACTAATGCTAAAATGAATGAATTTGAGGCGGCTA
TGGGACTTTGTGTTCTAGATGATATTATAGAAATTAAGAGCAAAAGGAAAGTTATTACAGAGATATATGA
GGCTGGGTTAGATGGATTGGTAAAGTTTCAAGAACAGAATCAGCATTCTAGTAGGAATTATAGCTATTTT
CCAGTAATATTTAGGACTGAGGAGGAACTTCTCAGAGTACAGAAAGCACTAATACAAAATGATATAATAT
CGCGTAGATATTTTTATCCATCATTAGATAGTCTTAGTTATATAGAGCCAAAGCAGTATATGCCAATCTC
AAGAGATATATCTAAAAGAATATTATGTTTGCCAATTTATGCAGAGTTAGAAGACGATAAAATTAATAAA
ATAATTAATAATATCAAAGAGGTTTCCTCATGA

>lcl|NC_006570.2_cds_YP_170393.1_1273 [gene=wbtH] [protein=asparagine synthase] [protein_id=YP_170393.1] [location=complement(1505653..1507539)]
ATGTGTGGAGTAGTAGGCTTTTACTCATTTAATAAAGAAGAAGGTTTTGACTCAATAATTAATCAATCAT
TGCTTTCTATAAAGCATAGAGGGTCGGATGATAGTGGGTATTGGTGCGACAATCAAGTTACTCTGGGGCA
TACTAGATTATCAATACACGATATAACTAATGCGGGACATCAGCCAATGTTATCTAATAGCGGTAATACT
GCTATTGTGTTTAATGGAGAAATATATAATTACTTATCCATAAAAAATCAGCTATTAAGTGAATATTCAA
ATCTTAAATTTAAAAGTAACAGTGATACTGAGGTTTTGGTCAATGCTATTGAACTTTGGGGTATAGATAA
AACTTTAGAAAAAATGCATAGGAATGTTTGCTTTTGGAGTTTACAGTAGAAAAACTAGTTGCTTAATACTA
GCTAGAGATAGATTTGGCGAGAAGCCATTATATTTTGGTATCCAAAATGGTATTTTGGGTTTTGCATCAG
AATTGAAGGCACTTAAGCCATTAAAGGAATGTGGCTGGAGGTTTGATATAGATAGAGATGCTTTAGCAAC
ATATATGAGGTATGCTTATGTACCAACACCATACTCTATTTATAAAAATATATCTAAACTAAATGTAGGT
AGTTACATAAAATTTGATGCTAAAGGTAATAGTAAAGAGTATAAATATTGGGATTCTAAAAAAGTACTAG

ATTCAGAAAAATATAAAGATTCGTATGATCAAGCAATCCTAGATTTAGAAATTAAGCTTAAAAGTACACT
ATCAATACAAATGCAGTCAGATGTTCCTCTAGGAGCATTTTTATCCGGAGGAATTGACTCAACAACTGTA
GTTGCTCTTATGCAAAGTATGTCTAAAGATAAGATAAACACTTTTAGTATAGGTTTTAATCAAAAAGAAT
ATAATGAAGCTGAGCATGCAAGAGCAGTAGCAAAACATATAGGTACAAACCACACAGATATGTATGTTAC
AGAAAGAGATGCTCTTGATGTAATACCAAAACTTGCTGGAATATATGACGAGCCCTTTGCTGATTCATCA
CAAATACCAACGTATCTTGTGAGTAAAATAGCTAAGTCGAAAGTAACAGTTGCACTATCAGGTGACGCTG
GTGATGAGCTCTTTGGCGGTTATAATAGATACTTTTTAGCACCAAATATTGCTAAAAAAATCAAATTTGC
TAAGTTACTTAAATATGCACCAGATGCTTGGATAAAAAAAGCTGAGATATTAAATTTTGGTAAGTTCGCT
TTATTAGCAGATAAACTACTAAAACTAAAAAGAGTTCTCGAAAAAGCAAAAACAAATAAAGAGCTTTATG
TACTACTTTGTTCACAAATAAATGATACTAGCTTTGTGTTAGGAGCAAAAGAGTATGATATATTAAGAGA
TAAGAATATTTATGATATTCCACAATTATCTTTCCAAGAGTGGATGATGTTTGTTGATTCTAATACATAT
ATGATAGATGATATATTGGTTAAGGTTGATAGAGCAGCTATGGCTAACTCTCTAGAGACAAGAGTGCCAT
TTTTAGATCATAATATTTATGAATTTGCTTATTCCTTACCAATTGACTATAAAATACAACGAGGTAACGG
AAAAAGAATTTTGAAAGATTTGTTATATAAATATGTGCCAGAAAGTTTGGTCAATAGGTCTAAGATGGGG
TTTGGTATTCCGCTTGCTAAATGGTTAAGAGAAGATTTACGAGAGTGGGCAGATAATTTACTGGATTATA
GTAAAATAGACAAGCAAGGTTACTTAAGTCCTGAGGTGGTGCAAAAATATTGGCAAGAGCATTTGAGTGG
TAAAAGAAATTGGCAAGCAATATTATGGAATATTCTAATTTTTCAGGAGTGGTTAGATAATGAGTAA

>lcl|NC_006570.2_cds_YP_170394.1_1274 [gene=wbtG] [protein=glycosyl transferase family protein] [protein_id=YP_170394.1] [location=complement(1507539..1508639)]
TTGAAAAGGTTTGTACATTTAATAATAAACCTTAACCAAGGTGGTGCTGAAACAATGCTTTATAAACTTT
GCAAATCTATGGATAAGTCAATATATCATATTACGATTATATCACTTATGGGTAGGGGAGTATTTGCAAA
TAAGTTAGAAGCTTATGGTGTTAAAGTTTATACATTAAATTTAAATAAATTTAATGTACTATTTGTATTG
TTTAAATATATTAAGATTATCAGAAGAATAAAGCCTGATGTTATTCATGCTTGGATGTATCATGCAAATG
TAATTTCTATATTATGCAAGCCTTTTTATAGAAAGACTAAATATATAAATAGTATAAGAATGGGATTGGA
GAATTATGATGGTCATAAGAATCTTACAAAGTTTATGATAAAGTTGAATGCAAAATTTTCTAAGTTCTCA
GATTTAACATTAAATAATTCAAAGAAATCATTAGAAGATCATCAAAATATAGGTTTTAAAAACCAATGCT
TTATAGCAAATGGTTTTGATAAAGATGTTTTTAAACCGAGCTTTTTAAAGTATGAAAAATTTCGTTTAAA
TAATGATTTAGATGATAATGTTAAAATTATAGGTATCATAGCAAGAAATCATGCTGATAAAAATATTTCT
CGTTTCTTACAAATAGCTAATTTATTGTTAAAAAGTAATCCTAGTTTACGGTTTTTAATTGCTGGAAGAG
AGTGTTCGAAAATAGATATAGGTAGTTATCTAGATAACAAAAGTAATGTAAATAAGTTTTTTGTATTTGA
ATCTGTGGATTCTAGTGAATACTTACCAGTATTAGATTTATATTTGTCTACATCAAAAGTTGAAGGTTTT
CCAAATATACTTGCAGAAGCCATGCTATGTGAAGTTCCTATTGTTGCTTCTAATGTTGGAGATTGTAAAG
ATATACTTAATGGATACGGTGAAGTTTTTGAGCTTAGTCAAGGTAATAAAGAAATAATAGAAAAGATTAT
GAAAGTTTTAGAAACAACGGTAGTCATGAAAAAGCGCATGAGAGAATATATAATAAATAATTTTAGTATA
GAAGCTATTTTGGAAAAACACGAAAAACTTTATCATGAGGGCAGTGTCTAA

>lcl|NC_006570.2_cds_YP_170396.1_1276 [gene=wbtF] [protein=NAD dependent epimerase] [protein_id=YP_170396.1] [location=complement(1509882..1510853)]
GTGGCTTACGATAATGTTAAATTTCCTCATGGTTCGTTTTTTTTGGTGACTGGAGGTGCGGGTTTTATTG
GCTCTAATTTATGTGAAGTTTTACTTAGTAAGGGTTATAGAGTTAGGTGTTTAGATGATCTCTCAAATGG
TCACTATCACAATGTTGAGCCGTTTTTAACTAATTCTAATTATGAGTTTATAAAAGGTGATATTAGAGAT
TTAGATACTTGCATGAAAGCTTGTGAAGGTATTGATTATGTTCTACATCAAGCTGCTTGGGGAAGCGTAC
CAAGAAGTATTGAGATGCCATTAGTGTATGAAGATATAAATGTTAAAGGTGCATTAAATATGCTTGAAGC
GGCTAGACAAAATAACGTTAAAAAATTTGTCTATGCTTCTAGTTCATCAGTATATGGTGATGAGCCAAAT
TTACCTAAAAAAGAAGGTAGAGAAGGAAATGTTTTATCACCCTATGCATTTACAAAGAAAGCTAATGAAG
AGTGGGCGAGACTATACACAAAGTTATATGGTCTAGATACTTATGGTCTAAGATATTTTAATGTTTTCGG
TAGAAGACAAGATCCTAATGGTGCGTATGCAGCAGTTATACCTAAATTTATCAAACAGTTATTAAATGAT
GAAGCGCCAACTATAAATGGAGATGGTAAACAGTCGAGAGATTTTACATATATAGAGAATGTTATTGAGG
CAAATCTTAAAGCATGTTTAGCAGATAGTAAGTATGCCGGAGAGTCTTTTAATATAGCTTATGGAGGTAG
AGAGTATCTTATAGATTTGTACTATAATCTTTGTGATGCCTTGGGTAAAAAAATAGAGCCAAATTTTGGT
CCAGATAGAGCGGGTGATATTAAGCATAGTAATGCTGATATTTCGAAGGCTAGGAATATGCTCGGATATA
ATCCGGAATATGATTTTGAATTAGGCATAAAGCATGCTGTTGAGTGGTATTTAATTAATTAA

>lcl|NC_006570.2_cds_YP_170397.1_1277 [gene=wbtE] [protein=UDP-glucose/GDP-mannose dehydrogenase] [protein_id=YP_170397.1] [location=complement(1510853..1512163)]
ATGAGTTTATATGAGGATATAGTCGCTAAAAGAGAAAAGGTTTCATTGGTTGGCTTGGGTTATGTTGGTT
TACCAATAGCTATTGCATTTGCAAAAAAAATAGATGTGTTAGGATTTGATATTTGTGAAACAAAAGTTCA
ACATTATAAGGATGGTTTTGATCCAACAAAAGAAGTAGGAGATGAGGCTGTCAGAAATACGACAATGAAA
TTTAGTTGTGATGAAACAAGTCTTAAAGAGTGTAAATTTCATATTGTTGCAGTTCCTACACCAGTTAAAG
CAGATAAAACTCCTGATTTGACGCCGATTATTAAGGCAAGTGAGACGGTTGGTAGGAATCTTGTCAAAGG
CGCTTATGTTGTGTTTGAATCAACTGTTTATCCTGGTGTTACAGAAGATGTTTGCGTACCAATACTTGAA
AAAGAGTCTGGCTTGAGGTCTGGTGAAGATTTCAAAGTTGGTTACTCTCCTGAGAGGATAAATCCTGGTG
ATAAGGTTCATAGGTTAGAAACAATTATCAAAGTAGTATCTGGTATGGATGAAGAGTCTTTAGATACTAT

AGCAAAAGTTTATGAGCTAGTAGTAGACGCAGGAGTTTATAGAGCTAGTAGTATAAAAGTGGCTGAAGCT
GCTAAGGTTATAGAAAACTCTCAAAGAGATGTTAATATAGCTTTTGTTAATGAGTTATCGATAATATTTA
ATCAGATGGGTATTGATACTCTAGAGGTTTTAGCAGCAGCTGCAACTAAATGGAATTTCTTAAACTTTAA
GCCTGGTCTTGTTGGTGGACATTGTATTGGTGTTGACCCATATTACCTAACGTACAAGGCAGCTGAGCTT
GGATATCATTCTCAGGTAATATTATCTGGTCGTAGGATAAATGATAGTATGGGTAAATTTGTAGTTGAGA
ATTTAGTCAAAAAACTGATATCTGCAGATATACCTGTTAAGCGAGCTAGAGTAGCAATTTTCGGCTTTAC
TTTTAAAGAAGACTGTCCTGACACTAGGAATACTCGAGTTATAGATATGGTAAAAGAGCTCAACGAGTAT
GGTATAGAGCCATATATTATAGATCCGGTAGCTGATAAAGAAGAGGCTAAACATGAGTATGGACTTGAGT
TTGATGATCTAAGTAAAATGGTCAATCTAGATGCGATCATTATTGCTGTTAGTCACGAACAGTTTAAAGA
TATAACAAAGCAACAGTTTGATAGGCTATATGCGCATAATTCTAGAAAGATTATATTTGACATCAAAGGT
AGTTTAGATAAATCTGAGTTTGAAAAAGATTATATTTATTGGAGATTGTAG

>lcl|NC_006570.2_cds_YP_170398.1_1278 [gene=wbtD] [protein=galacturonosyl transferase] [protein_id=YP_170398.1] [location=complement(1512181..1513272)]
ATGAGAAGTAAGTTATTATTCATAGCTAATGATTTTGATATTGTAATATATCGTTTCAGAAGAGAAGTAA
TCGAGTCTTTTGCTGCTAAAGAGTATGAGATAGTACTAGTAACACCATATTCTAAGAAAGCAGAGGTTTT
TTGTAAAAGTCTTGGTGTTAAGTATATAAATGTTGATATAGATAGACGAGGCAAAAATCCTTTTAAGGAT
TTGCTTCTTTTATTTAACTATTTCAAAATAATAAAAAAAGAAAAACCTGATTACATTTTTAGCTATACAA
TTAAACCAAATTTGTATGTTGGGTTAGTGAATTTGTTTTTAGGAAGAAGTTTTATCCAAATGTAACAGG
CTTAGGAAGTGTTTTTGCTAATCATGGTATTGTTCAGAAGTTTATAATATCTTTATATAAGTTATCATTT
AAAAGCACCACAAAAGTATTCTTTCAGAATGAGCAAAATAAAAAGTTATTTATAGCTAAGAAAATAATCA
GTGGAGAAAAATCAATATTATTACCAGGTTCTGGGGTAAACTTAGATGAAAATAAATATGTTGACTATCC
TAAAGACCAAGGAATATTAAAATTCGTTTTTCTTGGCCGAATAATGAAAGAAAAGGGGATTTATGAATTG
TTAGAAGCCTTTGCTATACTTGAGAAAAAATATAAAAATATTAGTCTTGACATTTATGGTTTTTGTGATG
AAAATAAATCTAATTTTATGGGAAAGGTTAATACGATAAAATCAGTAAAATTTTATGGTTTTACTGATAA
TACTAAAGAAAAAATAGCTAGTGCACATGCAGTTGTTTTGCCATCTTACCATGAAGGAATGTCAAATGTG
CTGTTAGAAGCAGCTGCGATAGGTAGACCTGTAATTGCGTCAGATATTCCTGGGTGTAGAGAAATTTTTG
ATGATGGTCTCTCTGGCTTATCATGTAACCCTAATGATGTGAGTTCTTTACGTAACTCATTAGAGCAGTT
TATAAATATGTCGTATACTGATAAAATAGCTATGAGCTATAAAGCTAGAGCTAAGATAGAAAAAGATTTT
GATAGAAGTATTGTTGTCAATGCATACTTACAGCAAAATTAA

>lcl|NC_006570.2_cds_YP_170406.1_1286 [gene=gmk] [protein=guanylate kinase] [protein_id=YP_170406.1] [location=complement(1521774..1522346)]
ATGAATAACTATATTTTTATAGTTTCTGCACCATCTGGCGCGGGTAAAAGTTCTTTACTAAAAGCTTTTT
TAGCAACTGACATAGGCAAGGATAACTACGCTGTAGCAATCTCCCACACTACTAGAGAGCCACGTGTTGG
CGAGATCAATAGTAGAGAATATTATTTTGTCACAGTAGCTGAATTTGAACAATTACTTAGTCAAGATGGC
TTTATTGAATATGCTAAAGTTTTCAAAAACTATTATGGTACATCTAAAGCTGAGCTTGATAGATTGCTTG
CATTAGGTAAGAATATTATCCTTGAGATTGACTGGCAAGGTGCACAACAAACACGTGCTATTTATGGCGA
TAGGGCTAAGAGTATATTTATATTACCGCCTTCTTTAGATGAGTTAAGAAAACGTCTAGAAAAAAGAAAT
ACAGATTCTAAGGAGACTATAGATTATCGTATGGAGCAAGCACAATCAGAGATTTCACATGCTGATGAAT
ATGATTATCTGCTGGTTAACGATGATTTTAGCCAGTCATTAGAACAATTCTGTAAATATTTCGAACAAAA
TATCCAAAGTTAA

>lcl|NC_006570.2_cds_YP_170408.1_1288 [gene=ppiC] [protein=peptidyl-prolyl cis-trans isomerase] [protein_id=YP_170408.1] [location=complement(1524237..1524515)]
ATGAAAGCTTCAGCTAGACATTTGCTAGTACAATCAGAATCTGAATGTCAACAAATCAAAAAAGACATAA
CTGAAGGTAAAATAACTTTTGAAGAAGCGGCTAGAAAGCACTCTCTATGTCCATCTGGAGCTAGAGGCGG
AGATCTAGGAACCTTTTCTCAAGGACAAATGGTTCCTGAGTTTGATAGAGTAGTATTCAACGATGAATTA
CATAAAGTTCATGGTCCAGTACAAACTCAATTTGGTTATCATCTATTAGAAATTACATCGCGCGGATAA

>lcl|NC_006570.2_cds_YP_170418.1_1297 [gene=lpd] [protein=dihydrolipoamide dehydrogenase] [protein_id=YP_170418.1] [location=complement(1532885..1534297)]
ATGAGTGATATTAAAACACAAGTTGTAGTTTTAGGTAGTGGTCCTGGTGGATATAGTGCAGCTTTTAGAG
CAGCTGACTTAGGATTAGAAGTTGTTTTAGTAGAGAGATATGCCGAGATCGGTGGTGTGTGCCTAAATGT
GGGATGTATCCCATCAAAAGCTATGTTGCATATTGCCAAAGTTATCAATGAGGCTCGTCATCTAGAATCT
CTTGGTATAATCGAGATGGGTGGCCTAAAAATCAATAGAGAAAACCTCTTAAAATATAAAGATGGGGTTA
TCGGTAAGCTTACTGGTGGTCTAAAGGGTATGGCTCAGATGAGAAAAGTAAAAATCGTACAAGGTTACGG
TAAATTTACTTCTGATAAAGAACTTGCTGTTGAGGCTGCTGATGGTAAAGTTACAAAAATTGCTTTTGAT
AACTGTATTATTGCCGCGGGTTCTAGTGTTATTAAGCTACCTTTTGTACCAGAGGACGATAGAATTATTG
ACTCTACAGGCGCTCTTGAGATGAAAGAGATTCCAGAAACTATGCTTGTAGTTGGTGGTGGAATTATCGG
TCTTGAGATGGCACAAGTATATTCTGAGTTAGGTACGAAGATCACGGTTGTTGAGTTTGCTGATCAGCTT
ATGAATGGTGTCGATAAGGATCTAGTCAAAGCTTATCAAAAAGTAAATAGTCGTTATGATGTGCGCCTAA
AAACAGCTGTAACAGCTATGGAAGCTAAAGAAGATGGTATCTATGTAACTATGGAAGGCGATCATCCTGC
AAAAGATGAGAGATTCGATAGAGTACTTATGGCTATTGGTCGTAAACCAAATGGTAAACTAATTGATGCT

GAAAAAGCAGGTGTTAAAGTTGATGAGAGAGGCTTTATCCCAGTAGATAAGCAATTACGTACAAATGTGC
CTCACATTTTTGCTATTGGAGATATTGTTGGTCAACCTATGCTTGCTCATAAAGCTGTACCGGAAGGTAG
AACAGCTGCTGAAGTAATATCAGGACTAAATCATAGTTTTGATCCTTTAGTGATTCCTTCAGTTGCTTAT
ACTGATCCAGAGGTTGCTTGGGTTGGTGAGACTGAGACTTCTGCAAAAGCTAAAGGTATCAAGTATGAAA
AAGGTGTATTCCCATGGGCAGCTAGTGGTAGATCGCTAAGTATCGATAGATCAGAGGGTATGACAAAAAT
TCTATTTGATGAAAATCATAAGATTATTGGAGCTTCTATCGTTGGTACTCATGCTGGCGAGCTTATTTCA
GAAGCAGCTATTGCAATAGAAATGGGTTGTGATGCTGAAGATATAGCTCTTACAGTACATCCACATCCAA
CTCTATCTGAAAGCTTAATGATGGCTACAGAAGTTTATGAAGGTACTGCTACAGATCTTCCGCCGCAAAA
GAAGAAAAAGTAA

>lcl|NC_006570.2_cds_YP_170419.1_1298 [gene=aceF] [protein=dihydrolipoamide acetyltransferase] [protein_id=YP_170419.1] [location=complement(1534311..1536206)]
ATGTCTATAGAGATAGTTAAAGTCCCTGATATTGGGGATTATGATAACGTTGATGTGATCGAGGTTAATG
TTGCTGTAGGTGATGTTATTGCTGAAGAAGATTCACTAATTACGTTAGAAACAGATAAAGCAAGTATGGA
GGTTCCATCTCCATTTGCAGGTAAGATCACTAAATTAACTGTAAAAGTTGGTGATAAAGTTTCTCAGGGA
ACTGCAATAATGGAAGTTGAGGTTGAGAGTGCTGCTGATCAAGCTGCTACTACACAATCACAACCTCAAA
CAACTAGTTCAGCTCCTGTAGCTGCTACGACGAACCTGATTGTTGATGTCGAGGTTCCAGATATTGGCGA
CTATGATAGTGTTGATGTGATTGAGGTGTCTGTAAAAGTAGGTGATGAGATAGCAGAAGAGGATTCGCTA
ATTACGCTTGAAACATATAAGGCTAGTATGGAAGTACCATCGCCTGTAGCGGGTAAGGTTGTTGAAGTTA
TTACTAAGGTTGGTGACAAGGTTTCACAAGGTAGCTTGATTTTAAAGGTTGAAACAGGCTCTAGTGCACA
AGCTCCAGCTCAAGAACAGTCACAACAATCTGCACCAGTTAAATCCGCTGCTGAAGAAATTATTGATGTA
AAAGTTCCTGATATCGGTGATTACGATAGTGTTGATGTGATTGAAGTATCTGTAGCTGTTGGTGATAAGA
TTGAAGAAGAAGATTCTTTGATTACATTAGAAACTGATAAGGCAAGCATGGAGGTTCCGTCTCCAGTTGC
TGGTGAAGTTGTTGAAATAATCACAAAAGTTGGTGATAAAGTTTCACAAGGTAGTTTAATTCTTAAGGTC
AAAACACAAGGTTCAGCACCGGTAGAGCAAACTAGTTCACAACCAGCGCCAGCTAAACAAGAGCAAGCTA
AACAGCAAGCTGCTACACCTGCTGCTCCAACGCCAGCATCAAGTTCGGTAAATGAGTATGCTGTAGATAA
TTCTAATGCACATGCGTCTCCTGCAGTGAGAAAGCTAGCACGAATTCTAAATATTGATCTAAGTAAGGTT
AAAGCTACAGGGCGTAAAGGTCGTGTAACAAAAGAAGATTGTTATAACTATATTAAGCATGCTGTCACAC
AAGTTCAAACTGGTAAGGTCGCTGCTAGTGGTAGTGGTTTAGATCTTTTAGATGATCCTGTTGTTGATTT
TGCTAAGTTTGGTGAGATTGAAACTCAACCATTATCAAGAATTAACAAGATTAGTGCTAAGAATTTACAT
CGTAACTGGGTGAAGATTCCTCATGTTACATTCTATGATGATGCGGATGTCACAGACTTAGAAGAGTTCA
GAAATGCTAAGAAAGCCTTTGCTGAGAAAAAAGGTATTAAGATTACACCTTTATCATTCTTGGTTAAAGC
TGCTGCAGTTGCATTACAAGAGTTCCCAAGATTTAATAGCTCATTATCAAATGATGGTGAGAACTTAATT
ATCAAGAAGTATTATAATATTGGTTTTGCTGCAGATACTCCAGCTGGTTTAATGGTTCCAGTTGTCAAAG
ATGCTGATAAAAAGGGTATCATTGAAATATCAAAAGATATTATGGAGTTGGCTGGCAAAGCTCGTGATGG
TAAACTTGGCGCAAAGGATATGACAGGTGCTACATTTACTATCTCAAGCTTAGGCGTGTTAGGTACTACG
TCATTTACGCCTATTATAAATATGCCAGAGGTGGCTATTATGGGTGTATCTAAGACAGCAGTGAAGCCTA
TTTGGAATGGTAAAGAGTTTATTCCTAGAACTATGCTACCATTATCATTATCTACAGATCATAGAGTGAT
AGATGGCGCATTAGCAGCTAAATTCTTAACTAGATATTGTCAGATTTTATCTGATTTACGTGAAATCATA
ATGTAA

>lcl|NC_006570.2_cds_YP_170420.1_1299 [gene=aceE] [protein=pyruvate dehydrogenase subunit E1] [protein_id=YP_170420.1] [location=complement(1536224..1538905)]
ATGTCGGATTTCACTAAAGATATTGATGTTTTAGAAACCCAAGAATGGCTTGAGGCTTTTGAAGATGTCA
TAAAAAGAGAAGGTGCTGATAGAGCCAAGTTTTTATTTGAACAATTACTTGCTAAAGGTTCCGAGCTTGG
TATAAAAAGTGCTTACTCAACTGCTAAAGTTAAGAACTATGTCAACTCTATAGATGTCTCTGAACAACCA
AGTTATCCAGGAGATATAGAGCTTGAACAAAAGATTGAAGCTATCAATAGATGGAACTCTACAGTTATAG
TAGCTGCAGCTAATAAGAAAGATGGTTCTATAGGTGGACATATTGGTACTGGTGCCGGCGCTATGACGCT
TTATGAAGTAGGTTTCAACCATTTTTGGAAAGCACCCAATGATGAACATGCTGGTGATTTGATATTCTAT
CAAGGACATCTTTCTCCTATTGTTTATGCGCGTTCATTCCTAGAAGGTAGAATAACTGCAGAACAGTTAG
AAAACTTTAGAAAGCAAGCATTTAATGGGCAAAATGCGGTATCTTCATATCCTCATCCTTATCTACAACC
GACTTATTGGCAGTTCCCAACTGTTTCTATGGGGTTAGGACCACTTCAAGCTATTTATCAAGCTAGATTT
ATGAAGTATCTAGAGGCTAGGGGTTTAGCAAAAACATCTGATCGCAAAGTTTGGGCATTCTGTGGTGATG
GCGAGATGGATGAGCCAGAATCAATTGGTTCTATCACTAGAGCAGGTCGTGAAGGGCTGGATAACCTTAT
ATTTGTAGTTAATTGTAATCTACAAAGGCTAGATGGTCTGGTAAATGGTAATGGTAATATTGTTGAAGAA
TTAGCAGATGTATTTGCTGGTGCTGGTTGGAATGTTATCAATGTGTTGTGGAGTAGTGACTGGGATAAAT
TATTACAAGATCCAAGAGGTGGTAAAAAACTTGCTCAAAGGTTAAGTGAATTAAATGATGGACAATTCCA
TACAATTAAAGCTCATGGTGGTGCTGAGTGTCGTAGAGTTGTCTTTAGCGGTGATGAAGATTTAGAAGCG
CTAGCTAAGGATATGACAGATGATGATATTCAAGCATTACGTCGTGGAGGCCATGATCCATTGAAAATTT
ATGCAGCATATAAGAAGGCGGTAGAGAATCCAAATGGTCGTCCAACACTAATTCTTCCTATGACTGTTAA
GGGCTATGGTTTAGGGGAGTGGGGTGAGTCAAAAAATATTGCCCACAATGTCAAAAAACTTGATACTGAT
GCACTTGAGCACATCAAAAATAGATTCAATGTCCCGGCTTCAAAAGAGGATATCGAAAACTATAGACTCA
TCAAGCTAGATGAAAATTCGCCTGAGATGAAATATCTACACTCTAAGAGAAAAGACCTAGGTGGTTATAT
TCCTAAGAGATTGGAGAATAAGCAAGCATTAGAGATTCCAAGCTATAAAGATTTTGCTAAGAGCTTATTG

GATGATAGTGGTGATAGAGAATTTTCAACTACTACCGCATTTGTAAGAATATTATCTCACTTGGCAAAAG
ATAAGAATCTAGGAAAGCATATTGTACCAATCACGGTAGACGAGTCACGTACTTTTGGTATGGAGGGCTT
GTTTAGGCAGTTAGGTATCTATAACCCACATGGTCAGCAATATGTGCCAGAAGATAAACAGCAAGTAATG
TTCTATAAAGAGTCTGTAGATGGTCAGATTTTACAAGAAGGTATTAATGAGCAAGGAGGTTTTTGTTCTT
GGATTGCCGCAGCTACTTCATATAGCGTACACAAGGTTCCAATGATTCCTTTCATGATTTACTACTCAAT
GTTTGGTTTCCAAAGATTTGGTGATCTTGCTTGGGCAGCTGGCGATTCGATGGCAAAAGGTTTTGTAATT
GGTGGGACTTCAGGACGTACAACTCTAAATGGCGAAGGTTTGCAGCATGAAGATGGTCATAGCCATATCC
AAGCTGGTCTTATACCTAATTGTATCTCTTATGATCCTACATATGCTTATGAATTAGCTGTAATCCTTGG
CGAAGGTATGAAGAGAATGTATGTTGATGGTGATCATGTATATTACTATATTACAGTAATGAATGAGAAC
TACTCTCATAGAGCGATGCCAGAGGGCTGTGAAGAAGGTATCATCAAGGGCTTATATAAGTTAGATGAAG
AGAAAAAGCCAGCATCTAAGCATGTACAGCTATTGGGTTCTGGTTCTATTCTAAGAGAAGTTGAAGCTAC
AGCTAAGATGCTAAAAGAAGAGTATGGAATTACTTCTAATGTTTGGAGTATGACTTCAGCAAATGAGCTT
TATAGAGAAGCTAAAGATGTAGCTAGAGCTAATATGCTTCACCCAACAGCAGAGAAAAAAGAAAGCTATA
TCGAGAAGTGCTTTAAAAATGAGCAAGGTCCTGTAATCGCTTCTACGGACTATATTAAGCTTTATACTGA
TCAGTTAAGAGAATTTATACCGCATACTTTTGTGAACTTAGGTACAGACGGCTTTGGTAGATCTGATACT
CGAGCAGCGCTAAGAAGTTTCTTTGAGGTTGATAGATATCATGTCGTTGTTGCTTCATTATATGCTCTAT
ATTTAGATGGCAAAGTTGAGGCTAGCGAAGTTAAAACTGCGATTGAGAAGTACAATATAGATCCAGAGCG
TATGGCACCTTTTTATAGCTAA

>lcl|NC_006570.2_cds_YP_170421.1_1300 [gene=FTT_1486c] [protein=hypothetical protein] [protein_id=YP_170421.1] [location=complement(1539205..1540341)]
GTGTCGCTTAAAAATATTATCTTAGAGCGGATAAAATCTTCTAAACAGCCATTACTATTTAGAGATTTTA
TGCAGATGGCTTTATACTATCCTCAGTTGGGTTATTATTCTAGAGCAAAAGAAAAAATCTCCTCTCAAGG
TGATTTTATTACTGCTACATCGCAAACTTCTTTATTTGCGCGAACTTTTGCTAGACAGTTTGTAACTATA
ATTTCTCAACTTGGTAACGACTGTAGTGTCATAGAATTTGGCGCTGGTAATGGTAAGTTTGCTGCAGATT
GTGTTGATGAACTTGAGAGTTTGGCAATATTGCCAAAACGCTACATAATTGTAGAGTTAAGTAATGATTT
AAGATTAAGGCAGCAGCAGTATATCAAAGAGAATCTATCGCATTTATATGATAGATTTATCTGGCTTGAT
AAATTGCCTGCAGAAAAAAATTAAGGCTATTGTCTTTGCCAATGAGCTTTTAGACGCGATGCCAGTTGATA
TTTTTAGATCTGAAAATAATAAACTAATACAGCAAGGGGTTATTAGAAAAGGTGATACTTTTGAATTTTC
TGATATGCCAAAAAATGATGTCCGTTTTGAGTATGAATCAACTAAAATACTAAATGATGGTATTACTTTT
AATGATGGTTATACTAGTGAGATAAATACTTGGATTCGCCCATGGGTTAAGTCTTTGCGCGAGGTTTTGT
CACAGGGTATAGTTTTCTTATGTGATTATGGTTACCATAGGTCTCTGTATTATTCAAAAGATAGATATAT
GGGGACTTTAGCATGTTATCATCAGCATCATGTTAATTTTGAACCATTTATAAATATTGGTGAGCAAGAT
ATTACCGCGCATGTTGATTTTACAACTGTTGCGGAGGCTGCTATTGAGGAGGGGTTTCAACTTGATGGTT
TTATGACGCAAGCAAATTTTTTGAAAAGAGCTAATATAGCTGAAGTTTTTAGTAATATATCTCAACGTTT
GAGTACAAATCAGCTTTTAAAATATAGTAATGATATAAAAGATTTACTTCTTAATGATAAGCTCGCTGAA
GTCTTTAAAGTTATGGCTTTTTCTTTAGATTTTGATTTTGTTTTAGAGGTGTTTGATAATCAGGATAATA
TTGATTATTTATTATAG

>lcl|NC_006570.2_cds_YP_170422.1_1301 [gene=coaE] [protein=dephospho-CoA kinase] [protein_id=YP_170422.1] [location=1540469..1541083]
ATGAATTTTATAAATACTTATCCAATTGGTATAACTGGCGGTATTGCAAGTGGTAAATCAACAGCAACAA
GAATTCTAAAAGAGAAGCTAAACTTAAATGTTGTCTGTGCAGATACCATAAGTAGAGAAATCACTAAAAA
ACCCTCGGTAATAAAAAAAATAGCTGAAAAATTTGGTGATGAAATAGTGATGAATAAGCAGATAAACAGA
GCAATGCTTAGAGCTATTATCACTGAATCTAAAGAAGCTAAAAAAATGGTTAGAAGACTATCTACATCCAG
TAATCAATAAAGAGATAAAAAAACAAGTAAAAGAGTCTGACACTGTGATGACTATTGTCGATATCCCACT
GCTAGGACCATACAACTTCCGTCACTATGACTATCTAAAGAAAGTAATAGTTATCAAAGCAGATCTAGAA
ACTAGAATTAGAAGATTAATGGAACGTGACGGCAAAAACAGACAACAAGCTGTTGCTTTTATCAACCTAC
AAATATCTGATAAAGAAAGAGAAAAAATAGCTGATTTTGTAATAGACAATACTGAACTAACTAATCAGGA
ATTAGAAAGTAAATTAATCACAACAATTAATGAAATAACCAACTTGACTAACTAA

>lcl|NC_006570.2_cds_YP_170423.1_1302 [gene=trpS] [protein=tryptophanyl-tRNA synthetase] [protein_id=YP_170423.1] [location=1541380..1542384]
ATGTCACAAAAAATAATTTTAACTGGTGTCACTCCATCAGGGACGCCACATTTAGGTAACTATATCGGTG
CTATTAAACCAGCAATCGAAATGATTAAAAATGATCAATATAAATGTATGTACTTTATTGCTGATCAACA
TTCGTTAATCAAACTTTGTGATAAAAAATTACGCCAGCAATACATTTATGAAATAGCTGCATCTTGGTTA
GCACTTGGACTTGACCCTGACAAAGCTTATTTCTATCGTCAGTCAGATATTCCTGAGATAATGGAATTAA
CGTGGATTATTAGTACAACTACTGCTAAAGGATTACTTAACCGAGCTCACGCTTACAAAGCCTTAGTTGA
TCAGAATCTTCAAGAGGAGAATGCTGATCCTGATAAAGGTATAACTATGGGACTATTTAACTACCCAGTA
CTTATGGCTGCAGATATTCTTATATTCGATGCGGACTTAGTTCCTGTTGGTAAGGATCAAATTCAACATA
TAGAAATAGCTCGAGATATCGCTAACCGTTTCAACCATATTTACCAAAAACCAGTTCTCAAGGCTCCTCA
AGCTCTGACAAGTGAAGATAGTCAAACTATACTTGGTTTAGATGGTCGCAAGATGTCAAAAAGCTACGAT
AATACTATAGCCATTTTCTCAACGGAGAAAAAACTTAGAAAACAAGTAATGAAAATTATTACAAATTCTC
AAATGCCTGAAGAGAAAAAAGATCCAAATAACTGTACTATTTTCGCCATATACAAAAGTATTGCAAGCCA

AACAGAGATAGCAGCCTTAGAGGAAAAATACTTAGCAGGTGGACTTGGTTGGGGAGATGCCAAACAAATA
CTTTTTGAAAAAATAAACGAATATCTCAGAGATGCTAGAGAAAAATATGACTATTATATAAATAATCCTA
AAATTGTCGATGATATTCTTAACCAAGGAGCTGCGAAAGTACGTCCTTTGGCAAAAGATAAACTCAAAGA
AGTTAAAGACATTATAGGAATGTAA

>lcl|NC_006570.2_cds_YP_170424.1_1303 [gene=FTT_1489] [protein=hypothetical protein] [protein_id=YP_170424.1] [location=1542386..1543111]
ATGCAGAAGTTAGATAATATAATTATTGCTAACTACAGCATTCATTCTTTAGCGCTTATATGCTGGGCAA
AGCAAAATTTATCGGGTAACTTCTGTATCTTATCAGTAGATACAGGTTTCGCTGCGGCTAATTGGAATAG
TTATTTAGAAGCGGTTTTTGACTGGCTAACAAAAGAAAATATCAGCTATTTTCATTTAAAATCAGAAAAT
ACCTTCCAAGAGCTAGTTTTAGCACGTAAGCAATTCCCATCGCAACAATTTAGCTGGTGTGCGAGTTTTC
TTAAAGGTATAACTCTACTTAATAAAATCGATGAACTTGATGCTAACGGTAATGCCACTATCCTACTTAG
TCATCGCAGCGATATGTCCAGTGTCTCACGGCTTTTAAAGAATCTAGATGAAGAAGAAAGATATGATTAT
AGACCTCTAGCATATCCTTTACTAAATCATAGCTATGATGATATTCTTGATTTAACTAAAGAGTTACCAT
TCAAAGCTGCTAAATACAGTCTTGAATGTCAACCATGTATACATTTGACTCAACAACAGTTTAAGATATT
AACTAAAGAAGATATTGAAAAAGTAACTGACTTAGAGGTTAAAACAAATAGCTTTATGTTTGGAGATATG
CCTTTTGATAAGTTAGATGATGGTTTCTTTACACAAAAAGATTTATTAGAAGAATTATCAAAGGCATGCA
GCTGGGAGTATAGCTGTGGCTTATAA

>lcl|NC_006570.2_cds_YP_170433.1_1310 [gene=accA] [protein=acetyl-CoA carboxylase carboxyltransferase subunit alpha] [protein_id=YP_170433.1] [location=complement(1551112..1552059)]
ATGAATTATTTAGACTTTGAGTCAAAAATAAAAGAAATCGAAGATAAAATTACATCTTTGTCACATGTGT
TTGAAGATGAAAAAACAGAGGTTGAGATTAAAAAGCTAAGTAAAAAAAGACTTGAGCTTATGGAGTCCAC
TTATTCTAAGCTTACAGACTGGCAAGTAGTACAATTATCACGTCATCCTGATAGACCATATTTCAAAGAT
TTATTACCATTAATTTTTACTGATTTTCAAGAACTACATGGTGATAGAACTTTTGGTGATGACTTAGCTG
TTATCGGTGGTTTGGCAAAATTAAACAATAAGCCTGTCATGGTGATTGGTCAAGAAAAAGGTCGTGATAC
TAAGAGTAAAATAAAACATAACTTTGGTATGATGCATCCAGAAGGGTATCGTAAAGCTTTAAGACTTATG
AAGTTAGCAGAGAAGTTTAATATGCCAGTTGTGACTTTTATTGATACTCCTGGTGCATACCCTGGTATCA
AGGCAGAAGAACGTGGTCAAAGTGAAGCTATTGCAAGAAATTTATTAGAGATGAGTGCACTAAAAGTTCC
TGTTGTATGCATTGTGATTGGTGAGGGCTGTTCTGGTGGTGCCTTAGGAATAGGTGTTGGTGATAGATTA
CTTATGCTACAGTATAGTTATTTTGCGACTATTTCACCTGAGGGTTGTGCTTCAATTCTACACAAAACTG
CTGAGAAAGCATCTGAAGTTACACAAATGATGAATATTACTTCAGGTCGTCTTAAAGAGTTAAAGATCGT
TGATGAAGTGATTCCAGAACCTTTAGGTGGAGCTCACCGTGATTACGAAACTACAGCTACTAATATCAGA
AAAGCTGTGGCTGCCGAGCTAAAAATACTTTCTGAGATGACAGTCGAACAAAGAAATTCTAGAAGGTATG
ATAAGCTAATGTCTTTTGGAAGATTTAAAGAAGCTTAG

>lcl|NC_006570.2_cds_YP_170456.1_1333 [gene=idh] [protein=isocitrate dehydrogenase] [protein_id=YP_170456.1] [location=complement(1582649..1584892)]
GTGAAATTCTATCTACAGGAGATTTTTATGCATAAAATATTTTATACATTTACAGATGAAGCACCAGCAT
TGGCAACAGGATCATTTTTACCAATAGTTGAGAGCTTTACTAAAGTTGCAGATATAGAGTTAGAAACGAA
AGATATTTCTCTATCAGCGCGGATATTGGCTAACTTTAATGATTACCTTACTGCAGAGCAAAAGTGTTCT
GATGATTTAGCGATACTTGGAGAGCTTGCAAAAACTCCAGATGCTAATATTATAAAACTTCCAAATATTA
GTGCATCAATTCCTCAGCTTACAGCAGCTATTAAAGAACTTCAGTCAAAGGGTTATAAAATACCTGATTA
TCCATATGAGCCAAAAGATGATAAAGAGCAAGAAATTAAAGCACGCTATGCAAAAGTTTTAGGGAGTGCC
GTAAACCCTGTTTTAAGAGAAGGTAACTCTGATCGTAGAGTTGCTGATGCTGTTAAGAAGTACGCCGAGA
AACATCCACATTCTATGGGTGAATGGACAAAAGACTCTAAATCTCATGTGGCAAGTATGAGTGCTGATGA
CTTTTATGCTAATGAGAAATCTTATATTGTACCAAAAGCAACTAAGGTTAAAATAGTCCATATAGATACA
AAAGGCACTCAAACTGTGCTTAAAGATAATCTTGCGCTAGAAGAAAAAGAAATTATTGATGCTACTAAGA
TATCTATAAAAGCTTTAAGAGAATTCTACAAAAATGAAATAGCTAAGGCTAAAAAAGAAGGTACGCTATT
ATCTCTCCACCTAAAAGCTACGATGATGAAAGTTTCTGATCCAATATTGTTTGGTCATGCGGTAGAGATC
TTTTTCGAAGATATTTTCAAAAAGTATGCTAAAGAGTTTAAAGAGTTAGGTGTAAATCCTCGTAATGGTT
GGGGTGATGCAGTAGAGAAAATCAAGCAACTTCCACAAGAACTTCAAGATAAAATTAATGCCGATATTGA
AAAAGTATTTGCAAAGCAGCCTGATATTGCGATGGTTAATTCTAACAAGGGTATTACAAACCTAAATGTT
CCGAGTGATGTAATTATTGATGCTTCTATGCCTGCGGCTATTCGTTCATCTGGTAAGATGTGGAACAAAG
ATGGCAAACTTCAAGATATGAAAGCGATGATTCCTGATAGATGTTATGCTGGAGTGTATGCGGCAACTAT
CGATTTCTGTAAAGAAAATGGCGCTTTTGATGTGGCTACTATGGGTGATGTTTCTAACGTTGGTCTGATG
GCGAAAAAAGCAGAAGAGTATGGTTCTCATGATAAGACATTTGAAATCCAAGCTGATGGTAAAGTTGAAG
TTATTGATGCTGAAGGTAATGTAATATTTGAGCATCAAGTCGAAAAAGGCGATATTTGGCGTGCTTGTCA
AACTAAAGATATCGCAGTTAAAGACTGGGTTAAACTAGTTGTTAATAGAGCTAAAATTACTCAAAATCCT
GCGATTTTTTGGTTGGATTCAAAAAGAGCTCATGATAGAAACCTAATTGCTAAAGTGAATGAGTATTTAA
CTCATCATGATACTACTGGCTTAGATATTCAAATATTATCTCCAGTAGAAGCTACGAAATACTCTCTAAA
AAGAATGAAAGAGGGTAAAAATACAATTTCTGTGACAGGTAACGTTTTAAGAGATTACTTGACAGATTTA
TTTCCTATTTTAGAGCTTGGTACTTCTGCTAAGATGCTTTCTATAGTTCCGCTATTAGCTGGAGGTGGAT

TATTTGAAACTGGGGCTGGTGGTTCTGCGCCTAAACACGTTGAGCAACTTATTGAGGAAAATCACCTAAG
ATGGGATTCTTTAGGTGAGTTTTTGGCTTTAGGAGCATCATTAGAAGACTTGGCTATCAAAACTAGAGAT
GCTAAAATCAAGGTTTTAGCTGAGACATTATCACAGGCAAACAAAGATTTCTTGGATAATGATAAATCGC
CGCGTCGTAAAGTTGGTGAGCTTGATACTCGAGGAAGTCATTTCTATTTGGCTTACTATTGGGTTAAGGC
TCTAGCTGAGCAAACAGGTAATGCTGAATTAAAAGCTAAATTTGAGCCAATTTATATTGAACTAAAAGCT
AATAAGGATAAAATTGTCAAAGAGCTTAATGATGCTCAAGGCAAAAAAGTTGATGTTGGTGGCTACTATC
ATATGGATAGAGTAAAATTAGATGCAGTGATGAGGCCGAGTAAAACTTTTAATACAATTTTGGCGCAAAT
ATAA

>lcl|NC_006570.2_cds_YP_170480.1_1356 [gene=lepB] [protein=signal peptidase I] [protein_id=YP_170480.1] [location=complement(1617874..1618737)]
ATGGAAATCTTAAACTATATTTTAAACTTGGGCTTTACTTTTTGGCTTTTATTCTTAACCATTGCCAGTG
GTTTAATTTATATTATTGATTTTGTGTTCTTCCAAAAATCAAGATTAGCAGCATATACAGATGAATTAAA
AGGTCTTTCTAAGAAGCAAAAACGTCAGTTCTATAAAGATAGAGGATTAAAAGCACCTTTTATTGCTGAT
CAGGCGAGATCTTTATTTAGTGTATTTTTTGTAGTTTTTCTACTTAGAACCTTCTTGATTGGTAATTTTT
TAATTCCAACTGCATCAATGACACCAACACTTCCAGTTGGTGATTTTATTTTTGTCAATAAAACTGCTTA
TGGTATCAGAGCACCATTTACCAATGAGACTTTAATAAAAGTTGGTGAACCCAAAAGAGGTGATATTGTA
GTATTTCATTTTCCAGTTAATCCTAATGTTGATTTTGTAAAACGAGTGATCGGTTTGCCTGGCGATGTAA
TTTCGTATAAAGACAAAATGTTGACAATAAATGGTAAAAAACTTGAATATACTAATTGTAATCGTGATGC
AATGAACTATTATAATCAGTCTTTAGCTGCTGGTAGTGGCGATACAGTATGTACGGAAAACCTTGATGGA
GTTAAACATGAGGTTGATTGGATAGAGTCTATAAAGGGAACTGATTTTGAAAACCTTAGAGTCCCAGCAG
GTCAATACTTTGTTATGGGAGATAATCGTGATAATAGTGAAGATAGTCGCTATTGGGGTTTTGTACCTGA
CAAAGATCTAGTTGGTAAAGCAAAAGTTGTTTGGATGAGCTGGGATAAGATAGATAAAAAGGTTCGCTGG
GATGAAATTGGTAAGGTCTTTTAA

>lcl|NC_006570.2_cds_YP_170481.1_1357 [gene=FTT_1557c] [protein=two-component response regulator] [protein_id=YP_170481.1] [location=complement(1618742..1619428)]
ATGAGAATATTGTTGGCTGAAGATGATCTTCATTTGGGTGAAGGCTTATTAGAAGCTTTGCAAAAAGAGG
GTTTGATTGTAAATCTAGTTTCAGATGGTGAGGCAGCGCAAACTTTTATAGAATCTGGATTGTATGATAT
AGTCGTCTTAGATATTGGTATGCCAATAAAAACTGGTTTAGAAGTTCTAAGAAATATTAGAAATAGAGGT
ATTAAAGTACCAATAATACTGTTGACTGCTAGAGATGGTTTAGAAGATAGAATTAAAGGTCTTGATCTTG
GTGCTGATGATTATCTAACTAAGCCATTTGAGTTAAAAGAGCTAGTAGCTAGAATTAAAGCTATTTCTCG
TCGTATTGATACTAGATCTGGTAAAAGTGTTAATGAAGAAATTAGATTTGGAGATTATAGTTTTAATCCA
AGCTCTGAAACGGTAACCAAAGATGGAGTTATTATTCCTGTTTCAAAAAAAGAGTTAGCGCTTTTAGCTA
TATTGGTACAAAATGCTGGTAGGGTAGTACCAAAGACACAGCTTTTAGAAGAAGTATATTCTACTGATAA
GGAAATGGATACAAACACCCTAGAAGTACATATGCATAATTTAAGAAAGAAAATTAACATTCCTAATTTT
ATACAGACTATTAGAGGTGTTGGTTACTTTGTACAAAAGGATAAAGTAATTAAGTAA

>lcl|NC_006570.2_cds_YP_170484.1_1360 [gene=kdtA] [protein=3-deoxy-D-manno-octulosonic acid transferase] [protein_id=YP_170484.1] [location=1622819..1624114]
GTGGAACATTTAAAAAAATTCTTTTATGTTTTTCTAGCTCATATATATTCGAGCATTTTTATAACATTAA
TACCTATTTTATATCTGAAAAAATTTAAAAGAAGCTTTAAAAACATTAACTACAGAAAAAGATGGGCAGA
GAGATTCGCCCAGATACCAATACGCCTTAACAGCAGTATATGGATACATTCAGTTTCTGTAGGTGAAGCT
GTCTCTGCTGAACCACTAGTAAAAGAATTACTAAAGAATTTTCCTAATGAGAATTTTGTTATAACTACAA
CAACACCAACAGGTAGCGATGTTGTGAATAGTCTTTATAGTAACTATCCCAATGTACACCATATGTATAT
CCCTTATGATGTAATTCCTTTTATTAATAGTTTTTTTGCTAAGACTAATCCAAAGATTTTTATAATCGTT
GAAACAGAAATTTGGCCAAATATTCTAAATAAGTGTTTTGCTGAAAAGGTTCCAGTAGTAATAACAAATG
CGCGACTATCAAAAAAAATCGATGCGTAATTACACTAAAATCCCTTTTGCTAAAGAATTTTTATTTAAGAA
TATCTCTCATATTAACGCTCAGACAGAGAAAGATGCAAAAAGATTTTATTCTCTAGCTGTAGATAAAAAC
AACATTTCTGTGACAGGAAACTTAAAATACAATCTTATCACTCCAGAAAACCTAGAAAATAAGATGTATA
GCTTGAAAGACAGCCTAAAAGGTAGACCAGTATGGATTGCAGGGAGTACTCATCAAGGTGAGGAAGAAAT
AATTCTTGAAGCACATAAACAAATCCTAAAAATACATCCAGATTGTTTATTAATACTAGTTCCTAGGCAC
AAAGAACGCTTTCAGAAAGTAGAAAAGCTAATTGTTTATAATTCTTTGAAGTATCAAAAAAGAAGCTCTT
TTGAATGTCAAATCTTTAGTGATACTCAAGTATATTTAGGCGATACAATGGGCGAACTACTTCATCTTTA
TTATATTGCAGATATAACTTTTGTTGGTGGAAGTTTAATAGACAATGGTGGACATAATTTACTTGAACCA
GCCGCATTAGCAAAGCCAATTTTAAGTGGTCCAAGCCTTTTTAATTTCAGCCAAATATCCAAAGAACTAA
TTCGTAATAAAGCTCTAATTCGCATAAGAAACCAACAAGAAATTGCAAATAATATCCTCAAAATACTAGA
AGATAAACAACTATTACAACAGATGTCATCGGGCGCTCTTAAGACTTTTAAAAGCCATAGTGATGTACTC
GAAAAACAGTATAACAATATCGTAAAATTCTTATGA

>lcl|NC_006570.2_cds_YP_170490.1_1365 [gene=lpxB] [protein=lipid-A-disaccharide synthase] [protein_id=YP_170490.1] [location=complement(1629617..1630759)]
ATGAGAATAGGTATTGTAGCTGGAGAGCTTTCGGGCGATCAGTTAGGTGGCACACTAGTTGAGGCTTTAA

AGCAAAAATACCCTAATGCAATTATTGAAGGTATTGGTGGACCAAAAATGGCAGCCGCTGGTTTTAAAAG
CCTTTATCCGATGGATGCATTGTCGTTAATCGGCTTCTTAGAAATTATTTCAAAAGGTTTGCGCATATTA
AGTATCCGCCGTAAAATCATCAACTATTTTAAGCAAAATAAACCTGATATATTTATAGGCATAGATGCTC
CTGATTTTAATCTTACAGTTGAAAAAGAATTAAGATCTGCGGGTATCAAAACTATACATTATGTAAGTCC
TAAAATATGGGTTTGGCGTGAATACCGTATAAAAAAAATACGTAAAGCAACTGATAAAATATTAGCAATT
TTACCTTTTGAAACTGAATACTATAAAAATAGACATAAATTTGAAGCTATATATGTTGGTCATCCATTAG
CGAAGAATATTCCAATACATATTGACCGAGCTAAATATAGAGATAAACTTGGATTAAAAGGTAGTTCTTT
ACCAATATTATCAGTTTTACCAGGAAGTCGTACGACAGAAGTTTCAAGATTGTTGCCACTATTTTTGTTA
GCATTACAAAAATTGGTAGATGCTGGATATAAATTTAAAGCGATTATGCCCTTAGCTAAGCCATCATTAA
AACCATTATTTGCTAAATATAAAGAACAAATTGATAGTTTAGGTATCGAAGTTTTTGAAACGAATTCACA
TGATGTTTTAAAAGCATCTGATTTAAGTTTATTGGCATCAGGAACAGCTACATTGGAGGCTATGTTATGC
AAATTACCTATGGTTGTAGGCTATAAACTATCATGGCTATCGGCTTTAATTGGTAGGATGCTTATTGGTA
ATCATAGTTATTGGGCTTTCCCTAATATTTTGCATAAAAATGAAATTATCAAAGAATTAATACAAGAAGA
CTGTACAGTAGATAATTTATTTAGCGAGTTAAAAAGGTTGTTTGATGATAAGCGGCGTAATGATTATATA
GTAGAAGAATTTGAAAAAATTCACAAAGAAATGGTTATCGATACTGAGAGTAAGATCATTCAAGTATTAG
ATACTATGATAGAAAAATCTTAA

>lcl|NC_006570.2_cds_YP_170491.1_1366 [gene=lpxA] [protein=acyl-[acyl-carrier-protein]--UDP-N-acetylglucosamine O-acyltransferase] [protein_id=YP_170491.1] [location=complement(1630759..1631538)]
GTGATACATAGTTTGGCAGTAGTACATGAGAGCGCCAAAATAGCCGATAGTGCTATAATAGGGCCATTCT
GTGTTATTGGGAAGAATGTTGTTATTGGTGAAAATACTGAGTTAAAAAGCCATGTAACAATTGGTGATAA
TGCAGTTATAGGCAAAAATAATCGCATTTTCCAATATGCTTCAATTGGTGATGATCCAATTGATTATACA
TATAAAAAAGGTGACTTTTCTCAAGTTGTAATTGGGGATAATAATATTATCAGAGAGTGTGCTACTATTC
ATGGTGGAACTGCAAAAGAAATTGGGGTAACATCTGTTGGTAATAACAACATTATTATGTGTTATGTACA
TATTGGGCATGATTGCAAAATGGGCAGTTACATAAACTTAGTAAATGGTGTTGGTCTAGCTGGACATGTG
CATATTGATGATTATGCAATATTAAGTTCTAATGTTGGTGTTCATCAATTTTGTAGAGTTGGTAAACATG
CTTTTATTGCTCATGCAGCACTAGTTGGTAAGGATGTACCCCCGTATTTAATGGTTACTGCTGTGAATGC
TGGCTCAACTCCTTGTGGTATAAATACAGAAGGTTTAAAGCGTCGTGGATTTACGCCTGAAGAGATGAAA
AAAATCAAAGAAGTCTACAAGGTGTTGTATCGCAAAGGTTTGATGATGAAAGAAGCTTTTGAGATAATCA
AAGCGATGGCAAAAGAAGATAAAGTTCTAGAGCCTTTTGTTGATGTGATTGGTACTTCGCGAAGAGGTAT
ACTAAGATAA

>lcl|NC_006570.2_cds_YP_170492.1_1367 [gene=fabZ] [protein=(3R)-hydroxymyristoyl-ACP dehydratase] [protein_id=YP_170492.1] [location=complement(1631541..1632032)]
ATGAGCCAGTTTAATCAAAATAATAAACAGATAGATGTAATGGGGATTAGAAAAATCTTACCTCACAGGT
ATCCTTTTGCACTTTTGGATAAGATCGTTGATTGGAGTGTTGAAGATAGAACGATTGTTGCACAAAAAAA
TGTCACTATAAATGAAGATTTTTTCAATGGACATTTTCCTGATTTCCCAGTTATGCCTGGTGTACTGATA
GTTGAAGCGATGGCTCAAGCAACGGCAATACTTGGTGAATTAATGGCAGAGACATTGTTCGCACATGTTG
TTGAGAAAGCTGGTGGTGGTAGAAGAACATTCATGCTAGCTGGTATTGATAAGGTAAGGGTCAAAAGACC
TGTAGTACCAGGAGATGTGTTAGTGATTGAGTCGCGTATGGTAAAACAAAAAAATATTATCTGTACAGCA
GAGTCAGTCGCAAAGGTTGATGGACAAATTGTTTGTTCAGCTGAATTAATGGCAGCATATAAGGACTACT
AA

>lcl|NC_006570.2_cds_YP_170493.1_1368 [gene=lpxD] [protein=UDP-3-O-[3-hydroxymyristoyl] glucosamine N-acyltransferase] [protein_id=YP_170493.1] [location=complement(1632047..1633060)]
ATGTATAGTTTAGATTTTCTAGCGTCAAAGCTTGATGGTGAGGTTAAGGGCGATAAGAATGTCGAGATAA
AAAAAATAGCTACACTATCACAAGCTGGTGAAGGTGATATTTCTTTTTGTACTAACCCTAAATATTTAAA
AGCATTGTCTGAAACAAAAGCTTCTGCAGTTTTAATAACAGAAGAGGTACTAGAGTTTTGTAATACAAAC
GCGGTGGTACTATCAAATCCTTATATGGCTTTAGCAAAGGTTATGGAGCTATTTGATAAATCGCCGCGTC
CTGATGGTAAAATTCATAGTAAAGCTGTGATAGCCGCAAGTGCTATAATTGGTGAAAATGTCACTATAGG
CGCTAATGCGGTAGTTGGTGAAAATGTAGTAATTGGCGACAATGTTTATATAGGTGCCTGTGCAACTATT
GATAATGGTACCAAGATAGGTAATGACACGCTGATAAAGAGCAATGTATCTATAGCTCACGATGTTGTGA
TTGGTACTGGGTGTATTATTCATCAAAATGCAGTAATTGGCTGTGATGGTTTTGGTAATGCAAGAGATGA
GGATGGTAGTTGGACAAAAATTCCTCAGCTAGGAAGAGTAATTATCGAAGATGATGTTGAGATTGGTTCT
GGTACAACTGTTGATAGAGGAGCTATTGATGATACAATAATTAAAAAGGGCGCACGTATTGATAATTTAG
TACAGATAGCTCATAATGTAGTTATCGGTAGAAACACCGCTTTAGCCGGTGTTACAGCTGTTGCAGGTAG
TACAACGATTGGTGATAACTGCCTTATTGGAGGTCAGTCAGCAATAACTGGCCATATTAGTATTTGTGAT
AATACTATTATAGGTGGTGCATCTAATATTGGTAAGTCAATCACTAAGCCAGGAATGTACTATGCTGCGT
TTGAAGCTAAACCTAGGATTCAATGGGGTAGGTTCGTAGCTAAATTAGCTAAGATTGATACTCTAATTAC
AAAAGTAAAGCAACTAGAAGAAAAAATTAAATAA

>lcl|NC_006570.2_cds_YP_170494.1_1369 [gene=ompH] [protein=outer membrane protein OmpH] [protein_id=YP_170494.1] [location=complement(1633079..1633582)]
ATGAAAAAAATTGCTTTATCAATATGCGTTTTAGCAAGTGCATTTACCGCAGCGTATGCAGATACTAAAA
TAGCTGTAGTTAATCCAGTTGAGATCTTCAATGATTCTGATCTAGGATCTGTAAGTGTTAAAAAACTTGA
AAATGATCTTAAACCAGATGCTACTAAGCTTAAGCAAGAACAAGATAATATCATGCAACAGATAAAAACT
TTGCAAGATAATTCTGCAACAATGACTAAGAGTGAATTAGATAAGAAACAGCAACAGATTCAACAAGAGC
AACAGAATTTTGCTGAAAAAGCACGAATCTTACAACAAAAAGAATATACAGCAAAAGATAAACTATCAAA
GAAATTCCAAGCTTCATTTGACAAAGCTGTTCAAACCATAGCTAAGCAAAAAAATTATAATGTTGTGCTT
ACAACACAAGCTTTAGCGTATGTTAATAATGTTGATGATATATCTAGTCAAGTTGTTGAGTTGATGAATA
AAGACTCTGAATAA

>lcl|NC_006570.2_cds_YP_170495.1_1370 [gene=FTT_1573c] [protein=hypothetical protein] [protein_id=YP_170495.1] [location=complement(1633718..1636096)]
GTGTTTTTTTTAAGAAAGAAGTTTGTGTTAGCCTCTCTTCTTGTGGGTATTGCATTTAATGGTTGGGCTG
ATAATGATAGCTTTATATTAGATAATGTATCTATCAATGGCTTAGAGGGGTTACAAAGTGATGTAGTTAA
GAGCCGCATTGGCTATAAAAAAGGTAGCTATATCACCCCAGAAGATACAAACCAGATTATTAATAACCTT
TATGGTACTGGATTTTTCAATAGTGTCGATTTATACCGTAAAGGGAGTAATCTTTTTATTAACGTCAAAG
AAAGACCGATTATAGCTGGATTTAGTTTTAGTGGTAATAAAAAGCTTAGTAAGGATAATCTTGAGAAAGT
CTTTACTGATGCAGGTATATATGTAGGTAATGTTTATAATCCTAATACGATGTTTTTGCTTAAGCAGTCT
TTGCTTAATCAGTACTCTATGATGGGCTTATATGGTGCAAAAATAGAGGAAAACATTCGAAAGCTACCAA
ATAATAGAATTGATATAAATGTAAACTTTCAAGAGGGTAAACCAGCAATTATTGATTCAATAAATTTTGT
CGGTAATAAAAGTTTTGCTGATAGTGATTTAGATAGTAGTGTTGCATTTGAAGTACCGTCCATATGGAAT
TTATGGGGATTTTTGGCACCATTTGATAGTTATTCTCCAGATGGAATGAATCAGTCTGTCCAAGGCTTAA
CTAATTATTATTTAGACAGAGGTTATTTAGATTTTAAGGTTACCTCAAAGCAAGCATCAATGTCTAAGGA
TCGCGAGCATTCGTATATTACTTTTGATGTTGCAGAAGGTCAGATTTATAAGGTTGGAAGTGTTTCCTTA
ACAGGTAAGTTTATTCTACCAAAATCAGAGTTAGAGTCTTTGGTGAAGATAAAACAGGGAGAGGTATTTT
CAAAATCTAAATTAGTTCAGACAGTTGAGGGGATAAAAACTCTACTAGGTAGTAAAGGATATGCCTTTGC
TACGGTTAATCCTATTCCTACAGTTGATAAGGATAATCATATAGTTTCTTTTAAGATTGTTGTTGATGCA
GGAAAAAAGGTTTATGTAAATAGAATAAATTTCTTTGGTAATAATGTCACTAATGATTACGTATTTCGTC
GTCAATTACAATACTATGAACAGAGTCAATATAACAAAGAGGCAATAGATAAATCTCAAAGAAGATTAGA
GCAACTACCATATGTTGGTGCAGCTGATATGGAACTTGTTCCTGTTGCAGGGTCTGATGATTTGGTTGAT
GTAAATTATAATATTAAAGAAAGGAATGCTAACTCGATAAGTGGAAGTTTAGGCTTCTCGGATTTATATG
GATTTATGATAGGCGGTAGGCTGAATATGCCTAACGTTTTTGGTACTGGAAATACTTTTAATCTTAATGC
GCAACTTTCAATTCCTTTCCAGCAGTTAGATATAAGTTATATTGATCAGTTTTTTACTACATCAGGGGTT
AGCCAAAGTATTTCTGCATATATAAATAGATCAAACTTTGCTAAAACAAATGCTGTTGCTGCGTATCAAT
TAGATACGATTGGTGCAAGATTGATGTATGGAGTGCCTATATCAACATTTAGTAATGTTTCAGGAGGTAT
AACTTTTGCTAATAATACGGTTAAGCAGTCAGATGGATACCAGTCTTCAATTGTACAGTGGTTTATCCAG
CAGCAGGGTGGTAGAAACAATTTTAACGAGCCGGCACTGACAGCAGGTTGGAGTTATGATAACTCAAATA
AATATATTTTTGCTACAGATGGTGGTTCATTTAATCTAAATGGTTCAGTTAACATTCCAGTTATCAGTAA
TATTAATGCTTATAAGATAGAGGTTGGAGGAACTTATAATATAGCAGTGCCAAATACTGACATGTCAGCA
TTAACTATTAGAGCTGGAGTTCAGTATGGTGGTGGTTATGGTAAGACAAAACAGCTGCCTTTTTACGAGA
ACTTCTACGGAGGTGGTTGGGGAAGTGTGCGTGGTTTCTTACAAGGTTCTCTTGGTCCACGTGATATTAA
TCTGGTAAATGGTGAGGTAGGTAACTCAATTGGGGGTAACCTTAATATTTATAATAACTATGATGTATTA
TTCCCTGTACCTTTTATCAAGGATAGTTCTAAGATGAGAATAGGTGCTTTTTTAGATATAGGTAATACGT
ATACAACATATAATCTAAATGGAGTAGTAGCACCTCAACCTAAGCAAGAAACTACTCCTTCATTTTCTAA
TCTAAAGTATTCGGTTGGCGTTGAGTTTAGATGGGCTTCACCAATGGGCCCACTAGCTGTATCATTTGCT
CAGCCATTTAATGTACAAAAAGGTGATATAACTCAAGTATTTCAATTCTCATTAGGACAAAATTTCTAA

>lcl|NC_006570.2_cds_YP_170496.1_1371 [gene=dxr] [protein=1-deoxy-D-xylulose 5-phosphate reductoisomerase] [protein_id=YP_170496.1] [location=complement(1636102..1637259)]
ATGTTTAAAAAAACTAAGATTACTATATTAGGAGCTACAGGATCTATAGGTGATAGCACTTTAGCTGTTA
TTAGAGAGACTAATGATTTTGAAGTTTTTGCATTAACTGCATTTAGTAATGTTGAAAAGCTGGCAGAGCT
ATGTCAAGAGTTTAAGCCTAAATTTGCTGTAGTTCCAGACTTGTCAAAAAAACAAAAGTTACAATCATTA
GTTACTGATGTTGAGGTTTTGGTTGGTGAAAGTGGGCTTGAAAAAGTATCTAGCTTAGCTGAAATTGATA
TTGTCATGTCTGCTATAGTTGGTATAGCAGGGTTAAAACCAACCTTTGCAGCAGCTAAAGCTGGTAAGAA
AATTTTGCTTGCTAATAAAGAATCTTTGGTAACAGCAGGACACTTGCTGATAGATGAGGTTGTAAAGAAT
AATGCTCAACTTATTCCTGTGGATAGTGAACATAATGCTATATTTCAGTGTATTGATAATCATGATAAGA
AGTGTTTGCCAGAGATTGATAAGATAATTTTAACAGCATCTGGAGGTCCTTTTAGAGACAAACAACTACA
TGAGTTAACGGATGTAACACCCGAGCAAGCATGCAATCATCCTAATTGGCAAATGGGCAGAAAAATATCG
GTTGATTCATCAACTATGGTAAATAAAGCGTTAGAAGTTATCGAAGCGTATTGGCTTTTTTCTGTTTCAG
CTGATAAAATAGGCGTTCTGATTCATCCGCAGAGTGTAACTCATTCTATGGTCAGATATGTTGATGGTAG
CTATATAGCGCAGTTAGGTGTGCCTGATATGAAAACACCTATAGCCAACGCAATGTACTATCCAAAGAGA
GGATCAGTTAACGTTGAGAGCTTAGATTTTACAAAATATCAGCTAACCTTCAGAGAAGCTTGTTTTGAAA

GATTTGAAGCTTTGAAAATAGTTTTTAATAATTTACAAAATAAAAATTATGCTGCTAACATAGTTTTTAA
TGCTGCTAATGAGGAGCTTGTGGCTGCATTTTTAAACAAAAAAATTAAATATTTAGAGATAATAGAAGTA
AACAAAAAAGTAACAAAAGAGTTAAATTTTGAGAATCCAAAAAATATAGAAGAAGTTTTTGAAATAGATA
GAAAAACTCGTGAATATGTGGATTCTGTTTTGGGGTAA

>lcl|NC_006570.2_cds_YP_170497.1_1372 [gene=gyrA] [protein=DNA gyrase subunit A] [protein_id=YP_170497.1] [location=complement(1637265..1639871)]
ATGTCTATAATTACTAAAGAGTCATCATCTATAAATATCGAAAAAGAACTAAAACAATCGTATCTTGATT
ATGCTATGAGTGTAATTGTTGGTCGTGCTTTGCCAGATGTGCGTGATGGTCTTAAGCCTGTCCATCGTCG
TGTACTTTTTGCAATGAATGAACTATCAAATTATTACAATAGACCATATAAAAAGTCTGCAAGGGTTGTC
GGTGATGTAATCGGTAAATATCACCCTCATGGAGATACAGCTGTTTACGATACTATTGTCAGAATGGCAC
AACCTTTCTCATTGCGCTATACGCTAGTAGATGGACAAGGTAACTTTGGTTCTGTTGATGGTGATTCTCC
TGCAGCAATGCGTTATACCGAAATTAGAATGGAAAAACTAACGCATGAGCTCTTGATCGATATAGATAAA
GAAACTGTAGACTTTTCACCTAACTATGATAATACTGAGTTAGTTCCAGACGTGTTGCCAACTAGAGTTC
CAAACCTTTTAGTAAATGGGTCATCAGGTATTGCTGTTGGTATGGCAACTAATATTCCGCCGCATAATAT
GACTGAGGTTATCAATGGTACTATAGCTCTTATTGATAATCCTAACTTAACTATCGAAGAACTTATTGAG
TATATTCTAGCTCCAGATTTTCCTACGGGGTCATATATTAATGGTACAGATGGTATTTTAGAGGCTTATA
AAACTGGTCGCGGCCGTGTGATTATGCGTGCTAAAGCTGATATTCATGAAGATGAAGCATCTGGTAAAGC
ACAGATAATAGTCACAGAGATACCTTACCAAGTAAACAAAGCTAAGCTTGTAGAGAAGATTGCTGAGCTT
GTTAAAGATAAGAAAGTAGCAGGTATATCTGAGTTAAGAGATGAGTCTGACAAAGATGGTATTCGAGTTG
TTATTGATTTAAAAAGAGATGAGTCACCTGAAGTTGTGTTAAATACTTTATATGCTCAAACACAATTACA
ATGTAGTTTTGGGATTAATATGGTTGCTCTTAGTGACAATAGACCTAAGTTACTGAATCTTAAAGAAATT
CTAGAGCAGTTTATTAAGCATAGAAAGGAAGTAGTAACAAGAAGAACAATTTTTGAACTAAGAAAGTCAA
AAGAGCGAGCGCATATTTTAGAAGGCTTACTACTGTCATTGGCTAATATTGATGAGATGATTAAACTAAT
TAAGGCATCTCCATCACCAGCTGATGCAAAAGAGTCAATGCTTGCTAGATCATGGAATGGTTCTATGGTT
AAGAGTATGCTAGAGGGTGTAGATGTTAAAATGTATCGTAAAGAAACTCTAGCATCTCATTATGGCATTC
AAGTTGATTCTTCATATAATCTAACTGAAGAGCAAGCGGATGCAATTCTTGCTTTGAGATTACATCGATT
GACAGGTTTGGAACAAGATAAGATTGTTAATGAATTTAAAGAGCTTATCGATAGAGTTAAATATCTAATT
AGTATTTTGAGTGATATTAAAGAGCTTATTCGTGTCGTTAAAGATGAACTTGTTGAGATTCGAGATAACT
ATGGTGATCAAAGAAAATCAGAAATTATAGAGTCAAGATTAGATCTAACTAGAGAAGATCTTATCGCAGA
AGAAGATATGGTTGTGACTCTATCTATGGATGGCTATGTTAAAACTCAACCGTTAAGTATGTATAATGCG
CAAAAACGTGGTGGCGTTGGTAAATCTGCAACTAAGACTAAAGAAGAAGATAGTATATTTAAGTTAATGC
TTGCTTCAACTCATGATACTATGTTGTGTTTTTCAAGCTTAGGTAGAGTGTATTGGTCAAAAGTCTATGA
CTTCCCAGTTGCAAGTAGAATTTCTAAAGGTAGACCAATTAACAATATTTTACCATTAGAGAAAGATGAA
AGAATAACGGCAATGATGCCAATTTCTCAATTTAATGAGGGCTGGTATGTGTTTATGGCAACTAAACTTG
GTAGGGTCAAGAAAGTTGACTTATCAGAATTTGCTAGACCACGTTCTAAGGGTAAGATAGCAATTGGCTT
AAATGATGGTGATGAGTTATCCTATGTTGCTCTTACAGATGGTAATAAACAAATTATGATGTTTTCTGAC
GCCGGTAAAGCAATTCGTTTTGATGAGTCTGATGTCAGAGCGATGGGTCGTTCAGCAGCAGGTGTTACAG
GTATGCGTTTACATCATAATCAAAAGATAGTCTCTGTAATTGTTACTAATCCAGATGAGGGGATTGTCTT
AGCTGCAACAGAGAATGGATATGGTAAGCGTACCGCAGTTTCAGAATATCGTAAAACTAAACGAGCAAGT
CAAGGTGTAATAGCTATATCAACATCTGAAAGAAATGGTAAAGTTGTAGTAGCTGTCCTAGTTGAAAATG
ATGAAGATATTGTAATGATAACTGATAATGGCACTTTGGTAAGAACATCTTCTGATGAGGTTAGAGAATG
TGGACGCTCTGCCCAGGGGGTTAGATTAATTAATCTGAGAAATAATGAAAAGCTTATTAGCTTAAAAGTA
GTTAAGCAAGATGATGTTGAGGATAATCAAGATATAGAAAATAATGAAGATATAGAATCTTCTGATGTTA
CTGTGCAGAGTGAATAA

>lcl|NC_006570.2_cds_YP_170508.1_1383 [gene=ubiG] [protein=3-demethylubiquinone-9 3-methyltransferase] [protein_id=YP_170508.1] [location=complement(1652791..1653486)]
ATGATTAATATAGATAACAATGAAGTTGATAAGTTCTCTCGCCTAGCTGATAGCTGGTGGAACCATAATG
GTGAACTAAAAACTCTACATCAAGTTAACCCACTAAGATTAGAATTTATCAAAAAATTTACAAGCCTTGA
TAATAAAAAAATTATCGATATCGGTTGTGGCGGTGGAATACTTAGTGAATCACTTACGACAAATAATAAT
GATGTTTATGGTTTAGATGCCTCTTCAGAAGCCATAAATGTCGCTAAGCAACATGCTAAGCAGAATAAGC
TTAAAATTAACTATATAAACTCAACTATAGAGGATTTTGTCACACAAGGAAATTTAGACTTTGATATTGT
AACTTGTATGGAAATGTTGGAGCATGTTCCAGAGCCAGAGAGTATAATCGCCTCCATCGCAAAACTAATT
AAAAAAGACGGCTTATTTTTTGCTTCTACTTTAAACAGGAATATTAAGTCATATTTACTATCAATAGTTG
CTGCTGAGCATATTCTAAAAATGGTTCCACAAGGCACACATCAATATAATAAATTTATCAAGCCTTATGA
ACTGATAAAAACTACTGAGAAATATGGTTTTGTAGCATTGGAAATAATAGGTGTGCATTATAACCCTCTT
ACAAACAGTTTCAAACTTGCCAGTGGCGCTGATGTTAATTATATTATAGCTTTTAGAAAGGTATAA

>lcl|NC_006570.2_cds_YP_170516.1_1389 [gene=fumA] [protein=fumerate hydratase] [protein_id=YP_170516.1] [location=complement(1666398..1667912)]
ATGGCTGTTATCAAAGCAGAAGATCTAGTAAATAGTGTTGCAGAAGCATTGCAATATATATCTTATTATC
ATCCAAAAGATTTTATTGATGCAATGTATGAAGCATATCAGCGTGAAGAGTCAAAACCAGCTAAAGATGC

AATGGTGCAAATTCTAATAAACTCGAGAATGTCTGCTATTGGTAAGCGTCCGATCTGCCAAGATACAGGG
ATGGTATGCGCTTTTGTCAAAGTTGGCATGGATGCAAAACTTGACAAGACTGATAGAACAATTACAGAAC
TTATTAATGAGGGTGTGCGTCGTGGCTATAATGATCCGCATAACCCTTTGAGAGCATCTATGGTTTTTCC
GCCACATGGATCTAGAAAAAACACTAAAGATAATACTCCAGCAATTGTTCATATTGATTTAGTTCATGGT
GATAAAATTGAGGTTGATATTGCTGCAAAAGGTGGTGGATCTGAATTTAAATCAAAATTTAAGGTGTTAA
ATCCTAGTGATAGCATAATTGATTGGGTTGAAGAGATGTTGCCAACTATGGGGGCTGGCTGGTGTCCACC
TGGAATTATTGGTATAGGTATTGGAGGCACTGCAGAAAAAGCGATACTTTTGGCTAAGGAATCTCTTAGT
GAAGAGATTAATATCCAAGATATTATCAAAAATGGCCCACAAAATGAAACAGAGCAACTAAGATTAGATA
TCTATAATCGTGTCAATGCTCTGGGTATAGGTGCACAAGGCTTAGGTGGTTTGACAACAGTTTTAGATGT
CAAAATAAATGAATATCCAACACATGCTGCGTGTAAGCCAGTTGCTTTGATTCCTAACTGTGCAGCTACT
CGCCATGTTCATTTTGTTTTAGATGGTTCTGGTGCCGTTGATTTACCAGCTCCTAAGATAGAAGACTGGC
CAGTAATTGAGCAAGCACAAAATGATGATGTTAAGAGAATAAATCTTGATACAGTTACTAGAGAAGAGAT
TGAGGCATTAAGGTCGGGAGATAACGTTTTGATTAGCGGTAAAATCTTAACTGGTCGAGATGCTGCACAT
AAGCGTCTACAAGATATGTATAATGCTGGAGAAGATTTTCCTGTTGATCTTAACGGTAGATTTATCTACT
ACGTAGGTCCTGTTGATCCAGTAGGTGATGAGGTGGTTGGTCCAGCTGGTCCAACTACTGCTACGCGTAT
GGATAAGTTTACGCCATTTATGTTAGAGAAAGCTGGTATCATGGGAATGATAGGTAAATCAGAGCGTGGT
CAGGCAACGATTGATTCTATCAAGAAAAATAAAGCTATTTACTTCATGGGTGTAGGTGGTGCTGCTTATC
TCATATCTAAATCTATCAAAAAAGCAGAAGTTGTTGCATTTGAAGATCTTGGTATGGAGGCAATTTATGA
GTTTGAGGTTGAGGATATGCCAGTAACTGTGGCTGTTGACTCTTTAGGTGTTTCTGCTCATCAGCAAGGT
CCTAAACTTTGGAAACAAAAAATAGCTGAAATTAATAAGGTCTAA

>lcl|NC_006570.2_cds_YP_170519.1_1393 [gene=rpmB] [protein=50S ribosomal protein L28] [protein_id=YP_170519.1] [location=1671314..1671550]
ATGTCTAAAGTTTGTATAGTTACAGGTAAAAGACCTGCTACTGGAAATAATGTTTCACATGCACAAAACA
AAACAAAAAGAAGATTCCTACCAAATCTTCATGCACACAGATTTTGGGTAGAAAGTGAAAATAGATACAT
AAAGCTAAGAGTTAGCTCTAAAGGTATGAGAATCATTGACAAGAAAGGTATCGATACTGTTCTTAGTGAT
CTTAGAGCTCAAGGTCATAAAATTTAA

>lcl|NC_006570.2_cds_YP_170523.1_1397 [gene=minE] [protein=cell division topological specificity factor MinE] [protein_id=YP_170523.1] [location=1673320..1673592]
ATGCTAGCTAAACTTTTTGGATTAAGTAAAAAACAACAGAGTGCTTCAGTAGCTAAAGAAAGGCTACAGA
TCATTGTTGCTCATCAAAGAAGTGAGTTACATCCAAGATCTTCTAAGATAAGTAGCCACTTACTTGCGGA
ACTCAAAGATGAAATAATTGAAGTTGTCAAAAAATATGTTGCTTTGTCTGAAGAGAATATTAGAGATATT
GATCTAAAAGTTGAAGATAGTAGCAAAAATTCAACTATAGAAGTTAATATTCCTTTTAACTAA

>lcl|NC_006570.2_cds_YP_170531.1_1405 [gene=cysS] [protein=cysteinyl-tRNA synthetase] [protein_id=YP_170531.1] [location=1679445..1680839]
ATGGATTTTTGCTTTATGATTTTTTATAACTCTTTATCAGGACAAAAAGAACAATTTAAGCCGATTGAGG
CTAATAAAATCAAAATGTATGCTTGTGGTGTAACAGTTTATGATGATTGTCATATTGGTCACGCTAGGAC
TTATATAGCTTTTGATGTGATCAATAGATATTTTAAGTACCGTGGTTATGATGTTACATTAGTTAGAAAT
ATTACCGATATTGATGACAAGATTATCAAAAGAGCTAATGAAAATGGTGAATCTACCACTGAGTTAGTTG
AGAGAAATATTAAGGCAATGCACGATGTTTTTGCTAGGCTTAATATTCTTAAACCTTCTAAAGAACCAAG
AGCTACAGAAACAATTCCCGAAATGGTAGCAATGATCGAAACTTTGATTAAGAAAGGTTATGCTTATCAA
GGAGCTAATAGCGATGTCTTTTATCGCGTAACTAAATTTGCTGATTATGGTAAGTTAAGTAAGCAAAATC
TTGAGGCTCTTCAACAAGGTTCAAGAGTTGATGTGGTTGAGGAAAAAGAAAATCCAATGGATTTTGTACT
TTGGAAGATGGCAAAAGAAGGTGAGCCAGCTTGGGATTCGCCATGGGGTGCAGGTCGTCCAGGTTGGCAT
ATAGAGTGTTCTGCAATGTCTAAGAAACTTTTGGGCGATACTTTTGATATTCATGCTGGCGGTTCTGATC
TTAGATTCCCACATCATGAGAATGAAATAGCGCAATCAGAAGCTTGTAATGAATGTACTTTTGCAAATTA
TTGGCTACATTCTGGCATGGTCAAAGTAAATGCTGAAAAGATGTCTAAATCCTTAAACAATTTCTTTACG
ATAGTTGAAGTTTTAGAAGAGTATCATCCTGAAGTTGTTAGATATTTCTTAGCCTCAACGGTATATAGAA
GTGAGATTAACTACTCAAAAGAGAATCTTGAAAATGCAAAGGCTTCTGTAGAAAGATTATTTAATGCATT
AAGAGATATTGAGCCAATTGAGGTGAATCTTCCTGATGATGCTAGTGAGTATGAAGAGAAATTTATCAAA
GCTATGGATAATGATTTTAATACTCCAGAAGCTTTAGCTGTTTTATTTAGCTTAGCTAAAGAAATAAATA
CTCTTAAGACAACAAATAAATATAAAGCTAGTGGCTATGCATATTTGTTGCGAAAGCTTTGTGATGTTTT
GGGTATTTTGTTTACTGATATTGAAGAGTATTTCAAGCAAGGTGATGGTGCGGATGCCAGTGAGATTGAG
AAGCTTATAGCCGAGCGTACTCAAGCTAAAAAAGACAAAAATTATGTGCGTGCCGATGAGATAAGAAATC
AGCTCCAACAGCAAGGAATAATATTAGAAGATAGTGCAACTGGTACTACATGGAAGAAAGGTTAA

>lcl|NC_006570.2_cds_YP_170543.1_1414 [gene=putP] [protein=sodium/proline permease] [protein_id=YP_170543.1] [location=complement(1693525..1694991)]
ATGAGTCAAAATAGCATACTTTGGATTACATTTATAATATACATAATAATCATTTTTGGCATAGGGATAT
ACTCATATTTTCAAACTAAAAAAGTTTCAGATTATATGTTAGGTGGACGTTCCTTAAATGCACCAATTGC
GGCACTAGGTGCTGGAGCCTCTGATATGGGATCATGGTTATTATTAGCTCTTCCTGGAGCGTTTATGGTC

TCAGGTATTAACCAAATATGGCTGCCATTGGGGTTGACAATTGGGGCATTTATAAACTGGGGTGTTATCG
CAAGAAGATTGCGAATATATACTGAAATAGCAAGAGATTCTATTACTATACCTGCCTATTTTGAAAATAG
GTTCCACGATAGCAAAGGGATGCTAAGATCGTTAACAGCCATAGTTGTAGTAATATTTTTTACGATATAT
ATTGGTGCTGGTTTTGTCTCTGGTGGAGTATTATTTAGTTCAATGTTTGGTATTTCATATCATCAAGCAC
TATTACTTACAGCAGCAATAATATTCATTTATACATGCGTTGGTGGTTTTTTGGCAATATCTTGGATTGA
TTTTTTTCAAGGTAGTTTGATGCTGTTAGCTTTGGTTATTGTTCCAATTGTAGTGTATTTTGATATTGGC
AGTGACAATATTGGTTCGGTGCTATCAAATATTGATATCAAAGGATTTTACGATATTACATCAGGTGTGC
CATTAATTACGATTATCTCTCTTTTGGCATGGGGGTTAGGGTACTTTGGTCAACCACATATTATCGTACG
CTTCATGGCAATAAAAGATCCAAATAAGACTTCAAAAGCAATGTTTATTTGTATGACGTGGATGATTTTA
GCCTTACTAGGAGCTGCTTGTGTTGGTATTTTAGGTGCCGCATATTATAAGGATGGAATCGCAAATCCAG
AATCTGTATTTTTGAAGTTATCAGCAGTATTTTTTAATCCATGGATGGATGGCGTTTTATTAGCAGCAGT
ATTATCTGCAGTGATGAGTACTTCATCGGCGCAACTATTGTCACTATCTAGTGCTTTTTCGGTTGATGTG
TATGCTAAATTCATTCGTAGCAAAGCAAGTCACAGAGAGCTGCTCAATGTCAGTAGATTGATAGTGTTTT
TAGTAACAATTGCGGCAATTATCTTGTCATATAACCCAGATACTACGATTTTAAATTTGGTTGGCTTTTC
ATGGGCTGGTCTTGGTAGTTCGTTTGGAGCTGTTGTGATTTTTTCTCTATTTTGGTCAAGAATGAATAAA
ATTGGAGCTATAGCAGGTATATTATCAGGAGCGCTAGCTGTTTTAATTTGGTCTATGTTTGAGCATTTAG
GTGGCTGGTTTAAGGTATATGCGATGGTGCCTGGTTTTGCACTAAGTAGTATATGTATAATTGTATTTAG
CTTGTTAACTTCAAAACCAGAAGAGACAGTACAGTTGGAATATCAAAAATATAAACAATCATTATAA

>lcl|NC_006570.2_cds_YP_170546.1_1417 [gene=FTT_1633c] [protein=amino acid transporter] [protein_id=YP_170546.1] [location=complement(1696950..1698404)]
ATGTCGCAGAAACTTAAACGTGGCTTACATACTCGTCATATGTCAATGATAGCTCTAGGTGGCTGTATTG
GTACTGGTTTATTTGTAGCACTTGGTGGAGCAATCGCTGATGCGGGTCCTGGAGGAACTGTCTTGGCGTA
TGTTATTATAGCTATAATGGTTTATTTCTTGATGGCGAGTCTTGGCGAAATGGCTGCGCATAGCCCTGTT
AGTGGCACATTTTGTGAATATGCTACGCGCTATGTTGATCCAGCGTTAGGCTTTAGTACCGGTTGGAGTT
ATTGGTTTAATTGGGCTATTACAGTTGCTACAGAGGTTATTGCCGCAGCGTTAATTATGCAGTATTGGTT
TCCAGGTAGTTCAATTCTGTTGTGGAGTGGATTTTTCTTTGTACTAGTTTTTGCTTTGAATATCTTCTCA
GTAAAAATATATGGTGAAGTTGAATATTGGTTATCTTTTATAAAAGTTTCTACAGTTATTATATTTATAA
TTGTTGGTTTCTTATCAATACTTGGTTTAGTAGGTAATCACCAAAGTGTTGGTTTTCAGAACTGGCATAT
CGGAGATGCTCCTTTTCATAATGGTTGGTGGGGCTTTATATCAGTATTCATGATTGCTGGATTTTCTTTC
CAAGGTAGTGAGCTTATAGGTGTAACAGCTGGAGAAGCGAAAGATCCAAATACATCTATACCAAAAGCAA
TCAAACAAACATTTTGGCGTTTATTTATATTTTATATACTTGCTGTAGTGATTATTAGCTTCTTGATTCC
ATACAATAATCCATCTTTGATAAAAGCTGGAGCAAGTAATGATGTTTCAGTTAGTCCATTTACAATAGTT
TTTGAAAACGTTGGTTTGAATTCAGCGGCAACTATTATGAATGTAATTATATTAACTGCGATAATATCTG
CATGTAATGCAAGTATGTATAGTGCGACAAGGGTACTATGGCATTTAGGTAACATTAAGCAAGCCCCGCA
GTTTTTTGCAACTACTAATTCAAAAGGTACGCCAATGATTGCTCTTTTGGTTACAGCAGTTATAGGCTCG
TCATTCTTCTTTGTATCTTTTGTTGGTAGTGGATATATATTTACATGGTTAGTCAACGTTTCAAGTTTAG
CGGGATTTATTGCATGGTTTACAATTGCACTTAGTCACTATCGTTTTAGAAGGGCATATATAAAGCAGGG
TAAAAGCTTAGAAGATTTACCATATGTAGCAAAGTTTTTCCCATGGGCGCCTATTATTGCTTTAACTATG
GTAAGTATAGTAATCGTTGGTCAAGGTGTTACAATGTTGACAATGGAGGGTAGAACTTGGTTTAGTGTAA
TAATAGAGTTTTTATCAACTTATATAGGTTTCTTTGCATTTGTGATACTATATTTTGTATATAAGTTTAT
TAAAAAGACAAAATTGATAAGACTAGAAGATTGTGATCTTACTAGAGAATCTTAG

>lcl|NC_006570.2_cds_YP_170547.1_1418 [gene=FTT_1634c] [protein=hypothetical protein] [protein_id=YP_170547.1] [location=complement(1698652..1699320)]
TTGGCAAGATTTCATATCATATTAGGTTTAGTTGTTTGTTTTTTTGCATGGATATTCTTTCTTATATTCC
CGAATTTGGATATACAATTCGCGGGACATTTTTATAATTCATCGGCACATCAATTTATTGATGGGTATGA
TGGCTTTTTAGGATTTTTGCATTGGTTTGCTAGATTTTTTCCAATATTTTTTTCAATAATAGTGATTTTA
TTTCTATTAGGATCGTTATTTATCGATAAGTTTAAGATTAAGTATAGAAAAGCTATATTCTTTATTGCGG
TATGCTTATGGATAGGTCCAGGTTTAGTTGTTAACTATGTGTTTAAAGATCATTGGGGGCGTCCAAGACC
AGTGATGGTTAAGCAATTTAATGGTGATAAAATTTTTCAACCACCATTCGTTATATCCTCACAATGTGAT
AAAAACTGCTCCTTTGTATGTGGTGATGCCTCAATGGGATTTTGGCTTTTTGCATTTATGCCATTACTAG
CTACAAGAAAAAAGAAGCTTGTTGCGTTTATCGCAGCAGTAGTTGCTGGTGGAGGTTTGGGATTGATGAG
AATGTCGCAAGGAGGGCATTTTTTTAGTGATGTTGTTTTCTGTGGCATATTTGTGTATATCTCAACCTGG
GTGGTTTATGCACTAATGTATCGTAAAAAAGAATATTGA

>lcl|NC_006570.2_cds_YP_170548.1_1419 [gene=ftsK] [protein=cell division protein] [protein_id=YP_170548.1] [location=1699442..1701943]
ATGACAGATAACAACATAAACAAAACAAGTCATGCAGTTGGTAGATTAAAAATAACTCTAGTAGTTATTT
TAACAGCTAGCATAATATATCTTTTTATCGCCTTATTTAGCTTTAATATTAATGACCCAGGCTGGAGTAG
TGTTTCATCTGAAACCACAATAAAAAATTACGCCGGACCCGTTGGTGCATACATAGCAAGTTTTATACTT
TCAATATTTGGTGTTATCGGCTTTATTTTACCTTTTTTACTTATTGATTTTGTCAGAATCCTCCTAATCA
AACGCAAACAACAAAGTCTTAGCTATCTTTTATTTACAGTGAAGACTATTGGAATTATTGTTTTCATACT
ATCTTGCTGTGGGCTTGCTGAGCTTTATCTTAGTTTTGCTAACTACTGGGTGCCTCAACGCTCTGGTGGT

ATTCTAGGTTATGAATCAGTCAAACTCACAATCAAATATTTAGGCTCAGTAGGTGGTAACTTTGCATTAT
TAATAGCATTGTTAGTAGGTCTAACGCTGTATTCAGGCACAACATGGATATATCTATTCAAAAACTTAGC
GATATTTGTTGCCAAAGTGTTTACTTATATCACAAAATCAAAAACTAATGATAATAAAAATATCCCAGAT
ATAAATGGCTTTGAAAATTTTGAAAGCAAAAATAACCTAACAAGTCCACTTAATAGAGATAATAAAGTAG
TCTCAAGTATCTTTGAAGATAACCAACAAACTCATAAAAAAGATATTTTCAGAGAAGTTTTAGACAATAC
AAAAGTTACAAATGAACTTTCATTTAGAGATCCTAAAACTGAATCCCCACAAAACTCAGATTTAGAAATA
GTATCAGATTCAGATTCAATCTTAGACCTAGATGTATTAGATGAAGATATCGATTTAGACTCTGAATTAT
CATCACAATCGGATAATGAGAGTAAACCCGCGATGACAAAAGAGCAACTAAAAGGGATCACAACAGTGTC
GTCTCCAATAAGCTCAAGTGCCAGTAAAGCTCTCAATAAAAAAATGCTACCGTCTTTAGATCTACTTATA
GAACCAGAAGCAAAACAAACTGTTATTTCTCAAGCACAGCTTGATGAGACATCTTCATTACTTGAACAAA
CTCTAAATGACTTCAATATTAATGCAAAAGTAGTTGCAGCATATCCAGGTCCGGTAATCACAAGGTATGA
GATAGACCTTGCAAGGGGTACAAAGGTTAGCAAACTTACAAATATTGCCCAAGATTTAGCTAGAGCTCTA
TCTACTACCGCGGTTAGAGTTGTTGAGGTAATTCCTGGTAAACCGTACGTAGGCTTAGAACTACCTAATC
CAACTAGACAAATGGTGAGAATTAAAGAAGTGCTAGCAGCTCCGGAGTTTGTCAAATCTAAAGCCCCTAC
CCTAATGGGAATAGGTGTCGATATATCTGGTAAACCGACATTTGCAGAACTTGCAAAAATGCCACATCTT
TTAGTAGCTGGTACAACAGGTTCAGGTAAATCTGTTGGTGTCAATGCAATGATTCTTAGTATGCTATACA
AGTGTAGTCCTGATGAGCTCAAATTTATCATGATTGATCCAAAGATGCTTGAGCTTTCAATCTATGATGG
TATACCACACTTATTGACTCCAGTTGTCACAGATATGACAGAGGCTGCGAACTCTTTGCGTTGGTGTGTC
AAAGAGATGGAACGTCGCTATGCCTTAATGTCAGCTGCTGGAGTGAGAAATATTGCCTTACTTAATGATA
AGATTGAACAAGCAGAGAAAGTTGGTAGACCTCTCAAAGATACGATGTTTATCAAGATGAATCCAGAAAG
AGCGCATGAAGCACCATTACTTACAAAAATGCCATACATCGTTGTTGTCGCAGATGAGTTTGCCGATATG
ATTATGGTTGTTGGTAAAAAGGTTGAGGAGTTAATCGCTAGACTTGCTCAAAAAGCGCGTGCAGCTGGTA
TTCATATAATTCTAGCAACACAAAGACCATCCGTTGATGTTGTAACTGGATTAATAAAGGCAAATATCCC
AACAAGAATGTCTTTTCAGGTGTCATCTAGAATTGACTCTAGGACAATACTCGATCAGCAAGGCGCTGAG
CAACTTCTAGGACAAGGTGACATGCTGTATCTTAAACCAGGCTTTGGTGCTCCTATGCGTATTCATGGTG
CTTTTGTTGATGATAACGAAGTACATAGAGTAGTAGAAGCTTGGAAAGAATATGGTGAGCCAGAATATGT
TCAAGACATTTTAGAAGCAGCGGAAGAATCTGAAAATGGTGGTAGCCCAAGTAACAGTGGTGACAGCGAA
GACCCTCTCTATAACGAAGCAGTAGAGATCGTTATCAAAACCCAAAAAGCCTCAATCTCTGCGGTACAAC
GTAAGCTAAAAATAGGCTATAACCGTTCAGCACGATTGATGGAAGAAATGGAAGAAAATGGTATTGTTTC
AGAGATGAATCAAAATGGAATGCGTGAAGTTCTAATAAAGAGAGACTCTTAA

>lcl|NC_006570.2_cds_YP_170549.1_1420 [gene=lolA] [protein=lipoprotein releasing system outer membrane lipoproteins carrier] [protein_id=YP_170549.1] [location=1701944..1702561]
ATGAAAAAGATAATTATATGTTTTATATTTGTTTTTAGTATCAATGTTAGCTTTGCTGATGCCACTAGCG
AGCTTATAGACAAGATAAAAAATATCCACTCGATGACTGCTAACTTCAATCAAAAGCTCATAGATGGACA
AACCAACAATAATCTTAACTCAAAGGGCAATATGAGCCTTAAAAAACCACAATATTTTAAATGGATAACA
ACATCTCCAAATAACCAAGAAATTGTCTCTAATGGTACAAAACTATGGATTTATGATGGTGATTTAGATC
AACTTATCATAAAAAAAGTTTCTAATGACATAGCTCAATTTCCTTATCTGATTCTTTTATCAAAAAATAC
CAATAACATCAATAAACTTTTTACTGTCACAGCGCAAGATAACAACAGCTATATTCTAAAACCTAAAAAT
GATCAAATGATCGATAGTATAAAAATTAAATTTACTCCAAATAACCAACTTGAGTATTTAGAGATTTCAA
CTTCACTAAATCAGTTCACAAAAATTGAGTTTAATAATGTAAAAACTGATGTAGATATAAGTAATACAAG
TTTTGATTTCAAAGCGCCTCAAAATACAGATATAATTGATGAAACTAAATTCGCATAA

>lcl|NC_006570.2_cds_YP_170550.1_1421 [gene=FTT_1637c] [protein=hypothetical protein] [protein_id=YP_170550.1] [location=complement(1702959..1703162)]
ATGATGAAACTTATATTGCGCTTAGCTCATTTGTTTGATAATAAAAAAGATAGAGCATATGTGAGCGAAG
TGGATAGATTTTTACAAGAGTTTGACAAAGCAAATCCACAAAAATCTGAGTCGCAAAAGAAAGAAATATT
AAAGCATCGAAATATTTTTAATAGAGAAGCAAAGCCTAAAGCTAGTTTCCTTGATGGTGAATAA

>lcl|NC_006570.2_cds_YP_170551.1_1422 [gene=trkH] [protein=potassium uptake protein] [protein_id=YP_170551.1] [location=1703274..1704728]
ATGATGTTAAGCCAGAAACCAAAAATAATTGGTATATTTTTAATGTTCCTTAGCCTTACCATGCTTAGTC
CTTTATTGGTTGACTATATATATGATGAAGATAATGCATATCCATTTGTGTTGAGTTTTACAGTAACATT
TTTATGTGGTTTTTTGCTTTGGTTTATTTCACGTAAATCAAATAAGAAACTATCAAATAGAGATGGTTTT
CTTATAGTTACACTCGTTTGGATATTTGTAACAGTCTTTGGTGCAATACCATATATGTCTTTTCCTGGAC
TAAATCTATCCTTTACTAATGCAGTATTTGAGTCTGTTTCTGGATTTACTACAACTGGTGGTACTGTGAT
TGAAGGACTTGATAAGCTTCCTCATAGCATTTTATTTTATCGACAACAAACTGAATTTTTCGGTGGCATG
GGCATTATTGTTCTATCAGTAGCAATTCTACCTTTACTAGGTGTCGGTGGTATGCAGTTATATAAAGCGG
AAGTATCAGGTCAATGGAAAGATGATAAAATCGCCCCTAAAATCTCCAGCACTGCCAAAGCACTTTGGAT
GGTATATCTATTACTTACTTTCTTATGCTTTATCTCTTATCTATTAGTTGGTGTAGAACCATTTGATGCG
ATATGCTATACATTCTCAACAGTATCAACTGGCGGTTTTGCGCCTTCGGATGCAAGCATGACAGATAAGC
CATTGGGAATGCTTATTGTATGTGCAATCTTTCTTTTCTTAGGTGCTACTAGTTTTAAAGCCCATTATAT
AGCTCTGTCAAAATTTAAAATAAGTCATTATTTTAGAAATATCGAGTTTAAAGCATATTTTTACTTCTTA

TTTTTTACCTCATTTATTGTATGTATCACTATAATTGCACATACAAATGACCTTTCAAATATTTTCTCAA
TAGTTACTAATAGCATTTTTCAAGTAATATCCATCAGCTCAAGTGCTGGCTTTGTTTCTGATAATAACTA
TTACTTATGGCCTAGCTTCTTGCCAATTATGTTAATGTTTATTGCCATAATTGGTGGTTGTGGTGGCTCT
ACCGCTGGTGGCTTAAAGATGATTAGAGCAATTTTATTTAAAGAAAAAGCTATACTTGAGGCTAAACGTG
TCATCCATCCACAAGGGGTTTTCACTGTTAAATTAGGTGATATCCATATATCAGAACAAGCTCTTAATAG
AGTTTCTGGATTTATCTCTGTTTATATTATAATTTTTGCTGGTGGTTGGTTAGCCTTGCTAGGTTGCGGT
CTAGATATCCCTACAGCATTCTCAACAATTGCTACCACTTTGTCTAATGTGGGTCCGGGACTAGGTGATA
TTGGTTCAAATTTTAAAAATCTCCCTAAAGAAGCTCTATGGATATGCAACTTTGCGATGATTGCAGGACG
TCTTGAGATATTTACAATTCTAGTATTATTTATGCCAGATTTTTGGAGAAAATAA

>lcl|NC_006570.2_cds_YP_170577.1_1447 [gene=ribD] [protein=riboflavin biosynthesis protein ribD] [protein_id=YP_170577.1] [location=1740586..1741653]
ATGAAAAATATCGATAAATATTATATGCAACAAGCACTTACTCTAGCAAATAGAGGTAGACTTACAGTAT
CACCTAATCCAATGGTTGGTTGCATAATAGTAAAAAATGGTGCAATTATCTCGGAAGGTTGGCATGAAAC
TGTAGGAGAAGCGCATGCAGAGGTTCACGCACTGACAAAAGCGGTTGATAAAGCAAAAGGAGCTACAGCA
TACGTAACACTAGAACCATGCTGTCACTGTGGAAGAACTCCTCCCTGTACTGATACCATAATCAAAGCTG
GGATAAAAAAAGTAATTATTGCTACTCTTGATCCAAACCCTAAAGTTGCTGGCAAAGGCGTCGAAAGACT
CAAAAATGCTGGTATAACAGTCGAAGTAGGCTTACTAGAGAAACAAGCACAAGAGTTAAATAAAATATTT
TTTCATTATCAAACAACCAAAAAACCTTTTGTTTATGCAAAATGGGCTATGTCATTAGATGGTAAAATAG
CTGTTAATGATGGTGACTCAAAAAAAATAAGCTCACATCAAGCATTCGTAAATACTCATGAATTGCGCAA
TATTTGTGACGCTATCTTAATTGGCAAGCAAACTCTAATTGATGACAATCCAAGTCTTGATGTAAGAATA
AATATCAATAAAATAAAACATCCAACTAGATTTATACTTGCTAATCATCTGACAACAATAAATCATAATT
GGCGAGTTTTAGATCAAAGGCATGCCAAAACGATTTTTGTTTGTTCAAAGATCTCTGCACGAGTAGCAAC
CAAGCTTAACCAACTTGGCATTGAATATTGGCTATTACCACAAAGTCAACATCAGGTTTGTTTAGATACC
TTACTTGAGAAGATGGGTAAGATTGGAATAACTAGTCTTCTTGTTGAAGGTGGTAATAAAACCCTAAATA
GTTTTATTAATCAAAAACTTGTTAATGAATTTTATACATACTTAGCTCCGGTAATAATAGCTGATTATAA
CCCCAAACAGCAGCTAAGCTTCAATCAAATCTCTGTAAGAGAGGATATAATAATAAACTCTTGTTTTAAG
GAAAATTCTAATGTTTAG

>lcl|NC_006570.2_cds_YP_170578.1_1448 [gene=ribB] [protein=riboflavin synthase subunit alpha] [protein_id=YP_170578.1] [location=1741646..1742251]
ATGTTTAGTGGCATAGTTCAGCAACTAGGAACAATTAAAAAAATAACAACTAAAGATAGTCTAAAAACTT
TTTGTATCAAATTTGATAATAGTCTTCAATGTAGCATTGGTGATAGTGTTGCGATTAATGGTACCTGTCT
TACTGTTACAAAACTTGATAAGCAAAACTTAACAGCTAATTTTGATGCAGTACCAGAGACACTTAAAAAA
ACTAATCTTGATACTCTTAAGGAGAACCAACTAGTAAATATCGAACTTGCAATGCGCTATGGTGATCATA
TTGGTGGGCATATGGTACAAGGTCATATTGATGAACCAGGGCAAATCAAAAGTATTCAAAATGTTGGTGG
TGCATGGTTAATTGAAATTTCTGCTTCTAGAGAATTTCTAAAATACTTAGTAAAGAAAGGTTTTGTAACT
ATAGATGGTATGAGTATAACTGTAATAAATGTACTCAAAGATTCATTTACTGTAACTTTGATACCTCACA
CCATTGAAGTTACTATCGCAAAAAACTATAGTAATGGTTCTATGGTAAATCTTGAAGCTGATGCAACAGG
TAAATATATCTATAAATATATACAAGGATTTAAAGAAAATGTTTGA

>lcl|NC_006570.2_cds_YP_170579.1_1449 [gene=ribA] [protein=3,4-dihydroxy-2-butanone-4-phosphate synthase] [protein_id=YP_170579.1] [location=1742244..1743455]
ATGTTTGAGCAAATAAAAAATAATGTTGAGAATGCCATAGAAGCTCTTAAGCAAGGCAAACCCGTAGTTG
TACTTGATGATTATGATAGAGAAAATGAGGGTGATCTAATTCTACCGGGTCAAAAAGCTACAGAAGAAAA
TATCGCATTTATGCTTGAGCATACAAGTGGCATCATTTGTTTGGCAATGGACTCAAAGAAAGCACGAGAA
TTAAATCTAACGCCAATGGTAGCTGCTGACCAAAACAATAGTACTTTTACAACTCCTTTTACTGTAACTA
TAGAGGCTAAAGAAGGGGTAACTACAGGAGTATCGGCAAAGGATAGAGCACATACTATTCAAGTTGCATC
AAAAGCTGATTCTAAAGCAGAAGAATTAGCTAGACCTGGACATATATTCCCTCTTATAGCCAATGATAAA
GGCGTACTTGGTAGAAATGGTCATACTGAGGCAACTGTTGATCTTATGAAACTAAGTGGTTTTAATAGCG
CTGGAGTTCTATGTGAGCTAATGAATAAAGATGGCACCATGATGAAAGCTGCTGAACTAGAAGCATTTGC
TAAAAAGCACGATCTGCCTCTTTTAACTATTGCAGAGCTTTACCAATATCGTTTAGCAACTGAAAATTTT
GTCACGAAAATGGCAAGCTCTACTATACCATTCAAGAAAATTGGCGAATTGGAAATGAGTGTGTATAAAG
ATAACTTTAGTGGTGATGAAGTTGTTGTTTTATCTAAACCTTATTCTGGTAATAATCCTTTAGTAAGAAT
GCATTCATCTTGCATAACAGGAGATATTTTTGGCTCGCTACGCTGTGACTGCCAAGATCAATTACATAAA
GGAATTGAAATGATTAGCGAAGAGGGTGGCTTTTTTATATATCTTGACCAAGAAGGTAGAGGTATTGGTT
TAACAAATAAGCTTAAAGCCTATAATTTGCAGATGAATGAAAATATGGACACTCTTGAAGCTAATTTAGC
TCTTGGCTTACCCGCTGACGCAAGAAAATACGATCTAGCTATACAAGTCCTAAAATATAACAATGTCAAC
CGCTGCCGATTAATATCTAATAATCCTGAAAAGCTAGCTGCTCTTAGAAACGTTGATATAGAAACACAAC
CAGTATATTGTGAGGCATTTGTAAACTCTCACAATAGAAACTACTTGATAACTAAAAAAATTAAAGCAAA
ACATACAATTAAAGGAATATAG

>lcl|NC_006570.2_cds_YP_170580.1_1450 [gene=ribH] [protein=6,7-dimethyl-8-ribityllumazine synthase] [protein_id=YP_170580.1] [location=1743465..1743908]

ATGAGAAAATTAGCAATCGTTGTAAGTGAATTTAACTCTTTGATAACTGATAAAATGCTTGAGGGTGCAT
TAGAAGAAGCATATGCTCAAGGCTTAAAAGATAGTCAAATTTGTATCAAAAAAGTTCCTGGAGCAGTTGA
ACTCCCATATGCAGCAAAGCTCCTAGCAGAAACTAAAGAGTTTGATGCAATAGTATTACTAGGTTGCGTA
ATCCGAGGCGAGACAGATCACTATGATTATGTTTGCGATCAGGTAAGTTATGGTACACAAAAAGTCATGC
ACCAATATAATTTACCAGTAATTTTTGGTATATTAACCACACACAACAAAGAGCAAGCCTTAGAAAGAGT
CGGTGGCAAAAAAGGTCATAAAGGTAAATACTCTATCCAAGCTGCCATAACGATGGCAAAGATGAAAAAA
GATATTTTAGAACAAGGAGTATGA

>lcl|NC_006570.2_cds_YP_170581.1_1451 [gene=def2] [protein=peptide deformylase] [protein_id=YP_170581.1] [location=1743905..1744429]
ATGAATATGTCTTTAGAAATTCTAAAGTACCCTCACCCAGTTTTAAAAGAGGTTGCTAAAGAAGTTACAA
AAGATGAAATCAATGATGATTTACGTGCAACTATTGCTGAAATGCATGAGCTAATGCTAGAAGCAAATGG
CGTGGGTTTAGCAGCAATACAAGTTGGTATCAAAAAAAGATTCTTTATCATGTATGATAACTTAGAAGAG
CAAAACCCTGAAATAATCACTATCATTAACCCTGAAATAATTGAACAAAACGGTAAAATAATTGATGAAG
AGGGCTGTCTTTCTTTTCCTGGTGTTTCCGCAAAGGTAAATAGAGCCACTGTAGTCAAAATAAAGGCACT
TAATGAATTTGGAGAAGAGATTGAAGTTGAAAAAGATGGATTTTTAGCTAGATGCATCCAACATGAGATA
GATCATCTTAATGGTATAACCTTCTTTGACCATCTTGGTTCACTAAAGCGCAAAATGATAGAGAAAAAGT
ATAAAAAACTAATGCAAGAAAATGCTAAGAGTTAA

>lcl|NC_006570.2_cds_YP_170583.1_1453 [gene=mnmA] [protein=tRNA-specific 2-thiouridylase MnmA] [protein_id=YP_170583.1] [location=complement(1745523..1746668)]
TTGGCTCTGTTGAAATTCCTCAAGAAGCTTTTTTATCAGTGCTTAAAAAATAAAATTATAGTTATTATGG
AAAATAAAAAAGTAATAGTAGGTATCTCAGGAGGTGTGGACTCATCAGTTTCAGCTTTGCTCTTGAAACA
GCAAGGTTATGATGTAACAGGTGTTTTTATGAAAAACTGGGAAGAAGATGATACAGATGAGTTTTGCTCA
GCGGAGCAAGATATCGCTGATGCTCAAGCTGTTTGTGACTCTATAGGGATACCTTTCAAAAAGATTAATT
TTGCCGCTGAATACTGGGATAATGTTTTTGAGCATTTTCTAATAGAGTATAAAGCTGGCAGAACACCTAA
TCCTGATATCTTATGCAATAAGGAAATCAAATTTAAAGCCTTTCTGAGCTATGTACATCTTTTAGGCGGT
GATTATATCGCTACAGGACATTATGCTCAAACAAGACTAGCCGCTGATGGTTCAGTACAGTTAGTCAAAG
GACTTGATGATAATAAGGATCAAACATATTTTTTATATACTCTAGGTCAAGAGCAACTTAGACAGACGAT
ATTTCCTATAGGCAACATTGAAAAATCTAAAGTGCGTGAAATTGCCAAAGAAAATAATCTTGTAACATTT
GATAAAAAAGATAGTACTGGTATCTGCTTCATAGGTGAACGTAAGTTTAAAGAATTTTTGTCTAAGTATT
TGCCTGCTCAAAAAGGTGAAATCCATGATGAAAATGGTATCAAGATCGGTATGCATGATGGATTGATGTA
TTATACAATTGGTCAAAGACAGGGGCTTGGTATAGGTGGTGTAAAAGATCGCCCTGAGGTACCTTGGTTT
GCAGCTAAAAAAGATTTAGAAAACAATGTCTTGATTGCTGTGCAAGGTCATGATCATCCATTACTATTTA
AGCAGTCACTACAAGCTATCGAGCTAAGTTGGGTTGCAGGTATGGCTCCGGCAGATAAATTTAGATGTGC
AGCAAAAGTCCGCTATAGACAAAAAGATCAGTCTTGTGAAGTCGAAGTAAATCAAGATGGTTCAGTTAAT
GTAACTTTTGATCAGCCACAAAGAGCAATCACACCAGGACAGTCAGTAGTATTTTATATTGATGATGTTT
GCTTAGGTGGGGGAGTAATTATTTAG

>lcl|NC_006570.2_cds_YP_170585.1_1455 [gene=rpsT] [protein=30S ribosomal protein S20] [protein_id=YP_170585.1] [location=1748670..1748942]
TTGGCTAACTCAAAACAAGCAAAAAAGAGAATTATTCAAGCTGAAAGAAATCGTCAACATAATGTTGCTC
GTCGTTCAATGATGAGAACTTTCTTAAAGAAAACTGCTTATGCAATTGAAAAAGGTGATGTAGAGGCAGC
AAAAGAAAACTTTGCTAAAGTTGTTCCTATATTAGATAAGTATGCTTCTAAAGGTCTAATCCACAAAAAC
AAAGCTGCTAGACATAAATCTCGTTTAAGCGCTAAAATCAAAGCTCTTGCTACAGCTGCTTAA

>lcl|NC_006570.2_cds_YP_170586.1_1456 [gene=olmA] [protein=outer membrane lipoprotein] [protein_id=YP_170586.1] [location=complement(1749016..1749372)]
ATGTTTAGACCAATAACTAAAAATCTATGTTTATTAATAGCTGCGTTTAGTTTATCAGCATGTGGAATTA
TACAACCATACACAGCTCCTGTACCGCAAGGTAAAGAGATAAAAGATAAAAAGCTTTTTGAAATTAAGCC
AAATATGACAAAAAGTGAGGTTACTTATATTCTTGGTTCTCCTGATATTATAGATACTTTTAATCCTAAT
CAATATGTATATATAAATACTTATAAGAGAAATATGCAAGATACTCAGTTTAGTGAGTCAAAGCTAATAT
TAACCTTTAATAATCAAGATAGGCTCATAGGTATATCAGGTAACTATGCGCCACCAACTAAGGATCCTGT
ATTCTAG

>lcl|NC_006570.2_cds_YP_170587.1_1457 [gene=lpcA] [protein=phosphoheptose isomerase] [protein_id=YP_170587.1] [location=complement(1749380..1749976)]
ATGACTTCTTTAGATAAAATCAATAGTTATTTTGAAAGTAGTATTCAAGCTAAAATAGAAACTGCAAATG
CACTACCTCCAGCTATTGCGCAAGCTGCAAAAGCTATGGTTTCTTGTCTAGAAAATGGTGGAAAAGTCCT
AGTTTGTGGCAATGGCAGTTCAGGAGTTATCGCTCAGCATTTTACATCTAAATTATTAAATCATTTTGAG
ATGGAGCGTCCTCCTCTTCCTGCAATAGCTTTGACAGGAGATGTTGCAACTATTACAGCTGTTGGTAATC
ATTATGGTTTTTCACAAATTTTTGCAAAACAAGTTGCTGCTCTAGGTAATGAAGATGATATTTTATTAGT
TATTACAACTAGTGGTGATTCTGAGAATATCCTTAGTGCTGTAGAAGAGGCACATGATCTGGAAATGAAA

GTAATAGCATTAACTGGTGGCAGTGGCGGAGCACTGCAGAATATGTATAATACCGATGATATTGAACTAA
GAGTGCCATCAGATAATATAGCAAATATTCAAGAAAATCATTTTCTCATAGTTCATTGCTTATGTGATAT
TATCGATCAGAAATTATTTGCAGGCTTAGAAGACTAG

>lcl|NC_006570.2_cds_YP_170600.1_1469 [gene=groES] [protein=co-chaperonin GroES] [protein_id=YP_170600.1] [location=1763937..1764224]
ATGAACATTCGTCCATTACAAGATAGAGTATTAGTTCGTCGTGCAGAAGAAGAAAAAAAATCTGCTGGTG
GAATTATCTTAACTGGTAGTGCTCAAGAGAAACCTAGCCAAGGTGAGGTTGTTGCTGTTGGTAATGGTAA
AAAATTGGATAATGGCACTACGCTACCTATGGATGTAAAAGTTGGTGATAAAGTGCTGTTTGGTAAATAC
TCTGGTAGTGAAGTAAAAGTTGGTGATGAAACTCTTCTAATGATGAGAGAAGAAGATATCATGGGTATTA
TTGCATAA

>lcl|NC_006570.2_cds_YP_170601.1_1470 [gene=groEL] [protein=molecular chaperone GroEL] [protein_id=YP_170601.1] [location=1764262..1765896]
ATGGCTGCAAAACAAGTTTTATTTTCAGATGAAGCTCGTGCAAAAATGCTAGATGGTGTTAACACACTAG
CAAATGCTGTAAAAGTTACTTTAGGTCCAAAAGGTCGTAATGTTGTTTTAGATAAATCATTTGGCACGCC
TACTATCACTAAAGATGGTGTATCTGTTGCTAAAGAAATTGAACTAGAAGATAAGTTTGAGAATATGGGT
GCTCAGATAGTTAAAGAAGTAGCTTCAAAGACAGCGGATGTTGCTGGTGATGGTACTACTACAGCGACTG
TACTTGCTCAGGCATTATTGACAGAGGGTCTAAAAGCTGTCGCTGCAGGTATGAATCCTATGGATCTAAA
AAGAGGTATCGACAAAGCAACTGCTAGGTTAGTTGAAGAATTAAAAGCACTTTCTAAACCATGTTCAGAT
CCAAAATCAATTGAGCAAGTTGGTACTATCTCTGCTAACTCTGATGCTACTGTAGGTAAGCTTATCGCTG
ACGCAATGGCAAAAGTTGGTAAAGAAGGTGTGATTACAGTTGAAGAAGGCAAAGGCTTTGAAGATGAGCT
TGATGTAGTTGAAGGTATGCAGTTTGATAGAGGTTATCTATCTCCGTATTTTGCAACAAATCAAGAGAAT
ATGACTACTGATTTAGAGAATCCATATATTCTAATAGTTGATAAGAAAATCTCTAATATCCGCGATTTAT
TACCGATATTAGAAGGTGTTTCTAAATCTGGTAGAGCGTTACTAATAATAGCTGAAGATGTAGAAAGTGA
AGCTCTAGCTACTTTAGTTGTAAATAATATGCGTGGTGTAGTTAAAGTATGTGCTGTCAAAGCTCCTGGC
TTTGGTGATAGAAGAAAAGCTATGCTAGAAGATATCGCTACTCTAACTGGAGCTACGTTTGTATCAGAAG
ACCTAAGCATGAAGTTAGAAGAAACTAACATGGAGCATTTAGGTACGGCTAGTAGAGTACAAGTAACAAA
AGATAATACAACAATTATTGATGGTGCTGGTGAAAAAGAAGCTATCGCTAAACGAATAAATGTAATCAAA
GCTAATATTGCTGAAGCTAACTCTGATTATGATCGTGAGAAGCTGCAAGAAAGATTGGCTAAACTTTCTG
GTGGTGTCGCGGTGATAAAAGTTGGTGCTGTTACAGAAGCTGAGATGAAAGAGAAGAAAGATCGTGTCGA
TGATGCTTTACATGCTACTCGTGCGGCTGTAGAAGAAGGTATTGTTGCTGGTGGTGGCGTTGCTTTAATT
AGAGCACAAAAAGCATTAGATGGCTTAACAGGTGAAAATGACGATCAAAACTATGGTATAGCGCTACTTA
GAAAAGCAATAGAAGCTCCTCTAAGACAGATAGTATCAAATGCTGGCGGTGAGTCTTCTGTAGTTGTTAA
CCAAGTTAAAGCTAATCAAGGTAACTATGGTTATAATGCTGCAAATGATACTTATGGTGATATGGTTGAG
ATGGGTATTTTAGATCCTACTAAAGTTACTCGTTCAGCTCTACAACATGCTGCTTCAATTGCTGGACTTA
TGATCACTACAGAGGCGATGATCGGTGAGATCAAAGAAGCTGCTCCTGCTATGCCTATGGGCGGTGGCAT
GGGCGGTATGCCTGGCATGATGTAA

>lcl|NC_006570.2_cds_YP_170625.1_1494 [gene=FTT_1723c] [protein=hypothetical protein] [protein_id=YP_170625.1] [location=complement(1808886..1809530)]
TTGATGCCACAAGTCTCAGCTTTTATTTTAGATTTTGAGAAATATAAAGCTGAGGACCTTAGAGGATATC
TTTGTTGCAGAAATATTGATTCAGCAAGGTTTAATAATAAGCAAAAAATATTTTCACAGTTTATTCGCTA
TTTTGTCTTAGAAGAGTTTTATCATATTTCTTCACCAATTTTTTTAAATCAACATGGTAAACCGTACTTG
GCAGATAACAGTGTTTTTTTCAATATTAGTCATACACAGACAAAGCTTATAATTGCTGTTGCTGATCAAG
AGATTGGTGTGGATATTGAAAAACTATCAGCTAGACGGAATATTATAAGAATCGCACAACGTTATTTTAC
TAGCTTAGAAAATCAATGGCTTGCTGCTAGTGATAATCCCGTCAAAGACTTTTATACGTTATGGACACTC
AAGGAAGCTCAGGTAAAAAGAGACTCGCTTGGGATAGCTAAAGGCTTGAGTGGTGCTAATTTTAGTAAAA
TCAATGACTCATGGCTTAGTAATAGTTATCCAGATGATTTTGTGACATTTTCATATGATGAATCAATAGT
TTCGATATGTTGTAAAAATATTGTCGCTCAGAAAATTAATCTATTCGAGATAGTAGATTTTAAATTTAAA
CAGATACAGCTTTAG

>lcl|NC_006570.2_cds_YP_170630.1_1499 [gene=nhaD] [protein=Na+/H+ antiporter] [protein_id=YP_170630.1] [location=1814742..1816169]
ATGTACAAAAAGATTATACTAATTTTATCAATAGTGCTGCTACCAATATTATCATTTGCAGAAGATGAGA
AAACACTAAGTGCTGTCAGTGTCAATGCGGTTAATCATCCTTTGGCAATAGCTGCTATTGTAGTTTTTAT
TTTAGCTTATCTACTAGTAATGACCGAGGATTTTACTAAACTAAATAAATCAAAGCCTGTAATTGTAGCT
GCTGGAGTAATCTGGATTCTAGTGGCTATTGTTGGTGAATCTCTAGGTGCTGATAATATTGTTCATAGCA
ACTTCAACCATATTATGACCGAATATGGCGAACTTTTACTTTTCTTATTAGTTGCTATGGCATATATTAA
CTTAATGGAAGATCGTAACGTTTTTGCTAAACTAAAGAGTTCTCTTTTACGTGCTGGTTTTGGTTATCTA
GCGACTTTTTGGCTTACTGGTATAATTGCGTTTTTCCTATCAGCAGTTGCGGATAACTTAACTACCGCTC
TAGTTATGAGTACCGTTGTAATATCTATCGGTAAAGATAATAAAAAATTTATAACGATGGCTTGTGTCAA
TATCGTAGTTGCCGCAAATGCTGGTGGCGCTTTTTCACCATTTGGTGATATTACTACGCTTATGGTTTGG
CAAACAGGCGTTATTAAATTTACTGAATTCTTTGCAATATTCTTACCTTCACTTGTCAATTTCTTAGTTC

CTGCGATTATTATGAGCTTTTTTATACCGAAAATGGAAACTCAATCAATAATTGAGGAAAAAGTTGAACT
TAAACGTGGTGCTATTCCTGTTATAGTGTTATTCATTCTAACAATTGCTACAGCAGTAGCATTTGAACAT
ACGCTTCACTTACCACCATCATTAGGAATGATGACAGGTTTTGGCTATGTAATGATTTATAATTATTTCT
ACGGTCTAAAAATAGCCCATGAAAATAAAAATCCAAAAAAACATAAGATGCCATCACATGCTTTTGATAT
TTTTGATAAAGTTAAAGAAGCTGAATGGGATACATTACTATTCTTCTATGGGATACTAGTTTCTGTACAA
GGTCTTGCTGCTCTTGGCTATTTAGGTATAGCTTCCCAATACATTTACACAGATATGCAATCTATTGCCC
CAAGCCTATTTTCCGCACATACTCAAGCAAACACTATAATTGGTATACTCTCAGCTATCATTGATAACAT
TCCCGTTATGTTTGCTGTATTAAGTATGAACCCTACTATGGAGCATGCTCAATGGCTCTTAATCACACTT
ACCGCAGGCGTTGGGGGTAGCTTATTAGCTATTGGATCTGCAGCTGGAGTTGCCGTGATGGGTAAATCTA
AAGGTAAATATACATTTATTGGTCACCTAAAATGGACTTGGGTAATTGCACTAGGGTATTTTGCGAGTAT
TATCGTTCATCTTCTAGTCAATCACTAG

>lcl|NC_006570.2_cds_YP_170631.1_1500 [gene=FTT_1730c] [protein=amino acid transporter] [protein_id=YP_170631.1] [location=complement(1817820..1819694)]
ATGAAGATAAAAAACCTGATATTTGGTTCACCAATTCCTAATGCAAAACAGCAAGAACAAAAAATAGGTT
TATTTGCCGGTTTTGCGATACTTTCTTCAAATGCACTATCTTCTGTATCATATGCAACAGGAGAGATATT
TATAGTCTTGGCAACTGCTGGAGCAGCAGCTGTTATGCAATACTCGATCGAAGTATCAATAATGGTGATA
TTACTAATATTATTGATGGGTTTTTCATATGCACAAGTAATACGTGCTCATCCAGAAGGCGGTGGTTCTT
ACTCAATAGTTAAAACACATTTTAATGAAAAGCTTTTATTGTTAACTTCGGCATCTCTTATAATTGATTA
TATTCTTACGGTAGCGGTTTCTGTTTCTACAGCAGCTGTAGCAATTAGTTCAGCTTTGCCTGTTTTAGAT
CATTATAGTGTTAAGCTTGCTTTAGGTCTTTTGGTACTTTTAATGATTATAAATCTTCGTGGTGTTAAAT
CTACTGCAAAAATTTTTATCTGGCCAACTTATATGTTTATTGTTTCAATTATTATCATGATAATTGTAGG
GATATATCAATATAATCATAACTCTTTGCATACATTTACATATACGCAAAATCAACTTGATCACATGCAA
GCTTCAATGGGTGTTTTAACTATAACTCTTTTGCTGCGTGCTTTTTCATCAGGTAGTGCTGCGTTAACAG
GTATTGAATCATACGCAAATGGGGTTTCAGCTTACAAGTCTCCAACTTTAGCTAAATCAATAATTGGTTT
GTTAACTATGACATTCTTATCAATACTAATGTTTGCTGGAGTTACTTTTATTGCTGCAAAAACAAGGATA
TTGCCAGATTTTTCAGAAAGTGTCCTATCACAAGTTGCTCATCAAGTTTTAGGTAATGGCTTTTTATATT
ATTTCTTGCAAGCATCTACTTGTTTGATTCTACTTATGGCGGCTAACACTTGTTTTACTGGATTTCCTAT
TTTAGCCTCGATTATGAGTAAGGATAACTACCTTCCCGAACAGCTGCAGCGTGTCGGTGATAGATTTGCA
TTTAGAAATGGTATTATAATGTTGACTATTTTATCAGCGATCTTAGTCATAATATTTGATGCTAAAGTTA
GTCTTCTAATACCATTATATGCTTTTGGCGTTTTTATTGCATTTACGCTTTGTCAAGCAGGATTAGTTAA
GTACTGGTATAGAAATAAACGTAAGTATAAAAGTTGGGGAATTAGAGCATTTATAAATGCTTTTGGTTGT
GTTGCTACATTTGTAGTATTAATTACAATCGTCGAGAGTAAATTTTTTGAAGGCGTTTGGATTGTCATAA
TTGCGATAGCGATTATCATGTATGGTTTATATGCGATAAAAACTCATTATATTAGAAGAGAGCATAACCT
GGCTCTAAGTGTTGATGAGGCTGTTGTAAACGCTTGTATACATGAAAATCTCAAACCAAAAATAGTTGTT
TTAGTTTCACGTATTCACCGTGGTACAATAGAGGCACTAAGATTAGCAAGAAATCTATCTGATGATATCA
CTCCAGTTTTTGTTTCGGCAAACCAAAAGAAAATTGAAAAGATTAAGTCTGAGTGGAAAAATTTAGCTTT
CAAAGAAAAACTCTTGATAATGCGACCAGTATATAACTCGTTTATAACTCCAGTATTGCAAATTTTGCAT
AAAAATGATTTAAGAGATCCTGAGCGAGGCTATTCTGTAGTGATAATTCCAGAAGTTGTAAATACAAAAT
GGTGGCATTTTTTGTTACATAATCAAAATTCGCGCATGGTTAAATTAGCTATAGCAGCAATGGATAAAAA
AGATGATAAAACTGCTACTAGAGTGGTGATTTCAGTTCCATACAAAGCAGAATAG

>lcl|NC_006570.2_cds_YP_170632.1_1501 [gene=obgE] [protein=GTPase ObgE] [protein_id=YP_170632.1] [location=complement(1819701..1820705)]
ATGAGATTTGTAGATGAAGTAGTGATTAAGCTTCAAGCAGGAAAAGGTGGTAATGGTTGTGTGAGTTTCC
GCCGTGAGAAATATGTTCCACGTGGAGGTCCTGATGGCGGTGATGGTGGTAATGGCGGTAGTATCTACCT
AAAAGCAGATGAAAATGTAAACACCTTGATTGATTACCGTTATAAAAGAGAATACTATGCTGAGAATGGT
CGTCCTGGAGAGGGGCGTAATTGTTATGGTAAAGCTGGAGAAGATTTATATCTAGTTGTACCGGTTGGTA
CTAGTGTTTTTGATATTGATACAAATAAAAAAATTGGCGAAGTACTACAGCATGGACAAACCTTTAAGCT
AGTATCAGGTGGTAAAAGAGGTATTGGTAATACACACTTCAAAAGTAGTACAAATCAAGCACCGAGGAAG
TTTACCTTAGGTGAAGAAGGTGAGTACAAAGAAGTTAGACTAGAACTTAATCTGTTAGCTGATGTTGCTT
TATTGGGCTTGCCTAATGCTGGTAAATCAACTCTTATTCGCTCAGTATCTGAAGCAACGCCTAAAGTTGC
TGATTATCCATTTACGACAATGTATCCTCACTTAGGGGTTGTCAAAGTCGGTGTAGATAGTTTTGTAATG
GCAGATATTCCAGGAGTTATTGAGGGTGCTGCTGAAGGTGCTGGTCTTGGACTTAGATTCTTAAAGCATC
TAACTCGAGCTAGGTGTGTATTGCATGTTGTTGATATTTGTCCTTTTAATGAGTCAGATCCTGTTGAGAA
CTATTTTGCTGTAGAAAAAGAACTTGAGAAATATAGTCAAGAATTATTTGATAAACCAAGATTTTTAGTT
ATTAACAAAATTGATCTACTAGCTGATAAAGTTGAGCAAAAATGTCAAGAGTTCGTTGAGCAAATAGGTT
ATCAAGGCAATTACTACACAATATCAGCAGCAATGAAAAAAGGAACAGATGAGTTGGCTAAAAAACTTAA
TGAGTTTTTACAAAAGCAAGAGTAA

>lcl|NC_006570.2_cds_YP_170646.1_1514 [gene=ssb] [protein=single-strand binding protein] [protein_id=YP_170646.1] [location=1839898..1840374]
ATGGCTAAAGGAACTGTAAATAAGGTTATTTTGCTAGGTAGATTGGGTAATGATCCAGAAGTTAGAACTA
CACAAAATGGAACTGTCGTAGCAACTTTAAGTATCGCGACTAATGATGGTATGGGTGAGAATATAACTAC

AGAATGGCATAGAGTAGTTATATTTGGTAAATCTGCTGAAGCCATTCAAAGGTATGCAAATAAAGGTACA
CAAATTTTTGTCGAAGGTCGATTACGCACTAATAAATGGCAAGATAAAAACGGTAATATGCAATATACAA
CTGAAGTAGTTGCGAGTAATTTCCAGTTTATCGGTGGTGGTTCTCAAGGCGGAGCAAATAACCAAAATAC
GCCAAACTTTAATCAGCAACCAAATAATAATTTTAACCAAAATCATCAGCAACCATCAAGACAAGATAAT
ATGCCTGATTTTGCTGAAATTAACTCATCTAATTTTGATGATGATATTCCATTTTAG

>lcl|NC_006570.2_cds_YP_170654.1_1522 [gene=FTT_1763c] [protein=acetyltransferase] [protein_id=YP_170654.1] [location=complement(1851225..1851977)]
ATGAAATCTTTTCTTAAAAAGATTTGGAATATTTTAATGTGGTTAAGGATGACAGCATTTCAGCTTTACT
CTTTTGCAGTAATTGGTGGCTGTAGTATCCTGATAAATATTTTTGCTTTTTTTAAATTGCCTTTATCATG
GAGAATGGCTGTTTGCTATGTATGGACTTATCTTTATTGGATAGGTATGTTAGTCTTTTTACAAGTTTTT
ATTCGTATAACAGGTCGTGTAAATATAGATAAAGATTATCCATGCATCTATGTTTCTAAGCACCAGTCTA
TGCTTGAGACTTTTATGTTTTATGGTTTGCTTGGTAAATGTCATTTTATTATGAAGCAAGAACTTTTTGA
TGCCCCAATTTTTGGTCCTGCTATGAAAAACTTAGGTAGTATTGCTATAGATAGAGACAAACCAAGAGAA
TCCTTAAAAAAAGTTGTCACAGATGGCAAACAGAGTTTGGCTGATGGTATAAATGTGGTTATTTTCCCTG
AGGGGACTAGAGTTAGTGTTGGTGAATACCCAGAATTTCAACGCTCGGCAATGAAATTAGCAGCAGATGC
TAATGTATATATTATTCCTGTTGCACATAATTTCGGTAGATTTTTCCCAAGAAAATGGGGACAAGTCATT
AAACCTGGTATAGCTAGGATGGATTTTGGTAAGAGAATAGATCCTAGAAACTTTGATTCAAAAACGCTAA
CGAGCTATTGTCATAAGGTTATAACTGAGAAAACTAAAGAGTTTAACGGTTAA

>lcl|NC_006570.2_cds_YP_170655.1_1523 [gene=FTT_1764c] [protein=ferredoxin] [protein_id=YP_170655.1] [location=complement(1852140..1852463)]
ATGCCATTTGTAGTTACTGAAAGTTGTATCAAATGTAAATACGGCGATTGCGTAGAAGTATGTCCGGTAG
ATTGTTTCTATGAAGGGCCAAATATGCTAGTAATCAATCCAGATGAGTGTATTGATTGCGCTCTTTGTGA
GCCTGAATGTCCTGTTAATGCGATTAAATCTAGTGATGATTTAAGCGAAAACGAAGAGCAAATGCTTGAT
CTTAACCGAGAACTAGCAGGTATTTGGCCAAATATCGTCGAAAAATGCGAACCTTGTGAAGATGCCGATA
ATTGGGCTTCTGTTCCTGATAAGTTAAAATATTTAGAAAAATAA

>lcl|NC_006570.2_cds_YP_170656.1_1524 [gene=FTT_1765] [protein=hypothetical protein] [protein_id=YP_170656.1] [location=1852683..1853336]
TTGGTTAAAAAAATGAAAGCTGAACTTGATCCGAGAAAACTTATTGCTGGAACAACGATATCAGTAGCTA
TCTTATTTTTTGTTACTTATCTTGTATCTATAGCTGATGGTCATAATACTTTATGTAATCCTTTTATTTC
AGGTTGCTCCGATATTACTCATGCTGGTTTTACTTCATATGAGAAATATATGCTCAAGGCAATTTTAATA
CCAACAGCAACATTGATGGCAGTGATATTCTTTTTTATCCAGAATCTACTTGTACAAATAAGTGATTATA
GCAAAGCGGTAATCAAGCAAGGTAAAGTAATACTTTTTTTAGCATCAGTTGGTTGTATTGGTTTGATAAT
AGGTACGGCTGTGATTGATGGCCAAGATACGCTTATGAATTTACATCTAAAAGCTGTTGCAGTATTTTTT
GTATTAATGAGTATTTGCCAAGTGTGGTACACGATTATTGAATATAGATATTCCTCTAGGTTAAATAAAA
TTCCACTGATATTACGTAGAATCGCAATTTTAATAACAATCGCATTTTCAATATGGTCAGTATTTATGGA
TCAAACAACCGTTAATCATAATATTGTTGAGTGGTGGGGTGTTTATGCACTTATTTTTTGGTTTTGGACA
TTTTCTATAACTAAAATTAAATAG
